# A Simple Predictive Enhancer Syntax for Hindbrain Patterning Is Conserved in Vertebrate Genomes

**DOI:** 10.1371/journal.pone.0130413

**Published:** 2015-07-01

**Authors:** Joseph Grice, Boris Noyvert, Laura Doglio, Greg Elgar

**Affiliations:** The Francis Crick Institute Mill Hill Laboratory, The Ridgeway, Mill Hill, London, NW7 1AA, United Kingdom; Instituto Gulbenkian de Ciência, PORTUGAL

## Abstract

**Background:**

Determining the function of regulatory elements is fundamental for our understanding of development, disease and evolution. However, the sequence features that mediate these functions are often unclear and the prediction of tissue-specific expression patterns from sequence alone is non-trivial. Previous functional studies have demonstrated a link between PBX-HOX and MEIS/PREP binding interactions and hindbrain enhancer activity, but the defining grammar of these sites, if any exists, has remained elusive.

**Results:**

Here, we identify a shared sequence signature (syntax) within a heterogeneous set of conserved vertebrate hindbrain enhancers composed of spatially co-occurring PBX-HOX and MEIS/PREP transcription factor binding motifs. We use this syntax to accurately predict hindbrain enhancers in 89% of cases (67/75 predicted elements) from a set of conserved non-coding elements (CNEs). Furthermore, mutagenesis of the sites abolishes activity or generates ectopic expression, demonstrating their requirement for segmentally restricted enhancer activity in the hindbrain. We refine and use our syntax to predict over 3,000 hindbrain enhancers across the human genome. These sequences tend to be located near developmental transcription factors and are enriched in known hindbrain activating elements, demonstrating the predictive power of this simple model.

**Conclusion:**

Our findings support the theory that hundreds of CNEs, and perhaps thousands of regions across the human genome, function to coordinate gene expression in the developing hindbrain. We speculate that deeply conserved sequences of this kind contributed to the co-option of new genes into the hindbrain gene regulatory network during early vertebrate evolution by linking patterns of *hox* expression to downstream genes involved in segmentation and patterning, and evolutionarily newer instances may have continued to contribute to lineage-specific elaboration of the hindbrain.

## Introduction

Conserved non-coding elements (CNEs) have long been implicated in the transcriptional control of embryogenesis. CNEs act as tissue-specific enhancers in functional assays [1,2,3,4], are necessary for normal development [5,6]and are subject to stronger purifying selection than coding sequences in human populations [7]. However, the mechanisms by which CNEs regulate gene expression and the sequence features that encode their functions remain poorly understood. Indeed, several recent reviews [8,9,10]call for the need to elucidate grammars encoded within *cis*-regulatory elements in order to understand the evolution of adaptive morphologies and the ontogeny of developmental disorders.

The hindbrain is a structure unique to vertebrates composed of 7 segments known as rhombomeres (r1-7). Many studies have elucidated a number of genes and regulatory elements that form the gene regulatory network (GRN) underlying hindbrain segmentation conserved amongst all vertebrates [11] (reviewed in [12]). Normal hindbrain development is dependent upon nested patterns of HOX expression of paralogous groups 1–4, the proper interpretation of which requires the HOX cofactors PBX and MEIS/PREP. Loss of HOX, PBX, or MEIS function leads to failure to form boundaries between, and homeotic transformations of, rhombomeres [13,14,15,16], demonstrating the essential roles of these factors in both hindbrain segmentation and patterning. Presumably, HOX proteins and their cofactors activate unique repertoires of downstream genes in each segment, subsequently giving rise to the individual identities of rhombomeres and their derivatives. This necessitates the existence of segment specific genes and HOX-dependent regulatory elements coordinating their expression. However, segment-specific genes targeted directly by HOX proteins are undetermined and the regulatory elements that choreograph these patterns have yet to be characterised. Furthermore, the corresponding region of the central nervous system (CNS) in invertebrates is not segmented and accordingly lacks overlapping expression patterns of key segmentation genes such as *egr2* (*krox20*) and *mafb* (*kreisler*/*valentino*), raising questions about how the GRN controlling hindbrain segmentation arose in early vertebrates [17,18,19].

PBX, HOX and MEIS/PREP factors form trimers at enhancers in order to activate transcription of their target genes. PBX-HOX heterodimers bind to inseparable half-sites with the consensus TGATNNAT [20], whereas MEIS/PREP proteins bind the consensus CTGTCA at proximal, but potentially gapped and/or inverted, sites [21]. A number of vertebrate CNEs containing PBX-HOX binding motifs can act as hindbrain enhancers [22], and these may mediate the activation of genes by specific HOX proteins to activate segment-restricted expression. Furthermore, MEIS/PREP motifs were recently used as part of a classifier predicting hindbrain regulatory elements [23], though it was unclear to what extent these motifs contributed to the accuracy of predictions. Searching for incidences of these binding motifs in putative enhancers might identify additional transcriptional targets of HOX proteins and further embellish our knowledge of the downstream GRN involved in hindbrain patterning.

In this study, we further investigate the connection between vertebrate CNEs and hindbrain patterning. Initially, we identify novel hindbrain enhancers predicted by the presence of conserved PBX-HOX motifs. We then find that MEIS/PREP consensus sites are present in hindbrain positive (hb+) CNEs and absent from hindbrain negative (hb-) CNEs. We further define the grammar of these putative sites by phylogenetic footprinting. We confirm this correlation by analysing a set of CNEs from the CONDOR database [24]containing conserved co-occurrences of these motifs (syntax) to accurately predict hindbrain enhancer activity during functional assays. We use site-directed mutagenesis to demonstrate the necessity of these sites for robust and reproducible patterns of expression in the hindbrain. Finally, we analyse the occurrence of these motifs within 210Mb of conserved sequence from the human genome. We demonstrate that occurrences of this motif syntax are enriched around genes encoding developmental transcription factors, and also observe significant overlap with functionally determined human hindbrain enhancers from the VISTA enhancer browser[25]. We hypothesise that the hindbrain enhancers identified here are essential nodes in the hindbrain gene regulatory network, controlling the expression of sub-region specific genes that ultimately generate segmental identity. We suggest that this supports a model where the gain of these enhancers during early vertebrate evolution contributed to the elaboration of this region of the CNS and acquisition of hindbrain segmentation.

## Results

### Conserved PBX-HOX motifs are poor predictors of hindbrain enhancers

**Fig 1:**
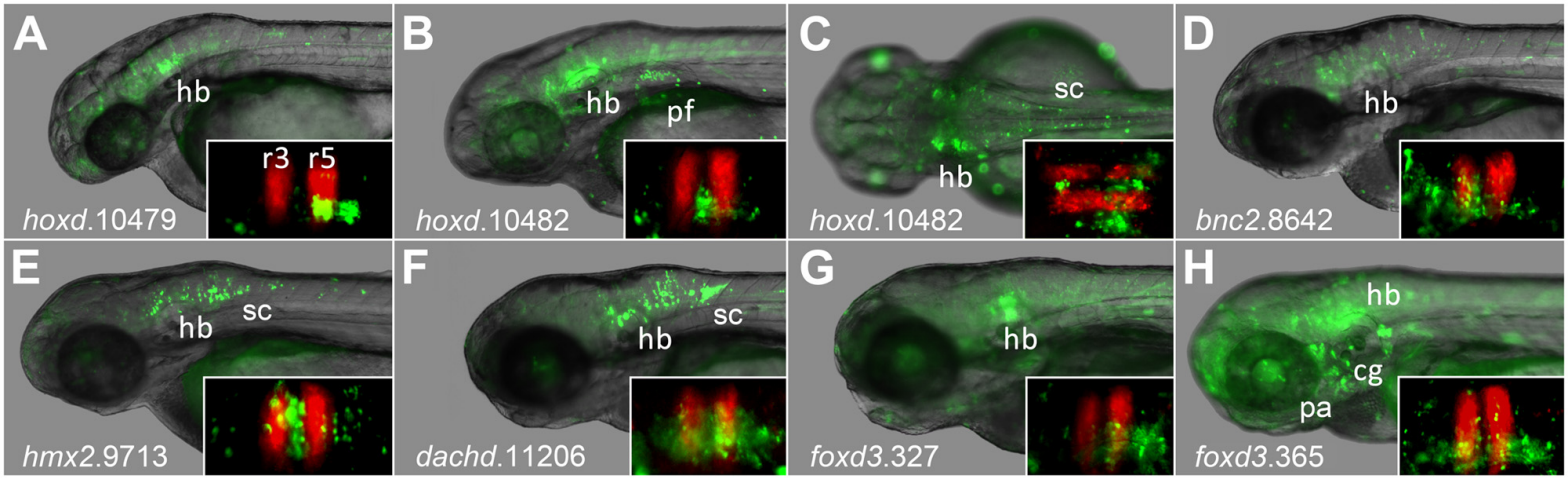
CNEs containing PBX-HOX motifs driving expression in hindbrain of transgenic zebrafish. Images show expression driven during transient transgenic assays (see methods) between 2 and 3 dpf. Insets show comparison with RFP in rhombomeres 3 and 5. *hoxd*.10479 (A) at ~42 hpf in ventral r5r6; hoxd.10482 lateral (B) and dorsal (C) views at ~56 hpf in lateral r2, r4, r6, pectoral fin and spinal cord; *bnc2*.8642 (D) at ~60 hpf in hindbrain; *hmx2*.9713 (E) at ~60 hpf in hindbrain and spinal cord; *dachd*.11206 (F) at ~72hpf in hindbrain and spinal cord; *foxd3*.327 (G) at ~72hpf in ventral r5 and r6; *foxd3*.365 throughout the central nervous system, most strongly in the ventral hindbrain, and cranial ganglia. fb: forebrain; mb: midbrain; hb: hindbrain; sc: spinal cord; pf: pectoral fin; pa: pharyngeal arches/neural crest; cg: cranial ganglia; r3 r5: rhombomeres 3 and 5.

With the aim of identifying novel hindbrain enhancers, we searched for instances of human:zebrafish conserved PBX-HOX motifs (TGATNNAT) within CNEs stored in the CONDOR database[24]. We identified 465 instances of the motif in 394 CNEs and selected a subset from across different loci for functional characterisation. 29 zebrafish sequences were initially assayed using an EGFP reporter in wild-type or KROX20:RFP zebrafish embryos, which express RFP in r3r5 (see methods). However, just 7 of these (24%) were considered by our criteria (see methods) to act as hindbrain enhancers (Fig 1). Four CNEs appear to be active in domains encompassing large portions of the hindbrain at these time-points (bnc2.8642, dachd.11206, foxd3.365, hmx2.9713) whilst the remaining three activated patterns of reporter expression restricted to certain regions of the hindbrain (foxd3.327 in ventral r5-6, hoxd.10479 in ventral r5-6 and hoxd.10482 in r4 and r6). The tissue specificities of all 29 elements can be found in S1 Table.

The enrichment for hindbrain enhancer activity detected in this set was considerably lower than that previously reported for a set of sequences tested on the basis of similar criteria (12/21, 57%) [22], indicating that sequence context might be important in determining the functionality of PBX-HOX motifs. We reasoned that those CNEs driving expression in hindbrain (hb+) might be distinguished from CNEs that did not (hb-) in their sequence content, perhaps representing binding sites for accessory factors.

### Conserved hindbrain enhancers contain both PBX-HOX and MEIS/PREP motifs

We searched for sets of enhancers from zebrafish tested using the same functional assay used here (see methods). We found two studies [22,26] matching our criteria and added these sequences to the 29 we had functionally tested. In total, there are 188 sequences, 38 of which are considered to be hb+. Those that comprise the hb- set are functionally heterogeneous, displaying either no enhancer activity at all or patterns of expression that do not include the hindbrain. The hb+ and hb- training sets can be found in S1 and S2 Texts, respectively.

To ascertain whether the two sets are enriched for different motifs, we derived over-represented position weight matrices (PWMs) *de novo* from each set using MEME[27]. We then compared each discovered PWM to known TF binding preferences stored in three online databases[28,29,30] using two different alignment algorithms[31,32](see methods).

**Fig 2:**
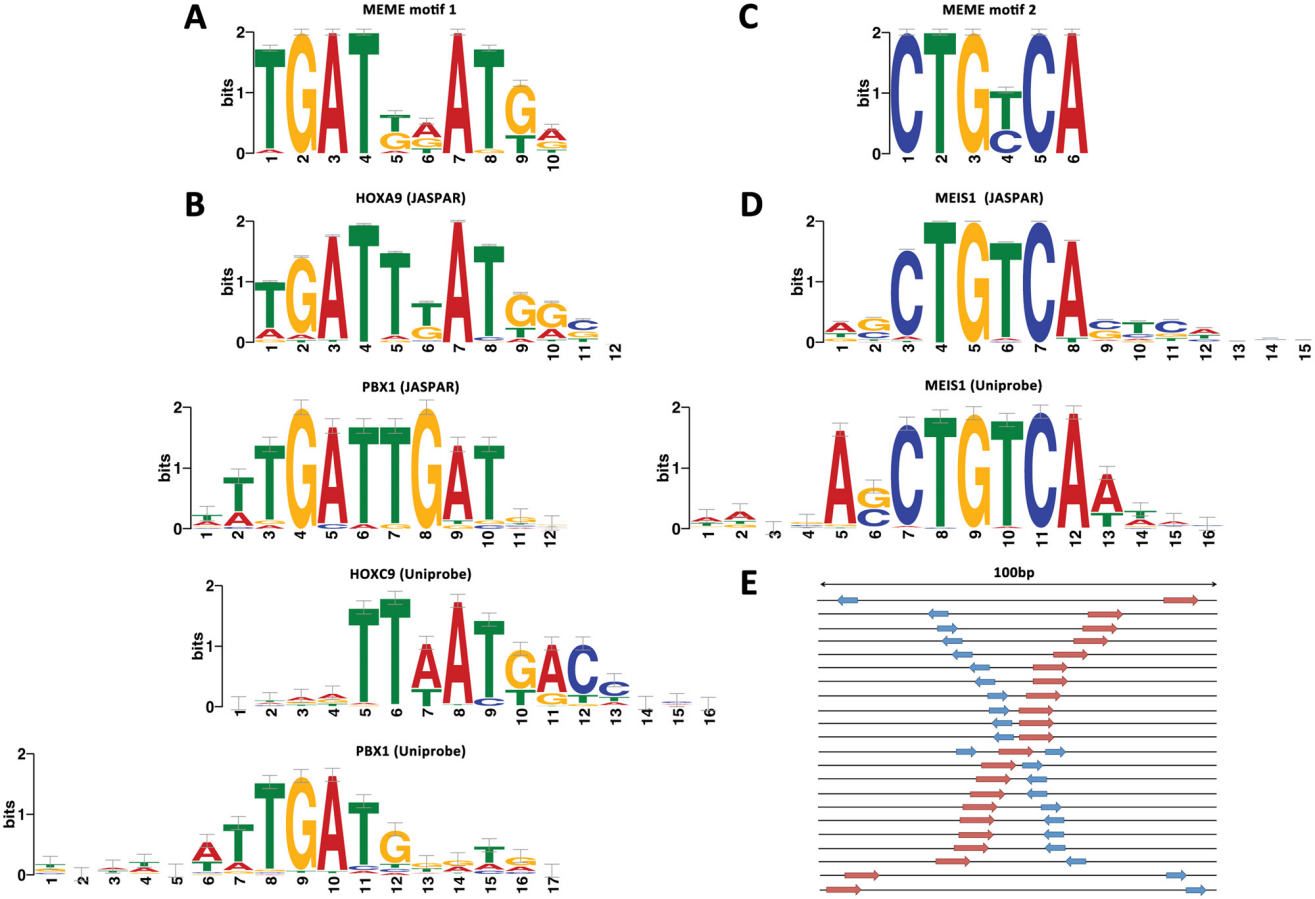
Hindbrain enhancers typically contain both PBX-HOX and MEIS/PREP motifs within 100bp. Motif discovery on a set of 38 hindbrain enhancers using MEME detects two enriched motifs. The first (A) resembles PBX and HOX binding preferences (B) and the second (C) resembles MEIS and PREP binding preferences (D). Furthermore, in all but one case these motifs occur within 100bp in positive enhancers, schematically represented in (E).

Reassuringly, the most significant PWM in the hb+ set (TGATDDATKD) is very similar to the PBX-HOX motif used to select most of the sequences (TGATNNAT) (Fig 2A). This PWM also closely resembles known *in vivo* PBX-HOX heterodimeric binding sites[33,34,35] and aligns well to experimentally determined binding preferences for PBX and HOX monomers (Fig 2B). A second PWM with the consensus CTGYCA was also discovered (Fig 2C). This PWM closely resembles MEIS, PREP and TGIF homeodomain binding sites and aligns well to published preferences for these factors (Fig 2D). We found neither motif to be over-represented in the hb- set or shuffled sequence controls. The PWMs discovered from the hb+ set can be found in S3 Text.

Next, we used two motif searching algorithms, FIMO[36] and MCAST{Grant, 2011 #452 to detect co-occurrences of the two discovered PWMs in both the hb+ and hb- sets (see methods). We detect enrichment of co-occurrences in the hb+ versus the hb- set using either method. FIMO detects co-occurrences in 25/38 hb+ sequences and 49/150 hb- sequences (Fisher's exact test p = 2.2×10^–4^). MCAST detects co-occurrences in 15/38 hb+ sequences and 7/150 hb- sequences (Fisher's exact test p = 1.8×10^–7^) reflecting a stricter match threshold. These data demonstrate that hb+ CNEs more commonly contain instances of MEIS/PREP motifs in addition to PBX-HOX motifs than hb- CNEs.

Finally, we searched the literature for studies on hindbrain enhancers to check for the presence of these motifs. PBX-HOX and MEIS/PREP motifs are necessary for hindbrain enhancer activity driven by a number of elements, for example mouse *hoxb1*, *hoxb2* and *hoxa3* regulatory elements in rhombomere 4 {Popperl, 1995 #587}[21,34,37,38,39,40], a *hoxa3* regulatory element in r5r6[39], an *egr2* enhancer (element C) for activation in r3-5[41,42,43], and a *meis2* enhancer (CNE 3299) for activation in r3-4 and neural crest[22]. In all these cases, the distance between sites is less than 20bp. Therefore, our bioinformatically identified signature appears to be consistent with several *in vivo* studies of hindbrain enhancers.

### MEIS/PREP motifs are frequently proximal to PBX-HOX motifs in hindbrain enhancers

In order to assess the grammar of the sites, we examined the conservation and distribution of PBX-HOX and MEIS/PREP motifs in both the hb+ and hb- sets using phylogenetic footprinting (see methods). 22 hb+ and 33 hb- sequences from our training sets have entries in the CONDOR database[24]. We extracted sequences from 25 vertebrate genomes stored in CONDOR to build multiple alignments using ClustalW2[44]. We then searched for incidences of conserved PBX-HOX and MEIS/PREP binding site motifs in the multiple alignments, and calculated a maximum distance between the two sites for each orthologue. Of the 22 hb+ sequences, all but one contains instances of both PBX-HOX and MEIS/PREP motifs within 100bp of one another in zebrafish. In fact for 19/22 alignments the motifs are separated by <50 bp and in 16/22 alignments by <25 bp (Fig 2C). In contrast, the 33 hb- sequence alignments all contain conserved PBX-HOX motifs, but only 4 contain conserved MEIS/PREP motifs. In 3 cases these are >100 bp from the PBX-HOX motif but in 1 case the two motifs are <25 bp apart. The alignments of the hb+ sequences can be found in S1 File. Since co-occurrences of PBX-HOX and MEIS/PREP motifs within 100bp have a strong correlation with hindbrain enhancer activity (21/22 hb+ sequences, 1/33 hb- sequences, Fisher's exact test p = 1.5×10^–12^), we decided to use this motif structure to predict additional hindbrain enhancers in CNEs.

### The presence of PBX-HOX and MEIS/PREP motifs is highly correlated with hindbrain enhancer function

The presence of MEIS/PREP motifs in the hb+ set suggests that these are acting together with PBX-HOX motifs and contributing to the enhancer activity observed during functional assays. Furthermore, our observations on the distribution of sites suggest that proximity might also be important for function. To test this, we used FIMO[36] to detect PBX-HOX and MEIS/PREP motifs within 100bp of each other in a set of 6691 CNEs in CONDOR[24] (S2 Table) and confirmed their conservation in other species using multiple alignments (S2 File). In total, we identified and functionally tested 75 additional CNEs matching our criteria in the zebrafish genome and checked for hindbrain expression at 2 and 3 dpf (see methods).

**Fig 3:**
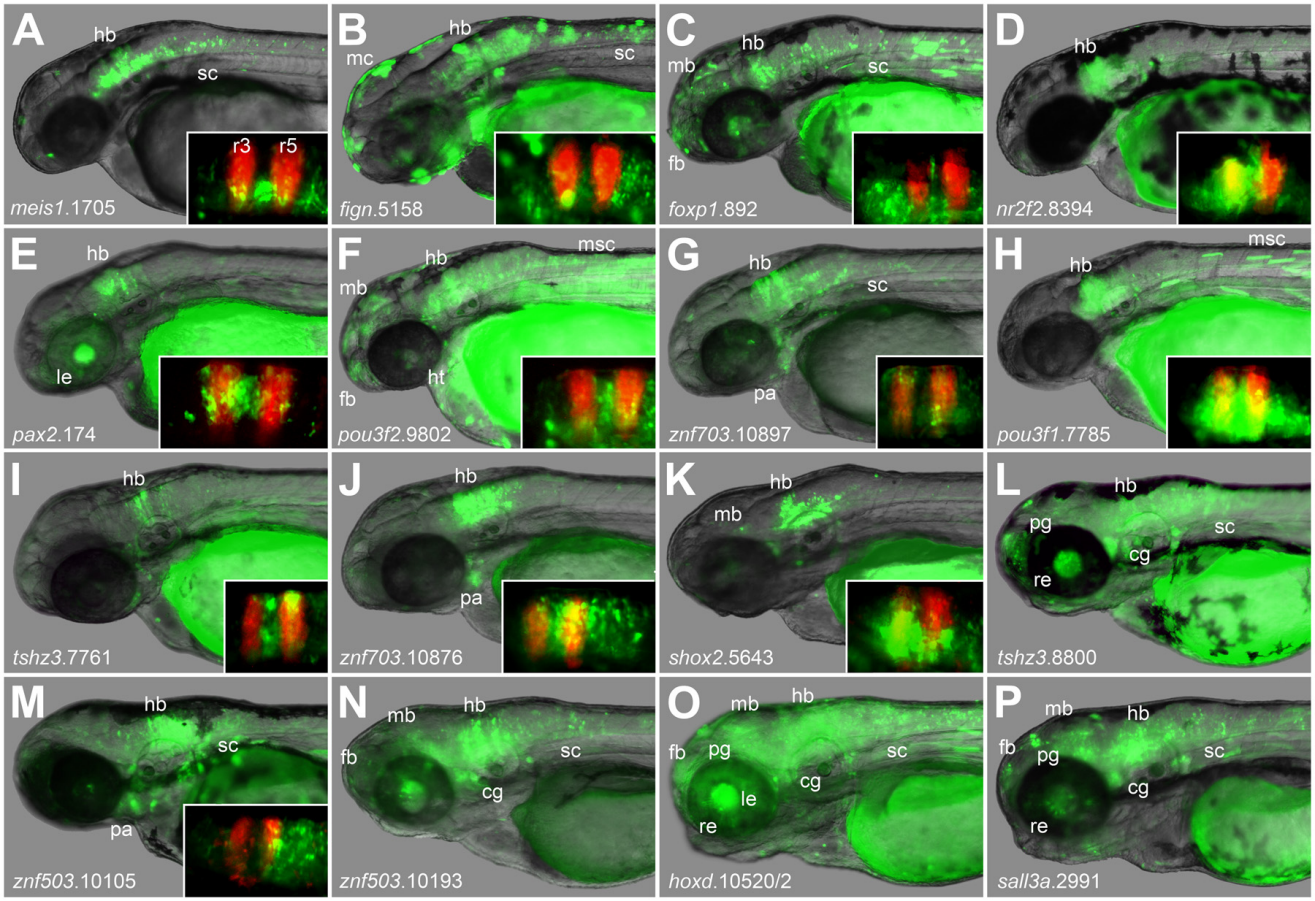
Conserved PBX-HOX and MEIS/PREP motifs predict hindbrain enhancers accurately. Images show expression driven during transient transgenic assays (see methods) between 2 and 3 dpf. Insets show comparison with RFP in rhombomeres 3 and 5. Constructs drive expression in hindbrain and in other tissues *meis1*.1705 (A) in hindbrain and spinal cord; *fign*.5158 (B) in hindbrain, spinal cord and melanocytes; *foxp1*.892 (C) in the central nervous system; *nr2f2*.8394 (D) in hindbrain; *pax2*.174 (E) in hindbrain and lens; *pou3f2*.9802 (F) in the central nervous system, heart and muscle; *znf703*.10897 (G) in hindbrain, spinal cord and pharyngeal arches/neural crest; *pou3f1*.7785 (H) in hindbrain; *tshz3*.*1*.7761 (I) in hindbrain; *znf703*.10876 (J) in hindbrain and pharyngeal arches/neural crest; *shox2*.5643 (K) in hindbrain; *tshz1*.8800 (L) in hindbrain, spinal cord, retina, pineal gland and cranial ganglia; *znf503*.10105 (M) in hindbrain, spinal cord and pharyngeal arches; *znf503*.10193 (N) in the central nervous system and cranial ganglia; *hoxd*.10520/1 (O) in the central nervous system, lens, retina, pineal gland and cranial ganglia; and *sall3a*.2991 (P) in the central nervous system, retina, pineal gland and cranial ganglia. fb: forebrain; mb: midbrain; hb: hindbrain; sc: spinal cord; pa: pharyngeal arches/neural crest; cg: cranial ganglia; mc: melanocytes; msc: trunk muscle cells; le: lens; re: retina; pg: pineal gland.

84% (63/75) of these CNEs are active in hindbrain at 2 days post-fertilisation (dpf), whilst 59% (44/75) are active at 3dpf. Overall, 89% (67/75) of CNEs display robust hindbrain enhancer activity according to our criteria (see methods) at one or both time points. Furthermore, for 55/75 CNEs, hindbrain is the predominant region of expression at one time point. These enhancers generate a variety of different expression patterns (Fig 3 and S3 Table), suggesting that they contain a range of different binding sites that modulate the function of the PBX-HOX and MEIS/PREP motifs. The coordinates of all the CNEs assayed in this study are available in S4 Table.

These results demonstrate that conserved co-occurrences of PBX-HOX and MEIS/PREP motifs are strongly associated with significant hindbrain enhancer activity, lending support to the model that these motifs constitute a hindbrain enhancer syntax.

### PBX-HOX and MEIS/PREP motifs are necessary for hindbrain enhancer function

We next aimed to show that intact instances of the PBX-HOX-MEIS/PREP syntax are required for the function of these hindbrain enhancers. We selected four enhancers from different loci with a variety of segment specificities. We mutated each motif separately to test their ability to drive reporter expression independently (see methods).

**Fig 4:**
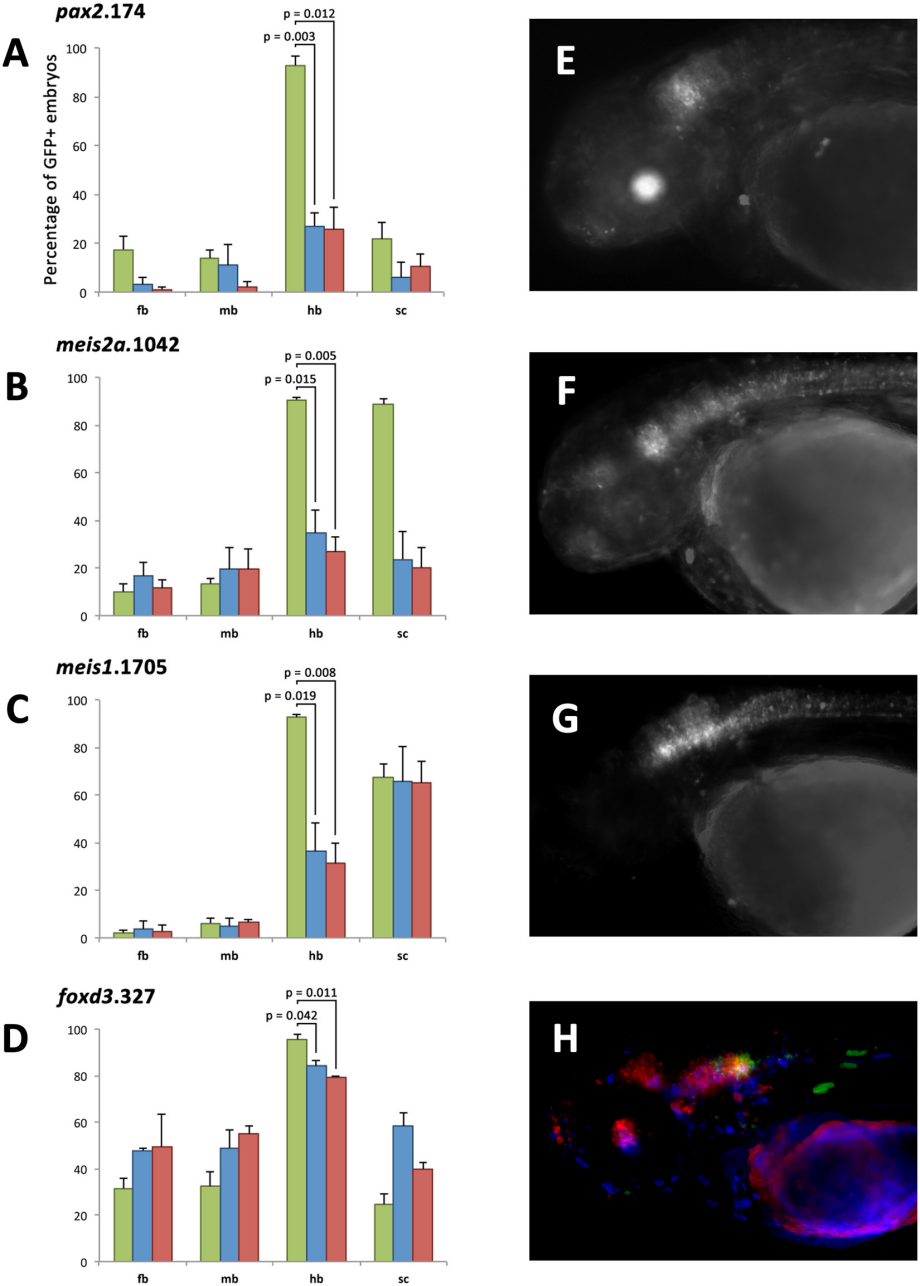
The PBX-HOX and MEIS/PREP motifs of four enhancers are essential for their function. Histograms for four elements, *pax2*.174 (A), *meis2a*.1042 (B), *meis1*.1705 (C) and *foxd3*.327 (D), showing the number of embryos with GFP positive cells in forebrain (fb), midbrain (mb), hindbrain (hb) and spinal cord (sc) when expressing wild-type (green), MEIS/PREP site mutant (blue) or PBX-HOX site mutant (red) constructs. Annotation displays p values for one-tailed paired t tests. All mutant constructs show a significant (student's t test p = <0.05) reduction in the number of embryos positive for hindbrain. Wild-type *pax2*.174 (E) drives expression in hindbrain and lens (green), whereas mutant constructs do not drive this pattern. Wild-type *meis2a*.1042 (F) drives expression in the central nervous system particularly the anterior hindbrain. Mutant constructs fail to recapitulate this expression. Wild-type *meis1*.1705 (G) drives expression in the hindbrain and spinal cord, but mutant constructs drive expression only in spinal cord. Wild-type *foxd3*.327 (H) drives expression in posterior hindbrain (green), but expression driven by constructs where either the MEIS/PREP (blue) or the PBX-HOX (red) motif is mutated is frequently ectopic in midbrain, anterior hindbrain and spinal cord.

In all cases, mutating either the PBX-HOX or MEIS/PREP motif results in a significant reduction in hindbrain enhancer activity (Fig 4A–4D), demonstrating that both motifs are required for efficient hindbrain enhancer function. In the first three cases (*pax2*.174, *meis2*.1042 and *meis1*.1705) we observe a reduction in hindbrain expression from ~90% of embryos in wild-type (wt) constructs to ~30% in the mutant constructs. Furthermore, embryos expressing mutant constructs have very few GFP positive cells, in contrast to the robust and widespread pattern driven by the wt construct (Fig 4E–4G). For the fourth element (*foxd3*.327) there is only a modest reduction in hindbrain activity in both mutants, from ~90% to ~80%, but we also observe a loss of specificity of expression driven by mutant constructs. The wt element is restricted to rhombomeres 5 and 6 whereas mutant constructs drive expression more frequently in midbrain, anterior hindbrain and spinal cord (Fig 4D and 4H).

These data indicate that these four distinct elements each require an intact instance of the hindbrain enhancer syntax to re-capitulate the expression patterns of the wt enhancers, and that removing the components of the hindbrain syntax leads to either a loss of expression (for *pax2*.174, *meis2a*.1042 and meis1.1705) or a loss of specificity (for *foxd3*.327).

We also tested 8 CNEs from the *meis2a* locus that contain only one of the two motifs (PBX-HOX or MEIS/PREP). This gene was chosen because it is known to be strongly expressed in the hindbrain and a number of hindbrain enhancers have been identified from this region[22]. We found that despite their association with a known hindbrain gene, only 2 of these elements generate an appreciable level of hindbrain expression. None have their expression limited to hindbrain, although they often drive reporter expression specifically in other tissues, such as midbrain, retina, cranial ganglia and spinal cord (S1 Fig and S5 Table).

### Using the hindbrain syntax to predict putative hindbrain enhancers genome-wide

**Fig 5:**
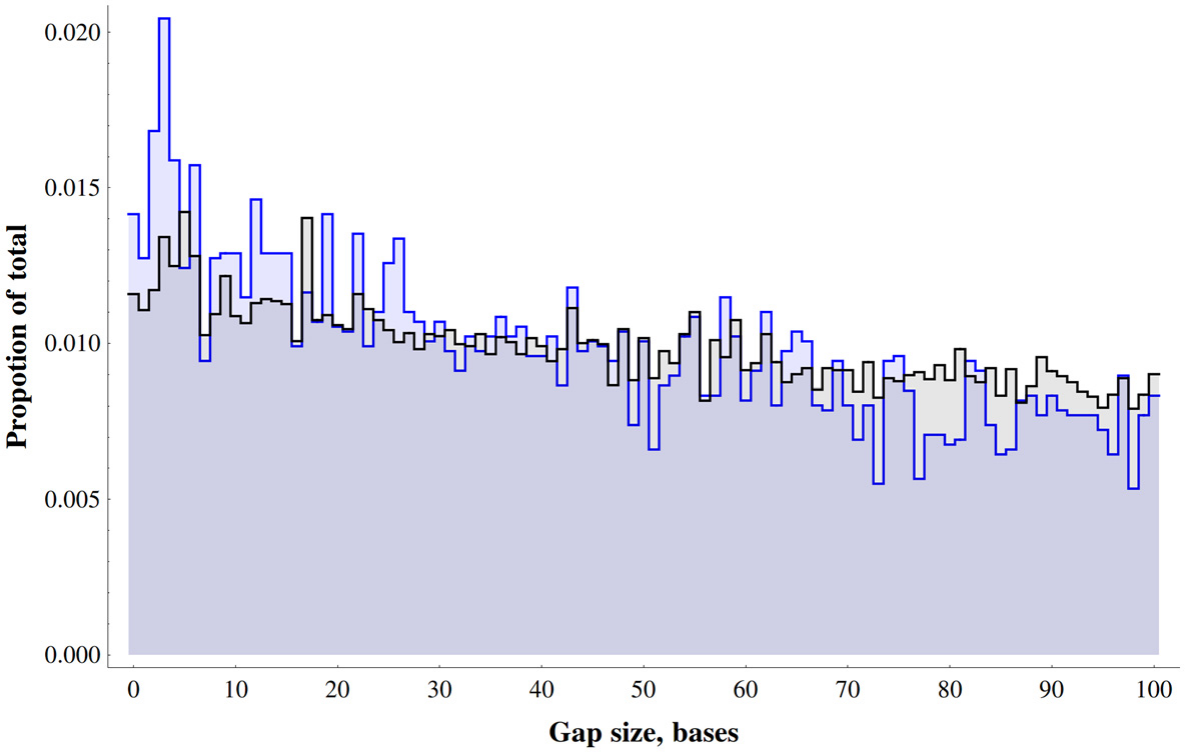
Distances between PBX-HOX and MEIS/PREP motifs across the whole human genome. Graph shows the distribution of gaps between PBX-HOX and MEIS/PREP motifs across the whole genome (black) and human conserved regions defined by GERP (blue).

Next we examined whether our simple hindbrain syntax is sufficiently discriminative to predict signatures of hindbrain enhancers genome-wide. To this end, we identified all co-occurrences of canonical PBX-HOX (TGATNNATKR) and MEIS/PREP (CTGTCA) motifs with a gap size between zero and 100 bp (Fig 5) across the entire human genome. We then selected those instances that fall within 210Mb of conserved sequence in the genome defined using GERP [45]. We identified 31,081 pairs of sites across the whole (soft-masked) genome, with 6,360 of these in GERP regions (S6 Table), representing a 1.5 fold enrichment for these co-occurrences in conserved portions of the genome. There is also a clear bias towards very short distances between the two motifs, because the real number of elements with a gap of 40bp or lower is much higher than the number found in simulated data (S2 Fig, p = 6.0×10^–25^); this is consistent with the fact that over 75% of the hb+ elements we functionally tested have a gap of less than 40bp between motifs. This enrichment for short gap size is clearer in, but not exclusive to, the conserved portion of the genome as might be expected for developmental enhancers orchestrating hindbrain patterning.

In line with our experimental data, we extracted all 3,122 instances of hindbrain syntax where the gap between motifs is less than or equal to 40bp (henceforth ‘hb_40 elements’) from the GERP conserved portion of the human genome (S6 Table) and assessed their suitability as hindbrain enhancers. hb_40 elements occur at a frequency which is 3.28 times higher (per Mb) within functionally annotated hindbrain enhancers from the VISTA Enhancer Browser[25] than in all GERP regions (Table 1). Furthermore, 394/3122 hb_40 elements are located in a set of 42,144 previously predicted hindbrain enhancers[23], 2.54 times as many as expected by chance (Table 1). Using a hypergeometric test, hb_40 elements are significantly enriched within VISTA annotated hindbrain enhancers with respect to all GERP regions (observed: 21, Expected: 1.4, hypergeometric test p = 4.0×10^–18^) and the full set of VISTA enhancers (observed: 21; expected: 8; hypergeometric test p = 9.6×10^–6^).

**Table 1:** Number of hb_40 elements per Mb in different types of sequence.

Region type	Total size	Total number of hb_40 elements	Number of hb_40 elements per Mb
GERP regions	210786169	3122	14.8
All Vista enhancers	3076652	60	19.5
Positive Vista enhancers	1733795	39	22.5
Hindbrain enhancers from Burzynski et al.	10455007	394	37.7
Hindbrain positive Vista enhancers	452387	22	48.6*
CNEs	776585	111	142.9

GERP regions, CNEs, enhancers, positive enhancers, positive hindbrain enhancers.

* This figure is artificially low compared with the CNEs, as the Vista enhancers have long flanking regions (an average size of 1,779bp compared with 116bp for CNEs). In terms of numbers, 21/238 hb+ Vista enhancers (11.34%) have hb_40 syntax compared with 93/6691 CNEs (1.4%).

The distribution of hb_40 elements across the human genome is non-uniform as demonstrated by enrichment for certain gene ontology (GO) terms associated with co-localised genes. The top ten clusters include GO terms relating to two broad functional classes: transcription factor activity and developmental patterning (S7 Table). Furthermore, of the 3,122 hb_40 elements, 93 are found within (human) CNEs in CONDOR (75 of which are conserved through to zebrafish and have been functionally assayed in this study) with a further 59 located within 1kb, and 118 located within 10kb, of a CNE (S3 Fig). Even if we remove the 93 cases found within CNEs, hb_40 elements remain clustered in a non-random fashion across the genome and are more likely to be located near developmental transcription factor genes (see methods).

The majority of the hb_40 elements occur in intergenic regions and introns. Of the 3122 hb_40 elements: 1071 occur in introns and may be intronic regulatory elements; 156 overlap exons, so may perform a dual coding/regulatory function; 10 occur within 1kb of the most 5’ transcription start site, suggesting that these occur within promoters; and the remaining 1885 are in intergenic regions and may be distal enhancers.

It remains possible that the association between this syntax and hb enhancer activity is not limited to elements with a distance of 40bp or less. We performed the tests again using various distances up to 100bp and found that all the tests still provided significant results. However, we believe that hb_40 syntax in particular, in line with our experimentally defined hindbrain enhancers, represents a simple hindbrain enhancer predictor with both good sensitivity and more importantly high specificity.

## Discussion

### A common core syntax unites functionally heterogeneous hindbrain enhancers

Here, we have identified a simple syntax within functionally validated hindbrain enhancers, consisting of the spatially restricted co-occurrence of conserved PBX-HOX and MEIS/PREP binding motifs, that predicts hindbrain enhancers with high accuracy. Furthermore, the individual components of this syntax are required for the ordinary function of these enhancers Proximal PBX-HOX and MEIS/PREP motifs have been implicated in hindbrain enhancer function in a number of individual cases, most commonly at HOX auto- and cross- regulatory elements but also at *egr2* element C. These are likely to be elements mediating the establishment of *hox* and *egr2* expression patterns, ultimately responsible for organising the initial segmentation of the hindbrain. Our use of these motifs to predict hindbrain enhancers and our subsequent mutagenesis experiments confirm this known association in a larger number of examples. The association of these CNEs with developmental regulatory genes identifies putative HOX targets, suggesting that these may lie directly downstream of the collinear HOX code in all vertebrates, with the possible exception of the lamprey. Furthermore, our identification of this signature in human GERP regions provides a resource for those wishing to study the function of human hindbrain enhancers.

There are many differences between our approach and that used by Burzynski *et al*. [23]. Firstly, the set of hindbrain enhancers we used for motif discovery were highly conserved and directed restricted expression in the hindbrain, suggesting that these enhancers play a role in patterning and segmentation. In contrast, Burzynski *et al*. began with a more heterogeneous set of enhancers, perhaps reflecting their lower conservation and more heterogeneous functions. Our model consisted of two simple motifs identified *de novo*, whereas Burzynski *et al*. used machine learning to apply hundreds of motifs predictively to the human genome. Whilst this approach takes advantage of a large number of motifs and identifies thousands of putative hindbrain enhancers, the contribution of each motif and the underlying grammar of motif pairs are difficult to assess. In contrast, our simple model composed of two functionally defined motifs allows us to assess their spatial relationships easily and perform informative mutagenesis experiments.

Recent ChIP-seq data for MEIS1, PBX1 and PREP1 consistently identify both the TGATNNAT and CTGTCA motifs as over-represented, suggesting that in the majority of cases these factors are binding as trimers with HOX and other PBX, MEIS, and PREP family members[46]. Furthermore, both PBX-HOX and MEIS/PREP motifs are enriched within HOXA2 Chip-seq peaks[47]. This indicates that these motifs recruit the appropriate factors, suggesting that the elements described here are likely to be bound by PBX, HOX and MEIS/PREP proteins *in vivo*.

There are several documented cases of these binding sites being critical for the normal function of hindbrain enhancers from different genes. For example, mutating a PBX-HOX and MEIS/PREP motif pair in a r3-4 specific enhancer from the *meis2* locus leads to ectopic expression crossing both the anterior (r2/3 interface) and posterior (r4/r5 interface) expression boundaries [22]. It therefore appears that these binding sites impart some degree of segment specificity to enhancers that contain them, though additional sites may be present that further define the observed expression [22]. Our findings are consistent with these previously published data. PBX-HOX and MEIS/PREP motif pairs in 4 enhancers from different genes are essential for their ordinary function, despite the fact that they each have distinct segment specificities. This suggests that each enhancer uses the common cofactors PBX and MEIS/PREP, but can bind particular HOX proteins in order to achieve specificity.

In our original set of functional enhancers, the two motifs contributing to our model always occurred within 100bp. Within this range, however, there was considerable variability, but a bias towards shorter distances, indicating that proximity may be important for function. We did not observe any periodicity of distances, as has been reported previously for motifs mediating co-binding in drosophila enhancers[48].

We note a variety of tissue specificities present in these elements aside from hindbrain (S1, S3 and S5 Tables). Each element has its own tissue unique tissue specificity profile reflective of their distinct sequences and independent evolution. We observe a large proportion of elements that express in forebrain, midbrain, spinal cord and neural crest/pharyngeal arches. This is consistent with the expression of PBX and MEIS/PREP proteins throughout the CNS and the expression of HOX proteins posterior of the r1/r2 boundary in the hindbrain and spinal cord (ZFIN gene expression database), and the known roles of these factors in patterning these tissues. That we discover enhancers of other tissues is not surprising since our model was only designed to identify hindbrain enhancers, and was not designed to exclude enhancers of other tissues.

CNEs have an average length of 112bp, and share >60% identity across this length, and the average length of PCR products assayed in this study is 324bp. This suggests that additional binding sites are present which contribute to the observed heterogeneity in function. Even focusing on patterns of expression within the hindbrain, a propensity for spatio-temporal variation amongst the set of enhancers is evident. This may reflect the diverse roles of these elements during hindbrain development. Potential roles include orchestrating the establishment of rhombomere-specific gene expression (for anteroposteriorly restricted elements), progenitor pool maintenance (for earlier and medially restricted elements), and neurogenesis/axonal targeting (for later and laterally restricted elements).

PBX-HOX heterodimeric binding sites are known to be inseparable [20], but PBX-MEIS heterodimers can form on gapped or inverted sites [21]. Our observations on the distribution of sites within functional enhancers are consistent with known data on the formation of PBX, HOX and MEIS/PREP heterotrimers. The position of the MEIS/PREP site with respect to the PBX-HOX varies greatly between the enhancers in the set. Whilst site distance and orientation of sites is variable amongst the set as a whole, orthologous enhancers conserve these parameters. Whether this is somehow important to the functions of particular CNEs will require further investigation. Through the introduction of insertions, deletions and/or inversions to these enhancers, the roles of site spacing, order and orientation may be explored in more detail. Furthermore, experiments of this kind will distinguish between the “enhanceosome” and “billboard” models of enhancer function with respect to deeply conserved non-coding elements. Finally, a knowledge of the grammar within regulatory elements allows some assessment of the likely functional consequences of variants or mutations at specific positions within these sequences, particularly in relation to phenotype and disease.

### Acquisition of HOX-dependent cis-regulatory elements in the vertebrate lineage

Previous work made an overt connection between CNEs and hindbrain development for the first time [22]. Here we expand this dataset to include over 70 conserved hindbrain enhancers. Although originally identified in mammal-fugu alignments [2,49], thousands of CNEs are also identifiable between human and other gnathostome genomes, including chicken, xenopus, zebrafish and chimaera [50,51,52]. Fugu CNEs map to ~0.8Mb of the human genome; a proportion of this has also been identified in the lamprey, mapping to ~27kb of the human genome[4,53]. Far fewer conserved elements are detectable, even at lower identity, in invertebrate chordates [54,55] and beyond [56,57], suggesting that nearly all mammal-fugu CNEs, at least in their present forms, are either vertebrate or gnathostome novelties. In accordance with this, the hindbrain enhancers discovered herein are distributed across the breadth of gnathostomes, but very few are detectable in lamprey and none are detectable in protochordates. This correlates well with both the acquisition of hindbrain segmentation (stem jawless vertebrates[11]) and the elaboration of hindbrain morphology (stem gnathostomes), suggesting that the conserved enhancers reported here might have played a role in this acquisition and elaboration.

The existence of large sets of CNEs identifiable within, but not between, particular lineages[58,59] has lead to the supposition that these control phylotypic aspects of embryogenesis of the group concerned[60,61]. Molecular evidence also supports this idea. For example, genes expressed most highly at the vertebrate phylotypic stage tend to associate with more CNEs than those expressed at other times[62] and the conserved portion of the genome becomes increasingly marked by histone acetylation, thought to indicate regulatory element use, as the phylotypic stage approaches[63]. Our data are consistent with, and further support, the view that conserved elements control phylotypic aspects of development. That many CNEs act as hindbrain enhancers in functional assays suggests that they coordinate the development of the hindbrain, a shared-derived vertebrate character. We propose the segment-specific hindbrain enhancers identified in this study lie directly downstream of the *hox* code and may mediate regulatory cascades that subsequently identify rhombomeres.

It should be noted, however, that conserved elements are subject to some degree of lineage-specific sequence [8] and functional [22,64] divergence. Whether such changes are adaptations or not is unclear. Further studies on elucidating the expression of elements orthologous to those identified here in their host species, together with ancestral reconstruction and mutagenesis to track the effects of lineage-specific mutations, will allow the diversification of these enhancers to be investigated. The functional implications of such alterations may then be assessed.

### Vertebrate CNEs couple the *hox* code to downstream genes

Patterns of *hox* expression have often been thought to provide “positional information” to embryos rather than encoding any particular structure[65], presumably because HOX proteins target, and subsequently activate, different genes in different metazoan groups. Thus, the coupling of particular HOX proteins to novel targets by the acquisition of new *cis*-regulatory elements is likely to play a role in the assignment of distinct identities to body segments, generating novel morphology over the course of evolution. The vertebrate phylotypic stage exhibits the earliest, and simplest, pattern of *hox* gene expression along the antero-posterior axis of the embryo, and by comparison with amphioxus it appears that this pattern is largely ancestral[66,67,68].

Regulatory elements from both amphioxus and ciona *hox* cluster can drive segment-restricted hindbrain expression in vertebrate embryos[69,70]. However, in these assays the elements are being visualised in the context of the vertebrate hindbrain GRN, where there are distinct boundaries between *hox* expression patterns that foreshadow and delineate morphological boundaries. These elements share no sequence identity with elements from the vertebrate clusters but could be considered analogous, as they appear to be responsible for setting up initial, ancestral patterns of *hox* expression. Despite this conservation of *hox* expression patterns amongst chordates, the region expressing *hox* paralogs 1–4 in protochordates is not segmented and lacks overlapping patterns of *egr2* and *mafb* expression[17,18,19].

In contrast, vertebrate *hox* paralogs 1–4 and their cofactors are known to play a role in both the initial segmentation and subsequent identification of rhombomeres [13,14,15,16]. The evolution of the hindbrain from the ancestral, unsegmented region has therefore involved the coupling of the *hox* code to both the segmentation machinery and to genes involved in determining rhombomere identities and the subsequent patterning of rhombomeric derivatives, such as cranial nerves and neural-crest derived craniofacial structures. We propose that the CNEs identified in this study are the extant remnants of ancient regulatory elements that coupled the collinear *hox* code to segment-specific sets of target genes during early vertebrate evolution.

Indeed, the co-option of *egr2* to the hindbrain GRN may have arisen in this way, because a conserved enhancer responsible for the initiation of *egr2* is activated by a PBX-HOX-MEIS heterotrimer [42]. Further studies tracking the gain or loss of these motifs could test the validity of this mechanism for the gain of novel hindbrain enhancers. To this end, distantly related orthologous sequences to the hindbrain enhancers studied here, such as those from the sea lamprey, may shed light on the correlation between the presence of PBX-HOX and MEIS/PREP motifs and segmentally restricted, HOX-dependent enhancer function.

We propose that the enhancers studied here mediate the establishment of a GRN controlling both the segmentation and identification of rhombomeres. This model suggests a mechanistic link between a genomic signature (thousands of CNEs) and a lineage-specific character (hb segmentation and subsequent elaboration), providing insight in to how the early vertebrate hindbrain evolved from the unsegmented region of the CNS in the invertebrate ancestor.

### The hindbrain syntax predicts a genome-wide set of putative human hindbrain enhancers

We have identified >3000 conserved occurrences of the hindbrain syntax (hb_40) in the human genome. We posit that these regions are likely to be located within functional, HOX-dependant hindbrain regulatory elements. Much like deeply conserved sequences, these sites associate closely with transcription factors involved in development, indicating that many may be regulatory elements controlling their expression to choreograph embryogenesis. Furthermore, we find that there is significant overlap between these sites and functional human enhancers from the VISTA enhancer browser [25], especially enhancers annotated for hindbrain expression. Therefore, many more of these sites may correspond to the locations of human hindbrain enhancers, although this remains to be tested.

Future work could head in several directions. Functional studies on these putative hindbrain enhancers, such as enhancer assays or knockout using CRISPR or TALENS, will assess their roles during development. The predictive power of the hb_40 syntax needs to be rigorously tested in different vertebrates. Finally, the methods used herein can be applied to elucidate similar enhancer grammars for other tissues or organs. Using a combination of these approaches, comprehensive catalogues of putative tissue-specific enhancers can be built, allowing for targeted functional studies to assess their roles during development and to aid us in understanding the function of the non-coding portion of the human genome.

## Materials and Methods

All animal work in this study was performed under Home Office UK Project License PPL 80/2459

### *In vivo* transient enhancer assay

The general approach for enhancer assays has been described previously [71]. Zebrafish orthologues of CNEs were amplified from zebrafish genomic DNA by PCR (primer sequences available upon request). PCR products were cloned in to the pCR8/GW/TOPO entry vector (Invitrogen) according to the manufacturer's guidelines, then transferred to the pGW_*cfos*EGFP expression vector (sequence available upon request) by Gateway LR recombination (Invitrogen) according to the manufacturer's guidelines. Plasmid isolates were obtained using the QIAprep spin miniprep kit (Qiagen). Inserts were confirmed by Sanger sequencing.

Tol2 transposase mRNA was transcribed in-vitro from a linearised vector containing the Tol2 transposase gene using the mMESSAGE mMACHINE SP6 kit (Invitrogen). pGW_cfosEGFP expression constructs were co-injected alongside Tol2 transposase mRNA and phenol red (tracer) in to wild-type or KROX20:RFP zebrafish. F0 embryos were incubated at 28°C and screened for EGFP expression at 24 h, 48 h and/or 72 h using fluorescence microscopy. Sequences were considered to be hindbrain enhancers if there were GFP positive cells in r2 to r6, in at least 20% of injected embryos, at any of the three time points. At least 30 embryos were assayed in each case. For the comparison of wt and mutant constructs, three sets of replicates were performed for each construct (wt, pbx-hox- and meis/prep-, mutant sequences available upon request). Each replicate comprised at least 15 injected embryos and each set of three replicates totalled at least 70 embryos.

### Motif discovery

We performed *de novo* motif discovery on both the hb+ and hb- sets using MEME[27], allowing any number of motif repetitions per sequence to contribute to the PWM and a PWM width range of 6–10 bp. In all cases, shuffled sequence controls were also performed. The derived PWMs were compared to known TF binding preferences stored in JASPAR[30], TRANSFAC[28] and Uniprobe[29] using TOMTOM[31] and STAMP[32].

### Searching for motif co-occurrences in CNEs

We submitted the PBX-HOX and MEIS/PREP PWMs derived from the hb+ set to FIMO [36] and MCAST [72] and used these to search the hb+ and hb- sets. Motifs were considered to co-occur if they occurred within 100bp in the same sequence.

### Phylogenetic footprinting

Multiple alignments of CNEs, derived from BLAST hits, were retrieved from the CONDOR database and realigned using ClustalW2 [44]. As CONDOR uses some older genome assemblies to generate alignments, in some instances zebrafish, chicken and/or frog CNEs were identified by BLAST, using the fugu (for zebrafish) or human (for chicken and frog) CNE sequence as a query. Between 16 and 25 vertebrate orthologues were aligned in each case.

### Identification of candidate hindbrain enhancers

4 orthologous sets of CNEs (human, mouse, rat/dog and fugu) were downloaded from CONDOR, and submitted to FIMO [36] together with PWMs describing PBX-HOX and MEIS/PREP binding preferences, roughly conforming to TGATDDATKD and CTGYCA respectively. Elements were considered candidates for functional assay if they a) contained significant matches (p = <0.001) to both motifs; b) these were within 100 bp of one another; c) They were positionally conserved in all aligned species. Positional conservation of the two motifs in other vertebrates (including zebrafish) was confirmed by phylogenetic footprinting as described above.

### Site directed mutagenesis

Mutations were introduced to constructs using the Quickchange approach (Agilent) according to the manufacturer’s guidelines. Mutant constructs were then assayed as described above, and compared to wt constructs as a control to assess the effects of the mutations on enhancer activity.

### Genome-wide prediction of putative hindbrain enhancers

#### Motif matches

We searched for matches of MEIS/PREP ("CTGTCA") and PBX-HOX ("TGATNNATKR") motifs in the standard repeat masked version of hg19 assembly of the human genome (chromosomes 1–22,X,Y). Motif matches in the masked sequence were not taken into account. A total of 937137 MEIS/PREP matches and 281306 PBX-HOX matches were found. Then we filtered out motif matches that are not evolutionarily conserved. We used a set of conserved elements computed using GERP++ software[45]. There are 1044996 conserved regions (GERP regions) in the set ranging in size from 4 to 2000 bases and covering a total of 210.7Mb in the human genome. The number of MEIS/PREP and PBX-HOX conserved sites is 156677 and 48608 respectively.

Next we produced a list of pairs of MEIS/PREP and PBX-HOX sites separated by 100 or less bases (but not overlapping). There are 31081 such pairs across the genome, 6360 of which fall into GERP regions (S6 Table). The hb_40 elements are the conserved MEIS/PREP-PBX-HOX sites with up to 40 bases between the MEIS/PREP and PBX-HOX motifs. There are 3122 hb_40 elements. The non-CNE hb_40 elements are a subset of the hb_40 set excluding those 93 that are located fully inside a CNE.

#### Random simulations

To demonstrate that the gap sizes between the MEIS/PREP and PBX-HOX sites tend to be smaller than one would expect by chance, we randomly choose 156677 6mer and 48608 10mer locations across the GERP regions and then calculated the number of "hb_40 elements" in the simulated set. We performed 10,000 simulations. The number of "hb_40 elements" ranged from 2408 to 2837 with a mean at 2604 and standard deviation 50.5 (S2 Fig). The real value of 3122 is therefore 10.25 standard deviations above the mean; this is equivalent to a p-value of 6x10^-25^.

#### Calculating enrichment of hb_40 elements in hindbrain enhancer sets

A list of 1729 human enhancers (Vista enhancers) was obtained from the VISTA Enhancer Browser [25]. There are 237 enhancers annotated as hindbrain positive in the list.

We use a hypergeometric test to show that among all the GERP regions the hindbrain enhancers are enriched in hb_40 elements. Out of the total of 1044996 GERP regions 489 overlap the hindbrain enhancers and 2998 (2923) have at least one hb_40 (non-CNE hb_40) element in it. By chance one would expect to get on average only 1.4 GERP regions both having the hindbrain function and containing an hb_40 site, but there are 21 (14) GERP regions of this kind giving enrichment p-value 4.0×10^–18^ (2.2×10^–10^).

The hindbrain enhancers are also enriched in hb_40 elements in comparison to the full set of Vista enhancers. Out of 1729 Vista enhancers 58 (37) contain at least one hb_40 (non-CNE hb_40) element, in the subset of 237 hindbrain enhancers there are 21 (14) containing an hb_40 (non-CNE hb_40) element, though one can expect only 8 (5) by chance. The hypergeometric test p-value is 9.6×10^–6^ (1.8×10^–4^).

We downloaded a list of 42144 predicted enhancers from Burzynski *et al*. [23] covering 10.5 Mb. We used liftOver tool to convert the coordinates from hg18 to hg19 (losing 7 regions) and then calculated the number of hb_40 elements per Mb.

#### Analysis of enriched GO terms for genes near hb_40 elements

We took each non-CNE hb_40 element, expanded it by 100Kb on both sides, merged any overlapping regions, and using Biomart pulled the Uniprot IDs of genes residing in these regions. Then we ran a GO term enrichment analysis using GoStat for the pulled set of genes against the whole set of human genes in the Uniprot database [73].

#### Analysis of distance of hb_40 elements from CNEs

We computed the distance from the middle point of each hb_40 element to the middle point of the closest CNE and compared the distribution of these distances to the background distribution of distances from each GERP region to the closest CNE (S3 Fig). The former distribution is heavily skewed towards smaller distances compared to the background one.

The proportion of distances of 10Kb or less is 2.5% for the background distribution but it is significantly larger for the hb_40 element distances—8.6%, and even for the non-CNE hb_40 element distances—5.9%. The first decile of the hb_40 (non-CNE hb_40) distances is 21.5Kb (69.5Kb) compared to 849Kb for the background distribution. The 10% shortest distances from hb_40 (non-CNE hb_40) to CNE are significantly smaller than the 10% shortest distances from the background distribution, the p-values from a Mann-Whitney test are 4×10^-129^(1×10^–62^).

#### Analysis of distribution of hb_40 elements with respect to gene regions

Annotations from the human genome were downloaded from biomart and their overlap was compared to the hb_40 elements. There are a total of 3122 hb_40 elements. 1227 hb_40 elements overlap 875 gene regions (defined as the region from "Gene start" to "Gene end" in biomart). The number of hb_40 that overlap coding sequence is 156, in 151 different gene regions. 1 hb_40 element is found within 100bp of the most 5’ TSS of a gene and only 10 are found within 1kb. hb_40 elements also occasionally overlap each other; this is caused by the occurrence of multiple PBX-HOX and MEIS motifs with a distance of no more than 40bp between each. There are 76 such overlaps.

## Supporting Information

S1 Fig Enhancer activity driven by *meis2a* CNEs containing PBX-HOX or MEIS/PREP motifs. Images show the hindbrain and midbrain region of 48 hpf zebrafish embryos. GFP shows enhancer activity driven by injected constructs. RFP shows hindbrain rhombomeres 3 and 5. cg: cranial ganglia; hb: hindbrain; mb: midbrain.
(TIF)

S2 Fig Distribution of gaps between PBX-HOX motifs and MEIS/PREP motifs. A) Graph shows the distances between PBX-HOX and MEIS/PREP motifs in human GERP regions (blue) against the distribution of distances in an average of 10,000 sets of simulated data (black). B) Graph shows the distribution of the numbers of hb_40 elements in 10,000 simulated sets (grey) and the number in the human genome (red).
(PNG)

S3 Fig Distribution of hb_40 elements with respect to CNEs. Graph shows the distribution of distances from non-CNE hb_40 elements (in blue) and all GERP regions (in black) to the closest CNE.
(PNG)

S1 File Phylogenetic footprinting of hb+ enhancers. Clustalw2 alignments of all the hb+ elements, showing the conservation and distribution of PBX-HOX and MEIS/PREP motifs.
(PPTX)

S2 File Phylogenetic footprinting of hindbrain enhancer candidates. Clustalw2 alignments of all the hb+ candidates as determined by FIMO, showing the conservation and distribution of PBX-HOX and MEIS/PREP motifs.
(PPTX)

S1 Table Tissue specificity data for 29 CNEs containing conserved PBX-HOX motifs. Table shows the total number of injected embryos, the total number of GFP positive embryos and the number of embryos positive for each tissue. 7/29 elements were considered to be hindbrain enhancers.
(XLSX)

S2 Table FIMO output file. Table of CNEs containing significant hits to both PBX-HOX and MEIS/PREP motifs in human, mouse, rat and fugu CNEs, and coordinates of each candidate CNE.
(XLSX)

S3 Table Tissue specificity data for 75 CNEs containing conserved PBX-HOX and MEIS/PREP motifs. Table shows the total number of injected embryos, the total number of GFP positive embryos and the number of embryos positive for each tissue. 67/75 elements were considered to be hindbrain enhancers.
(XLSX)

S4 Table Locations of all CNEs assayed in this study. The coordinates of the elements assayed in this study in the zebrafish genome and the corresponding regions in the human genome are shown.
(XLSX)

S5 Table Tissue specificity data for 8 *meis2a* CNEs containing PBX-HOX or MEIS/PREP motifs. Table shows the total number of injected embryos, the total number of GFP positive embryos and the number of embryos positive for each tissue. 2/8 elements were considered to be hindbrain enhancers and 0/8 were considered to be hindbrain specific.
(XLSX)

S6 Table PBX-HOX and MEIS/PREP motifs located within 100bp in the human genome. Table shows the locations of all PBX-Hox and MEIS/PREP motifs located within 100bp (hb_100 elements). The gap between sites and whether the motifs fall within a GERP region are displayed.
(XLSX)

S7 Table GO terms enriched in genes associated with hb_40 elements. Table showing GO accessions, descriptions and p-values associated with each term according to GOSTAT.
(XLSX)

S1 Text MEME test set. Sequences of 38 hindbrain enhancers used for MEME analysis.
(TXT)

S2 Text MEME control set. Sequences of 160 control elements not active in hindbrain.
(TXT)

S3 Text PWMs derived from the hb+ set. Frequency matrices of two motifs enriched in the hb+ set (PBX-HOX and MEIS/PREP).
(TXT)
