# Supplementary figures and images for: A Simple Predictive Enhancer Syntax for Hindbrain Patterning Is Conserved in Vertebrate Genomes
Source: PLoS One. 2015 Jul 1;10(7):e0130413. doi: 10.1371/journal.pone.0130413 (PMC4489388; doi:10.1371/journal.pone.0130413)

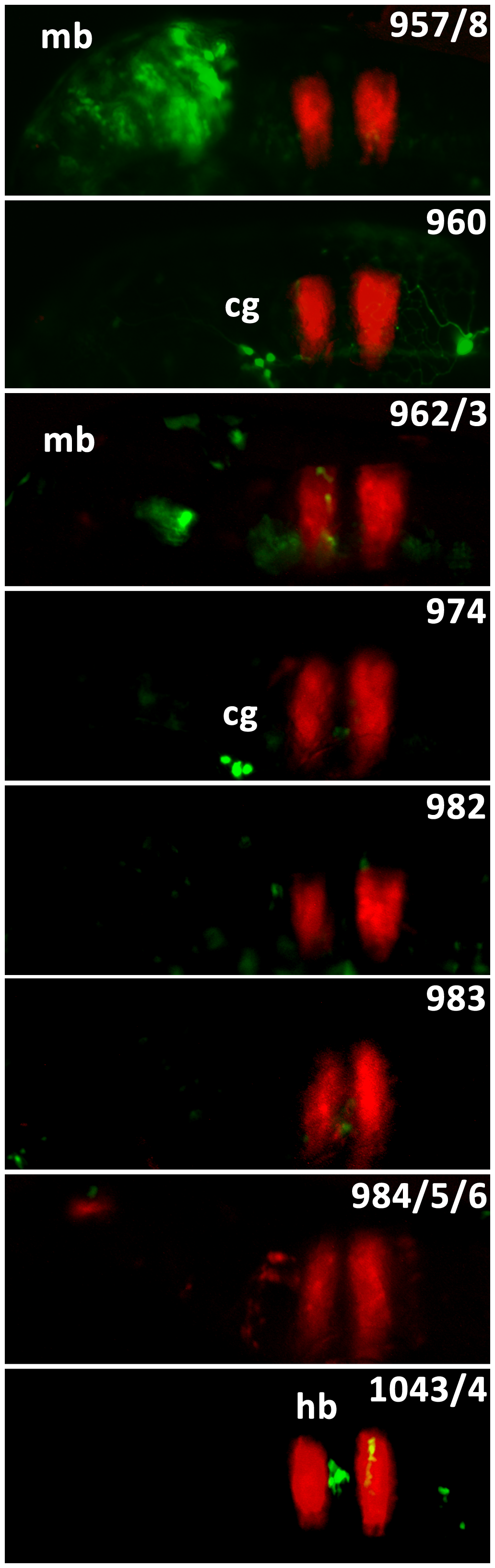

Supplement: S1 Fig — Images show the hindbrain and midbrain region of 48 hpf zebrafish embryos. GFP shows enhancer activity driven by injected constructs. RFP shows hindbrain rhombomeres 3 and 5. cg: cranial ganglia; hb: hindbrain; mb: midbrain. (TIF) [file pone.0130413.s001.tif]

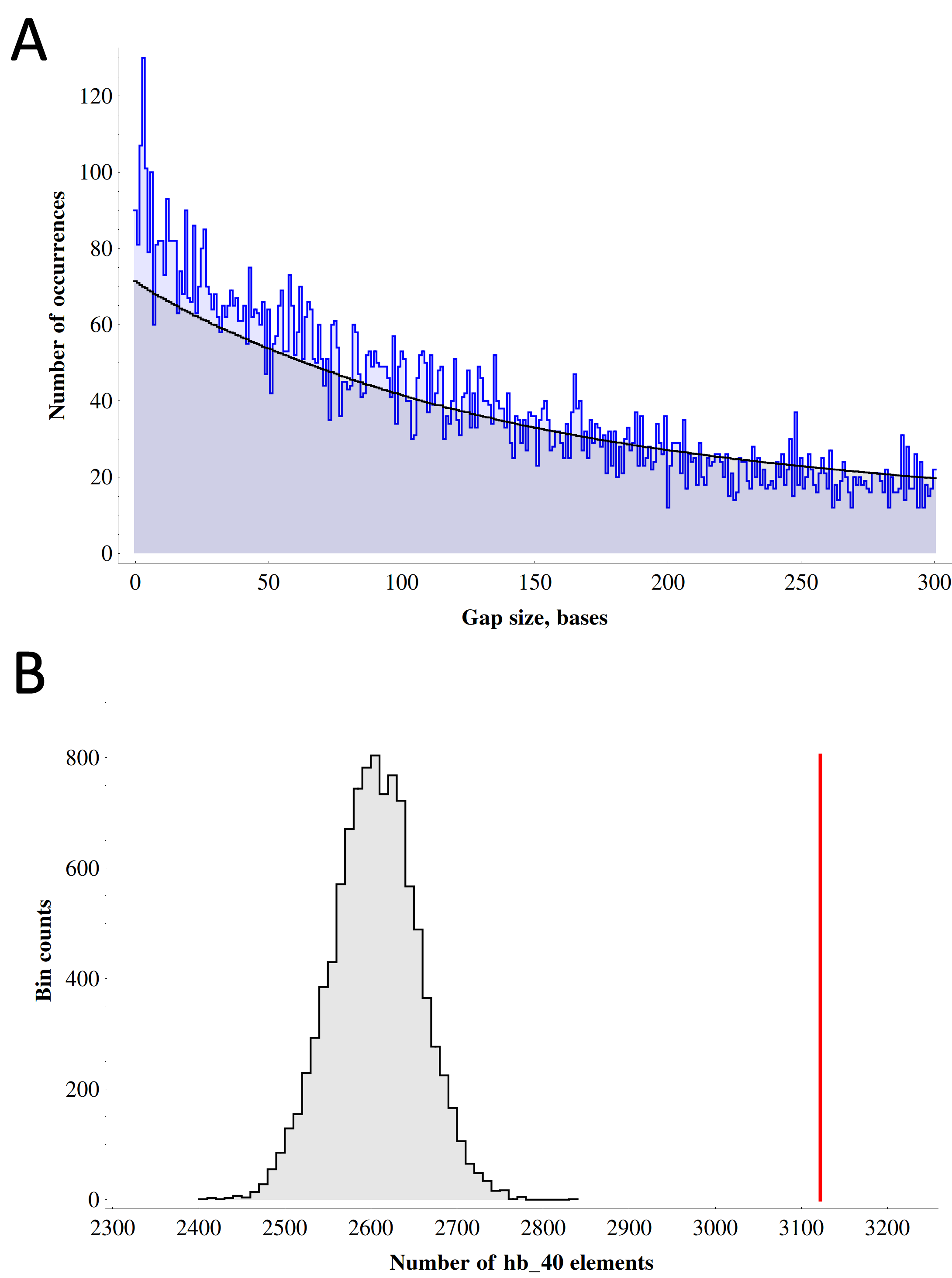

Supplement: S2 Fig — A) Graph shows the distances between PBX-HOX and MEIS/PREP motifs in human GERP regions (blue) against the distribution of distances in an average of 10,000 sets of simulated data (black). B) Graph shows the distribution of the numbers of hb_40 elements in 10,000 simulated sets (grey) and the number in the human genome (red). (PNG) [file pone.0130413.s002.png]

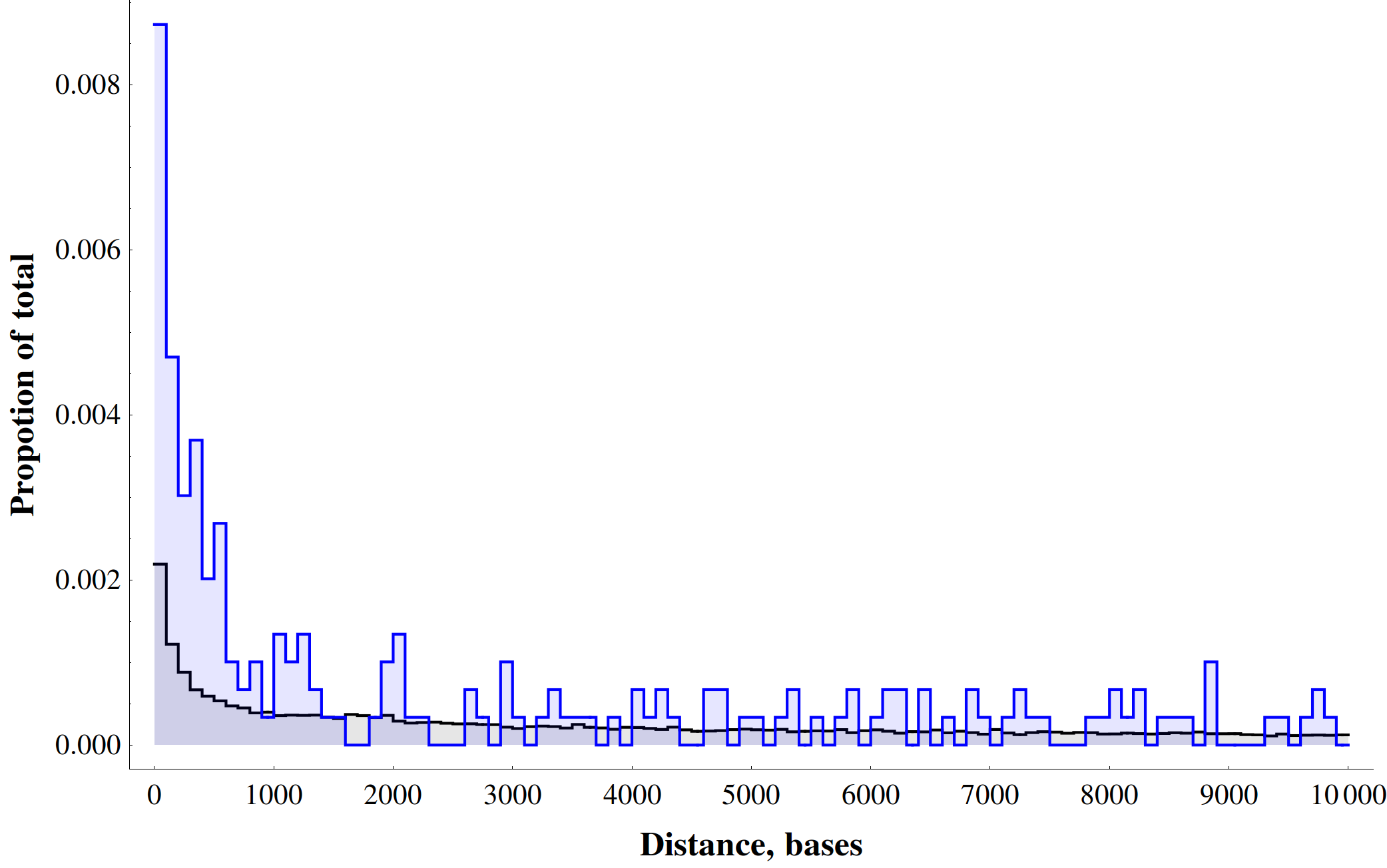

Supplement: S3 Fig — Graph shows the distribution of distances from non-CNE hb_40 elements (in blue) and all GERP regions (in black) to the closest CNE. (PNG) [file pone.0130413.s003.png]
