# Supplementary material for: A Simple Predictive Enhancer Syntax for Hindbrain Patterning Is Conserved in Vertebrate Genomes
Source: PLoS One. 2015 Jul 1;10(7):e0130413. doi: 10.1371/journal.pone.0130413 (PMC4489388; doi:10.1371/journal.pone.0130413)

## Slide 1
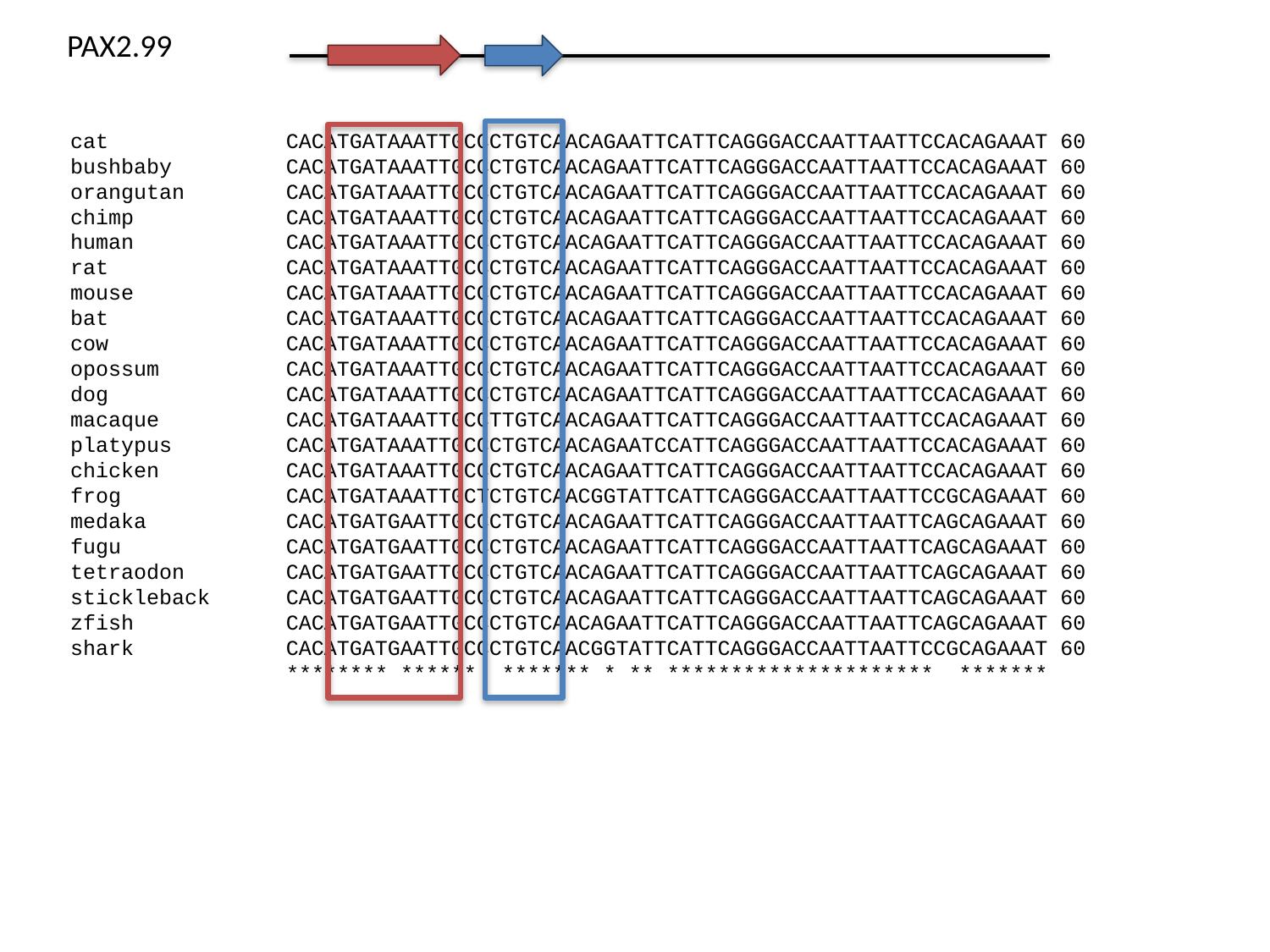

PAX2.99
cat CACATGATAAATTGCCCTGTCAACAGAATTCATTCAGGGACCAATTAATTCCACAGAAAT 60
bushbaby CACATGATAAATTGCCCTGTCAACAGAATTCATTCAGGGACCAATTAATTCCACAGAAAT 60
orangutan CACATGATAAATTGCCCTGTCAACAGAATTCATTCAGGGACCAATTAATTCCACAGAAAT 60
chimp CACATGATAAATTGCCCTGTCAACAGAATTCATTCAGGGACCAATTAATTCCACAGAAAT 60
human CACATGATAAATTGCCCTGTCAACAGAATTCATTCAGGGACCAATTAATTCCACAGAAAT 60
rat CACATGATAAATTGCCCTGTCAACAGAATTCATTCAGGGACCAATTAATTCCACAGAAAT 60
mouse CACATGATAAATTGCCCTGTCAACAGAATTCATTCAGGGACCAATTAATTCCACAGAAAT 60
bat CACATGATAAATTGCCCTGTCAACAGAATTCATTCAGGGACCAATTAATTCCACAGAAAT 60
cow CACATGATAAATTGCCCTGTCAACAGAATTCATTCAGGGACCAATTAATTCCACAGAAAT 60
opossum CACATGATAAATTGCCCTGTCAACAGAATTCATTCAGGGACCAATTAATTCCACAGAAAT 60
dog CACATGATAAATTGCCCTGTCAACAGAATTCATTCAGGGACCAATTAATTCCACAGAAAT 60
macaque CACATGATAAATTGCCTTGTCAACAGAATTCATTCAGGGACCAATTAATTCCACAGAAAT 60
platypus CACATGATAAATTGCCCTGTCAACAGAATCCATTCAGGGACCAATTAATTCCACAGAAAT 60
chicken CACATGATAAATTGCCCTGTCAACAGAATTCATTCAGGGACCAATTAATTCCACAGAAAT 60
frog CACATGATAAATTGCTCTGTCAACGGTATTCATTCAGGGACCAATTAATTCCGCAGAAAT 60
medaka CACATGATGAATTGCCCTGTCAACAGAATTCATTCAGGGACCAATTAATTCAGCAGAAAT 60
fugu CACATGATGAATTGCCCTGTCAACAGAATTCATTCAGGGACCAATTAATTCAGCAGAAAT 60
tetraodon CACATGATGAATTGCCCTGTCAACAGAATTCATTCAGGGACCAATTAATTCAGCAGAAAT 60
stickleback CACATGATGAATTGCCCTGTCAACAGAATTCATTCAGGGACCAATTAATTCAGCAGAAAT 60
zfish CACATGATGAATTGCCCTGTCAACAGAATTCATTCAGGGACCAATTAATTCAGCAGAAAT 60
shark CACATGATGAATTGCCCTGTCAACGGTATTCATTCAGGGACCAATTAATTCCGCAGAAAT 60
 ******** ****** ******* * ** ********************* *******

## Slide 2
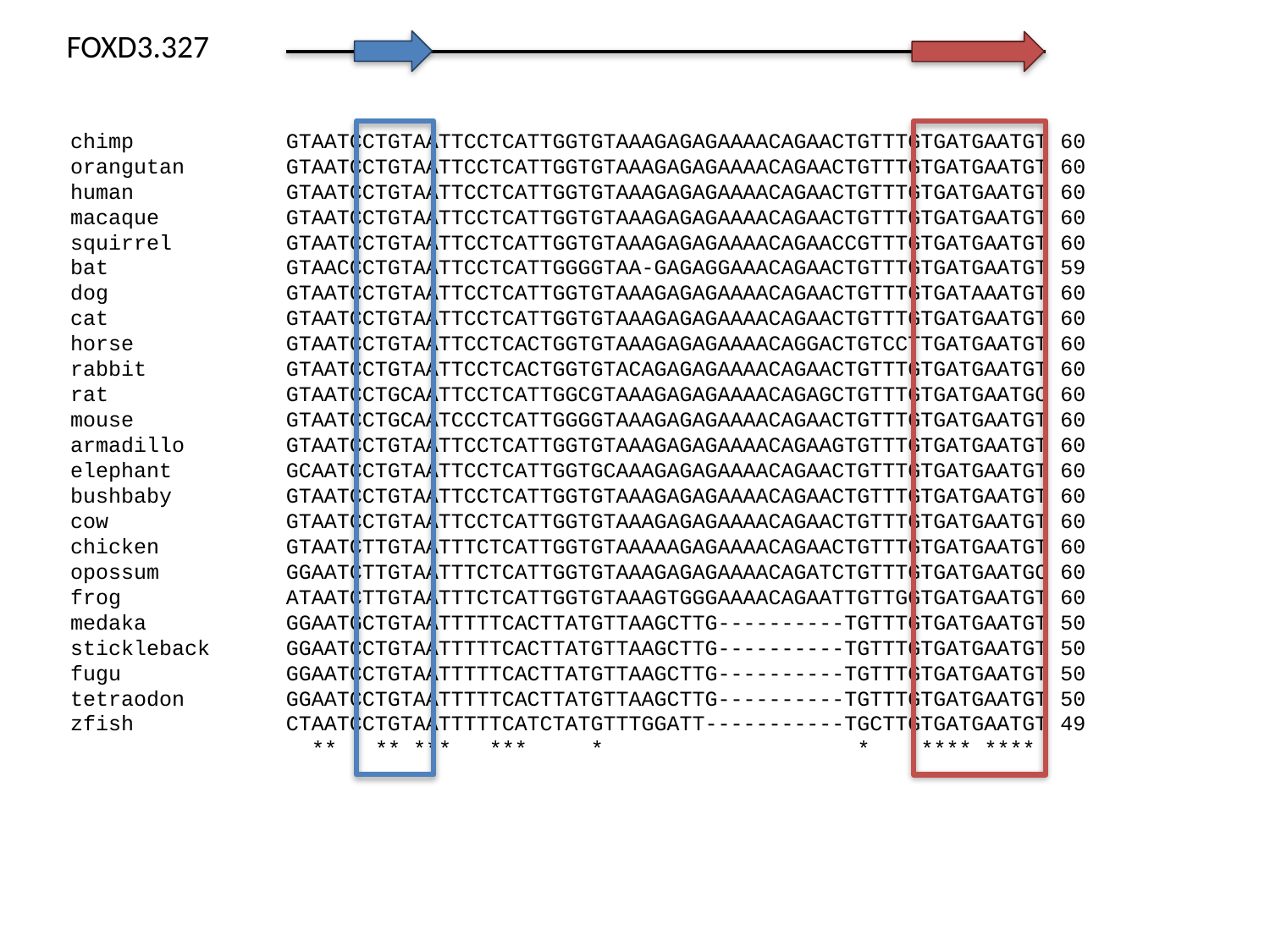

FOXD3.327
chimp GTAATCCTGTAATTCCTCATTGGTGTAAAGAGAGAAAACAGAACTGTTTGTGATGAATGT 60
orangutan GTAATCCTGTAATTCCTCATTGGTGTAAAGAGAGAAAACAGAACTGTTTGTGATGAATGT 60
human GTAATCCTGTAATTCCTCATTGGTGTAAAGAGAGAAAACAGAACTGTTTGTGATGAATGT 60
macaque GTAATCCTGTAATTCCTCATTGGTGTAAAGAGAGAAAACAGAACTGTTTGTGATGAATGT 60
squirrel GTAATCCTGTAATTCCTCATTGGTGTAAAGAGAGAAAACAGAACCGTTTGTGATGAATGT 60
bat GTAACCCTGTAATTCCTCATTGGGGTAA-GAGAGGAAACAGAACTGTTTGTGATGAATGT 59
dog GTAATCCTGTAATTCCTCATTGGTGTAAAGAGAGAAAACAGAACTGTTTGTGATAAATGT 60
cat GTAATCCTGTAATTCCTCATTGGTGTAAAGAGAGAAAACAGAACTGTTTGTGATGAATGT 60
horse GTAATCCTGTAATTCCTCACTGGTGTAAAGAGAGAAAACAGGACTGTCCTTGATGAATGT 60
rabbit GTAATCCTGTAATTCCTCACTGGTGTACAGAGAGAAAACAGAACTGTTTGTGATGAATGT 60
rat GTAATCCTGCAATTCCTCATTGGCGTAAAGAGAGAAAACAGAGCTGTTTGTGATGAATGC 60
mouse GTAATCCTGCAATCCCTCATTGGGGTAAAGAGAGAAAACAGAACTGTTTGTGATGAATGT 60
armadillo GTAATCCTGTAATTCCTCATTGGTGTAAAGAGAGAAAACAGAAGTGTTTGTGATGAATGT 60
elephant GCAATCCTGTAATTCCTCATTGGTGCAAAGAGAGAAAACAGAACTGTTTGTGATGAATGT 60
bushbaby GTAATCCTGTAATTCCTCATTGGTGTAAAGAGAGAAAACAGAACTGTTTGTGATGAATGT 60
cow GTAATCCTGTAATTCCTCATTGGTGTAAAGAGAGAAAACAGAACTGTTTGTGATGAATGT 60
chicken GTAATCTTGTAATTTCTCATTGGTGTAAAAAGAGAAAACAGAACTGTTTGTGATGAATGT 60
opossum GGAATCTTGTAATTTCTCATTGGTGTAAAGAGAGAAAACAGATCTGTTTGTGATGAATGC 60
frog ATAATCTTGTAATTTCTCATTGGTGTAAAGTGGGAAAACAGAATTGTTGGTGATGAATGT 60
medaka GGAATGCTGTAATTTTTCACTTATGTTAAGCTTG----------TGTTTGTGATGAATGT 50
stickleback GGAATCCTGTAATTTTTCACTTATGTTAAGCTTG----------TGTTTGTGATGAATGT 50
fugu GGAATCCTGTAATTTTTCACTTATGTTAAGCTTG----------TGTTTGTGATGAATGT 50
tetraodon GGAATCCTGTAATTTTTCACTTATGTTAAGCTTG----------TGTTTGTGATGAATGT 50
zfish CTAATCCTGTAATTTTTCATCTATGTTTGGATT-----------TGCTTGTGATGAATGT 49
 ** ** *** *** * * **** ****

## Slide 3
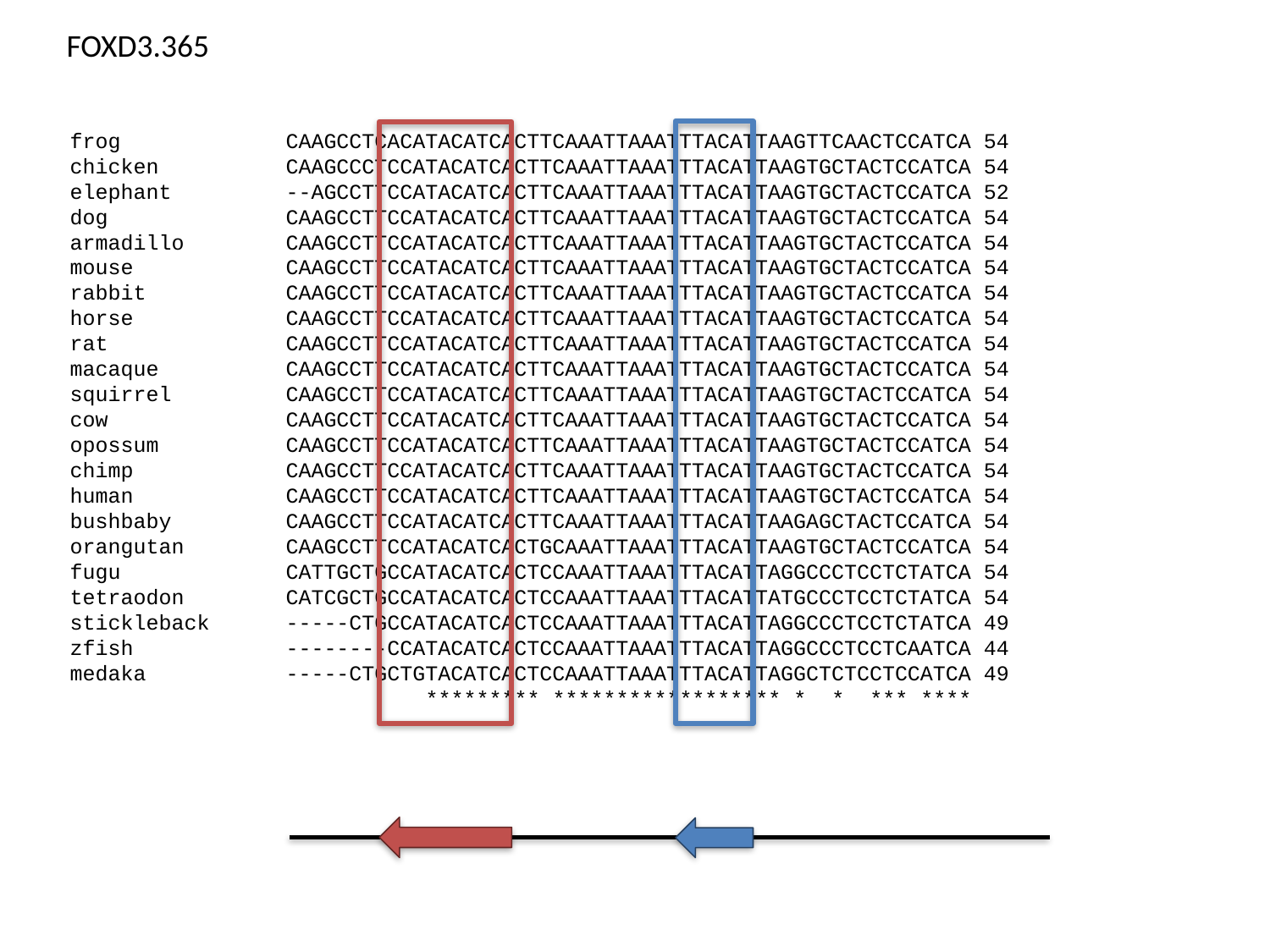

FOXD3.365
frog CAAGCCTCACATACATCACTTCAAATTAAATTTACATTAAGTTCAACTCCATCA 54
chicken CAAGCCCTCCATACATCACTTCAAATTAAATTTACATTAAGTGCTACTCCATCA 54
elephant --AGCCTTCCATACATCACTTCAAATTAAATTTACATTAAGTGCTACTCCATCA 52
dog CAAGCCTTCCATACATCACTTCAAATTAAATTTACATTAAGTGCTACTCCATCA 54
armadillo CAAGCCTTCCATACATCACTTCAAATTAAATTTACATTAAGTGCTACTCCATCA 54
mouse CAAGCCTTCCATACATCACTTCAAATTAAATTTACATTAAGTGCTACTCCATCA 54
rabbit CAAGCCTTCCATACATCACTTCAAATTAAATTTACATTAAGTGCTACTCCATCA 54
horse CAAGCCTTCCATACATCACTTCAAATTAAATTTACATTAAGTGCTACTCCATCA 54
rat CAAGCCTTCCATACATCACTTCAAATTAAATTTACATTAAGTGCTACTCCATCA 54
macaque CAAGCCTTCCATACATCACTTCAAATTAAATTTACATTAAGTGCTACTCCATCA 54
squirrel CAAGCCTTCCATACATCACTTCAAATTAAATTTACATTAAGTGCTACTCCATCA 54
cow CAAGCCTTCCATACATCACTTCAAATTAAATTTACATTAAGTGCTACTCCATCA 54
opossum CAAGCCTTCCATACATCACTTCAAATTAAATTTACATTAAGTGCTACTCCATCA 54
chimp CAAGCCTTCCATACATCACTTCAAATTAAATTTACATTAAGTGCTACTCCATCA 54
human CAAGCCTTCCATACATCACTTCAAATTAAATTTACATTAAGTGCTACTCCATCA 54
bushbaby CAAGCCTTCCATACATCACTTCAAATTAAATTTACATTAAGAGCTACTCCATCA 54
orangutan CAAGCCTTCCATACATCACTGCAAATTAAATTTACATTAAGTGCTACTCCATCA 54
fugu CATTGCTGCCATACATCACTCCAAATTAAATTTACATTAGGCCCTCCTCTATCA 54
tetraodon CATCGCTGCCATACATCACTCCAAATTAAATTTACATTATGCCCTCCTCTATCA 54
stickleback -----CTGCCATACATCACTCCAAATTAAATTTACATTAGGCCCTCCTCTATCA 49
zfish --------CCATACATCACTCCAAATTAAATTTACATTAGGCCCTCCTCAATCA 44
medaka -----CTGCTGTACATCACTCCAAATTAAATTTACATTAGGCTCTCCTCCATCA 49
 ********* ****************** * * *** ****

## Slide 4
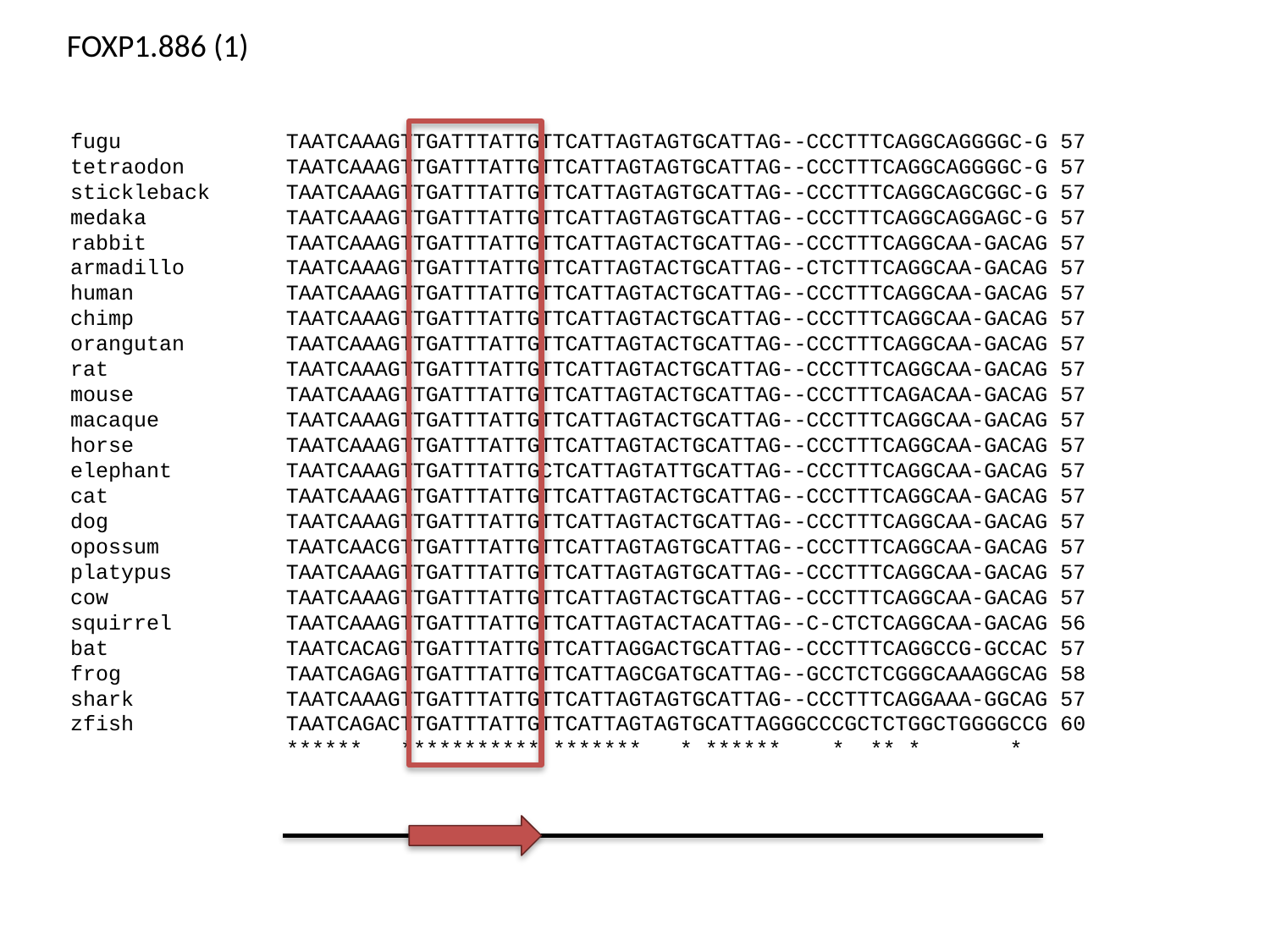

FOXP1.886 (1)
fugu TAATCAAAGTTGATTTATTGTTCATTAGTAGTGCATTAG--CCCTTTCAGGCAGGGGC-G 57
tetraodon TAATCAAAGTTGATTTATTGTTCATTAGTAGTGCATTAG--CCCTTTCAGGCAGGGGC-G 57
stickleback TAATCAAAGTTGATTTATTGTTCATTAGTAGTGCATTAG--CCCTTTCAGGCAGCGGC-G 57
medaka TAATCAAAGTTGATTTATTGTTCATTAGTAGTGCATTAG--CCCTTTCAGGCAGGAGC-G 57
rabbit TAATCAAAGTTGATTTATTGTTCATTAGTACTGCATTAG--CCCTTTCAGGCAA-GACAG 57
armadillo TAATCAAAGTTGATTTATTGTTCATTAGTACTGCATTAG--CTCTTTCAGGCAA-GACAG 57
human TAATCAAAGTTGATTTATTGTTCATTAGTACTGCATTAG--CCCTTTCAGGCAA-GACAG 57
chimp TAATCAAAGTTGATTTATTGTTCATTAGTACTGCATTAG--CCCTTTCAGGCAA-GACAG 57
orangutan TAATCAAAGTTGATTTATTGTTCATTAGTACTGCATTAG--CCCTTTCAGGCAA-GACAG 57
rat TAATCAAAGTTGATTTATTGTTCATTAGTACTGCATTAG--CCCTTTCAGGCAA-GACAG 57
mouse TAATCAAAGTTGATTTATTGTTCATTAGTACTGCATTAG--CCCTTTCAGACAA-GACAG 57
macaque TAATCAAAGTTGATTTATTGTTCATTAGTACTGCATTAG--CCCTTTCAGGCAA-GACAG 57
horse TAATCAAAGTTGATTTATTGTTCATTAGTACTGCATTAG--CCCTTTCAGGCAA-GACAG 57
elephant TAATCAAAGTTGATTTATTGCTCATTAGTATTGCATTAG--CCCTTTCAGGCAA-GACAG 57
cat TAATCAAAGTTGATTTATTGTTCATTAGTACTGCATTAG--CCCTTTCAGGCAA-GACAG 57
dog TAATCAAAGTTGATTTATTGTTCATTAGTACTGCATTAG--CCCTTTCAGGCAA-GACAG 57
opossum TAATCAACGTTGATTTATTGTTCATTAGTAGTGCATTAG--CCCTTTCAGGCAA-GACAG 57
platypus TAATCAAAGTTGATTTATTGTTCATTAGTAGTGCATTAG--CCCTTTCAGGCAA-GACAG 57
cow TAATCAAAGTTGATTTATTGTTCATTAGTACTGCATTAG--CCCTTTCAGGCAA-GACAG 57
squirrel TAATCAAAGTTGATTTATTGTTCATTAGTACTACATTAG--C-CTCTCAGGCAA-GACAG 56
bat TAATCACAGTTGATTTATTGTTCATTAGGACTGCATTAG--CCCTTTCAGGCCG-GCCAC 57
frog TAATCAGAGTTGATTTATTGTTCATTAGCGATGCATTAG--GCCTCTCGGGCAAAGGCAG 58
shark TAATCAAAGTTGATTTATTGTTCATTAGTAGTGCATTAG--CCCTTTCAGGAAA-GGCAG 57
zfish TAATCAGACTTGATTTATTGTTCATTAGTAGTGCATTAGGGCCCGCTCTGGCTGGGGCCG 60
 ****** *********** ******* * ****** * ** * *

## Slide 5
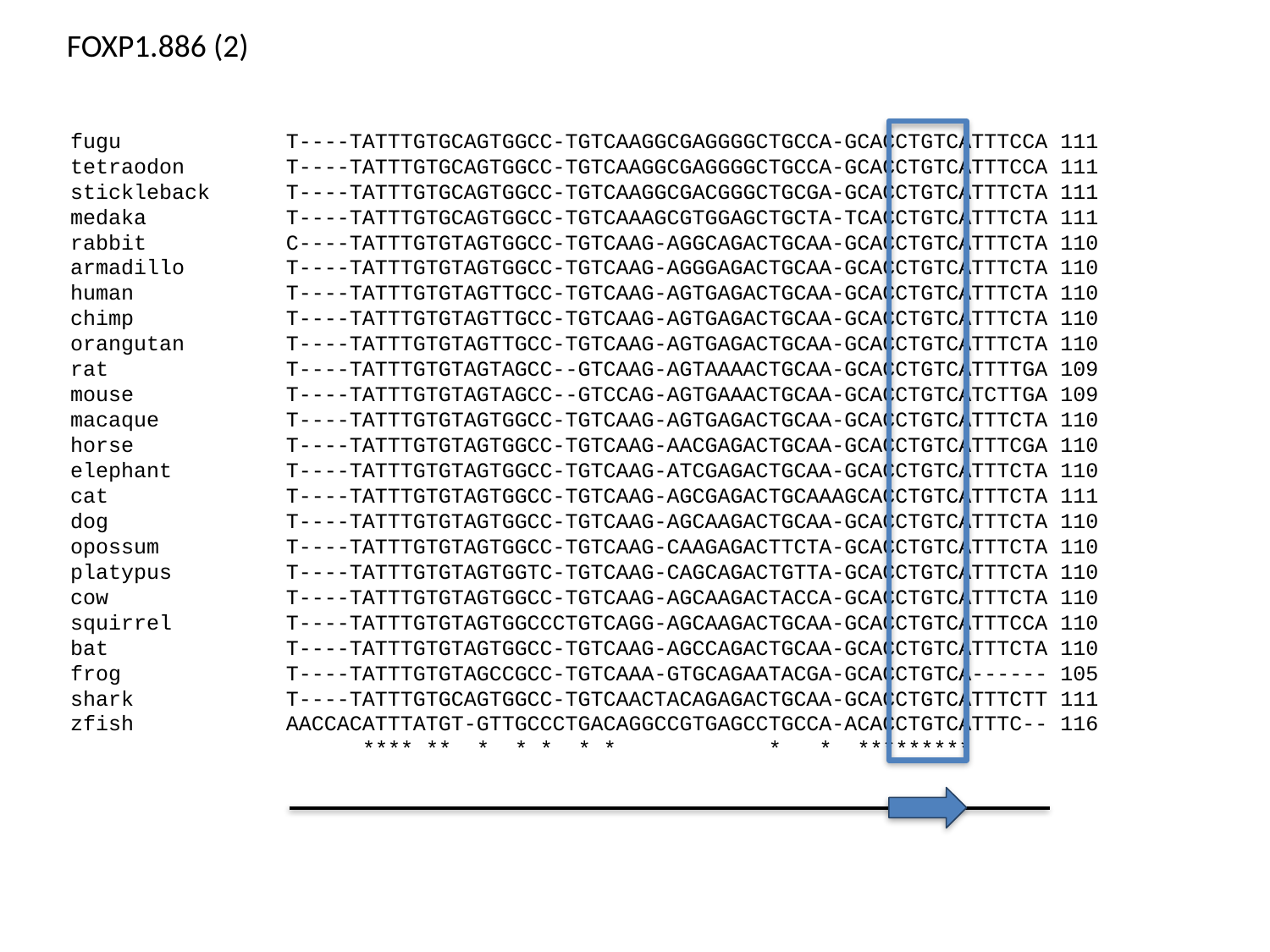

FOXP1.886 (2)
fugu T----TATTTGTGCAGTGGCC-TGTCAAGGCGAGGGGCTGCCA-GCACCTGTCATTTCCA 111
tetraodon T----TATTTGTGCAGTGGCC-TGTCAAGGCGAGGGGCTGCCA-GCACCTGTCATTTCCA 111
stickleback T----TATTTGTGCAGTGGCC-TGTCAAGGCGACGGGCTGCGA-GCACCTGTCATTTCTA 111
medaka T----TATTTGTGCAGTGGCC-TGTCAAAGCGTGGAGCTGCTA-TCACCTGTCATTTCTA 111
rabbit C----TATTTGTGTAGTGGCC-TGTCAAG-AGGCAGACTGCAA-GCACCTGTCATTTCTA 110
armadillo T----TATTTGTGTAGTGGCC-TGTCAAG-AGGGAGACTGCAA-GCACCTGTCATTTCTA 110
human T----TATTTGTGTAGTTGCC-TGTCAAG-AGTGAGACTGCAA-GCACCTGTCATTTCTA 110
chimp T----TATTTGTGTAGTTGCC-TGTCAAG-AGTGAGACTGCAA-GCACCTGTCATTTCTA 110
orangutan T----TATTTGTGTAGTTGCC-TGTCAAG-AGTGAGACTGCAA-GCACCTGTCATTTCTA 110
rat T----TATTTGTGTAGTAGCC--GTCAAG-AGTAAAACTGCAA-GCACCTGTCATTTTGA 109
mouse T----TATTTGTGTAGTAGCC--GTCCAG-AGTGAAACTGCAA-GCACCTGTCATCTTGA 109
macaque T----TATTTGTGTAGTGGCC-TGTCAAG-AGTGAGACTGCAA-GCACCTGTCATTTCTA 110
horse T----TATTTGTGTAGTGGCC-TGTCAAG-AACGAGACTGCAA-GCACCTGTCATTTCGA 110
elephant T----TATTTGTGTAGTGGCC-TGTCAAG-ATCGAGACTGCAA-GCACCTGTCATTTCTA 110
cat T----TATTTGTGTAGTGGCC-TGTCAAG-AGCGAGACTGCAAAGCACCTGTCATTTCTA 111
dog T----TATTTGTGTAGTGGCC-TGTCAAG-AGCAAGACTGCAA-GCACCTGTCATTTCTA 110
opossum T----TATTTGTGTAGTGGCC-TGTCAAG-CAAGAGACTTCTA-GCACCTGTCATTTCTA 110
platypus T----TATTTGTGTAGTGGTC-TGTCAAG-CAGCAGACTGTTA-GCACCTGTCATTTCTA 110
cow T----TATTTGTGTAGTGGCC-TGTCAAG-AGCAAGACTACCA-GCACCTGTCATTTCTA 110
squirrel T----TATTTGTGTAGTGGCCCTGTCAGG-AGCAAGACTGCAA-GCACCTGTCATTTCCA 110
bat T----TATTTGTGTAGTGGCC-TGTCAAG-AGCCAGACTGCAA-GCACCTGTCATTTCTA 110
frog T----TATTTGTGTAGCCGCC-TGTCAAA-GTGCAGAATACGA-GCACCTGTCA------ 105
shark T----TATTTGTGCAGTGGCC-TGTCAACTACAGAGACTGCAA-GCACCTGTCATTTCTT 111
zfish AACCACATTTATGT-GTTGCCCTGACAGGCCGTGAGCCTGCCA-ACACCTGTCATTTC-- 116
 **** ** * * * * * * * *********

## Slide 6
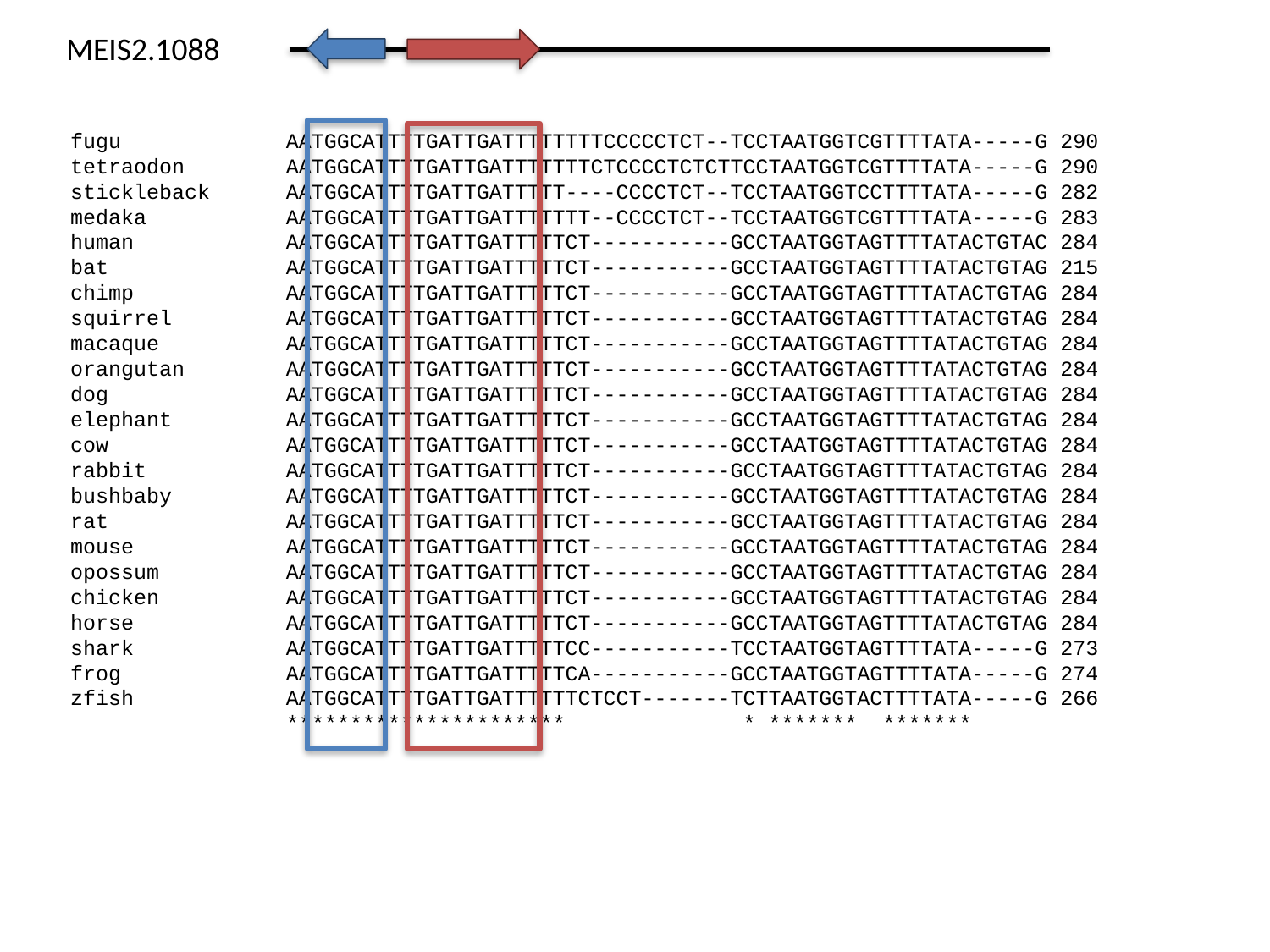

MEIS2.1088
fugu AATGGCATTTTGATTGATTTTTTTTCCCCCTCT--TCCTAATGGTCGTTTTATA-----G 290
tetraodon AATGGCATTTTGATTGATTTTTTTCTCCCCTCTCTTCCTAATGGTCGTTTTATA-----G 290
stickleback AATGGCATTTTGATTGATTTTT----CCCCTCT--TCCTAATGGTCCTTTTATA-----G 282
medaka AATGGCATTTTGATTGATTTTTTT--CCCCTCT--TCCTAATGGTCGTTTTATA-----G 283
human AATGGCATTTTGATTGATTTTTCT-----------GCCTAATGGTAGTTTTATACTGTAC 284
bat AATGGCATTTTGATTGATTTTTCT-----------GCCTAATGGTAGTTTTATACTGTAG 215
chimp AATGGCATTTTGATTGATTTTTCT-----------GCCTAATGGTAGTTTTATACTGTAG 284
squirrel AATGGCATTTTGATTGATTTTTCT-----------GCCTAATGGTAGTTTTATACTGTAG 284
macaque AATGGCATTTTGATTGATTTTTCT-----------GCCTAATGGTAGTTTTATACTGTAG 284
orangutan AATGGCATTTTGATTGATTTTTCT-----------GCCTAATGGTAGTTTTATACTGTAG 284
dog AATGGCATTTTGATTGATTTTTCT-----------GCCTAATGGTAGTTTTATACTGTAG 284
elephant AATGGCATTTTGATTGATTTTTCT-----------GCCTAATGGTAGTTTTATACTGTAG 284
cow AATGGCATTTTGATTGATTTTTCT-----------GCCTAATGGTAGTTTTATACTGTAG 284
rabbit AATGGCATTTTGATTGATTTTTCT-----------GCCTAATGGTAGTTTTATACTGTAG 284
bushbaby AATGGCATTTTGATTGATTTTTCT-----------GCCTAATGGTAGTTTTATACTGTAG 284
rat AATGGCATTTTGATTGATTTTTCT-----------GCCTAATGGTAGTTTTATACTGTAG 284
mouse AATGGCATTTTGATTGATTTTTCT-----------GCCTAATGGTAGTTTTATACTGTAG 284
opossum AATGGCATTTTGATTGATTTTTCT-----------GCCTAATGGTAGTTTTATACTGTAG 284
chicken AATGGCATTTTGATTGATTTTTCT-----------GCCTAATGGTAGTTTTATACTGTAG 284
horse AATGGCATTTTGATTGATTTTTCT-----------GCCTAATGGTAGTTTTATACTGTAG 284
shark AATGGCATTTTGATTGATTTTTCC-----------TCCTAATGGTAGTTTTATA-----G 273
frog AATGGCATTTTGATTGATTTTTCA-----------GCCTAATGGTAGTTTTATA-----G 274
zfish AATGGCATTTTGATTGATTTTTTCTCCT-------TCTTAATGGTACTTTTATA-----G 266
 ********************** * ******* *******

## Slide 7
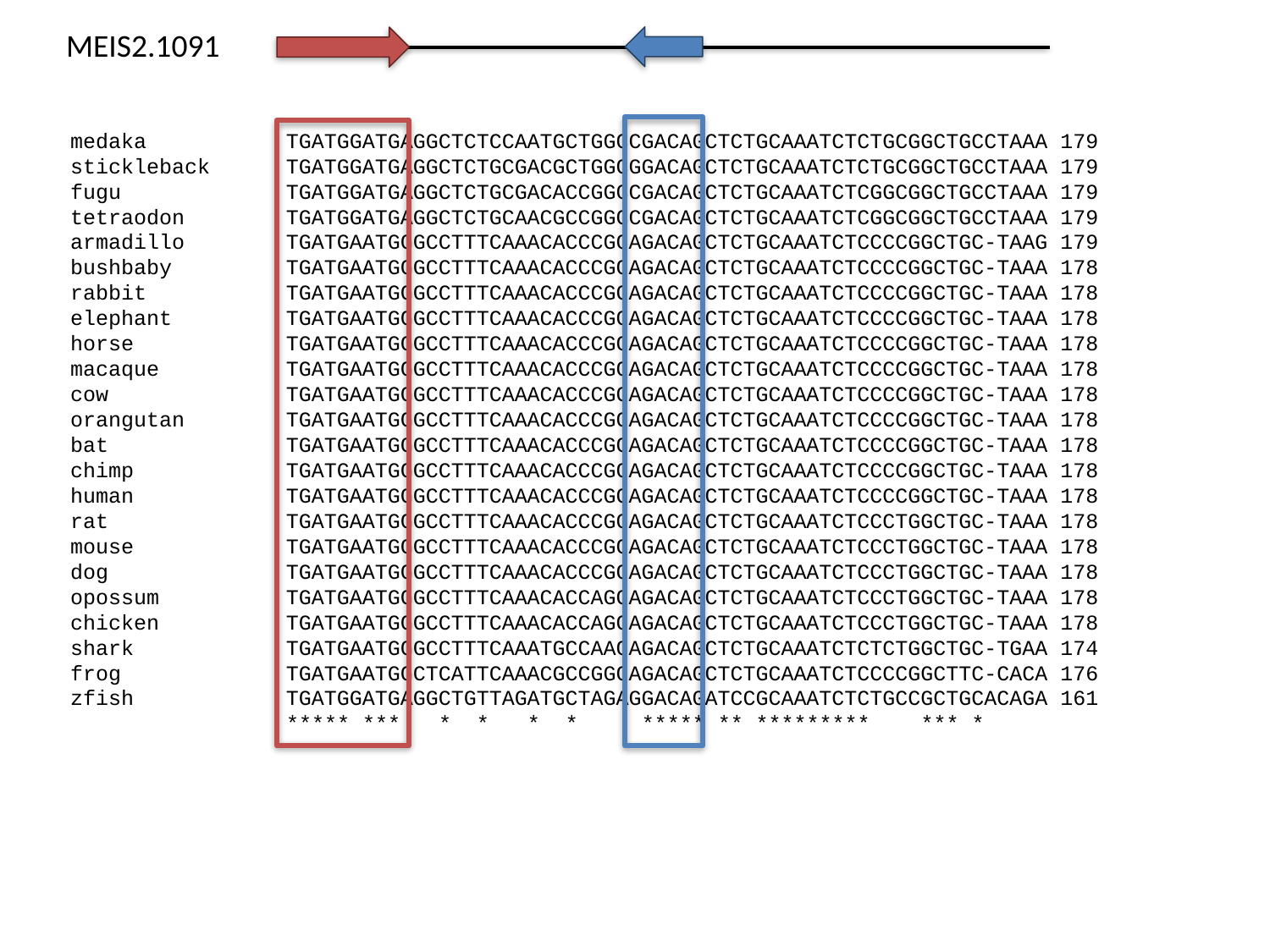

MEIS2.1091
medaka TGATGGATGAGGCTCTCCAATGCTGGCCGACAGCTCTGCAAATCTCTGCGGCTGCCTAAA 179
stickleback TGATGGATGAGGCTCTGCGACGCTGGCGGACAGCTCTGCAAATCTCTGCGGCTGCCTAAA 179
fugu TGATGGATGAGGCTCTGCGACACCGGCCGACAGCTCTGCAAATCTCGGCGGCTGCCTAAA 179
tetraodon TGATGGATGAGGCTCTGCAACGCCGGCCGACAGCTCTGCAAATCTCGGCGGCTGCCTAAA 179
armadillo TGATGAATGGGCCTTTCAAACACCCGGAGACAGCTCTGCAAATCTCCCCGGCTGC-TAAG 179
bushbaby TGATGAATGGGCCTTTCAAACACCCGGAGACAGCTCTGCAAATCTCCCCGGCTGC-TAAA 178
rabbit TGATGAATGGGCCTTTCAAACACCCGGAGACAGCTCTGCAAATCTCCCCGGCTGC-TAAA 178
elephant TGATGAATGGGCCTTTCAAACACCCGGAGACAGCTCTGCAAATCTCCCCGGCTGC-TAAA 178
horse TGATGAATGGGCCTTTCAAACACCCGGAGACAGCTCTGCAAATCTCCCCGGCTGC-TAAA 178
macaque TGATGAATGGGCCTTTCAAACACCCGGAGACAGCTCTGCAAATCTCCCCGGCTGC-TAAA 178
cow TGATGAATGGGCCTTTCAAACACCCGGAGACAGCTCTGCAAATCTCCCCGGCTGC-TAAA 178
orangutan TGATGAATGGGCCTTTCAAACACCCGGAGACAGCTCTGCAAATCTCCCCGGCTGC-TAAA 178
bat TGATGAATGGGCCTTTCAAACACCCGGAGACAGCTCTGCAAATCTCCCCGGCTGC-TAAA 178
chimp TGATGAATGGGCCTTTCAAACACCCGGAGACAGCTCTGCAAATCTCCCCGGCTGC-TAAA 178
human TGATGAATGGGCCTTTCAAACACCCGGAGACAGCTCTGCAAATCTCCCCGGCTGC-TAAA 178
rat TGATGAATGGGCCTTTCAAACACCCGGAGACAGCTCTGCAAATCTCCCTGGCTGC-TAAA 178
mouse TGATGAATGGGCCTTTCAAACACCCGGAGACAGCTCTGCAAATCTCCCTGGCTGC-TAAA 178
dog TGATGAATGGGCCTTTCAAACACCCGGAGACAGCTCTGCAAATCTCCCTGGCTGC-TAAA 178
opossum TGATGAATGGGCCTTTCAAACACCAGGAGACAGCTCTGCAAATCTCCCTGGCTGC-TAAA 178
chicken TGATGAATGGGCCTTTCAAACACCAGGAGACAGCTCTGCAAATCTCCCTGGCTGC-TAAA 178
shark TGATGAATGGGCCTTTCAAATGCCAAGAGACAGCTCTGCAAATCTCTCTGGCTGC-TGAA 174
frog TGATGAATGGCTCATTCAAACGCCGGGAGACAGCTCTGCAAATCTCCCCGGCTTC-CACA 176
zfish TGATGGATGAGGCTGTTAGATGCTAGAGGACAGATCCGCAAATCTCTGCCGCTGCACAGA 161
 ***** *** * * * * ***** ** ********* *** *

## Slide 8
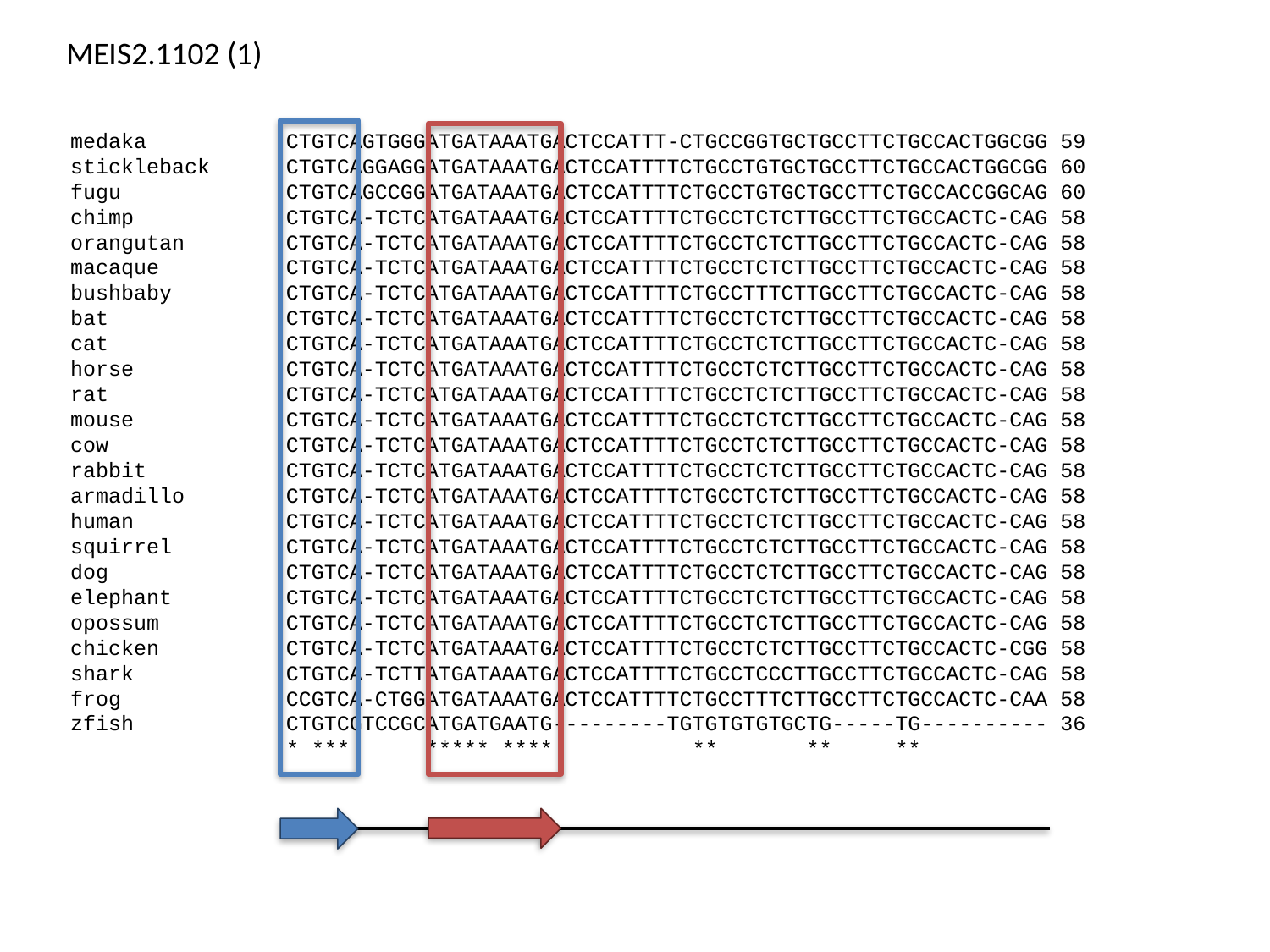

MEIS2.1102 (1)
medaka CTGTCAGTGGGATGATAAATGACTCCATTT-CTGCCGGTGCTGCCTTCTGCCACTGGCGG 59
stickleback CTGTCAGGAGGATGATAAATGACTCCATTTTCTGCCTGTGCTGCCTTCTGCCACTGGCGG 60
fugu CTGTCAGCCGGATGATAAATGACTCCATTTTCTGCCTGTGCTGCCTTCTGCCACCGGCAG 60
chimp CTGTCA-TCTCATGATAAATGACTCCATTTTCTGCCTCTCTTGCCTTCTGCCACTC-CAG 58
orangutan CTGTCA-TCTCATGATAAATGACTCCATTTTCTGCCTCTCTTGCCTTCTGCCACTC-CAG 58
macaque CTGTCA-TCTCATGATAAATGACTCCATTTTCTGCCTCTCTTGCCTTCTGCCACTC-CAG 58
bushbaby CTGTCA-TCTCATGATAAATGACTCCATTTTCTGCCTTTCTTGCCTTCTGCCACTC-CAG 58
bat CTGTCA-TCTCATGATAAATGACTCCATTTTCTGCCTCTCTTGCCTTCTGCCACTC-CAG 58
cat CTGTCA-TCTCATGATAAATGACTCCATTTTCTGCCTCTCTTGCCTTCTGCCACTC-CAG 58
horse CTGTCA-TCTCATGATAAATGACTCCATTTTCTGCCTCTCTTGCCTTCTGCCACTC-CAG 58
rat CTGTCA-TCTCATGATAAATGACTCCATTTTCTGCCTCTCTTGCCTTCTGCCACTC-CAG 58
mouse CTGTCA-TCTCATGATAAATGACTCCATTTTCTGCCTCTCTTGCCTTCTGCCACTC-CAG 58
cow CTGTCA-TCTCATGATAAATGACTCCATTTTCTGCCTCTCTTGCCTTCTGCCACTC-CAG 58
rabbit CTGTCA-TCTCATGATAAATGACTCCATTTTCTGCCTCTCTTGCCTTCTGCCACTC-CAG 58
armadillo CTGTCA-TCTCATGATAAATGACTCCATTTTCTGCCTCTCTTGCCTTCTGCCACTC-CAG 58
human CTGTCA-TCTCATGATAAATGACTCCATTTTCTGCCTCTCTTGCCTTCTGCCACTC-CAG 58
squirrel CTGTCA-TCTCATGATAAATGACTCCATTTTCTGCCTCTCTTGCCTTCTGCCACTC-CAG 58
dog CTGTCA-TCTCATGATAAATGACTCCATTTTCTGCCTCTCTTGCCTTCTGCCACTC-CAG 58
elephant CTGTCA-TCTCATGATAAATGACTCCATTTTCTGCCTCTCTTGCCTTCTGCCACTC-CAG 58
opossum CTGTCA-TCTCATGATAAATGACTCCATTTTCTGCCTCTCTTGCCTTCTGCCACTC-CAG 58
chicken CTGTCA-TCTCATGATAAATGACTCCATTTTCTGCCTCTCTTGCCTTCTGCCACTC-CGG 58
shark CTGTCA-TCTTATGATAAATGACTCCATTTTCTGCCTCCCTTGCCTTCTGCCACTC-CAG 58
frog CCGTCA-CTGGATGATAAATGACTCCATTTTCTGCCTTTCTTGCCTTCTGCCACTC-CAA 58
zfish CTGTCGTCCGCATGATGAATG---------TGTGTGTGTGCTG-----TG---------- 36
 * *** ***** **** ** ** **

## Slide 9
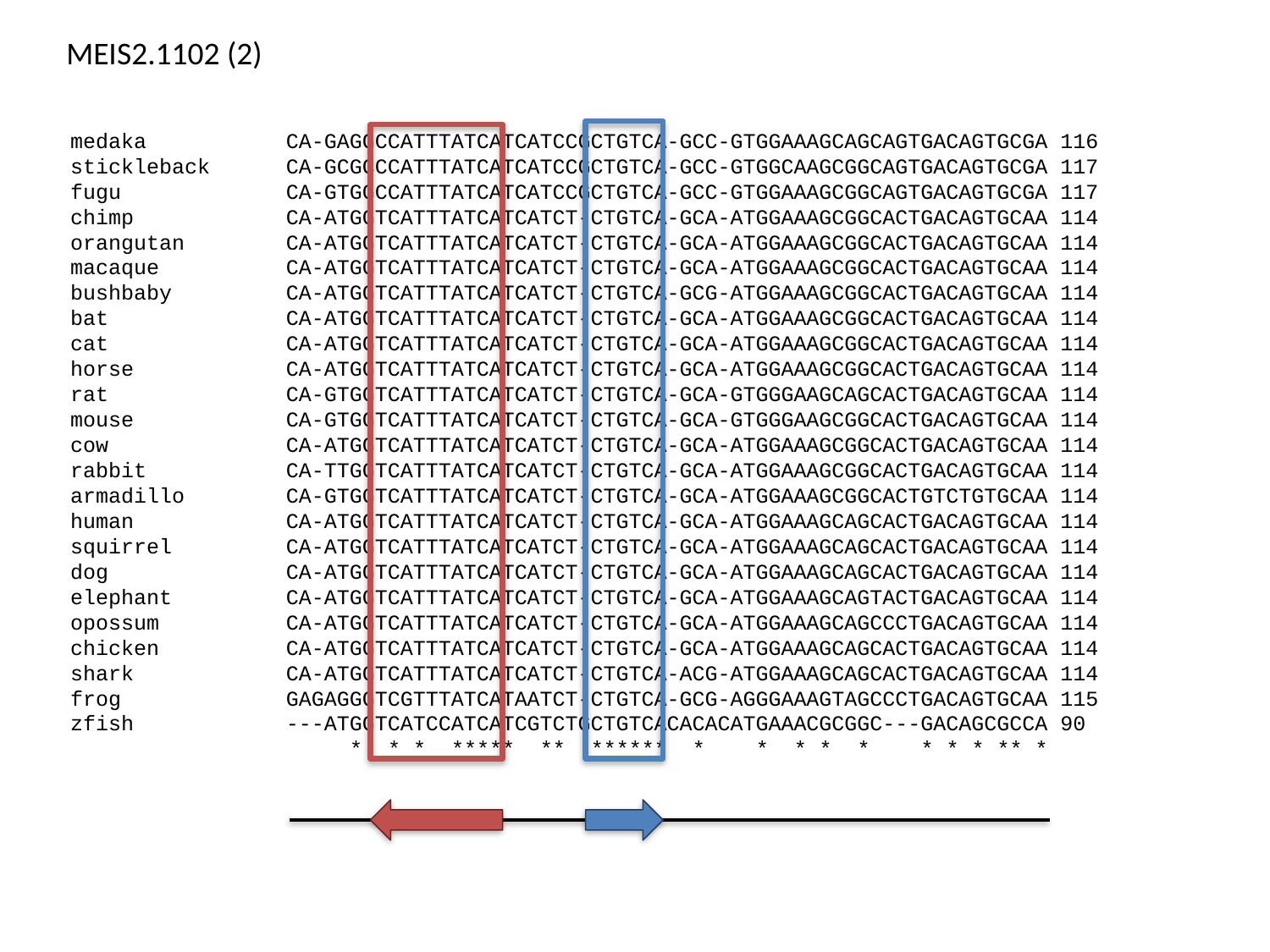

MEIS2.1102 (2)
medaka CA-GAGGCCATTTATCATCATCCGCTGTCA-GCC-GTGGAAAGCAGCAGTGACAGTGCGA 116
stickleback CA-GCGCCCATTTATCATCATCCGCTGTCA-GCC-GTGGCAAGCGGCAGTGACAGTGCGA 117
fugu CA-GTGGCCATTTATCATCATCCGCTGTCA-GCC-GTGGAAAGCGGCAGTGACAGTGCGA 117
chimp CA-ATGGTCATTTATCATCATCT-CTGTCA-GCA-ATGGAAAGCGGCACTGACAGTGCAA 114
orangutan CA-ATGGTCATTTATCATCATCT-CTGTCA-GCA-ATGGAAAGCGGCACTGACAGTGCAA 114
macaque CA-ATGGTCATTTATCATCATCT-CTGTCA-GCA-ATGGAAAGCGGCACTGACAGTGCAA 114
bushbaby CA-ATGGTCATTTATCATCATCT-CTGTCA-GCG-ATGGAAAGCGGCACTGACAGTGCAA 114
bat CA-ATGGTCATTTATCATCATCT-CTGTCA-GCA-ATGGAAAGCGGCACTGACAGTGCAA 114
cat CA-ATGGTCATTTATCATCATCT-CTGTCA-GCA-ATGGAAAGCGGCACTGACAGTGCAA 114
horse CA-ATGGTCATTTATCATCATCT-CTGTCA-GCA-ATGGAAAGCGGCACTGACAGTGCAA 114
rat CA-GTGGTCATTTATCATCATCT-CTGTCA-GCA-GTGGGAAGCAGCACTGACAGTGCAA 114
mouse CA-GTGGTCATTTATCATCATCT-CTGTCA-GCA-GTGGGAAGCGGCACTGACAGTGCAA 114
cow CA-ATGGTCATTTATCATCATCT-CTGTCA-GCA-ATGGAAAGCGGCACTGACAGTGCAA 114
rabbit CA-TTGGTCATTTATCATCATCT-CTGTCA-GCA-ATGGAAAGCGGCACTGACAGTGCAA 114
armadillo CA-GTGGTCATTTATCATCATCT-CTGTCA-GCA-ATGGAAAGCGGCACTGTCTGTGCAA 114
human CA-ATGGTCATTTATCATCATCT-CTGTCA-GCA-ATGGAAAGCAGCACTGACAGTGCAA 114
squirrel CA-ATGGTCATTTATCATCATCT-CTGTCA-GCA-ATGGAAAGCAGCACTGACAGTGCAA 114
dog CA-ATGGTCATTTATCATCATCT-CTGTCA-GCA-ATGGAAAGCAGCACTGACAGTGCAA 114
elephant CA-ATGGTCATTTATCATCATCT-CTGTCA-GCA-ATGGAAAGCAGTACTGACAGTGCAA 114
opossum CA-ATGGTCATTTATCATCATCT-CTGTCA-GCA-ATGGAAAGCAGCCCTGACAGTGCAA 114
chicken CA-ATGGTCATTTATCATCATCT-CTGTCA-GCA-ATGGAAAGCAGCACTGACAGTGCAA 114
shark CA-ATGGTCATTTATCATCATCT-CTGTCA-ACG-ATGGAAAGCAGCACTGACAGTGCAA 114
frog GAGAGGGTCGTTTATCATAATCT-CTGTCA-GCG-AGGGAAAGTAGCCCTGACAGTGCAA 115
zfish ---ATGGTCATCCATCATCGTCTGCTGTCACACACATGAAACGCGGC---GACAGCGCCA 90
 * * * ***** ** ****** * * * * * * * * ** *

## Slide 10
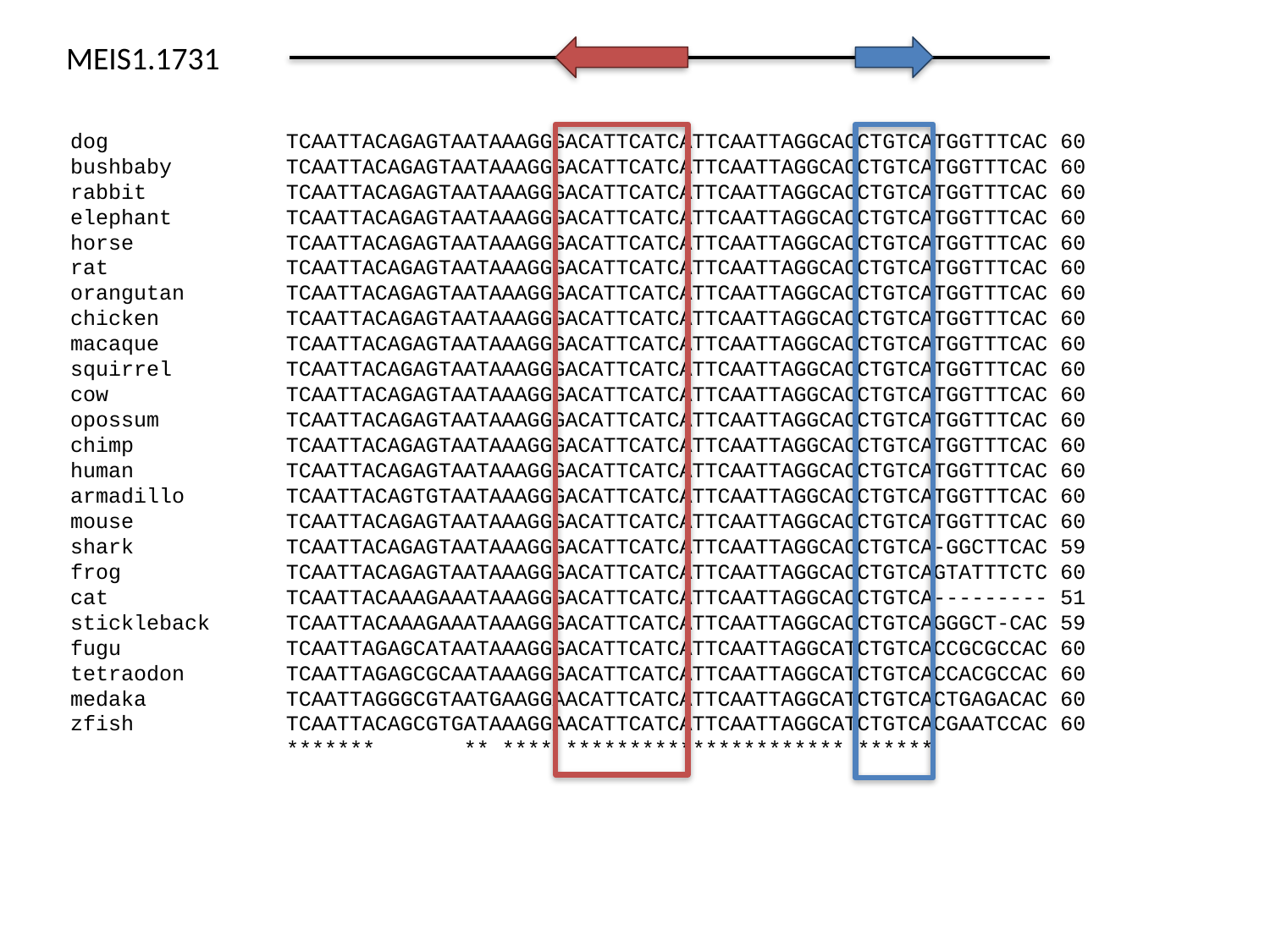

MEIS1.1731
dog TCAATTACAGAGTAATAAAGGGACATTCATCATTCAATTAGGCACCTGTCATGGTTTCAC 60
bushbaby TCAATTACAGAGTAATAAAGGGACATTCATCATTCAATTAGGCACCTGTCATGGTTTCAC 60
rabbit TCAATTACAGAGTAATAAAGGGACATTCATCATTCAATTAGGCACCTGTCATGGTTTCAC 60
elephant TCAATTACAGAGTAATAAAGGGACATTCATCATTCAATTAGGCACCTGTCATGGTTTCAC 60
horse TCAATTACAGAGTAATAAAGGGACATTCATCATTCAATTAGGCACCTGTCATGGTTTCAC 60
rat TCAATTACAGAGTAATAAAGGGACATTCATCATTCAATTAGGCACCTGTCATGGTTTCAC 60
orangutan TCAATTACAGAGTAATAAAGGGACATTCATCATTCAATTAGGCACCTGTCATGGTTTCAC 60
chicken TCAATTACAGAGTAATAAAGGGACATTCATCATTCAATTAGGCACCTGTCATGGTTTCAC 60
macaque TCAATTACAGAGTAATAAAGGGACATTCATCATTCAATTAGGCACCTGTCATGGTTTCAC 60
squirrel TCAATTACAGAGTAATAAAGGGACATTCATCATTCAATTAGGCACCTGTCATGGTTTCAC 60
cow TCAATTACAGAGTAATAAAGGGACATTCATCATTCAATTAGGCACCTGTCATGGTTTCAC 60
opossum TCAATTACAGAGTAATAAAGGGACATTCATCATTCAATTAGGCACCTGTCATGGTTTCAC 60
chimp TCAATTACAGAGTAATAAAGGGACATTCATCATTCAATTAGGCACCTGTCATGGTTTCAC 60
human TCAATTACAGAGTAATAAAGGGACATTCATCATTCAATTAGGCACCTGTCATGGTTTCAC 60
armadillo TCAATTACAGTGTAATAAAGGGACATTCATCATTCAATTAGGCACCTGTCATGGTTTCAC 60
mouse TCAATTACAGAGTAATAAAGGGACATTCATCATTCAATTAGGCACCTGTCATGGTTTCAC 60
shark TCAATTACAGAGTAATAAAGGGACATTCATCATTCAATTAGGCACCTGTCA-GGCTTCAC 59
frog TCAATTACAGAGTAATAAAGGGACATTCATCATTCAATTAGGCACCTGTCAGTATTTCTC 60
cat TCAATTACAAAGAAATAAAGGGACATTCATCATTCAATTAGGCACCTGTCA--------- 51
stickleback TCAATTACAAAGAAATAAAGGGACATTCATCATTCAATTAGGCACCTGTCAGGGCT-CAC 59
fugu TCAATTAGAGCATAATAAAGGGACATTCATCATTCAATTAGGCATCTGTCACCGCGCCAC 60
tetraodon TCAATTAGAGCGCAATAAAGGGACATTCATCATTCAATTAGGCATCTGTCACCACGCCAC 60
medaka TCAATTAGGGCGTAATGAAGGAACATTCATCATTCAATTAGGCATCTGTCACTGAGACAC 60
zfish TCAATTACAGCGTGATAAAGGAACATTCATCATTCAATTAGGCATCTGTCACGAATCCAC 60
 ******* ** **** ********************** ******

## Slide 11
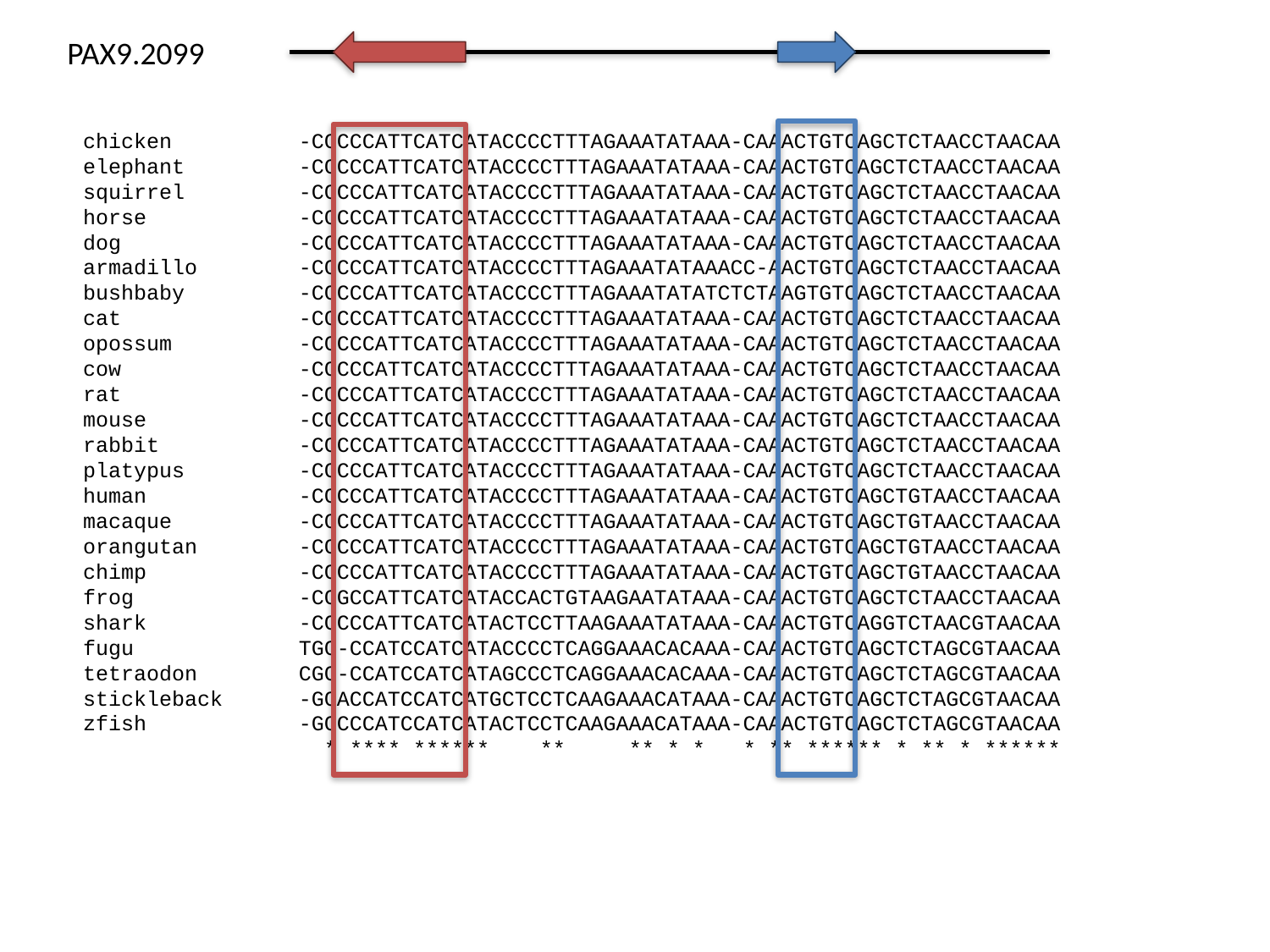

PAX9.2099
chicken -CCCCCATTCATCATACCCCTTTAGAAATATAAA-CAAACTGTCAGCTCTAACCTAACAA
elephant -CCCCCATTCATCATACCCCTTTAGAAATATAAA-CAAACTGTCAGCTCTAACCTAACAA
squirrel -CCCCCATTCATCATACCCCTTTAGAAATATAAA-CAAACTGTCAGCTCTAACCTAACAA
horse -CCCCCATTCATCATACCCCTTTAGAAATATAAA-CAAACTGTCAGCTCTAACCTAACAA
dog -CCCCCATTCATCATACCCCTTTAGAAATATAAA-CAAACTGTCAGCTCTAACCTAACAA
armadillo -CCCCCATTCATCATACCCCTTTAGAAATATAAACC-AACTGTCAGCTCTAACCTAACAA
bushbaby -CCCCCATTCATCATACCCCTTTAGAAATATATCTCTAAGTGTCAGCTCTAACCTAACAA
cat -CCCCCATTCATCATACCCCTTTAGAAATATAAA-CAAACTGTCAGCTCTAACCTAACAA
opossum -CCCCCATTCATCATACCCCTTTAGAAATATAAA-CAAACTGTCAGCTCTAACCTAACAA
cow -CCCCCATTCATCATACCCCTTTAGAAATATAAA-CAAACTGTCAGCTCTAACCTAACAA
rat -CCCCCATTCATCATACCCCTTTAGAAATATAAA-CAAACTGTCAGCTCTAACCTAACAA
mouse -CCCCCATTCATCATACCCCTTTAGAAATATAAA-CAAACTGTCAGCTCTAACCTAACAA
rabbit -CCCCCATTCATCATACCCCTTTAGAAATATAAA-CAAACTGTCAGCTCTAACCTAACAA
platypus -CCCCCATTCATCATACCCCTTTAGAAATATAAA-CAAACTGTCAGCTCTAACCTAACAA
human -CCCCCATTCATCATACCCCTTTAGAAATATAAA-CAAACTGTCAGCTGTAACCTAACAA
macaque -CCCCCATTCATCATACCCCTTTAGAAATATAAA-CAAACTGTCAGCTGTAACCTAACAA
orangutan -CCCCCATTCATCATACCCCTTTAGAAATATAAA-CAAACTGTCAGCTGTAACCTAACAA
chimp -CCCCCATTCATCATACCCCTTTAGAAATATAAA-CAAACTGTCAGCTGTAACCTAACAA
frog -CCGCCATTCATCATACCACTGTAAGAATATAAA-CAAACTGTCAGCTCTAACCTAACAA
shark -CCCCCATTCATCATACTCCTTAAGAAATATAAA-CAAACTGTCAGGTCTAACGTAACAA
fugu TGC-CCATCCATCATACCCCTCAGGAAACACAAA-CAAACTGTCAGCTCTAGCGTAACAA
tetraodon CGC-CCATCCATCATAGCCCTCAGGAAACACAAA-CAAACTGTCAGCTCTAGCGTAACAA
stickleback -GCACCATCCATCATGCTCCTCAAGAAACATAAA-CAAACTGTCAGCTCTAGCGTAACAA
zfish -GCCCCATCCATCATACTCCTCAAGAAACATAAA-CAAACTGTCAGCTCTAGCGTAACAA
 * **** ****** ** ** * * * ** ****** * ** * ******

## Slide 12
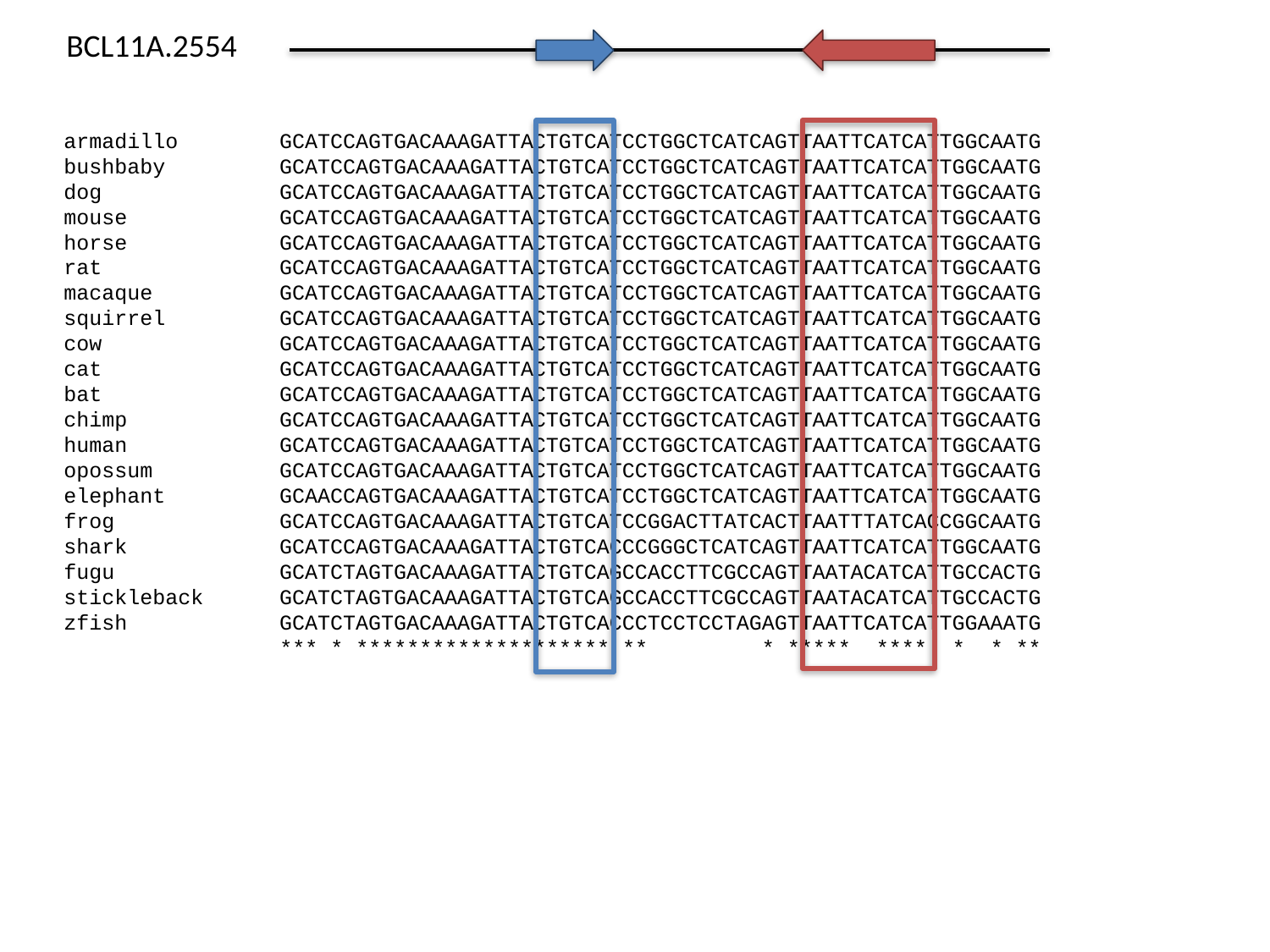

BCL11A.2554
armadillo GCATCCAGTGACAAAGATTACTGTCATCCTGGCTCATCAGTTAATTCATCATTGGCAATG
bushbaby GCATCCAGTGACAAAGATTACTGTCATCCTGGCTCATCAGTTAATTCATCATTGGCAATG
dog GCATCCAGTGACAAAGATTACTGTCATCCTGGCTCATCAGTTAATTCATCATTGGCAATG
mouse GCATCCAGTGACAAAGATTACTGTCATCCTGGCTCATCAGTTAATTCATCATTGGCAATG
horse GCATCCAGTGACAAAGATTACTGTCATCCTGGCTCATCAGTTAATTCATCATTGGCAATG
rat GCATCCAGTGACAAAGATTACTGTCATCCTGGCTCATCAGTTAATTCATCATTGGCAATG
macaque GCATCCAGTGACAAAGATTACTGTCATCCTGGCTCATCAGTTAATTCATCATTGGCAATG
squirrel GCATCCAGTGACAAAGATTACTGTCATCCTGGCTCATCAGTTAATTCATCATTGGCAATG
cow GCATCCAGTGACAAAGATTACTGTCATCCTGGCTCATCAGTTAATTCATCATTGGCAATG
cat GCATCCAGTGACAAAGATTACTGTCATCCTGGCTCATCAGTTAATTCATCATTGGCAATG
bat GCATCCAGTGACAAAGATTACTGTCATCCTGGCTCATCAGTTAATTCATCATTGGCAATG
chimp GCATCCAGTGACAAAGATTACTGTCATCCTGGCTCATCAGTTAATTCATCATTGGCAATG
human GCATCCAGTGACAAAGATTACTGTCATCCTGGCTCATCAGTTAATTCATCATTGGCAATG
opossum GCATCCAGTGACAAAGATTACTGTCATCCTGGCTCATCAGTTAATTCATCATTGGCAATG
elephant GCAACCAGTGACAAAGATTACTGTCATCCTGGCTCATCAGTTAATTCATCATTGGCAATG
frog GCATCCAGTGACAAAGATTACTGTCATCCGGACTTATCACTTAATTTATCACCGGCAATG
shark GCATCCAGTGACAAAGATTACTGTCACCCGGGCTCATCAGTTAATTCATCATTGGCAATG
fugu GCATCTAGTGACAAAGATTACTGTCAGCCACCTTCGCCAGTTAATACATCATTGCCACTG
stickleback GCATCTAGTGACAAAGATTACTGTCAGCCACCTTCGCCAGTTAATACATCATTGCCACTG
zfish GCATCTAGTGACAAAGATTACTGTCACCCTCCTCCTAGAGTTAATTCATCATTGGAAATG
 *** * ******************** ** * ***** **** * * **

## Slide 13
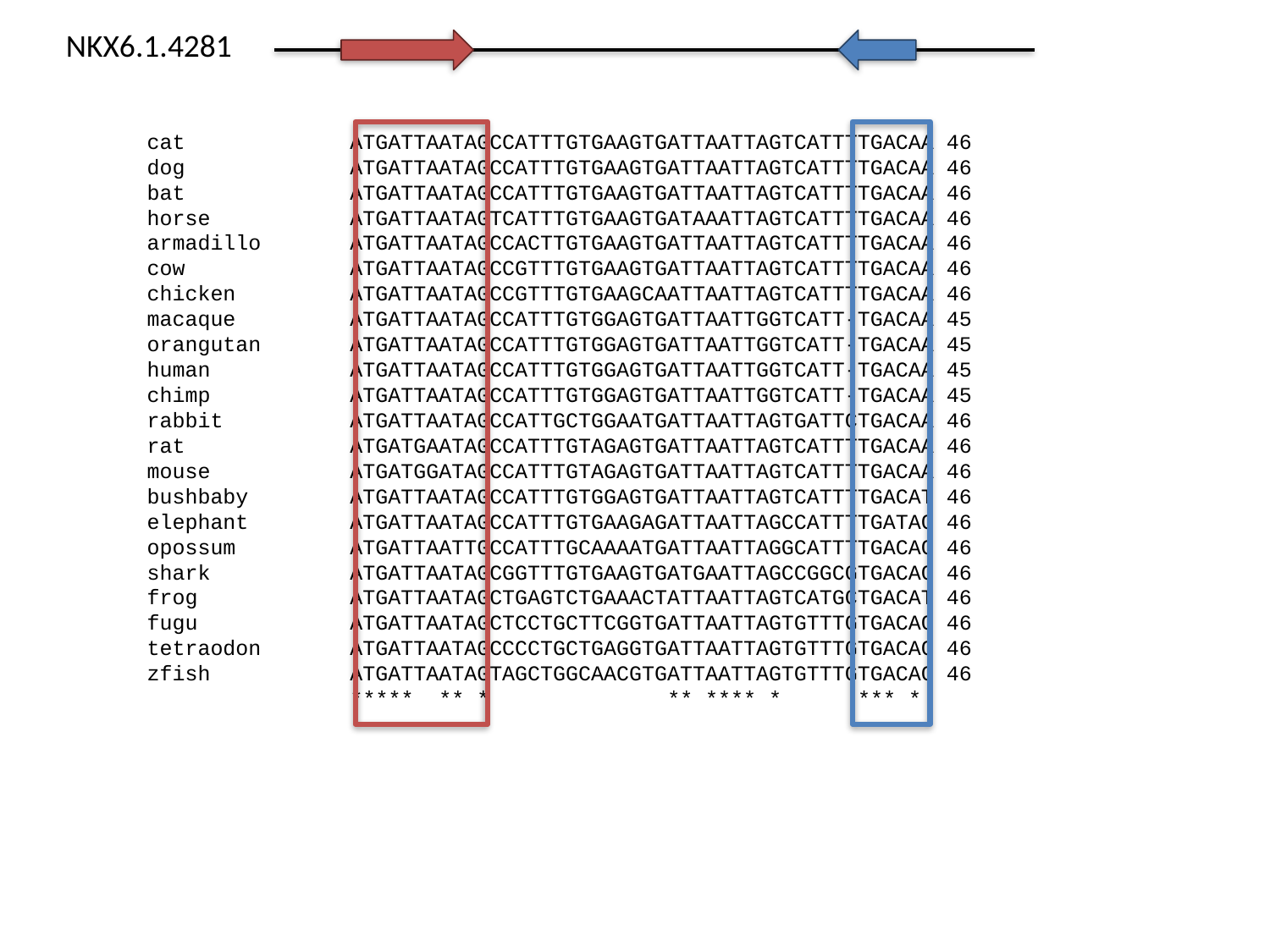

NKX6.1.4281
cat ATGATTAATAGCCATTTGTGAAGTGATTAATTAGTCATTTTGACAA 46
dog ATGATTAATAGCCATTTGTGAAGTGATTAATTAGTCATTTTGACAA 46
bat ATGATTAATAGCCATTTGTGAAGTGATTAATTAGTCATTTTGACAA 46
horse ATGATTAATAGTCATTTGTGAAGTGATAAATTAGTCATTTTGACAA 46
armadillo ATGATTAATAGCCACTTGTGAAGTGATTAATTAGTCATTTTGACAA 46
cow ATGATTAATAGCCGTTTGTGAAGTGATTAATTAGTCATTTTGACAA 46
chicken ATGATTAATAGCCGTTTGTGAAGCAATTAATTAGTCATTTTGACAA 46
macaque ATGATTAATAGCCATTTGTGGAGTGATTAATTGGTCATT-TGACAA 45
orangutan ATGATTAATAGCCATTTGTGGAGTGATTAATTGGTCATT-TGACAA 45
human ATGATTAATAGCCATTTGTGGAGTGATTAATTGGTCATT-TGACAA 45
chimp ATGATTAATAGCCATTTGTGGAGTGATTAATTGGTCATT-TGACAA 45
rabbit ATGATTAATAGCCATTGCTGGAATGATTAATTAGTGATTCTGACAA 46
rat ATGATGAATAGCCATTTGTAGAGTGATTAATTAGTCATTTTGACAA 46
mouse ATGATGGATAGCCATTTGTAGAGTGATTAATTAGTCATTTTGACAA 46
bushbaby ATGATTAATAGCCATTTGTGGAGTGATTAATTAGTCATTTTGACAT 46
elephant ATGATTAATAGCCATTTGTGAAGAGATTAATTAGCCATTTTGATAG 46
opossum ATGATTAATTGCCATTTGCAAAATGATTAATTAGGCATTTTGACAG 46
shark ATGATTAATAGCGGTTTGTGAAGTGATGAATTAGCCGGCGTGACAG 46
frog ATGATTAATAGCTGAGTCTGAAACTATTAATTAGTCATGCTGACAT 46
fugu ATGATTAATAGCTCCTGCTTCGGTGATTAATTAGTGTTTGTGACAG 46
tetraodon ATGATTAATAGCCCCTGCTGAGGTGATTAATTAGTGTTTGTGACAG 46
zfish ATGATTAATAGTAGCTGGCAACGTGATTAATTAGTGTTTGTGACAG 46
 ***** ** * ** **** * *** *

## Slide 14
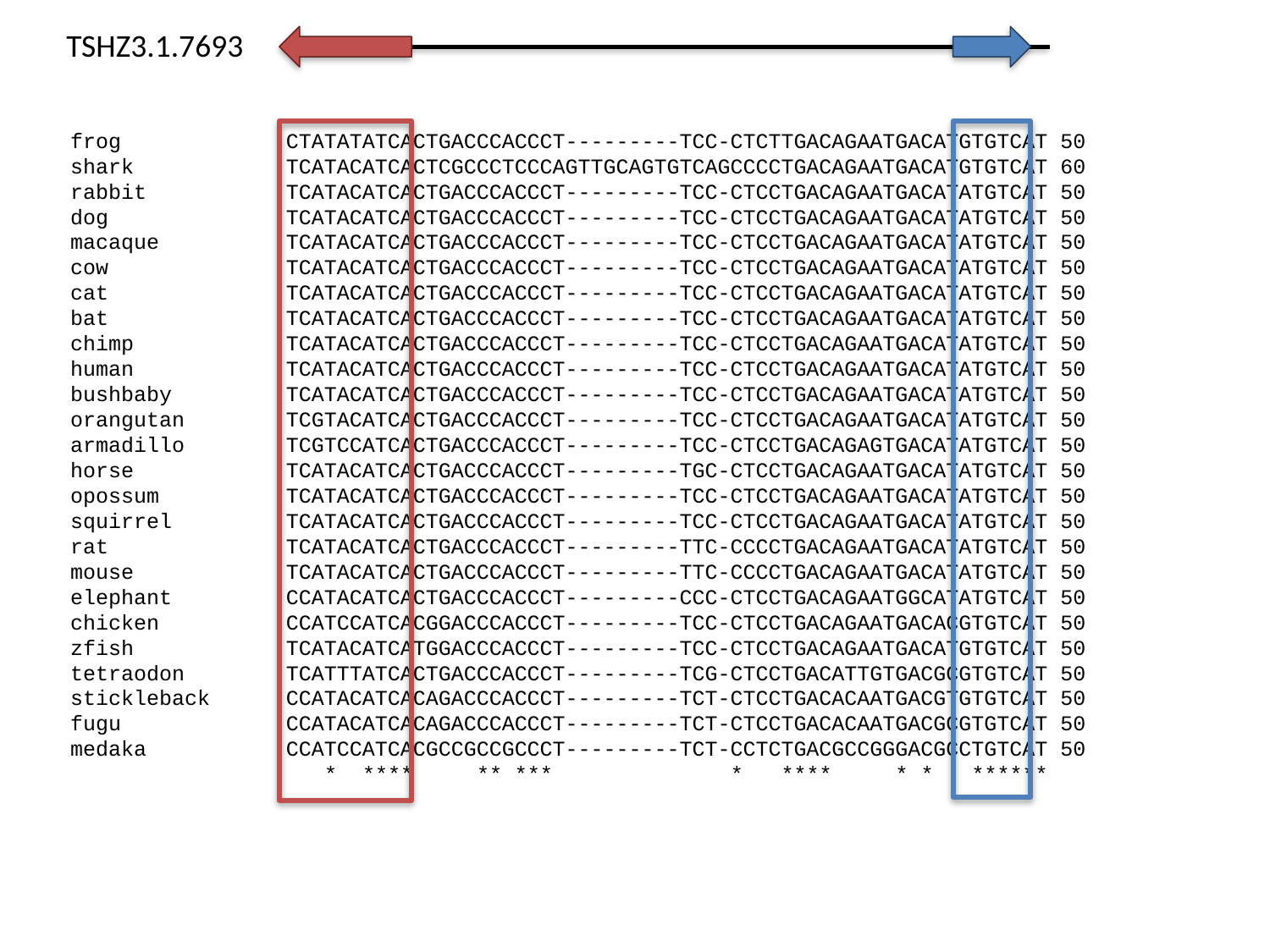

TSHZ3.1.7693
frog CTATATATCACTGACCCACCCT---------TCC-CTCTTGACAGAATGACATGTGTCAT 50
shark TCATACATCACTCGCCCTCCCAGTTGCAGTGTCAGCCCCTGACAGAATGACATGTGTCAT 60
rabbit TCATACATCACTGACCCACCCT---------TCC-CTCCTGACAGAATGACATATGTCAT 50
dog TCATACATCACTGACCCACCCT---------TCC-CTCCTGACAGAATGACATATGTCAT 50
macaque TCATACATCACTGACCCACCCT---------TCC-CTCCTGACAGAATGACATATGTCAT 50
cow TCATACATCACTGACCCACCCT---------TCC-CTCCTGACAGAATGACATATGTCAT 50
cat TCATACATCACTGACCCACCCT---------TCC-CTCCTGACAGAATGACATATGTCAT 50
bat TCATACATCACTGACCCACCCT---------TCC-CTCCTGACAGAATGACATATGTCAT 50
chimp TCATACATCACTGACCCACCCT---------TCC-CTCCTGACAGAATGACATATGTCAT 50
human TCATACATCACTGACCCACCCT---------TCC-CTCCTGACAGAATGACATATGTCAT 50
bushbaby TCATACATCACTGACCCACCCT---------TCC-CTCCTGACAGAATGACATATGTCAT 50
orangutan TCGTACATCACTGACCCACCCT---------TCC-CTCCTGACAGAATGACATATGTCAT 50
armadillo TCGTCCATCACTGACCCACCCT---------TCC-CTCCTGACAGAGTGACATATGTCAT 50
horse TCATACATCACTGACCCACCCT---------TGC-CTCCTGACAGAATGACATATGTCAT 50
opossum TCATACATCACTGACCCACCCT---------TCC-CTCCTGACAGAATGACATATGTCAT 50
squirrel TCATACATCACTGACCCACCCT---------TCC-CTCCTGACAGAATGACATATGTCAT 50
rat TCATACATCACTGACCCACCCT---------TTC-CCCCTGACAGAATGACATATGTCAT 50
mouse TCATACATCACTGACCCACCCT---------TTC-CCCCTGACAGAATGACATATGTCAT 50
elephant CCATACATCACTGACCCACCCT---------CCC-CTCCTGACAGAATGGCATATGTCAT 50
chicken CCATCCATCACGGACCCACCCT---------TCC-CTCCTGACAGAATGACACGTGTCAT 50
zfish TCATACATCATGGACCCACCCT---------TCC-CTCCTGACAGAATGACATGTGTCAT 50
tetraodon TCATTTATCACTGACCCACCCT---------TCG-CTCCTGACATTGTGACGCGTGTCAT 50
stickleback CCATACATCACAGACCCACCCT---------TCT-CTCCTGACACAATGACGTGTGTCAT 50
fugu CCATACATCACAGACCCACCCT---------TCT-CTCCTGACACAATGACGCGTGTCAT 50
medaka CCATCCATCACGCCGCCGCCCT---------TCT-CCTCTGACGCCGGGACGCCTGTCAT 50
 * **** ** *** * **** * * ******

## Slide 15
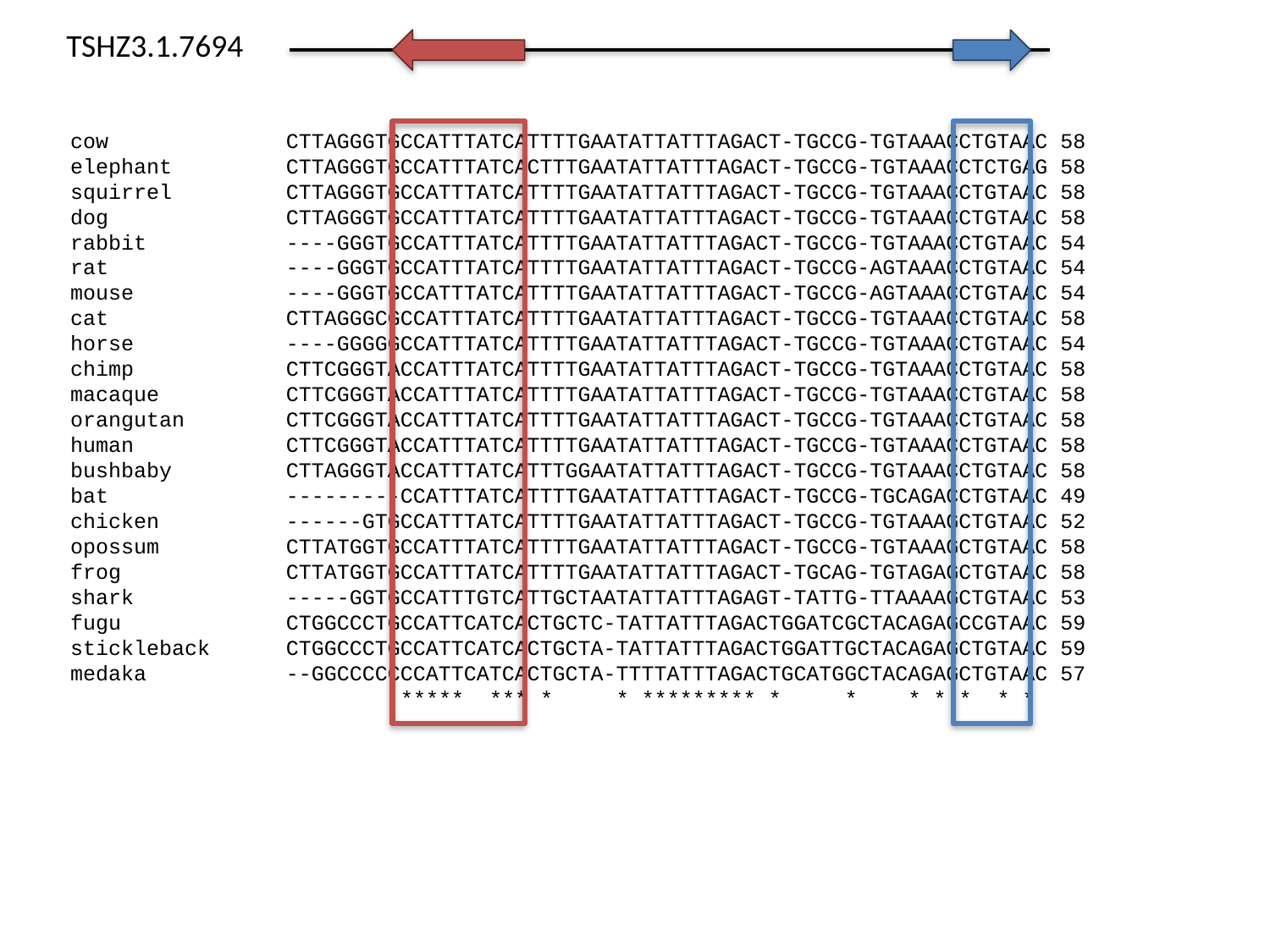

TSHZ3.1.7694
cow CTTAGGGTGCCATTTATCATTTTGAATATTATTTAGACT-TGCCG-TGTAAACCTGTAAC 58
elephant CTTAGGGTGCCATTTATCACTTTGAATATTATTTAGACT-TGCCG-TGTAAACCTCTGAG 58
squirrel CTTAGGGTGCCATTTATCATTTTGAATATTATTTAGACT-TGCCG-TGTAAACCTGTAAC 58
dog CTTAGGGTGCCATTTATCATTTTGAATATTATTTAGACT-TGCCG-TGTAAACCTGTAAC 58
rabbit ----GGGTGCCATTTATCATTTTGAATATTATTTAGACT-TGCCG-TGTAAACCTGTAAC 54
rat ----GGGTGCCATTTATCATTTTGAATATTATTTAGACT-TGCCG-AGTAAACCTGTAAC 54
mouse ----GGGTGCCATTTATCATTTTGAATATTATTTAGACT-TGCCG-AGTAAACCTGTAAC 54
cat CTTAGGGCGCCATTTATCATTTTGAATATTATTTAGACT-TGCCG-TGTAAACCTGTAAC 58
horse ----GGGGGCCATTTATCATTTTGAATATTATTTAGACT-TGCCG-TGTAAACCTGTAAC 54
chimp CTTCGGGTACCATTTATCATTTTGAATATTATTTAGACT-TGCCG-TGTAAACCTGTAAC 58
macaque CTTCGGGTACCATTTATCATTTTGAATATTATTTAGACT-TGCCG-TGTAAACCTGTAAC 58
orangutan CTTCGGGTACCATTTATCATTTTGAATATTATTTAGACT-TGCCG-TGTAAACCTGTAAC 58
human CTTCGGGTACCATTTATCATTTTGAATATTATTTAGACT-TGCCG-TGTAAACCTGTAAC 58
bushbaby CTTAGGGTACCATTTATCATTTGGAATATTATTTAGACT-TGCCG-TGTAAACCTGTAAC 58
bat ---------CCATTTATCATTTTGAATATTATTTAGACT-TGCCG-TGCAGACCTGTAAC 49
chicken ------GTGCCATTTATCATTTTGAATATTATTTAGACT-TGCCG-TGTAAAGCTGTAAC 52
opossum CTTATGGTGCCATTTATCATTTTGAATATTATTTAGACT-TGCCG-TGTAAAGCTGTAAC 58
frog CTTATGGTGCCATTTATCATTTTGAATATTATTTAGACT-TGCAG-TGTAGAGCTGTAAC 58
shark -----GGTGCCATTTGTCATTGCTAATATTATTTAGAGT-TATTG-TTAAAAGCTGTAAC 53
fugu CTGGCCCTGCCATTCATCACTGCTC-TATTATTTAGACTGGATCGCTACAGAGCCGTAAC 59
stickleback CTGGCCCTGCCATTCATCACTGCTA-TATTATTTAGACTGGATTGCTACAGAGCTGTAAC 59
medaka --GGCCCCCCCATTCATCACTGCTA-TTTTATTTAGACTGCATGGCTACAGAGCTGTAAC 57
 ***** *** * * ********* * * * * * * *

## Slide 16
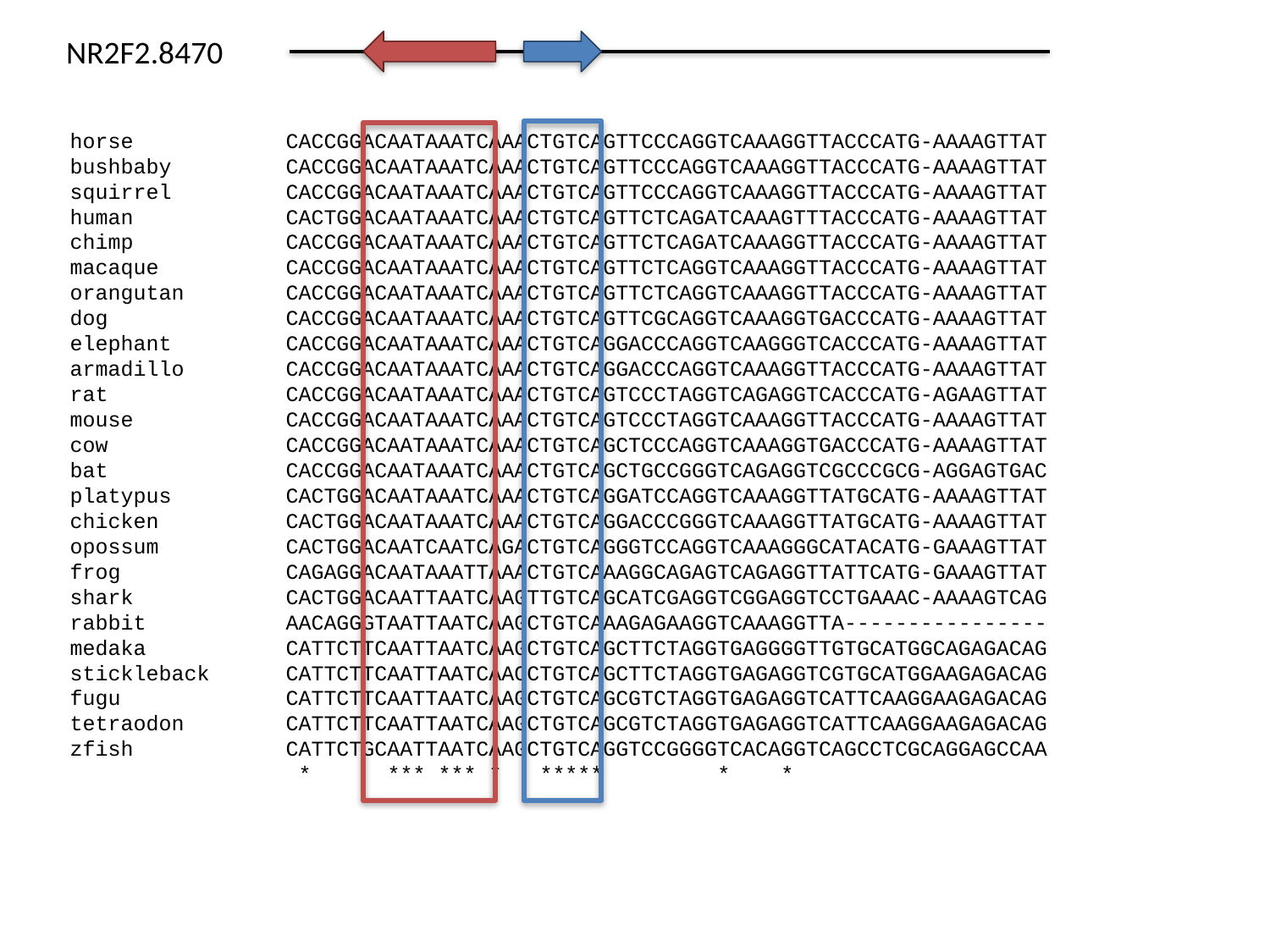

NR2F2.8470
horse CACCGGACAATAAATCAAACTGTCAGTTCCCAGGTCAAAGGTTACCCATG-AAAAGTTAT
bushbaby CACCGGACAATAAATCAAACTGTCAGTTCCCAGGTCAAAGGTTACCCATG-AAAAGTTAT
squirrel CACCGGACAATAAATCAAACTGTCAGTTCCCAGGTCAAAGGTTACCCATG-AAAAGTTAT
human CACTGGACAATAAATCAAACTGTCAGTTCTCAGATCAAAGTTTACCCATG-AAAAGTTAT
chimp CACCGGACAATAAATCAAACTGTCAGTTCTCAGATCAAAGGTTACCCATG-AAAAGTTAT
macaque CACCGGACAATAAATCAAACTGTCAGTTCTCAGGTCAAAGGTTACCCATG-AAAAGTTAT
orangutan CACCGGACAATAAATCAAACTGTCAGTTCTCAGGTCAAAGGTTACCCATG-AAAAGTTAT
dog CACCGGACAATAAATCAAACTGTCAGTTCGCAGGTCAAAGGTGACCCATG-AAAAGTTAT
elephant CACCGGACAATAAATCAAACTGTCAGGACCCAGGTCAAGGGTCACCCATG-AAAAGTTAT
armadillo CACCGGACAATAAATCAAACTGTCAGGACCCAGGTCAAAGGTTACCCATG-AAAAGTTAT
rat CACCGGACAATAAATCAAACTGTCAGTCCCTAGGTCAGAGGTCACCCATG-AGAAGTTAT
mouse CACCGGACAATAAATCAAACTGTCAGTCCCTAGGTCAAAGGTTACCCATG-AAAAGTTAT
cow CACCGGACAATAAATCAAACTGTCAGCTCCCAGGTCAAAGGTGACCCATG-AAAAGTTAT
bat CACCGGACAATAAATCAAACTGTCAGCTGCCGGGTCAGAGGTCGCCCGCG-AGGAGTGAC
platypus CACTGGACAATAAATCAAACTGTCAGGATCCAGGTCAAAGGTTATGCATG-AAAAGTTAT
chicken CACTGGACAATAAATCAAACTGTCAGGACCCGGGTCAAAGGTTATGCATG-AAAAGTTAT
opossum CACTGGACAATCAATCAGACTGTCAGGGTCCAGGTCAAAGGGCATACATG-GAAAGTTAT
frog CAGAGGACAATAAATTAAACTGTCAAAGGCAGAGTCAGAGGTTATTCATG-GAAAGTTAT
shark CACTGGACAATTAATCAAGTTGTCAGCATCGAGGTCGGAGGTCCTGAAAC-AAAAGTCAG
rabbit AACAGGGTAATTAATCAAGCTGTCAAAGAGAAGGTCAAAGGTTA----------------
medaka CATTCTTCAATTAATCAAGCTGTCAGCTTCTAGGTGAGGGGTTGTGCATGGCAGAGACAG
stickleback CATTCTTCAATTAATCAACCTGTCAGCTTCTAGGTGAGAGGTCGTGCATGGAAGAGACAG
fugu CATTCTTCAATTAATCAAGCTGTCAGCGTCTAGGTGAGAGGTCATTCAAGGAAGAGACAG
tetraodon CATTCTTCAATTAATCAAGCTGTCAGCGTCTAGGTGAGAGGTCATTCAAGGAAGAGACAG
zfish CATTCTGCAATTAATCAAGCTGTCAGGTCCGGGGTCACAGGTCAGCCTCGCAGGAGCCAA
 * *** *** * ***** * *

## Slide 17
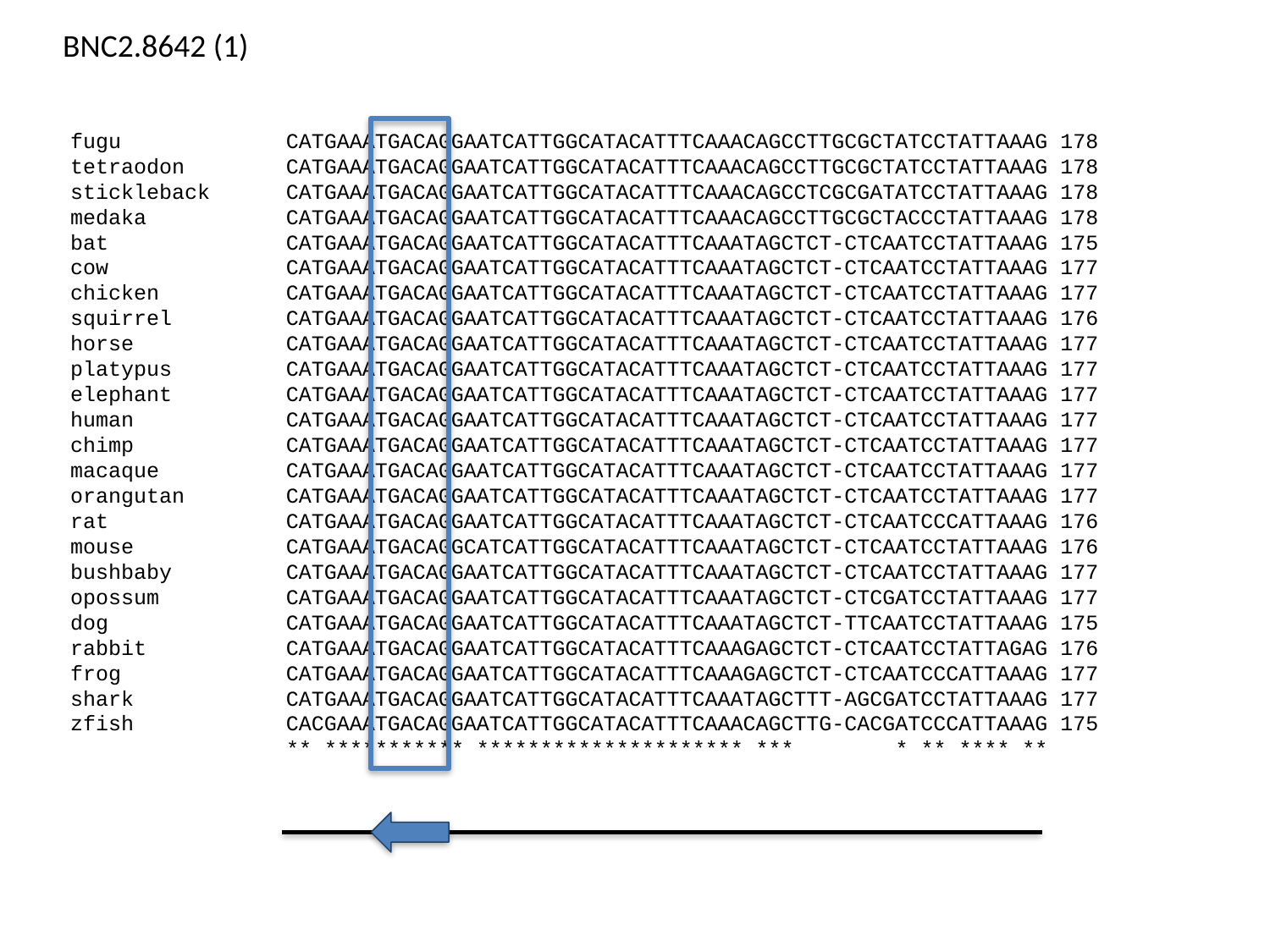

BNC2.8642 (1)
fugu CATGAAATGACAGGAATCATTGGCATACATTTCAAACAGCCTTGCGCTATCCTATTAAAG 178
tetraodon CATGAAATGACAGGAATCATTGGCATACATTTCAAACAGCCTTGCGCTATCCTATTAAAG 178
stickleback CATGAAATGACAGGAATCATTGGCATACATTTCAAACAGCCTCGCGATATCCTATTAAAG 178
medaka CATGAAATGACAGGAATCATTGGCATACATTTCAAACAGCCTTGCGCTACCCTATTAAAG 178
bat CATGAAATGACAGGAATCATTGGCATACATTTCAAATAGCTCT-CTCAATCCTATTAAAG 175
cow CATGAAATGACAGGAATCATTGGCATACATTTCAAATAGCTCT-CTCAATCCTATTAAAG 177
chicken CATGAAATGACAGGAATCATTGGCATACATTTCAAATAGCTCT-CTCAATCCTATTAAAG 177
squirrel CATGAAATGACAGGAATCATTGGCATACATTTCAAATAGCTCT-CTCAATCCTATTAAAG 176
horse CATGAAATGACAGGAATCATTGGCATACATTTCAAATAGCTCT-CTCAATCCTATTAAAG 177
platypus CATGAAATGACAGGAATCATTGGCATACATTTCAAATAGCTCT-CTCAATCCTATTAAAG 177
elephant CATGAAATGACAGGAATCATTGGCATACATTTCAAATAGCTCT-CTCAATCCTATTAAAG 177
human CATGAAATGACAGGAATCATTGGCATACATTTCAAATAGCTCT-CTCAATCCTATTAAAG 177
chimp CATGAAATGACAGGAATCATTGGCATACATTTCAAATAGCTCT-CTCAATCCTATTAAAG 177
macaque CATGAAATGACAGGAATCATTGGCATACATTTCAAATAGCTCT-CTCAATCCTATTAAAG 177
orangutan CATGAAATGACAGGAATCATTGGCATACATTTCAAATAGCTCT-CTCAATCCTATTAAAG 177
rat CATGAAATGACAGGAATCATTGGCATACATTTCAAATAGCTCT-CTCAATCCCATTAAAG 176
mouse CATGAAATGACAGGCATCATTGGCATACATTTCAAATAGCTCT-CTCAATCCTATTAAAG 176
bushbaby CATGAAATGACAGGAATCATTGGCATACATTTCAAATAGCTCT-CTCAATCCTATTAAAG 177
opossum CATGAAATGACAGGAATCATTGGCATACATTTCAAATAGCTCT-CTCGATCCTATTAAAG 177
dog CATGAAATGACAGGAATCATTGGCATACATTTCAAATAGCTCT-TTCAATCCTATTAAAG 175
rabbit CATGAAATGACAGGAATCATTGGCATACATTTCAAAGAGCTCT-CTCAATCCTATTAGAG 176
frog CATGAAATGACAGGAATCATTGGCATACATTTCAAAGAGCTCT-CTCAATCCCATTAAAG 177
shark CATGAAATGACAGGAATCATTGGCATACATTTCAAATAGCTTT-AGCGATCCTATTAAAG 177
zfish CACGAAATGACAGGAATCATTGGCATACATTTCAAACAGCTTG-CACGATCCCATTAAAG 175
 ** *********** ********************* *** * ** **** **

## Slide 18
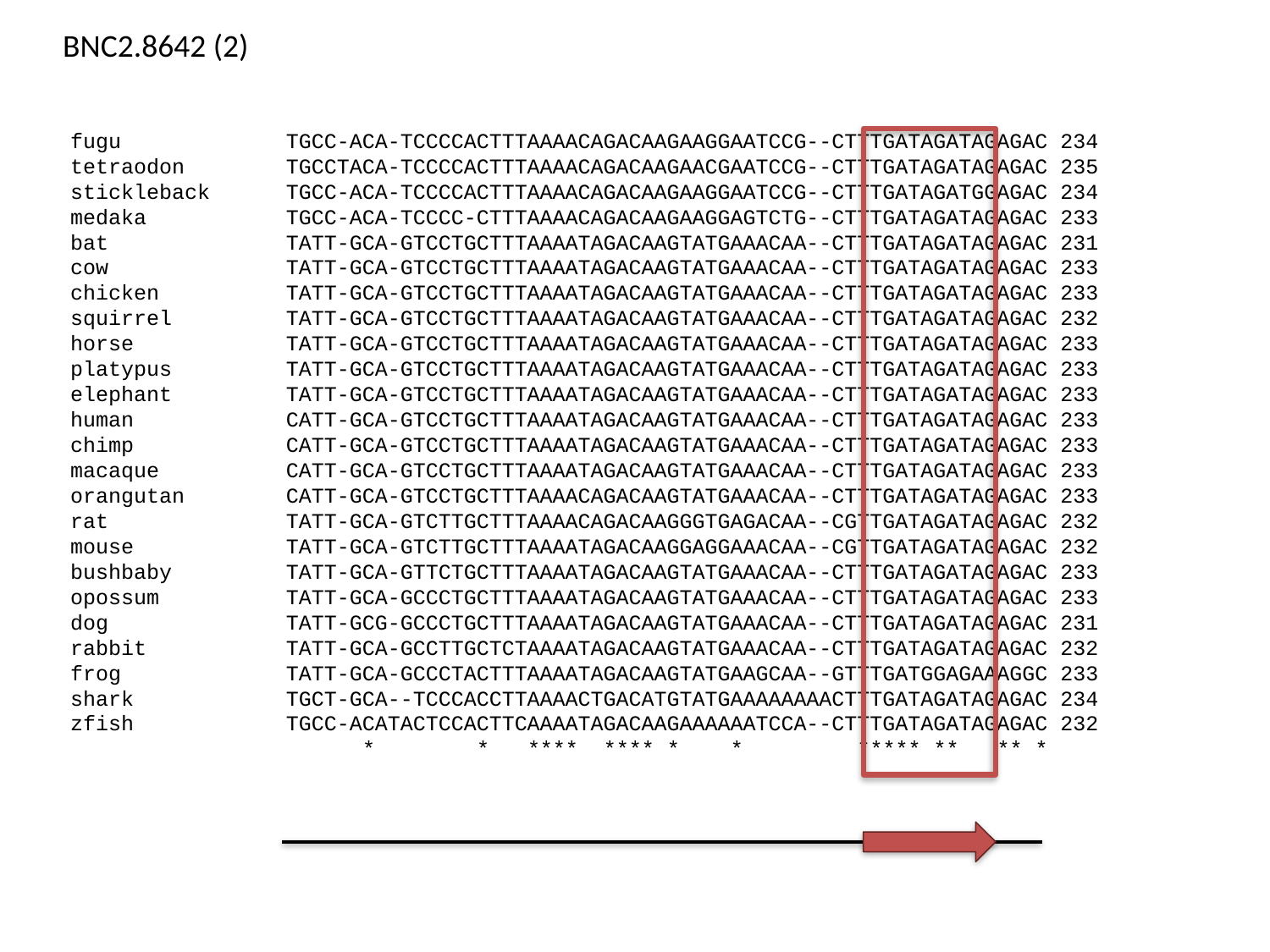

BNC2.8642 (2)
fugu TGCC-ACA-TCCCCACTTTAAAACAGACAAGAAGGAATCCG--CTTTGATAGATAGAGAC 234
tetraodon TGCCTACA-TCCCCACTTTAAAACAGACAAGAACGAATCCG--CTTTGATAGATAGAGAC 235
stickleback TGCC-ACA-TCCCCACTTTAAAACAGACAAGAAGGAATCCG--CTTTGATAGATGGAGAC 234
medaka TGCC-ACA-TCCCC-CTTTAAAACAGACAAGAAGGAGTCTG--CTTTGATAGATAGAGAC 233
bat TATT-GCA-GTCCTGCTTTAAAATAGACAAGTATGAAACAA--CTTTGATAGATAGAGAC 231
cow TATT-GCA-GTCCTGCTTTAAAATAGACAAGTATGAAACAA--CTTTGATAGATAGAGAC 233
chicken TATT-GCA-GTCCTGCTTTAAAATAGACAAGTATGAAACAA--CTTTGATAGATAGAGAC 233
squirrel TATT-GCA-GTCCTGCTTTAAAATAGACAAGTATGAAACAA--CTTTGATAGATAGAGAC 232
horse TATT-GCA-GTCCTGCTTTAAAATAGACAAGTATGAAACAA--CTTTGATAGATAGAGAC 233
platypus TATT-GCA-GTCCTGCTTTAAAATAGACAAGTATGAAACAA--CTTTGATAGATAGAGAC 233
elephant TATT-GCA-GTCCTGCTTTAAAATAGACAAGTATGAAACAA--CTTTGATAGATAGAGAC 233
human CATT-GCA-GTCCTGCTTTAAAATAGACAAGTATGAAACAA--CTTTGATAGATAGAGAC 233
chimp CATT-GCA-GTCCTGCTTTAAAATAGACAAGTATGAAACAA--CTTTGATAGATAGAGAC 233
macaque CATT-GCA-GTCCTGCTTTAAAATAGACAAGTATGAAACAA--CTTTGATAGATAGAGAC 233
orangutan CATT-GCA-GTCCTGCTTTAAAACAGACAAGTATGAAACAA--CTTTGATAGATAGAGAC 233
rat TATT-GCA-GTCTTGCTTTAAAACAGACAAGGGTGAGACAA--CGTTGATAGATAGAGAC 232
mouse TATT-GCA-GTCTTGCTTTAAAATAGACAAGGAGGAAACAA--CGTTGATAGATAGAGAC 232
bushbaby TATT-GCA-GTTCTGCTTTAAAATAGACAAGTATGAAACAA--CTTTGATAGATAGAGAC 233
opossum TATT-GCA-GCCCTGCTTTAAAATAGACAAGTATGAAACAA--CTTTGATAGATAGAGAC 233
dog TATT-GCG-GCCCTGCTTTAAAATAGACAAGTATGAAACAA--CTTTGATAGATAGAGAC 231
rabbit TATT-GCA-GCCTTGCTCTAAAATAGACAAGTATGAAACAA--CTTTGATAGATAGAGAC 232
frog TATT-GCA-GCCCTACTTTAAAATAGACAAGTATGAAGCAA--GTTTGATGGAGAAAGGC 233
shark TGCT-GCA--TCCCACCTTAAAACTGACATGTATGAAAAAAAACTTTGATAGATAGAGAC 234
zfish TGCC-ACATACTCCACTTCAAAATAGACAAGAAAAAATCCA--CTTTGATAGATAGAGAC 232
 * * **** **** * * ***** ** ** *

## Slide 19
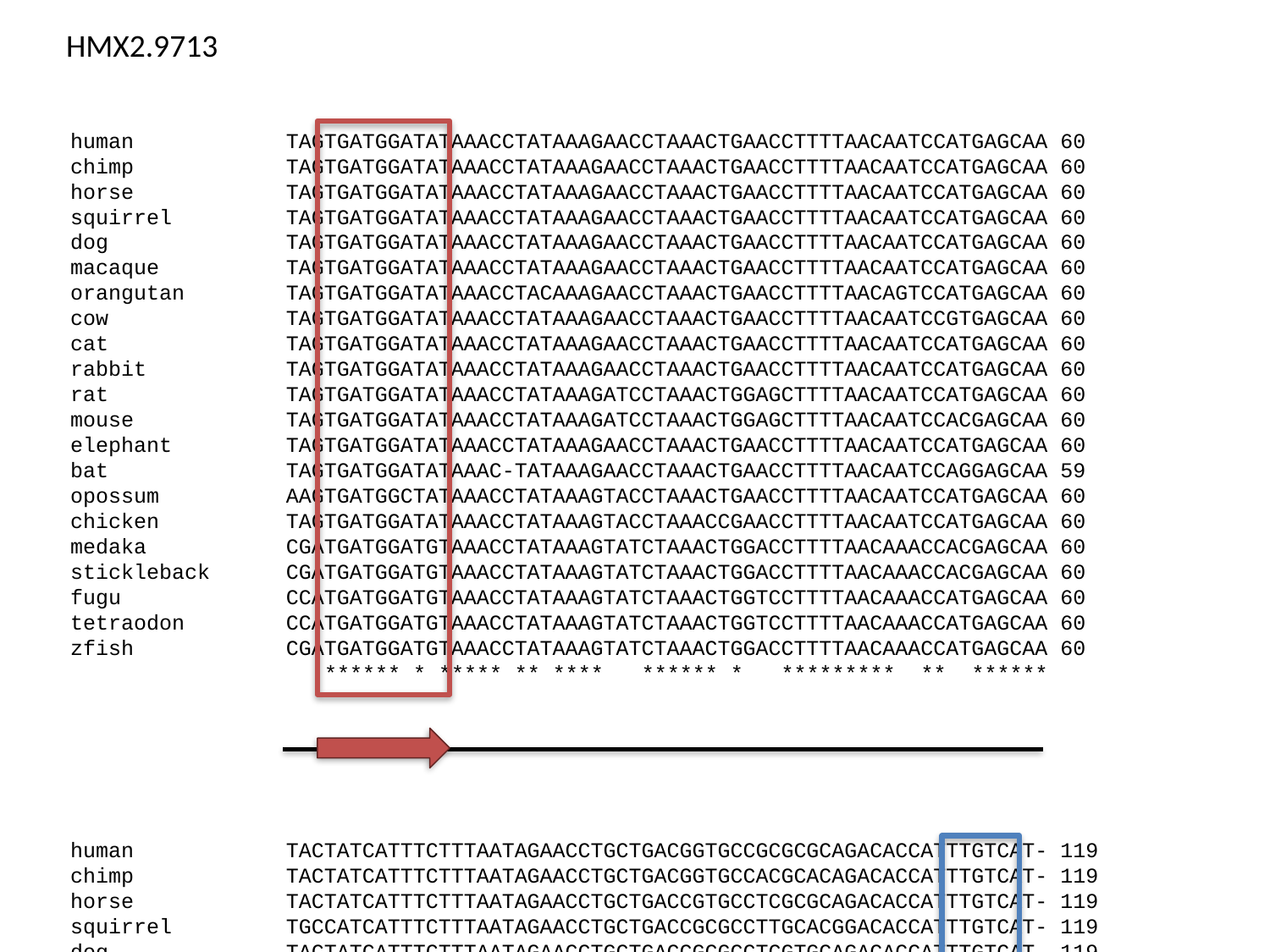

HMX2.9713
human TAGTGATGGATATAAACCTATAAAGAACCTAAACTGAACCTTTTAACAATCCATGAGCAA 60
chimp TAGTGATGGATATAAACCTATAAAGAACCTAAACTGAACCTTTTAACAATCCATGAGCAA 60
horse TAGTGATGGATATAAACCTATAAAGAACCTAAACTGAACCTTTTAACAATCCATGAGCAA 60
squirrel TAGTGATGGATATAAACCTATAAAGAACCTAAACTGAACCTTTTAACAATCCATGAGCAA 60
dog TAGTGATGGATATAAACCTATAAAGAACCTAAACTGAACCTTTTAACAATCCATGAGCAA 60
macaque TAGTGATGGATATAAACCTATAAAGAACCTAAACTGAACCTTTTAACAATCCATGAGCAA 60
orangutan TAGTGATGGATATAAACCTACAAAGAACCTAAACTGAACCTTTTAACAGTCCATGAGCAA 60
cow TAGTGATGGATATAAACCTATAAAGAACCTAAACTGAACCTTTTAACAATCCGTGAGCAA 60
cat TAGTGATGGATATAAACCTATAAAGAACCTAAACTGAACCTTTTAACAATCCATGAGCAA 60
rabbit TAGTGATGGATATAAACCTATAAAGAACCTAAACTGAACCTTTTAACAATCCATGAGCAA 60
rat TAGTGATGGATATAAACCTATAAAGATCCTAAACTGGAGCTTTTAACAATCCATGAGCAA 60
mouse TAGTGATGGATATAAACCTATAAAGATCCTAAACTGGAGCTTTTAACAATCCACGAGCAA 60
elephant TAGTGATGGATATAAACCTATAAAGAACCTAAACTGAACCTTTTAACAATCCATGAGCAA 60
bat TAGTGATGGATATAAAC-TATAAAGAACCTAAACTGAACCTTTTAACAATCCAGGAGCAA 59
opossum AAGTGATGGCTATAAACCTATAAAGTACCTAAACTGAACCTTTTAACAATCCATGAGCAA 60
chicken TAGTGATGGATATAAACCTATAAAGTACCTAAACCGAACCTTTTAACAATCCATGAGCAA 60
medaka CGATGATGGATGTAAACCTATAAAGTATCTAAACTGGACCTTTTAACAAACCACGAGCAA 60
stickleback CGATGATGGATGTAAACCTATAAAGTATCTAAACTGGACCTTTTAACAAACCACGAGCAA 60
fugu CCATGATGGATGTAAACCTATAAAGTATCTAAACTGGTCCTTTTAACAAACCATGAGCAA 60
tetraodon CCATGATGGATGTAAACCTATAAAGTATCTAAACTGGTCCTTTTAACAAACCATGAGCAA 60
zfish CGATGATGGATGTAAACCTATAAAGTATCTAAACTGGACCTTTTAACAAACCATGAGCAA 60
 ****** * ***** ** **** ****** * ********* ** ******
human TACTATCATTTCTTTAATAGAACCTGCTGACGGTGCCGCGCGCAGACACCATTTGTCAT- 119
chimp TACTATCATTTCTTTAATAGAACCTGCTGACGGTGCCACGCACAGACACCATTTGTCAT- 119
horse TACTATCATTTCTTTAATAGAACCTGCTGACCGTGCCTCGCGCAGACACCATTTGTCAT- 119
squirrel TGCCATCATTTCTTTAATAGAACCTGCTGACCGCGCCTTGCACGGACACCATTTGTCAT- 119
dog TACTATCATTTCTTTAATAGAACCTGCTGACCGCGCCTCGTGCAGACACCATTTGTCAT- 119
macaque TACTATCATTTCTTTAATAGAACCTGCTGACCGTGCCGTGCACAGGCACCATTTGTCAT- 119
orangutan TACTATCATTTCTTTAATAGAACCTGCTGACCGTGCGGTGTGCAGACACCATTTGTCAT- 119
cow CACTATCATTTCTTTAATAGAACCTGCTGACCGTGCCTTGCACAGACACCATTTGTCAC- 119
cat TACTATCATTTCTTTAATAGAACCTGCTGACCGTGCCTCGCACAGATGCTATTTGTCAT- 119
rabbit TACTATCATTTCTTTAATAGAACCTGCTGACCGTGCCTTGCACAGCCGCCATTTGTCAT- 119
rat TACTATCATTTCTTTAATAGAACCTGCTGACCGTGCCTTGCACAGACACTATTTGTCAT- 119
mouse TGCTATCATTTCTTTAATAGAACCTGCTGACCGTGCCTTGCACAGACACTATTTGTCAT- 119
elephant TACTATCATTTCTTTAATAGAACCTGCTGACCGTGTCTTGCACAGACATCATTTGTCAT- 119
bat TACTATCATTTCTTTAATGGAACCCGCTGACCGTGCCCCGCGCAGACGCCATCTGTCAT- 118
opossum TCCTATCATTTCTTTAATAGAACTCGCTGACCAGGCCTTTCTCCAATGCCGTTTGTCAT- 119
chicken TACTATCATTTCTTTAATAGAACTCACTGGCCGTGCCTTTCTCAGATGCCATTTGTCAT- 119
medaka CACTATCATTTCTCTAATAGAACCTGCTGACCGTGTCATTCCCTCCTGCCATTTGTCATT 120
stickleback CACTATCATTTCTCTAATAGAACCTGCTGACCGTGTCATTCCCTCCTGCCATTTGTCATT 120
fugu CACTATCATTTCTCTAATAGAACCTGCTGACCGTGTCATTCCCCGCTGCCATTCGTCATC 120
tetraodon CACTATCATTTCTCTAATAGAACCTGCTGACCGTGTCATTCCCCGCTGCCATTCGTCATC 120
zfish TACTATCATTTCTTTAATAGAACCTGCTGACCGTGTCATTCCGTTCTGCCATTTGTCAT- 119
 * ********* **** **** *** * * * ****

## Slide 20
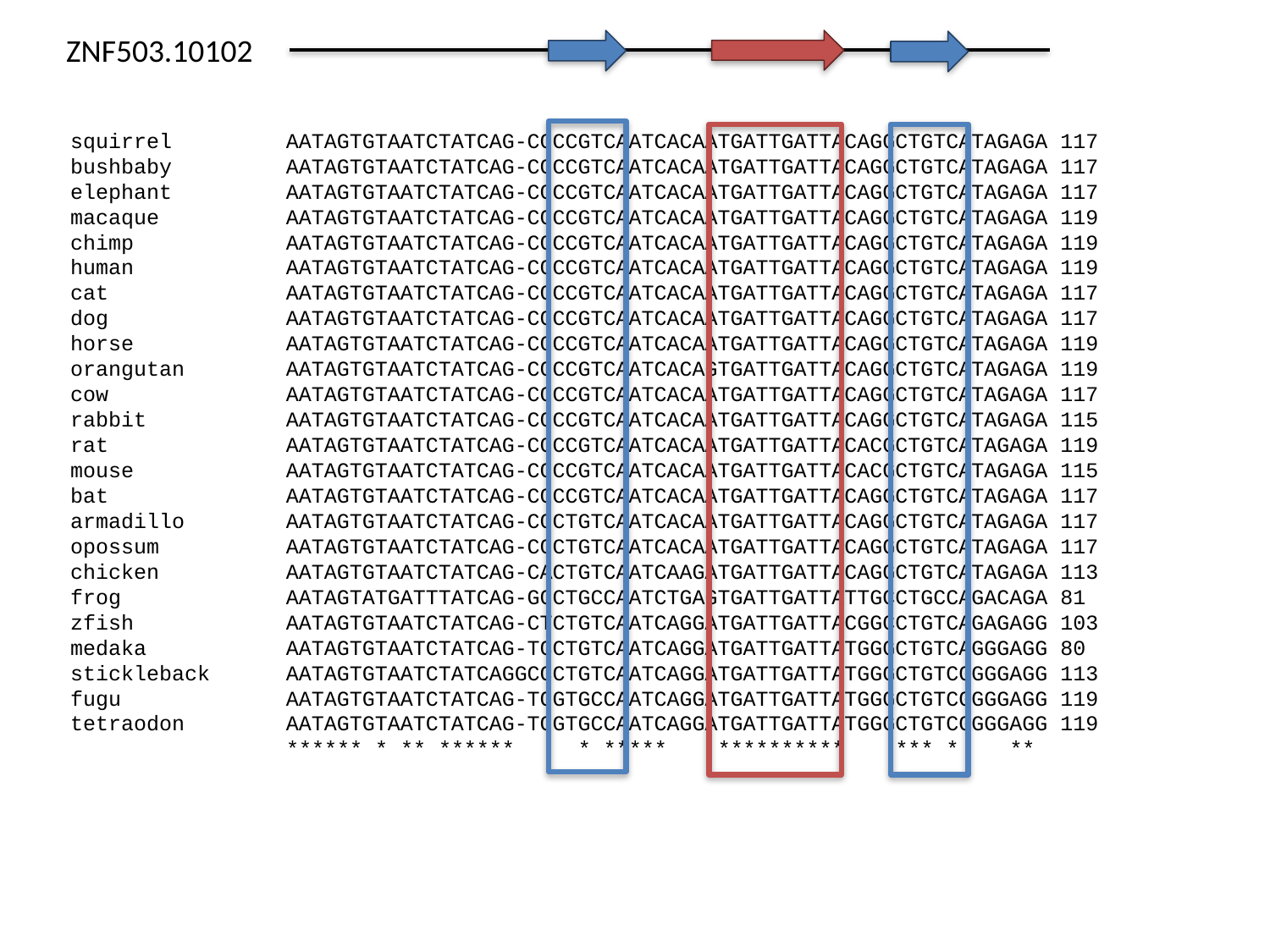

ZNF503.10102
squirrel AATAGTGTAATCTATCAG-CGCCGTCAATCACAATGATTGATTACAGGCTGTCATAGAGA 117
bushbaby AATAGTGTAATCTATCAG-CGCCGTCAATCACAATGATTGATTACAGGCTGTCATAGAGA 117
elephant AATAGTGTAATCTATCAG-CGCCGTCAATCACAATGATTGATTACAGGCTGTCATAGAGA 117
macaque AATAGTGTAATCTATCAG-CGCCGTCAATCACAATGATTGATTACAGGCTGTCATAGAGA 119
chimp AATAGTGTAATCTATCAG-CGCCGTCAATCACAATGATTGATTACAGGCTGTCATAGAGA 119
human AATAGTGTAATCTATCAG-CGCCGTCAATCACAATGATTGATTACAGGCTGTCATAGAGA 119
cat AATAGTGTAATCTATCAG-CGCCGTCAATCACAATGATTGATTACAGGCTGTCATAGAGA 117
dog AATAGTGTAATCTATCAG-CGCCGTCAATCACAATGATTGATTACAGGCTGTCATAGAGA 117
horse AATAGTGTAATCTATCAG-CGCCGTCAATCACAATGATTGATTACAGGCTGTCATAGAGA 119
orangutan AATAGTGTAATCTATCAG-CGCCGTCAATCACAGTGATTGATTACAGGCTGTCATAGAGA 119
cow AATAGTGTAATCTATCAG-CGCCGTCAATCACAATGATTGATTACAGGCTGTCATAGAGA 117
rabbit AATAGTGTAATCTATCAG-CGCCGTCAATCACAATGATTGATTACAGGCTGTCATAGAGA 115
rat AATAGTGTAATCTATCAG-CGCCGTCAATCACAATGATTGATTACACGCTGTCATAGAGA 119
mouse AATAGTGTAATCTATCAG-CGCCGTCAATCACAATGATTGATTACACGCTGTCATAGAGA 115
bat AATAGTGTAATCTATCAG-CGCCGTCAATCACAATGATTGATTACAGGCTGTCATAGAGA 117
armadillo AATAGTGTAATCTATCAG-CGCTGTCAATCACAATGATTGATTACAGGCTGTCATAGAGA 117
opossum AATAGTGTAATCTATCAG-CGCTGTCAATCACAATGATTGATTACAGGCTGTCATAGAGA 117
chicken AATAGTGTAATCTATCAG-CACTGTCAATCAAGATGATTGATTACAGGCTGTCATAGAGA 113
frog AATAGTATGATTTATCAG-GGCTGCCAATCTGAGTGATTGATTATTGCCTGCCAGACAGA 81
zfish AATAGTGTAATCTATCAG-CTCTGTCAATCAGGATGATTGATTACGGCCTGTCAGAGAGG 103
medaka AATAGTGTAATCTATCAG-TCCTGTCAATCAGGATGATTGATTATGGGCTGTCAGGGAGG 80
stickleback AATAGTGTAATCTATCAGGCCCTGTCAATCAGGATGATTGATTATGGGCTGTCGGGGAGG 113
fugu AATAGTGTAATCTATCAG-TGGTGCCAATCAGGATGATTGATTATGGGCTGTCGGGGAGG 119
tetraodon AATAGTGTAATCTATCAG-TGGTGCCAATCAGGATGATTGATTATGGGCTGTCGGGGAGG 119
 ****** * ** ****** * ***** ********** *** * **

## Slide 21
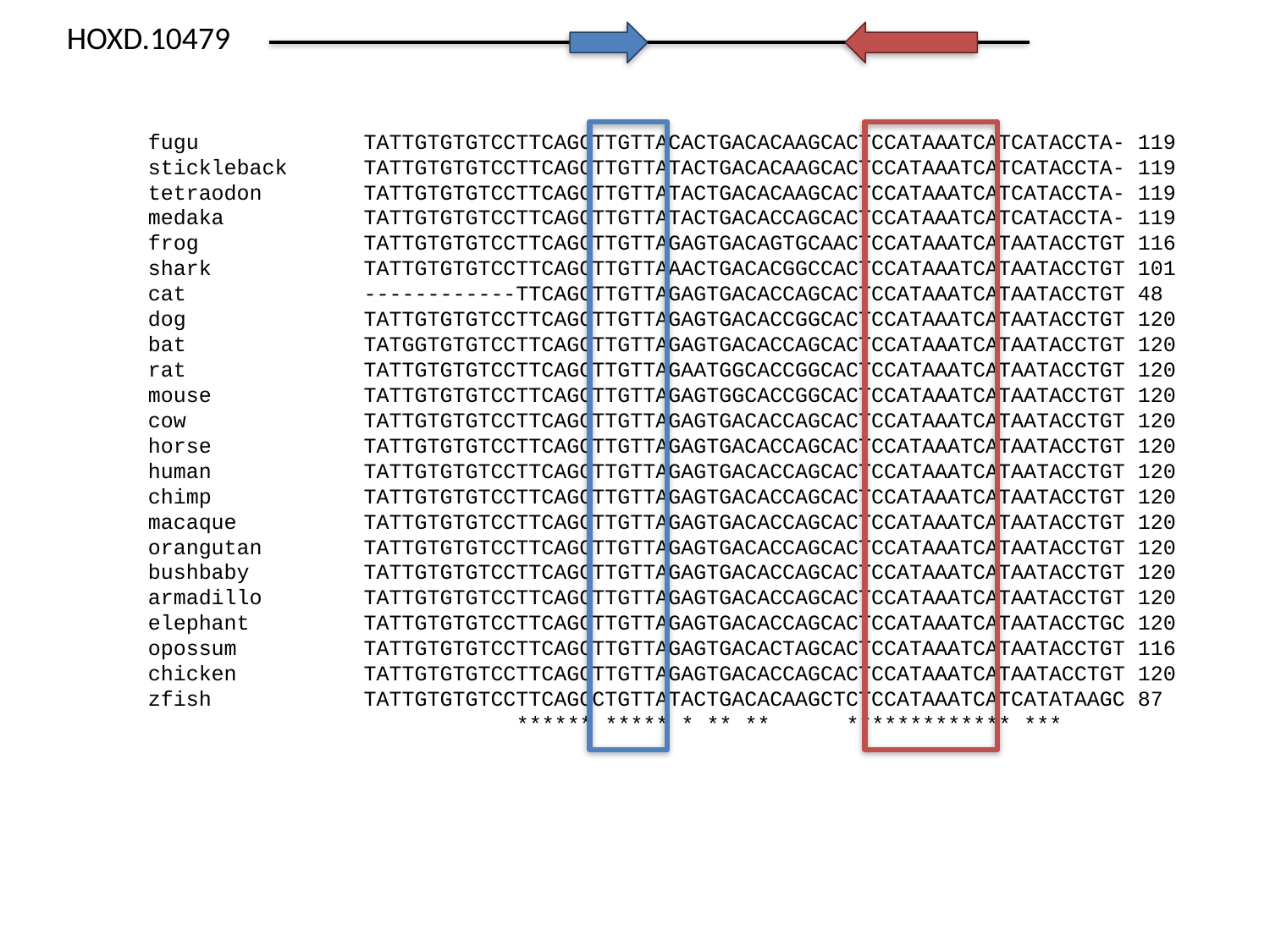

HOXD.10479
fugu TATTGTGTGTCCTTCAGCTTGTTACACTGACACAAGCACTCCATAAATCATCATACCTA- 119
stickleback TATTGTGTGTCCTTCAGCTTGTTATACTGACACAAGCACTCCATAAATCATCATACCTA- 119
tetraodon TATTGTGTGTCCTTCAGCTTGTTATACTGACACAAGCACTCCATAAATCATCATACCTA- 119
medaka TATTGTGTGTCCTTCAGCTTGTTATACTGACACCAGCACTCCATAAATCATCATACCTA- 119
frog TATTGTGTGTCCTTCAGCTTGTTAGAGTGACAGTGCAACTCCATAAATCATAATACCTGT 116
shark TATTGTGTGTCCTTCAGCTTGTTAAACTGACACGGCCACTCCATAAATCATAATACCTGT 101
cat ------------TTCAGCTTGTTAGAGTGACACCAGCACTCCATAAATCATAATACCTGT 48
dog TATTGTGTGTCCTTCAGCTTGTTAGAGTGACACCGGCACTCCATAAATCATAATACCTGT 120
bat TATGGTGTGTCCTTCAGCTTGTTAGAGTGACACCAGCACTCCATAAATCATAATACCTGT 120
rat TATTGTGTGTCCTTCAGCTTGTTAGAATGGCACCGGCACTCCATAAATCATAATACCTGT 120
mouse TATTGTGTGTCCTTCAGCTTGTTAGAGTGGCACCGGCACTCCATAAATCATAATACCTGT 120
cow TATTGTGTGTCCTTCAGCTTGTTAGAGTGACACCAGCACTCCATAAATCATAATACCTGT 120
horse TATTGTGTGTCCTTCAGCTTGTTAGAGTGACACCAGCACTCCATAAATCATAATACCTGT 120
human TATTGTGTGTCCTTCAGCTTGTTAGAGTGACACCAGCACTCCATAAATCATAATACCTGT 120
chimp TATTGTGTGTCCTTCAGCTTGTTAGAGTGACACCAGCACTCCATAAATCATAATACCTGT 120
macaque TATTGTGTGTCCTTCAGCTTGTTAGAGTGACACCAGCACTCCATAAATCATAATACCTGT 120
orangutan TATTGTGTGTCCTTCAGCTTGTTAGAGTGACACCAGCACTCCATAAATCATAATACCTGT 120
bushbaby TATTGTGTGTCCTTCAGCTTGTTAGAGTGACACCAGCACTCCATAAATCATAATACCTGT 120
armadillo TATTGTGTGTCCTTCAGCTTGTTAGAGTGACACCAGCACTCCATAAATCATAATACCTGT 120
elephant TATTGTGTGTCCTTCAGCTTGTTAGAGTGACACCAGCACTCCATAAATCATAATACCTGC 120
opossum TATTGTGTGTCCTTCAGCTTGTTAGAGTGACACTAGCACTCCATAAATCATAATACCTGT 116
chicken TATTGTGTGTCCTTCAGCTTGTTAGAGTGACACCAGCACTCCATAAATCATAATACCTGT 120
zfish TATTGTGTGTCCTTCAGCCTGTTATACTGACACAAGCTCTCCATAAATCATCATATAAGC 87
 ****** ***** * ** ** ************* ***

## Slide 22
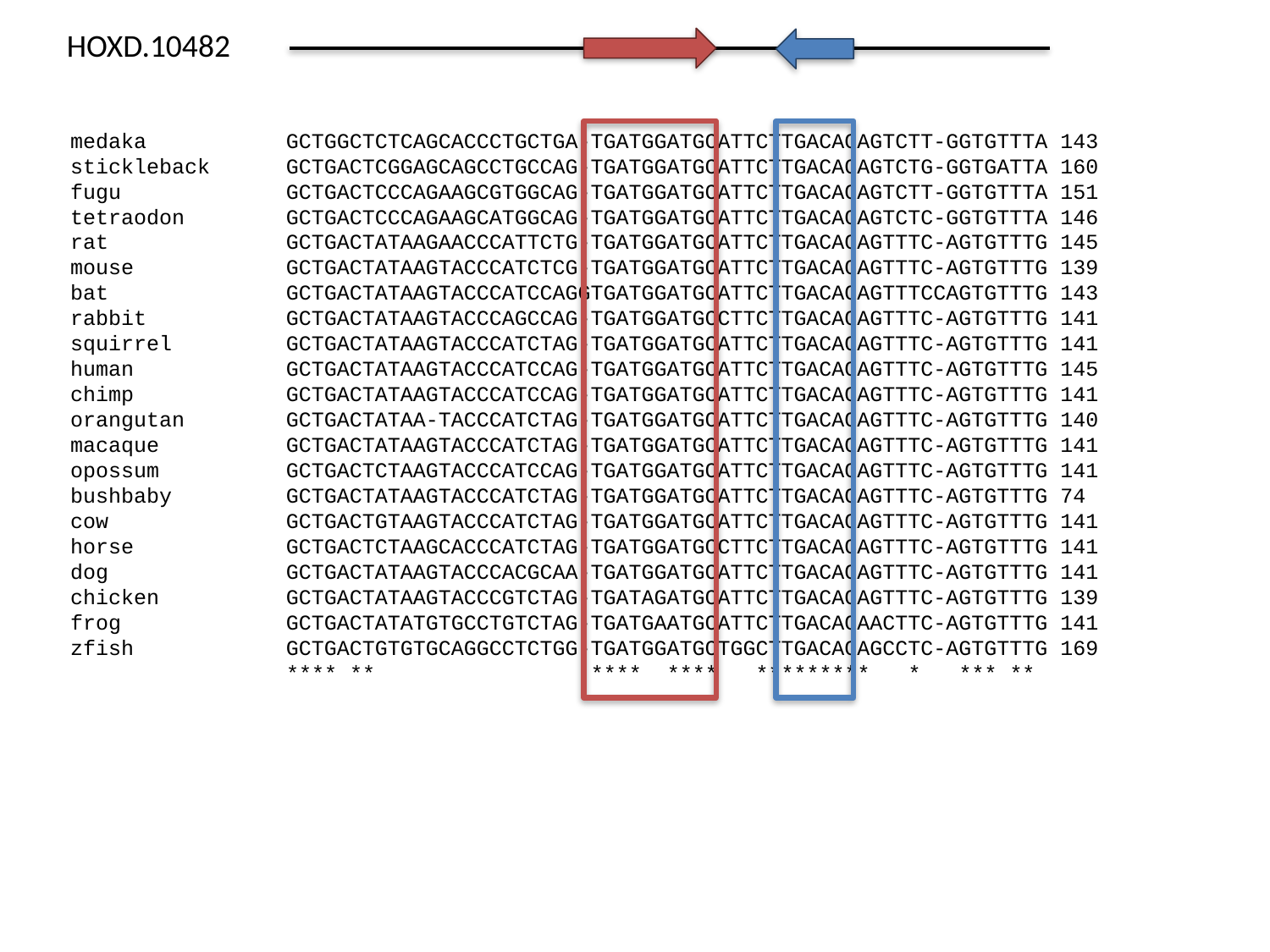

HOXD.10482
medaka GCTGGCTCTCAGCACCCTGCTGA-TGATGGATGCATTCTTGACACAGTCTT-GGTGTTTA 143
stickleback GCTGACTCGGAGCAGCCTGCCAG-TGATGGATGCATTCTTGACACAGTCTG-GGTGATTA 160
fugu GCTGACTCCCAGAAGCGTGGCAG-TGATGGATGCATTCTTGACACAGTCTT-GGTGTTTA 151
tetraodon GCTGACTCCCAGAAGCATGGCAG-TGATGGATGCATTCTTGACACAGTCTC-GGTGTTTA 146
rat GCTGACTATAAGAACCCATTCTG-TGATGGATGCATTCTTGACACAGTTTC-AGTGTTTG 145
mouse GCTGACTATAAGTACCCATCTCG-TGATGGATGCATTCTTGACACAGTTTC-AGTGTTTG 139
bat GCTGACTATAAGTACCCATCCAGGTGATGGATGCATTCTTGACACAGTTTCCAGTGTTTG 143
rabbit GCTGACTATAAGTACCCAGCCAG-TGATGGATGCCTTCTTGACACAGTTTC-AGTGTTTG 141
squirrel GCTGACTATAAGTACCCATCTAG-TGATGGATGCATTCTTGACACAGTTTC-AGTGTTTG 141
human GCTGACTATAAGTACCCATCCAG-TGATGGATGCATTCTTGACACAGTTTC-AGTGTTTG 145
chimp GCTGACTATAAGTACCCATCCAG-TGATGGATGCATTCTTGACACAGTTTC-AGTGTTTG 141
orangutan GCTGACTATAA-TACCCATCTAG-TGATGGATGCATTCTTGACACAGTTTC-AGTGTTTG 140
macaque GCTGACTATAAGTACCCATCTAG-TGATGGATGCATTCTTGACACAGTTTC-AGTGTTTG 141
opossum GCTGACTCTAAGTACCCATCCAG-TGATGGATGCATTCTTGACACAGTTTC-AGTGTTTG 141
bushbaby GCTGACTATAAGTACCCATCTAG-TGATGGATGCATTCTTGACACAGTTTC-AGTGTTTG 74
cow GCTGACTGTAAGTACCCATCTAG-TGATGGATGCATTCTTGACACAGTTTC-AGTGTTTG 141
horse GCTGACTCTAAGCACCCATCTAG-TGATGGATGCCTTCTTGACACAGTTTC-AGTGTTTG 141
dog GCTGACTATAAGTACCCACGCAA-TGATGGATGCATTCTTGACACAGTTTC-AGTGTTTG 141
chicken GCTGACTATAAGTACCCGTCTAG-TGATAGATGCATTCTTGACACAGTTTC-AGTGTTTG 139
frog GCTGACTATATGTGCCTGTCTAG-TGATGAATGCATTCTTGACACAACTTC-AGTGTTTG 141
zfish GCTGACTGTGTGCAGGCCTCTGG-TGATGGATGCTGGCTTGACACAGCCTC-AGTGTTTG 169
 **** ** **** **** ********* * *** **

## Slide 23
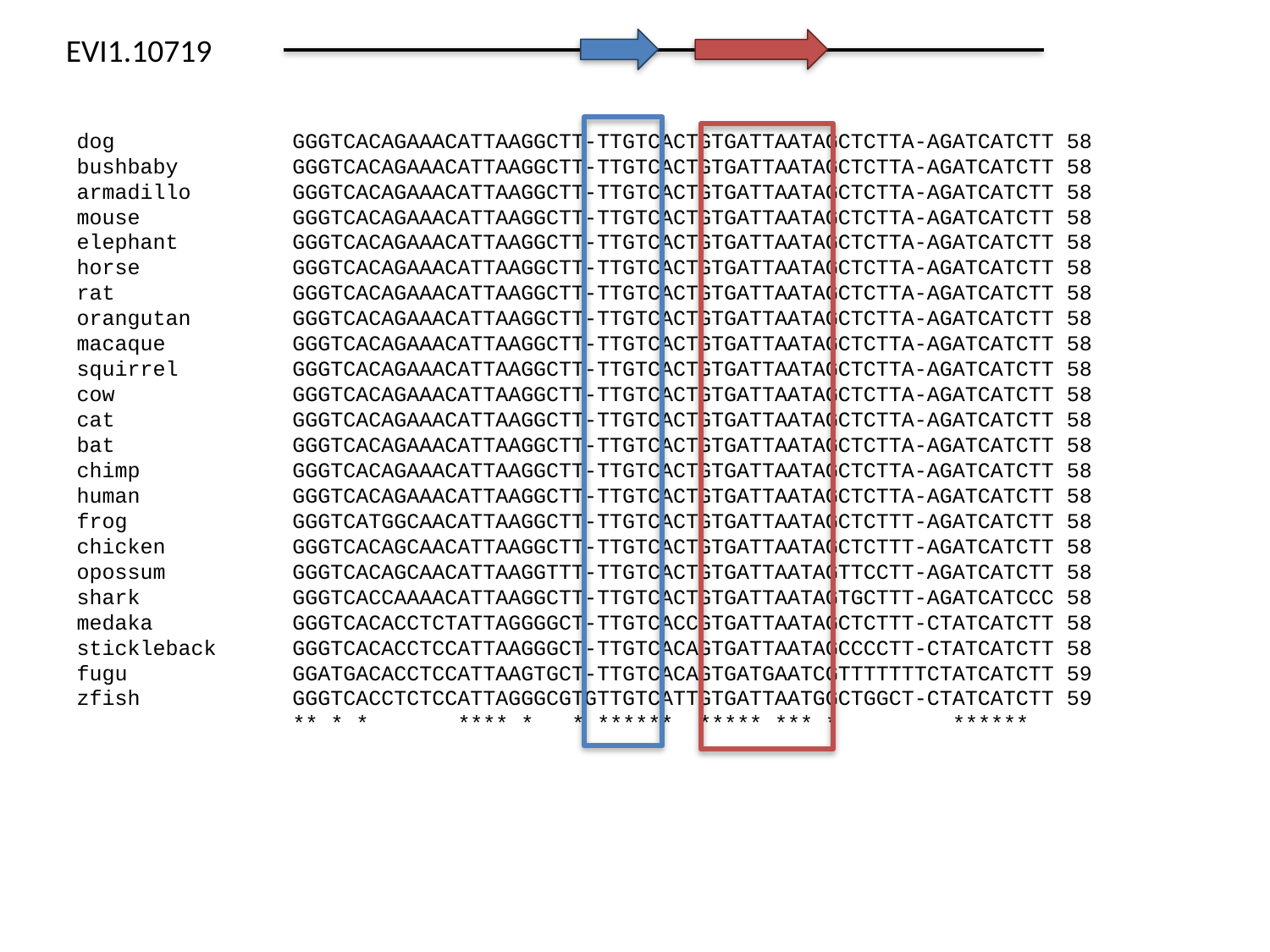

EVI1.10719
dog GGGTCACAGAAACATTAAGGCTT-TTGTCACTGTGATTAATAGCTCTTA-AGATCATCTT 58
bushbaby GGGTCACAGAAACATTAAGGCTT-TTGTCACTGTGATTAATAGCTCTTA-AGATCATCTT 58
armadillo GGGTCACAGAAACATTAAGGCTT-TTGTCACTGTGATTAATAGCTCTTA-AGATCATCTT 58
mouse GGGTCACAGAAACATTAAGGCTT-TTGTCACTGTGATTAATAGCTCTTA-AGATCATCTT 58
elephant GGGTCACAGAAACATTAAGGCTT-TTGTCACTGTGATTAATAGCTCTTA-AGATCATCTT 58
horse GGGTCACAGAAACATTAAGGCTT-TTGTCACTGTGATTAATAGCTCTTA-AGATCATCTT 58
rat GGGTCACAGAAACATTAAGGCTT-TTGTCACTGTGATTAATAGCTCTTA-AGATCATCTT 58
orangutan GGGTCACAGAAACATTAAGGCTT-TTGTCACTGTGATTAATAGCTCTTA-AGATCATCTT 58
macaque GGGTCACAGAAACATTAAGGCTT-TTGTCACTGTGATTAATAGCTCTTA-AGATCATCTT 58
squirrel GGGTCACAGAAACATTAAGGCTT-TTGTCACTGTGATTAATAGCTCTTA-AGATCATCTT 58
cow GGGTCACAGAAACATTAAGGCTT-TTGTCACTGTGATTAATAGCTCTTA-AGATCATCTT 58
cat GGGTCACAGAAACATTAAGGCTT-TTGTCACTGTGATTAATAGCTCTTA-AGATCATCTT 58
bat GGGTCACAGAAACATTAAGGCTT-TTGTCACTGTGATTAATAGCTCTTA-AGATCATCTT 58
chimp GGGTCACAGAAACATTAAGGCTT-TTGTCACTGTGATTAATAGCTCTTA-AGATCATCTT 58
human GGGTCACAGAAACATTAAGGCTT-TTGTCACTGTGATTAATAGCTCTTA-AGATCATCTT 58
frog GGGTCATGGCAACATTAAGGCTT-TTGTCACTGTGATTAATAGCTCTTT-AGATCATCTT 58
chicken GGGTCACAGCAACATTAAGGCTT-TTGTCACTGTGATTAATAGCTCTTT-AGATCATCTT 58
opossum GGGTCACAGCAACATTAAGGTTT-TTGTCACTGTGATTAATAGTTCCTT-AGATCATCTT 58
shark GGGTCACCAAAACATTAAGGCTT-TTGTCACTGTGATTAATAGTGCTTT-AGATCATCCC 58
medaka GGGTCACACCTCTATTAGGGGCT-TTGTCACCGTGATTAATAGCTCTTT-CTATCATCTT 58
stickleback GGGTCACACCTCCATTAAGGGCT-TTGTCACAGTGATTAATAGCCCCTT-CTATCATCTT 58
fugu GGATGACACCTCCATTAAGTGCT-TTGTCACAGTGATGAATCGTTTTTTTCTATCATCTT 59
zfish GGGTCACCTCTCCATTAGGGCGTGTTGTCATTGTGATTAATGGCTGGCT-CTATCATCTT 59
 ** * * **** * * ****** ***** *** * ******

## Slide 24
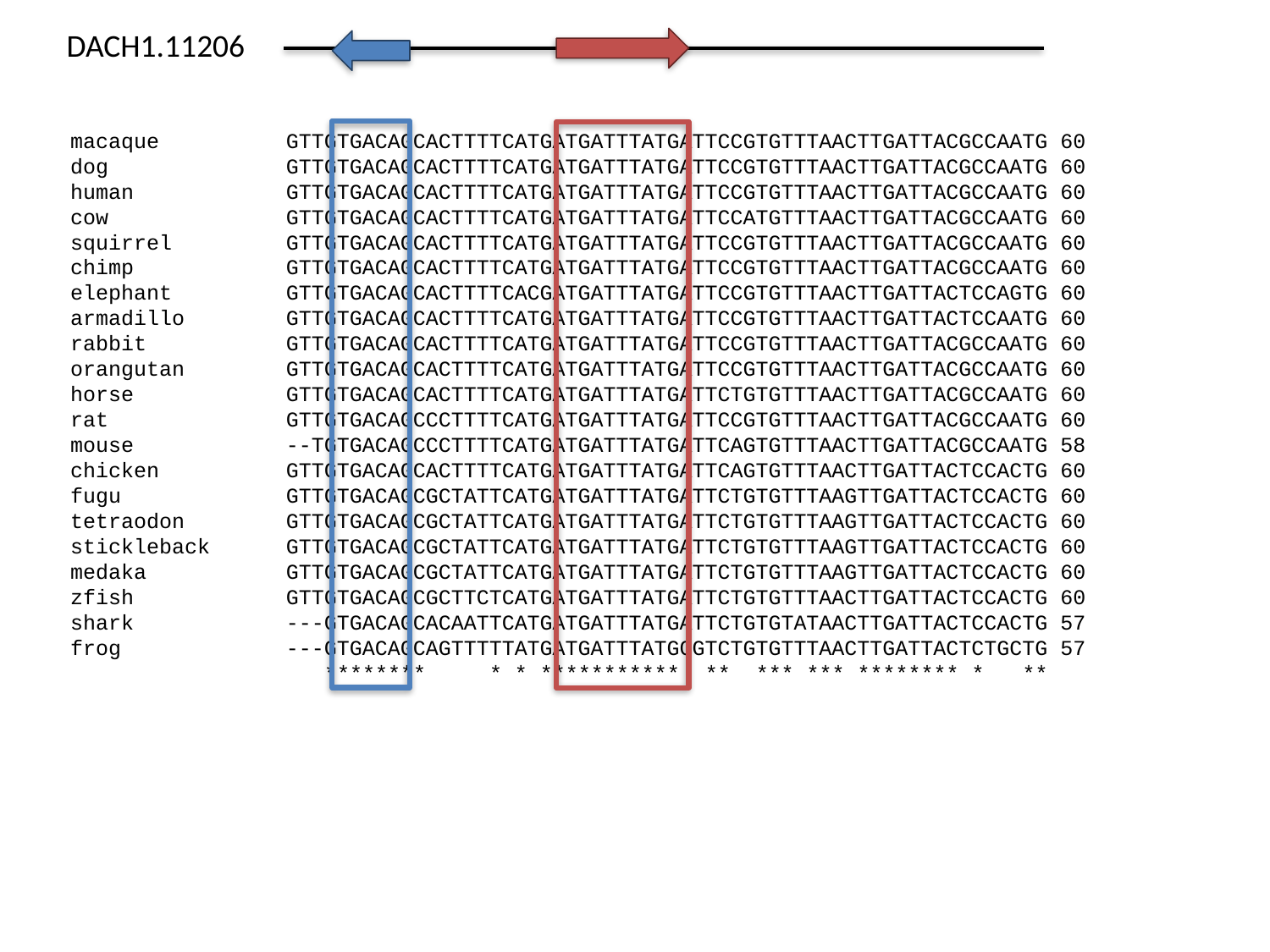

DACH1.11206
macaque GTTGTGACAGCACTTTTCATGATGATTTATGATTCCGTGTTTAACTTGATTACGCCAATG 60
dog GTTGTGACAGCACTTTTCATGATGATTTATGATTCCGTGTTTAACTTGATTACGCCAATG 60
human GTTGTGACAGCACTTTTCATGATGATTTATGATTCCGTGTTTAACTTGATTACGCCAATG 60
cow GTTGTGACAGCACTTTTCATGATGATTTATGATTCCATGTTTAACTTGATTACGCCAATG 60
squirrel GTTGTGACAGCACTTTTCATGATGATTTATGATTCCGTGTTTAACTTGATTACGCCAATG 60
chimp GTTGTGACAGCACTTTTCATGATGATTTATGATTCCGTGTTTAACTTGATTACGCCAATG 60
elephant GTTGTGACAGCACTTTTCACGATGATTTATGATTCCGTGTTTAACTTGATTACTCCAGTG 60
armadillo GTTGTGACAGCACTTTTCATGATGATTTATGATTCCGTGTTTAACTTGATTACTCCAATG 60
rabbit GTTGTGACAGCACTTTTCATGATGATTTATGATTCCGTGTTTAACTTGATTACGCCAATG 60
orangutan GTTGTGACAGCACTTTTCATGATGATTTATGATTCCGTGTTTAACTTGATTACGCCAATG 60
horse GTTGTGACAGCACTTTTCATGATGATTTATGATTCTGTGTTTAACTTGATTACGCCAATG 60
rat GTTGTGACAGCCCTTTTCATGATGATTTATGATTCCGTGTTTAACTTGATTACGCCAATG 60
mouse --TGTGACAGCCCTTTTCATGATGATTTATGATTCAGTGTTTAACTTGATTACGCCAATG 58
chicken GTTGTGACAGCACTTTTCATGATGATTTATGATTCAGTGTTTAACTTGATTACTCCACTG 60
fugu GTTGTGACAGCGCTATTCATGATGATTTATGATTCTGTGTTTAAGTTGATTACTCCACTG 60
tetraodon GTTGTGACAGCGCTATTCATGATGATTTATGATTCTGTGTTTAAGTTGATTACTCCACTG 60
stickleback GTTGTGACAGCGCTATTCATGATGATTTATGATTCTGTGTTTAAGTTGATTACTCCACTG 60
medaka GTTGTGACAGCGCTATTCATGATGATTTATGATTCTGTGTTTAAGTTGATTACTCCACTG 60
zfish GTTGTGACAGCGCTTCTCATGATGATTTATGATTCTGTGTTTAACTTGATTACTCCACTG 60
shark ---GTGACAGCACAATTCATGATGATTTATGATTCTGTGTATAACTTGATTACTCCACTG 57
frog ---GTGACAGCAGTTTTTATGATGATTTATGGGTCTGTGTTTAACTTGATTACTCTGCTG 57
 ******** * * *********** ** *** *** ******** * **

## Slide 25
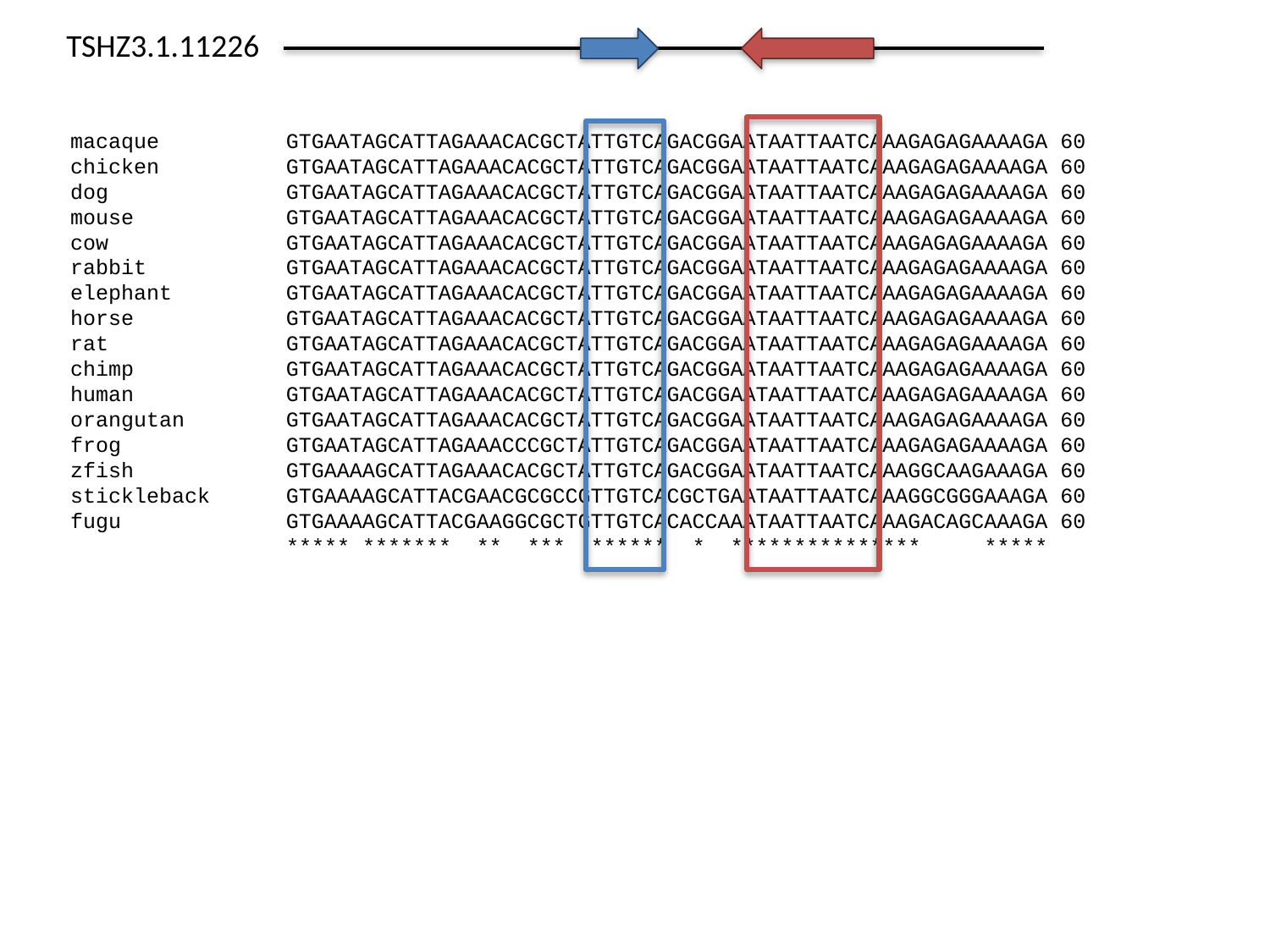

TSHZ3.1.11226
macaque GTGAATAGCATTAGAAACACGCTATTGTCAGACGGAATAATTAATCAAAGAGAGAAAAGA 60
chicken GTGAATAGCATTAGAAACACGCTATTGTCAGACGGAATAATTAATCAAAGAGAGAAAAGA 60
dog GTGAATAGCATTAGAAACACGCTATTGTCAGACGGAATAATTAATCAAAGAGAGAAAAGA 60
mouse GTGAATAGCATTAGAAACACGCTATTGTCAGACGGAATAATTAATCAAAGAGAGAAAAGA 60
cow GTGAATAGCATTAGAAACACGCTATTGTCAGACGGAATAATTAATCAAAGAGAGAAAAGA 60
rabbit GTGAATAGCATTAGAAACACGCTATTGTCAGACGGAATAATTAATCAAAGAGAGAAAAGA 60
elephant GTGAATAGCATTAGAAACACGCTATTGTCAGACGGAATAATTAATCAAAGAGAGAAAAGA 60
horse GTGAATAGCATTAGAAACACGCTATTGTCAGACGGAATAATTAATCAAAGAGAGAAAAGA 60
rat GTGAATAGCATTAGAAACACGCTATTGTCAGACGGAATAATTAATCAAAGAGAGAAAAGA 60
chimp GTGAATAGCATTAGAAACACGCTATTGTCAGACGGAATAATTAATCAAAGAGAGAAAAGA 60
human GTGAATAGCATTAGAAACACGCTATTGTCAGACGGAATAATTAATCAAAGAGAGAAAAGA 60
orangutan GTGAATAGCATTAGAAACACGCTATTGTCAGACGGAATAATTAATCAAAGAGAGAAAAGA 60
frog GTGAATAGCATTAGAAACCCGCTATTGTCAGACGGAATAATTAATCAAAGAGAGAAAAGA 60
zfish GTGAAAAGCATTAGAAACACGCTATTGTCAGACGGAATAATTAATCAAAGGCAAGAAAGA 60
stickleback GTGAAAAGCATTACGAACGCGCCGTTGTCACGCTGAATAATTAATCAAAGGCGGGAAAGA 60
fugu GTGAAAAGCATTACGAAGGCGCTGTTGTCACACCAAATAATTAATCAAAGACAGCAAAGA 60
 ***** ******* ** *** ****** * *************** *****

## Slide 26
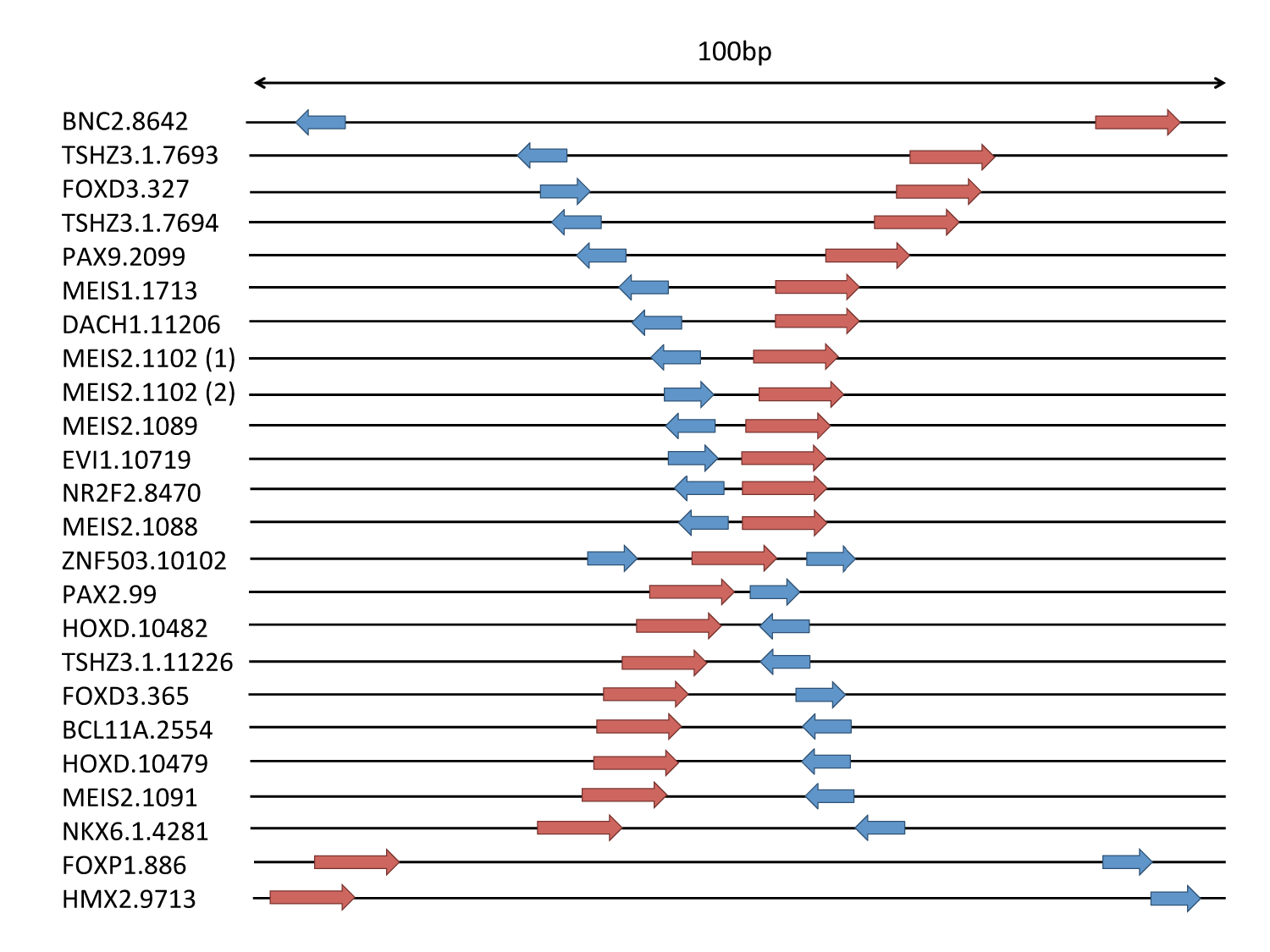

Supplement: S1 File — Clustalw2 alignments of all the hb+ elements, showing the conservation and distribution of PBX-HOX and MEIS/PREP motifs. (PPTX) [file pone.0130413.s004.pptx]
