# Supplementary material for: A Simple Predictive Enhancer Syntax for Hindbrain Patterning Is Conserved in Vertebrate Genomes
Source: PLoS One. 2015 Jul 1;10(7):e0130413. doi: 10.1371/journal.pone.0130413 (PMC4489388; doi:10.1371/journal.pone.0130413)
Supplement: S2 File — Clustalw2 alignments of all the hb+ candidates as determined by FIMO, showing the conservation and distribution of PBX-HOX and MEIS/PREP motifs. (PPTX) [file pone.0130413.s005.pptx]

## Slide 1
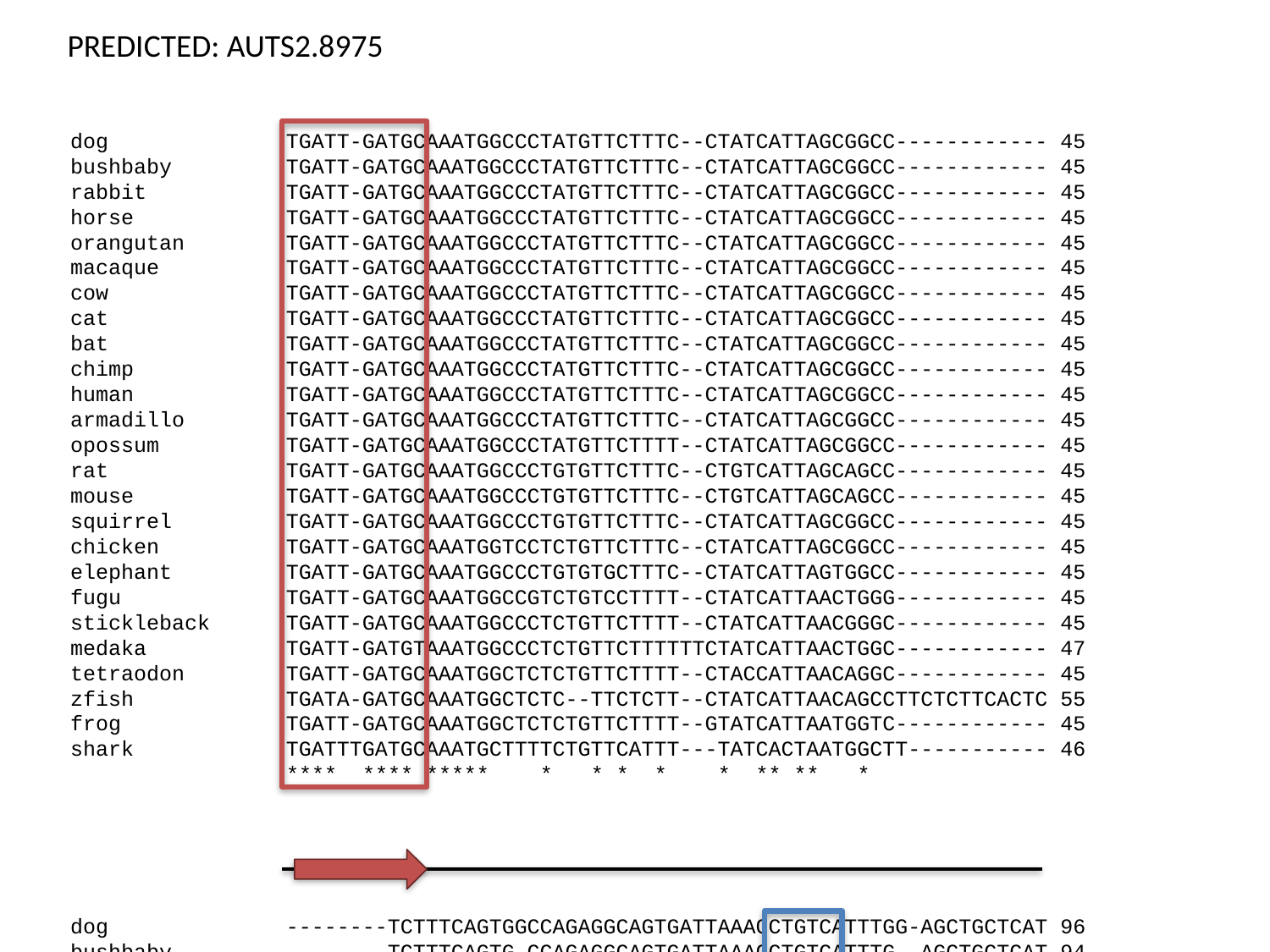

PREDICTED: AUTS2.8975
dog TGATT-GATGCAAATGGCCCTATGTTCTTTC--CTATCATTAGCGGCC------------ 45
bushbaby TGATT-GATGCAAATGGCCCTATGTTCTTTC--CTATCATTAGCGGCC------------ 45
rabbit TGATT-GATGCAAATGGCCCTATGTTCTTTC--CTATCATTAGCGGCC------------ 45
horse TGATT-GATGCAAATGGCCCTATGTTCTTTC--CTATCATTAGCGGCC------------ 45
orangutan TGATT-GATGCAAATGGCCCTATGTTCTTTC--CTATCATTAGCGGCC------------ 45
macaque TGATT-GATGCAAATGGCCCTATGTTCTTTC--CTATCATTAGCGGCC------------ 45
cow TGATT-GATGCAAATGGCCCTATGTTCTTTC--CTATCATTAGCGGCC------------ 45
cat TGATT-GATGCAAATGGCCCTATGTTCTTTC--CTATCATTAGCGGCC------------ 45
bat TGATT-GATGCAAATGGCCCTATGTTCTTTC--CTATCATTAGCGGCC------------ 45
chimp TGATT-GATGCAAATGGCCCTATGTTCTTTC--CTATCATTAGCGGCC------------ 45
human TGATT-GATGCAAATGGCCCTATGTTCTTTC--CTATCATTAGCGGCC------------ 45
armadillo TGATT-GATGCAAATGGCCCTATGTTCTTTC--CTATCATTAGCGGCC------------ 45
opossum TGATT-GATGCAAATGGCCCTATGTTCTTTT--CTATCATTAGCGGCC------------ 45
rat TGATT-GATGCAAATGGCCCTGTGTTCTTTC--CTGTCATTAGCAGCC------------ 45
mouse TGATT-GATGCAAATGGCCCTGTGTTCTTTC--CTGTCATTAGCAGCC------------ 45
squirrel TGATT-GATGCAAATGGCCCTGTGTTCTTTC--CTATCATTAGCGGCC------------ 45
chicken TGATT-GATGCAAATGGTCCTCTGTTCTTTC--CTATCATTAGCGGCC------------ 45
elephant TGATT-GATGCAAATGGCCCTGTGTGCTTTC--CTATCATTAGTGGCC------------ 45
fugu TGATT-GATGCAAATGGCCGTCTGTCCTTTT--CTATCATTAACTGGG------------ 45
stickleback TGATT-GATGCAAATGGCCCTCTGTTCTTTT--CTATCATTAACGGGC------------ 45
medaka TGATT-GATGTAAATGGCCCTCTGTTCTTTTTTCTATCATTAACTGGC------------ 47
tetraodon TGATT-GATGCAAATGGCTCTCTGTTCTTTT--CTACCATTAACAGGC------------ 45
zfish TGATA-GATGCAAATGGCTCTC--TTCTCTT--CTATCATTAACAGCCTTCTCTTCACTC 55
frog TGATT-GATGCAAATGGCTCTCTGTTCTTTT--GTATCATTAATGGTC------------ 45
shark TGATTTGATGCAAATGCTTTTCTGTTCATTT---TATCACTAATGGCTT----------- 46
 **** **** ***** * * * * * ** ** *
dog --------TCTTTCAGTGGCCAGAGGCAGTGATTAAAGCTGTCATTTGG-AGCTGCTCAT 96
bushbaby --------TCTTTCAGTG-CCAGAGGCAGTGATTAAAGCTGTCATTTG--AGCTGCTCAT 94
rabbit --------TCTTTCAGTGGCCAGAGGCAGTGATTAAAGCTGTCATTTGG-AGCTGCTCAT 96
horse --------TCTTTCAGTGGCCAGAGGCAGTGATTAAAGCTGTCATTTGG-AGCTGCTCAT 96
orangutan --------TCTTTCAGTGGCCAGAGGCAGTGATTAAAGCTGTCATTTGG-AGCTGCTCAT 96
macaque --------TCTTTCAGTGGCCAGAGGCAGTGATTAAAGCTGTCATTTGG-AGCTGCTCAT 96
cow --------TCTTTCAGTGGCCAGAGGCAGTGATTAAAGCTGTCATTTGG-AGCTGCTCAT 96
cat --------TCTTTCAGTGGCCAGAGGCAGTGATTAAAGCTGTCATTTGG-AGCTGCTCAT 96
bat --------TCTTTCAGTGGCCAGAGGCAGTGATTAAAGCTGTCATTTGG-AGCTGCTCAT 96
chimp --------TCTTTCAGTGGCCAGAGGCAGTGATTAAAGCTGTCATTTGG-AGCTGCTCAT 96
human --------TCTTTCAGTGGCCAGAGGCAGTGATTAAAGCTGTCATTTGG-AGCTGCTCAT 96
armadillo --------TCTTTCAGTGGCCAGAGGCAATGATTAAAGCTGTCATTTGG-AGCTGCTCAT 96
opossum --------TCTTTCAGTGGCCAGAGGCAGTGATTAAAGCTGTCATTTGG-AGCTGCTCAT 96
rat --------TCTTTCAGTGGCCAGAGGCAGTGATTAAAGCTGTCATTTGG-AGCTGCTCAT 96
mouse --------TCTTTCAGTGGCCAGAGGCAGTGATTAAAGCTGTCATTTGG-AGCTGCTCAT 96
squirrel --------TCTTTCAGTGGCCAGAGGCAGTGATTAAAGCTGTCATTTGG-AGCTGCTCAT 96
chicken --------TCTTTCAGTGGCCAGAGGCAGTGATTAAAGCTGTCATTTGG-AGCTGCTCAT 96
elephant --------TCTCTCAGTGGCCAGAGGCAGTGATTAAAGCTGTCATTTGG-AGCTGCTCAT 96
fugu -------CTTTGC-TGTGGCCACAGGCACTGATTAAAGCCATCATTTTT-AGCTACTTGT 96
stickleback -------CCTTCCCTGTGGCCACAGGCGGTGATTAAAGCTGTCATTTCC-AGCTACTCAT 97
medaka -------CCTTTGCCGTGGCCACAGGCAGTGATTAAAGCTGTCATTTCC-AGCGACTCAT 99
tetraodon -------CCCTACCG--GGCCACAGGCAATGATTGAAGCTGTCTTTTTTTACCTACTCAT 96
zfish TCTTTCTCTCTCTCGACGGCCACAGGCAGTGATTAAAGCTGTCATCTAC-AGCTACTCAT 114
frog --------TCTCTCAGTGGCCAGAGGCAGTGATTAAAGCTGTCATTTGG-AGCTGCTCAT 96
shark -------CTCTCTCTGTGGCCAGAGGCGGGGATTAAGGCTGGC--TTCAGGGCGGCTCAT 97
 * * *** **** **** * ** * * * ** *

## Slide 2
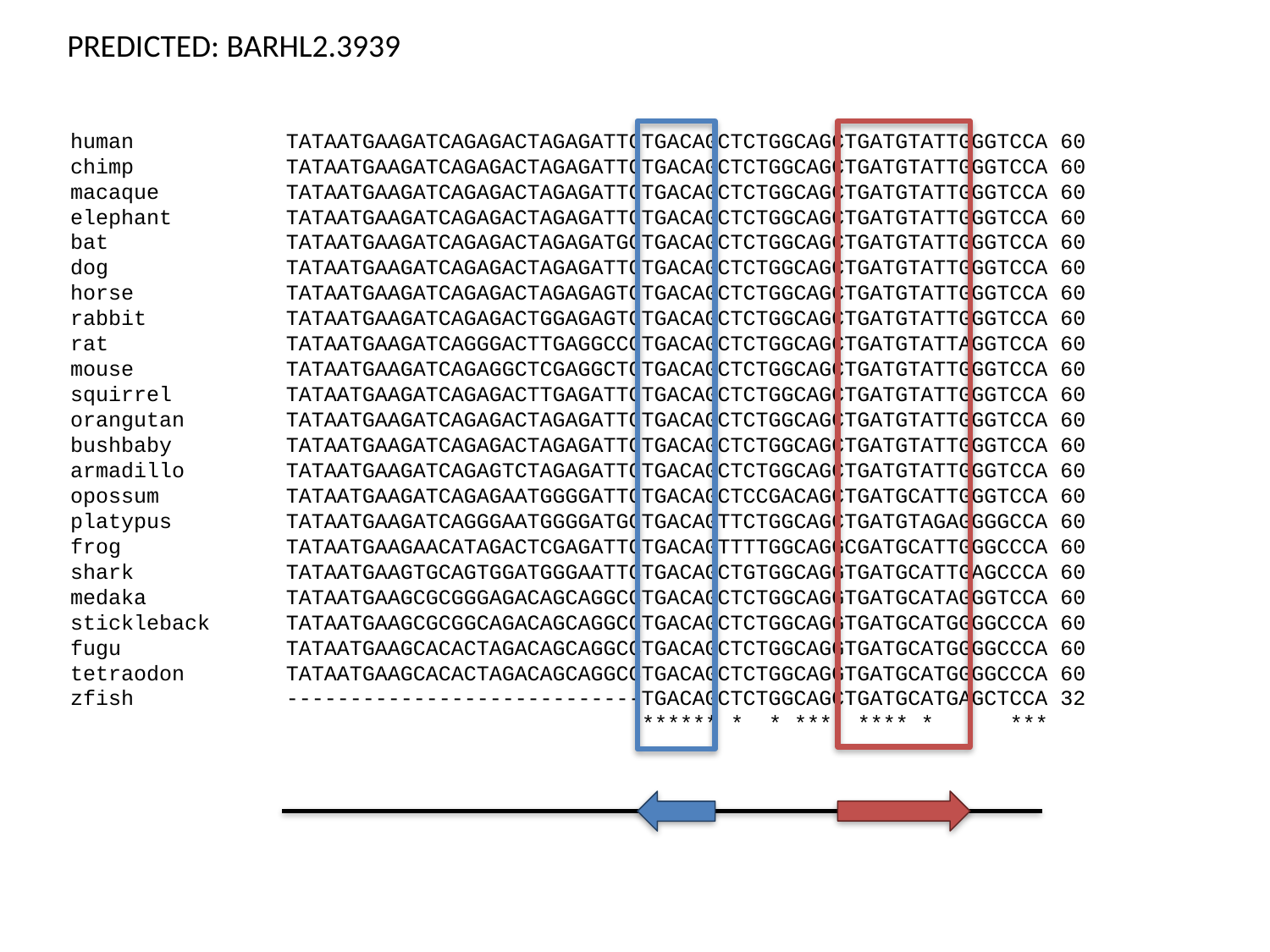

PREDICTED: BARHL2.3939
human TATAATGAAGATCAGAGACTAGAGATTGTGACAGCTCTGGCAGCTGATGTATTGGGTCCA 60
chimp TATAATGAAGATCAGAGACTAGAGATTGTGACAGCTCTGGCAGCTGATGTATTGGGTCCA 60
macaque TATAATGAAGATCAGAGACTAGAGATTGTGACAGCTCTGGCAGCTGATGTATTGGGTCCA 60
elephant TATAATGAAGATCAGAGACTAGAGATTGTGACAGCTCTGGCAGCTGATGTATTGGGTCCA 60
bat TATAATGAAGATCAGAGACTAGAGATGGTGACAGCTCTGGCAGCTGATGTATTGGGTCCA 60
dog TATAATGAAGATCAGAGACTAGAGATTGTGACAGCTCTGGCAGCTGATGTATTGGGTCCA 60
horse TATAATGAAGATCAGAGACTAGAGAGTGTGACAGCTCTGGCAGCTGATGTATTGGGTCCA 60
rabbit TATAATGAAGATCAGAGACTGGAGAGTGTGACAGCTCTGGCAGCTGATGTATTGGGTCCA 60
rat TATAATGAAGATCAGGGACTTGAGGCCGTGACAGCTCTGGCAGCTGATGTATTAGGTCCA 60
mouse TATAATGAAGATCAGAGGCTCGAGGCTGTGACAGCTCTGGCAGCTGATGTATTGGGTCCA 60
squirrel TATAATGAAGATCAGAGACTTGAGATTGTGACAGCTCTGGCAGCTGATGTATTGGGTCCA 60
orangutan TATAATGAAGATCAGAGACTAGAGATTGTGACAGCTCTGGCAGCTGATGTATTGGGTCCA 60
bushbaby TATAATGAAGATCAGAGACTAGAGATTGTGACAGCTCTGGCAGCTGATGTATTGGGTCCA 60
armadillo TATAATGAAGATCAGAGTCTAGAGATTGTGACAGCTCTGGCAGCTGATGTATTGGGTCCA 60
opossum TATAATGAAGATCAGAGAATGGGGATTGTGACAGCTCCGACAGCTGATGCATTGGGTCCA 60
platypus TATAATGAAGATCAGGGAATGGGGATGGTGACAGTTCTGGCAGCTGATGTAGAGGGGCCA 60
frog TATAATGAAGAACATAGACTCGAGATTCTGACAGTTTTGGCAGGCGATGCATTGGGCCCA 60
shark TATAATGAAGTGCAGTGGATGGGAATTCTGACAGCTGTGGCAGGTGATGCATTGAGCCCA 60
medaka TATAATGAAGCGCGGGAGACAGCAGGCCTGACAGCTCTGGCAGGTGATGCATAGGGTCCA 60
stickleback TATAATGAAGCGCGGCAGACAGCAGGCCTGACAGCTCTGGCAGGTGATGCATGGGGCCCA 60
fugu TATAATGAAGCACACTAGACAGCAGGCCTGACAGCTCTGGCAGGTGATGCATGGGGCCCA 60
tetraodon TATAATGAAGCACACTAGACAGCAGGCCTGACAGCTCTGGCAGGTGATGCATGGGGCCCA 60
zfish ----------------------------TGACAGCTCTGGCAGCTGATGCATGAGCTCCA 32
 ****** * * *** **** * ***

## Slide 3
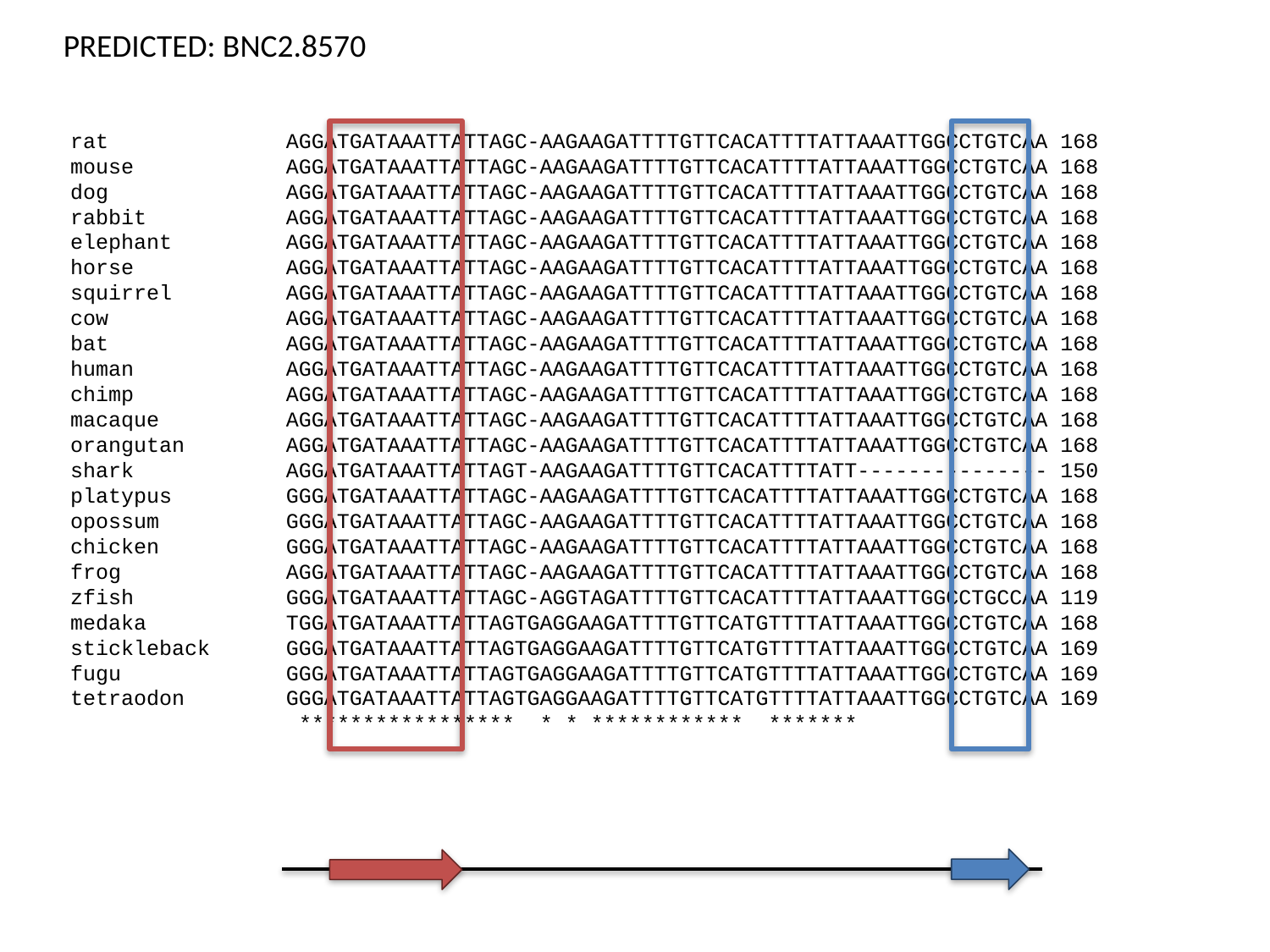

PREDICTED: BNC2.8570
rat AGGATGATAAATTATTAGC-AAGAAGATTTTGTTCACATTTTATTAAATTGGCCTGTCAA 168
mouse AGGATGATAAATTATTAGC-AAGAAGATTTTGTTCACATTTTATTAAATTGGCCTGTCAA 168
dog AGGATGATAAATTATTAGC-AAGAAGATTTTGTTCACATTTTATTAAATTGGCCTGTCAA 168
rabbit AGGATGATAAATTATTAGC-AAGAAGATTTTGTTCACATTTTATTAAATTGGCCTGTCAA 168
elephant AGGATGATAAATTATTAGC-AAGAAGATTTTGTTCACATTTTATTAAATTGGCCTGTCAA 168
horse AGGATGATAAATTATTAGC-AAGAAGATTTTGTTCACATTTTATTAAATTGGCCTGTCAA 168
squirrel AGGATGATAAATTATTAGC-AAGAAGATTTTGTTCACATTTTATTAAATTGGCCTGTCAA 168
cow AGGATGATAAATTATTAGC-AAGAAGATTTTGTTCACATTTTATTAAATTGGCCTGTCAA 168
bat AGGATGATAAATTATTAGC-AAGAAGATTTTGTTCACATTTTATTAAATTGGCCTGTCAA 168
human AGGATGATAAATTATTAGC-AAGAAGATTTTGTTCACATTTTATTAAATTGGCCTGTCAA 168
chimp AGGATGATAAATTATTAGC-AAGAAGATTTTGTTCACATTTTATTAAATTGGCCTGTCAA 168
macaque AGGATGATAAATTATTAGC-AAGAAGATTTTGTTCACATTTTATTAAATTGGCCTGTCAA 168
orangutan AGGATGATAAATTATTAGC-AAGAAGATTTTGTTCACATTTTATTAAATTGGCCTGTCAA 168
shark AGGATGATAAATTATTAGT-AAGAAGATTTTGTTCACATTTTATT--------------- 150
platypus GGGATGATAAATTATTAGC-AAGAAGATTTTGTTCACATTTTATTAAATTGGCCTGTCAA 168
opossum GGGATGATAAATTATTAGC-AAGAAGATTTTGTTCACATTTTATTAAATTGGCCTGTCAA 168
chicken GGGATGATAAATTATTAGC-AAGAAGATTTTGTTCACATTTTATTAAATTGGCCTGTCAA 168
frog AGGATGATAAATTATTAGC-AAGAAGATTTTGTTCACATTTTATTAAATTGGCCTGTCAA 168
zfish GGGATGATAAATTATTAGC-AGGTAGATTTTGTTCACATTTTATTAAATTGGCCTGCCAA 119
medaka TGGATGATAAATTATTAGTGAGGAAGATTTTGTTCATGTTTTATTAAATTGGCCTGTCAA 168
stickleback GGGATGATAAATTATTAGTGAGGAAGATTTTGTTCATGTTTTATTAAATTGGCCTGTCAA 169
fugu GGGATGATAAATTATTAGTGAGGAAGATTTTGTTCATGTTTTATTAAATTGGCCTGTCAA 169
tetraodon GGGATGATAAATTATTAGTGAGGAAGATTTTGTTCATGTTTTATTAAATTGGCCTGTCAA 169
 ***************** * * ************ *******

## Slide 4
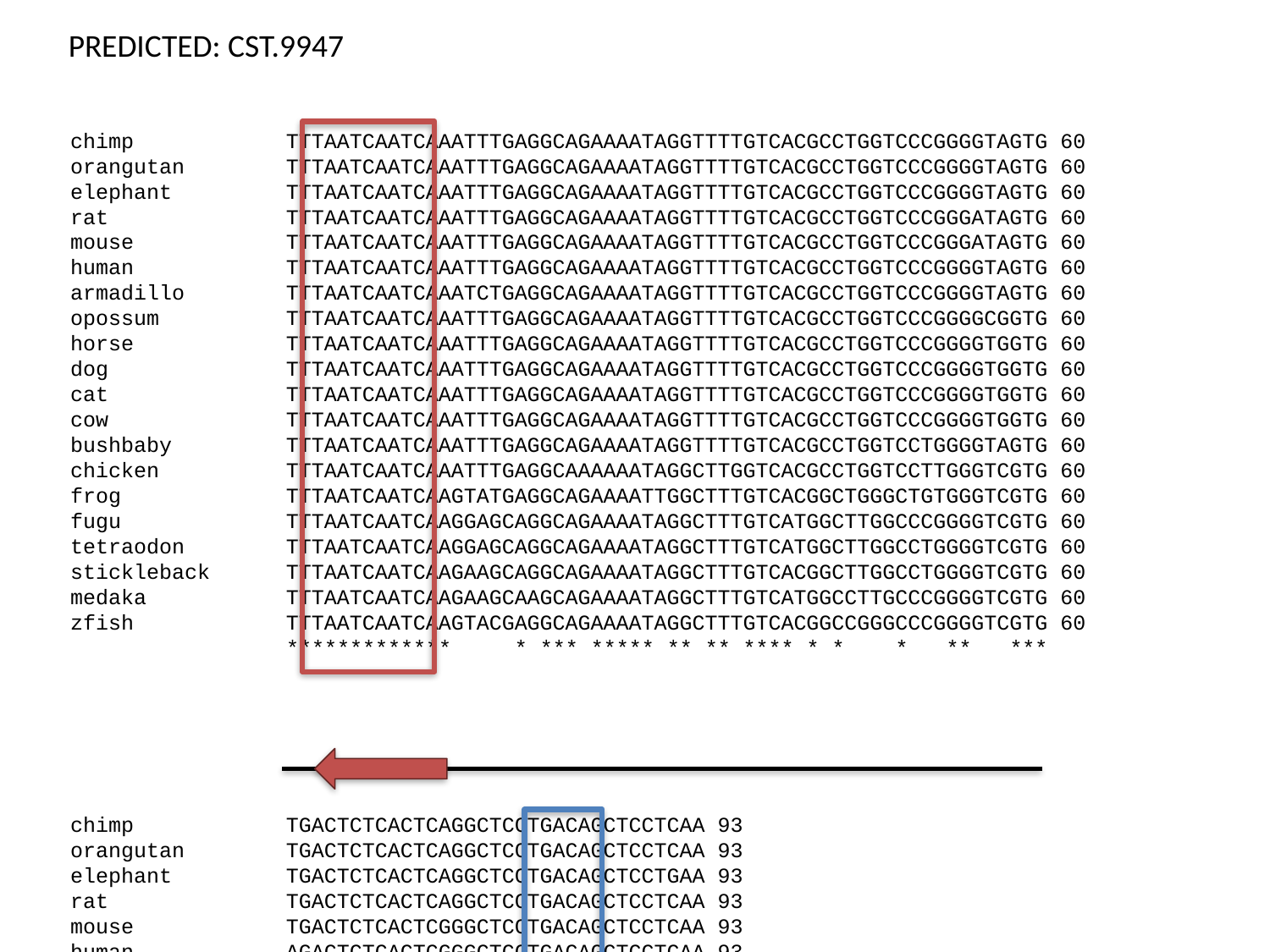

PREDICTED: CST.9947
chimp TTTAATCAATCAAATTTGAGGCAGAAAATAGGTTTTGTCACGCCTGGTCCCGGGGTAGTG 60
orangutan TTTAATCAATCAAATTTGAGGCAGAAAATAGGTTTTGTCACGCCTGGTCCCGGGGTAGTG 60
elephant TTTAATCAATCAAATTTGAGGCAGAAAATAGGTTTTGTCACGCCTGGTCCCGGGGTAGTG 60
rat TTTAATCAATCAAATTTGAGGCAGAAAATAGGTTTTGTCACGCCTGGTCCCGGGATAGTG 60
mouse TTTAATCAATCAAATTTGAGGCAGAAAATAGGTTTTGTCACGCCTGGTCCCGGGATAGTG 60
human TTTAATCAATCAAATTTGAGGCAGAAAATAGGTTTTGTCACGCCTGGTCCCGGGGTAGTG 60
armadillo TTTAATCAATCAAATCTGAGGCAGAAAATAGGTTTTGTCACGCCTGGTCCCGGGGTAGTG 60
opossum TTTAATCAATCAAATTTGAGGCAGAAAATAGGTTTTGTCACGCCTGGTCCCGGGGCGGTG 60
horse TTTAATCAATCAAATTTGAGGCAGAAAATAGGTTTTGTCACGCCTGGTCCCGGGGTGGTG 60
dog TTTAATCAATCAAATTTGAGGCAGAAAATAGGTTTTGTCACGCCTGGTCCCGGGGTGGTG 60
cat TTTAATCAATCAAATTTGAGGCAGAAAATAGGTTTTGTCACGCCTGGTCCCGGGGTGGTG 60
cow TTTAATCAATCAAATTTGAGGCAGAAAATAGGTTTTGTCACGCCTGGTCCCGGGGTGGTG 60
bushbaby TTTAATCAATCAAATTTGAGGCAGAAAATAGGTTTTGTCACGCCTGGTCCTGGGGTAGTG 60
chicken TTTAATCAATCAAATTTGAGGCAAAAAATAGGCTTGGTCACGCCTGGTCCTTGGGTCGTG 60
frog TTTAATCAATCAAGTATGAGGCAGAAAATTGGCTTTGTCACGGCTGGGCTGTGGGTCGTG 60
fugu TTTAATCAATCAAGGAGCAGGCAGAAAATAGGCTTTGTCATGGCTTGGCCCGGGGTCGTG 60
tetraodon TTTAATCAATCAAGGAGCAGGCAGAAAATAGGCTTTGTCATGGCTTGGCCTGGGGTCGTG 60
stickleback TTTAATCAATCAAGAAGCAGGCAGAAAATAGGCTTTGTCACGGCTTGGCCTGGGGTCGTG 60
medaka TTTAATCAATCAAGAAGCAAGCAGAAAATAGGCTTTGTCATGGCCTTGCCCGGGGTCGTG 60
zfish TTTAATCAATCAAGTACGAGGCAGAAAATAGGCTTTGTCACGGCCGGGCCCGGGGTCGTG 60
 ************* * *** ***** ** ** **** * * * ** ***
chimp TGACTCTCACTCAGGCTCCTGACAGCTCCTCAA 93
orangutan TGACTCTCACTCAGGCTCCTGACAGCTCCTCAA 93
elephant TGACTCTCACTCAGGCTCCTGACAGCTCCTGAA 93
rat TGACTCTCACTCAGGCTCCTGACAGCTCCTCAA 93
mouse TGACTCTCACTCGGGCTCCTGACAGCTCCTCAA 93
human AGACTCTCACTCGGGCTCCTGACAGCTCCTCAA 93
armadillo TGACTCTCACTCAGGCTCCTGACAGCTCCTCAA 93
opossum TGGCTCTCACTCAGGCTCCTGACAGCTCCTCA- 92
horse TGGCTCTCACTCAGGCTCCTGACAGCTCCTCAA 93
dog TGGCTCTCACTCAGGCTCCTGACAGCTCCTCAA 93
cat TGGCTCTCACTCAGGCTCCTGACAGCTCCTCAA 93
cow TGGCTCTCACTCAGGCTCCTGACAGCTCCTCAA 93
bushbaby TGACTCTCACTCAGGCTCCTGACAGCTCCTCAA 93
chicken TGACACTCACTCAGGGTCCTGACAGCTCCTCAA 93
frog TGACAATCACACTGTCTCTTGACAGCT------ 87
fugu TGATGCCTGCTCTGCCCGGTGACAGGTCTTCAA 93
tetraodon TGATGCCTGCTCTGCCCGGTGACAGGTCTTCAA 93
stickleback TGATGCCTGGTCTTCCCGGTGACAGGTCTTCAA 93
medaka TGATGCCTGCTCTGCCTGGTGACAGGTCTTCAA 93
zfish TGATGCTCGCTTTGCGCACTGACAGCTCCTCAA 93
 * ****** *

## Slide 5
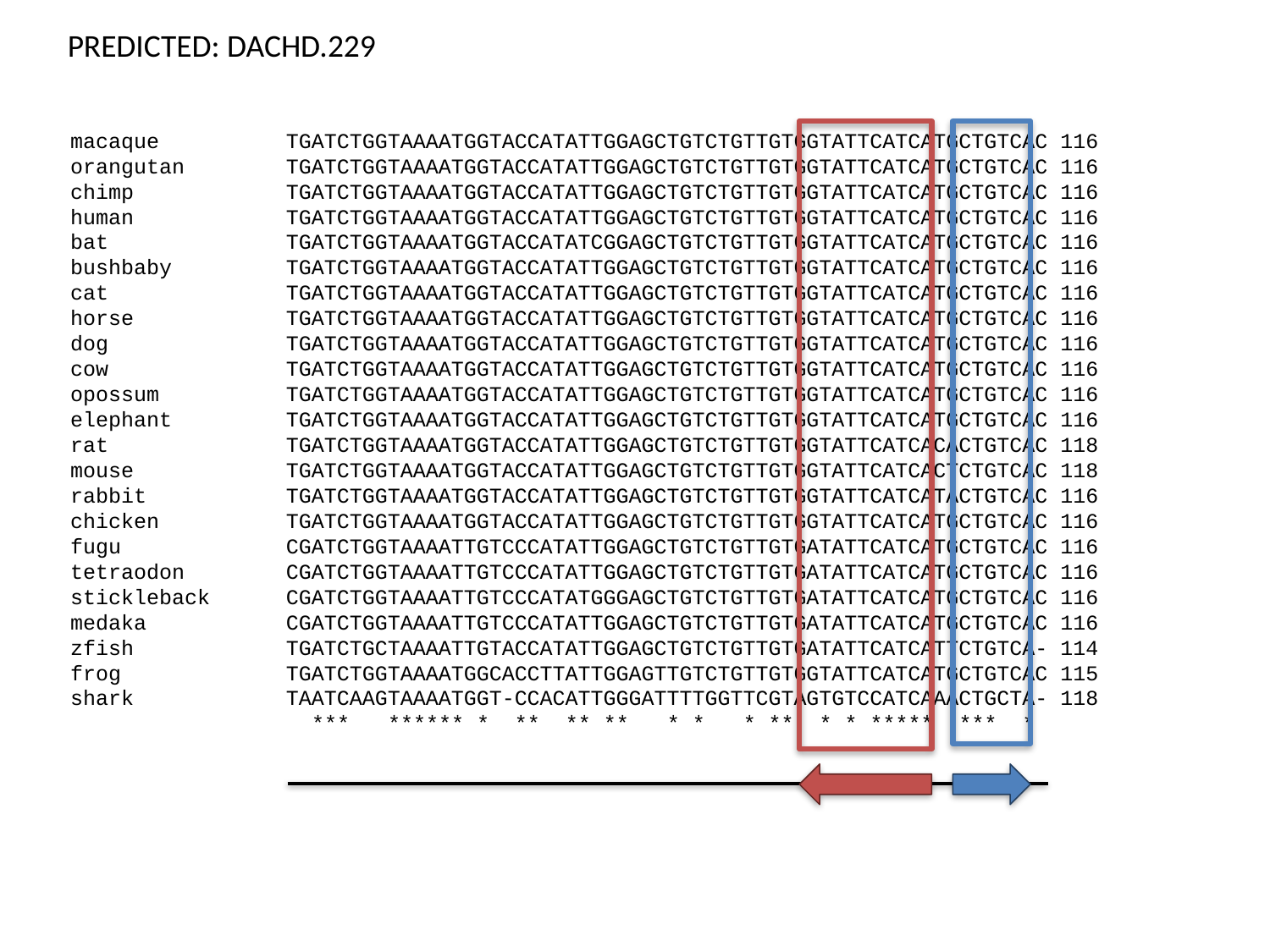

PREDICTED: DACHD.229
macaque TGATCTGGTAAAATGGTACCATATTGGAGCTGTCTGTTGTGGTATTCATCATGCTGTCAC 116
orangutan TGATCTGGTAAAATGGTACCATATTGGAGCTGTCTGTTGTGGTATTCATCATGCTGTCAC 116
chimp TGATCTGGTAAAATGGTACCATATTGGAGCTGTCTGTTGTGGTATTCATCATGCTGTCAC 116
human TGATCTGGTAAAATGGTACCATATTGGAGCTGTCTGTTGTGGTATTCATCATGCTGTCAC 116
bat TGATCTGGTAAAATGGTACCATATCGGAGCTGTCTGTTGTGGTATTCATCATGCTGTCAC 116
bushbaby TGATCTGGTAAAATGGTACCATATTGGAGCTGTCTGTTGTGGTATTCATCATGCTGTCAC 116
cat TGATCTGGTAAAATGGTACCATATTGGAGCTGTCTGTTGTGGTATTCATCATGCTGTCAC 116
horse TGATCTGGTAAAATGGTACCATATTGGAGCTGTCTGTTGTGGTATTCATCATGCTGTCAC 116
dog TGATCTGGTAAAATGGTACCATATTGGAGCTGTCTGTTGTGGTATTCATCATGCTGTCAC 116
cow TGATCTGGTAAAATGGTACCATATTGGAGCTGTCTGTTGTGGTATTCATCATGCTGTCAC 116
opossum TGATCTGGTAAAATGGTACCATATTGGAGCTGTCTGTTGTGGTATTCATCATGCTGTCAC 116
elephant TGATCTGGTAAAATGGTACCATATTGGAGCTGTCTGTTGTGGTATTCATCATGCTGTCAC 116
rat TGATCTGGTAAAATGGTACCATATTGGAGCTGTCTGTTGTGGTATTCATCACACTGTCAC 118
mouse TGATCTGGTAAAATGGTACCATATTGGAGCTGTCTGTTGTGGTATTCATCACTCTGTCAC 118
rabbit TGATCTGGTAAAATGGTACCATATTGGAGCTGTCTGTTGTGGTATTCATCATACTGTCAC 116
chicken TGATCTGGTAAAATGGTACCATATTGGAGCTGTCTGTTGTGGTATTCATCATGCTGTCAC 116
fugu CGATCTGGTAAAATTGTCCCATATTGGAGCTGTCTGTTGTGATATTCATCATGCTGTCAC 116
tetraodon CGATCTGGTAAAATTGTCCCATATTGGAGCTGTCTGTTGTGATATTCATCATGCTGTCAC 116
stickleback CGATCTGGTAAAATTGTCCCATATGGGAGCTGTCTGTTGTGATATTCATCATGCTGTCAC 116
medaka CGATCTGGTAAAATTGTCCCATATTGGAGCTGTCTGTTGTGATATTCATCATGCTGTCAC 116
zfish TGATCTGCTAAAATTGTACCATATTGGAGCTGTCTGTTGTGATATTCATCATTCTGTCA- 114
frog TGATCTGGTAAAATGGCACCTTATTGGAGTTGTCTGTTGTGGTATTCATCATGCTGTCAC 115
shark TAATCAAGTAAAATGGT-CCACATTGGGATTTTGGTTCGTAGTGTCCATCAAACTGCTA- 118
 *** ****** * ** ** ** * * * ** * * ***** *** *

## Slide 6
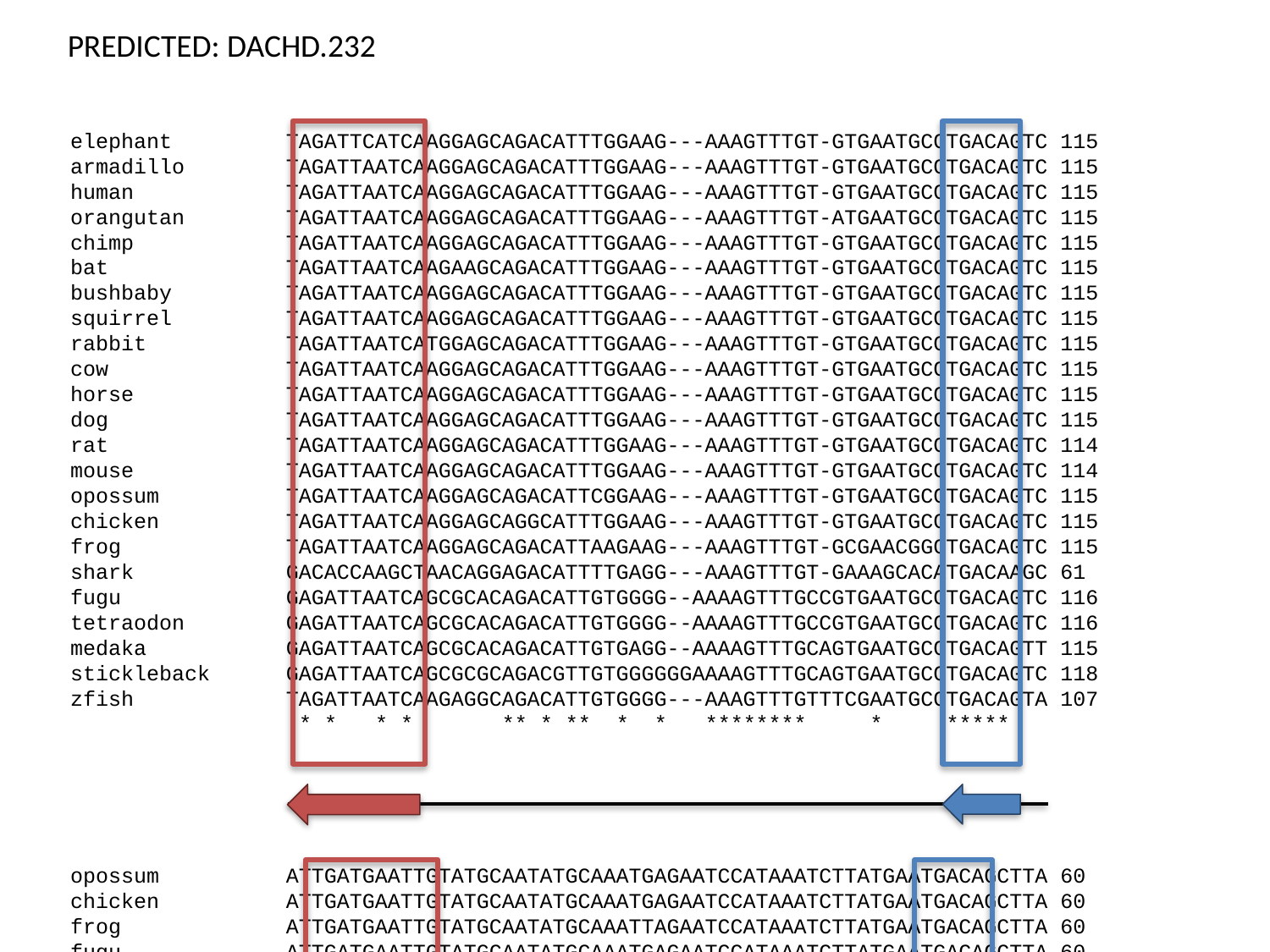

PREDICTED: DACHD.232
elephant TAGATTCATCAAGGAGCAGACATTTGGAAG---AAAGTTTGT-GTGAATGCCTGACAGTC 115
armadillo TAGATTAATCAAGGAGCAGACATTTGGAAG---AAAGTTTGT-GTGAATGCCTGACAGTC 115
human TAGATTAATCAAGGAGCAGACATTTGGAAG---AAAGTTTGT-GTGAATGCCTGACAGTC 115
orangutan TAGATTAATCAAGGAGCAGACATTTGGAAG---AAAGTTTGT-ATGAATGCCTGACAGTC 115
chimp TAGATTAATCAAGGAGCAGACATTTGGAAG---AAAGTTTGT-GTGAATGCCTGACAGTC 115
bat TAGATTAATCAAGAAGCAGACATTTGGAAG---AAAGTTTGT-GTGAATGCCTGACAGTC 115
bushbaby TAGATTAATCAAGGAGCAGACATTTGGAAG---AAAGTTTGT-GTGAATGCCTGACAGTC 115
squirrel TAGATTAATCAAGGAGCAGACATTTGGAAG---AAAGTTTGT-GTGAATGCCTGACAGTC 115
rabbit TAGATTAATCATGGAGCAGACATTTGGAAG---AAAGTTTGT-GTGAATGCCTGACAGTC 115
cow TAGATTAATCAAGGAGCAGACATTTGGAAG---AAAGTTTGT-GTGAATGCCTGACAGTC 115
horse TAGATTAATCAAGGAGCAGACATTTGGAAG---AAAGTTTGT-GTGAATGCCTGACAGTC 115
dog TAGATTAATCAAGGAGCAGACATTTGGAAG---AAAGTTTGT-GTGAATGCCTGACAGTC 115
rat TAGATTAATCAAGGAGCAGACATTTGGAAG---AAAGTTTGT-GTGAATGCCTGACAGTC 114
mouse TAGATTAATCAAGGAGCAGACATTTGGAAG---AAAGTTTGT-GTGAATGCCTGACAGTC 114
opossum TAGATTAATCAAGGAGCAGACATTCGGAAG---AAAGTTTGT-GTGAATGCCTGACAGTC 115
chicken TAGATTAATCAAGGAGCAGGCATTTGGAAG---AAAGTTTGT-GTGAATGCCTGACAGTC 115
frog TAGATTAATCAAGGAGCAGACATTAAGAAG---AAAGTTTGT-GCGAACGGCTGACAGTC 115
shark GACACCAAGCTAACAGGAGACATTTTGAGG---AAAGTTTGT-GAAAGCACATGACAAGC 61
fugu GAGATTAATCAGCGCACAGACATTGTGGGG--AAAAGTTTGCCGTGAATGCCTGACAGTC 116
tetraodon GAGATTAATCAGCGCACAGACATTGTGGGG--AAAAGTTTGCCGTGAATGCCTGACAGTC 116
medaka GAGATTAATCAGCGCACAGACATTGTGAGG--AAAAGTTTGCAGTGAATGCCTGACAGTT 115
stickleback GAGATTAATCAGCGCGCAGACGTTGTGGGGGGAAAAGTTTGCAGTGAATGCCTGACAGTC 118
zfish TAGATTAATCAAGAGGCAGACATTGTGGGG---AAAGTTTGTTTCGAATGCCTGACAGTA 107
 * * * * ** * ** * * ******** * *****
opossum ATTGATGAATTGTATGCAATATGCAAATGAGAATCCATAAATCTTATGAATGACAGCTTA 60
chicken ATTGATGAATTGTATGCAATATGCAAATGAGAATCCATAAATCTTATGAATGACAGCTTA 60
frog ATTGATGAATTGTATGCAATATGCAAATTAGAATCCATAAATCTTATGAATGACAGCTTA 60
fugu ATTGATGAATTGTATGCAATATGCAAATGAGAATCCATAAATCTTATGAATGACAGCTTA 60
tetraodon ATTGATGAATTGTATGCAATATGCAAATGAGAATCCATAAATCTTATGAATGACAGCTTA 60
medaka ATTGATGAATTGTATGCAATATGCAAATGAGAATCCATAAATCTTATGAATGACAGCTTA 60
stickleback ATTGATGAATTGTATGCAATATGCAAATGAGAATCCATAAATCTTATGAATGACAGCTTA 60
zfish ATTGATGAATTGTATGCAATATGCAAATGAGAATCCATAAATCTTATGAATGACAGCTTA 60
shark ATTGATGAATTGTATGCAATATGCAAATGAGAATACATAAATCTTATGAATGACAGCTTA 60
rat ATTGATGAATTGTATGCAATATGCAAATGAGAATCCATAAATCTTATGAATGACAGCTTA 60
mouse ATTGATGAATTGTATGCAATATGCAAATGAGAATCCATAAATCTTATGAATGACAGCTTA 60
human ATTGATGAATTGTATGCAATATGCAAATGAGAATCCATAAATCTTATGAATGACAGCTTA 60
orangutan ATTGATGAATTGTATGCAATATGCAAATGAGAATCCATAAATCTTATGAATGACAGCTTA 60
chimp ATTGATGAATTGTATGCAATATGCAAATGAGAATCCATAAATCTTATGAATGACAGCTTA 60
bushbaby ATTGATGAATTGTATGCAATATGCAAATGAGAATCCATAAATCTTATGAATGACAGCTTA 60
squirrel ATTGATGAATTGTATGCAATATGCAAATGAGAATCCATAAATCTTATGAATGACAGCTTA 60
rabbit ATTGATGAATTGTATGCAATATGCAAATGAGAATCCATAAATCTTATGAATGACAGCTTA 60
elephant ATTGATGAATTGCATGCAATATGCAAATGAGAATCCATAAATCTTATGAATGACAGCTTA 60
armadillo ATTGATGAATTGTATGCAATATGCAAATGAGAATCCATAAATCTTATGAATGACAGCTTA 60
dog ATTGATGAATTGTATGCAATATGCAAATGAGAATCCATAAATCTTATGAATGACAGCTTA 60
horse ATTGATGAATTGTATGCAATATGCAAATGAGAATCCATAAATCTTATGAATGACAGCTTA 60
cow ATTGATGAATTGTATGCAATATGCAAATGAGAATCCATAAATCTTATGAATGACAGCTTA 60
bat ATTGATGAATTGTATGCAATATGCAAATGAGAATCCATAAATCTTATGAATGACAGCTTA 60
 ************ *************** ***** *************************

## Slide 7
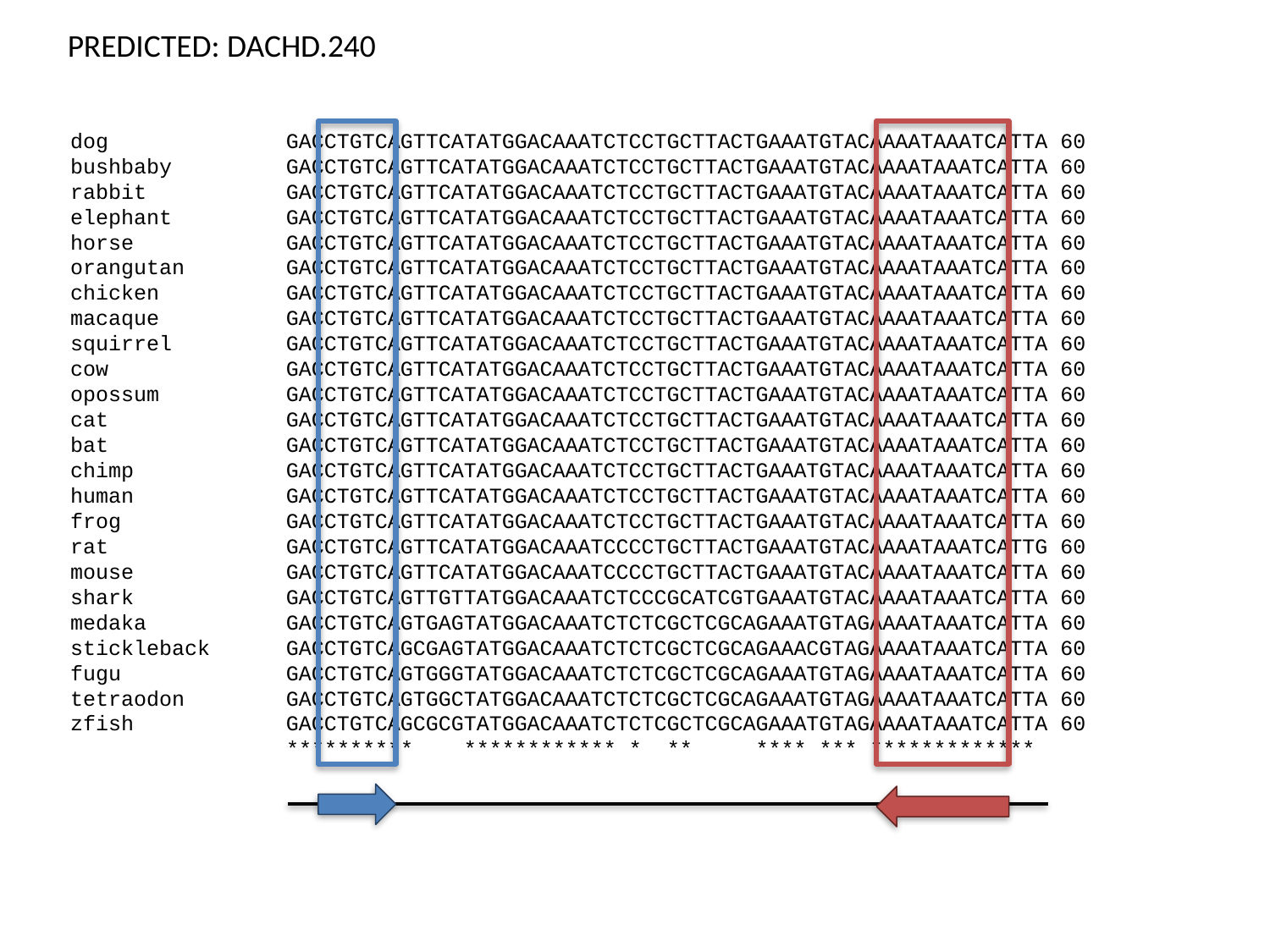

PREDICTED: DACHD.240
dog GACCTGTCAGTTCATATGGACAAATCTCCTGCTTACTGAAATGTACAAAATAAATCATTA 60
bushbaby GACCTGTCAGTTCATATGGACAAATCTCCTGCTTACTGAAATGTACAAAATAAATCATTA 60
rabbit GACCTGTCAGTTCATATGGACAAATCTCCTGCTTACTGAAATGTACAAAATAAATCATTA 60
elephant GACCTGTCAGTTCATATGGACAAATCTCCTGCTTACTGAAATGTACAAAATAAATCATTA 60
horse GACCTGTCAGTTCATATGGACAAATCTCCTGCTTACTGAAATGTACAAAATAAATCATTA 60
orangutan GACCTGTCAGTTCATATGGACAAATCTCCTGCTTACTGAAATGTACAAAATAAATCATTA 60
chicken GACCTGTCAGTTCATATGGACAAATCTCCTGCTTACTGAAATGTACAAAATAAATCATTA 60
macaque GACCTGTCAGTTCATATGGACAAATCTCCTGCTTACTGAAATGTACAAAATAAATCATTA 60
squirrel GACCTGTCAGTTCATATGGACAAATCTCCTGCTTACTGAAATGTACAAAATAAATCATTA 60
cow GACCTGTCAGTTCATATGGACAAATCTCCTGCTTACTGAAATGTACAAAATAAATCATTA 60
opossum GACCTGTCAGTTCATATGGACAAATCTCCTGCTTACTGAAATGTACAAAATAAATCATTA 60
cat GACCTGTCAGTTCATATGGACAAATCTCCTGCTTACTGAAATGTACAAAATAAATCATTA 60
bat GACCTGTCAGTTCATATGGACAAATCTCCTGCTTACTGAAATGTACAAAATAAATCATTA 60
chimp GACCTGTCAGTTCATATGGACAAATCTCCTGCTTACTGAAATGTACAAAATAAATCATTA 60
human GACCTGTCAGTTCATATGGACAAATCTCCTGCTTACTGAAATGTACAAAATAAATCATTA 60
frog GACCTGTCAGTTCATATGGACAAATCTCCTGCTTACTGAAATGTACAAAATAAATCATTA 60
rat GACCTGTCAGTTCATATGGACAAATCCCCTGCTTACTGAAATGTACAAAATAAATCATTG 60
mouse GACCTGTCAGTTCATATGGACAAATCCCCTGCTTACTGAAATGTACAAAATAAATCATTA 60
shark GACCTGTCAGTTGTTATGGACAAATCTCCCGCATCGTGAAATGTACAAAATAAATCATTA 60
medaka GACCTGTCAGTGAGTATGGACAAATCTCTCGCTCGCAGAAATGTAGAAAATAAATCATTA 60
stickleback GACCTGTCAGCGAGTATGGACAAATCTCTCGCTCGCAGAAACGTAGAAAATAAATCATTA 60
fugu GACCTGTCAGTGGGTATGGACAAATCTCTCGCTCGCAGAAATGTAGAAAATAAATCATTA 60
tetraodon GACCTGTCAGTGGCTATGGACAAATCTCTCGCTCGCAGAAATGTAGAAAATAAATCATTA 60
zfish GACCTGTCAGCGCGTATGGACAAATCTCTCGCTCGCAGAAATGTAGAAAATAAATCATTA 60
 ********** ************ * ** **** *** *************

## Slide 8
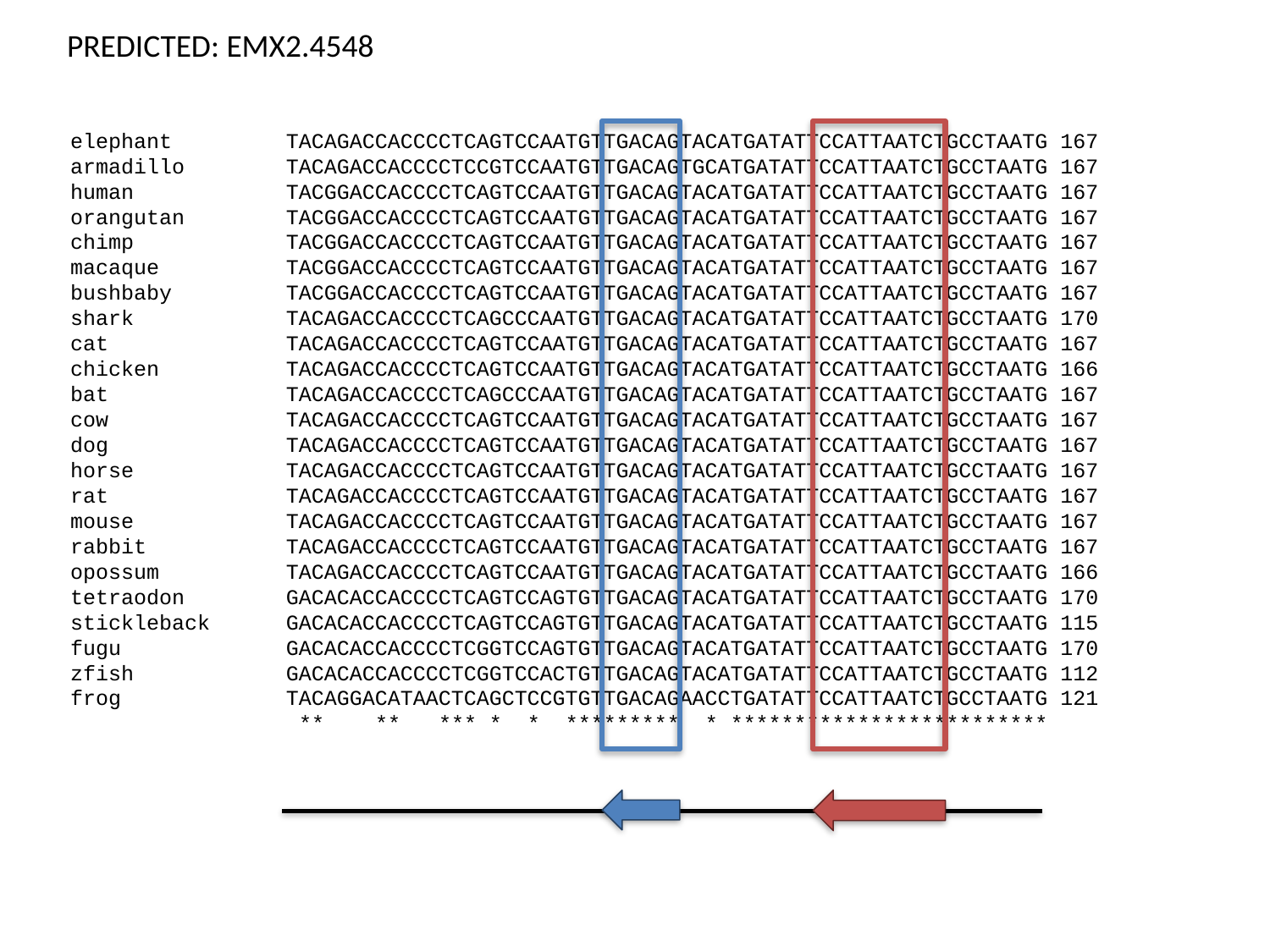

PREDICTED: EMX2.4548
elephant TACAGACCACCCCTCAGTCCAATGTTGACAGTACATGATATTCCATTAATCTGCCTAATG 167
armadillo TACAGACCACCCCTCCGTCCAATGTTGACAGTGCATGATATTCCATTAATCTGCCTAATG 167
human TACGGACCACCCCTCAGTCCAATGTTGACAGTACATGATATTCCATTAATCTGCCTAATG 167
orangutan TACGGACCACCCCTCAGTCCAATGTTGACAGTACATGATATTCCATTAATCTGCCTAATG 167
chimp TACGGACCACCCCTCAGTCCAATGTTGACAGTACATGATATTCCATTAATCTGCCTAATG 167
macaque TACGGACCACCCCTCAGTCCAATGTTGACAGTACATGATATTCCATTAATCTGCCTAATG 167
bushbaby TACGGACCACCCCTCAGTCCAATGTTGACAGTACATGATATTCCATTAATCTGCCTAATG 167
shark TACAGACCACCCCTCAGCCCAATGTTGACAGTACATGATATTCCATTAATCTGCCTAATG 170
cat TACAGACCACCCCTCAGTCCAATGTTGACAGTACATGATATTCCATTAATCTGCCTAATG 167
chicken TACAGACCACCCCTCAGTCCAATGTTGACAGTACATGATATTCCATTAATCTGCCTAATG 166
bat TACAGACCACCCCTCAGCCCAATGTTGACAGTACATGATATTCCATTAATCTGCCTAATG 167
cow TACAGACCACCCCTCAGTCCAATGTTGACAGTACATGATATTCCATTAATCTGCCTAATG 167
dog TACAGACCACCCCTCAGTCCAATGTTGACAGTACATGATATTCCATTAATCTGCCTAATG 167
horse TACAGACCACCCCTCAGTCCAATGTTGACAGTACATGATATTCCATTAATCTGCCTAATG 167
rat TACAGACCACCCCTCAGTCCAATGTTGACAGTACATGATATTCCATTAATCTGCCTAATG 167
mouse TACAGACCACCCCTCAGTCCAATGTTGACAGTACATGATATTCCATTAATCTGCCTAATG 167
rabbit TACAGACCACCCCTCAGTCCAATGTTGACAGTACATGATATTCCATTAATCTGCCTAATG 167
opossum TACAGACCACCCCTCAGTCCAATGTTGACAGTACATGATATTCCATTAATCTGCCTAATG 166
tetraodon GACACACCACCCCTCAGTCCAGTGTTGACAGTACATGATATTCCATTAATCTGCCTAATG 170
stickleback GACACACCACCCCTCAGTCCAGTGTTGACAGTACATGATATTCCATTAATCTGCCTAATG 115
fugu GACACACCACCCCTCGGTCCAGTGTTGACAGTACATGATATTCCATTAATCTGCCTAATG 170
zfish GACACACCACCCCTCGGTCCACTGTTGACAGTACATGATATTCCATTAATCTGCCTAATG 112
frog TACAGGACATAACTCAGCTCCGTGTTGACAGAACCTGATATTCCATTAATCTGCCTAATG 121
 ** ** *** * * ********* * *************************

## Slide 9
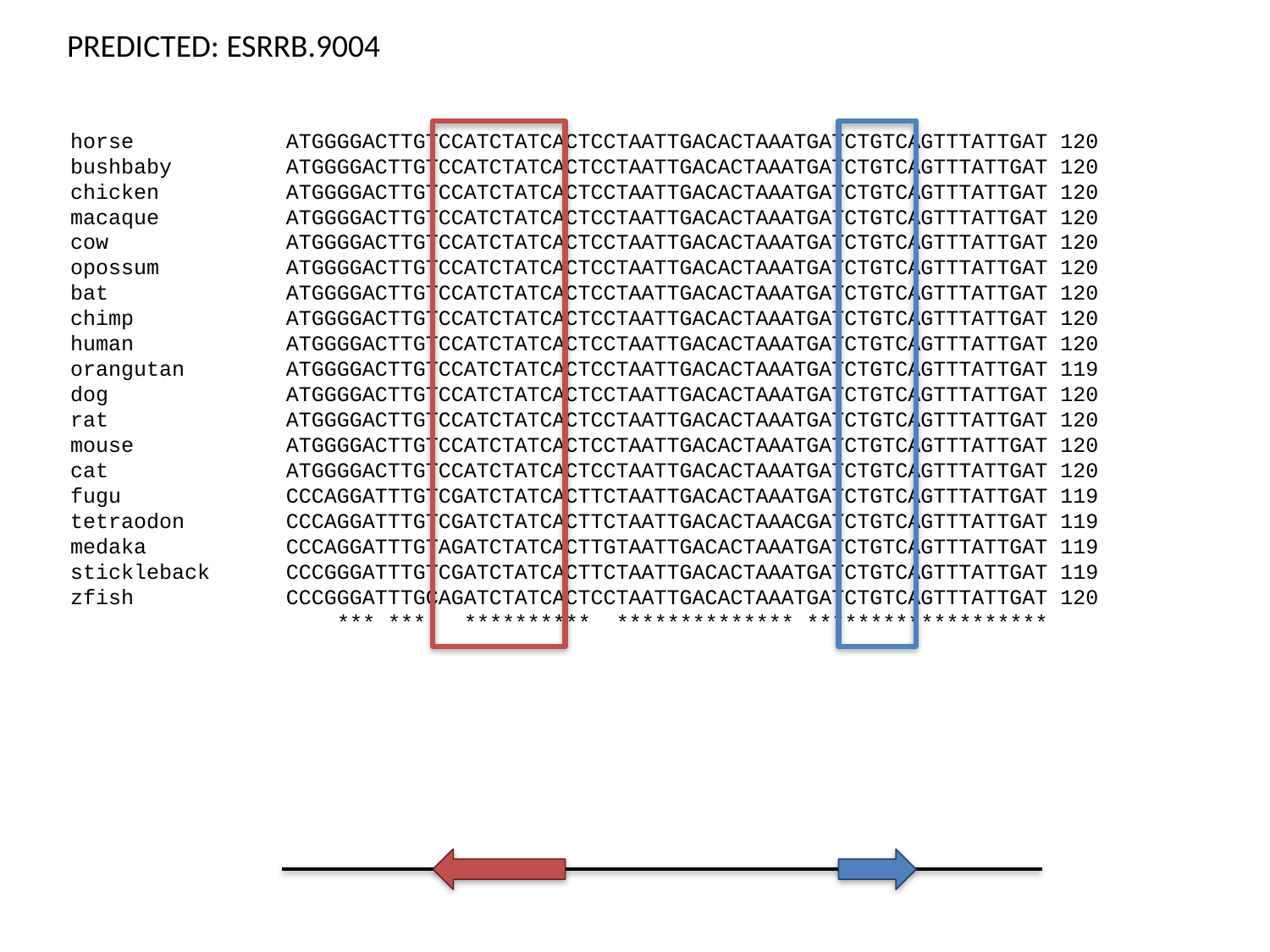

PREDICTED: ESRRB.9004
horse ATGGGGACTTGTCCATCTATCACTCCTAATTGACACTAAATGATCTGTCAGTTTATTGAT 120
bushbaby ATGGGGACTTGTCCATCTATCACTCCTAATTGACACTAAATGATCTGTCAGTTTATTGAT 120
chicken ATGGGGACTTGTCCATCTATCACTCCTAATTGACACTAAATGATCTGTCAGTTTATTGAT 120
macaque ATGGGGACTTGTCCATCTATCACTCCTAATTGACACTAAATGATCTGTCAGTTTATTGAT 120
cow ATGGGGACTTGTCCATCTATCACTCCTAATTGACACTAAATGATCTGTCAGTTTATTGAT 120
opossum ATGGGGACTTGTCCATCTATCACTCCTAATTGACACTAAATGATCTGTCAGTTTATTGAT 120
bat ATGGGGACTTGTCCATCTATCACTCCTAATTGACACTAAATGATCTGTCAGTTTATTGAT 120
chimp ATGGGGACTTGTCCATCTATCACTCCTAATTGACACTAAATGATCTGTCAGTTTATTGAT 120
human ATGGGGACTTGTCCATCTATCACTCCTAATTGACACTAAATGATCTGTCAGTTTATTGAT 120
orangutan ATGGGGACTTGTCCATCTATCACTCCTAATTGACACTAAATGATCTGTCAGTTTATTGAT 119
dog ATGGGGACTTGTCCATCTATCACTCCTAATTGACACTAAATGATCTGTCAGTTTATTGAT 120
rat ATGGGGACTTGTCCATCTATCACTCCTAATTGACACTAAATGATCTGTCAGTTTATTGAT 120
mouse ATGGGGACTTGTCCATCTATCACTCCTAATTGACACTAAATGATCTGTCAGTTTATTGAT 120
cat ATGGGGACTTGTCCATCTATCACTCCTAATTGACACTAAATGATCTGTCAGTTTATTGAT 120
fugu CCCAGGATTTGTCGATCTATCACTTCTAATTGACACTAAATGATCTGTCAGTTTATTGAT 119
tetraodon CCCAGGATTTGTCGATCTATCACTTCTAATTGACACTAAACGATCTGTCAGTTTATTGAT 119
medaka CCCAGGATTTGTAGATCTATCACTTGTAATTGACACTAAATGATCTGTCAGTTTATTGAT 119
stickleback CCCGGGATTTGTCGATCTATCACTTCTAATTGACACTAAATGATCTGTCAGTTTATTGAT 119
zfish CCCGGGATTTGCAGATCTATCACTCCTAATTGACACTAAATGATCTGTCAGTTTATTGAT 120
 *** *** ********** ************** *******************

## Slide 10
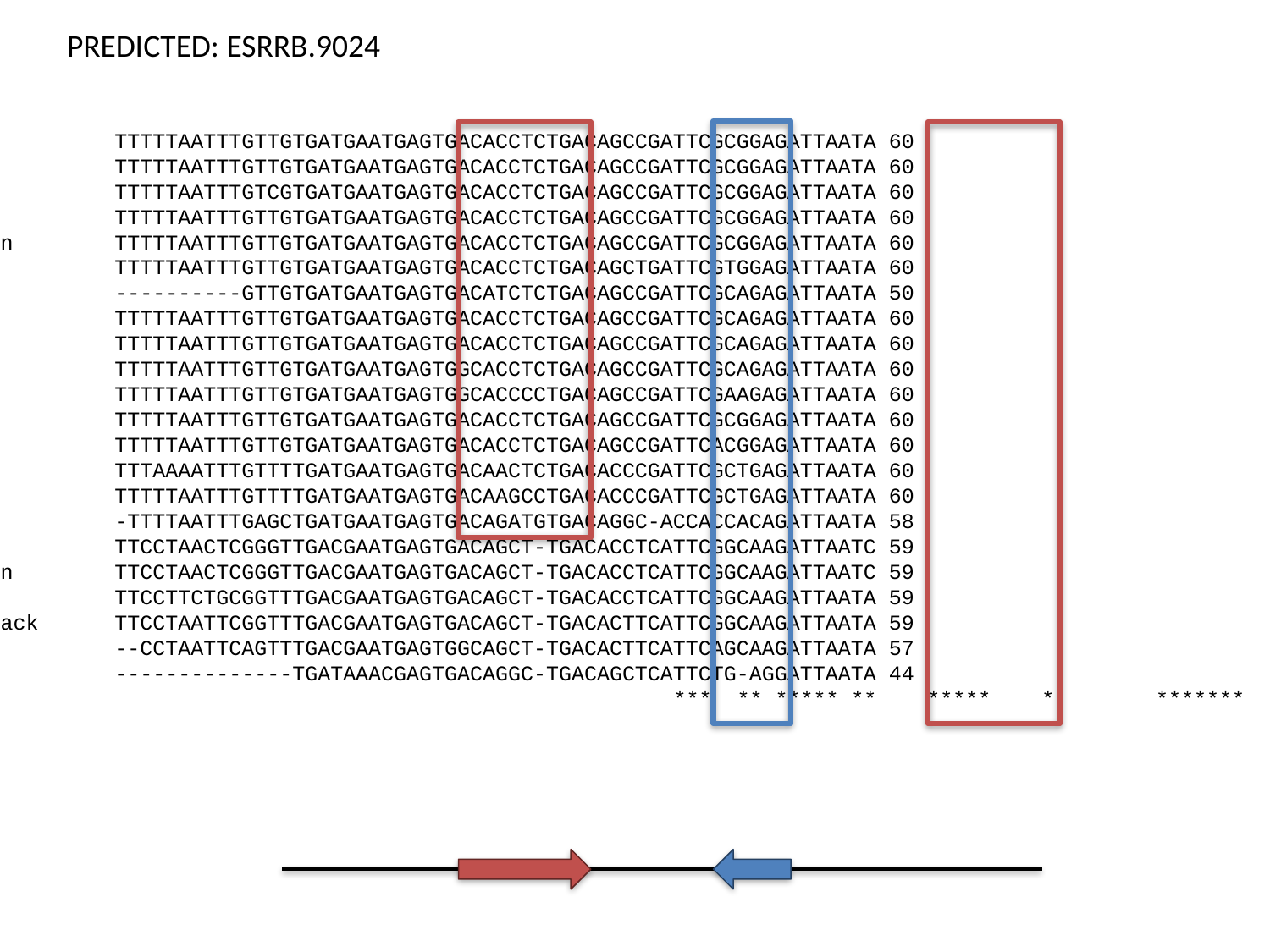

PREDICTED: ESRRB.9024
human TTTTTAATTTGTTGTGATGAATGAGTGACACCTCTGACAGCCGATTCGCGGAGATTAATA 60
chimp TTTTTAATTTGTTGTGATGAATGAGTGACACCTCTGACAGCCGATTCGCGGAGATTAATA 60
horse TTTTTAATTTGTCGTGATGAATGAGTGACACCTCTGACAGCCGATTCGCGGAGATTAATA 60
cow TTTTTAATTTGTTGTGATGAATGAGTGACACCTCTGACAGCCGATTCGCGGAGATTAATA 60
orangutan TTTTTAATTTGTTGTGATGAATGAGTGACACCTCTGACAGCCGATTCGCGGAGATTAATA 60
macaque TTTTTAATTTGTTGTGATGAATGAGTGACACCTCTGACAGCTGATTCGTGGAGATTAATA 60
bat ----------GTTGTGATGAATGAGTGACATCTCTGACAGCCGATTCGCAGAGATTAATA 50
rabbit TTTTTAATTTGTTGTGATGAATGAGTGACACCTCTGACAGCCGATTCGCAGAGATTAATA 60
squirrel TTTTTAATTTGTTGTGATGAATGAGTGACACCTCTGACAGCCGATTCGCAGAGATTAATA 60
rat TTTTTAATTTGTTGTGATGAATGAGTGGCACCTCTGACAGCCGATTCGCAGAGATTAATA 60
mouse TTTTTAATTTGTTGTGATGAATGAGTGGCACCCCTGACAGCCGATTCGAAGAGATTAATA 60
dog TTTTTAATTTGTTGTGATGAATGAGTGACACCTCTGACAGCCGATTCGCGGAGATTAATA 60
bushbaby TTTTTAATTTGTTGTGATGAATGAGTGACACCTCTGACAGCCGATTCACGGAGATTAATA 60
opossum TTTAAAATTTGTTTTGATGAATGAGTGACAACTCTGACACCCGATTCGCTGAGATTAATA 60
chicken TTTTTAATTTGTTTTGATGAATGAGTGACAAGCCTGACACCCGATTCGCTGAGATTAATA 60
frog -TTTTAATTTGAGCTGATGAATGAGTGACAGATGTGACAGGC-ACCACCACAGATTAATA 58
fugu TTCCTAACTCGGGTTGACGAATGAGTGACAGCT-TGACACCTCATTCGGCAAGATTAATC 59
tetraodon TTCCTAACTCGGGTTGACGAATGAGTGACAGCT-TGACACCTCATTCGGCAAGATTAATC 59
zfish TTCCTTCTGCGGTTTGACGAATGAGTGACAGCT-TGACACCTCATTCGGCAAGATTAATA 59
stickleback TTCCTAATTCGGTTTGACGAATGAGTGACAGCT-TGACACTTCATTCGGCAAGATTAATA 59
medaka --CCTAATTCAGTTTGACGAATGAGTGGCAGCT-TGACACTTCATTCAGCAAGATTAATA 57
shark --------------TGATAAACGAGTGACAGGC-TGACAGCTCATTCTG-AGGATTAATA 44
						 *** ** ***** ** ***** * *******

## Slide 11
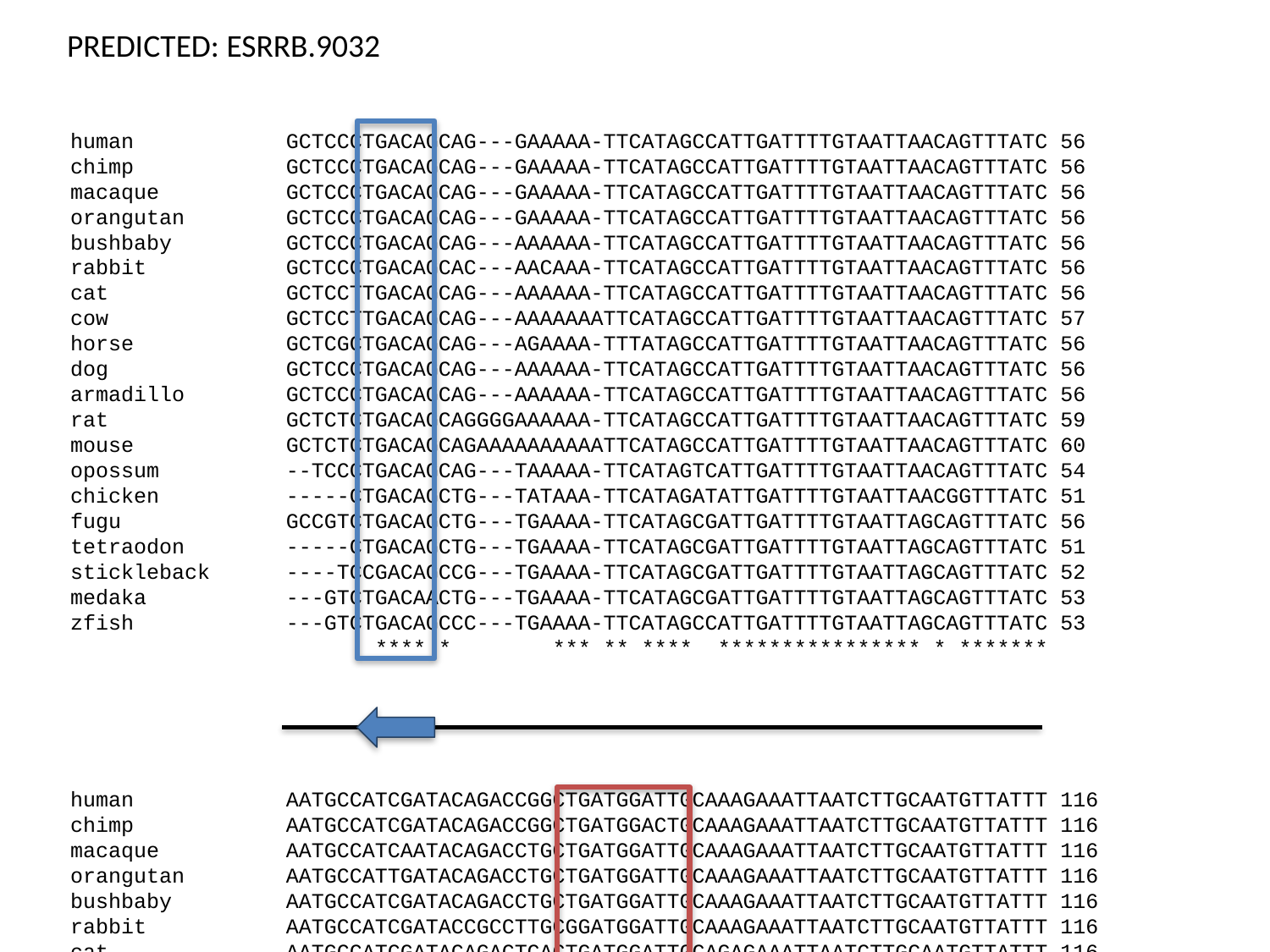

PREDICTED: ESRRB.9032
human GCTCCCTGACAGCAG---GAAAAA-TTCATAGCCATTGATTTTGTAATTAACAGTTTATC 56
chimp GCTCCCTGACAGCAG---GAAAAA-TTCATAGCCATTGATTTTGTAATTAACAGTTTATC 56
macaque GCTCCCTGACAGCAG---GAAAAA-TTCATAGCCATTGATTTTGTAATTAACAGTTTATC 56
orangutan GCTCCCTGACAGCAG---GAAAAA-TTCATAGCCATTGATTTTGTAATTAACAGTTTATC 56
bushbaby GCTCCCTGACAGCAG---AAAAAA-TTCATAGCCATTGATTTTGTAATTAACAGTTTATC 56
rabbit GCTCCCTGACAGCAC---AACAAA-TTCATAGCCATTGATTTTGTAATTAACAGTTTATC 56
cat GCTCCTTGACAGCAG---AAAAAA-TTCATAGCCATTGATTTTGTAATTAACAGTTTATC 56
cow GCTCCTTGACAGCAG---AAAAAAATTCATAGCCATTGATTTTGTAATTAACAGTTTATC 57
horse GCTCGCTGACAGCAG---AGAAAA-TTTATAGCCATTGATTTTGTAATTAACAGTTTATC 56
dog GCTCCCTGACAGCAG---AAAAAA-TTCATAGCCATTGATTTTGTAATTAACAGTTTATC 56
armadillo GCTCCCTGACAGCAG---AAAAAA-TTCATAGCCATTGATTTTGTAATTAACAGTTTATC 56
rat GCTCTCTGACAGCAGGGGAAAAAA-TTCATAGCCATTGATTTTGTAATTAACAGTTTATC 59
mouse GCTCTCTGACAGCAGAAAAAAAAAATTCATAGCCATTGATTTTGTAATTAACAGTTTATC 60
opossum --TCCCTGACAGCAG---TAAAAA-TTCATAGTCATTGATTTTGTAATTAACAGTTTATC 54
chicken -----CTGACAGCTG---TATAAA-TTCATAGATATTGATTTTGTAATTAACGGTTTATC 51
fugu GCCGTCTGACAGCTG---TGAAAA-TTCATAGCGATTGATTTTGTAATTAGCAGTTTATC 56
tetraodon -----CTGACAGCTG---TGAAAA-TTCATAGCGATTGATTTTGTAATTAGCAGTTTATC 51
stickleback ----TCCGACAGCCG---TGAAAA-TTCATAGCGATTGATTTTGTAATTAGCAGTTTATC 52
medaka ---GTCTGACAACTG---TGAAAA-TTCATAGCGATTGATTTTGTAATTAGCAGTTTATC 53
zfish ---GTCTGACAGCCC---TGAAAA-TTCATAGCCATTGATTTTGTAATTAGCAGTTTATC 53
 **** * *** ** **** **************** * *******
human AATGCCATCGATACAGACCGGCTGATGGATTGCAAAGAAATTAATCTTGCAATGTTATTT 116
chimp AATGCCATCGATACAGACCGGCTGATGGACTGCAAAGAAATTAATCTTGCAATGTTATTT 116
macaque AATGCCATCAATACAGACCTGCTGATGGATTGCAAAGAAATTAATCTTGCAATGTTATTT 116
orangutan AATGCCATTGATACAGACCTGCTGATGGATTGCAAAGAAATTAATCTTGCAATGTTATTT 116
bushbaby AATGCCATCGATACAGACCTGCTGATGGATTGCAAAGAAATTAATCTTGCAATGTTATTT 116
rabbit AATGCCATCGATACCGCCTTGCGGATGGATTGCAAAGAAATTAATCTTGCAATGTTATTT 116
cat AATGCCATCGATACAGACTCACTGATGGATTGCAGAGAAATTAATCTTGCAATGTTATTT 116
cow AATGCCATCAATACAGACTCGCTGATAGATTGCAAAGAAATTAATCTTGCAATGTTATTT 117
horse AATGCCATCGATACAGACTCACTGATGGATTGCAAAGAAATTAATCTTGCAATGTTATTT 116
dog AATGCCATCGATACAGACTCACTGATGGATTGCAAAGAAATTAATCTTGCGATGTTATTT 116
armadillo AATGCCATCGATACAGACTCGCTGATGGATTGCAAAGAAATTAATCTTGCAGTGTTATTT 116
rat AATGCCATCGATACAGACTTGCTGATGGATTGCAAAGAAATTGATCTTGCAATGTTATTT 119
mouse AATGCCATTGATACAGACTTGCTGATGGATTGCAAAGAAATTGATCTTGCAATGTTATTT 120
opossum AATGCCATCAATACAGACTTGCTGATGGATTGCAAAGAAATTAATCCTGCAAAGTTTTTT 114
chicken AATGCCATTGACACCAGCTCATGGATGGATTGAAAAGAAATTAATCCTGCAGTGTTATTT 111
fugu AATCGGATCGACGCGGGCTCACTGATGGATTGGGAGGAAATTGTTTCTTCAAAGTTGTGT 116
tetraodon AATCGGATCGACGCAGGCTCACTGATGGATTGTGAGGAAATTGTTTCTTTAAAGTTGTGT 111
stickleback AATCGGATCGACGTGGGCTCGCTGATGGATTGCGGGGAAATTGTTCCTTTAAAGTTGTTT 112
medaka AATTGGATCGACGTGAACTCACTGATGGATTGCAAGGAAATTGTTTCTTCAAAGTT---- 109
zfish AATCGTATCGACAGGCTCCTTCTGATGGATTGCAAGGAAATTGTTTCTTCAGACGTGTTT 113
 *** ** * * *** ** ** ****** * * *

## Slide 12
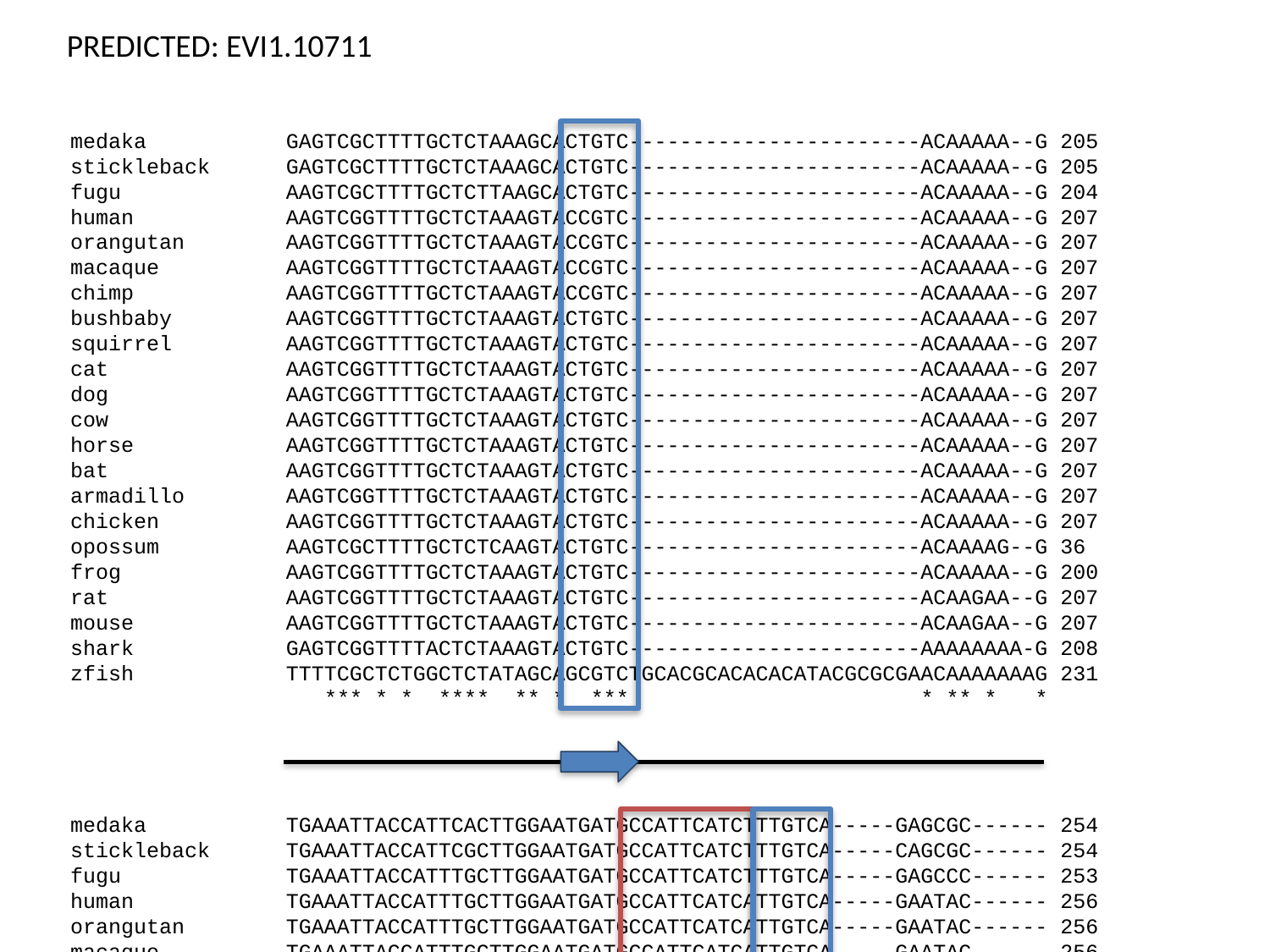

PREDICTED: EVI1.10711
medaka GAGTCGCTTTTGCTCTAAAGCACTGTC-----------------------ACAAAAA--G 205
stickleback GAGTCGCTTTTGCTCTAAAGCACTGTC-----------------------ACAAAAA--G 205
fugu AAGTCGCTTTTGCTCTTAAGCACTGTC-----------------------ACAAAAA--G 204
human AAGTCGGTTTTGCTCTAAAGTACCGTC-----------------------ACAAAAA--G 207
orangutan AAGTCGGTTTTGCTCTAAAGTACCGTC-----------------------ACAAAAA--G 207
macaque AAGTCGGTTTTGCTCTAAAGTACCGTC-----------------------ACAAAAA--G 207
chimp AAGTCGGTTTTGCTCTAAAGTACCGTC-----------------------ACAAAAA--G 207
bushbaby AAGTCGGTTTTGCTCTAAAGTACTGTC-----------------------ACAAAAA--G 207
squirrel AAGTCGGTTTTGCTCTAAAGTACTGTC-----------------------ACAAAAA--G 207
cat AAGTCGGTTTTGCTCTAAAGTACTGTC-----------------------ACAAAAA--G 207
dog AAGTCGGTTTTGCTCTAAAGTACTGTC-----------------------ACAAAAA--G 207
cow AAGTCGGTTTTGCTCTAAAGTACTGTC-----------------------ACAAAAA--G 207
horse AAGTCGGTTTTGCTCTAAAGTACTGTC-----------------------ACAAAAA--G 207
bat AAGTCGGTTTTGCTCTAAAGTACTGTC-----------------------ACAAAAA--G 207
armadillo AAGTCGGTTTTGCTCTAAAGTACTGTC-----------------------ACAAAAA--G 207
chicken AAGTCGGTTTTGCTCTAAAGTACTGTC-----------------------ACAAAAA--G 207
opossum AAGTCGCTTTTGCTCTCAAGTACTGTC-----------------------ACAAAAG--G 36
frog AAGTCGGTTTTGCTCTAAAGTACTGTC-----------------------ACAAAAA--G 200
rat AAGTCGGTTTTGCTCTAAAGTACTGTC-----------------------ACAAGAA--G 207
mouse AAGTCGGTTTTGCTCTAAAGTACTGTC-----------------------ACAAGAA--G 207
shark GAGTCGGTTTTACTCTAAAGTACTGTC-----------------------AAAAAAAA-G 208
zfish TTTTCGCTCTGGCTCTATAGCAGCGTCTGCACGCACACACATACGCGCGAACAAAAAAAG 231
 *** * * **** ** * *** * ** * *
medaka TGAAATTACCATTCACTTGGAATGATGCCATTCATCTTTGTCA-----GAGCGC------ 254
stickleback TGAAATTACCATTCGCTTGGAATGATGCCATTCATCTTTGTCA-----CAGCGC------ 254
fugu TGAAATTACCATTTGCTTGGAATGATGCCATTCATCTTTGTCA-----GAGCCC------ 253
human TGAAATTACCATTTGCTTGGAATGATGCCATTCATCATTGTCA-----GAATAC------ 256
orangutan TGAAATTACCATTTGCTTGGAATGATGCCATTCATCATTGTCA-----GAATAC------ 256
macaque TGAAATTACCATTTGCTTGGAATGATGCCATTCATCATTGTCA-----GAATAC------ 256
chimp TGAAATTACCATTTGCTTGGAATGATGCCATTCATCATTGTCA-----GAATAC------ 256
bushbaby TGAAATTACCATTCGCTTGGAATGATGCCATTCATCATTGTCA-----GAATAC------ 256
squirrel TGAAATTACCATTTGCTTGGAATGATGCCATTCATCATTGTCA-----GAATAC------ 256
cat TGAAATTACCATTTGCTTGGAATGATGCCATTCATCATTGTCA-----GAACAC------ 256
dog TGAAATTACCATTTGCTTGGAATGATGCCATTCATCATTGTCA-----GAACAC------ 256
cow TGAAATTACCATTTGCTTGGAATGATGCCATTCATCATTGTCA-----GAACAC------ 256
horse TGAAATTACCATTTGCTTGGAATGATGCCATTCATCATTGTCA-----GAACAC------ 256
bat TGAAATTACCATTTGCTTGGAATGATGCCATTCATCATTGTCA-----GAACGC------ 256
armadillo TGAAATTACCATTTGCTTGGAATGATGCCATTCATCATTGTCA-----GAATAC------ 256
chicken TGAAATTACCATTTGCTTGGAATGATGCCATTCATCATTGTCA-----GAATTC------ 256
opossum TGAAATTACCATTTGCTTGGAGGGATGCCATTCATCATTGTCA-----GAACCC------ 85
frog TGAAATTACCATTTGCTTGGAATGATGCCATTCATCATTGTCA-----GAATAC------ 249
rat TGAAATTACCATTTGCTTGGAACGATGCCATTCATCATTGTCA-----GAATGC------ 256
mouse TGAAATTACCATTTGCTTGGAACTATGCCATTCATCATTGTCA-----GAATAC------ 256
shark TGAAATTACCATTTTCTTCGAACGATGCCATTCATCATTGTCA-----GAACAC------ 257
zfish TGAAATTACTATTCAGTTGGAATGGTGCCATTCATCTTTGTCAAGTGCGGACGCGGTCCC 291
 ********* *** ** ** *********** ****** *

## Slide 13
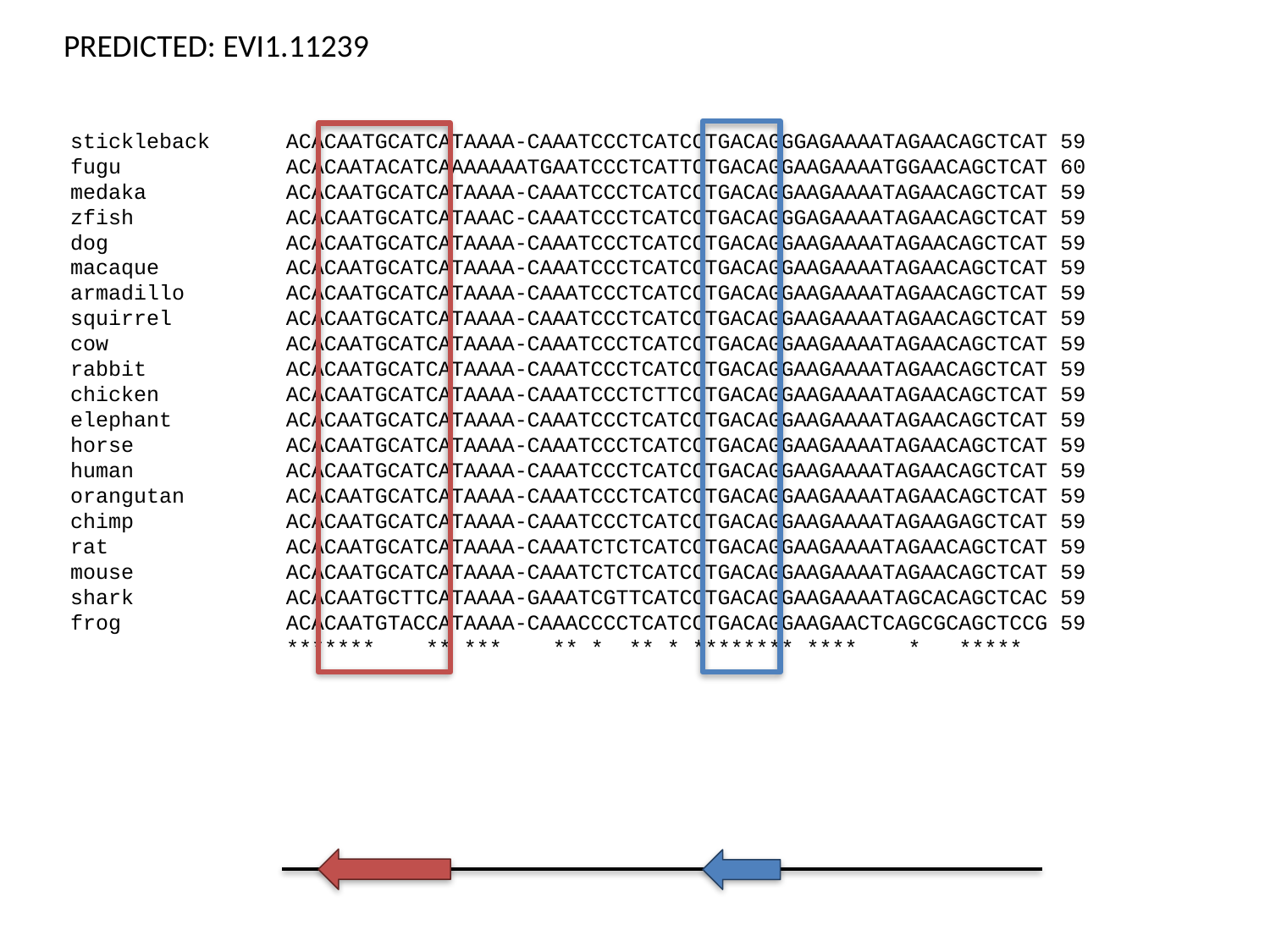

PREDICTED: EVI1.11239
stickleback ACACAATGCATCATAAAA-CAAATCCCTCATCCTGACAGGGAGAAAATAGAACAGCTCAT 59
fugu ACACAATACATCAAAAAAATGAATCCCTCATTCTGACAGGAAGAAAATGGAACAGCTCAT 60
medaka ACACAATGCATCATAAAA-CAAATCCCTCATCCTGACAGGAAGAAAATAGAACAGCTCAT 59
zfish ACACAATGCATCATAAAC-CAAATCCCTCATCCTGACAGGGAGAAAATAGAACAGCTCAT 59
dog ACACAATGCATCATAAAA-CAAATCCCTCATCCTGACAGGAAGAAAATAGAACAGCTCAT 59
macaque ACACAATGCATCATAAAA-CAAATCCCTCATCCTGACAGGAAGAAAATAGAACAGCTCAT 59
armadillo ACACAATGCATCATAAAA-CAAATCCCTCATCCTGACAGGAAGAAAATAGAACAGCTCAT 59
squirrel ACACAATGCATCATAAAA-CAAATCCCTCATCCTGACAGGAAGAAAATAGAACAGCTCAT 59
cow ACACAATGCATCATAAAA-CAAATCCCTCATCCTGACAGGAAGAAAATAGAACAGCTCAT 59
rabbit ACACAATGCATCATAAAA-CAAATCCCTCATCCTGACAGGAAGAAAATAGAACAGCTCAT 59
chicken ACACAATGCATCATAAAA-CAAATCCCTCTTCCTGACAGGAAGAAAATAGAACAGCTCAT 59
elephant ACACAATGCATCATAAAA-CAAATCCCTCATCCTGACAGGAAGAAAATAGAACAGCTCAT 59
horse ACACAATGCATCATAAAA-CAAATCCCTCATCCTGACAGGAAGAAAATAGAACAGCTCAT 59
human ACACAATGCATCATAAAA-CAAATCCCTCATCCTGACAGGAAGAAAATAGAACAGCTCAT 59
orangutan ACACAATGCATCATAAAA-CAAATCCCTCATCCTGACAGGAAGAAAATAGAACAGCTCAT 59
chimp ACACAATGCATCATAAAA-CAAATCCCTCATCCTGACAGGAAGAAAATAGAAGAGCTCAT 59
rat ACACAATGCATCATAAAA-CAAATCTCTCATCCTGACAGGAAGAAAATAGAACAGCTCAT 59
mouse ACACAATGCATCATAAAA-CAAATCTCTCATCCTGACAGGAAGAAAATAGAACAGCTCAT 59
shark ACACAATGCTTCATAAAA-GAAATCGTTCATCCTGACAGGAAGAAAATAGCACAGCTCAC 59
frog ACACAATGTACCATAAAA-CAAACCCCTCATCCTGACAGGAAGAACTCAGCGCAGCTCCG 59
 ******* ** *** ** * ** * ******** **** * *****

## Slide 14
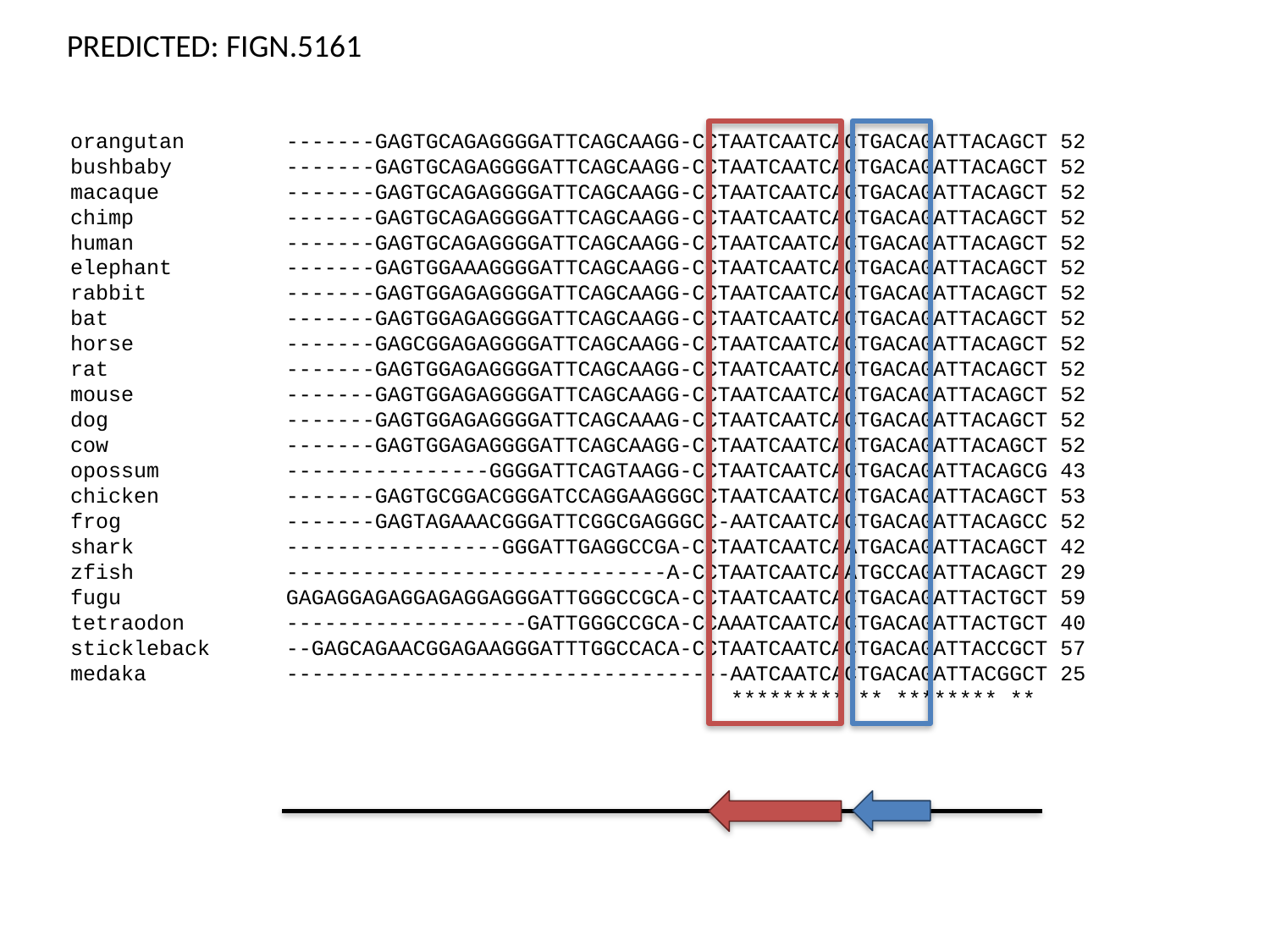

PREDICTED: FIGN.5161
orangutan -------GAGTGCAGAGGGGATTCAGCAAGG-CCTAATCAATCACTGACAGATTACAGCT 52
bushbaby -------GAGTGCAGAGGGGATTCAGCAAGG-CCTAATCAATCACTGACAGATTACAGCT 52
macaque -------GAGTGCAGAGGGGATTCAGCAAGG-CCTAATCAATCACTGACAGATTACAGCT 52
chimp -------GAGTGCAGAGGGGATTCAGCAAGG-CCTAATCAATCACTGACAGATTACAGCT 52
human -------GAGTGCAGAGGGGATTCAGCAAGG-CCTAATCAATCACTGACAGATTACAGCT 52
elephant -------GAGTGGAAAGGGGATTCAGCAAGG-CCTAATCAATCACTGACAGATTACAGCT 52
rabbit -------GAGTGGAGAGGGGATTCAGCAAGG-CCTAATCAATCACTGACAGATTACAGCT 52
bat -------GAGTGGAGAGGGGATTCAGCAAGG-CCTAATCAATCACTGACAGATTACAGCT 52
horse -------GAGCGGAGAGGGGATTCAGCAAGG-CCTAATCAATCACTGACAGATTACAGCT 52
rat -------GAGTGGAGAGGGGATTCAGCAAGG-CCTAATCAATCACTGACAGATTACAGCT 52
mouse -------GAGTGGAGAGGGGATTCAGCAAGG-CCTAATCAATCACTGACAGATTACAGCT 52
dog -------GAGTGGAGAGGGGATTCAGCAAAG-CCTAATCAATCACTGACAGATTACAGCT 52
cow -------GAGTGGAGAGGGGATTCAGCAAGG-CCTAATCAATCACTGACAGATTACAGCT 52
opossum ----------------GGGGATTCAGTAAGG-CCTAATCAATCACTGACAGATTACAGCG 43
chicken -------GAGTGCGGACGGGATCCAGGAAGGGCCTAATCAATCACTGACAGATTACAGCT 53
frog -------GAGTAGAAACGGGATTCGGCGAGGGCC-AATCAATCACTGACAGATTACAGCC 52
shark -----------------GGGATTGAGGCCGA-CCTAATCAATCAATGACAGATTACAGCT 42
zfish ------------------------------A-CCTAATCAATCAATGCCAGATTACAGCT 29
fugu GAGAGGAGAGGAGAGGAGGGATTGGGCCGCA-CCTAATCAATCACTGACAGATTACTGCT 59
tetraodon -------------------GATTGGGCCGCA-CCAAATCAATCACTGACAGATTACTGCT 40
stickleback --GAGCAGAACGGAGAAGGGATTTGGCCACA-CCTAATCAATCACTGACAGATTACCGCT 57
medaka -----------------------------------AATCAATCACTGACAGATTACGGCT 25
 ********* ** ******** **

## Slide 15
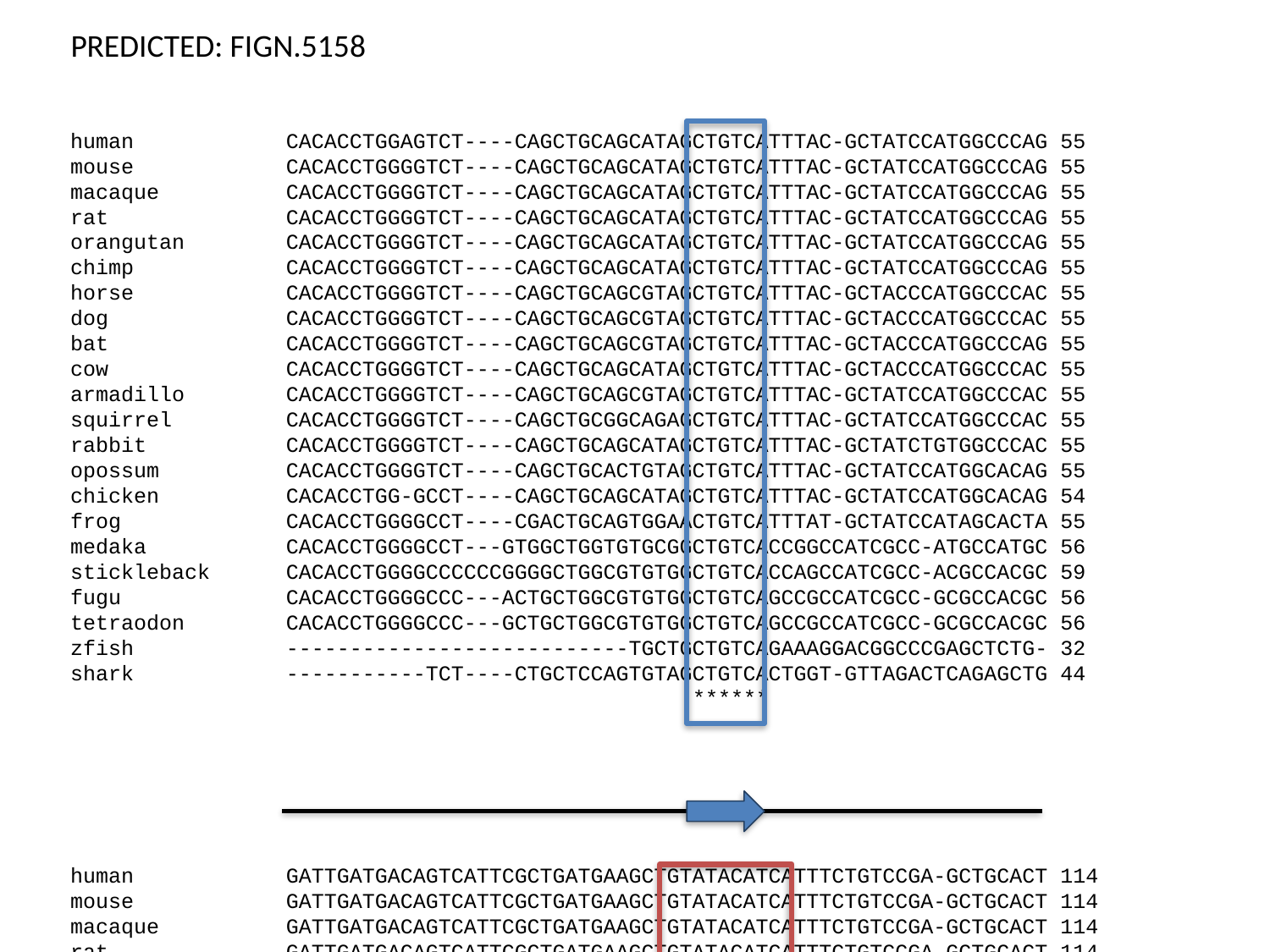

PREDICTED: FIGN.5158
human CACACCTGGAGTCT----CAGCTGCAGCATAGCTGTCATTTAC-GCTATCCATGGCCCAG 55
mouse CACACCTGGGGTCT----CAGCTGCAGCATAGCTGTCATTTAC-GCTATCCATGGCCCAG 55
macaque CACACCTGGGGTCT----CAGCTGCAGCATAGCTGTCATTTAC-GCTATCCATGGCCCAG 55
rat CACACCTGGGGTCT----CAGCTGCAGCATAGCTGTCATTTAC-GCTATCCATGGCCCAG 55
orangutan CACACCTGGGGTCT----CAGCTGCAGCATAGCTGTCATTTAC-GCTATCCATGGCCCAG 55
chimp CACACCTGGGGTCT----CAGCTGCAGCATAGCTGTCATTTAC-GCTATCCATGGCCCAG 55
horse CACACCTGGGGTCT----CAGCTGCAGCGTAGCTGTCATTTAC-GCTACCCATGGCCCAC 55
dog CACACCTGGGGTCT----CAGCTGCAGCGTAGCTGTCATTTAC-GCTACCCATGGCCCAC 55
bat CACACCTGGGGTCT----CAGCTGCAGCGTAGCTGTCATTTAC-GCTACCCATGGCCCAG 55
cow CACACCTGGGGTCT----CAGCTGCAGCATAGCTGTCATTTAC-GCTACCCATGGCCCAC 55
armadillo CACACCTGGGGTCT----CAGCTGCAGCGTAGCTGTCATTTAC-GCTATCCATGGCCCAC 55
squirrel CACACCTGGGGTCT----CAGCTGCGGCAGAGCTGTCATTTAC-GCTATCCATGGCCCAC 55
rabbit CACACCTGGGGTCT----CAGCTGCAGCATAGCTGTCATTTAC-GCTATCTGTGGCCCAC 55
opossum CACACCTGGGGTCT----CAGCTGCACTGTAGCTGTCATTTAC-GCTATCCATGGCACAG 55
chicken CACACCTGG-GCCT----CAGCTGCAGCATAGCTGTCATTTAC-GCTATCCATGGCACAG 54
frog CACACCTGGGGCCT----CGACTGCAGTGGAACTGTCATTTAT-GCTATCCATAGCACTA 55
medaka CACACCTGGGGCCT---GTGGCTGGTGTGCGGCTGTCACCGGCCATCGCC-ATGCCATGC 56
stickleback CACACCTGGGGCCCCCCGGGGCTGGCGTGTGGCTGTCACCAGCCATCGCC-ACGCCACGC 59
fugu CACACCTGGGGCCC---ACTGCTGGCGTGTGGCTGTCAGCCGCCATCGCC-GCGCCACGC 56
tetraodon CACACCTGGGGCCC---GCTGCTGGCGTGTGGCTGTCAGCCGCCATCGCC-GCGCCACGC 56
zfish ---------------------------TGCTGCTGTCAGAAAGGACGGCCCGAGCTCTG- 32
shark -----------TCT----CTGCTCCAGTGTAGCTGTCACTGGT-GTTAGACTCAGAGCTG 44
 ******
human GATTGATGACAGTCATTCGCTGATGAAGCTGTATACATCATTTCTGTCCGA-GCTGCACT 114
mouse GATTGATGACAGTCATTCGCTGATGAAGCTGTATACATCATTTCTGTCCGA-GCTGCACT 114
macaque GATTGATGACAGTCATTCGCTGATGAAGCTGTATACATCATTTCTGTCCGA-GCTGCACT 114
rat GATTGATGACAGTCATTCGCTGATGAAGCTGTATACATCATTTCTGTCCGA-GCTGCACT 114
orangutan GATTGATGACAGTCATTCGCTGATGAAGCTGTATACATCATTTCTGTCCGA-GCTGCACT 114
chimp GATTGATGACAGTCATTCGCTGATGAAGCTGTATACATCATTTCTGTCCGA-GCTGCACT 114
horse GATTGATGACAGTCATTCGCTGATGAAGCTGTATACATCATTTCTGTCCGA-GCTGCACT 114
dog GATTGATGACAGTCATTCGCTGATGAAGCTGTATACATCATTTCTGTCCGA-GCTGCACT 114
bat GATTGATGACAGTCATTCGCTGATGAAGCTGTATACATCATTTCTGTCCGA-GCTGCACT 114
cow GATTGATGACAGTCATTCGCTGATGAAGCTGTATACATCATTTCTGTCCGA-GCTGCACT 114
armadillo GATTGATGACAGTCATTCGCTGATGAAGCTGTATACATCATTTCTGTCCGA-GCTGCACT 114
squirrel GATTGATGACAGTCATTCGCTGATGAAGCTGTATACATCATTTCTGTCCGA-GCTGCACT 114
rabbit GATTGATGACAGTCATTCGCTGATGAAGCTGTATACATCATTTCTGTCCGA-GCTGCACT 114
opossum GATTGATGACAGTCATTCGCTGATGAAGCTGTATACATCATTTCTGTCCGA-GCTGCACT 114
chicken GATTGATGACAGTCATTCGCTGATGAAGCTGTATACATCATTTCTGTCCAG-GCTGCACT 113
frog GATTGATGACAGTCATTCGCTGATGAAGCTGTATACATCATTTCTGTCCAG-GCTGCATT 114
medaka GATTGATGACAGCTATTCACTGATGAAGCTGTATTCATCATTCCTGTCCAGGGCTGCACT 116
stickleback GATTGATGACAGCTATTCACTGATGAAGCTGTATTCATCATTCCTGTCCAGGGCTGCGCT 119
fugu GATTGATGACAGCTATTCACTGATGAAGCTGTATTCATCATTCCTGTCCAGGGCTGCACT 116
tetraodon GATTGATGACAGCTATTCACTGATGAAGCTGTATTCATCATTCCTGTCCAGGGCTGCACT 116
zfish GATTGATGACAGTCATTCAGTGATGAAGCTGTATTCATCATGCCTGTCCAGAGCTGCACT 92
shark AATTGATGACAGTCATTCAGTGATGAAGAAGTA--CATGGTGTCTGTGCGGGTCTGTACT 102
 *********** **** ******** *** *** * **** * *** *

## Slide 16
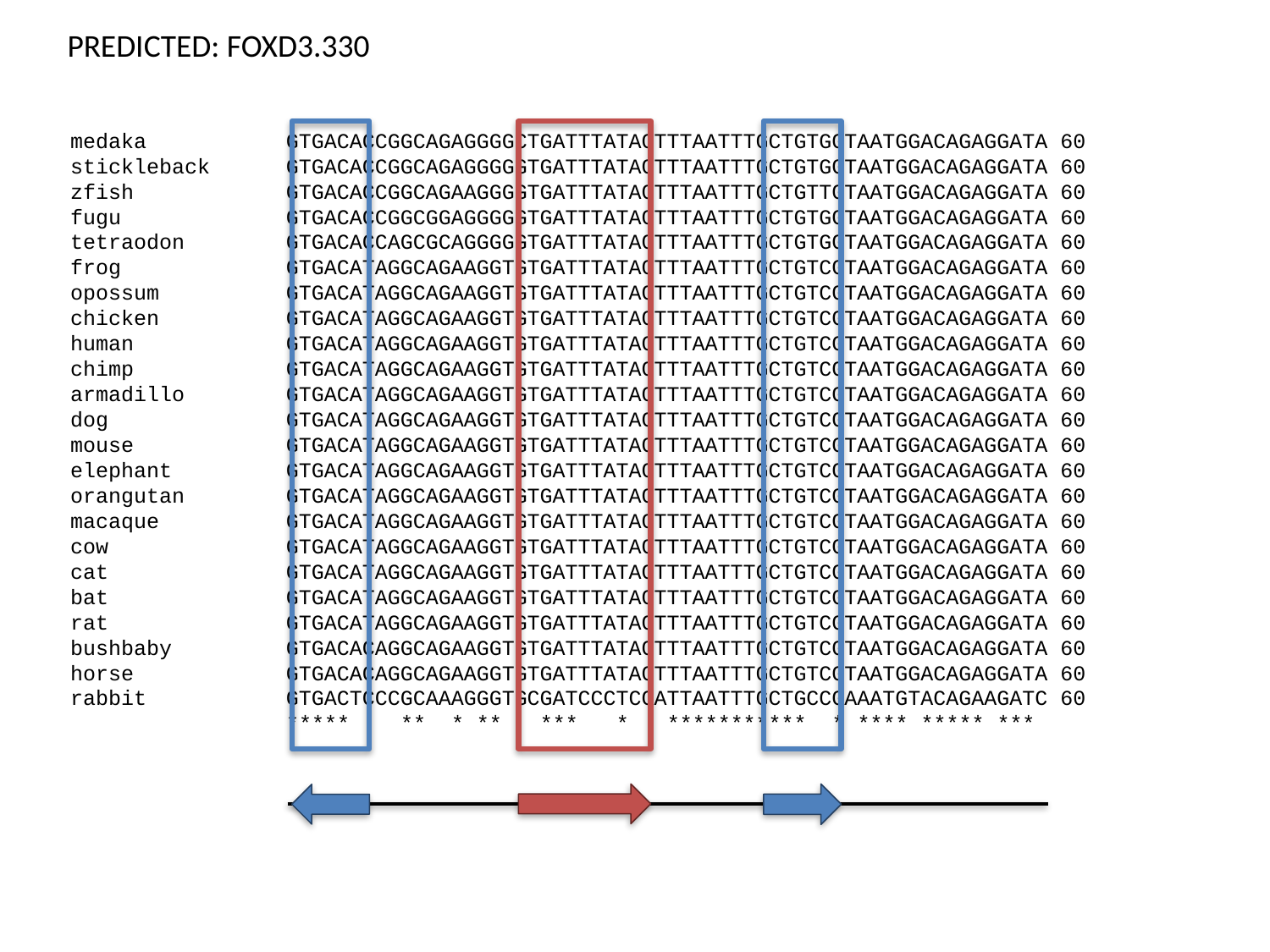

PREDICTED: FOXD3.330
medaka GTGACACCGGCAGAGGGGCTGATTTATAGTTTAATTTGCTGTGGTAATGGACAGAGGATA 60
stickleback GTGACACCGGCAGAGGGGGTGATTTATAGTTTAATTTGCTGTGGTAATGGACAGAGGATA 60
zfish GTGACACCGGCAGAAGGGGTGATTTATAGTTTAATTTGCTGTTGTAATGGACAGAGGATA 60
fugu GTGACACCGGCGGAGGGGGTGATTTATAGTTTAATTTGCTGTGGTAATGGACAGAGGATA 60
tetraodon GTGACACCAGCGCAGGGGGTGATTTATAGTTTAATTTGCTGTGGTAATGGACAGAGGATA 60
frog GTGACATAGGCAGAAGGTGTGATTTATAGTTTAATTTGCTGTCGTAATGGACAGAGGATA 60
opossum GTGACATAGGCAGAAGGTGTGATTTATAGTTTAATTTGCTGTCGTAATGGACAGAGGATA 60
chicken GTGACATAGGCAGAAGGTGTGATTTATAGTTTAATTTGCTGTCGTAATGGACAGAGGATA 60
human GTGACATAGGCAGAAGGTGTGATTTATAGTTTAATTTGCTGTCGTAATGGACAGAGGATA 60
chimp GTGACATAGGCAGAAGGTGTGATTTATAGTTTAATTTGCTGTCGTAATGGACAGAGGATA 60
armadillo GTGACATAGGCAGAAGGTGTGATTTATAGTTTAATTTGCTGTCGTAATGGACAGAGGATA 60
dog GTGACATAGGCAGAAGGTGTGATTTATAGTTTAATTTGCTGTCGTAATGGACAGAGGATA 60
mouse GTGACATAGGCAGAAGGTGTGATTTATAGTTTAATTTGCTGTCGTAATGGACAGAGGATA 60
elephant GTGACATAGGCAGAAGGTGTGATTTATAGTTTAATTTGCTGTCGTAATGGACAGAGGATA 60
orangutan GTGACATAGGCAGAAGGTGTGATTTATAGTTTAATTTGCTGTCGTAATGGACAGAGGATA 60
macaque GTGACATAGGCAGAAGGTGTGATTTATAGTTTAATTTGCTGTCGTAATGGACAGAGGATA 60
cow GTGACATAGGCAGAAGGTGTGATTTATAGTTTAATTTGCTGTCGTAATGGACAGAGGATA 60
cat GTGACATAGGCAGAAGGTGTGATTTATAGTTTAATTTGCTGTCGTAATGGACAGAGGATA 60
bat GTGACATAGGCAGAAGGTGTGATTTATAGTTTAATTTGCTGTCGTAATGGACAGAGGATA 60
rat GTGACATAGGCAGAAGGTGTGATTTATAGTTTAATTTGCTGTCGTAATGGACAGAGGATA 60
bushbaby GTGACACAGGCAGAAGGTGTGATTTATAGTTTAATTTGCTGTCGTAATGGACAGAGGATA 60
horse GTGACACAGGCAGAAGGTGTGATTTATAGTTTAATTTGCTGTCGTAATGGACAGAGGATA 60
rabbit GTGACTCCCGCAAAGGGTGCGATCCCTCCATTAATTTGCTGCCGAAATGTACAGAAGATC 60
 ***** ** * ** *** * *********** * **** ***** ***

## Slide 17
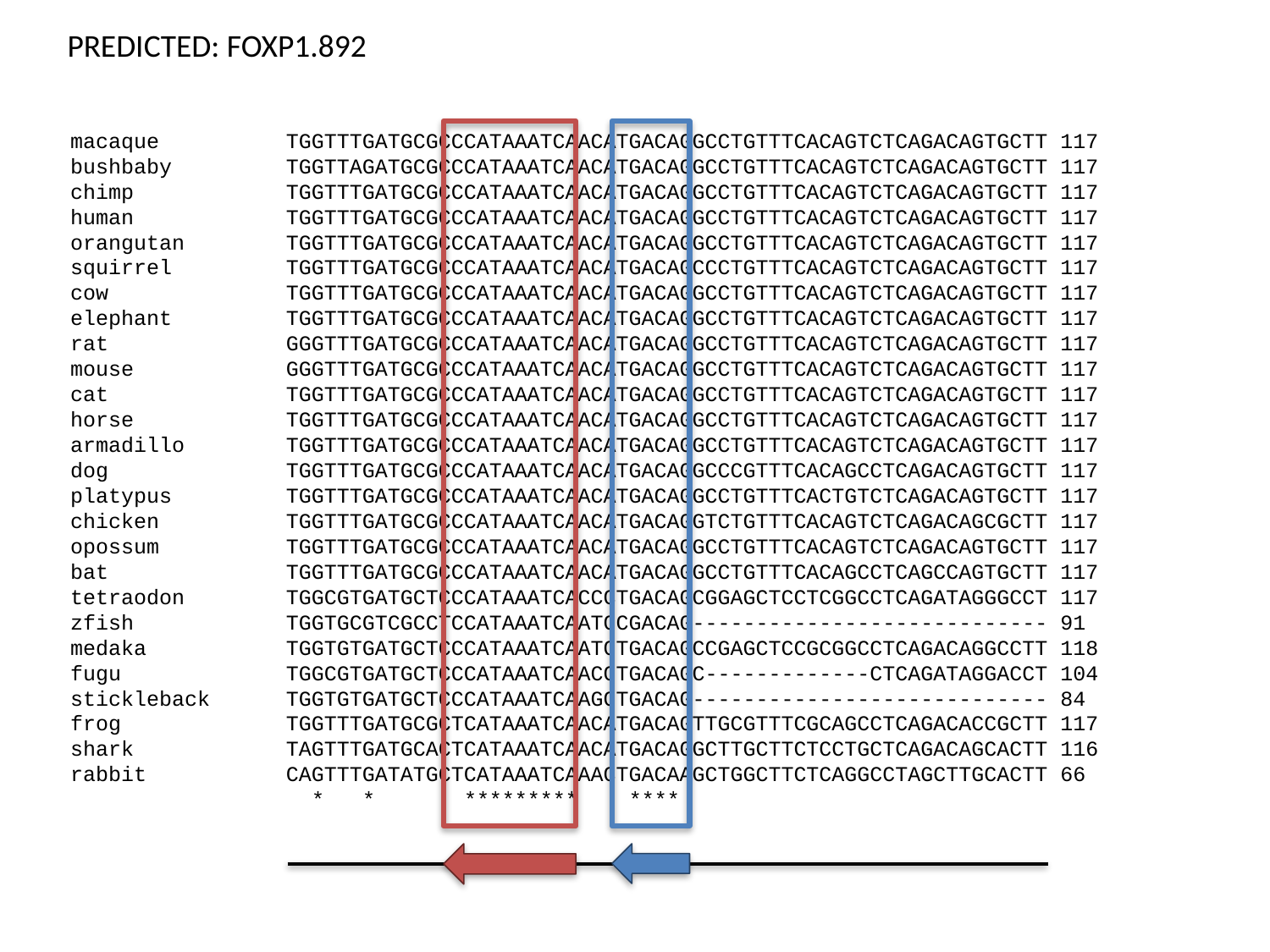

PREDICTED: FOXP1.892
macaque TGGTTTGATGCGCCCATAAATCAACATGACAGGCCTGTTTCACAGTCTCAGACAGTGCTT 117
bushbaby TGGTTAGATGCGCCCATAAATCAACATGACAGGCCTGTTTCACAGTCTCAGACAGTGCTT 117
chimp TGGTTTGATGCGCCCATAAATCAACATGACAGGCCTGTTTCACAGTCTCAGACAGTGCTT 117
human TGGTTTGATGCGCCCATAAATCAACATGACAGGCCTGTTTCACAGTCTCAGACAGTGCTT 117
orangutan TGGTTTGATGCGCCCATAAATCAACATGACAGGCCTGTTTCACAGTCTCAGACAGTGCTT 117
squirrel TGGTTTGATGCGCCCATAAATCAACATGACAGCCCTGTTTCACAGTCTCAGACAGTGCTT 117
cow TGGTTTGATGCGCCCATAAATCAACATGACAGGCCTGTTTCACAGTCTCAGACAGTGCTT 117
elephant TGGTTTGATGCGCCCATAAATCAACATGACAGGCCTGTTTCACAGTCTCAGACAGTGCTT 117
rat GGGTTTGATGCGCCCATAAATCAACATGACAGGCCTGTTTCACAGTCTCAGACAGTGCTT 117
mouse GGGTTTGATGCGCCCATAAATCAACATGACAGGCCTGTTTCACAGTCTCAGACAGTGCTT 117
cat TGGTTTGATGCGCCCATAAATCAACATGACAGGCCTGTTTCACAGTCTCAGACAGTGCTT 117
horse TGGTTTGATGCGCCCATAAATCAACATGACAGGCCTGTTTCACAGTCTCAGACAGTGCTT 117
armadillo TGGTTTGATGCGCCCATAAATCAACATGACAGGCCTGTTTCACAGTCTCAGACAGTGCTT 117
dog TGGTTTGATGCGCCCATAAATCAACATGACAGGCCCGTTTCACAGCCTCAGACAGTGCTT 117
platypus TGGTTTGATGCGCCCATAAATCAACATGACAGGCCTGTTTCACTGTCTCAGACAGTGCTT 117
chicken TGGTTTGATGCGCCCATAAATCAACATGACAGGTCTGTTTCACAGTCTCAGACAGCGCTT 117
opossum TGGTTTGATGCGCCCATAAATCAACATGACAGGCCTGTTTCACAGTCTCAGACAGTGCTT 117
bat TGGTTTGATGCGCCCATAAATCAACATGACAGGCCTGTTTCACAGCCTCAGCCAGTGCTT 117
tetraodon TGGCGTGATGCTCCCATAAATCACCCTGACAGCGGAGCTCCTCGGCCTCAGATAGGGCCT 117
zfish TGGTGCGTCGCCTCCATAAATCAATGCGACAG---------------------------- 91
medaka TGGTGTGATGCTCCCATAAATCAATGTGACAGCCGAGCTCCGCGGCCTCAGACAGGCCTT 118
fugu TGGCGTGATGCTCCCATAAATCAACGTGACAGC-------------CTCAGATAGGACCT 104
stickleback TGGTGTGATGCTCCCATAAATCAAGGTGACAG---------------------------- 84
frog TGGTTTGATGCGCTCATAAATCAACATGACAGTTGCGTTTCGCAGCCTCAGACACCGCTT 117
shark TAGTTTGATGCACTCATAAATCAACATGACAGGCTTGCTTCTCCTGCTCAGACAGCACTT 116
rabbit CAGTTTGATATGCTCATAAATCAAACTGACAAGCTGGCTTCTCAGGCCTAGCTTGCACTT 66
 * * ********* ****

## Slide 18
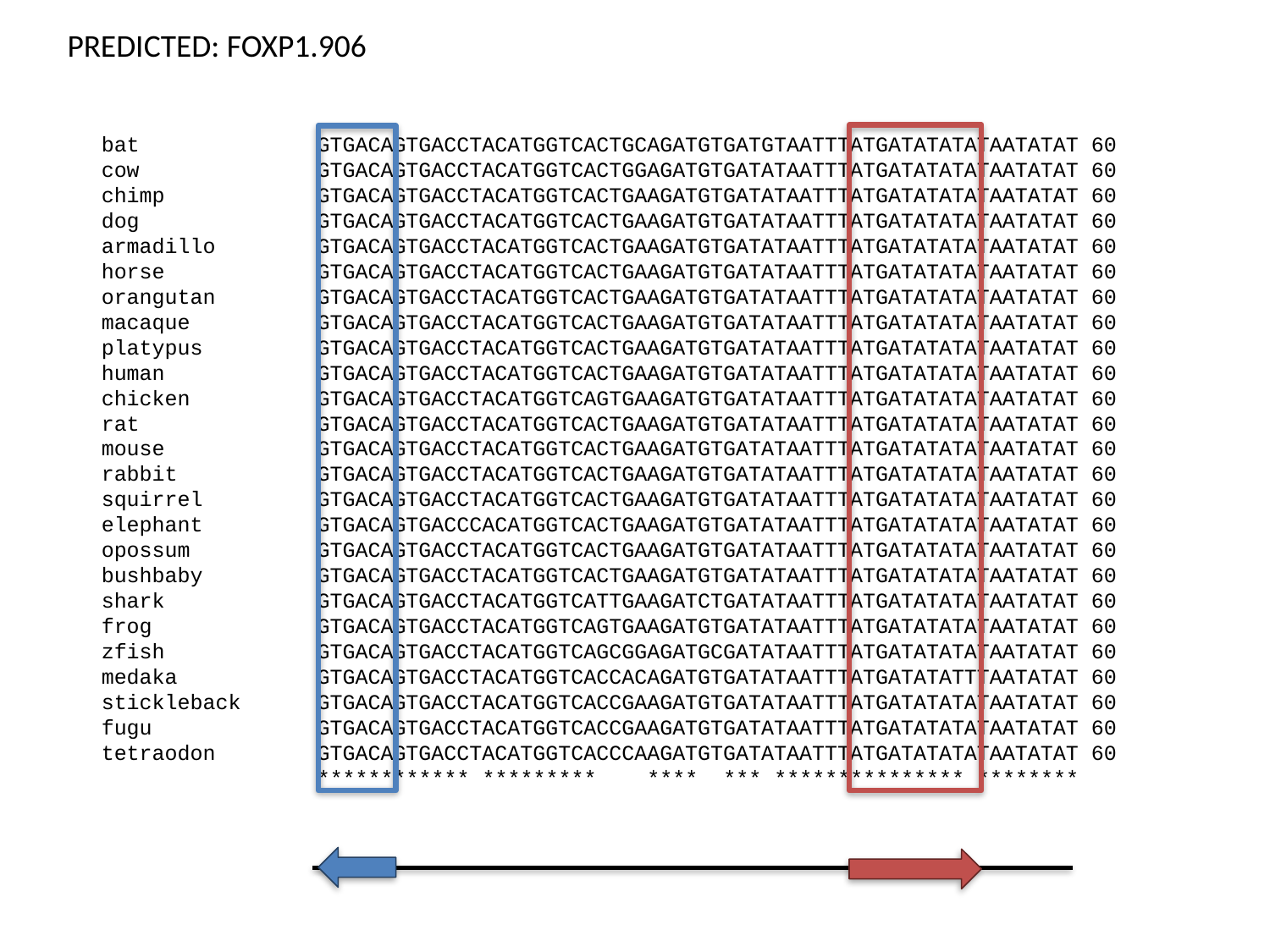

PREDICTED: FOXP1.906
bat GTGACAGTGACCTACATGGTCACTGCAGATGTGATGTAATTTATGATATATATAATATAT 60
cow GTGACAGTGACCTACATGGTCACTGGAGATGTGATATAATTTATGATATATATAATATAT 60
chimp GTGACAGTGACCTACATGGTCACTGAAGATGTGATATAATTTATGATATATATAATATAT 60
dog GTGACAGTGACCTACATGGTCACTGAAGATGTGATATAATTTATGATATATATAATATAT 60
armadillo GTGACAGTGACCTACATGGTCACTGAAGATGTGATATAATTTATGATATATATAATATAT 60
horse GTGACAGTGACCTACATGGTCACTGAAGATGTGATATAATTTATGATATATATAATATAT 60
orangutan GTGACAGTGACCTACATGGTCACTGAAGATGTGATATAATTTATGATATATATAATATAT 60
macaque GTGACAGTGACCTACATGGTCACTGAAGATGTGATATAATTTATGATATATATAATATAT 60
platypus GTGACAGTGACCTACATGGTCACTGAAGATGTGATATAATTTATGATATATATAATATAT 60
human GTGACAGTGACCTACATGGTCACTGAAGATGTGATATAATTTATGATATATATAATATAT 60
chicken GTGACAGTGACCTACATGGTCAGTGAAGATGTGATATAATTTATGATATATATAATATAT 60
rat GTGACAGTGACCTACATGGTCACTGAAGATGTGATATAATTTATGATATATATAATATAT 60
mouse GTGACAGTGACCTACATGGTCACTGAAGATGTGATATAATTTATGATATATATAATATAT 60
rabbit GTGACAGTGACCTACATGGTCACTGAAGATGTGATATAATTTATGATATATATAATATAT 60
squirrel GTGACAGTGACCTACATGGTCACTGAAGATGTGATATAATTTATGATATATATAATATAT 60
elephant GTGACAGTGACCCACATGGTCACTGAAGATGTGATATAATTTATGATATATATAATATAT 60
opossum GTGACAGTGACCTACATGGTCACTGAAGATGTGATATAATTTATGATATATATAATATAT 60
bushbaby GTGACAGTGACCTACATGGTCACTGAAGATGTGATATAATTTATGATATATATAATATAT 60
shark GTGACAGTGACCTACATGGTCATTGAAGATCTGATATAATTTATGATATATATAATATAT 60
frog GTGACAGTGACCTACATGGTCAGTGAAGATGTGATATAATTTATGATATATATAATATAT 60
zfish GTGACAGTGACCTACATGGTCAGCGGAGATGCGATATAATTTATGATATATATAATATAT 60
medaka GTGACAGTGACCTACATGGTCACCACAGATGTGATATAATTTATGATATATTTAATATAT 60
stickleback GTGACAGTGACCTACATGGTCACCGAAGATGTGATATAATTTATGATATATATAATATAT 60
fugu GTGACAGTGACCTACATGGTCACCGAAGATGTGATATAATTTATGATATATATAATATAT 60
tetraodon GTGACAGTGACCTACATGGTCACCCAAGATGTGATATAATTTATGATATATATAATATAT 60
 ************ ********* **** *** *************** ********

## Slide 19
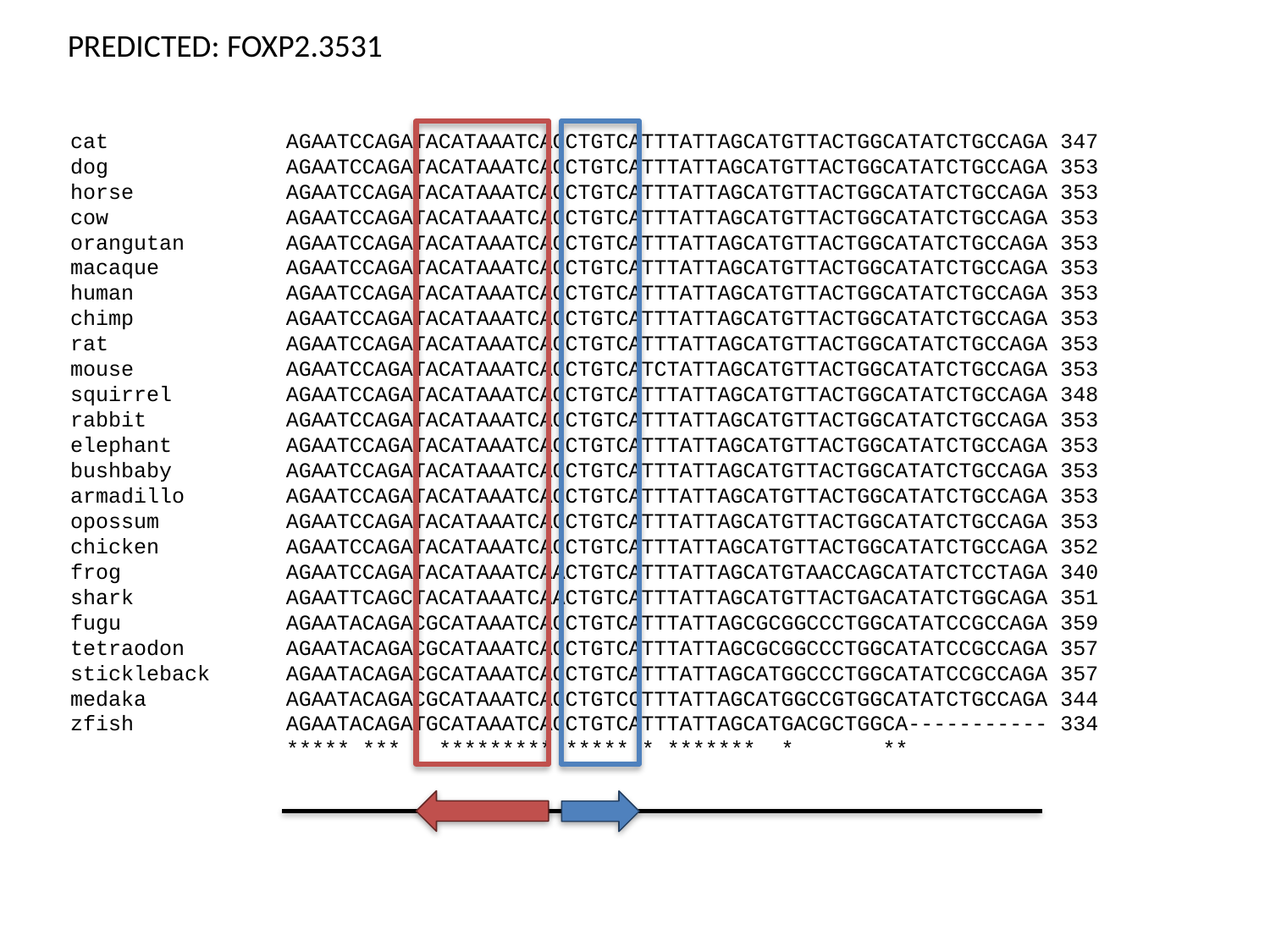

PREDICTED: FOXP2.3531
cat AGAATCCAGATACATAAATCAGCTGTCATTTATTAGCATGTTACTGGCATATCTGCCAGA 347
dog AGAATCCAGATACATAAATCAGCTGTCATTTATTAGCATGTTACTGGCATATCTGCCAGA 353
horse AGAATCCAGATACATAAATCAGCTGTCATTTATTAGCATGTTACTGGCATATCTGCCAGA 353
cow AGAATCCAGATACATAAATCAGCTGTCATTTATTAGCATGTTACTGGCATATCTGCCAGA 353
orangutan AGAATCCAGATACATAAATCAGCTGTCATTTATTAGCATGTTACTGGCATATCTGCCAGA 353
macaque AGAATCCAGATACATAAATCAGCTGTCATTTATTAGCATGTTACTGGCATATCTGCCAGA 353
human AGAATCCAGATACATAAATCAGCTGTCATTTATTAGCATGTTACTGGCATATCTGCCAGA 353
chimp AGAATCCAGATACATAAATCAGCTGTCATTTATTAGCATGTTACTGGCATATCTGCCAGA 353
rat AGAATCCAGATACATAAATCAGCTGTCATTTATTAGCATGTTACTGGCATATCTGCCAGA 353
mouse AGAATCCAGATACATAAATCAGCTGTCATCTATTAGCATGTTACTGGCATATCTGCCAGA 353
squirrel AGAATCCAGATACATAAATCAGCTGTCATTTATTAGCATGTTACTGGCATATCTGCCAGA 348
rabbit AGAATCCAGATACATAAATCAGCTGTCATTTATTAGCATGTTACTGGCATATCTGCCAGA 353
elephant AGAATCCAGATACATAAATCAGCTGTCATTTATTAGCATGTTACTGGCATATCTGCCAGA 353
bushbaby AGAATCCAGATACATAAATCAGCTGTCATTTATTAGCATGTTACTGGCATATCTGCCAGA 353
armadillo AGAATCCAGATACATAAATCAGCTGTCATTTATTAGCATGTTACTGGCATATCTGCCAGA 353
opossum AGAATCCAGATACATAAATCAGCTGTCATTTATTAGCATGTTACTGGCATATCTGCCAGA 353
chicken AGAATCCAGATACATAAATCAGCTGTCATTTATTAGCATGTTACTGGCATATCTGCCAGA 352
frog AGAATCCAGATACATAAATCAACTGTCATTTATTAGCATGTAACCAGCATATCTCCTAGA 340
shark AGAATTCAGCTACATAAATCAACTGTCATTTATTAGCATGTTACTGACATATCTGGCAGA 351
fugu AGAATACAGACGCATAAATCAGCTGTCATTTATTAGCGCGGCCCTGGCATATCCGCCAGA 359
tetraodon AGAATACAGACGCATAAATCAGCTGTCATTTATTAGCGCGGCCCTGGCATATCCGCCAGA 357
stickleback AGAATACAGACGCATAAATCAGCTGTCATTTATTAGCATGGCCCTGGCATATCCGCCAGA 357
medaka AGAATACAGACGCATAAATCAGCTGTCCTTTATTAGCATGGCCGTGGCATATCTGCCAGA 344
zfish AGAATACAGATGCATAAATCAGCTGTCATTTATTAGCATGACGCTGGCA----------- 334
 ***** *** ********* ***** * ******* * **

## Slide 20
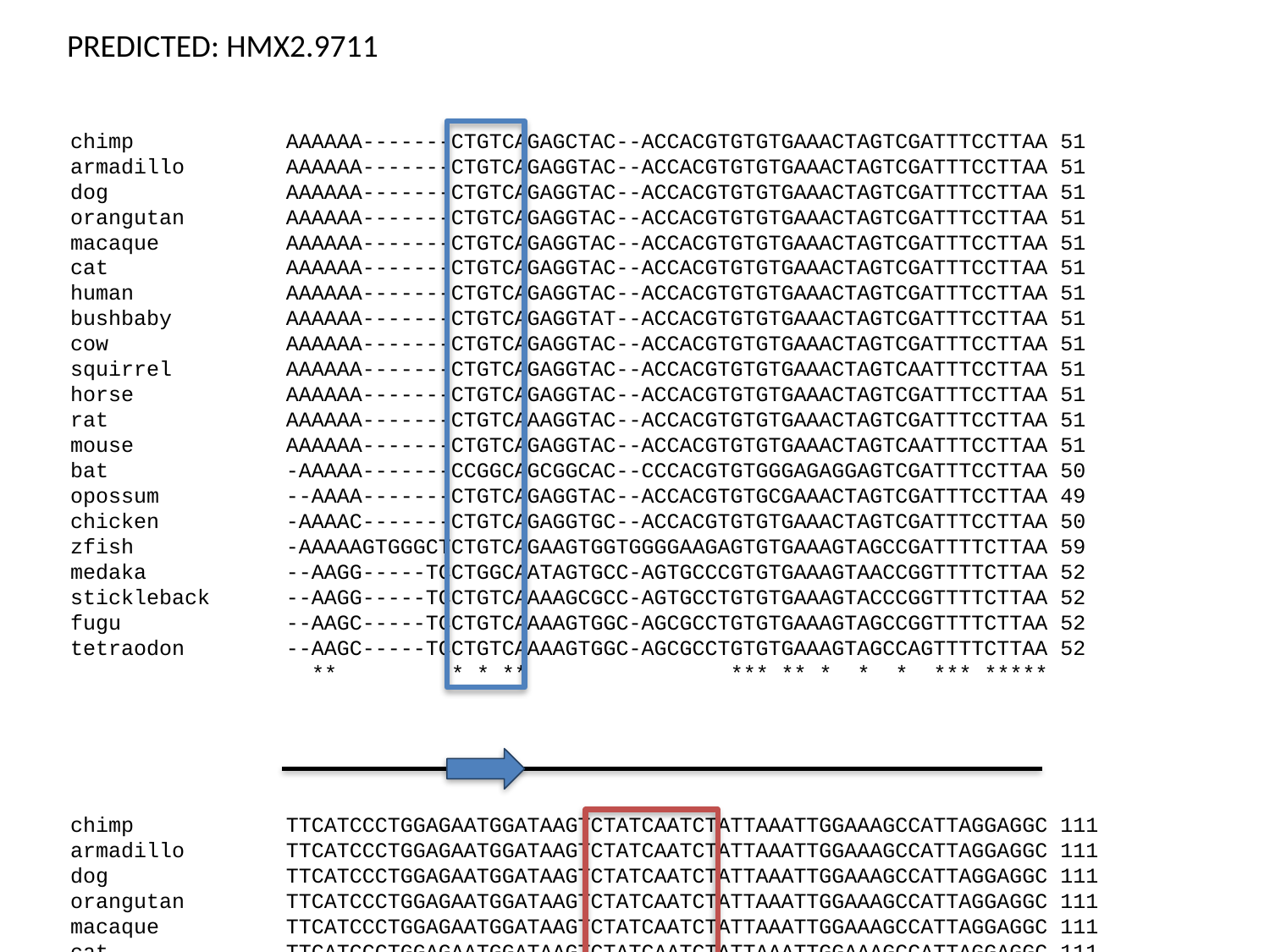

PREDICTED: HMX2.9711
chimp AAAAAA-------CTGTCAGAGCTAC--ACCACGTGTGTGAAACTAGTCGATTTCCTTAA 51
armadillo AAAAAA-------CTGTCAGAGGTAC--ACCACGTGTGTGAAACTAGTCGATTTCCTTAA 51
dog AAAAAA-------CTGTCAGAGGTAC--ACCACGTGTGTGAAACTAGTCGATTTCCTTAA 51
orangutan AAAAAA-------CTGTCAGAGGTAC--ACCACGTGTGTGAAACTAGTCGATTTCCTTAA 51
macaque AAAAAA-------CTGTCAGAGGTAC--ACCACGTGTGTGAAACTAGTCGATTTCCTTAA 51
cat AAAAAA-------CTGTCAGAGGTAC--ACCACGTGTGTGAAACTAGTCGATTTCCTTAA 51
human AAAAAA-------CTGTCAGAGGTAC--ACCACGTGTGTGAAACTAGTCGATTTCCTTAA 51
bushbaby AAAAAA-------CTGTCAGAGGTAT--ACCACGTGTGTGAAACTAGTCGATTTCCTTAA 51
cow AAAAAA-------CTGTCAGAGGTAC--ACCACGTGTGTGAAACTAGTCGATTTCCTTAA 51
squirrel AAAAAA-------CTGTCAGAGGTAC--ACCACGTGTGTGAAACTAGTCAATTTCCTTAA 51
horse AAAAAA-------CTGTCAGAGGTAC--ACCACGTGTGTGAAACTAGTCGATTTCCTTAA 51
rat AAAAAA-------CTGTCAAAGGTAC--ACCACGTGTGTGAAACTAGTCGATTTCCTTAA 51
mouse AAAAAA-------CTGTCAGAGGTAC--ACCACGTGTGTGAAACTAGTCAATTTCCTTAA 51
bat -AAAAA-------CCGGCAGCGGCAC--CCCACGTGTGGGAGAGGAGTCGATTTCCTTAA 50
opossum --AAAA-------CTGTCAGAGGTAC--ACCACGTGTGCGAAACTAGTCGATTTCCTTAA 49
chicken -AAAAC-------CTGTCAGAGGTGC--ACCACGTGTGTGAAACTAGTCGATTTCCTTAA 50
zfish -AAAAAGTGGGCTCTGTCAGAAGTGGTGGGGAAGAGTGTGAAAGTAGCCGATTTTCTTAA 59
medaka --AAGG-----TCCTGGCAATAGTGCC-AGTGCCCGTGTGAAAGTAACCGGTTTTCTTAA 52
stickleback --AAGG-----TCCTGTCAAAAGCGCC-AGTGCCTGTGTGAAAGTACCCGGTTTTCTTAA 52
fugu --AAGC-----TCCTGTCAAAAGTGGC-AGCGCCTGTGTGAAAGTAGCCGGTTTTCTTAA 52
tetraodon --AAGC-----TCCTGTCAAAAGTGGC-AGCGCCTGTGTGAAAGTAGCCAGTTTTCTTAA 52
 ** * * ** *** ** * * * *** *****
chimp TTCATCCCTGGAGAATGGATAAGTCTATCAATCTATTAAATTGGAAAGCCATTAGGAGGC 111
armadillo TTCATCCCTGGAGAATGGATAAGTCTATCAATCTATTAAATTGGAAAGCCATTAGGAGGC 111
dog TTCATCCCTGGAGAATGGATAAGTCTATCAATCTATTAAATTGGAAAGCCATTAGGAGGC 111
orangutan TTCATCCCTGGAGAATGGATAAGTCTATCAATCTATTAAATTGGAAAGCCATTAGGAGGC 111
macaque TTCATCCCTGGAGAATGGATAAGTCTATCAATCTATTAAATTGGAAAGCCATTAGGAGGC 111
cat TTCATCCCTGGAGAATGGATAAGTCTATCAATCTATTAAATTGGAAAGCCATTAGGAGGC 111
human TTCATCCCTGGAGAATGGATAAGTCTATCAATCTATTAAATTGGAAAGCCATTAGGAGGC 111
bushbaby TTCATCCCTGGAGAATGGATAAGTCTATCAATCTATTAAATTGGAAAGCCATTAGGAGGC 111
cow TTCATCCCTGGAGAATGGATAAGTCTATCAATCTATTAAATTGGAAAGCCATTAGGAGGC 111
squirrel TTCATCCCAGGAGAATGGATAAGTCTATCAATCTATTAAATTGGAAAGCCATTAGGAGGC 111
horse TTCATCCCTGGAGAATGGATAAGTCTATCAATCTATTAAATTGGAAAGCCATTAGGAGGC 111
rat TTCATCCCTGGAGAATGGATAAGTCTATCAATCTATTAAATTGGAAAGCCATTAGGAGGC 111
mouse TTCATCCCAGGAGAATGGATAAGTCTATCAATCTATTAAATTGGAAAGCCATTAGGAGGC 111
bat TTCATCCCTGGAGAATGGATAAGTCTATCAATCTATTAAATTGGAAAGCCATTAGGAGGC 110
opossum TTCATCCCTGGAGAATGGATAAGTCTATCAATCTATTAAATTGGAAAGCCATTAGGAGGC 109
chicken TTCATCCCTTGAGAATGGATAAGTCTATCAATCTATTAAATTGGAAAGCCATTAGGAGGC 110
zfish TACATCCCCTGGGAGCAGATAAGTCTATCAATCTGTTAGATTGGAAAGCCATTAGGTGCC 119
medaka TTCATCCCTCAGGACCGGATAAGTCTATCAATCTGTTAAATTGGAAAGCCATTAGGTGCC 112
stickleback TTCATCCCTTAGGACCGGATAAGTCTATCAATCTGTTAAATTGGAAAGCCATTAGGTGCC 112
fugu TTCATCCCCCAGGACCGGATAAGTCTATCAATCTGTTAGATTGGAAAGCCATTAGGTGCC 112
tetraodon TTCATCCCCCAGGACCGGATAAGTCTATCAATCTGTTAAATTGGAAAGCCATTAGGCGCC 112
 * ****** ** ***************** *** ***************** * *

## Slide 21
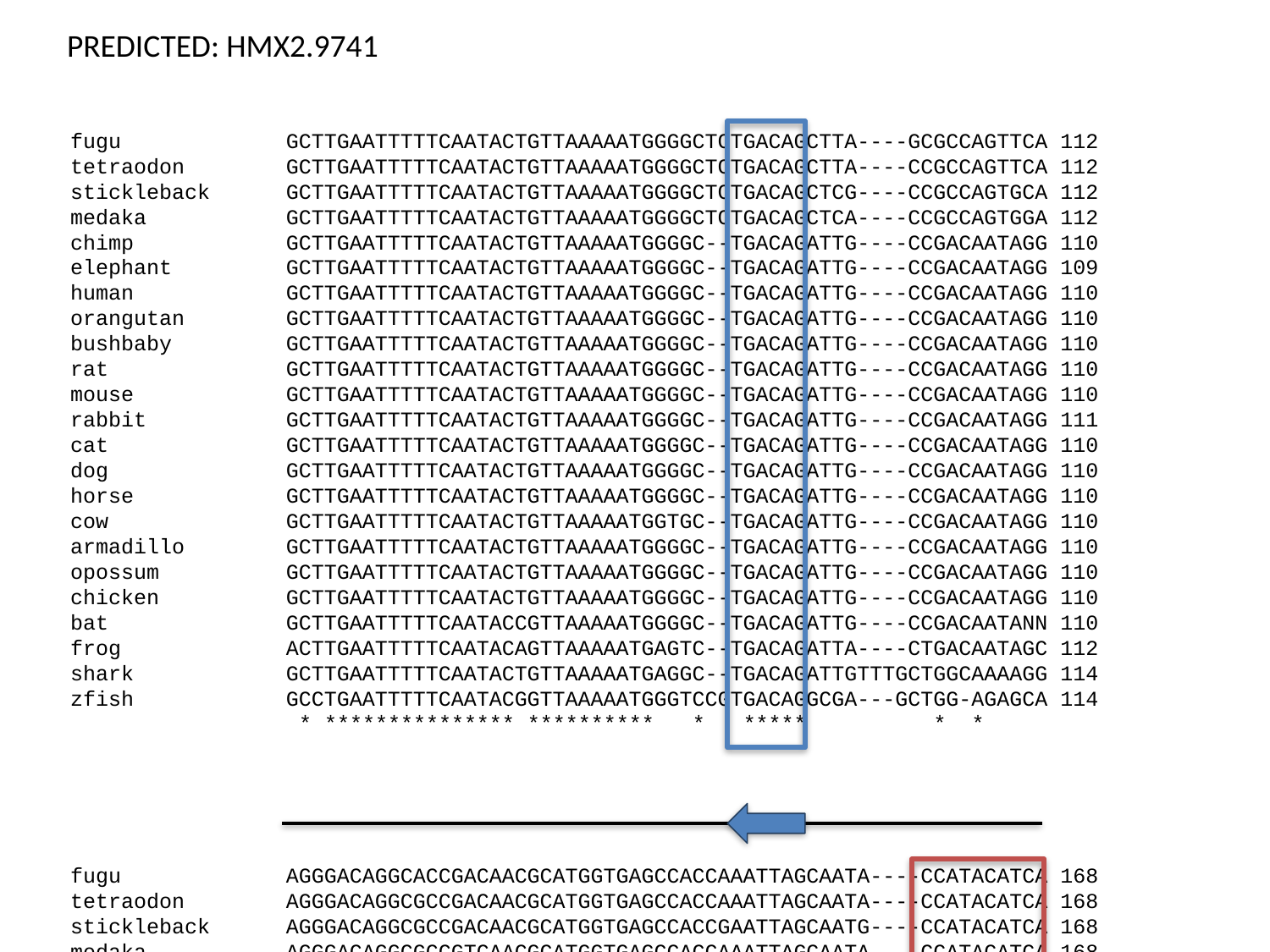

PREDICTED: HMX2.9741
fugu GCTTGAATTTTTCAATACTGTTAAAAATGGGGCTCTGACAGCTTA----GCGCCAGTTCA 112
tetraodon GCTTGAATTTTTCAATACTGTTAAAAATGGGGCTCTGACAGCTTA----CCGCCAGTTCA 112
stickleback GCTTGAATTTTTCAATACTGTTAAAAATGGGGCTCTGACAGCTCG----CCGCCAGTGCA 112
medaka GCTTGAATTTTTCAATACTGTTAAAAATGGGGCTCTGACAGCTCA----CCGCCAGTGGA 112
chimp GCTTGAATTTTTCAATACTGTTAAAAATGGGGC--TGACAGATTG----CCGACAATAGG 110
elephant GCTTGAATTTTTCAATACTGTTAAAAATGGGGC--TGACAGATTG----CCGACAATAGG 109
human GCTTGAATTTTTCAATACTGTTAAAAATGGGGC--TGACAGATTG----CCGACAATAGG 110
orangutan GCTTGAATTTTTCAATACTGTTAAAAATGGGGC--TGACAGATTG----CCGACAATAGG 110
bushbaby GCTTGAATTTTTCAATACTGTTAAAAATGGGGC--TGACAGATTG----CCGACAATAGG 110
rat GCTTGAATTTTTCAATACTGTTAAAAATGGGGC--TGACAGATTG----CCGACAATAGG 110
mouse GCTTGAATTTTTCAATACTGTTAAAAATGGGGC--TGACAGATTG----CCGACAATAGG 110
rabbit GCTTGAATTTTTCAATACTGTTAAAAATGGGGC--TGACAGATTG----CCGACAATAGG 111
cat GCTTGAATTTTTCAATACTGTTAAAAATGGGGC--TGACAGATTG----CCGACAATAGG 110
dog GCTTGAATTTTTCAATACTGTTAAAAATGGGGC--TGACAGATTG----CCGACAATAGG 110
horse GCTTGAATTTTTCAATACTGTTAAAAATGGGGC--TGACAGATTG----CCGACAATAGG 110
cow GCTTGAATTTTTCAATACTGTTAAAAATGGTGC--TGACAGATTG----CCGACAATAGG 110
armadillo GCTTGAATTTTTCAATACTGTTAAAAATGGGGC--TGACAGATTG----CCGACAATAGG 110
opossum GCTTGAATTTTTCAATACTGTTAAAAATGGGGC--TGACAGATTG----CCGACAATAGG 110
chicken GCTTGAATTTTTCAATACTGTTAAAAATGGGGC--TGACAGATTG----CCGACAATAGG 110
bat GCTTGAATTTTTCAATACCGTTAAAAATGGGGC--TGACAGATTG----CCGACAATANN 110
frog ACTTGAATTTTTCAATACAGTTAAAAATGAGTC--TGACAGATTA----CTGACAATAGC 112
shark GCTTGAATTTTTCAATACTGTTAAAAATGAGGC--TGACAGATTGTTTGCTGGCAAAAGG 114
zfish GCCTGAATTTTTCAATACGGTTAAAAATGGGTCCGTGACAGGCGA---GCTGG-AGAGCA 114
 * *************** ********** * ***** * *
fugu AGGGACAGGCACCGACAACGCATGGTGAGCCACCAAATTAGCAATA----CCATACATCA 168
tetraodon AGGGACAGGCGCCGACAACGCATGGTGAGCCACCAAATTAGCAATA----CCATACATCA 168
stickleback AGGGACAGGCGCCGACAACGCATGGTGAGCCACCGAATTAGCAATG----CCATACATCA 168
medaka AGGGACAGGCGCCGTCAACGCATGGTGAGCCACCAAATTAGCAATA----CCATACATCA 168
chimp AGCAACGGGCCCAGACGCTGCGTCCGGAGCCGCTGAATTAGCAATA----CCATATATCA 166
elephant AGCAACGGGCCCAGACGCGGCGTCCGGAGCCGCTGAATTAGCCATA----CCATATATCA 165
human AGCAACGGGCCCAGACGCTGCGTCCGGAGCCGCTGAATTAGCAATA----CCATATATCA 166
orangutan AGCAACGGGCCCAGACGCTGCGTCCGGAGCCGCTGAATTAGCAATA----CCATATATCA 166
bushbaby AGCAACGGGCCCAGACGCTGCGTCCGGAGCCACTGAATTAGCAATA----CCATATATCA 166
rat AGCAACGGGCCCAGAC--TGCGTCCGGAGCCGCTGAATTAGCAATA----CCATATATCA 164
mouse AGCAACGGGCCCAGAC--TGCGTCCGGAGCTGCTGGATTAGCAATA----CCATATATCA 164
rabbit AG-AAGGGGCCAAGTC--TGCGTCCGGAGCCGCTGAATTAGCAATA----CCATATATCA 164
cat GGCACCGGGCCCAGGCACTGCGTTCGGAGCCGCTGAATTAGCAATC----CCATATATCA 166
dog G-TAACGGGCCCAGGCACTCCGTCCGGAGCCGCTGAATTAGCAGTA----CCATATATCA 165
horse GGCAACGGGCTCAGGCACTGCGTCCGGAGCCGCTGAATTAGCAATA----CCATATATCA 166
cow GACAACGGGCCCAGGCAAGGCGTCCGGAGTCGCTGAATTAGCAATACCATCCATATATCA 170
armadillo AACCACGGGCCC-------GCGTCCGGAGCTGCCGAATTAGCACTA----CCATATATCA 159
opossum AGCCGCGG-CCTAGACACTGCGTATGAAGCCGCTGAATTAGCAATA----CCATATATCA 165
chicken AGCAGCGG-CCTAGACACTGCAAAGAGAGCCACTGAATTAGCAATA----CCATATATCA 165
bat NNNNNNNNNNNNNNNN--NNNNNNNNNNNNNNNNNAACCAGCAACG----CCATCTATCA 164
frog AGTTCTAG-CTGAGACACAGGGAGCAGAAGCACT--ATTAACCATA----TCATATATCA 165
shark AGGA--AGGCTCA-------TAAACAGGAACTTGGAATTAGTGGTA----GCATACATCA 161
zfish AAGGGCCGCCGTAGACAGCGCACAGAGGGCCACCGAATTAGCAATA----CCATACATCA 170
 * * *** ****

## Slide 22
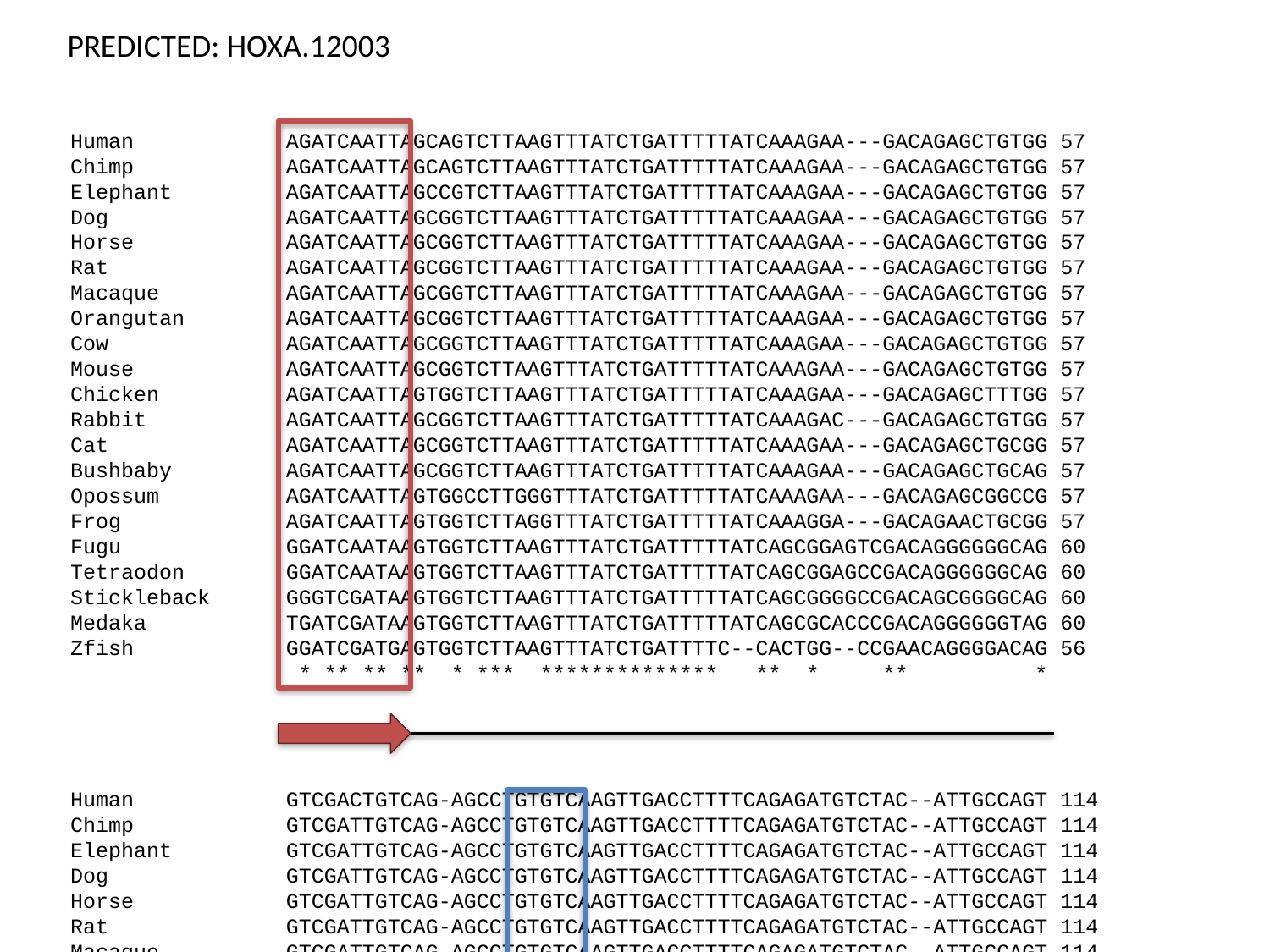

PREDICTED: HOXA.12003
Human AGATCAATTAGCAGTCTTAAGTTTATCTGATTTTTATCAAAGAA---GACAGAGCTGTGG 57
Chimp AGATCAATTAGCAGTCTTAAGTTTATCTGATTTTTATCAAAGAA---GACAGAGCTGTGG 57
Elephant AGATCAATTAGCCGTCTTAAGTTTATCTGATTTTTATCAAAGAA---GACAGAGCTGTGG 57
Dog AGATCAATTAGCGGTCTTAAGTTTATCTGATTTTTATCAAAGAA---GACAGAGCTGTGG 57
Horse AGATCAATTAGCGGTCTTAAGTTTATCTGATTTTTATCAAAGAA---GACAGAGCTGTGG 57
Rat AGATCAATTAGCGGTCTTAAGTTTATCTGATTTTTATCAAAGAA---GACAGAGCTGTGG 57
Macaque AGATCAATTAGCGGTCTTAAGTTTATCTGATTTTTATCAAAGAA---GACAGAGCTGTGG 57
Orangutan AGATCAATTAGCGGTCTTAAGTTTATCTGATTTTTATCAAAGAA---GACAGAGCTGTGG 57
Cow AGATCAATTAGCGGTCTTAAGTTTATCTGATTTTTATCAAAGAA---GACAGAGCTGTGG 57
Mouse AGATCAATTAGCGGTCTTAAGTTTATCTGATTTTTATCAAAGAA---GACAGAGCTGTGG 57
Chicken AGATCAATTAGTGGTCTTAAGTTTATCTGATTTTTATCAAAGAA---GACAGAGCTTTGG 57
Rabbit AGATCAATTAGCGGTCTTAAGTTTATCTGATTTTTATCAAAGAC---GACAGAGCTGTGG 57
Cat AGATCAATTAGCGGTCTTAAGTTTATCTGATTTTTATCAAAGAA---GACAGAGCTGCGG 57
Bushbaby AGATCAATTAGCGGTCTTAAGTTTATCTGATTTTTATCAAAGAA---GACAGAGCTGCAG 57
Opossum AGATCAATTAGTGGCCTTGGGTTTATCTGATTTTTATCAAAGAA---GACAGAGCGGCCG 57
Frog AGATCAATTAGTGGTCTTAGGTTTATCTGATTTTTATCAAAGGA---GACAGAACTGCGG 57
Fugu GGATCAATAAGTGGTCTTAAGTTTATCTGATTTTTATCAGCGGAGTCGACAGGGGGGCAG 60
Tetraodon GGATCAATAAGTGGTCTTAAGTTTATCTGATTTTTATCAGCGGAGCCGACAGGGGGGCAG 60
Stickleback GGGTCGATAAGTGGTCTTAAGTTTATCTGATTTTTATCAGCGGGGCCGACAGCGGGGCAG 60
Medaka TGATCGATAAGTGGTCTTAAGTTTATCTGATTTTTATCAGCGCACCCGACAGGGGGGTAG 60
Zfish GGATCGATGAGTGGTCTTAAGTTTATCTGATTTTC--CACTGG--CCGAACAGGGGACAG 56
 * ** ** ** * *** ************** ** * ** *
Human GTCGACTGTCAG-AGCCTGTGTCAAGTTGACCTTTTCAGAGATGTCTAC--ATTGCCAGT 114
Chimp GTCGATTGTCAG-AGCCTGTGTCAAGTTGACCTTTTCAGAGATGTCTAC--ATTGCCAGT 114
Elephant GTCGATTGTCAG-AGCCTGTGTCAAGTTGACCTTTTCAGAGATGTCTAC--ATTGCCAGT 114
Dog GTCGATTGTCAG-AGCCTGTGTCAAGTTGACCTTTTCAGAGATGTCTAC--ATTGCCAGT 114
Horse GTCGATTGTCAG-AGCCTGTGTCAAGTTGACCTTTTCAGAGATGTCTAC--ATTGCCAGT 114
Rat GTCGATTGTCAG-AGCCTGTGTCAAGTTGACCTTTTCAGAGATGTCTAC--ATTGCCAGT 114
Macaque GTCGATTGTCAG-AGCCTGTGTCAAGTTGACCTTTTCAGAGATGTCTAC--ATTGCCAGT 114
Orangutan GTCGATTGTCAG-AGCCTGTGTCAAGTTGACCTTTTCAGAGATGTCTAC--ATTGCCAGT 114
Cow GTCGATTGTCAG-AGCCTGTGTCAAGTTGACCTTTTCAGAGATGTCTAC--ATTGCTAGT 114
Mouse GTCGATTGTCAG-AGCCTGTGTCAAGTTGACCTTTTCAGAGATGTCTAC--ATTGCCAGT 114
Chicken GTCGATTGTCAG-AGCCTGTGTCAAGTTGACCTTTTCAGAGATGTCTAC--ATTGCCAGT 114
Rabbit GTCGATTGTCAG-AGCCTGTGTCAAGTTGACCTTTTCAGAGATGTCTAC--ATTGCCAGT 114
Cat GTCGATTGTCAG-AGCCTGTGTCAAGTTGACCTTTTCAGAGATGTCTAC--ATTGCCAGT 114
Bushbaby GTCGATTGTCAG-AGCCTGTGTCAAGTTGACCTTTTCAGAGATGTCTAC--ATTGCCAGT 114
Opossum GTCGATCGTCAG-AGCCTGTGTCAAGCTGACCTTTTCAGAGACGTCTAC--ATCGGCCGT 114
Frog GTCGATTGGCAG-AGCCTGTGTCAAGTTGACCTTTTCAGAGACGTCTAC--ATTGCCAGT 114
Fugu GTCGATGGGCTG-TGCCCGCGTCAAGTTGACCTTTTCAGGGACGACGG--GGAGAGCTCT 117
Tetraodon GTCGATGGGCCG-TGCCCGCGTCAAGTTGACCTTTTCAGGGACGACGG--GGAGAGCTCT 117
Stickleback GTCGATGGGCTG-TGCCCGCGTCAAGTTGACCTTTTCAGGGACGCCGGCGTGAGAGCTCC 119
Medaka GTCAATGTGCTG-TGCCCGCGTCAAGTTGACCTTTTCAGGGACGTTGGTGAGAGAGCCCC 119
Zfish GTCGATGGGACGCTGCCTGTGTCAAGTTGACCTTTTCAGGG-CATAGGC-GGCCAGTTGT 114
 *** * * *** * ****** ************ *

## Slide 23
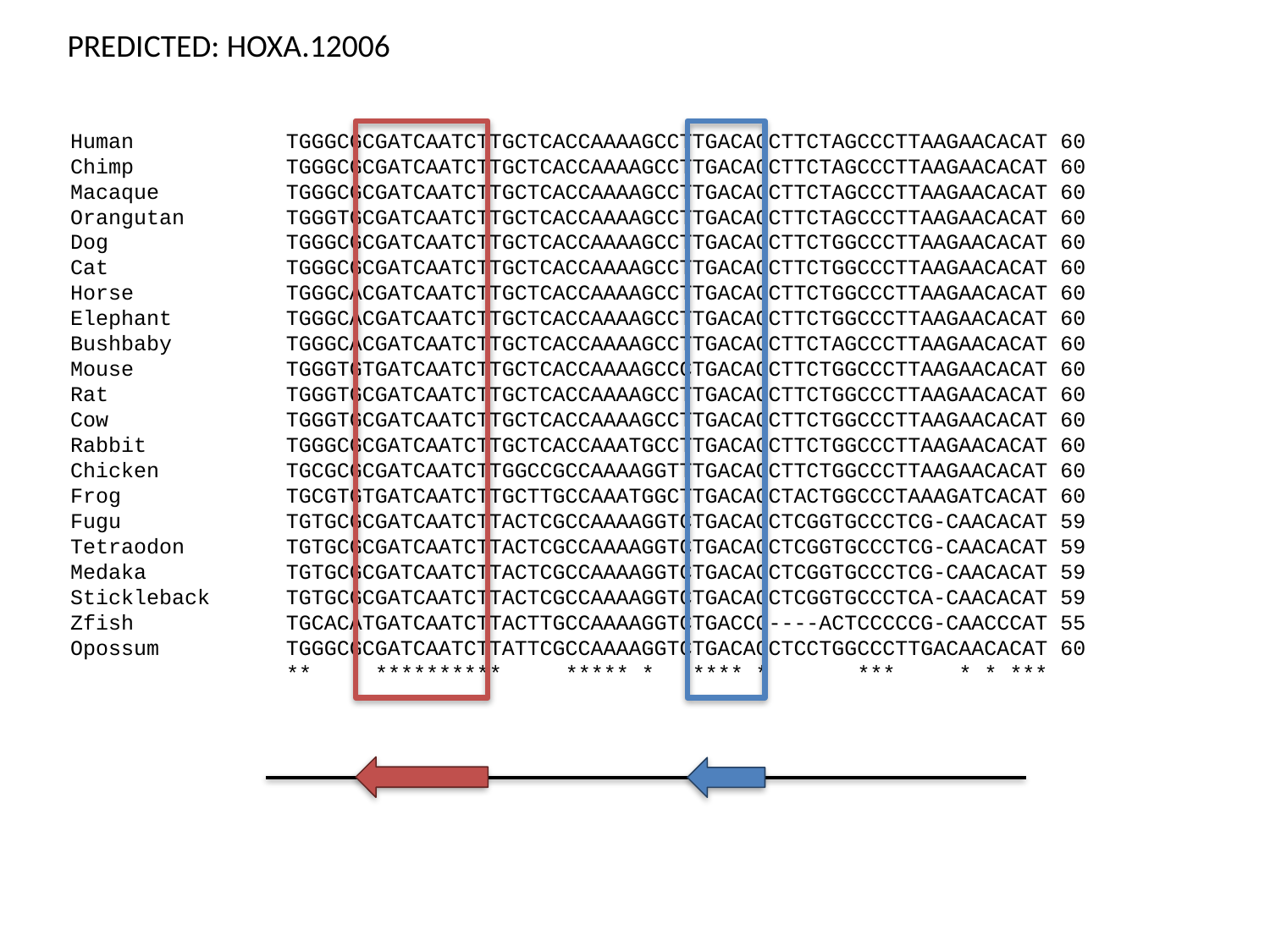

PREDICTED: HOXA.12006
Human TGGGCGCGATCAATCTTGCTCACCAAAAGCCTTGACAGCTTCTAGCCCTTAAGAACACAT 60
Chimp TGGGCGCGATCAATCTTGCTCACCAAAAGCCTTGACAGCTTCTAGCCCTTAAGAACACAT 60
Macaque TGGGCGCGATCAATCTTGCTCACCAAAAGCCTTGACAGCTTCTAGCCCTTAAGAACACAT 60
Orangutan TGGGTGCGATCAATCTTGCTCACCAAAAGCCTTGACAGCTTCTAGCCCTTAAGAACACAT 60
Dog TGGGCGCGATCAATCTTGCTCACCAAAAGCCTTGACAGCTTCTGGCCCTTAAGAACACAT 60
Cat TGGGCGCGATCAATCTTGCTCACCAAAAGCCTTGACAGCTTCTGGCCCTTAAGAACACAT 60
Horse TGGGCACGATCAATCTTGCTCACCAAAAGCCTTGACAGCTTCTGGCCCTTAAGAACACAT 60
Elephant TGGGCACGATCAATCTTGCTCACCAAAAGCCTTGACAGCTTCTGGCCCTTAAGAACACAT 60
Bushbaby TGGGCACGATCAATCTTGCTCACCAAAAGCCTTGACAGCTTCTAGCCCTTAAGAACACAT 60
Mouse TGGGTGTGATCAATCTTGCTCACCAAAAGCCCTGACAGCTTCTGGCCCTTAAGAACACAT 60
Rat TGGGTGCGATCAATCTTGCTCACCAAAAGCCTTGACAGCTTCTGGCCCTTAAGAACACAT 60
Cow TGGGTGCGATCAATCTTGCTCACCAAAAGCCTTGACAGCTTCTGGCCCTTAAGAACACAT 60
Rabbit TGGGCGCGATCAATCTTGCTCACCAAATGCCTTGACAGCTTCTGGCCCTTAAGAACACAT 60
Chicken TGCGCGCGATCAATCTTGGCCGCCAAAAGGTTTGACAGCTTCTGGCCCTTAAGAACACAT 60
Frog TGCGTGTGATCAATCTTGCTTGCCAAATGGCTTGACAGCTACTGGCCCTAAAGATCACAT 60
Fugu TGTGCGCGATCAATCTTACTCGCCAAAAGGTCTGACAGCTCGGTGCCCTCG-CAACACAT 59
Tetraodon TGTGCGCGATCAATCTTACTCGCCAAAAGGTCTGACAGCTCGGTGCCCTCG-CAACACAT 59
Medaka TGTGCGCGATCAATCTTACTCGCCAAAAGGTCTGACAGCTCGGTGCCCTCG-CAACACAT 59
Stickleback TGTGCGCGATCAATCTTACTCGCCAAAAGGTCTGACAGCTCGGTGCCCTCA-CAACACAT 59
Zfish TGCACATGATCAATCTTACTTGCCAAAAGGTCTGACCG----ACTCCCCCG-CAACCCAT 55
Opossum TGGGCGCGATCAATCTTATTCGCCAAAAGGTCTGACAGCTCCTGGCCCTTGACAACACAT 60
 ** ********** ***** * **** * *** * * ***

## Slide 24
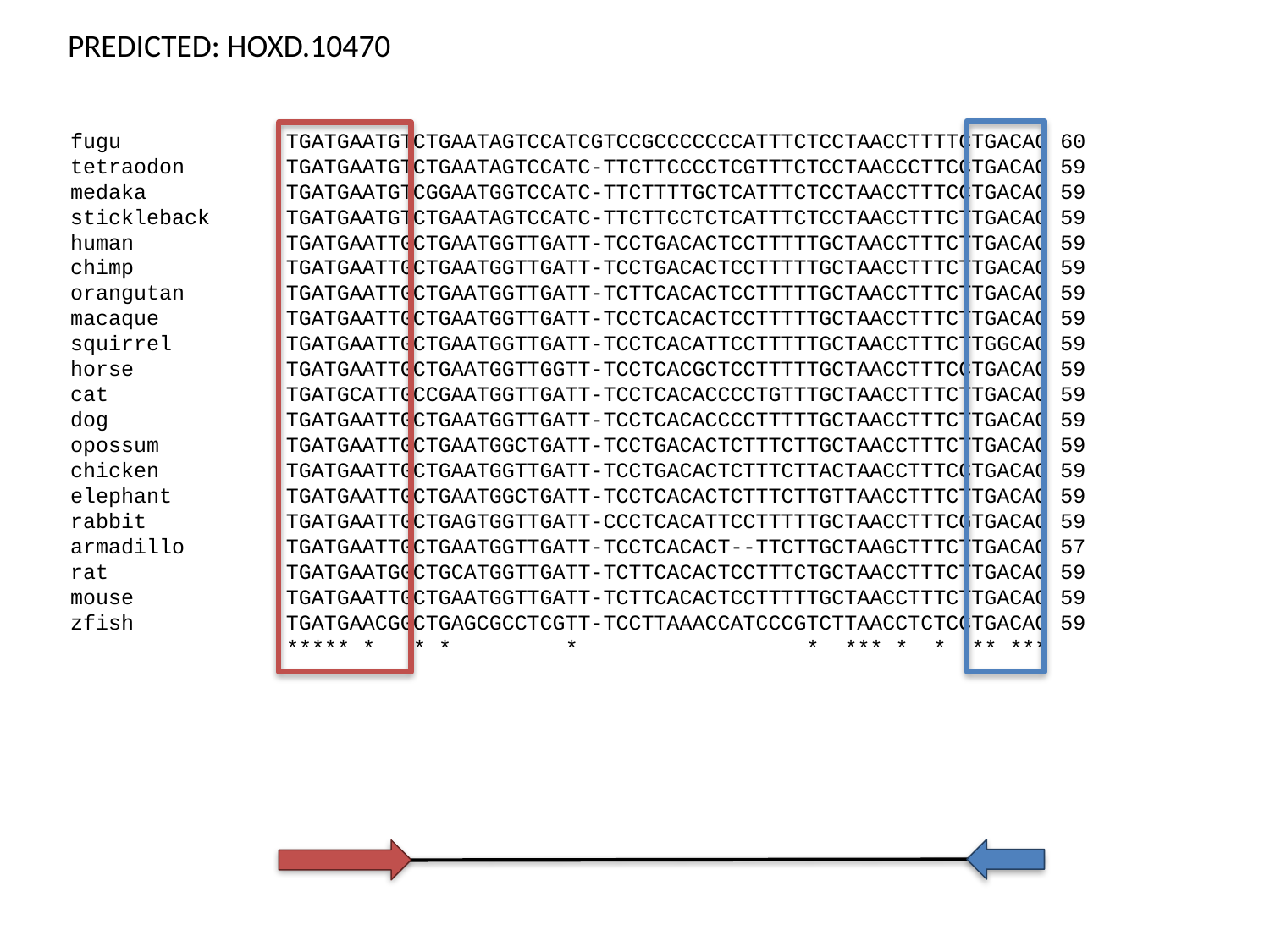

PREDICTED: HOXD.10470
fugu TGATGAATGTCTGAATAGTCCATCGTCCGCCCCCCCATTTCTCCTAACCTTTTCTGACAG 60
tetraodon TGATGAATGTCTGAATAGTCCATC-TTCTTCCCCTCGTTTCTCCTAACCCTTCCTGACAG 59
medaka TGATGAATGTCGGAATGGTCCATC-TTCTTTTGCTCATTTCTCCTAACCTTTCCTGACAG 59
stickleback TGATGAATGTCTGAATAGTCCATC-TTCTTCCTCTCATTTCTCCTAACCTTTCTTGACAG 59
human TGATGAATTGCTGAATGGTTGATT-TCCTGACACTCCTTTTTGCTAACCTTTCTTGACAG 59
chimp TGATGAATTGCTGAATGGTTGATT-TCCTGACACTCCTTTTTGCTAACCTTTCTTGACAG 59
orangutan TGATGAATTGCTGAATGGTTGATT-TCTTCACACTCCTTTTTGCTAACCTTTCTTGACAG 59
macaque TGATGAATTGCTGAATGGTTGATT-TCCTCACACTCCTTTTTGCTAACCTTTCTTGACAG 59
squirrel TGATGAATTGCTGAATGGTTGATT-TCCTCACATTCCTTTTTGCTAACCTTTCTTGGCAG 59
horse TGATGAATTGCTGAATGGTTGGTT-TCCTCACGCTCCTTTTTGCTAACCTTTCCTGACAG 59
cat TGATGCATTGCCGAATGGTTGATT-TCCTCACACCCCTGTTTGCTAACCTTTCTTGACAG 59
dog TGATGAATTGCTGAATGGTTGATT-TCCTCACACCCCTTTTTGCTAACCTTTCTTGACAG 59
opossum TGATGAATTGCTGAATGGCTGATT-TCCTGACACTCTTTCTTGCTAACCTTTCTTGACAG 59
chicken TGATGAATTGCTGAATGGTTGATT-TCCTGACACTCTTTCTTACTAACCTTTCCTGACAG 59
elephant TGATGAATTGCTGAATGGCTGATT-TCCTCACACTCTTTCTTGTTAACCTTTCTTGACAG 59
rabbit TGATGAATTGCTGAGTGGTTGATT-CCCTCACATTCCTTTTTGCTAACCTTTCGTGACAG 59
armadillo TGATGAATTGCTGAATGGTTGATT-TCCTCACACT--TTCTTGCTAAGCTTTCTTGACAG 57
rat TGATGAATGGCTGCATGGTTGATT-TCTTCACACTCCTTTCTGCTAACCTTTCTTGACAG 59
mouse TGATGAATTGCTGAATGGTTGATT-TCTTCACACTCCTTTTTGCTAACCTTTCTTGACAG 59
zfish TGATGAACGGCTGAGCGCCTCGTT-TCCTTAAACCATCCCGTCTTAACCTCTCCTGACAG 59
 ***** * * * * * *** * * ** ***

## Slide 25
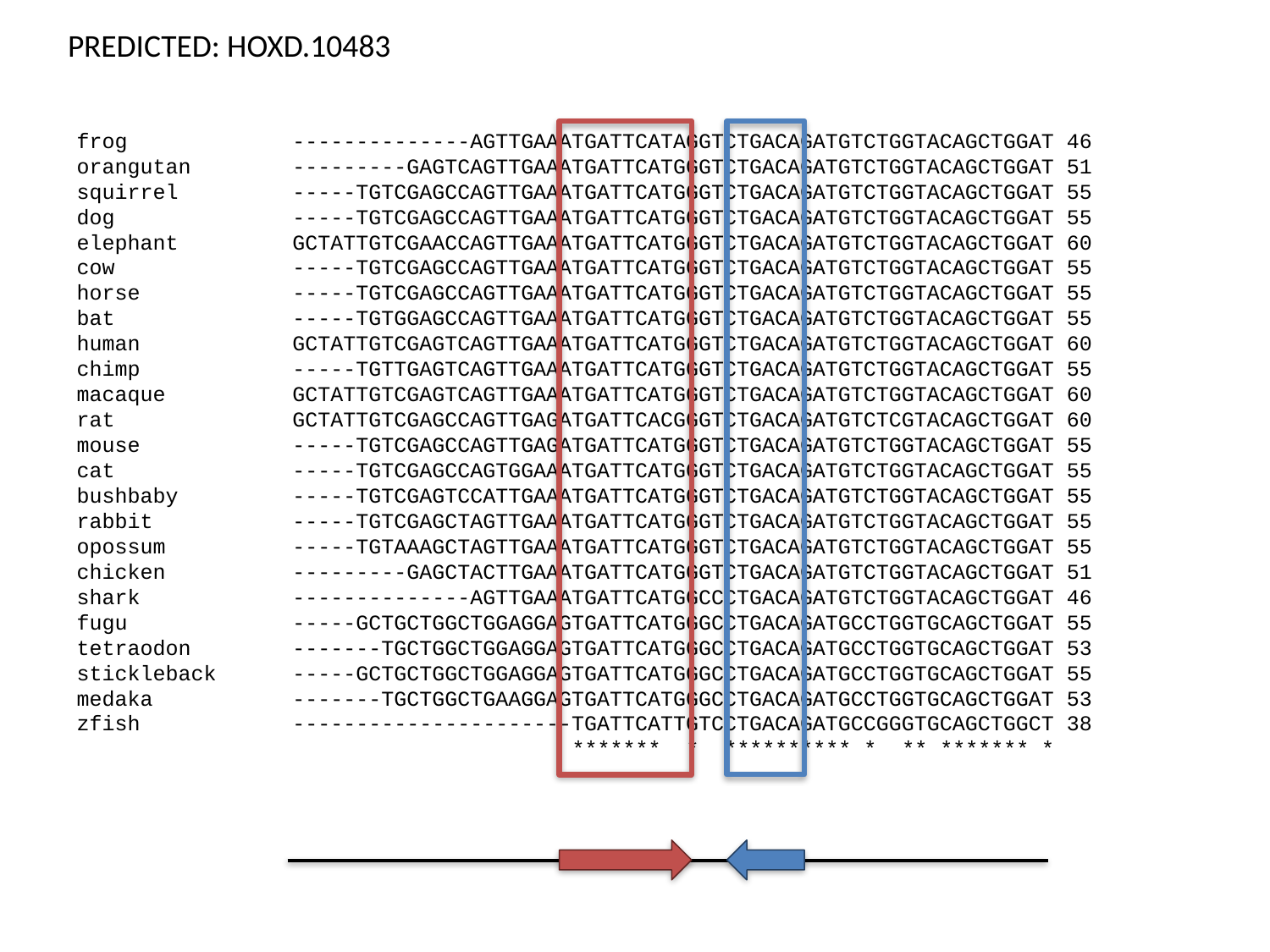

PREDICTED: HOXD.10483
frog --------------AGTTGAAATGATTCATAGGTCTGACAGATGTCTGGTACAGCTGGAT 46
orangutan ---------GAGTCAGTTGAAATGATTCATGGGTCTGACAGATGTCTGGTACAGCTGGAT 51
squirrel -----TGTCGAGCCAGTTGAAATGATTCATGGGTCTGACAGATGTCTGGTACAGCTGGAT 55
dog -----TGTCGAGCCAGTTGAAATGATTCATGGGTCTGACAGATGTCTGGTACAGCTGGAT 55
elephant GCTATTGTCGAACCAGTTGAAATGATTCATGGGTCTGACAGATGTCTGGTACAGCTGGAT 60
cow -----TGTCGAGCCAGTTGAAATGATTCATGGGTCTGACAGATGTCTGGTACAGCTGGAT 55
horse -----TGTCGAGCCAGTTGAAATGATTCATGGGTCTGACAGATGTCTGGTACAGCTGGAT 55
bat -----TGTGGAGCCAGTTGAAATGATTCATGGGTCTGACAGATGTCTGGTACAGCTGGAT 55
human GCTATTGTCGAGTCAGTTGAAATGATTCATGGGTCTGACAGATGTCTGGTACAGCTGGAT 60
chimp -----TGTTGAGTCAGTTGAAATGATTCATGGGTCTGACAGATGTCTGGTACAGCTGGAT 55
macaque GCTATTGTCGAGTCAGTTGAAATGATTCATGGGTCTGACAGATGTCTGGTACAGCTGGAT 60
rat GCTATTGTCGAGCCAGTTGAGATGATTCACGGGTCTGACAGATGTCTCGTACAGCTGGAT 60
mouse -----TGTCGAGCCAGTTGAGATGATTCATGGGTCTGACAGATGTCTGGTACAGCTGGAT 55
cat -----TGTCGAGCCAGTGGAAATGATTCATGGGTCTGACAGATGTCTGGTACAGCTGGAT 55
bushbaby -----TGTCGAGTCCATTGAAATGATTCATGGGTCTGACAGATGTCTGGTACAGCTGGAT 55
rabbit -----TGTCGAGCTAGTTGAAATGATTCATGGGTCTGACAGATGTCTGGTACAGCTGGAT 55
opossum -----TGTAAAGCTAGTTGAAATGATTCATGGGTCTGACAGATGTCTGGTACAGCTGGAT 55
chicken ---------GAGCTACTTGAAATGATTCATGGGTCTGACAGATGTCTGGTACAGCTGGAT 51
shark --------------AGTTGAAATGATTCATGGCCCTGACAGATGTCTGGTACAGCTGGAT 46
fugu -----GCTGCTGGCTGGAGGAGTGATTCATGGGCCTGACAGATGCCTGGTGCAGCTGGAT 55
tetraodon -------TGCTGGCTGGAGGAGTGATTCATGGGCCTGACAGATGCCTGGTGCAGCTGGAT 53
stickleback -----GCTGCTGGCTGGAGGAGTGATTCATGGGCCTGACAGATGCCTGGTGCAGCTGGAT 55
medaka -------TGCTGGCTGAAGGAGTGATTCATGGGCCTGACAGATGCCTGGTGCAGCTGGAT 53
zfish ----------------------TGATTCATTGTCCTGACAGATGCCGGGTGCAGCTGGCT 38
 ******* * ********** * ** ******* *

## Slide 26
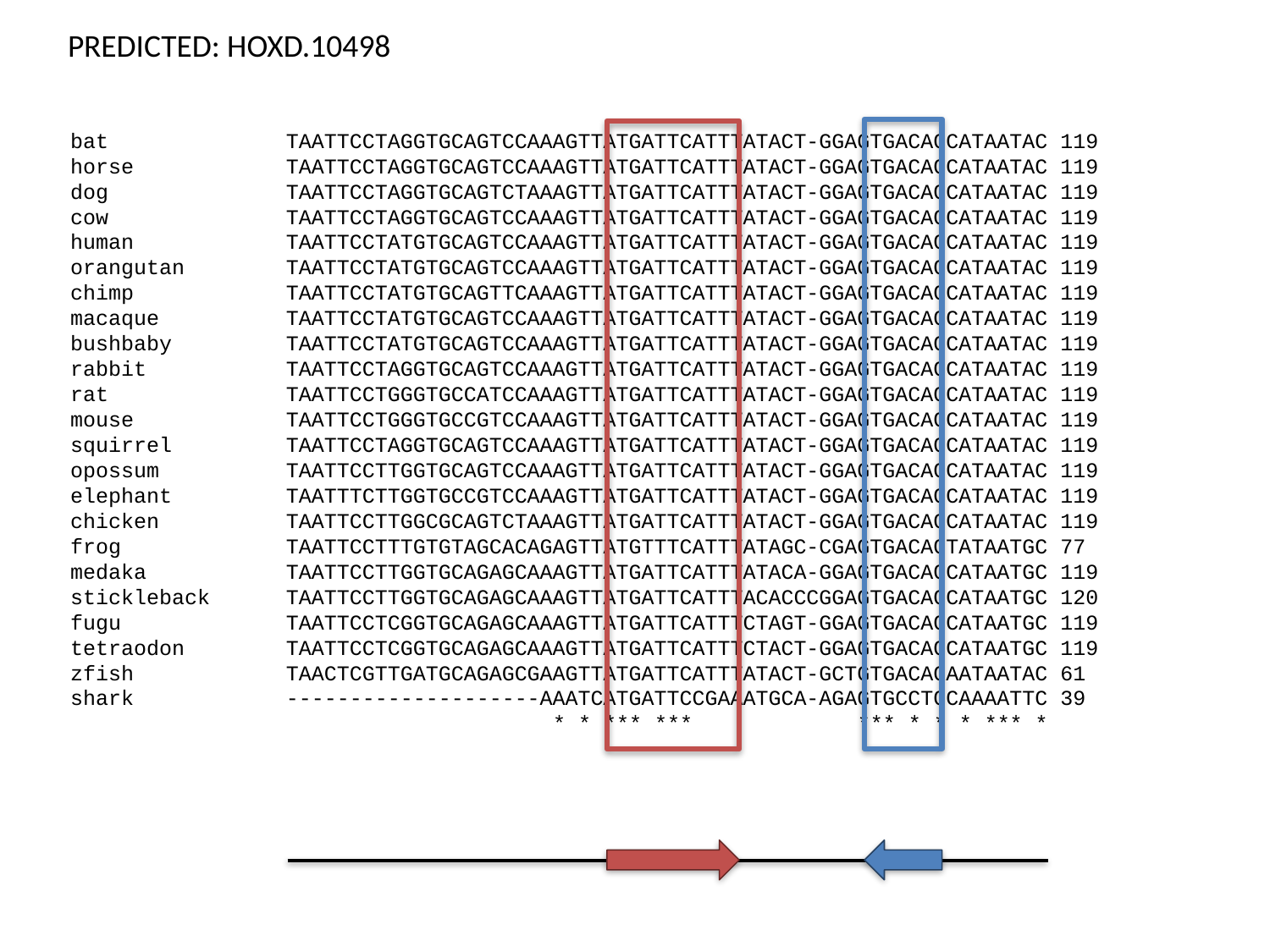

PREDICTED: HOXD.10498
bat TAATTCCTAGGTGCAGTCCAAAGTTATGATTCATTTATACT-GGAGTGACAGCATAATAC 119
horse TAATTCCTAGGTGCAGTCCAAAGTTATGATTCATTTATACT-GGAGTGACAGCATAATAC 119
dog TAATTCCTAGGTGCAGTCTAAAGTTATGATTCATTTATACT-GGAGTGACAGCATAATAC 119
cow TAATTCCTAGGTGCAGTCCAAAGTTATGATTCATTTATACT-GGAGTGACAGCATAATAC 119
human TAATTCCTATGTGCAGTCCAAAGTTATGATTCATTTATACT-GGAGTGACAGCATAATAC 119
orangutan TAATTCCTATGTGCAGTCCAAAGTTATGATTCATTTATACT-GGAGTGACAGCATAATAC 119
chimp TAATTCCTATGTGCAGTTCAAAGTTATGATTCATTTATACT-GGAGTGACAGCATAATAC 119
macaque TAATTCCTATGTGCAGTCCAAAGTTATGATTCATTTATACT-GGAGTGACAGCATAATAC 119
bushbaby TAATTCCTATGTGCAGTCCAAAGTTATGATTCATTTATACT-GGAGTGACAGCATAATAC 119
rabbit TAATTCCTAGGTGCAGTCCAAAGTTATGATTCATTTATACT-GGAGTGACAGCATAATAC 119
rat TAATTCCTGGGTGCCATCCAAAGTTATGATTCATTTATACT-GGAGTGACAGCATAATAC 119
mouse TAATTCCTGGGTGCCGTCCAAAGTTATGATTCATTTATACT-GGAGTGACAGCATAATAC 119
squirrel TAATTCCTAGGTGCAGTCCAAAGTTATGATTCATTTATACT-GGAGTGACAGCATAATAC 119
opossum TAATTCCTTGGTGCAGTCCAAAGTTATGATTCATTTATACT-GGAGTGACAGCATAATAC 119
elephant TAATTTCTTGGTGCCGTCCAAAGTTATGATTCATTTATACT-GGAGTGACAGCATAATAC 119
chicken TAATTCCTTGGCGCAGTCTAAAGTTATGATTCATTTATACT-GGAGTGACAGCATAATAC 119
frog TAATTCCTTTGTGTAGCACAGAGTTATGTTTCATTTATAGC-CGAGTGACAGTATAATGC 77
medaka TAATTCCTTGGTGCAGAGCAAAGTTATGATTCATTTATACA-GGAGTGACAGCATAATGC 119
stickleback TAATTCCTTGGTGCAGAGCAAAGTTATGATTCATTTACACCCGGAGTGACAGCATAATGC 120
fugu TAATTCCTCGGTGCAGAGCAAAGTTATGATTCATTTCTAGT-GGAGTGACAGCATAATGC 119
tetraodon TAATTCCTCGGTGCAGAGCAAAGTTATGATTCATTTCTACT-GGAGTGACAGCATAATGC 119
zfish TAACTCGTTGATGCAGAGCGAAGTTATGATTCATTTATACT-GCTGTGACAGAATAATAC 61
shark --------------------AAATCATGATTCCGAAATGCA-AGAGTGCCTGCAAAATTC 39
 * * *** *** *** * * * *** *

## Slide 27
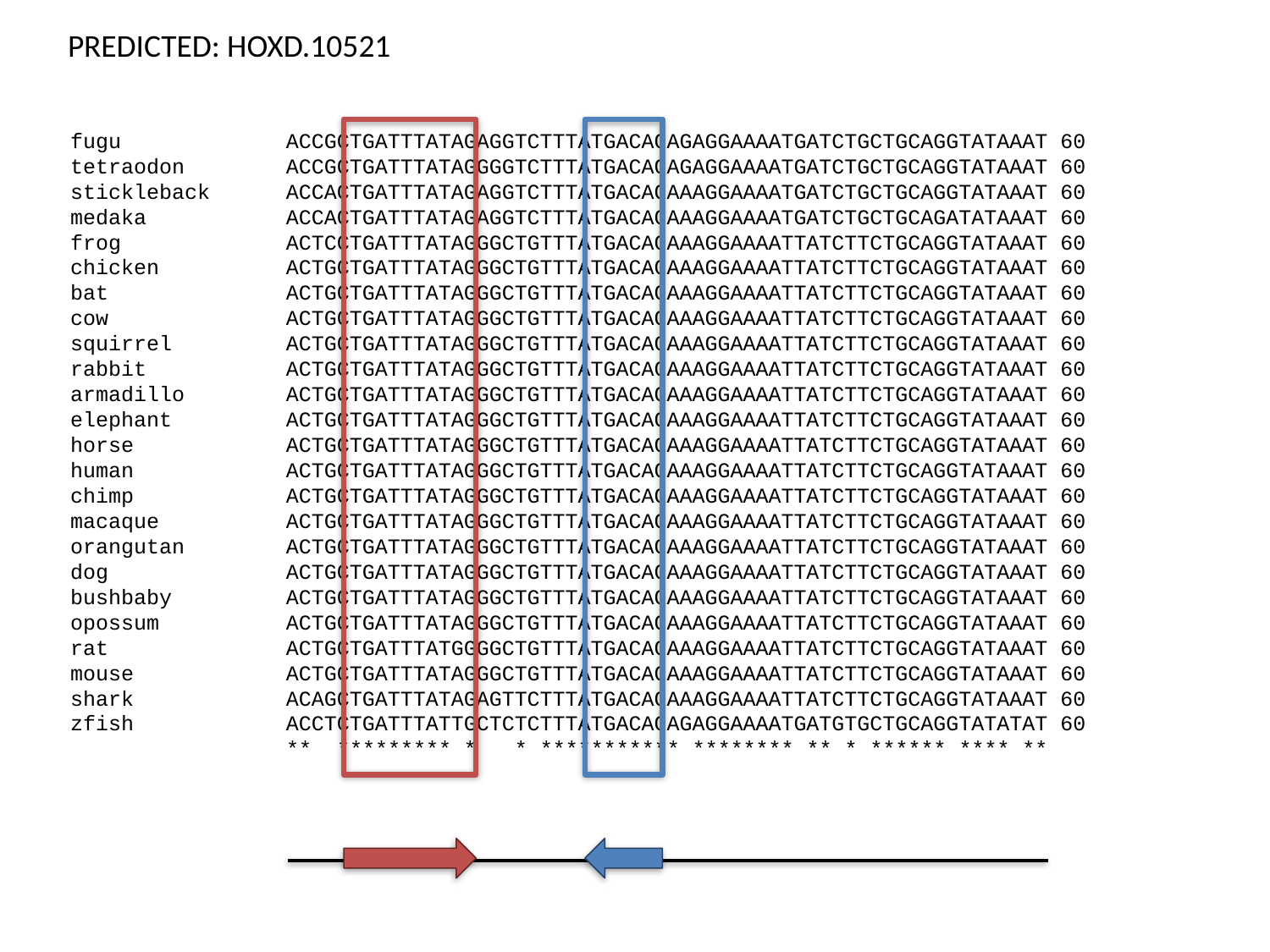

PREDICTED: HOXD.10521
fugu ACCGCTGATTTATAGAGGTCTTTATGACAGAGAGGAAAATGATCTGCTGCAGGTATAAAT 60
tetraodon ACCGCTGATTTATAGGGGTCTTTATGACAGAGAGGAAAATGATCTGCTGCAGGTATAAAT 60
stickleback ACCACTGATTTATAGAGGTCTTTATGACAGAAAGGAAAATGATCTGCTGCAGGTATAAAT 60
medaka ACCACTGATTTATAGAGGTCTTTATGACAGAAAGGAAAATGATCTGCTGCAGATATAAAT 60
frog ACTCCTGATTTATAGGGCTGTTTATGACAGAAAGGAAAATTATCTTCTGCAGGTATAAAT 60
chicken ACTGCTGATTTATAGGGCTGTTTATGACAGAAAGGAAAATTATCTTCTGCAGGTATAAAT 60
bat ACTGCTGATTTATAGGGCTGTTTATGACAGAAAGGAAAATTATCTTCTGCAGGTATAAAT 60
cow ACTGCTGATTTATAGGGCTGTTTATGACAGAAAGGAAAATTATCTTCTGCAGGTATAAAT 60
squirrel ACTGCTGATTTATAGGGCTGTTTATGACAGAAAGGAAAATTATCTTCTGCAGGTATAAAT 60
rabbit ACTGCTGATTTATAGGGCTGTTTATGACAGAAAGGAAAATTATCTTCTGCAGGTATAAAT 60
armadillo ACTGCTGATTTATAGGGCTGTTTATGACAGAAAGGAAAATTATCTTCTGCAGGTATAAAT 60
elephant ACTGCTGATTTATAGGGCTGTTTATGACAGAAAGGAAAATTATCTTCTGCAGGTATAAAT 60
horse ACTGCTGATTTATAGGGCTGTTTATGACAGAAAGGAAAATTATCTTCTGCAGGTATAAAT 60
human ACTGCTGATTTATAGGGCTGTTTATGACAGAAAGGAAAATTATCTTCTGCAGGTATAAAT 60
chimp ACTGCTGATTTATAGGGCTGTTTATGACAGAAAGGAAAATTATCTTCTGCAGGTATAAAT 60
macaque ACTGCTGATTTATAGGGCTGTTTATGACAGAAAGGAAAATTATCTTCTGCAGGTATAAAT 60
orangutan ACTGCTGATTTATAGGGCTGTTTATGACAGAAAGGAAAATTATCTTCTGCAGGTATAAAT 60
dog ACTGCTGATTTATAGGGCTGTTTATGACAGAAAGGAAAATTATCTTCTGCAGGTATAAAT 60
bushbaby ACTGCTGATTTATAGGGCTGTTTATGACAGAAAGGAAAATTATCTTCTGCAGGTATAAAT 60
opossum ACTGCTGATTTATAGGGCTGTTTATGACAGAAAGGAAAATTATCTTCTGCAGGTATAAAT 60
rat ACTGCTGATTTATGGGGCTGTTTATGACAGAAAGGAAAATTATCTTCTGCAGGTATAAAT 60
mouse ACTGCTGATTTATAGGGCTGTTTATGACAGAAAGGAAAATTATCTTCTGCAGGTATAAAT 60
shark ACAGCTGATTTATAGAGTTCTTTATGACAGAAAGGAAAATTATCTTCTGCAGGTATAAAT 60
zfish ACCTCTGATTTATTGCTCTCTTTATGACAGAGAGGAAAATGATGTGCTGCAGGTATATAT 60
 ** ********* * * *********** ******** ** * ****** **** **

## Slide 28
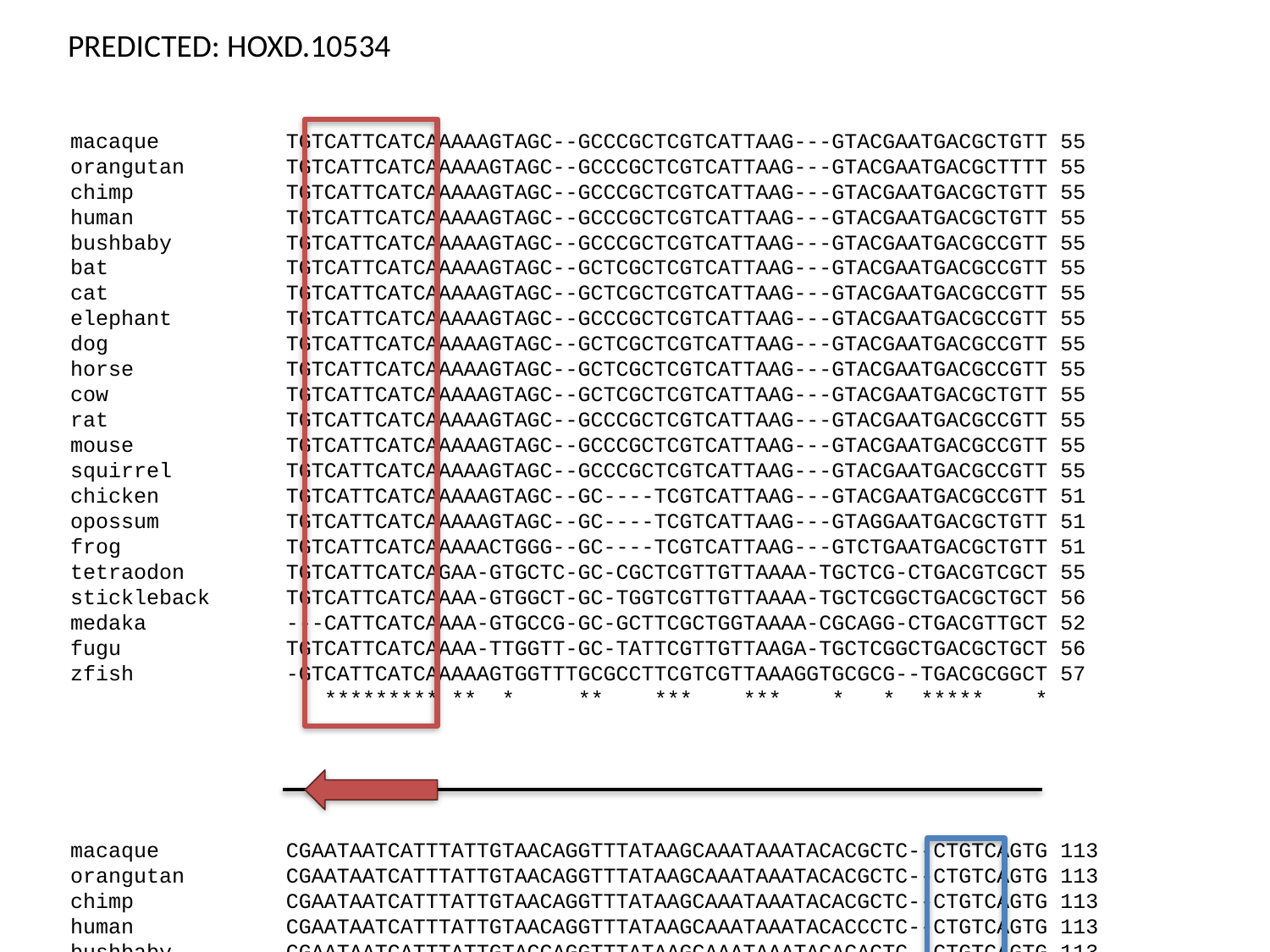

PREDICTED: HOXD.10534
macaque TGTCATTCATCAAAAAGTAGC--GCCCGCTCGTCATTAAG---GTACGAATGACGCTGTT 55
orangutan TGTCATTCATCAAAAAGTAGC--GCCCGCTCGTCATTAAG---GTACGAATGACGCTTTT 55
chimp TGTCATTCATCAAAAAGTAGC--GCCCGCTCGTCATTAAG---GTACGAATGACGCTGTT 55
human TGTCATTCATCAAAAAGTAGC--GCCCGCTCGTCATTAAG---GTACGAATGACGCTGTT 55
bushbaby TGTCATTCATCAAAAAGTAGC--GCCCGCTCGTCATTAAG---GTACGAATGACGCCGTT 55
bat TGTCATTCATCAAAAAGTAGC--GCTCGCTCGTCATTAAG---GTACGAATGACGCCGTT 55
cat TGTCATTCATCAAAAAGTAGC--GCTCGCTCGTCATTAAG---GTACGAATGACGCCGTT 55
elephant TGTCATTCATCAAAAAGTAGC--GCCCGCTCGTCATTAAG---GTACGAATGACGCCGTT 55
dog TGTCATTCATCAAAAAGTAGC--GCTCGCTCGTCATTAAG---GTACGAATGACGCCGTT 55
horse TGTCATTCATCAAAAAGTAGC--GCTCGCTCGTCATTAAG---GTACGAATGACGCCGTT 55
cow TGTCATTCATCAAAAAGTAGC--GCTCGCTCGTCATTAAG---GTACGAATGACGCTGTT 55
rat TGTCATTCATCAAAAAGTAGC--GCCCGCTCGTCATTAAG---GTACGAATGACGCCGTT 55
mouse TGTCATTCATCAAAAAGTAGC--GCCCGCTCGTCATTAAG---GTACGAATGACGCCGTT 55
squirrel TGTCATTCATCAAAAAGTAGC--GCCCGCTCGTCATTAAG---GTACGAATGACGCCGTT 55
chicken TGTCATTCATCAAAAAGTAGC--GC----TCGTCATTAAG---GTACGAATGACGCCGTT 51
opossum TGTCATTCATCAAAAAGTAGC--GC----TCGTCATTAAG---GTAGGAATGACGCTGTT 51
frog TGTCATTCATCAAAAACTGGG--GC----TCGTCATTAAG---GTCTGAATGACGCTGTT 51
tetraodon TGTCATTCATCAGAA-GTGCTC-GC-CGCTCGTTGTTAAAA-TGCTCG-CTGACGTCGCT 55
stickleback TGTCATTCATCAAAA-GTGGCT-GC-TGGTCGTTGTTAAAA-TGCTCGGCTGACGCTGCT 56
medaka ---CATTCATCAAAA-GTGCCG-GC-GCTTCGCTGGTAAAA-CGCAGG-CTGACGTTGCT 52
fugu TGTCATTCATCAAAA-TTGGTT-GC-TATTCGTTGTTAAGA-TGCTCGGCTGACGCTGCT 56
zfish -GTCATTCATCAAAAAGTGGTTTGCGCCTTCGTCGTTAAAGGTGCGCG--TGACGCGGCT 57
 ********* ** * ** *** *** * * ***** *
macaque CGAATAATCATTTATTGTAACAGGTTTATAAGCAAATAAATACACGCTC--CTGTCAGTG 113
orangutan CGAATAATCATTTATTGTAACAGGTTTATAAGCAAATAAATACACGCTC--CTGTCAGTG 113
chimp CGAATAATCATTTATTGTAACAGGTTTATAAGCAAATAAATACACGCTC--CTGTCAGTG 113
human CGAATAATCATTTATTGTAACAGGTTTATAAGCAAATAAATACACCCTC--CTGTCAGTG 113
bushbaby CGAATAATCATTTATTGTACCAGGTTTATAAGCAAATAAATACACACTC--CTGTCAGTG 113
bat CGAATAATCATTTATTGTAACAGGTTTATAAGCAAATAAATACACGCTC--CTGTCAGTG 113
cat CGAATAATCATTTATTGTAACAGGTTTATAAGCAAATAAATACACGCTC--CTGTCAGTG 113
elephant CGAATGATCATTTATTGTAACAGGTTTATAAGCAAATAAATACACGCTC--CTGTCAGTG 113
dog CGAATAATCATTTATTGTAACAGGTTTATAAGCAAATAAATACACGCTC--CTGTCAGTG 113
horse CGAATAATCATTTATTGTAACAGGTTTATAAGCAAATAAATACACGCTC--CTGTCAGTG 113
cow CGAATAATCATTTATTGTAACAGGTTTATAAGCAAATAAATACACGTTC--CTGTCAGTG 113
rat CGAATAATCATTTATTGTAACAGGTTTATAAGCAAATAAATACACGATC--CTGTCAGTG 113
mouse CGAATAATCATTTATTGTAACAGGTTTATAAGCAAATAAATACACGCTC--CTGTCAGTG 113
squirrel CGAATAATCATTTATTGTAACAGGTTTATAAGCAAATAAATACACGCTC--CTGTCAGTG 113
chicken CGAATAATCATTTATTGTAACAGGTTTATAAGCAAATAAATACAAGCTC--CTGTCAGTG 109
opossum CGAATAATCATTTATTGTAACAGGTTTATAAGCAAATAAATACGAGCTC--TTGTCAGTG 109
frog TGAATAATCATTTATTGTAACAGTTTTATAAGCAAATAAATAGAGGCTC--CTGTCAGTG 109
tetraodon CCAATGATCATTTATTGTAACAGGTTTATCAGCAAATAAATAGGAGAGGG-CTGTCACTT 114
stickleback CCAATGATCATTTATTGTAACAGGTTTATAAGCAAATAAATAGGAGAGGG-CTGTCACTT 115
medaka CCAATGATCATTTATTGTAACAGGTTTATAAGCAAATAAATAGGAGAGGG-CTGTCACTT 111
fugu CCAATGATCATTTATTGTAACAGGTTTATAAGCAAATAAATAGGAGAGGG-CTGTCACTT 115
zfish CGAATGATCATTTATTGTAACAGGTTTATAAGCAAATAAATAGGAGAGAGACTGTCATCG 117
 *** ************* *** ***** ************ *****

## Slide 29
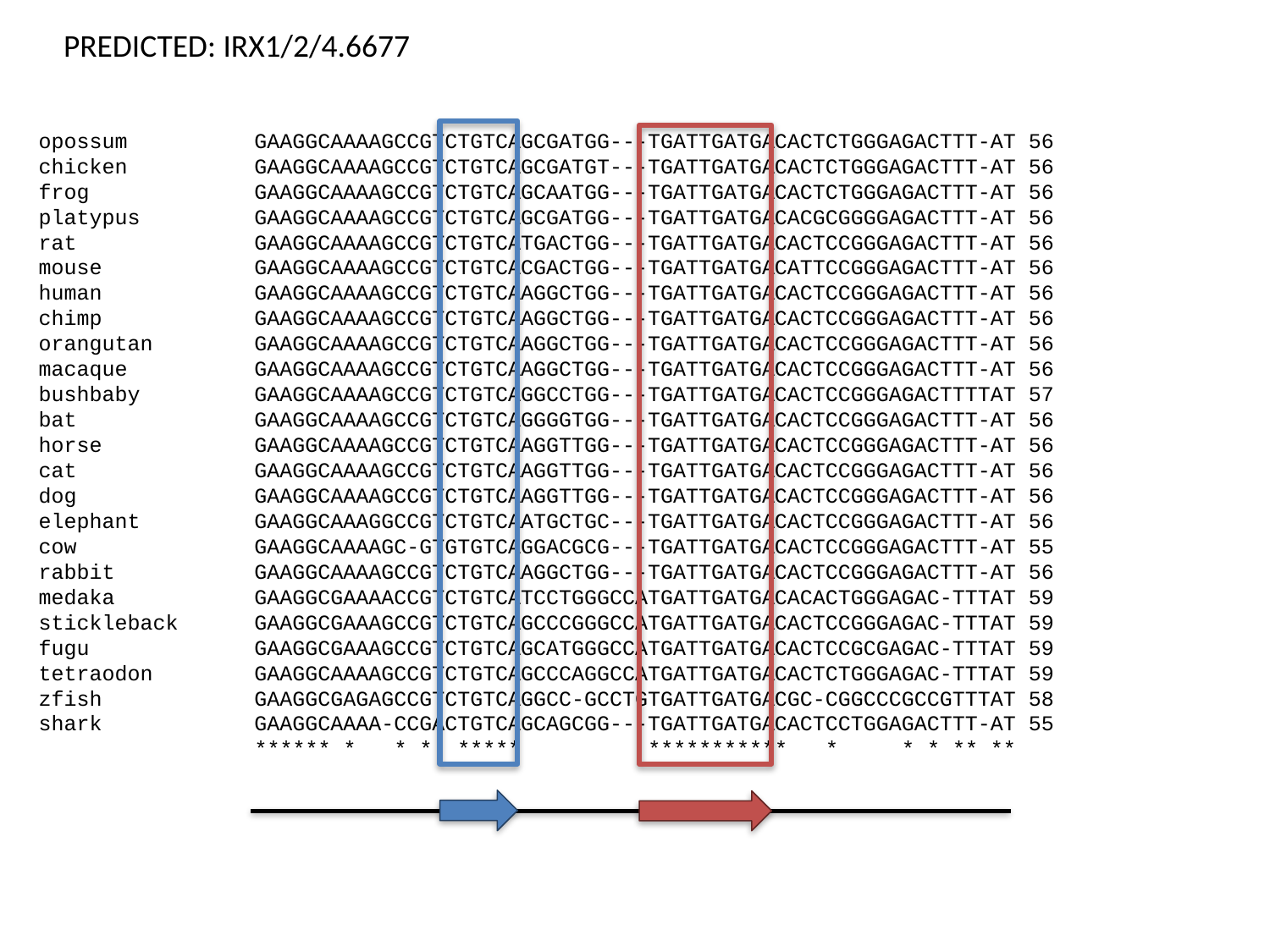

PREDICTED: IRX1/2/4.6677
opossum GAAGGCAAAAGCCGTCTGTCAGCGATGG---TGATTGATGACACTCTGGGAGACTTT-AT 56
chicken GAAGGCAAAAGCCGTCTGTCAGCGATGT---TGATTGATGACACTCTGGGAGACTTT-AT 56
frog GAAGGCAAAAGCCGTCTGTCAGCAATGG---TGATTGATGACACTCTGGGAGACTTT-AT 56
platypus GAAGGCAAAAGCCGTCTGTCAGCGATGG---TGATTGATGACACGCGGGGAGACTTT-AT 56
rat GAAGGCAAAAGCCGTCTGTCATGACTGG---TGATTGATGACACTCCGGGAGACTTT-AT 56
mouse GAAGGCAAAAGCCGTCTGTCACGACTGG---TGATTGATGACATTCCGGGAGACTTT-AT 56
human GAAGGCAAAAGCCGTCTGTCAAGGCTGG---TGATTGATGACACTCCGGGAGACTTT-AT 56
chimp GAAGGCAAAAGCCGTCTGTCAAGGCTGG---TGATTGATGACACTCCGGGAGACTTT-AT 56
orangutan GAAGGCAAAAGCCGTCTGTCAAGGCTGG---TGATTGATGACACTCCGGGAGACTTT-AT 56
macaque GAAGGCAAAAGCCGTCTGTCAAGGCTGG---TGATTGATGACACTCCGGGAGACTTT-AT 56
bushbaby GAAGGCAAAAGCCGTCTGTCAGGCCTGG---TGATTGATGACACTCCGGGAGACTTTTAT 57
bat GAAGGCAAAAGCCGTCTGTCAGGGGTGG---TGATTGATGACACTCCGGGAGACTTT-AT 56
horse GAAGGCAAAAGCCGTCTGTCAAGGTTGG---TGATTGATGACACTCCGGGAGACTTT-AT 56
cat GAAGGCAAAAGCCGTCTGTCAAGGTTGG---TGATTGATGACACTCCGGGAGACTTT-AT 56
dog GAAGGCAAAAGCCGTCTGTCAAGGTTGG---TGATTGATGACACTCCGGGAGACTTT-AT 56
elephant GAAGGCAAAGGCCGTCTGTCAATGCTGC---TGATTGATGACACTCCGGGAGACTTT-AT 56
cow GAAGGCAAAAGC-GTGTGTCAGGACGCG---TGATTGATGACACTCCGGGAGACTTT-AT 55
rabbit GAAGGCAAAAGCCGTCTGTCAAGGCTGG---TGATTGATGACACTCCGGGAGACTTT-AT 56
medaka GAAGGCGAAAACCGTCTGTCATCCTGGGCCATGATTGATGACACACTGGGAGAC-TTTAT 59
stickleback GAAGGCGAAAGCCGTCTGTCAGCCCGGGCCATGATTGATGACACTCCGGGAGAC-TTTAT 59
fugu GAAGGCGAAAGCCGTCTGTCAGCATGGGCCATGATTGATGACACTCCGCGAGAC-TTTAT 59
tetraodon GAAGGCAAAAGCCGTCTGTCAGCCCAGGCCATGATTGATGACACTCTGGGAGAC-TTTAT 59
zfish GAAGGCGAGAGCCGTCTGTCAGGCC-GCCTGTGATTGATGACGC-CGGCCCGCCGTTTAT 58
shark GAAGGCAAAA-CCGACTGTCAGCAGCGG---TGATTGATGACACTCCTGGAGACTTT-AT 55
 ****** * * * ***** *********** * * * ** **

## Slide 30
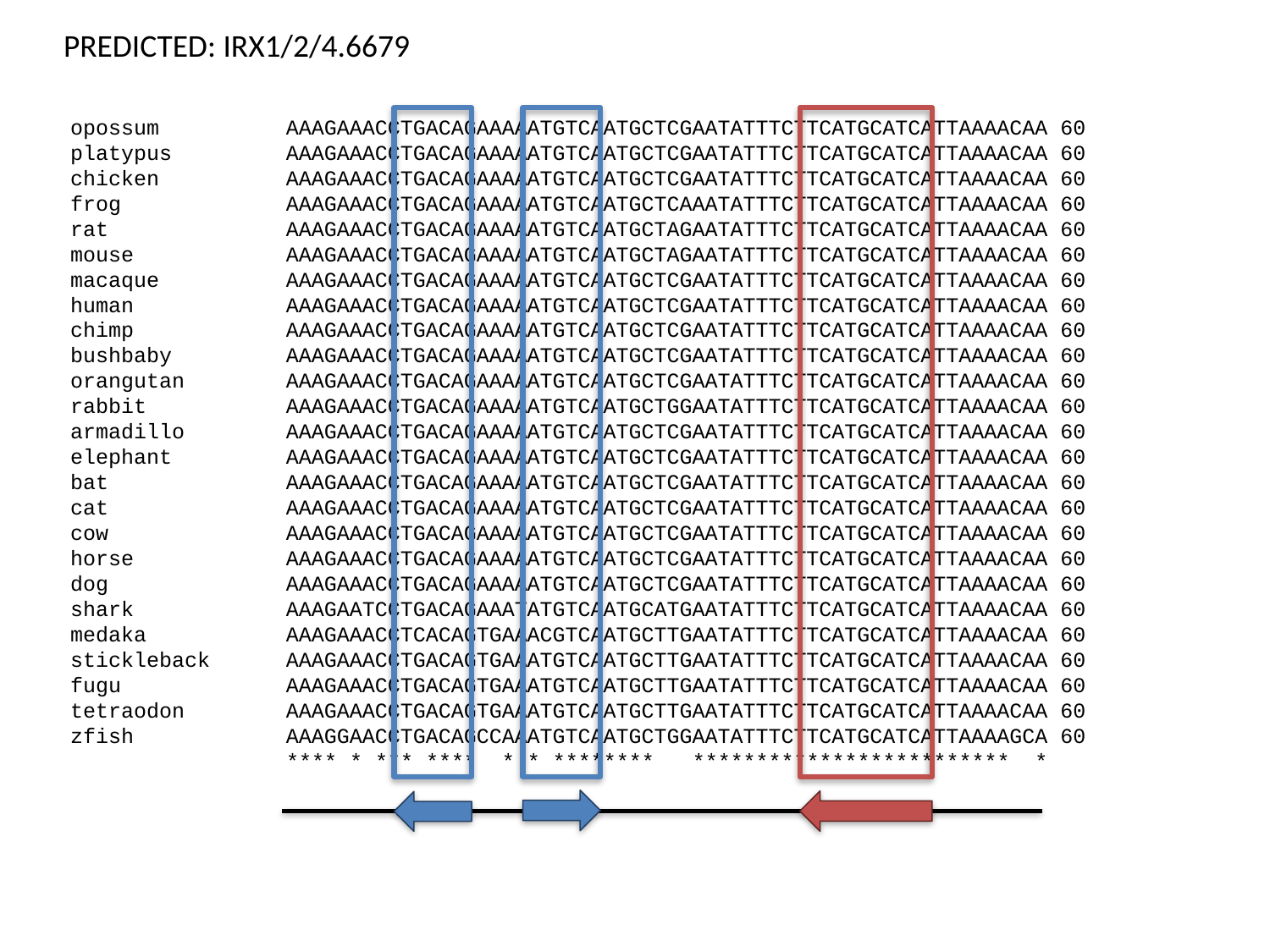

PREDICTED: IRX1/2/4.6679
opossum AAAGAAACCTGACAGAAAAATGTCAATGCTCGAATATTTCTTCATGCATCATTAAAACAA 60
platypus AAAGAAACCTGACAGAAAAATGTCAATGCTCGAATATTTCTTCATGCATCATTAAAACAA 60
chicken AAAGAAACCTGACAGAAAAATGTCAATGCTCGAATATTTCTTCATGCATCATTAAAACAA 60
frog AAAGAAACCTGACAGAAAAATGTCAATGCTCAAATATTTCTTCATGCATCATTAAAACAA 60
rat AAAGAAACCTGACAGAAAAATGTCAATGCTAGAATATTTCTTCATGCATCATTAAAACAA 60
mouse AAAGAAACCTGACAGAAAAATGTCAATGCTAGAATATTTCTTCATGCATCATTAAAACAA 60
macaque AAAGAAACCTGACAGAAAAATGTCAATGCTCGAATATTTCTTCATGCATCATTAAAACAA 60
human AAAGAAACCTGACAGAAAAATGTCAATGCTCGAATATTTCTTCATGCATCATTAAAACAA 60
chimp AAAGAAACCTGACAGAAAAATGTCAATGCTCGAATATTTCTTCATGCATCATTAAAACAA 60
bushbaby AAAGAAACCTGACAGAAAAATGTCAATGCTCGAATATTTCTTCATGCATCATTAAAACAA 60
orangutan AAAGAAACCTGACAGAAAAATGTCAATGCTCGAATATTTCTTCATGCATCATTAAAACAA 60
rabbit AAAGAAACCTGACAGAAAAATGTCAATGCTGGAATATTTCTTCATGCATCATTAAAACAA 60
armadillo AAAGAAACCTGACAGAAAAATGTCAATGCTCGAATATTTCTTCATGCATCATTAAAACAA 60
elephant AAAGAAACCTGACAGAAAAATGTCAATGCTCGAATATTTCTTCATGCATCATTAAAACAA 60
bat AAAGAAACCTGACAGAAAAATGTCAATGCTCGAATATTTCTTCATGCATCATTAAAACAA 60
cat AAAGAAACCTGACAGAAAAATGTCAATGCTCGAATATTTCTTCATGCATCATTAAAACAA 60
cow AAAGAAACCTGACAGAAAAATGTCAATGCTCGAATATTTCTTCATGCATCATTAAAACAA 60
horse AAAGAAACCTGACAGAAAAATGTCAATGCTCGAATATTTCTTCATGCATCATTAAAACAA 60
dog AAAGAAACCTGACAGAAAAATGTCAATGCTCGAATATTTCTTCATGCATCATTAAAACAA 60
shark AAAGAATCCTGACAGAAATATGTCAATGCATGAATATTTCTTCATGCATCATTAAAACAA 60
medaka AAAGAAACCTCACAGTGAAACGTCAATGCTTGAATATTTCTTCATGCATCATTAAAACAA 60
stickleback AAAGAAACCTGACAGTGAAATGTCAATGCTTGAATATTTCTTCATGCATCATTAAAACAA 60
fugu AAAGAAACCTGACAGTGAAATGTCAATGCTTGAATATTTCTTCATGCATCATTAAAACAA 60
tetraodon AAAGAAACCTGACAGTGAAATGTCAATGCTTGAATATTTCTTCATGCATCATTAAAACAA 60
zfish AAAGGAACCTGACAGCCAAATGTCAATGCTGGAATATTTCTTCATGCATCATTAAAAGCA 60
 **** * *** **** * * ******** ************************* *

## Slide 31
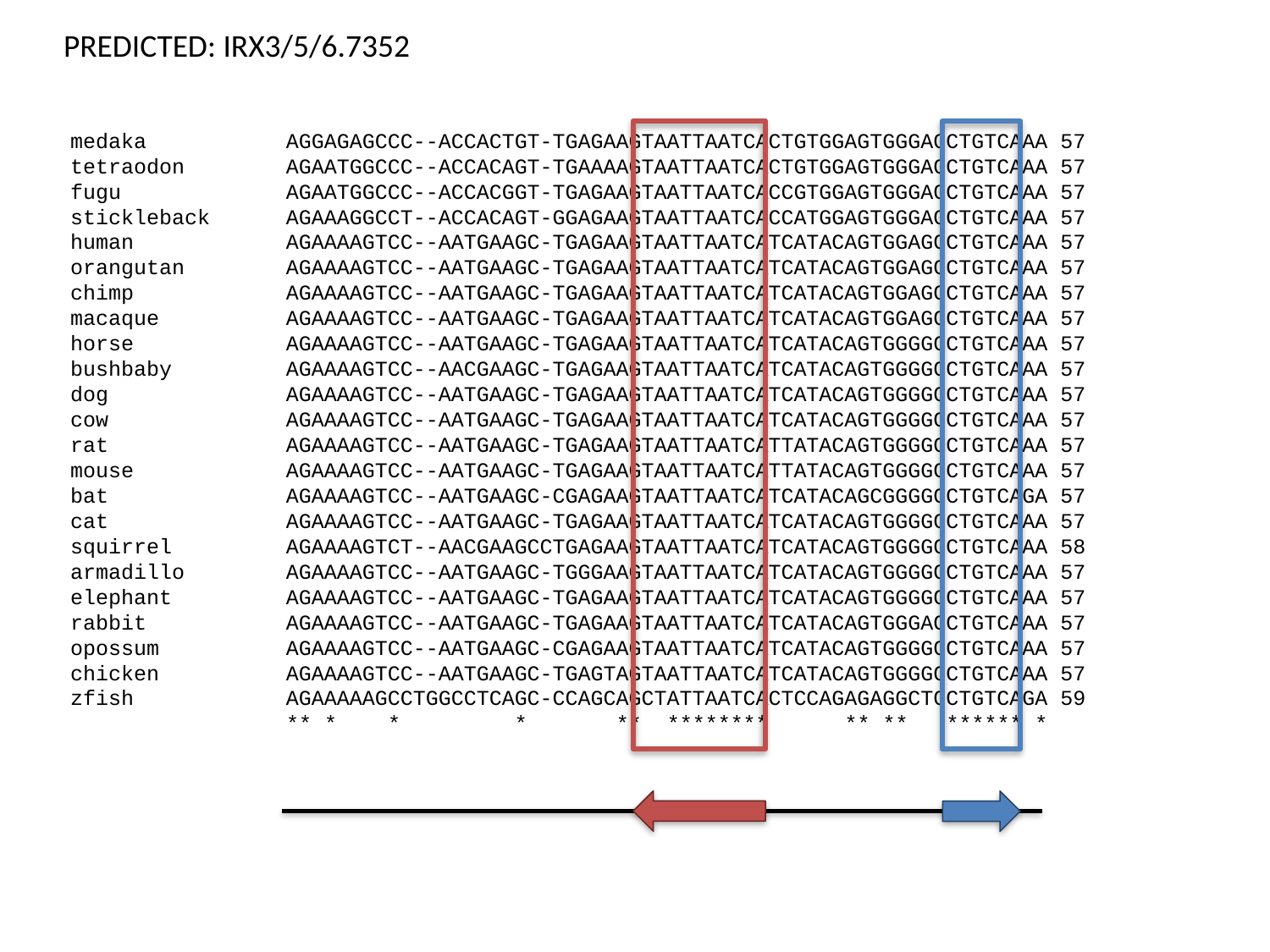

PREDICTED: IRX3/5/6.7352
medaka AGGAGAGCCC--ACCACTGT-TGAGAAGTAATTAATCACTGTGGAGTGGGAGCTGTCAAA 57
tetraodon AGAATGGCCC--ACCACAGT-TGAAAAGTAATTAATCACTGTGGAGTGGGAGCTGTCAAA 57
fugu AGAATGGCCC--ACCACGGT-TGAGAAGTAATTAATCACCGTGGAGTGGGAGCTGTCAAA 57
stickleback AGAAAGGCCT--ACCACAGT-GGAGAAGTAATTAATCACCATGGAGTGGGAGCTGTCAAA 57
human AGAAAAGTCC--AATGAAGC-TGAGAAGTAATTAATCATCATACAGTGGAGCCTGTCAAA 57
orangutan AGAAAAGTCC--AATGAAGC-TGAGAAGTAATTAATCATCATACAGTGGAGCCTGTCAAA 57
chimp AGAAAAGTCC--AATGAAGC-TGAGAAGTAATTAATCATCATACAGTGGAGCCTGTCAAA 57
macaque AGAAAAGTCC--AATGAAGC-TGAGAAGTAATTAATCATCATACAGTGGAGCCTGTCAAA 57
horse AGAAAAGTCC--AATGAAGC-TGAGAAGTAATTAATCATCATACAGTGGGGCCTGTCAAA 57
bushbaby AGAAAAGTCC--AACGAAGC-TGAGAAGTAATTAATCATCATACAGTGGGGCCTGTCAAA 57
dog AGAAAAGTCC--AATGAAGC-TGAGAAGTAATTAATCATCATACAGTGGGGCCTGTCAAA 57
cow AGAAAAGTCC--AATGAAGC-TGAGAAGTAATTAATCATCATACAGTGGGGCCTGTCAAA 57
rat AGAAAAGTCC--AATGAAGC-TGAGAAGTAATTAATCATTATACAGTGGGGCCTGTCAAA 57
mouse AGAAAAGTCC--AATGAAGC-TGAGAAGTAATTAATCATTATACAGTGGGGCCTGTCAAA 57
bat AGAAAAGTCC--AATGAAGC-CGAGAAGTAATTAATCATCATACAGCGGGGCCTGTCAGA 57
cat AGAAAAGTCC--AATGAAGC-TGAGAAGTAATTAATCATCATACAGTGGGGCCTGTCAAA 57
squirrel AGAAAAGTCT--AACGAAGCCTGAGAAGTAATTAATCATCATACAGTGGGGCCTGTCAAA 58
armadillo AGAAAAGTCC--AATGAAGC-TGGGAAGTAATTAATCATCATACAGTGGGGCCTGTCAAA 57
elephant AGAAAAGTCC--AATGAAGC-TGAGAAGTAATTAATCATCATACAGTGGGGCCTGTCAAA 57
rabbit AGAAAAGTCC--AATGAAGC-TGAGAAGTAATTAATCATCATACAGTGGGACCTGTCAAA 57
opossum AGAAAAGTCC--AATGAAGC-CGAGAAGTAATTAATCATCATACAGTGGGGCCTGTCAAA 57
chicken AGAAAAGTCC--AATGAAGC-TGAGTAGTAATTAATCATCATACAGTGGGGCCTGTCAAA 57
zfish AGAAAAAGCCTGGCCTCAGC-CCAGCAGCTATTAATCACTCCAGAGAGGCTCCTGTCAGA 59
 ** * * * ** ******** ** ** ****** *

## Slide 32
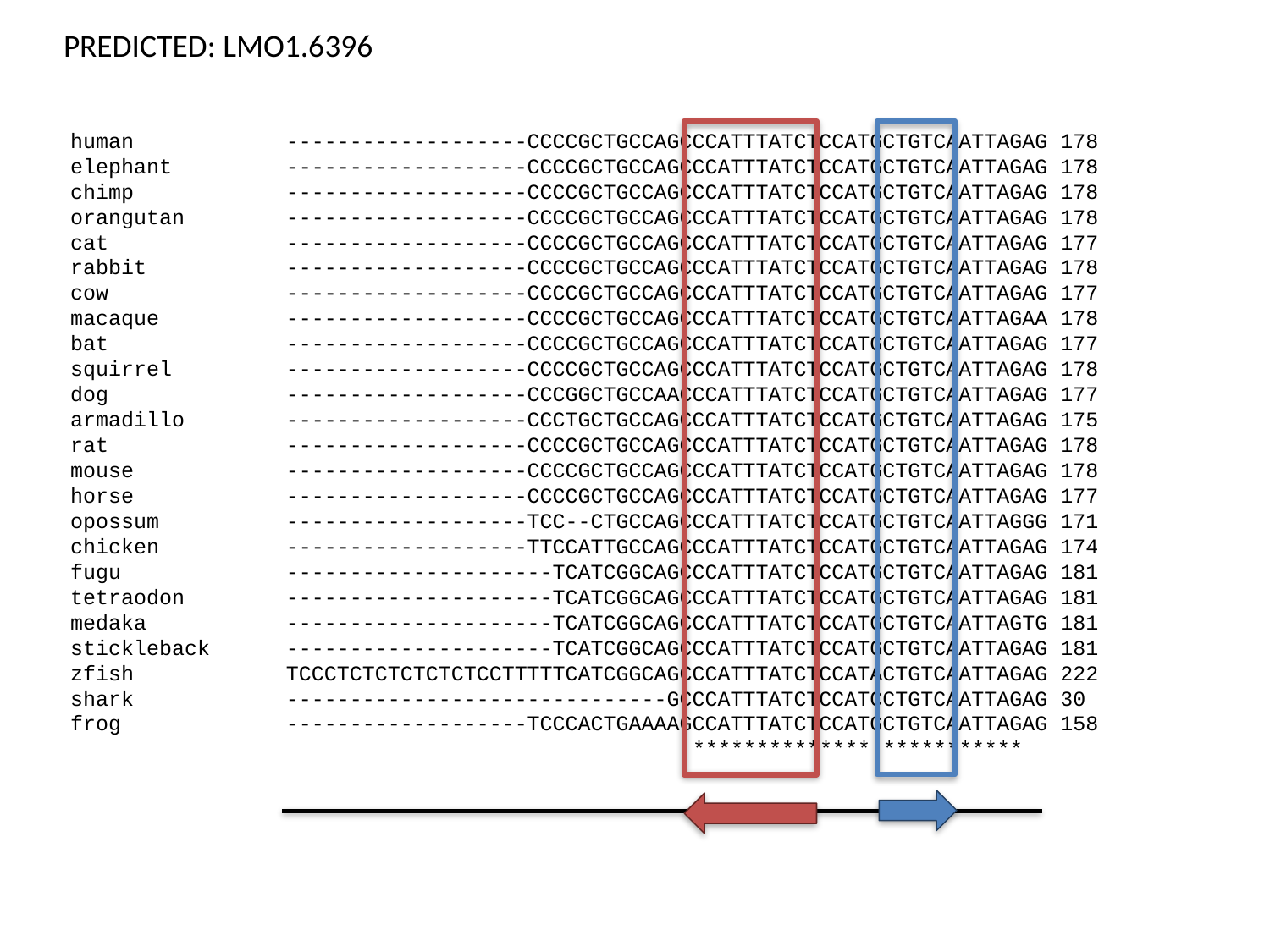

PREDICTED: LMO1.6396
human -------------------CCCCGCTGCCAGCCCATTTATCTCCATGCTGTCAATTAGAG 178
elephant -------------------CCCCGCTGCCAGCCCATTTATCTCCATGCTGTCAATTAGAG 178
chimp -------------------CCCCGCTGCCAGCCCATTTATCTCCATGCTGTCAATTAGAG 178
orangutan -------------------CCCCGCTGCCAGCCCATTTATCTCCATGCTGTCAATTAGAG 178
cat -------------------CCCCGCTGCCAGCCCATTTATCTCCATGCTGTCAATTAGAG 177
rabbit -------------------CCCCGCTGCCAGCCCATTTATCTCCATGCTGTCAATTAGAG 178
cow -------------------CCCCGCTGCCAGCCCATTTATCTCCATGCTGTCAATTAGAG 177
macaque -------------------CCCCGCTGCCAGCCCATTTATCTCCATGCTGTCAATTAGAA 178
bat -------------------CCCCGCTGCCAGCCCATTTATCTCCATGCTGTCAATTAGAG 177
squirrel -------------------CCCCGCTGCCAGCCCATTTATCTCCATGCTGTCAATTAGAG 178
dog -------------------CCCGGCTGCCAACCCATTTATCTCCATGCTGTCAATTAGAG 177
armadillo -------------------CCCTGCTGCCAGCCCATTTATCTCCATGCTGTCAATTAGAG 175
rat -------------------CCCCGCTGCCAGCCCATTTATCTCCATGCTGTCAATTAGAG 178
mouse -------------------CCCCGCTGCCAGCCCATTTATCTCCATGCTGTCAATTAGAG 178
horse -------------------CCCCGCTGCCAGCCCATTTATCTCCATGCTGTCAATTAGAG 177
opossum -------------------TCC--CTGCCAGCCCATTTATCTCCATGCTGTCAATTAGGG 171
chicken -------------------TTCCATTGCCAGCCCATTTATCTCCATGCTGTCAATTAGAG 174
fugu ---------------------TCATCGGCAGCCCATTTATCTCCATGCTGTCAATTAGAG 181
tetraodon ---------------------TCATCGGCAGCCCATTTATCTCCATGCTGTCAATTAGAG 181
medaka ---------------------TCATCGGCAGCCCATTTATCTCCATGCTGTCAATTAGTG 181
stickleback ---------------------TCATCGGCAGCCCATTTATCTCCATGCTGTCAATTAGAG 181
zfish TCCCTCTCTCTCTCTCCTTTTTCATCGGCAGCCCATTTATCTCCATACTGTCAATTAGAG 222
shark ------------------------------GCCCATTTATCTCCATCCTGTCAATTAGAG 30
frog -------------------TCCCACTGAAAAGCCATTTATCTCCATGCTGTCAATTAGAG 158
 ************** ***********

## Slide 33
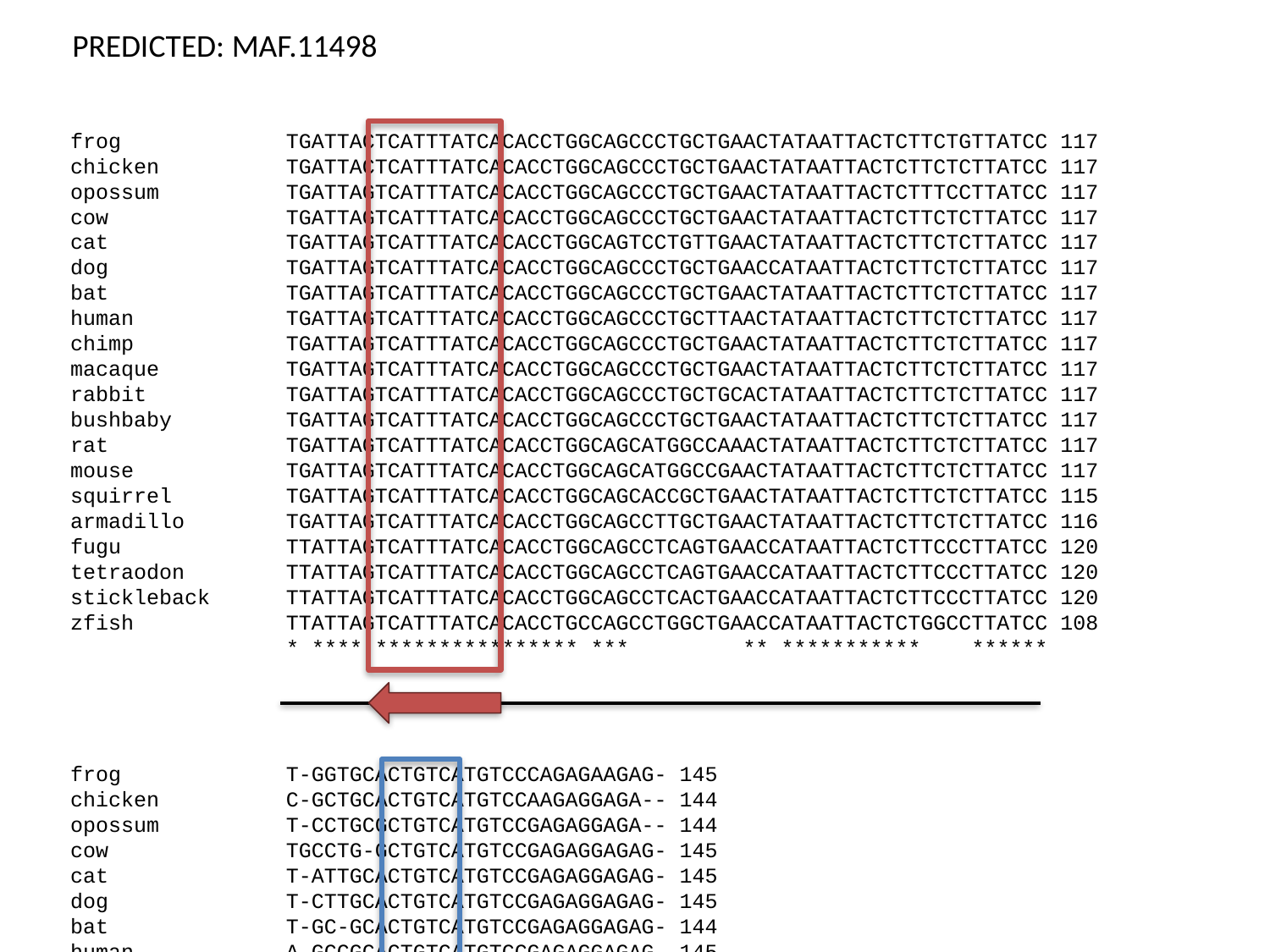

PREDICTED: MAF.11498
frog TGATTACTCATTTATCACACCTGGCAGCCCTGCTGAACTATAATTACTCTTCTGTTATCC 117
chicken TGATTACTCATTTATCACACCTGGCAGCCCTGCTGAACTATAATTACTCTTCTCTTATCC 117
opossum TGATTAGTCATTTATCACACCTGGCAGCCCTGCTGAACTATAATTACTCTTTCCTTATCC 117
cow TGATTAGTCATTTATCACACCTGGCAGCCCTGCTGAACTATAATTACTCTTCTCTTATCC 117
cat TGATTAGTCATTTATCACACCTGGCAGTCCTGTTGAACTATAATTACTCTTCTCTTATCC 117
dog TGATTAGTCATTTATCACACCTGGCAGCCCTGCTGAACCATAATTACTCTTCTCTTATCC 117
bat TGATTAGTCATTTATCACACCTGGCAGCCCTGCTGAACTATAATTACTCTTCTCTTATCC 117
human TGATTAGTCATTTATCACACCTGGCAGCCCTGCTTAACTATAATTACTCTTCTCTTATCC 117
chimp TGATTAGTCATTTATCACACCTGGCAGCCCTGCTGAACTATAATTACTCTTCTCTTATCC 117
macaque TGATTAGTCATTTATCACACCTGGCAGCCCTGCTGAACTATAATTACTCTTCTCTTATCC 117
rabbit TGATTAGTCATTTATCACACCTGGCAGCCCTGCTGCACTATAATTACTCTTCTCTTATCC 117
bushbaby TGATTAGTCATTTATCACACCTGGCAGCCCTGCTGAACTATAATTACTCTTCTCTTATCC 117
rat TGATTAGTCATTTATCACACCTGGCAGCATGGCCAAACTATAATTACTCTTCTCTTATCC 117
mouse TGATTAGTCATTTATCACACCTGGCAGCATGGCCGAACTATAATTACTCTTCTCTTATCC 117
squirrel TGATTAGTCATTTATCACACCTGGCAGCACCGCTGAACTATAATTACTCTTCTCTTATCC 115
armadillo TGATTAGTCATTTATCACACCTGGCAGCCTTGCTGAACTATAATTACTCTTCTCTTATCC 116
fugu TTATTAGTCATTTATCACACCTGGCAGCCTCAGTGAACCATAATTACTCTTCCCTTATCC 120
tetraodon TTATTAGTCATTTATCACACCTGGCAGCCTCAGTGAACCATAATTACTCTTCCCTTATCC 120
stickleback TTATTAGTCATTTATCACACCTGGCAGCCTCACTGAACCATAATTACTCTTCCCTTATCC 120
zfish TTATTAGTCATTTATCACACCTGCCAGCCTGGCTGAACCATAATTACTCTGGCCTTATCC 108
 * **** **************** *** ** *********** ******
frog T-GGTGCACTGTCATGTCCCAGAGAAGAG- 145
chicken C-GCTGCACTGTCATGTCCAAGAGGAGA-- 144
opossum T-CCTGCGCTGTCATGTCCGAGAGGAGA-- 144
cow TGCCTG-GCTGTCATGTCCGAGAGGAGAG- 145
cat T-ATTGCACTGTCATGTCCGAGAGGAGAG- 145
dog T-CTTGCACTGTCATGTCCGAGAGGAGAG- 145
bat T-GC-GCACTGTCATGTCCGAGAGGAGAG- 144
human A-GCCGCACTGTCATGTCCGAGAGGAGAG- 145
chimp A-GCCGCACTGTCATGTCCGAGAGGAGAG- 145
macaque T-GCCGCACTGTCATGTCCGAGAGGAGA-- 144
rabbit T-GCCGTACTGTCATGTCCGAGAGGAGAG- 145
bushbaby T-GCCGCACTGTCATGTCCGAGAGGAGAG- 145
rat TAACGGCACTGTCATGTCCGAGAGGAGAG- 146
mouse TAACGGCACTGTCATGTCCGAGAGGAGAG- 146
squirrel CGCCG-CACTGTCATGTCCGAGAGGAGAG- 143
armadillo C-GCCGCACTGTCATGTCCGAGAGGAGAG- 144
fugu CCCT-GCACTGTCATGTCGGTGAGAGCGAG 149
tetraodon CCCT-GCACTGTCATGTCGGTGAGAGCAAG 149
stickleback CCCC-ACACTGTCATGTCCATGAGAGGGAG 149
zfish CCTTCGTGCTGTCATGTC------------ 126
 **********

## Slide 34
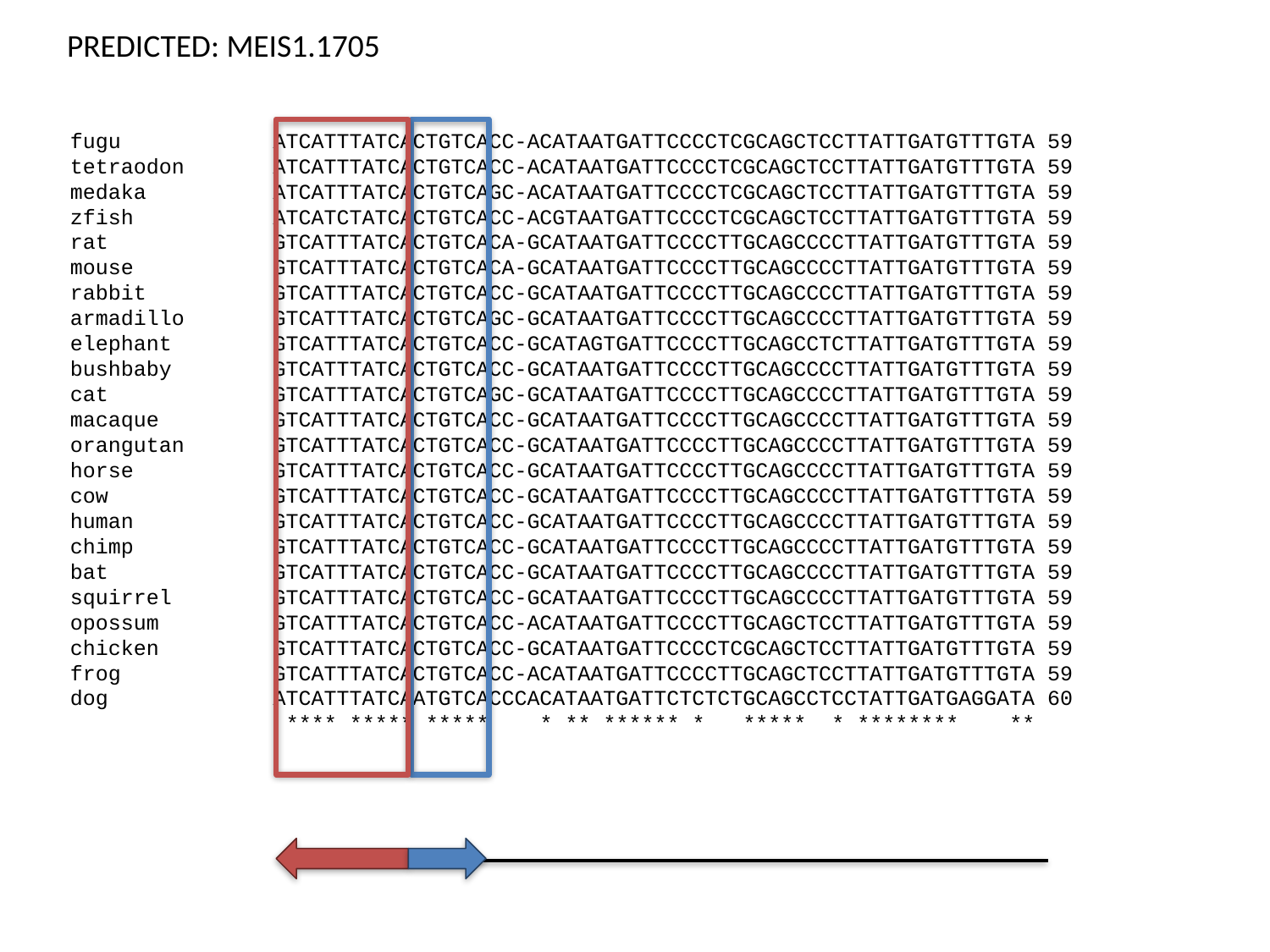

PREDICTED: MEIS1.1705
fugu ATCATTTATCACTGTCACC-ACATAATGATTCCCCTCGCAGCTCCTTATTGATGTTTGTA 59
tetraodon ATCATTTATCACTGTCACC-ACATAATGATTCCCCTCGCAGCTCCTTATTGATGTTTGTA 59
medaka ATCATTTATCACTGTCAGC-ACATAATGATTCCCCTCGCAGCTCCTTATTGATGTTTGTA 59
zfish ATCATCTATCACTGTCACC-ACGTAATGATTCCCCTCGCAGCTCCTTATTGATGTTTGTA 59
rat GTCATTTATCACTGTCACA-GCATAATGATTCCCCTTGCAGCCCCTTATTGATGTTTGTA 59
mouse GTCATTTATCACTGTCACA-GCATAATGATTCCCCTTGCAGCCCCTTATTGATGTTTGTA 59
rabbit GTCATTTATCACTGTCACC-GCATAATGATTCCCCTTGCAGCCCCTTATTGATGTTTGTA 59
armadillo GTCATTTATCACTGTCAGC-GCATAATGATTCCCCTTGCAGCCCCTTATTGATGTTTGTA 59
elephant GTCATTTATCACTGTCACC-GCATAGTGATTCCCCTTGCAGCCTCTTATTGATGTTTGTA 59
bushbaby GTCATTTATCACTGTCACC-GCATAATGATTCCCCTTGCAGCCCCTTATTGATGTTTGTA 59
cat GTCATTTATCACTGTCAGC-GCATAATGATTCCCCTTGCAGCCCCTTATTGATGTTTGTA 59
macaque GTCATTTATCACTGTCACC-GCATAATGATTCCCCTTGCAGCCCCTTATTGATGTTTGTA 59
orangutan GTCATTTATCACTGTCACC-GCATAATGATTCCCCTTGCAGCCCCTTATTGATGTTTGTA 59
horse GTCATTTATCACTGTCACC-GCATAATGATTCCCCTTGCAGCCCCTTATTGATGTTTGTA 59
cow GTCATTTATCACTGTCACC-GCATAATGATTCCCCTTGCAGCCCCTTATTGATGTTTGTA 59
human GTCATTTATCACTGTCACC-GCATAATGATTCCCCTTGCAGCCCCTTATTGATGTTTGTA 59
chimp GTCATTTATCACTGTCACC-GCATAATGATTCCCCTTGCAGCCCCTTATTGATGTTTGTA 59
bat GTCATTTATCACTGTCACC-GCATAATGATTCCCCTTGCAGCCCCTTATTGATGTTTGTA 59
squirrel GTCATTTATCACTGTCACC-GCATAATGATTCCCCTTGCAGCCCCTTATTGATGTTTGTA 59
opossum GTCATTTATCACTGTCACC-ACATAATGATTCCCCTTGCAGCTCCTTATTGATGTTTGTA 59
chicken GTCATTTATCACTGTCACC-GCATAATGATTCCCCTCGCAGCTCCTTATTGATGTTTGTA 59
frog GTCATTTATCACTGTCACC-ACATAATGATTCCCCTTGCAGCTCCTTATTGATGTTTGTA 59
dog ATCATTTATCAATGTCACCCACATAATGATTCTCTCTGCAGCCTCCTATTGATGAGGATA 60
 **** ***** ***** * ** ****** * ***** * ******** **

## Slide 35
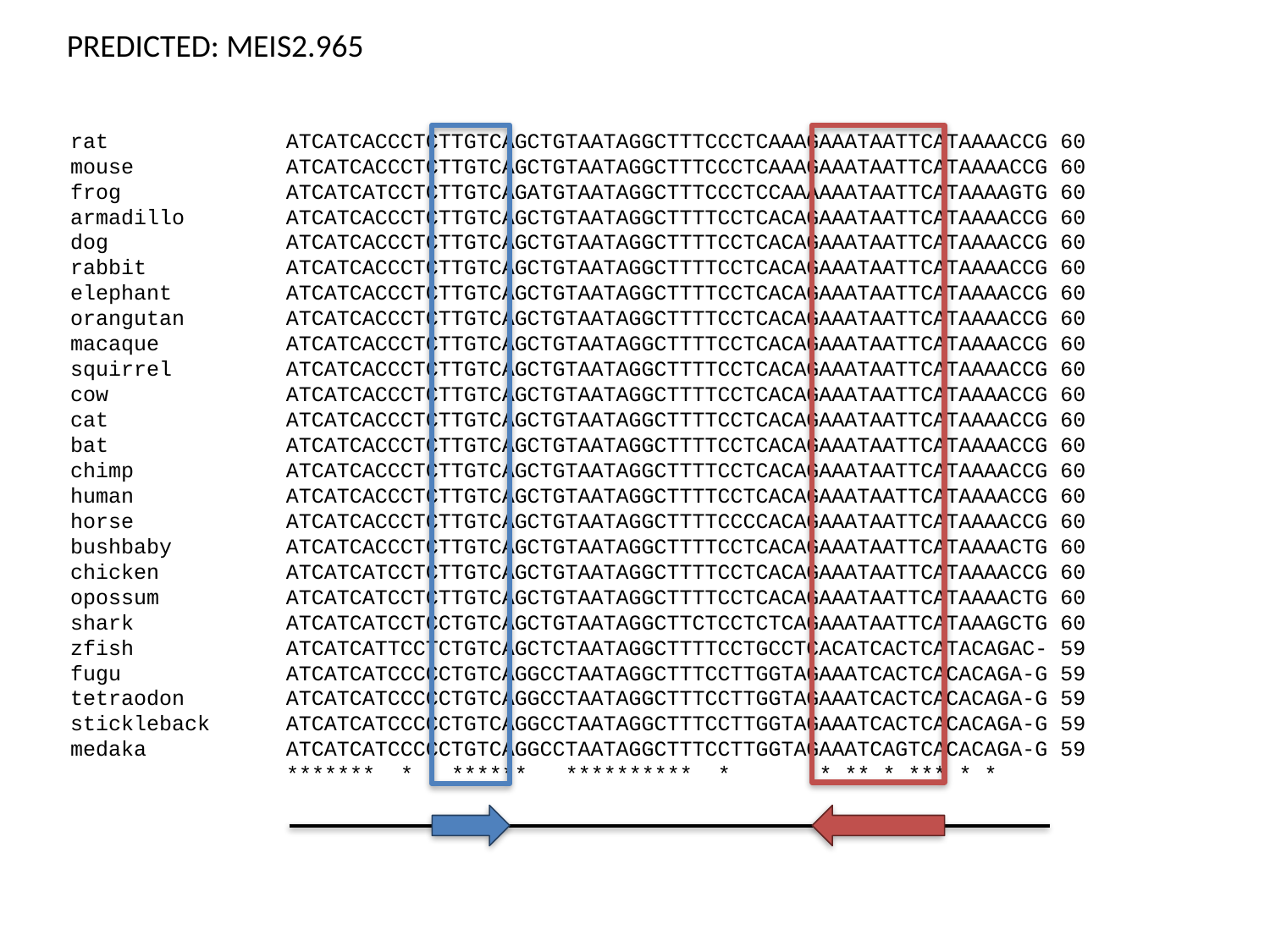

PREDICTED: MEIS2.965
rat ATCATCACCCTCTTGTCAGCTGTAATAGGCTTTCCCTCAAAGAAATAATTCATAAAACCG 60
mouse ATCATCACCCTCTTGTCAGCTGTAATAGGCTTTCCCTCAAAGAAATAATTCATAAAACCG 60
frog ATCATCATCCTCTTGTCAGATGTAATAGGCTTTCCCTCCAAAAAATAATTCATAAAAGTG 60
armadillo ATCATCACCCTCTTGTCAGCTGTAATAGGCTTTTCCTCACAGAAATAATTCATAAAACCG 60
dog ATCATCACCCTCTTGTCAGCTGTAATAGGCTTTTCCTCACAGAAATAATTCATAAAACCG 60
rabbit ATCATCACCCTCTTGTCAGCTGTAATAGGCTTTTCCTCACAGAAATAATTCATAAAACCG 60
elephant ATCATCACCCTCTTGTCAGCTGTAATAGGCTTTTCCTCACAGAAATAATTCATAAAACCG 60
orangutan ATCATCACCCTCTTGTCAGCTGTAATAGGCTTTTCCTCACAGAAATAATTCATAAAACCG 60
macaque ATCATCACCCTCTTGTCAGCTGTAATAGGCTTTTCCTCACAGAAATAATTCATAAAACCG 60
squirrel ATCATCACCCTCTTGTCAGCTGTAATAGGCTTTTCCTCACAGAAATAATTCATAAAACCG 60
cow ATCATCACCCTCTTGTCAGCTGTAATAGGCTTTTCCTCACAGAAATAATTCATAAAACCG 60
cat ATCATCACCCTCTTGTCAGCTGTAATAGGCTTTTCCTCACAGAAATAATTCATAAAACCG 60
bat ATCATCACCCTCTTGTCAGCTGTAATAGGCTTTTCCTCACAGAAATAATTCATAAAACCG 60
chimp ATCATCACCCTCTTGTCAGCTGTAATAGGCTTTTCCTCACAGAAATAATTCATAAAACCG 60
human ATCATCACCCTCTTGTCAGCTGTAATAGGCTTTTCCTCACAGAAATAATTCATAAAACCG 60
horse ATCATCACCCTCTTGTCAGCTGTAATAGGCTTTTCCCCACAGAAATAATTCATAAAACCG 60
bushbaby ATCATCACCCTCTTGTCAGCTGTAATAGGCTTTTCCTCACAGAAATAATTCATAAAACTG 60
chicken ATCATCATCCTCTTGTCAGCTGTAATAGGCTTTTCCTCACAGAAATAATTCATAAAACCG 60
opossum ATCATCATCCTCTTGTCAGCTGTAATAGGCTTTTCCTCACAGAAATAATTCATAAAACTG 60
shark ATCATCATCCTCCTGTCAGCTGTAATAGGCTTCTCCTCTCAGAAATAATTCATAAAGCTG 60
zfish ATCATCATTCCTCTGTCAGCTCTAATAGGCTTTTCCTGCCTCACATCACTCATACAGAC- 59
fugu ATCATCATCCCCCTGTCAGGCCTAATAGGCTTTCCTTGGTAGAAATCACTCACACAGA-G 59
tetraodon ATCATCATCCCCCTGTCAGGCCTAATAGGCTTTCCTTGGTAGAAATCACTCACACAGA-G 59
stickleback ATCATCATCCCCCTGTCAGGCCTAATAGGCTTTCCTTGGTAGAAATCACTCACACAGA-G 59
medaka ATCATCATCCCCCTGTCAGGCCTAATAGGCTTTCCTTGGTAGAAATCAGTCACACAGA-G 59
 ******* * ****** ********** * * ** * *** * *

## Slide 36
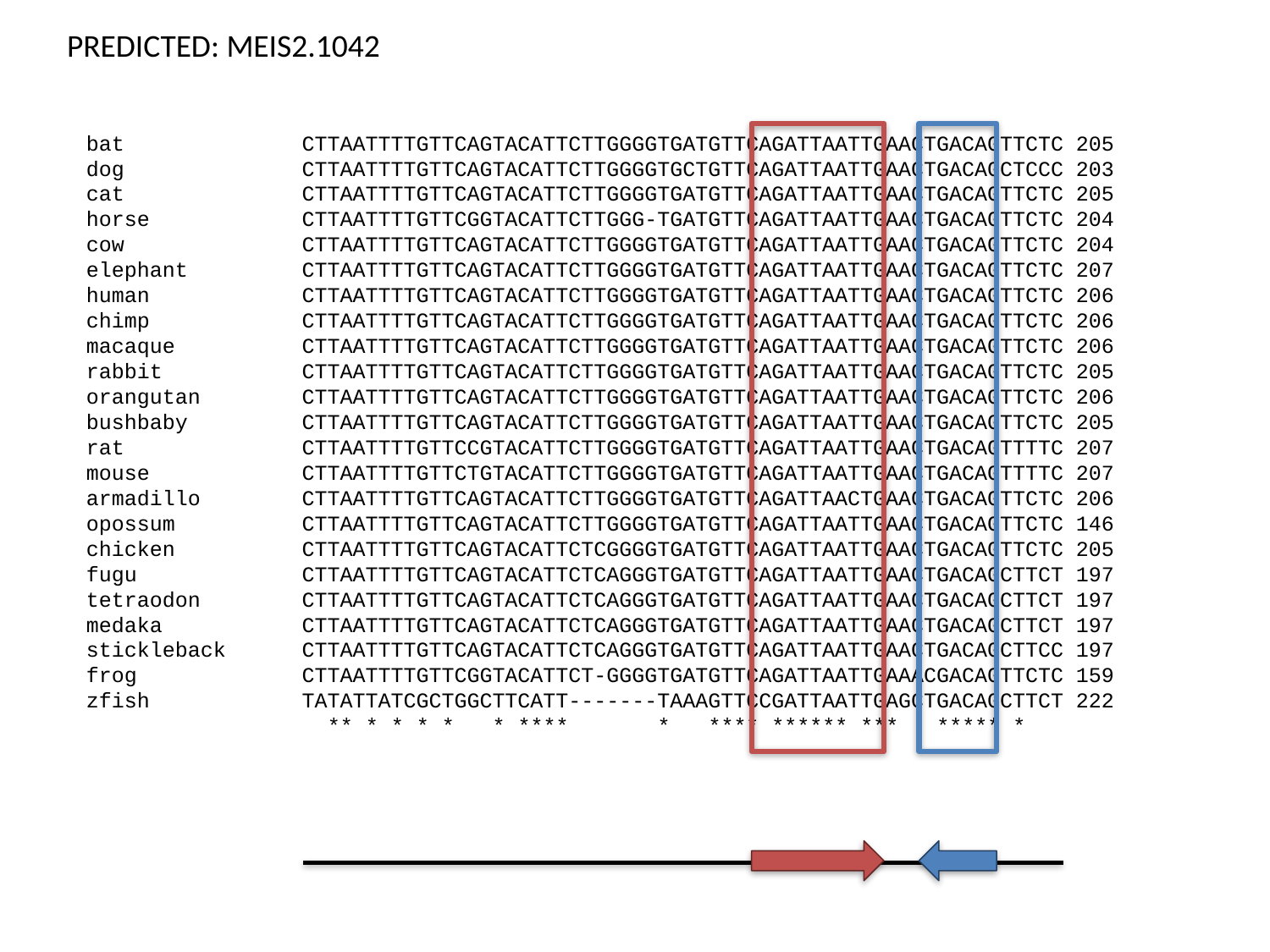

PREDICTED: MEIS2.1042
bat CTTAATTTTGTTCAGTACATTCTTGGGGTGATGTTCAGATTAATTGAACTGACAGTTCTC 205
dog CTTAATTTTGTTCAGTACATTCTTGGGGTGCTGTTCAGATTAATTGAACTGACAGCTCCC 203
cat CTTAATTTTGTTCAGTACATTCTTGGGGTGATGTTCAGATTAATTGAACTGACAGTTCTC 205
horse CTTAATTTTGTTCGGTACATTCTTGGG-TGATGTTCAGATTAATTGAACTGACAGTTCTC 204
cow CTTAATTTTGTTCAGTACATTCTTGGGGTGATGTTCAGATTAATTGAACTGACAGTTCTC 204
elephant CTTAATTTTGTTCAGTACATTCTTGGGGTGATGTTCAGATTAATTGAACTGACAGTTCTC 207
human CTTAATTTTGTTCAGTACATTCTTGGGGTGATGTTCAGATTAATTGAACTGACAGTTCTC 206
chimp CTTAATTTTGTTCAGTACATTCTTGGGGTGATGTTCAGATTAATTGAACTGACAGTTCTC 206
macaque CTTAATTTTGTTCAGTACATTCTTGGGGTGATGTTCAGATTAATTGAACTGACAGTTCTC 206
rabbit CTTAATTTTGTTCAGTACATTCTTGGGGTGATGTTCAGATTAATTGAACTGACAGTTCTC 205
orangutan CTTAATTTTGTTCAGTACATTCTTGGGGTGATGTTCAGATTAATTGAACTGACAGTTCTC 206
bushbaby CTTAATTTTGTTCAGTACATTCTTGGGGTGATGTTCAGATTAATTGAACTGACAGTTCTC 205
rat CTTAATTTTGTTCCGTACATTCTTGGGGTGATGTTCAGATTAATTGAACTGACAGTTTTC 207
mouse CTTAATTTTGTTCTGTACATTCTTGGGGTGATGTTCAGATTAATTGAACTGACAGTTTTC 207
armadillo CTTAATTTTGTTCAGTACATTCTTGGGGTGATGTTCAGATTAACTGAACTGACAGTTCTC 206
opossum CTTAATTTTGTTCAGTACATTCTTGGGGTGATGTTCAGATTAATTGAACTGACAGTTCTC 146
chicken CTTAATTTTGTTCAGTACATTCTCGGGGTGATGTTCAGATTAATTGAACTGACAGTTCTC 205
fugu CTTAATTTTGTTCAGTACATTCTCAGGGTGATGTTCAGATTAATTGAACTGACAGCTTCT 197
tetraodon CTTAATTTTGTTCAGTACATTCTCAGGGTGATGTTCAGATTAATTGAACTGACAGCTTCT 197
medaka CTTAATTTTGTTCAGTACATTCTCAGGGTGATGTTCAGATTAATTGAACTGACAGCTTCT 197
stickleback CTTAATTTTGTTCAGTACATTCTCAGGGTGATGTTCAGATTAATTGAACTGACAGCTTCC 197
frog CTTAATTTTGTTCGGTACATTCT-GGGGTGATGTTCAGATTAATTGAAACGACAGTTCTC 159
zfish TATATTATCGCTGGCTTCATT-------TAAAGTTCCGATTAATTGAGCTGACAGCTTCT 222
 ** * * * * * **** * **** ****** *** ***** *

## Slide 37
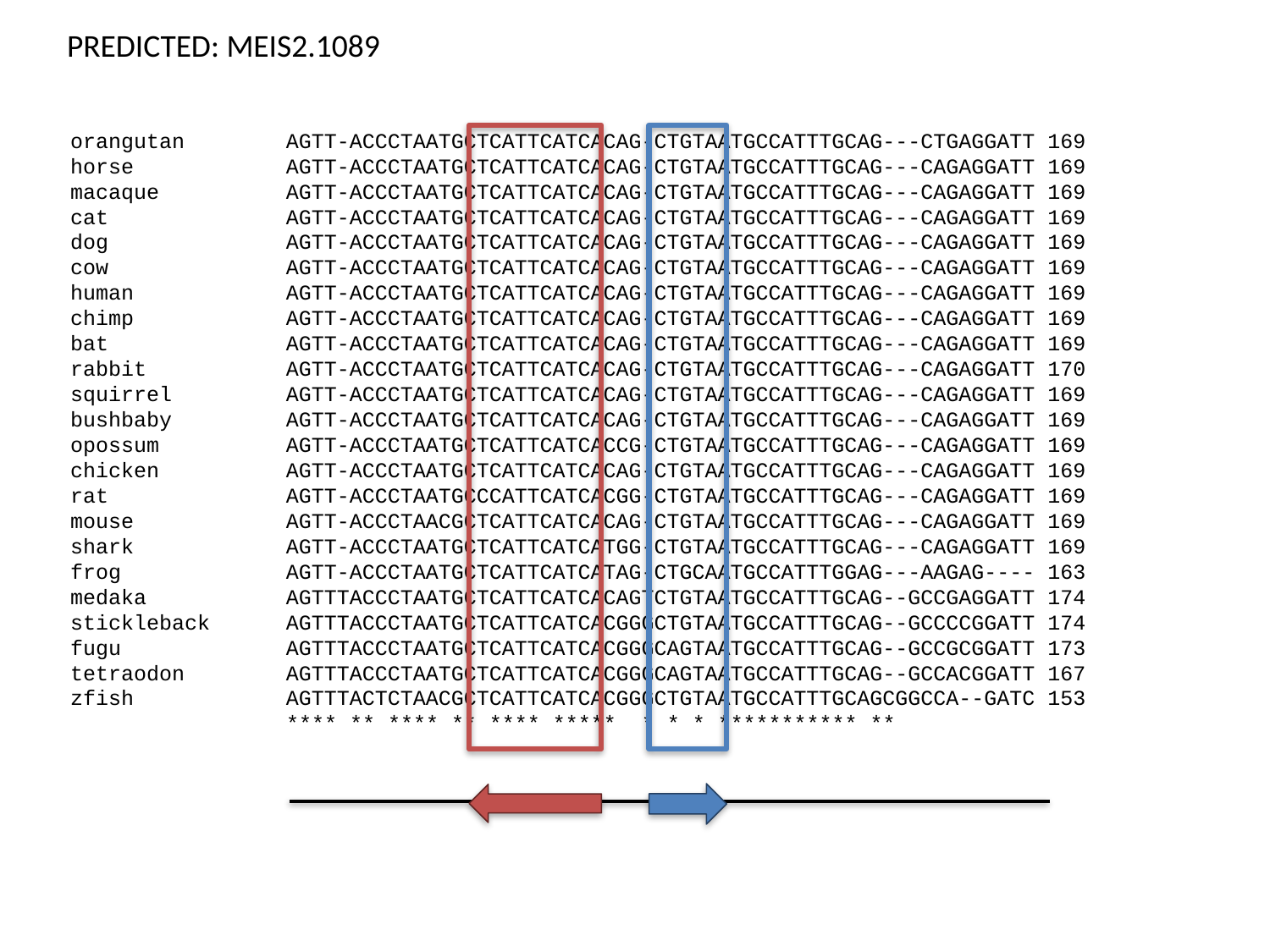

PREDICTED: MEIS2.1089
orangutan AGTT-ACCCTAATGCTCATTCATCACAG-CTGTAATGCCATTTGCAG---CTGAGGATT 169
horse AGTT-ACCCTAATGCTCATTCATCACAG-CTGTAATGCCATTTGCAG---CAGAGGATT 169
macaque AGTT-ACCCTAATGCTCATTCATCACAG-CTGTAATGCCATTTGCAG---CAGAGGATT 169
cat AGTT-ACCCTAATGCTCATTCATCACAG-CTGTAATGCCATTTGCAG---CAGAGGATT 169
dog AGTT-ACCCTAATGCTCATTCATCACAG-CTGTAATGCCATTTGCAG---CAGAGGATT 169
cow AGTT-ACCCTAATGCTCATTCATCACAG-CTGTAATGCCATTTGCAG---CAGAGGATT 169
human AGTT-ACCCTAATGCTCATTCATCACAG-CTGTAATGCCATTTGCAG---CAGAGGATT 169
chimp AGTT-ACCCTAATGCTCATTCATCACAG-CTGTAATGCCATTTGCAG---CAGAGGATT 169
bat AGTT-ACCCTAATGCTCATTCATCACAG-CTGTAATGCCATTTGCAG---CAGAGGATT 169
rabbit AGTT-ACCCTAATGCTCATTCATCACAG-CTGTAATGCCATTTGCAG---CAGAGGATT 170
squirrel AGTT-ACCCTAATGCTCATTCATCACAG-CTGTAATGCCATTTGCAG---CAGAGGATT 169
bushbaby AGTT-ACCCTAATGCTCATTCATCACAG-CTGTAATGCCATTTGCAG---CAGAGGATT 169
opossum AGTT-ACCCTAATGCTCATTCATCACCG-CTGTAATGCCATTTGCAG---CAGAGGATT 169
chicken AGTT-ACCCTAATGCTCATTCATCACAG-CTGTAATGCCATTTGCAG---CAGAGGATT 169
rat AGTT-ACCCTAATGCCCATTCATCACGG-CTGTAATGCCATTTGCAG---CAGAGGATT 169
mouse AGTT-ACCCTAACGCTCATTCATCACAG-CTGTAATGCCATTTGCAG---CAGAGGATT 169
shark AGTT-ACCCTAATGCTCATTCATCATGG-CTGTAATGCCATTTGCAG---CAGAGGATT 169
frog AGTT-ACCCTAATGCTCATTCATCATAG-CTGCAATGCCATTTGGAG---AAGAG---- 163
medaka AGTTTACCCTAATGCTCATTCATCACAGTCTGTAATGCCATTTGCAG--GCCGAGGATT 174
stickleback AGTTTACCCTAATGCTCATTCATCACGGGCTGTAATGCCATTTGCAG--GCCCCGGATT 174
fugu AGTTTACCCTAATGCTCATTCATCACGGGCAGTAATGCCATTTGCAG--GCCGCGGATT 173
tetraodon AGTTTACCCTAATGCTCATTCATCACGGGCAGTAATGCCATTTGCAG--GCCACGGATT 167
zfish AGTTTACTCTAACGCTCATTCATCACGGGCTGTAATGCCATTTGCAGCGGCCA--GATC 153
 **** ** **** ** **** ***** * * * *********** **

## Slide 38
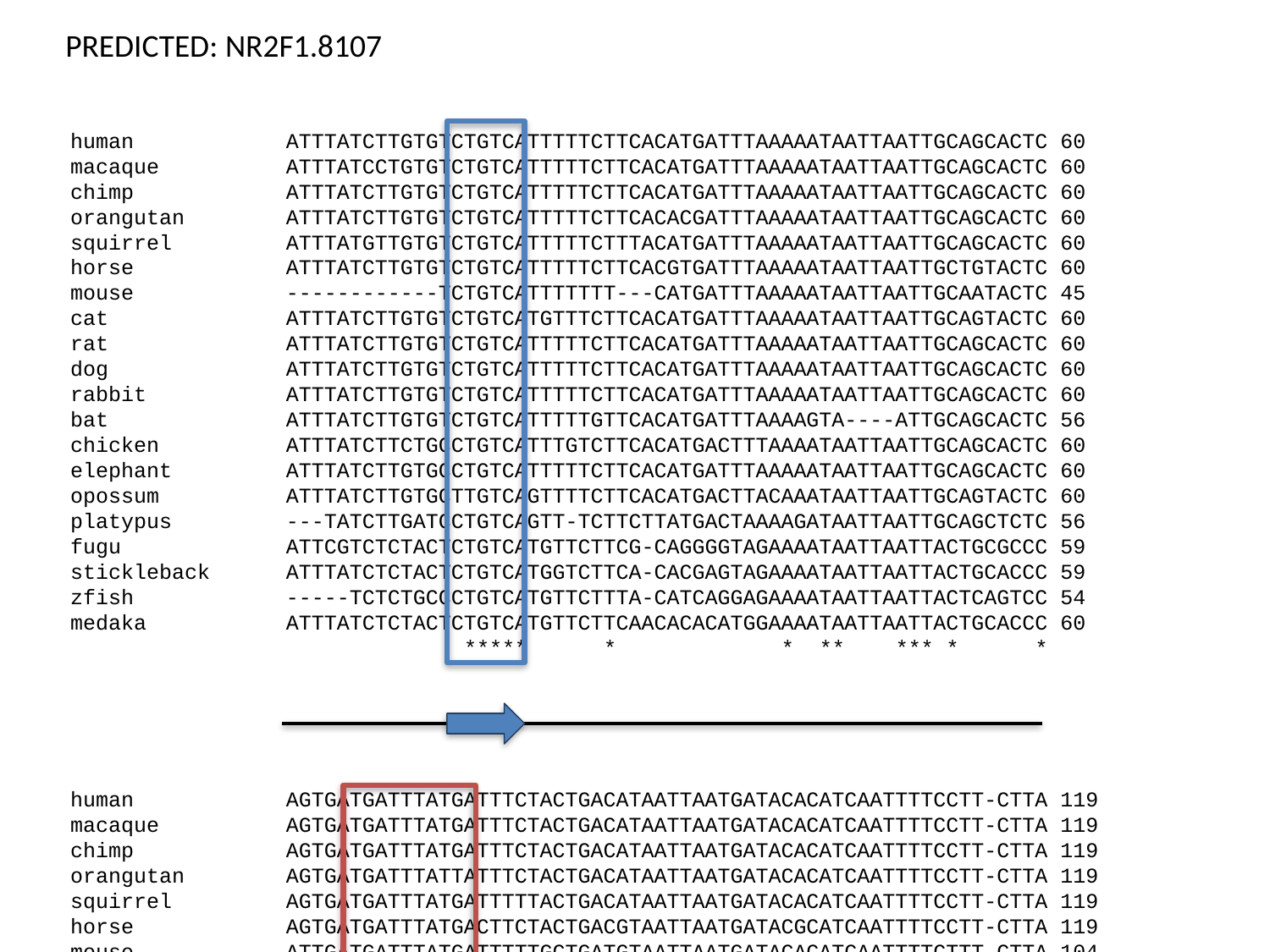

PREDICTED: NR2F1.8107
human ATTTATCTTGTGTCTGTCATTTTTCTTCACATGATTTAAAAATAATTAATTGCAGCACTC 60
macaque ATTTATCCTGTGTCTGTCATTTTTCTTCACATGATTTAAAAATAATTAATTGCAGCACTC 60
chimp ATTTATCTTGTGTCTGTCATTTTTCTTCACATGATTTAAAAATAATTAATTGCAGCACTC 60
orangutan ATTTATCTTGTGTCTGTCATTTTTCTTCACACGATTTAAAAATAATTAATTGCAGCACTC 60
squirrel ATTTATGTTGTGTCTGTCATTTTTCTTTACATGATTTAAAAATAATTAATTGCAGCACTC 60
horse ATTTATCTTGTGTCTGTCATTTTTCTTCACGTGATTTAAAAATAATTAATTGCTGTACTC 60
mouse ------------TCTGTCATTTTTTT---CATGATTTAAAAATAATTAATTGCAATACTC 45
cat ATTTATCTTGTGTCTGTCATGTTTCTTCACATGATTTAAAAATAATTAATTGCAGTACTC 60
rat ATTTATCTTGTGTCTGTCATTTTTCTTCACATGATTTAAAAATAATTAATTGCAGCACTC 60
dog ATTTATCTTGTGTCTGTCATTTTTCTTCACATGATTTAAAAATAATTAATTGCAGCACTC 60
rabbit ATTTATCTTGTGTCTGTCATTTTTCTTCACATGATTTAAAAATAATTAATTGCAGCACTC 60
bat ATTTATCTTGTGTCTGTCATTTTTGTTCACATGATTTAAAAGTA----ATTGCAGCACTC 56
chicken ATTTATCTTCTGCCTGTCATTTGTCTTCACATGACTTTAAAATAATTAATTGCAGCACTC 60
elephant ATTTATCTTGTGCCTGTCATTTTTCTTCACATGATTTAAAAATAATTAATTGCAGCACTC 60
opossum ATTTATCTTGTGCTTGTCAGTTTTCTTCACATGACTTACAAATAATTAATTGCAGTACTC 60
platypus ---TATCTTGATCCTGTCAGTT-TCTTCTTATGACTAAAAGATAATTAATTGCAGCTCTC 56
fugu ATTCGTCTCTACTCTGTCATGTTCTTCG-CAGGGGTAGAAAATAATTAATTACTGCGCCC 59
stickleback ATTTATCTCTACTCTGTCATGGTCTTCA-CACGAGTAGAAAATAATTAATTACTGCACCC 59
zfish -----TCTCTGCCCTGTCATGTTCTTTA-CATCAGGAGAAAATAATTAATTACTCAGTCC 54
medaka ATTTATCTCTACTCTGTCATGTTCTTCAACACACATGGAAAATAATTAATTACTGCACCC 60
 ***** * * ** *** * *
human AGTGATGATTTATGATTTCTACTGACATAATTAATGATACACATCAATTTTCCTT-CTTA 119
macaque AGTGATGATTTATGATTTCTACTGACATAATTAATGATACACATCAATTTTCCTT-CTTA 119
chimp AGTGATGATTTATGATTTCTACTGACATAATTAATGATACACATCAATTTTCCTT-CTTA 119
orangutan AGTGATGATTTATTATTTCTACTGACATAATTAATGATACACATCAATTTTCCTT-CTTA 119
squirrel AGTGATGATTTATGATTTTTACTGACATAATTAATGATACACATCAATTTTCCTT-CTTA 119
horse AGTGATGATTTATGACTTCTACTGACGTAATTAATGATACGCATCAATTTTCCTT-CTTA 119
mouse ATTGATGATTTATGATTTTTGCTGATGTAATTAATGATACACATCAATTTTCTTT-CTTA 104
cat AGTGATGATTTATGATTTCTACTGACGTAATTAATGATACACATCAATTTTCCTT-CTTA 119
rat AGTGATGATTTATGATTTCTACTGACGTAATTAATGATACACATCAATTTTCCTT-CTTA 119
dog AGTGATGATTTATGATTTCTACTGACGTAATTAATGATACACATCAATTTTCCTT-CTTA 119
rabbit AGCAATGATTCATGCTTTCTACTGACGTAATTAATGATACACATCAATTTTCCTT-CTTA 119
bat AGTGATGATTTATGATTTCTACTGACGTAATTAATGATACACATCAATTTTTCTT-CTTA 115
chicken AGAGATGATTTATGATTTCTACTGACGTAATTAATGATACACATCAATTTTTCTT-GTTA 119
elephant AGAGGTGATTTATGATTTCTACTGACGTAATTAATGATACACATCAATTTTCTTC-CTTA 119
opossum AGTGATGATTTATGATTTCTACTGACATAATTAATGATACACATCCATTTTCCTTTCTTA 120
platypus --TGGTAATTTATGATTTCTACTGACAAAATTAATGCCATACATCAATTTTCTTA-CTT- 112
fugu AGTGATGATTTATGATAT--ACTGGCATAATTAATGATACACATCAATTTTCCTG-CTTA 116
stickleback AGTGATGATTTATGATATT-ACTGACATAATTAAGGATACACATCAATTTTCCTG-CTTA 117
zfish ACTCATGATTTATGATATT-ACTGACATAATTAATGAAACACATCAATTTTCCTG-TTTA 112
medaka AGTGATGATTTATGATATC-ACAGACGTAATTAATGATACACATCAATTTTCCTG-CTAA 118
 * *** ** * * * ****** * * **** ***** * *

## Slide 39
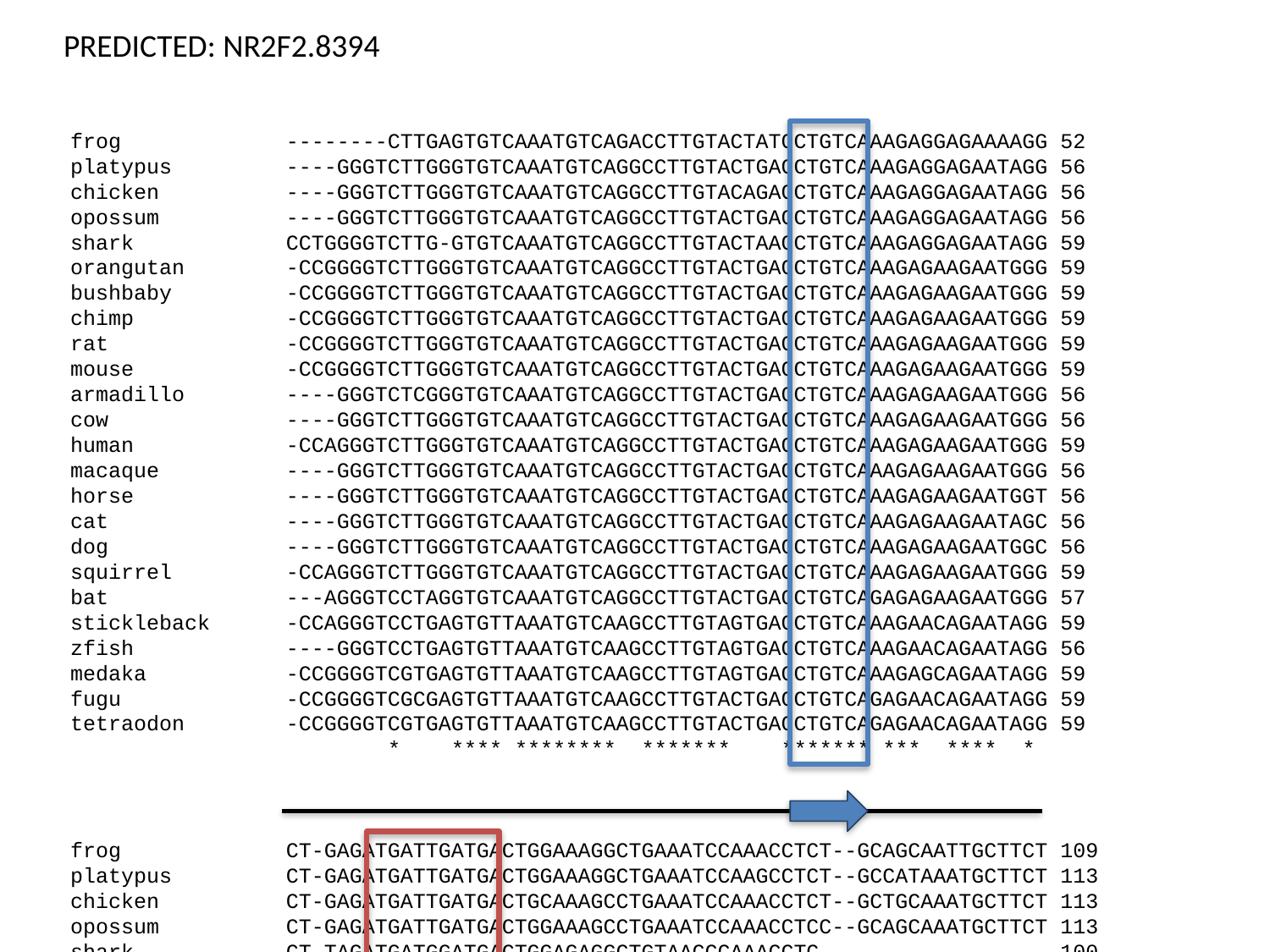

PREDICTED: NR2F2.8394
frog --------CTTGAGTGTCAAATGTCAGACCTTGTACTATGCTGTCAAAGAGGAGAAAAGG 52
platypus ----GGGTCTTGGGTGTCAAATGTCAGGCCTTGTACTGAGCTGTCAAAGAGGAGAATAGG 56
chicken ----GGGTCTTGGGTGTCAAATGTCAGGCCTTGTACAGAGCTGTCAAAGAGGAGAATAGG 56
opossum ----GGGTCTTGGGTGTCAAATGTCAGGCCTTGTACTGAGCTGTCAAAGAGGAGAATAGG 56
shark CCTGGGGTCTTG-GTGTCAAATGTCAGGCCTTGTACTAAGCTGTCAAAGAGGAGAATAGG 59
orangutan -CCGGGGTCTTGGGTGTCAAATGTCAGGCCTTGTACTGAGCTGTCAAAGAGAAGAATGGG 59
bushbaby -CCGGGGTCTTGGGTGTCAAATGTCAGGCCTTGTACTGAGCTGTCAAAGAGAAGAATGGG 59
chimp -CCGGGGTCTTGGGTGTCAAATGTCAGGCCTTGTACTGAGCTGTCAAAGAGAAGAATGGG 59
rat -CCGGGGTCTTGGGTGTCAAATGTCAGGCCTTGTACTGAGCTGTCAAAGAGAAGAATGGG 59
mouse -CCGGGGTCTTGGGTGTCAAATGTCAGGCCTTGTACTGAGCTGTCAAAGAGAAGAATGGG 59
armadillo ----GGGTCTCGGGTGTCAAATGTCAGGCCTTGTACTGAGCTGTCAAAGAGAAGAATGGG 56
cow ----GGGTCTTGGGTGTCAAATGTCAGGCCTTGTACTGAGCTGTCAAAGAGAAGAATGGG 56
human -CCAGGGTCTTGGGTGTCAAATGTCAGGCCTTGTACTGAGCTGTCAAAGAGAAGAATGGG 59
macaque ----GGGTCTTGGGTGTCAAATGTCAGGCCTTGTACTGAGCTGTCAAAGAGAAGAATGGG 56
horse ----GGGTCTTGGGTGTCAAATGTCAGGCCTTGTACTGAGCTGTCAAAGAGAAGAATGGT 56
cat ----GGGTCTTGGGTGTCAAATGTCAGGCCTTGTACTGAGCTGTCAAAGAGAAGAATAGC 56
dog ----GGGTCTTGGGTGTCAAATGTCAGGCCTTGTACTGAGCTGTCAAAGAGAAGAATGGC 56
squirrel -CCAGGGTCTTGGGTGTCAAATGTCAGGCCTTGTACTGAGCTGTCAAAGAGAAGAATGGG 59
bat ---AGGGTCCTAGGTGTCAAATGTCAGGCCTTGTACTGAGCTGTCAGAGAGAAGAATGGG 57
stickleback -CCAGGGTCCTGAGTGTTAAATGTCAAGCCTTGTAGTGAGCTGTCAAAGAACAGAATAGG 59
zfish ----GGGTCCTGAGTGTTAAATGTCAAGCCTTGTAGTGAGCTGTCAAAGAACAGAATAGG 56
medaka -CCGGGGTCGTGAGTGTTAAATGTCAAGCCTTGTAGTGAGCTGTCAAAGAGCAGAATAGG 59
fugu -CCGGGGTCGCGAGTGTTAAATGTCAAGCCTTGTACTGAGCTGTCAGAGAACAGAATAGG 59
tetraodon -CCGGGGTCGTGAGTGTTAAATGTCAAGCCTTGTACTGAGCTGTCAGAGAACAGAATAGG 59
 * **** ******** ******* ******* *** **** *
frog CT-GAGATGATTGATGACTGGAAAGGCTGAAATCCAAACCTCT--GCAGCAATTGCTTCT 109
platypus CT-GAGATGATTGATGACTGGAAAGGCTGAAATCCAAGCCTCT--GCCATAAATGCTTCT 113
chicken CT-GAGATGATTGATGACTGCAAAGCCTGAAATCCAAACCTCT--GCTGCAAATGCTTCT 113
opossum CT-GAGATGATTGATGACTGGAAAGCCTGAAATCCAAACCTCC--GCAGCAAATGCTTCT 113
shark CT-TAGATGATGGATGACTGGAGAGGCTGTAACCCAAACCTC------------------ 100
orangutan CT-GAGATGATTGATGACTGGGAGGCCTGAAACCCAAACCTCC--ACTGCAAATGCTTCT 116
bushbaby CT-GAGATGATTGATGACTGGGAGGCCTGAAACCCAAACCTCC--ACTGCAAATGCTTCT 116
chimp CT-GAGATGATTGATGACTGGGAGGCCTGAAACCCAAACCTCC--ACTGCAAATGCTTCT 116
rat CT-GTGATGATTGATGACTGGGAGGCCTGAAACTCAAACCTCC--ACTTCGAATGCTTCT 116
mouse CT-GTGATGATTGATGACTGGGAGGCCTGAAACCCAAACCTCC--ACTGCAAATGCTTCT 116
armadillo CA-GAGATGATTGATGACTGGGAGGCCTGAAACTCAAACCTCC--AGTGCAAATGCTTCT 113
cow CT-GAGATGATTGATGACTGGGAGGCCTGAAACCCAAACCTCC--ACTGCAAATGCTTCT 113
human CT-GAGATGATTGATGACTGGGAGGCCTGAAACCCAAACCTCC--ACTGCAAATGCTTCT 116
macaque TT-GAGATGATTGATGACTGGGAGGCCTGAAACCCAAACCTCC--ACTGCAAATGCTTCT 113
horse CT-GAGATGATTGATGACTGGGAGGCCTGAAACCCAAACCTCC--ACTGCAAATGCTTCT 113
cat CT-GGGATGATTGATGACGGGGAGGCTTGAAACCCACACCTCC--ACTGAAAATGCTGTT 113
dog CT-GGGATGATTGATGACTGGGAGGCTTGAAACCCAAACCTCC--ACTGCAAATGCTTCT 113
squirrel CT-GAGATGATTGATGACTGAGAAGTCTGAAACCCAAACCTCC--ACTGCAAATGCTTCT 116
bat CC-GAGATGATTGATGACTGGGAGGCCTGAAACCCAAACCTCC--GCTGCAAATGCTTCT 114
stickleback CCCGGCATGATTGATGACTGGAGAGCCTGAAACCAAAACCTCCCAATTGCAAATGCTACC 119
zfish CCCGGCATGATTGATGACTGGAGGGCCCAAAACCCAAACC-CCAGATTGCAAATGCTACC 115
medaka CCCGGGATGATTGATGACTGGAGAGCCTGAAACCAAAACCTCCCAATTGCAAATGCTACC 119
fugu CTCGGGATGATTGATGACCGCGGAGTCTGAAACCAAAACCTCCCAATTGCAAATGCTACC 119
tetraodon GCCGGCATGATTGATGACTGCGGAGTCTGAAACCAAAACCTCCCAATTGCAAATGCTACC 119
 ***** ****** * * ** * ** *

## Slide 40
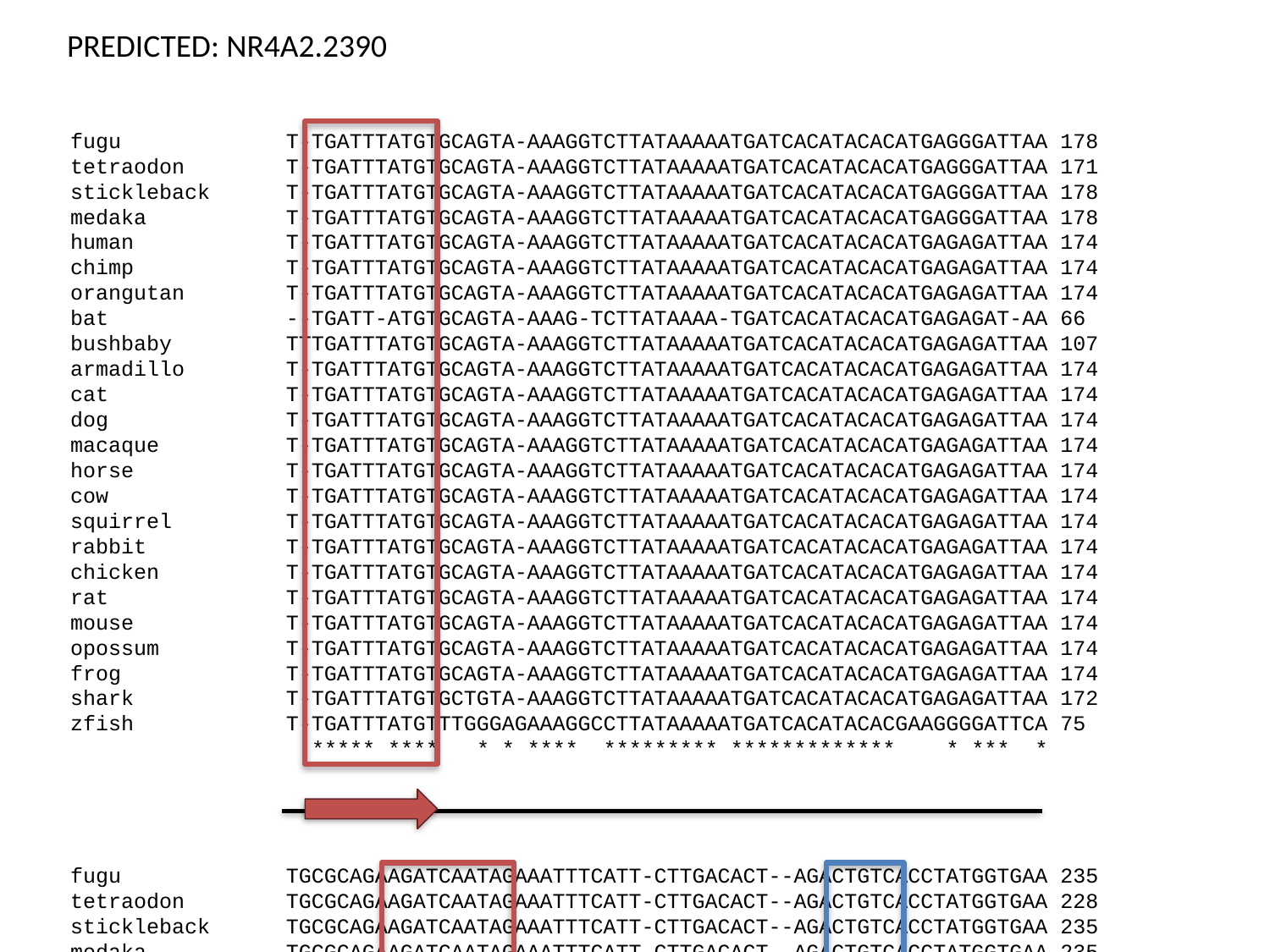

PREDICTED: NR4A2.2390
fugu T-TGATTTATGTGCAGTA-AAAGGTCTTATAAAAATGATCACATACACATGAGGGATTAA 178
tetraodon T-TGATTTATGTGCAGTA-AAAGGTCTTATAAAAATGATCACATACACATGAGGGATTAA 171
stickleback T-TGATTTATGTGCAGTA-AAAGGTCTTATAAAAATGATCACATACACATGAGGGATTAA 178
medaka T-TGATTTATGTGCAGTA-AAAGGTCTTATAAAAATGATCACATACACATGAGGGATTAA 178
human T-TGATTTATGTGCAGTA-AAAGGTCTTATAAAAATGATCACATACACATGAGAGATTAA 174
chimp T-TGATTTATGTGCAGTA-AAAGGTCTTATAAAAATGATCACATACACATGAGAGATTAA 174
orangutan T-TGATTTATGTGCAGTA-AAAGGTCTTATAAAAATGATCACATACACATGAGAGATTAA 174
bat --TGATT-ATGTGCAGTA-AAAG-TCTTATAAAA-TGATCACATACACATGAGAGAT-AA 66
bushbaby TTTGATTTATGTGCAGTA-AAAGGTCTTATAAAAATGATCACATACACATGAGAGATTAA 107
armadillo T-TGATTTATGTGCAGTA-AAAGGTCTTATAAAAATGATCACATACACATGAGAGATTAA 174
cat T-TGATTTATGTGCAGTA-AAAGGTCTTATAAAAATGATCACATACACATGAGAGATTAA 174
dog T-TGATTTATGTGCAGTA-AAAGGTCTTATAAAAATGATCACATACACATGAGAGATTAA 174
macaque T-TGATTTATGTGCAGTA-AAAGGTCTTATAAAAATGATCACATACACATGAGAGATTAA 174
horse T-TGATTTATGTGCAGTA-AAAGGTCTTATAAAAATGATCACATACACATGAGAGATTAA 174
cow T-TGATTTATGTGCAGTA-AAAGGTCTTATAAAAATGATCACATACACATGAGAGATTAA 174
squirrel T-TGATTTATGTGCAGTA-AAAGGTCTTATAAAAATGATCACATACACATGAGAGATTAA 174
rabbit T-TGATTTATGTGCAGTA-AAAGGTCTTATAAAAATGATCACATACACATGAGAGATTAA 174
chicken T-TGATTTATGTGCAGTA-AAAGGTCTTATAAAAATGATCACATACACATGAGAGATTAA 174
rat T-TGATTTATGTGCAGTA-AAAGGTCTTATAAAAATGATCACATACACATGAGAGATTAA 174
mouse T-TGATTTATGTGCAGTA-AAAGGTCTTATAAAAATGATCACATACACATGAGAGATTAA 174
opossum T-TGATTTATGTGCAGTA-AAAGGTCTTATAAAAATGATCACATACACATGAGAGATTAA 174
frog T-TGATTTATGTGCAGTA-AAAGGTCTTATAAAAATGATCACATACACATGAGAGATTAA 174
shark T-TGATTTATGTGCTGTA-AAAGGTCTTATAAAAATGATCACATACACATGAGAGATTAA 172
zfish T-TGATTTATGTTTGGGAGAAAGGCCTTATAAAAATGATCACATACACGAAGGGGATTCA 75
 ***** **** * * **** ********* ************* * *** *
fugu TGCGCAGAAGATCAATAGAAATTTCATT-CTTGACACT--AGACTGTCACCTATGGTGAA 235
tetraodon TGCGCAGAAGATCAATAGAAATTTCATT-CTTGACACT--AGACTGTCACCTATGGTGAA 228
stickleback TGCGCAGAAGATCAATAGAAATTTCATT-CTTGACACT--AGACTGTCACCTATGGTGAA 235
medaka TGCGCAGAAGATCAATAGAAATTTCATT-CTTGACACT--AGACTGTCACCTATGGTGAA 235
human TGCGCAGGAGATCAATAGAAATTTCATT-CTTGACGCT--AGACTGTCACCTATGGTGAA 231
chimp TGCGCAGGAGATCAATAGAAATTTCATT-CTTGACGCT--AGACTGTCACCTATGGTGAA 231
orangutan TGCGCAGGAGATCAATAGAAATTTCATT-CTTGACGCT--AGACTGTCACCTATGGTGAA 231
bat TGCGCAGGAGATCAATAGAAATTTCATT-CTTGACGCT--AGACTGTCACCTATGGTGAA 123
bushbaby TGCGCAGGAGATCAATAGAAATTTCATT-CTTGACGCT--AGACTGTCACCTATGGTGAA 164
armadillo TGCGCAGGAGATCAATAGAAATTTCATT-CTTGACGCT--AGACTGTCACCTATGGTGAA 231
cat TGCGCAGGAGATCAATAGAAATTTCATT-CTTGACGCT--AGACTGTCACCTATGGTGAA 231
dog TGCGCAGGAGATCAATAGAAATTTCATT-CTTGACGCT--AGACTGTCACCTATGGTGAA 231
macaque TGCGCAGGAGATCAATAGAAATTTCATT-CTTGACGCT--AGACTGTCACCTATGGTGAA 231
horse TGCGCAGGAGATCAATAGAAATTTCATT-CTTGACGCT--AGACTGTCACCTATGGTGAA 231
cow TGCGCAGGAGATCAATAGAAATTTCATT-CTTGACGCT--AGACTGTCACCTATGGTGAA 231
squirrel TGCGCAGGAGATCAATAGAAATTTCATT-CTTGACGCT--AGACTGTCACCTATGGTGAA 231
rabbit TGCGCAGGAGATCAATAGAAATTTCATT-CTTGACGCT--AGACTGTCACCTATGGTGAA 231
chicken TGCGCTGGAGATCAATAGAAATTTCATT-CTTGACGCT--AGACTGTCACCTATGGTGAA 231
rat TGCGCAGGAGATCAATAGAAATTTCATT-CTTGACGCT--AGACTGTCACCTATGGTGAA 231
mouse TGCGCAGGAGATCAATAGAAATTTCATT-CTTGACGCT--AGACTGTCACCTATGGTGAA 231
opossum TGCGCAGGAGATCAATAGAAATTTCATT-CTTGACGCT--AGACTGTCACCTATGGTGAA 231
frog TGCGCAGGAGATCAATAGAAATTTCATT-CTTGACGCT--AGACTGTCACCTATGGTGAA 231
shark TGCACAGGAGATCAATAGAAATTTCATT-CTTGACGCT--AGACTGTCACCTATGGTGAA 229
zfish TGAGCCTCAGATCAATAGACATCCTGTTTCTCAACGCTTTGGACCATCACCGACTGTGAA 135
 ** * *********** ** ** ** ** ** *** ***** * *****

## Slide 41
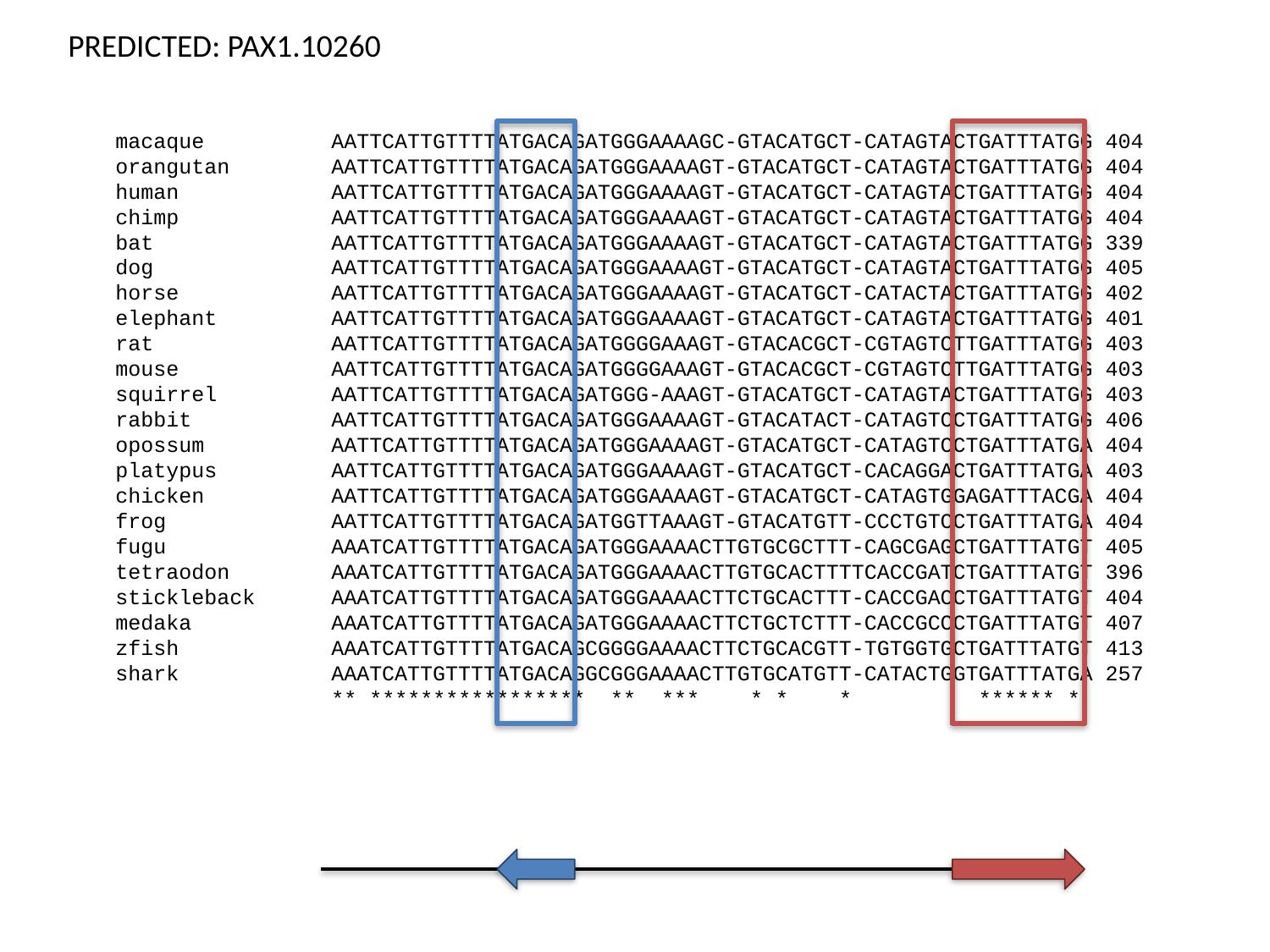

PREDICTED: PAX1.10260
macaque AATTCATTGTTTTATGACAGATGGGAAAAGC-GTACATGCT-CATAGTACTGATTTATGG 404
orangutan AATTCATTGTTTTATGACAGATGGGAAAAGT-GTACATGCT-CATAGTACTGATTTATGG 404
human AATTCATTGTTTTATGACAGATGGGAAAAGT-GTACATGCT-CATAGTACTGATTTATGG 404
chimp AATTCATTGTTTTATGACAGATGGGAAAAGT-GTACATGCT-CATAGTACTGATTTATGG 404
bat AATTCATTGTTTTATGACAGATGGGAAAAGT-GTACATGCT-CATAGTACTGATTTATGG 339
dog AATTCATTGTTTTATGACAGATGGGAAAAGT-GTACATGCT-CATAGTACTGATTTATGG 405
horse AATTCATTGTTTTATGACAGATGGGAAAAGT-GTACATGCT-CATACTACTGATTTATGG 402
elephant AATTCATTGTTTTATGACAGATGGGAAAAGT-GTACATGCT-CATAGTACTGATTTATGG 401
rat AATTCATTGTTTTATGACAGATGGGGAAAGT-GTACACGCT-CGTAGTCTTGATTTATGG 403
mouse AATTCATTGTTTTATGACAGATGGGGAAAGT-GTACACGCT-CGTAGTCTTGATTTATGG 403
squirrel AATTCATTGTTTTATGACAGATGGG-AAAGT-GTACATGCT-CATAGTACTGATTTATGG 403
rabbit AATTCATTGTTTTATGACAGATGGGAAAAGT-GTACATACT-CATAGTCCTGATTTATGG 406
opossum AATTCATTGTTTTATGACAGATGGGAAAAGT-GTACATGCT-CATAGTCCTGATTTATGA 404
platypus AATTCATTGTTTTATGACAGATGGGAAAAGT-GTACATGCT-CACAGGACTGATTTATGA 403
chicken AATTCATTGTTTTATGACAGATGGGAAAAGT-GTACATGCT-CATAGTGGAGATTTACGA 404
frog AATTCATTGTTTTATGACAGATGGTTAAAGT-GTACATGTT-CCCTGTCCTGATTTATGA 404
fugu AAATCATTGTTTTATGACAGATGGGAAAACTTGTGCGCTTT-CAGCGAGCTGATTTATGT 405
tetraodon AAATCATTGTTTTATGACAGATGGGAAAACTTGTGCACTTTTCACCGATCTGATTTATGT 396
stickleback AAATCATTGTTTTATGACAGATGGGAAAACTTCTGCACTTT-CACCGACCTGATTTATGT 404
medaka AAATCATTGTTTTATGACAGATGGGAAAACTTCTGCTCTTT-CACCGCCCTGATTTATGT 407
zfish AAATCATTGTTTTATGACAGCGGGGAAAACTTCTGCACGTT-TGTGGTGCTGATTTATGT 413
shark AAATCATTGTTTTATGACAGGCGGGAAAACTTGTGCATGTT-CATACTGGTGATTTATGA 257
 ** ***************** ** *** * * * ****** *

## Slide 42
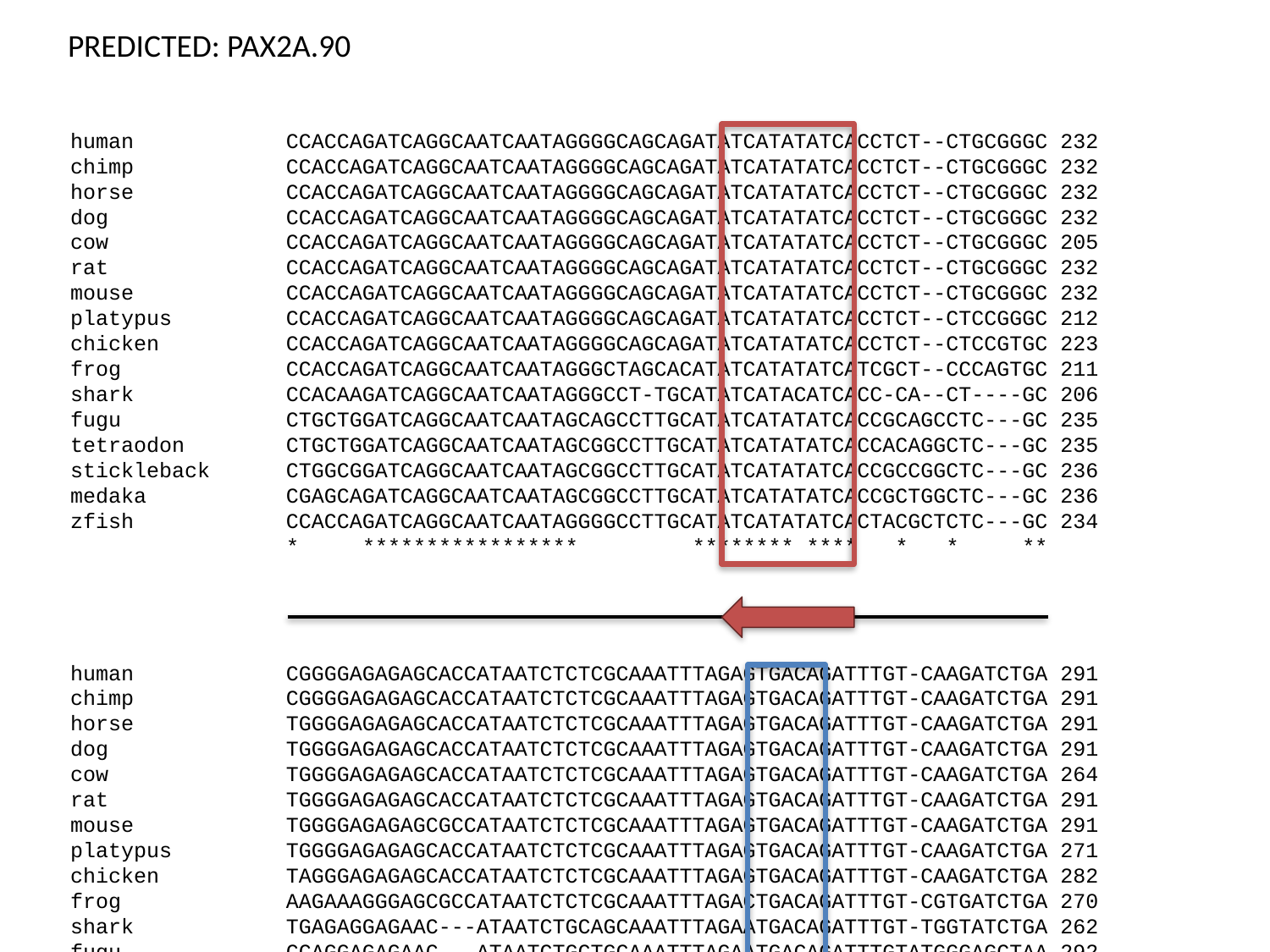

PREDICTED: PAX2A.90
human CCACCAGATCAGGCAATCAATAGGGGCAGCAGATATCATATATCACCTCT--CTGCGGGC 232
chimp CCACCAGATCAGGCAATCAATAGGGGCAGCAGATATCATATATCACCTCT--CTGCGGGC 232
horse CCACCAGATCAGGCAATCAATAGGGGCAGCAGATATCATATATCACCTCT--CTGCGGGC 232
dog CCACCAGATCAGGCAATCAATAGGGGCAGCAGATATCATATATCACCTCT--CTGCGGGC 232
cow CCACCAGATCAGGCAATCAATAGGGGCAGCAGATATCATATATCACCTCT--CTGCGGGC 205
rat CCACCAGATCAGGCAATCAATAGGGGCAGCAGATATCATATATCACCTCT--CTGCGGGC 232
mouse CCACCAGATCAGGCAATCAATAGGGGCAGCAGATATCATATATCACCTCT--CTGCGGGC 232
platypus CCACCAGATCAGGCAATCAATAGGGGCAGCAGATATCATATATCACCTCT--CTCCGGGC 212
chicken CCACCAGATCAGGCAATCAATAGGGGCAGCAGATATCATATATCACCTCT--CTCCGTGC 223
frog CCACCAGATCAGGCAATCAATAGGGCTAGCACATATCATATATCATCGCT--CCCAGTGC 211
shark CCACAAGATCAGGCAATCAATAGGGCCT-TGCATATCATACATCACC-CA--CT----GC 206
fugu CTGCTGGATCAGGCAATCAATAGCAGCCTTGCATATCATATATCACCGCAGCCTC---GC 235
tetraodon CTGCTGGATCAGGCAATCAATAGCGGCCTTGCATATCATATATCACCACAGGCTC---GC 235
stickleback CTGGCGGATCAGGCAATCAATAGCGGCCTTGCATATCATATATCACCGCCGGCTC---GC 236
medaka CGAGCAGATCAGGCAATCAATAGCGGCCTTGCATATCATATATCACCGCTGGCTC---GC 236
zfish CCACCAGATCAGGCAATCAATAGGGGCCTTGCATATCATATATCACTACGCTCTC---GC 234
 * ***************** ******** **** * * **
human CGGGGAGAGAGCACCATAATCTCTCGCAAATTTAGAGTGACAGATTTGT-CAAGATCTGA 291
chimp CGGGGAGAGAGCACCATAATCTCTCGCAAATTTAGAGTGACAGATTTGT-CAAGATCTGA 291
horse TGGGGAGAGAGCACCATAATCTCTCGCAAATTTAGAGTGACAGATTTGT-CAAGATCTGA 291
dog TGGGGAGAGAGCACCATAATCTCTCGCAAATTTAGAGTGACAGATTTGT-CAAGATCTGA 291
cow TGGGGAGAGAGCACCATAATCTCTCGCAAATTTAGAGTGACAGATTTGT-CAAGATCTGA 264
rat TGGGGAGAGAGCACCATAATCTCTCGCAAATTTAGAGTGACAGATTTGT-CAAGATCTGA 291
mouse TGGGGAGAGAGCGCCATAATCTCTCGCAAATTTAGAGTGACAGATTTGT-CAAGATCTGA 291
platypus TGGGGAGAGAGCACCATAATCTCTCGCAAATTTAGAGTGACAGATTTGT-CAAGATCTGA 271
chicken TAGGGAGAGAGCACCATAATCTCTCGCAAATTTAGAGTGACAGATTTGT-CAAGATCTGA 282
frog AAGAAAGGGAGCGCCATAATCTCTCGCAAATTTAGACTGACAGATTTGT-CGTGATCTGA 270
shark TGAGAGGAGAAC---ATAATCTGCAGCAAATTTAGAATGACAGATTTGT-TGGTATCTGA 262
fugu CCAGGAGAGAAC---ATAATCTGCTGCAAATTTAGAATGACAGATTTGTATGGGAGCTAA 292
tetraodon CCAGGAGAGAAC---ATAATCTGCTGCAAATTTAGAATGACAGATTTGTATGAGCGCTAA 292
stickleback CCAGGAGAGAAC---ATAATCTGCTGCAAATTTAGAATGACAGATTTGTATGGGAGCTAA 293
medaka CCAGGAGAGAAC---ATAATCTGCTGCAAATTTAGAATGACAGATTTGTACGGGAGCTAA 293
zfish CGAGGAGAGAAC---ATAATCTGCTGCAAATTTAGAATGACAGATTTGTAAGGGAGC--- 288
 * ** * ******* *********** ************ *

## Slide 43
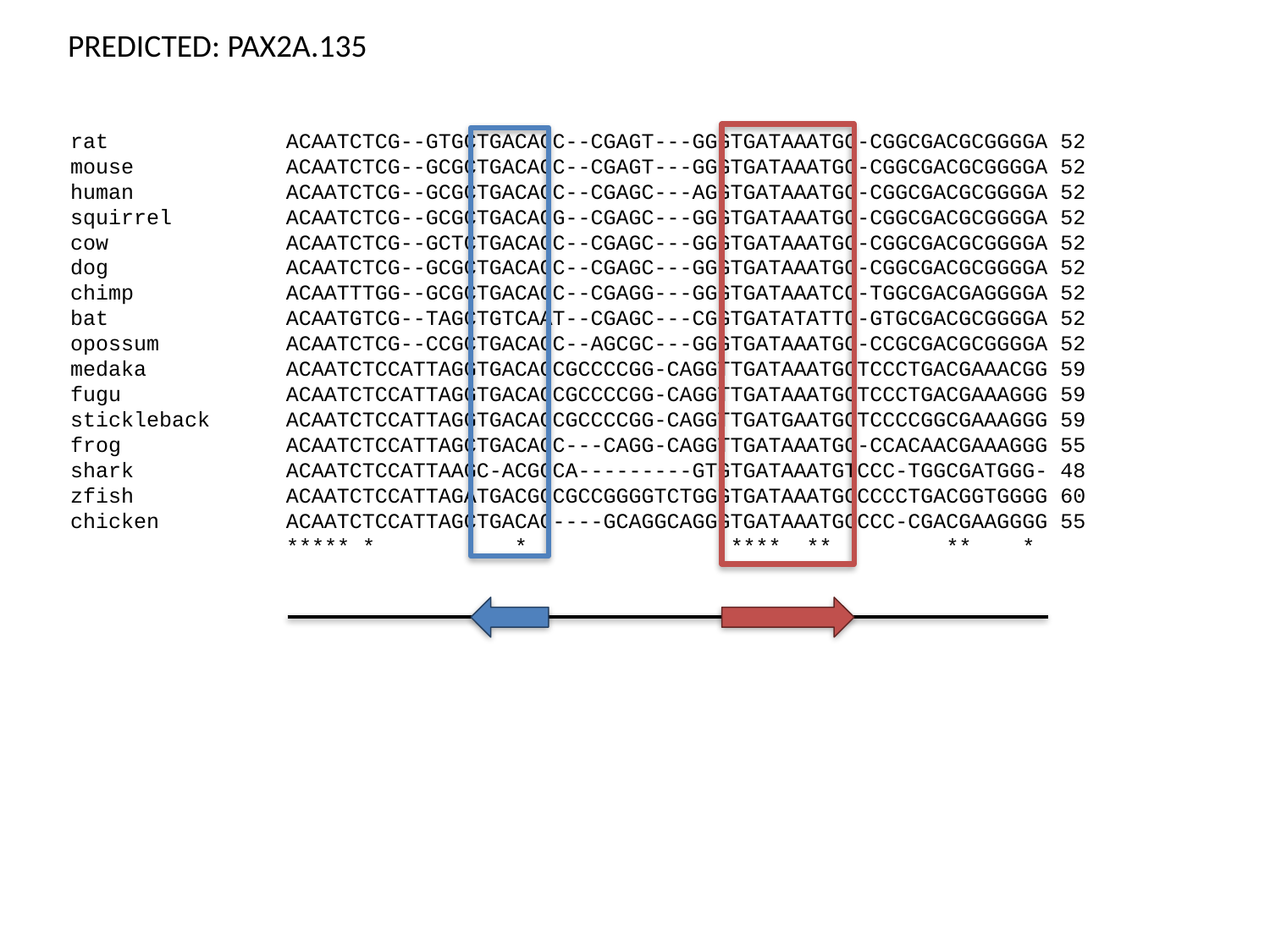

PREDICTED: PAX2A.135
rat ACAATCTCG--GTGCTGACAGC--CGAGT---GGGTGATAAATGC-CGGCGACGCGGGGA 52
mouse ACAATCTCG--GCGCTGACAGC--CGAGT---GGGTGATAAATGC-CGGCGACGCGGGGA 52
human ACAATCTCG--GCGCTGACAGC--CGAGC---AGGTGATAAATGC-CGGCGACGCGGGGA 52
squirrel ACAATCTCG--GCGCTGACAGG--CGAGC---GGGTGATAAATGC-CGGCGACGCGGGGA 52
cow ACAATCTCG--GCTCTGACAGC--CGAGC---GGGTGATAAATGC-CGGCGACGCGGGGA 52
dog ACAATCTCG--GCGCTGACAGC--CGAGC---GGGTGATAAATGC-CGGCGACGCGGGGA 52
chimp ACAATTTGG--GCGCTGACAGC--CGAGG---GGGTGATAAATCC-TGGCGACGAGGGGA 52
bat ACAATGTCG--TAGCTGTCAAT--CGAGC---CGGTGATATATTC-GTGCGACGCGGGGA 52
opossum ACAATCTCG--CCGCTGACAGC--AGCGC---GGGTGATAAATGC-CCGCGACGCGGGGA 52
medaka ACAATCTCCATTAGGTGACAGCGCCCCGG-CAGGTTGATAAATGCTCCCTGACGAAACGG 59
fugu ACAATCTCCATTAGGTGACAGCGCCCCGG-CAGGTTGATAAATGCTCCCTGACGAAAGGG 59
stickleback ACAATCTCCATTAGGTGACAGCGCCCCGG-CAGGTTGATGAATGCTCCCCGGCGAAAGGG 59
frog ACAATCTCCATTAGCTGACAGC---CAGG-CAGGTTGATAAATGC-CCACAACGAAAGGG 55
shark ACAATCTCCATTAAGC-ACGGCA---------GTGTGATAAATGTCCC-TGGCGATGGG- 48
zfish ACAATCTCCATTAGATGACGGCGCCGGGGTCTGGGTGATAAATGCCCCCTGACGGTGGGG 60
chicken ACAATCTCCATTAGCTGACAG----GCAGGCAGGGTGATAAATGCCCC-CGACGAAGGGG 55
 ***** * * **** ** ** *

## Slide 44
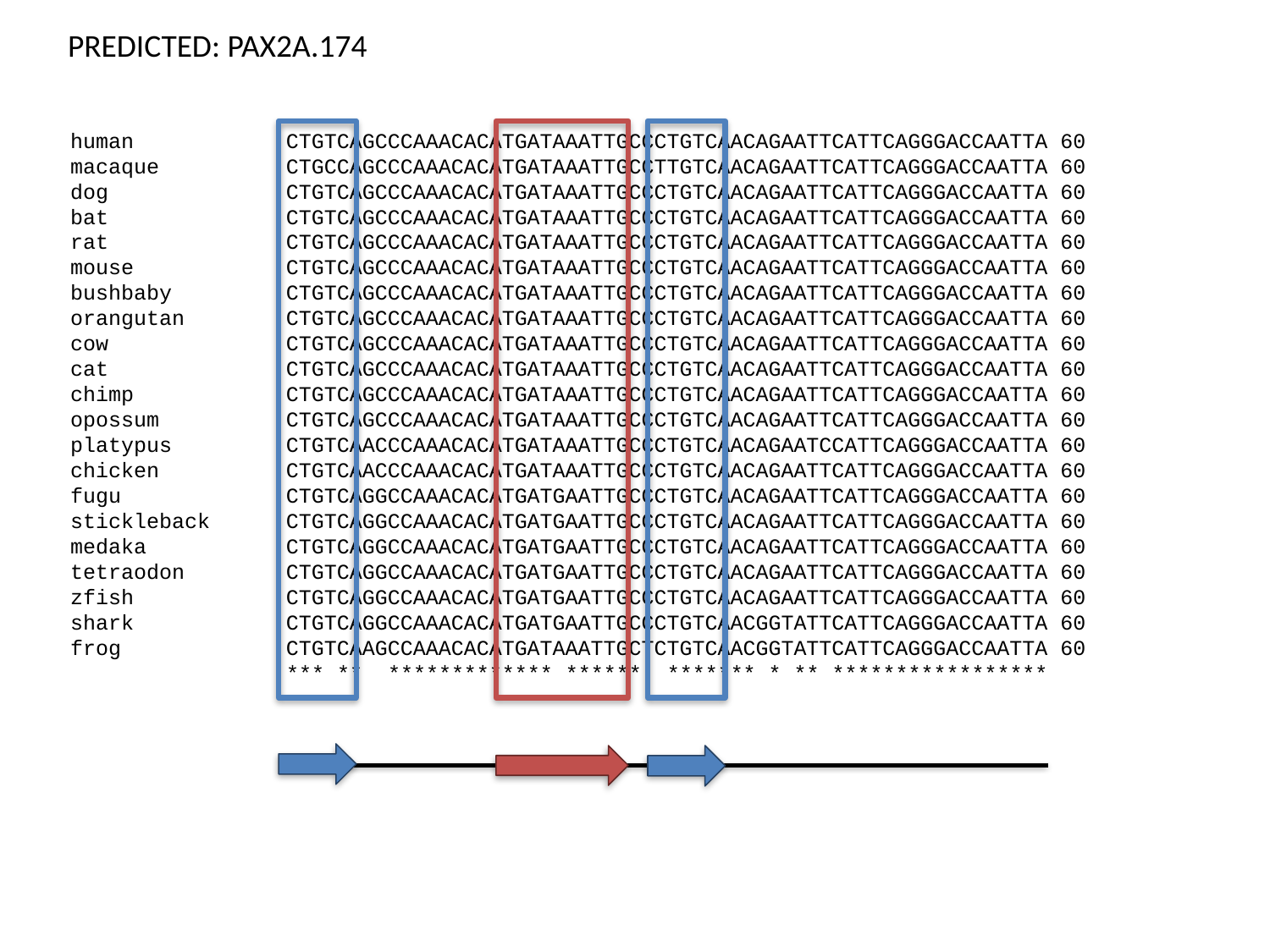

PREDICTED: PAX2A.174
human CTGTCAGCCCAAACACATGATAAATTGCCCTGTCAACAGAATTCATTCAGGGACCAATTA 60
macaque CTGCCAGCCCAAACACATGATAAATTGCCTTGTCAACAGAATTCATTCAGGGACCAATTA 60
dog CTGTCAGCCCAAACACATGATAAATTGCCCTGTCAACAGAATTCATTCAGGGACCAATTA 60
bat CTGTCAGCCCAAACACATGATAAATTGCCCTGTCAACAGAATTCATTCAGGGACCAATTA 60
rat CTGTCAGCCCAAACACATGATAAATTGCCCTGTCAACAGAATTCATTCAGGGACCAATTA 60
mouse CTGTCAGCCCAAACACATGATAAATTGCCCTGTCAACAGAATTCATTCAGGGACCAATTA 60
bushbaby CTGTCAGCCCAAACACATGATAAATTGCCCTGTCAACAGAATTCATTCAGGGACCAATTA 60
orangutan CTGTCAGCCCAAACACATGATAAATTGCCCTGTCAACAGAATTCATTCAGGGACCAATTA 60
cow CTGTCAGCCCAAACACATGATAAATTGCCCTGTCAACAGAATTCATTCAGGGACCAATTA 60
cat CTGTCAGCCCAAACACATGATAAATTGCCCTGTCAACAGAATTCATTCAGGGACCAATTA 60
chimp CTGTCAGCCCAAACACATGATAAATTGCCCTGTCAACAGAATTCATTCAGGGACCAATTA 60
opossum CTGTCAGCCCAAACACATGATAAATTGCCCTGTCAACAGAATTCATTCAGGGACCAATTA 60
platypus CTGTCAACCCAAACACATGATAAATTGCCCTGTCAACAGAATCCATTCAGGGACCAATTA 60
chicken CTGTCAACCCAAACACATGATAAATTGCCCTGTCAACAGAATTCATTCAGGGACCAATTA 60
fugu CTGTCAGGCCAAACACATGATGAATTGCCCTGTCAACAGAATTCATTCAGGGACCAATTA 60
stickleback CTGTCAGGCCAAACACATGATGAATTGCCCTGTCAACAGAATTCATTCAGGGACCAATTA 60
medaka CTGTCAGGCCAAACACATGATGAATTGCCCTGTCAACAGAATTCATTCAGGGACCAATTA 60
tetraodon CTGTCAGGCCAAACACATGATGAATTGCCCTGTCAACAGAATTCATTCAGGGACCAATTA 60
zfish CTGTCAGGCCAAACACATGATGAATTGCCCTGTCAACAGAATTCATTCAGGGACCAATTA 60
shark CTGTCAGGCCAAACACATGATGAATTGCCCTGTCAACGGTATTCATTCAGGGACCAATTA 60
frog CTGTCAAGCCAAACACATGATAAATTGCTCTGTCAACGGTATTCATTCAGGGACCAATTA 60
 *** ** ************* ****** ******* * ** *****************

## Slide 45
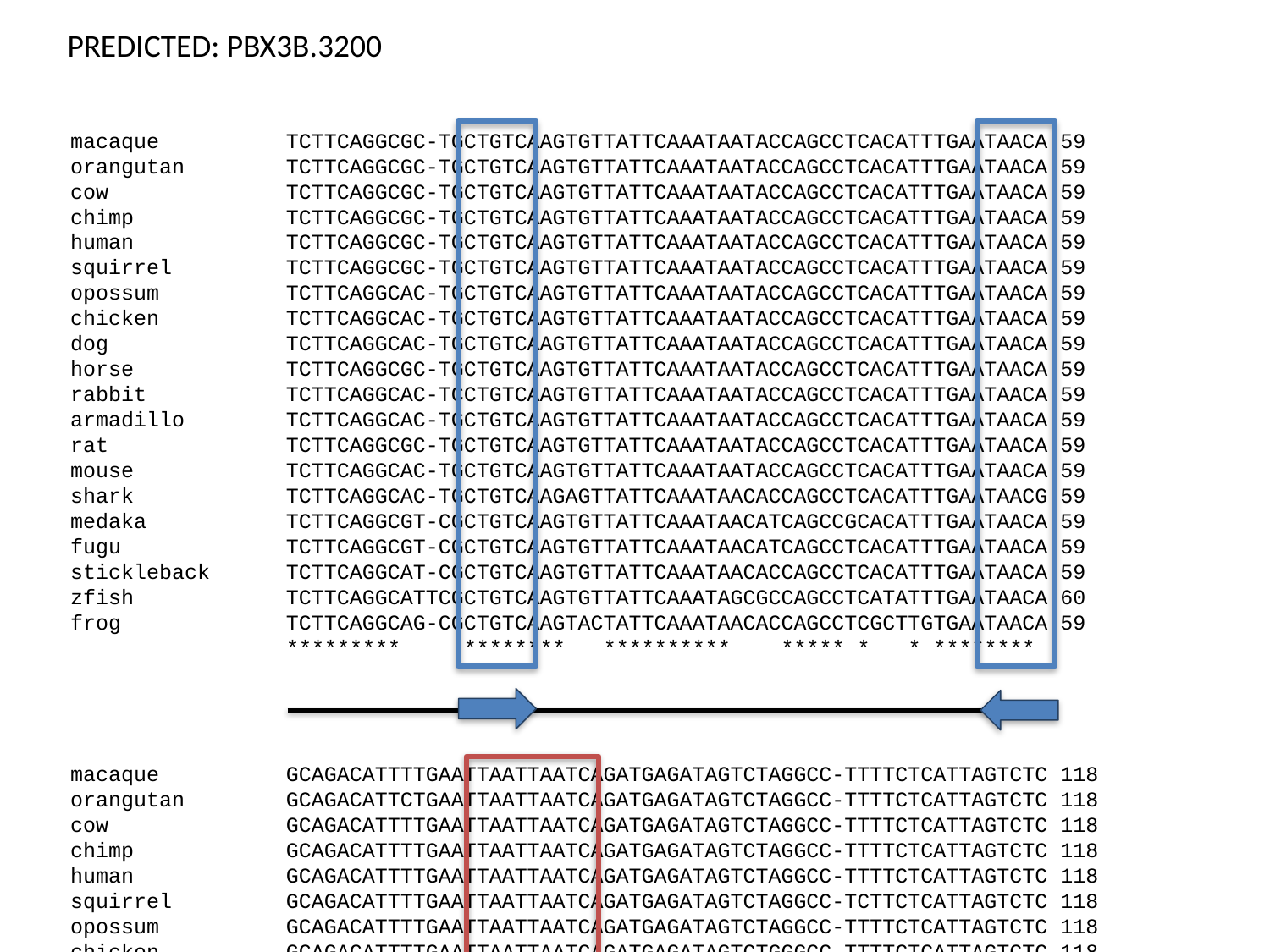

PREDICTED: PBX3B.3200
macaque TCTTCAGGCGC-TGCTGTCAAGTGTTATTCAAATAATACCAGCCTCACATTTGAATAACA 59
orangutan TCTTCAGGCGC-TGCTGTCAAGTGTTATTCAAATAATACCAGCCTCACATTTGAATAACA 59
cow TCTTCAGGCGC-TGCTGTCAAGTGTTATTCAAATAATACCAGCCTCACATTTGAATAACA 59
chimp TCTTCAGGCGC-TGCTGTCAAGTGTTATTCAAATAATACCAGCCTCACATTTGAATAACA 59
human TCTTCAGGCGC-TGCTGTCAAGTGTTATTCAAATAATACCAGCCTCACATTTGAATAACA 59
squirrel TCTTCAGGCGC-TGCTGTCAAGTGTTATTCAAATAATACCAGCCTCACATTTGAATAACA 59
opossum TCTTCAGGCAC-TGCTGTCAAGTGTTATTCAAATAATACCAGCCTCACATTTGAATAACA 59
chicken TCTTCAGGCAC-TGCTGTCAAGTGTTATTCAAATAATACCAGCCTCACATTTGAATAACA 59
dog TCTTCAGGCAC-TGCTGTCAAGTGTTATTCAAATAATACCAGCCTCACATTTGAATAACA 59
horse TCTTCAGGCGC-TGCTGTCAAGTGTTATTCAAATAATACCAGCCTCACATTTGAATAACA 59
rabbit TCTTCAGGCAC-TCCTGTCAAGTGTTATTCAAATAATACCAGCCTCACATTTGAATAACA 59
armadillo TCTTCAGGCAC-TGCTGTCAAGTGTTATTCAAATAATACCAGCCTCACATTTGAATAACA 59
rat TCTTCAGGCGC-TGCTGTCAAGTGTTATTCAAATAATACCAGCCTCACATTTGAATAACA 59
mouse TCTTCAGGCAC-TGCTGTCAAGTGTTATTCAAATAATACCAGCCTCACATTTGAATAACA 59
shark TCTTCAGGCAC-TGCTGTCAAGAGTTATTCAAATAACACCAGCCTCACATTTGAATAACG 59
medaka TCTTCAGGCGT-CGCTGTCAAGTGTTATTCAAATAACATCAGCCGCACATTTGAATAACA 59
fugu TCTTCAGGCGT-CGCTGTCAAGTGTTATTCAAATAACATCAGCCTCACATTTGAATAACA 59
stickleback TCTTCAGGCAT-CGCTGTCAAGTGTTATTCAAATAACACCAGCCTCACATTTGAATAACA 59
zfish TCTTCAGGCATTCGCTGTCAAGTGTTATTCAAATAGCGCCAGCCTCATATTTGAATAACA 60
frog TCTTCAGGCAG-CGCTGTCAAGTACTATTCAAATAACACCAGCCTCGCTTGTGAATAACA 59
 ********* ******** ********** ***** * * ********
macaque GCAGACATTTTGAATTAATTAATCAGATGAGATAGTCTAGGCC-TTTTCTCATTAGTCTC 118
orangutan GCAGACATTCTGAATTAATTAATCAGATGAGATAGTCTAGGCC-TTTTCTCATTAGTCTC 118
cow GCAGACATTTTGAATTAATTAATCAGATGAGATAGTCTAGGCC-TTTTCTCATTAGTCTC 118
chimp GCAGACATTTTGAATTAATTAATCAGATGAGATAGTCTAGGCC-TTTTCTCATTAGTCTC 118
human GCAGACATTTTGAATTAATTAATCAGATGAGATAGTCTAGGCC-TTTTCTCATTAGTCTC 118
squirrel GCAGACATTTTGAATTAATTAATCAGATGAGATAGTCTAGGCC-TCTTCTCATTAGTCTC 118
opossum GCAGACATTTTGAATTAATTAATCAGATGAGATAGTCTAGGCC-TTTTCTCATTAGTCTC 118
chicken GCAGACATTTTGAATTAATTAATCAGATGAGATAGTCTGGGCC-TTTTCTCATTAGTCTC 118
dog GCAGACATTTTGAATTAATTAATCAGATGAGATAGTTTAGGCC-TTTTCTCATTAGTCTC 118
horse GCAGACATTTTGAATTAATTAATCAGATGAGATAGTCTAGGCC-TTTTCTCATTAGTCTC 118
rabbit GCAGACATTTTGAATTAATTAATCAGATGAGATAGTCTAGGCC-TTTTCTCATTAGTCTC 118
armadillo GCAGACATTTTGAATTAATTAATCAGATGAGATAGTCTAGGCC-TTTTCTCATTAGTCTC 118
rat GCAGACATTTTGAATTAATTAATCAGATGAGATAGTCTAGGCC-TTTTCTCATTAGTCTC 118
mouse GCAGACATTTTGAATTAATTAATCAGATGAGATAGTCTAGGCC-TTTTCTCATTAGTCTC 118
shark GCAGACATTTTGAATTAATTAATCAGAGGAGATAGTCCAGGGC-TTTTCTCATTAGTCTT 118
medaka GCCGACATTTTGAATTAATTAATCAGACAAGATAGTC-TGGGCCTTTTCTCATTAGTCTA 118
fugu GCCGGCATTTTGAATTAATTAATCAGACAAGATAGTC-GGGCCTTTTTGTCATTAGTCTA 118
stickleback GCCGACATTTTGAATTAATTAATCAGACAAGATAGTC-TGGGCCTTTTCTCATTAGTCTA 118
zfish GCCGACATTTTGAATTAATTAATCAGCCGAGATCGTCCAGGGCCTTTTCTCATTAGCGGA 120
frog GCAGACATTTTGAATTAATTAATCAGCTGAGATAGCTCGATCCCTCTTCTCATTAGTCTG 119
 ** * **** **************** **** * * * ** *******

## Slide 46
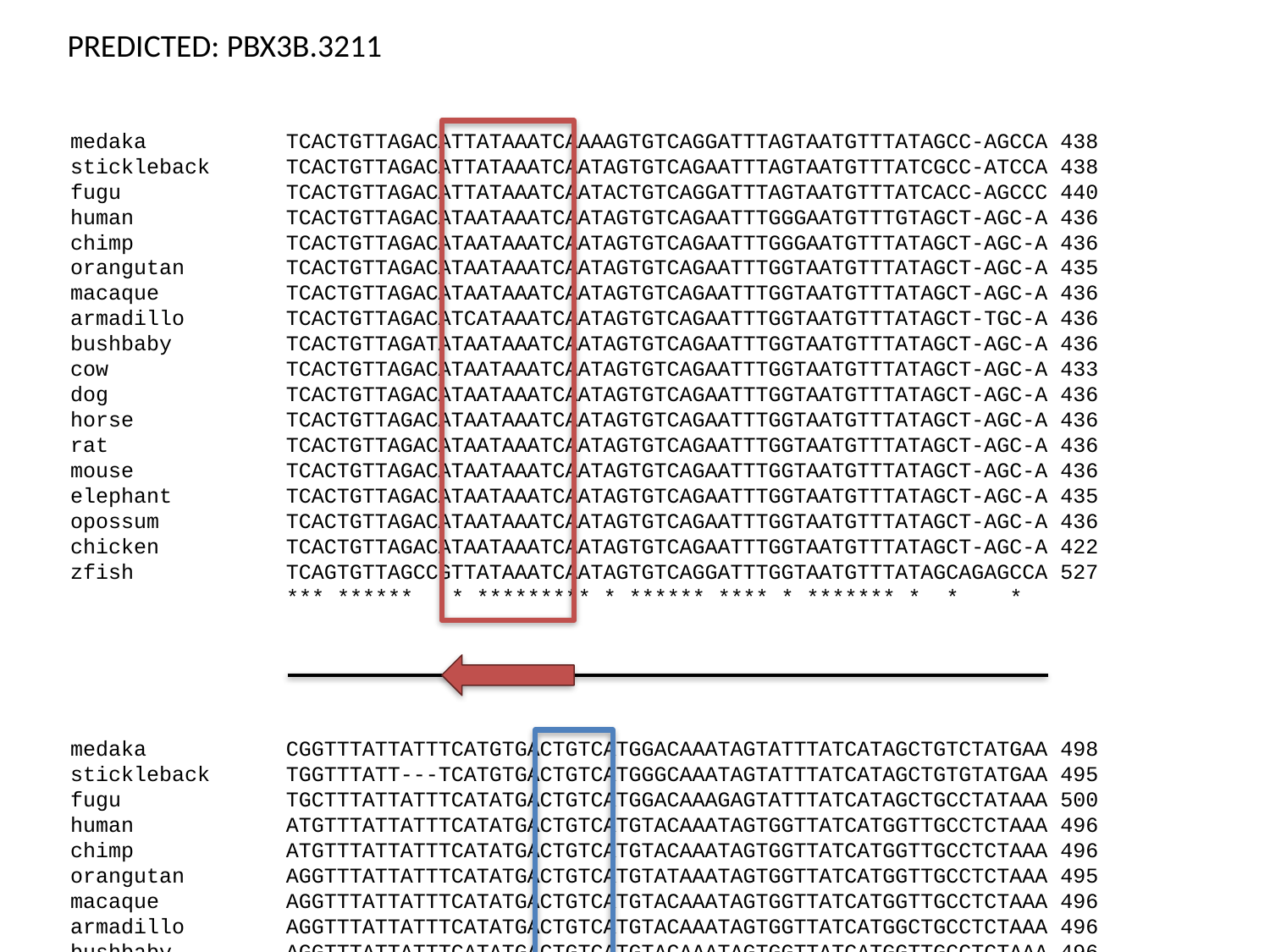

PREDICTED: PBX3B.3211
medaka TCACTGTTAGACATTATAAATCAAAAGTGTCAGGATTTAGTAATGTTTATAGCC-AGCCA 438
stickleback TCACTGTTAGACATTATAAATCAATAGTGTCAGAATTTAGTAATGTTTATCGCC-ATCCA 438
fugu TCACTGTTAGACATTATAAATCAATACTGTCAGGATTTAGTAATGTTTATCACC-AGCCC 440
human TCACTGTTAGACATAATAAATCAATAGTGTCAGAATTTGGGAATGTTTGTAGCT-AGC-A 436
chimp TCACTGTTAGACATAATAAATCAATAGTGTCAGAATTTGGGAATGTTTATAGCT-AGC-A 436
orangutan TCACTGTTAGACATAATAAATCAATAGTGTCAGAATTTGGTAATGTTTATAGCT-AGC-A 435
macaque TCACTGTTAGACATAATAAATCAATAGTGTCAGAATTTGGTAATGTTTATAGCT-AGC-A 436
armadillo TCACTGTTAGACATCATAAATCAATAGTGTCAGAATTTGGTAATGTTTATAGCT-TGC-A 436
bushbaby TCACTGTTAGATATAATAAATCAATAGTGTCAGAATTTGGTAATGTTTATAGCT-AGC-A 436
cow TCACTGTTAGACATAATAAATCAATAGTGTCAGAATTTGGTAATGTTTATAGCT-AGC-A 433
dog TCACTGTTAGACATAATAAATCAATAGTGTCAGAATTTGGTAATGTTTATAGCT-AGC-A 436
horse TCACTGTTAGACATAATAAATCAATAGTGTCAGAATTTGGTAATGTTTATAGCT-AGC-A 436
rat TCACTGTTAGACATAATAAATCAATAGTGTCAGAATTTGGTAATGTTTATAGCT-AGC-A 436
mouse TCACTGTTAGACATAATAAATCAATAGTGTCAGAATTTGGTAATGTTTATAGCT-AGC-A 436
elephant TCACTGTTAGACATAATAAATCAATAGTGTCAGAATTTGGTAATGTTTATAGCT-AGC-A 435
opossum TCACTGTTAGACATAATAAATCAATAGTGTCAGAATTTGGTAATGTTTATAGCT-AGC-A 436
chicken TCACTGTTAGACATAATAAATCAATAGTGTCAGAATTTGGTAATGTTTATAGCT-AGC-A 422
zfish TCAGTGTTAGCCGTTATAAATCAATAGTGTCAGGATTTGGTAATGTTTATAGCAGAGCCA 527
 *** ****** * ********* * ****** **** * ******* * * *
medaka CGGTTTATTATTTCATGTGACTGTCATGGACAAATAGTATTTATCATAGCTGTCTATGAA 498
stickleback TGGTTTATT---TCATGTGACTGTCATGGGCAAATAGTATTTATCATAGCTGTGTATGAA 495
fugu TGCTTTATTATTTCATATGACTGTCATGGACAAAGAGTATTTATCATAGCTGCCTATAAA 500
human ATGTTTATTATTTCATATGACTGTCATGTACAAATAGTGGTTATCATGGTTGCCTCTAAA 496
chimp ATGTTTATTATTTCATATGACTGTCATGTACAAATAGTGGTTATCATGGTTGCCTCTAAA 496
orangutan AGGTTTATTATTTCATATGACTGTCATGTATAAATAGTGGTTATCATGGTTGCCTCTAAA 495
macaque AGGTTTATTATTTCATATGACTGTCATGTACAAATAGTGGTTATCATGGTTGCCTCTAAA 496
armadillo AGGTTTATTATTTCATATGACTGTCATGTACAAATAGTGGTTATCATGGCTGCCTCTAAA 496
bushbaby AGGTTTATTATTTCATATGACTGTCATGTACAAATAGTGGTTATCATGGTTGCCTCTAAA 496
cow CGGTTTATTATTTCATATGACTGTCATGTACAAATAGTGGTTATCATGGCTGCCTCTAAA 493
dog AGGTTTATTATTTCATATGACTGTCATGTACAAATAGTGGTTATCATGGCTGCCTCTAAA 496
horse AGGTTTATTATTTCATATGACTGTCATGTACAAATAGTGGTTATCATGGCTGCCTCTAAA 496
rat AGGTTTATTATTTCATATGACTGTCATGTACAAATGGTGGTTATCATGGCTGCCTCTAAA 496
mouse AGGTTTATTATTTCATATGACTGTCATGTACAAATGGTGGTTATCATGGCTGCCTCTAAA 496
elephant AGGTTTATTATTTCATATGACTGTCATGTACAAATAGTGGTTATCATGGCTACCTCTAAA 495
opossum AGGTTTATTATTTCATATGACTGTCATGTACAAATAGTGGTTATCATGGCTGCTTCTAAA 496
chicken AGGTTTATTATTTCATATGACTGTCATGTACAAATAGTGGTTATCATGGCTGCCTCTAAA 482
zfish AGGTTTATTATTTCATGTGACTGTCATGGACAAATAGTATTTATCATAGCTGTCTATAAA 587
 ****** **** *********** *** ** ******* * * * * **

## Slide 47
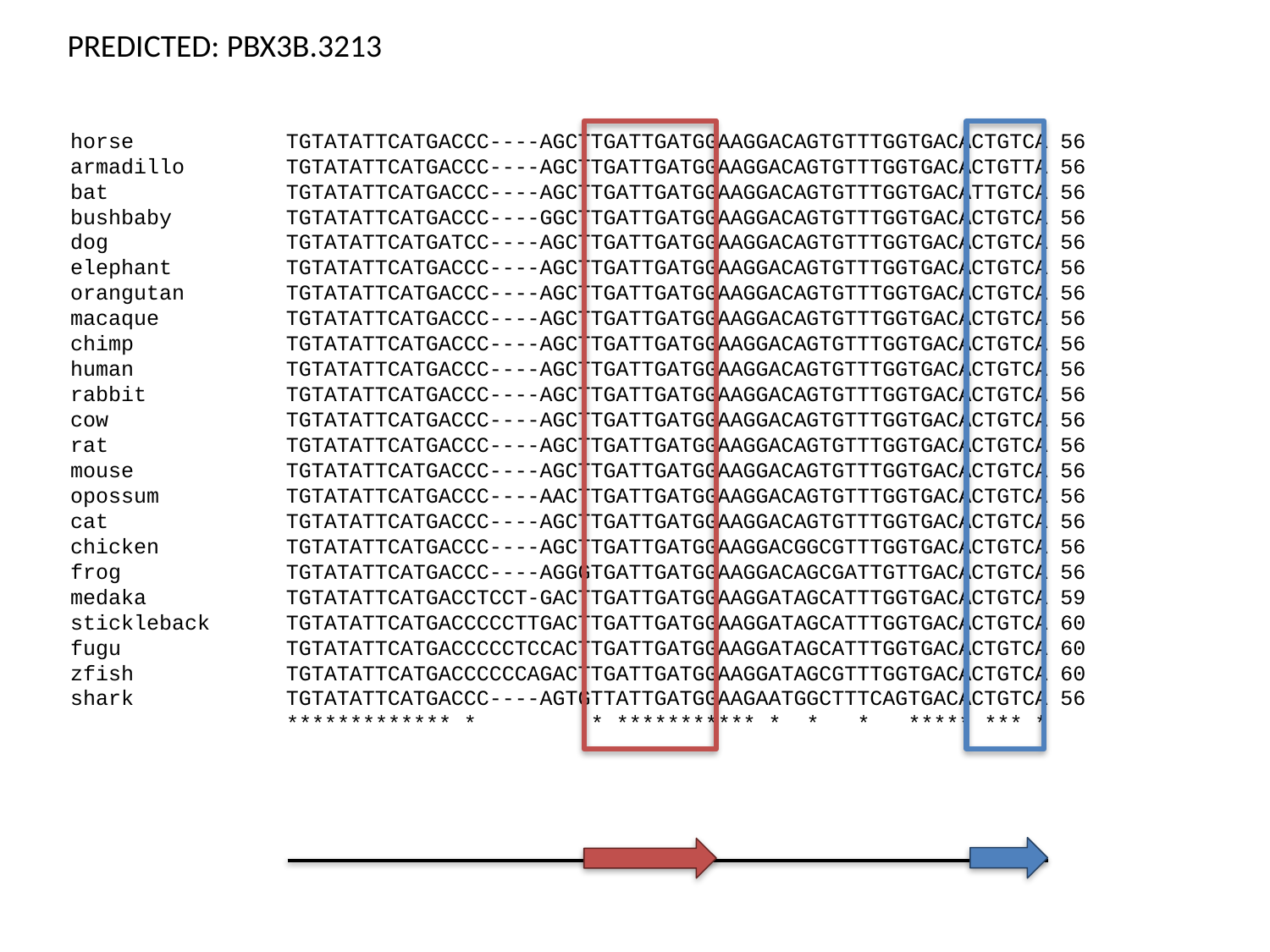

PREDICTED: PBX3B.3213
horse TGTATATTCATGACCC----AGCTTGATTGATGGAAGGACAGTGTTTGGTGACACTGTCA 56
armadillo TGTATATTCATGACCC----AGCTTGATTGATGGAAGGACAGTGTTTGGTGACACTGTTA 56
bat TGTATATTCATGACCC----AGCTTGATTGATGGAAGGACAGTGTTTGGTGACATTGTCA 56
bushbaby TGTATATTCATGACCC----GGCTTGATTGATGGAAGGACAGTGTTTGGTGACACTGTCA 56
dog TGTATATTCATGATCC----AGCTTGATTGATGGAAGGACAGTGTTTGGTGACACTGTCA 56
elephant TGTATATTCATGACCC----AGCTTGATTGATGGAAGGACAGTGTTTGGTGACACTGTCA 56
orangutan TGTATATTCATGACCC----AGCTTGATTGATGGAAGGACAGTGTTTGGTGACACTGTCA 56
macaque TGTATATTCATGACCC----AGCTTGATTGATGGAAGGACAGTGTTTGGTGACACTGTCA 56
chimp TGTATATTCATGACCC----AGCTTGATTGATGGAAGGACAGTGTTTGGTGACACTGTCA 56
human TGTATATTCATGACCC----AGCTTGATTGATGGAAGGACAGTGTTTGGTGACACTGTCA 56
rabbit TGTATATTCATGACCC----AGCTTGATTGATGGAAGGACAGTGTTTGGTGACACTGTCA 56
cow TGTATATTCATGACCC----AGCTTGATTGATGGAAGGACAGTGTTTGGTGACACTGTCA 56
rat TGTATATTCATGACCC----AGCTTGATTGATGGAAGGACAGTGTTTGGTGACACTGTCA 56
mouse TGTATATTCATGACCC----AGCTTGATTGATGGAAGGACAGTGTTTGGTGACACTGTCA 56
opossum TGTATATTCATGACCC----AACTTGATTGATGGAAGGACAGTGTTTGGTGACACTGTCA 56
cat TGTATATTCATGACCC----AGCTTGATTGATGGAAGGACAGTGTTTGGTGACACTGTCA 56
chicken TGTATATTCATGACCC----AGCTTGATTGATGGAAGGACGGCGTTTGGTGACACTGTCA 56
frog TGTATATTCATGACCC----AGGGTGATTGATGGAAGGACAGCGATTGTTGACACTGTCA 56
medaka TGTATATTCATGACCTCCT-GACTTGATTGATGGAAGGATAGCATTTGGTGACACTGTCA 59
stickleback TGTATATTCATGACCCCCTTGACTTGATTGATGGAAGGATAGCATTTGGTGACACTGTCA 60
fugu TGTATATTCATGACCCCCTCCACTTGATTGATGGAAGGATAGCATTTGGTGACACTGTCA 60
zfish TGTATATTCATGACCCCCCAGACTTGATTGATGGAAGGATAGCGTTTGGTGACACTGTCA 60
shark TGTATATTCATGACCC----AGTGTTATTGATGGAAGAATGGCTTTCAGTGACACTGTCA 56
 ************* * * *********** * * * ***** *** *

## Slide 48
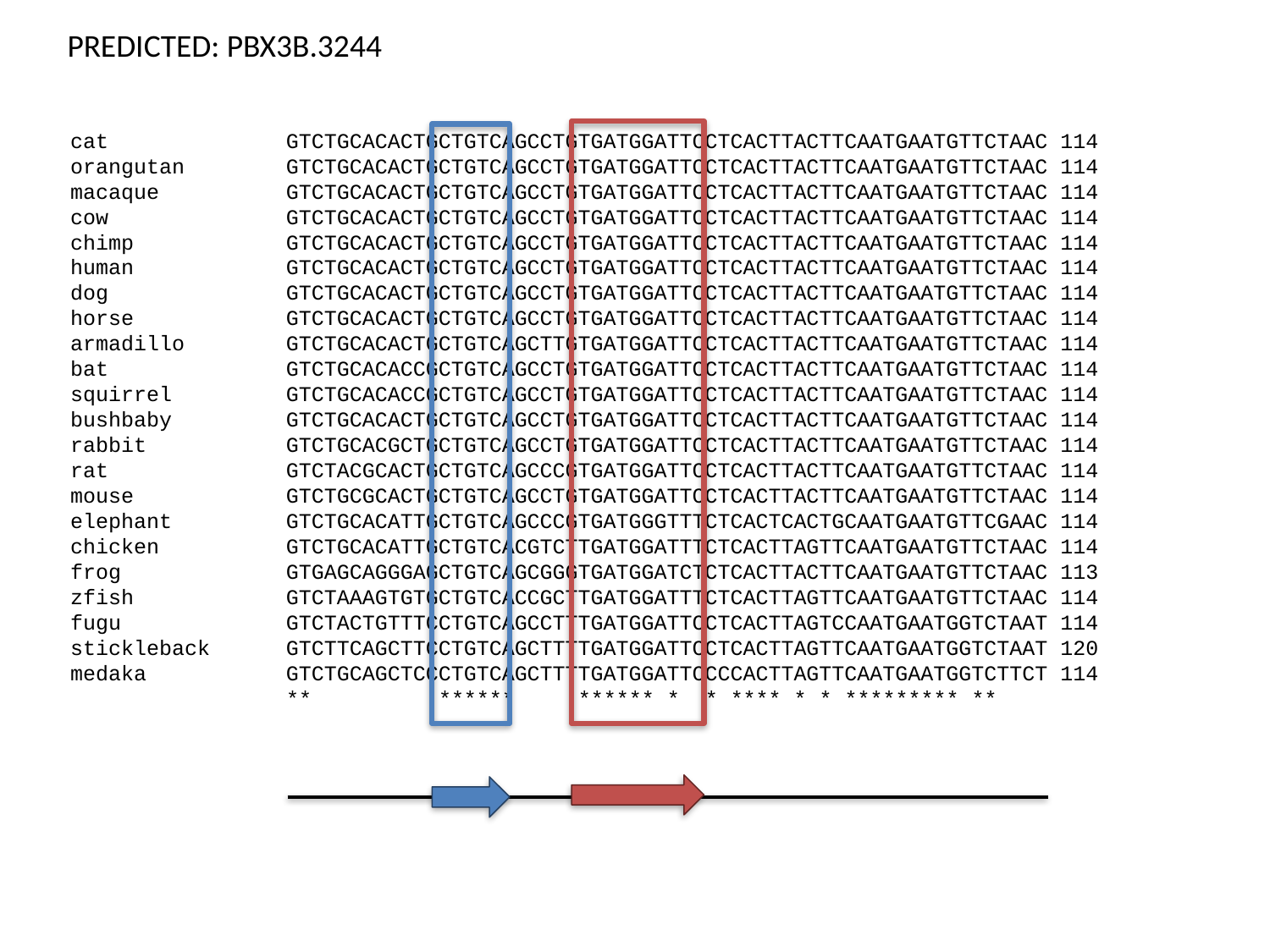

PREDICTED: PBX3B.3244
cat GTCTGCACACTGCTGTCAGCCTGTGATGGATTCCTCACTTACTTCAATGAATGTTCTAAC 114
orangutan GTCTGCACACTGCTGTCAGCCTGTGATGGATTCCTCACTTACTTCAATGAATGTTCTAAC 114
macaque GTCTGCACACTGCTGTCAGCCTGTGATGGATTCCTCACTTACTTCAATGAATGTTCTAAC 114
cow GTCTGCACACTGCTGTCAGCCTGTGATGGATTCCTCACTTACTTCAATGAATGTTCTAAC 114
chimp GTCTGCACACTGCTGTCAGCCTGTGATGGATTCCTCACTTACTTCAATGAATGTTCTAAC 114
human GTCTGCACACTGCTGTCAGCCTGTGATGGATTCCTCACTTACTTCAATGAATGTTCTAAC 114
dog GTCTGCACACTGCTGTCAGCCTGTGATGGATTCCTCACTTACTTCAATGAATGTTCTAAC 114
horse GTCTGCACACTGCTGTCAGCCTGTGATGGATTCCTCACTTACTTCAATGAATGTTCTAAC 114
armadillo GTCTGCACACTGCTGTCAGCTTGTGATGGATTCCTCACTTACTTCAATGAATGTTCTAAC 114
bat GTCTGCACACCGCTGTCAGCCTGTGATGGATTCCTCACTTACTTCAATGAATGTTCTAAC 114
squirrel GTCTGCACACCGCTGTCAGCCTGTGATGGATTCCTCACTTACTTCAATGAATGTTCTAAC 114
bushbaby GTCTGCACACTGCTGTCAGCCTGTGATGGATTCCTCACTTACTTCAATGAATGTTCTAAC 114
rabbit GTCTGCACGCTGCTGTCAGCCTGTGATGGATTCCTCACTTACTTCAATGAATGTTCTAAC 114
rat GTCTACGCACTGCTGTCAGCCCGTGATGGATTCCTCACTTACTTCAATGAATGTTCTAAC 114
mouse GTCTGCGCACTGCTGTCAGCCTGTGATGGATTCCTCACTTACTTCAATGAATGTTCTAAC 114
elephant GTCTGCACATTGCTGTCAGCCCGTGATGGGTTTCTCACTCACTGCAATGAATGTTCGAAC 114
chicken GTCTGCACATTGCTGTCACGTCTTGATGGATTTCTCACTTAGTTCAATGAATGTTCTAAC 114
frog GTGAGCAGGGAGCTGTCAGCGGGTGATGGATCTCTCACTTACTTCAATGAATGTTCTAAC 113
zfish GTCTAAAGTGTGCTGTCACCGCTTGATGGATTTCTCACTTAGTTCAATGAATGTTCTAAC 114
fugu GTCTACTGTTTCCTGTCAGCCTTTGATGGATTCCTCACTTAGTCCAATGAATGGTCTAAT 114
stickleback GTCTTCAGCTTCCTGTCAGCTTTTGATGGATTCCTCACTTAGTTCAATGAATGGTCTAAT 120
medaka GTCTGCAGCTCCCTGTCAGCTTTTGATGGATTCCCCACTTAGTTCAATGAATGGTCTTCT 114
 ** ****** ****** * * **** * * ********* **

## Slide 49
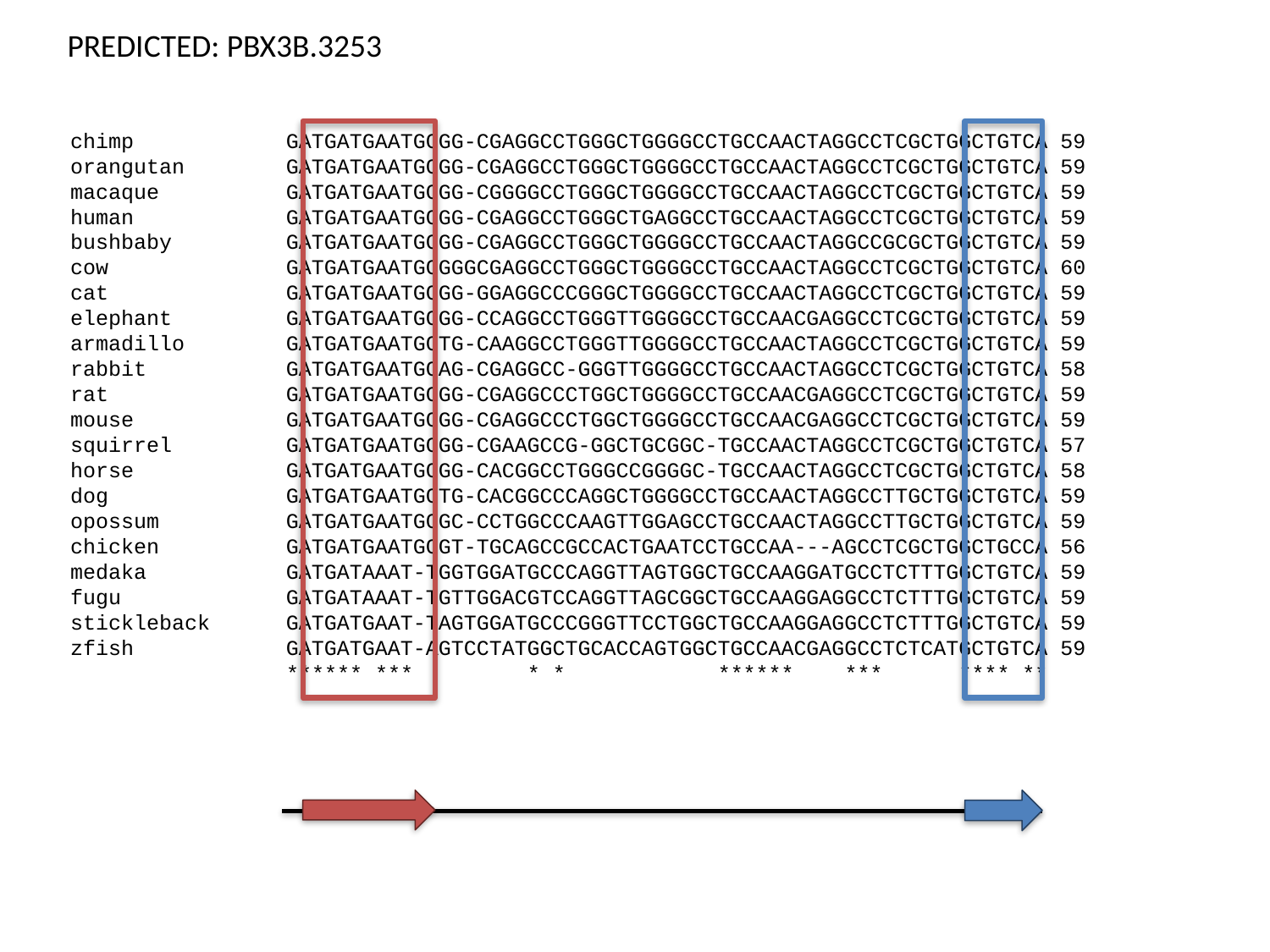

PREDICTED: PBX3B.3253
chimp GATGATGAATGCGG-CGAGGCCTGGGCTGGGGCCTGCCAACTAGGCCTCGCTGGCTGTCA 59
orangutan GATGATGAATGCGG-CGAGGCCTGGGCTGGGGCCTGCCAACTAGGCCTCGCTGGCTGTCA 59
macaque GATGATGAATGCGG-CGGGGCCTGGGCTGGGGCCTGCCAACTAGGCCTCGCTGGCTGTCA 59
human GATGATGAATGCGG-CGAGGCCTGGGCTGAGGCCTGCCAACTAGGCCTCGCTGGCTGTCA 59
bushbaby GATGATGAATGCGG-CGAGGCCTGGGCTGGGGCCTGCCAACTAGGCCGCGCTGGCTGTCA 59
cow GATGATGAATGCGGGCGAGGCCTGGGCTGGGGCCTGCCAACTAGGCCTCGCTGGCTGTCA 60
cat GATGATGAATGCGG-GGAGGCCCGGGCTGGGGCCTGCCAACTAGGCCTCGCTGGCTGTCA 59
elephant GATGATGAATGCGG-CCAGGCCTGGGTTGGGGCCTGCCAACGAGGCCTCGCTGGCTGTCA 59
armadillo GATGATGAATGCTG-CAAGGCCTGGGTTGGGGCCTGCCAACTAGGCCTCGCTGGCTGTCA 59
rabbit GATGATGAATGCAG-CGAGGCC-GGGTTGGGGCCTGCCAACTAGGCCTCGCTGGCTGTCA 58
rat GATGATGAATGCGG-CGAGGCCCTGGCTGGGGCCTGCCAACGAGGCCTCGCTGGCTGTCA 59
mouse GATGATGAATGCGG-CGAGGCCCTGGCTGGGGCCTGCCAACGAGGCCTCGCTGGCTGTCA 59
squirrel GATGATGAATGCGG-CGAAGCCG-GGCTGCGGC-TGCCAACTAGGCCTCGCTGGCTGTCA 57
horse GATGATGAATGCGG-CACGGCCTGGGCCGGGGC-TGCCAACTAGGCCTCGCTGGCTGTCA 58
dog GATGATGAATGCTG-CACGGCCCAGGCTGGGGCCTGCCAACTAGGCCTTGCTGGCTGTCA 59
opossum GATGATGAATGCGC-CCTGGCCCAAGTTGGAGCCTGCCAACTAGGCCTTGCTGGCTGTCA 59
chicken GATGATGAATGCGT-TGCAGCCGCCACTGAATCCTGCCAA---AGCCTCGCTGGCTGCCA 56
medaka GATGATAAAT-TGGTGGATGCCCAGGTTAGTGGCTGCCAAGGATGCCTCTTTGGCTGTCA 59
fugu GATGATAAAT-TGTTGGACGTCCAGGTTAGCGGCTGCCAAGGAGGCCTCTTTGGCTGTCA 59
stickleback GATGATGAAT-TAGTGGATGCCCGGGTTCCTGGCTGCCAAGGAGGCCTCTTTGGCTGTCA 59
zfish GATGATGAAT-AGTCCTATGGCTGCACCAGTGGCTGCCAACGAGGCCTCTCATGCTGTCA 59
 ****** *** * * ****** *** **** **

## Slide 50
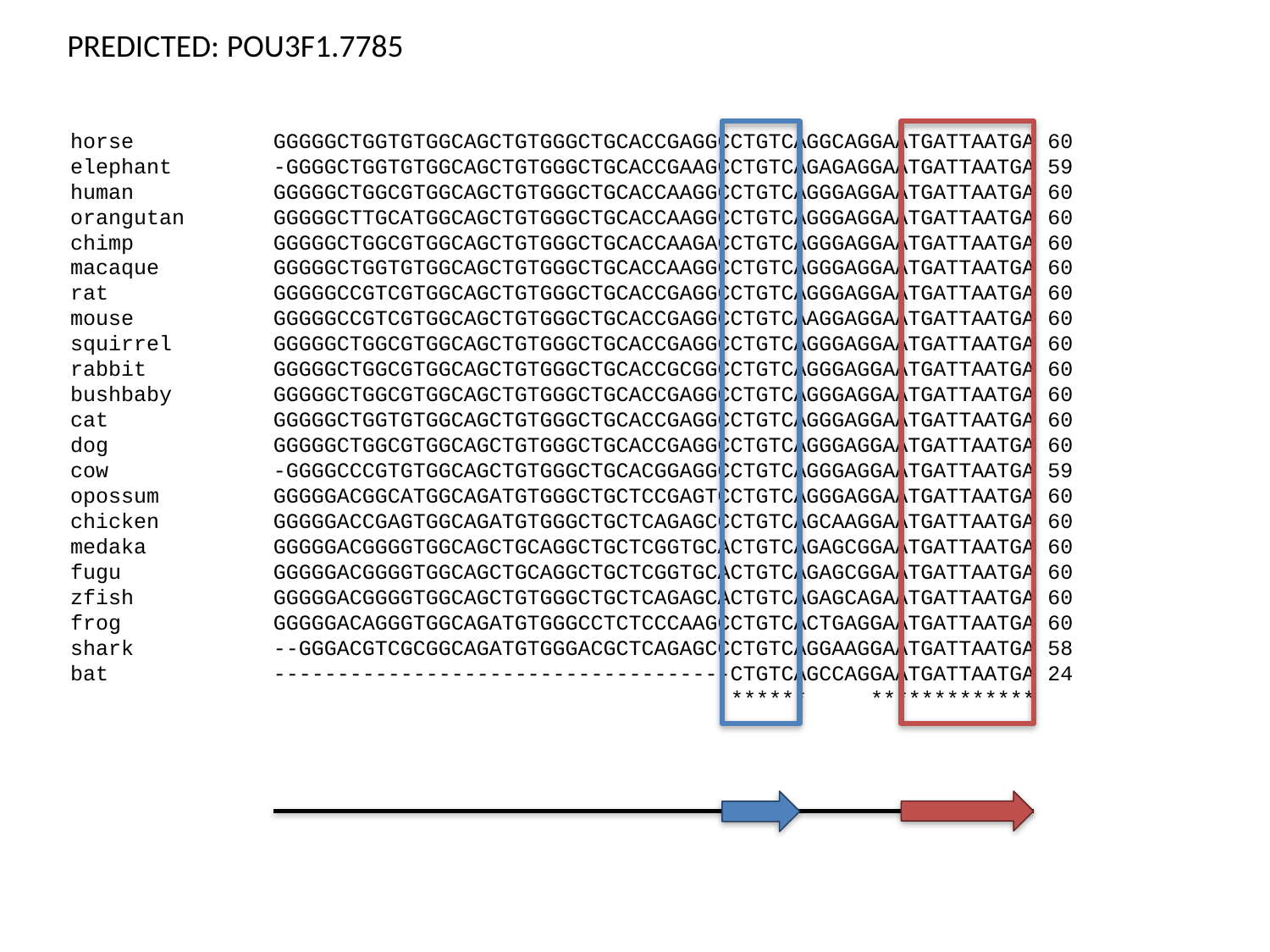

PREDICTED: POU3F1.7785
horse GGGGGCTGGTGTGGCAGCTGTGGGCTGCACCGAGGCCTGTCAGGCAGGAATGATTAATGA 60
elephant -GGGGCTGGTGTGGCAGCTGTGGGCTGCACCGAAGCCTGTCAGAGAGGAATGATTAATGA 59
human GGGGGCTGGCGTGGCAGCTGTGGGCTGCACCAAGGCCTGTCAGGGAGGAATGATTAATGA 60
orangutan GGGGGCTTGCATGGCAGCTGTGGGCTGCACCAAGGCCTGTCAGGGAGGAATGATTAATGA 60
chimp GGGGGCTGGCGTGGCAGCTGTGGGCTGCACCAAGACCTGTCAGGGAGGAATGATTAATGA 60
macaque GGGGGCTGGTGTGGCAGCTGTGGGCTGCACCAAGGCCTGTCAGGGAGGAATGATTAATGA 60
rat GGGGGCCGTCGTGGCAGCTGTGGGCTGCACCGAGGCCTGTCAGGGAGGAATGATTAATGA 60
mouse GGGGGCCGTCGTGGCAGCTGTGGGCTGCACCGAGGCCTGTCAAGGAGGAATGATTAATGA 60
squirrel GGGGGCTGGCGTGGCAGCTGTGGGCTGCACCGAGGCCTGTCAGGGAGGAATGATTAATGA 60
rabbit GGGGGCTGGCGTGGCAGCTGTGGGCTGCACCGCGGCCTGTCAGGGAGGAATGATTAATGA 60
bushbaby GGGGGCTGGCGTGGCAGCTGTGGGCTGCACCGAGGCCTGTCAGGGAGGAATGATTAATGA 60
cat GGGGGCTGGTGTGGCAGCTGTGGGCTGCACCGAGGCCTGTCAGGGAGGAATGATTAATGA 60
dog GGGGGCTGGCGTGGCAGCTGTGGGCTGCACCGAGGCCTGTCAGGGAGGAATGATTAATGA 60
cow -GGGGCCCGTGTGGCAGCTGTGGGCTGCACGGAGGCCTGTCAGGGAGGAATGATTAATGA 59
opossum GGGGGACGGCATGGCAGATGTGGGCTGCTCCGAGTCCTGTCAGGGAGGAATGATTAATGA 60
chicken GGGGGACCGAGTGGCAGATGTGGGCTGCTCAGAGCCCTGTCAGCAAGGAATGATTAATGA 60
medaka GGGGGACGGGGTGGCAGCTGCAGGCTGCTCGGTGCACTGTCAGAGCGGAATGATTAATGA 60
fugu GGGGGACGGGGTGGCAGCTGCAGGCTGCTCGGTGCACTGTCAGAGCGGAATGATTAATGA 60
zfish GGGGGACGGGGTGGCAGCTGTGGGCTGCTCAGAGCACTGTCAGAGCAGAATGATTAATGA 60
frog GGGGGACAGGGTGGCAGATGTGGGCCTCTCCCAAGCCTGTCACTGAGGAATGATTAATGA 60
shark --GGGACGTCGCGGCAGATGTGGGACGCTCAGAGCCCTGTCAGGAAGGAATGATTAATGA 58
bat ------------------------------------CTGTCAGCCAGGAATGATTAATGA 24
 ****** *************

## Slide 51
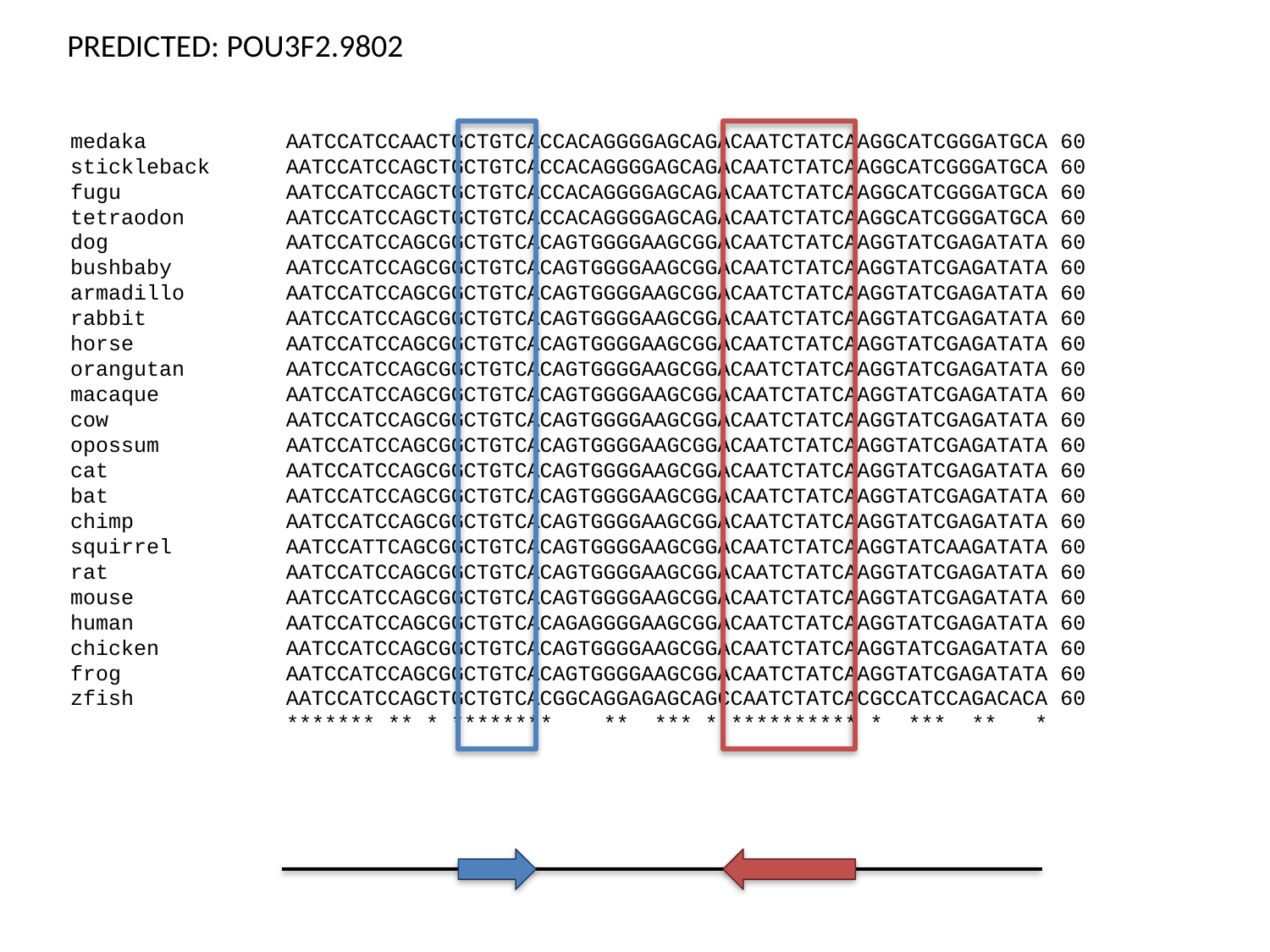

PREDICTED: POU3F2.9802
medaka AATCCATCCAACTGCTGTCACCACAGGGGAGCAGACAATCTATCAAGGCATCGGGATGCA 60
stickleback AATCCATCCAGCTGCTGTCACCACAGGGGAGCAGACAATCTATCAAGGCATCGGGATGCA 60
fugu AATCCATCCAGCTGCTGTCACCACAGGGGAGCAGACAATCTATCAAGGCATCGGGATGCA 60
tetraodon AATCCATCCAGCTGCTGTCACCACAGGGGAGCAGACAATCTATCAAGGCATCGGGATGCA 60
dog AATCCATCCAGCGGCTGTCACAGTGGGGAAGCGGACAATCTATCAAGGTATCGAGATATA 60
bushbaby AATCCATCCAGCGGCTGTCACAGTGGGGAAGCGGACAATCTATCAAGGTATCGAGATATA 60
armadillo AATCCATCCAGCGGCTGTCACAGTGGGGAAGCGGACAATCTATCAAGGTATCGAGATATA 60
rabbit AATCCATCCAGCGGCTGTCACAGTGGGGAAGCGGACAATCTATCAAGGTATCGAGATATA 60
horse AATCCATCCAGCGGCTGTCACAGTGGGGAAGCGGACAATCTATCAAGGTATCGAGATATA 60
orangutan AATCCATCCAGCGGCTGTCACAGTGGGGAAGCGGACAATCTATCAAGGTATCGAGATATA 60
macaque AATCCATCCAGCGGCTGTCACAGTGGGGAAGCGGACAATCTATCAAGGTATCGAGATATA 60
cow AATCCATCCAGCGGCTGTCACAGTGGGGAAGCGGACAATCTATCAAGGTATCGAGATATA 60
opossum AATCCATCCAGCGGCTGTCACAGTGGGGAAGCGGACAATCTATCAAGGTATCGAGATATA 60
cat AATCCATCCAGCGGCTGTCACAGTGGGGAAGCGGACAATCTATCAAGGTATCGAGATATA 60
bat AATCCATCCAGCGGCTGTCACAGTGGGGAAGCGGACAATCTATCAAGGTATCGAGATATA 60
chimp AATCCATCCAGCGGCTGTCACAGTGGGGAAGCGGACAATCTATCAAGGTATCGAGATATA 60
squirrel AATCCATTCAGCGGCTGTCACAGTGGGGAAGCGGACAATCTATCAAGGTATCAAGATATA 60
rat AATCCATCCAGCGGCTGTCACAGTGGGGAAGCGGACAATCTATCAAGGTATCGAGATATA 60
mouse AATCCATCCAGCGGCTGTCACAGTGGGGAAGCGGACAATCTATCAAGGTATCGAGATATA 60
human AATCCATCCAGCGGCTGTCACAGAGGGGAAGCGGACAATCTATCAAGGTATCGAGATATA 60
chicken AATCCATCCAGCGGCTGTCACAGTGGGGAAGCGGACAATCTATCAAGGTATCGAGATATA 60
frog AATCCATCCAGCGGCTGTCACAGTGGGGAAGCGGACAATCTATCAAGGTATCGAGATATA 60
zfish AATCCATCCAGCTGCTGTCACGGCAGGAGAGCAGCCAATCTATCACGCCATCCAGACACA 60
 ******* ** * ******** ** *** * ********** * *** ** *

## Slide 52
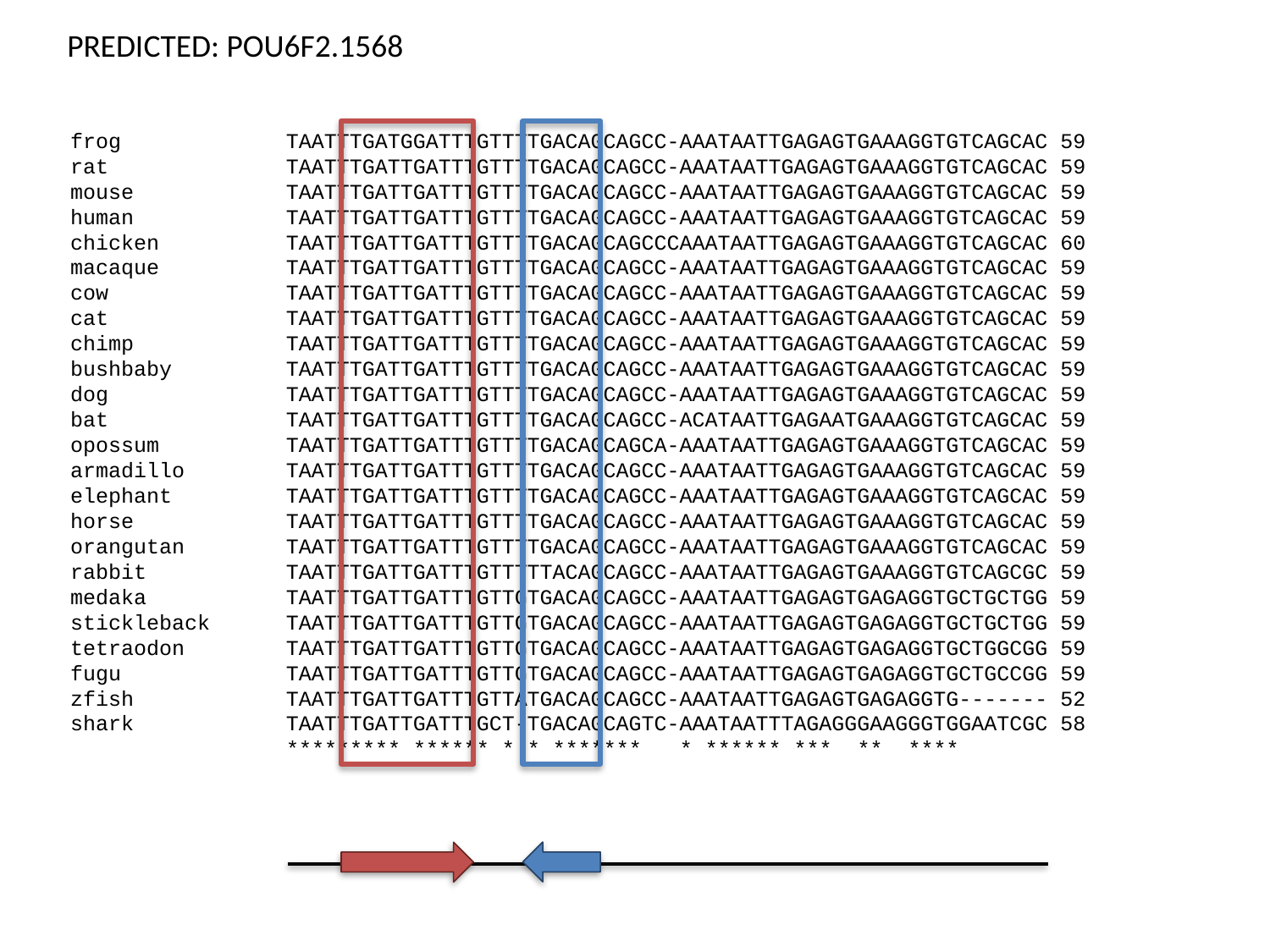

PREDICTED: POU6F2.1568
frog TAATTTGATGGATTTGTTTTGACAGCAGCC-AAATAATTGAGAGTGAAAGGTGTCAGCAC 59
rat TAATTTGATTGATTTGTTTTGACAGCAGCC-AAATAATTGAGAGTGAAAGGTGTCAGCAC 59
mouse TAATTTGATTGATTTGTTTTGACAGCAGCC-AAATAATTGAGAGTGAAAGGTGTCAGCAC 59
human TAATTTGATTGATTTGTTTTGACAGCAGCC-AAATAATTGAGAGTGAAAGGTGTCAGCAC 59
chicken TAATTTGATTGATTTGTTTTGACAGCAGCCCAAATAATTGAGAGTGAAAGGTGTCAGCAC 60
macaque TAATTTGATTGATTTGTTTTGACAGCAGCC-AAATAATTGAGAGTGAAAGGTGTCAGCAC 59
cow TAATTTGATTGATTTGTTTTGACAGCAGCC-AAATAATTGAGAGTGAAAGGTGTCAGCAC 59
cat TAATTTGATTGATTTGTTTTGACAGCAGCC-AAATAATTGAGAGTGAAAGGTGTCAGCAC 59
chimp TAATTTGATTGATTTGTTTTGACAGCAGCC-AAATAATTGAGAGTGAAAGGTGTCAGCAC 59
bushbaby TAATTTGATTGATTTGTTTTGACAGCAGCC-AAATAATTGAGAGTGAAAGGTGTCAGCAC 59
dog TAATTTGATTGATTTGTTTTGACAGCAGCC-AAATAATTGAGAGTGAAAGGTGTCAGCAC 59
bat TAATTTGATTGATTTGTTTTGACAGCAGCC-ACATAATTGAGAATGAAAGGTGTCAGCAC 59
opossum TAATTTGATTGATTTGTTTTGACAGCAGCA-AAATAATTGAGAGTGAAAGGTGTCAGCAC 59
armadillo TAATTTGATTGATTTGTTTTGACAGCAGCC-AAATAATTGAGAGTGAAAGGTGTCAGCAC 59
elephant TAATTTGATTGATTTGTTTTGACAGCAGCC-AAATAATTGAGAGTGAAAGGTGTCAGCAC 59
horse TAATTTGATTGATTTGTTTTGACAGCAGCC-AAATAATTGAGAGTGAAAGGTGTCAGCAC 59
orangutan TAATTTGATTGATTTGTTTTGACAGCAGCC-AAATAATTGAGAGTGAAAGGTGTCAGCAC 59
rabbit TAATTTGATTGATTTGTTTTTACAGCAGCC-AAATAATTGAGAGTGAAAGGTGTCAGCGC 59
medaka TAATTTGATTGATTTGTTGTGACAGCAGCC-AAATAATTGAGAGTGAGAGGTGCTGCTGG 59
stickleback TAATTTGATTGATTTGTTGTGACAGCAGCC-AAATAATTGAGAGTGAGAGGTGCTGCTGG 59
tetraodon TAATTTGATTGATTTGTTGTGACAGCAGCC-AAATAATTGAGAGTGAGAGGTGCTGGCGG 59
fugu TAATTTGATTGATTTGTTGTGACAGCAGCC-AAATAATTGAGAGTGAGAGGTGCTGCCGG 59
zfish TAATTTGATTGATTTGTTATGACAGCAGCC-AAATAATTGAGAGTGAGAGGTG------- 52
shark TAATTTGATTGATTTGCT-TGACAGCAGTC-AAATAATTTAGAGGGAAGGGTGGAATCGC 58
 ********* ****** * * ******* * ****** *** ** ****

## Slide 53
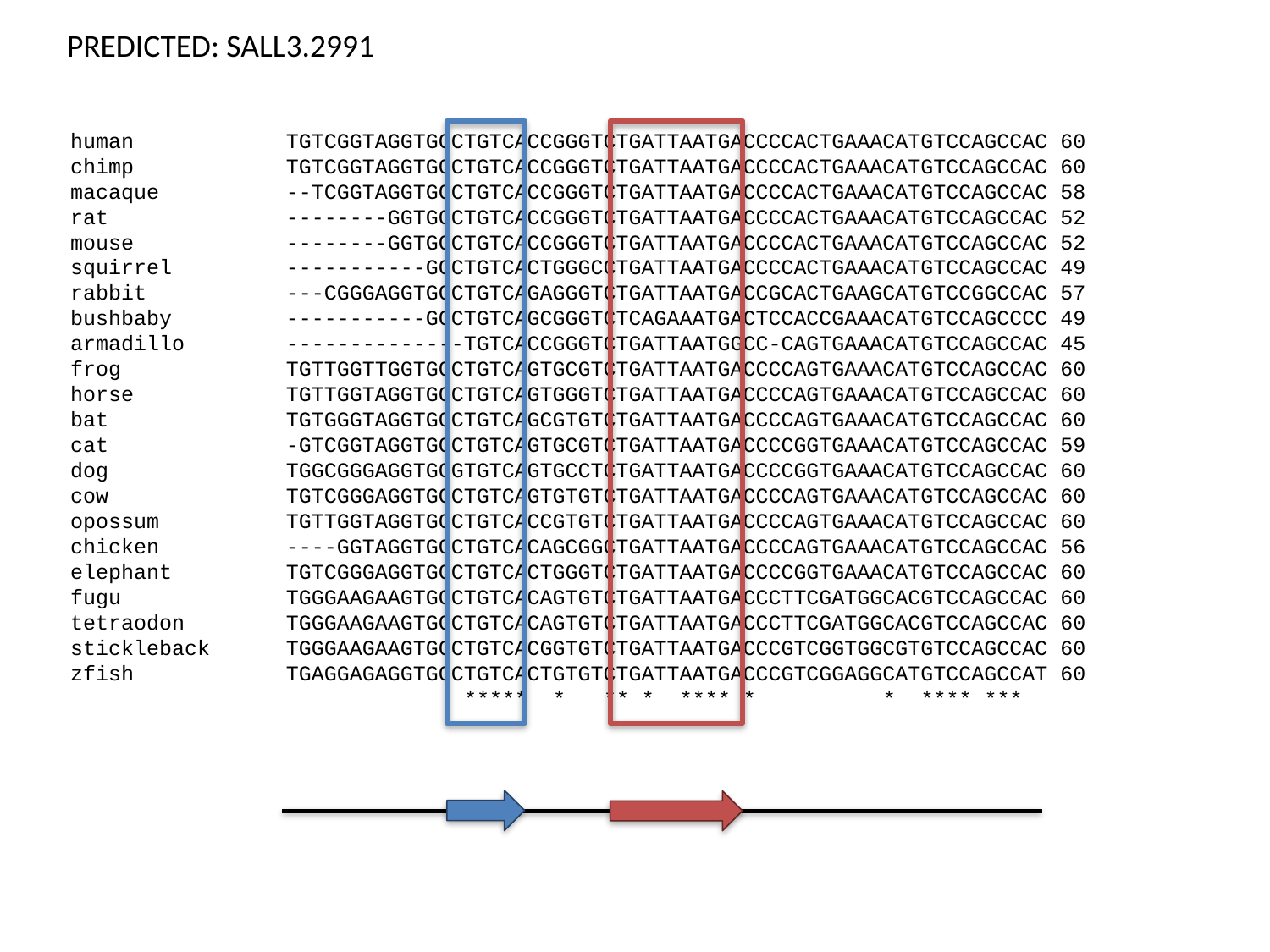

PREDICTED: SALL3.2991
human TGTCGGTAGGTGGCTGTCACCGGGTCTGATTAATGACCCCACTGAAACATGTCCAGCCAC 60
chimp TGTCGGTAGGTGGCTGTCACCGGGTCTGATTAATGACCCCACTGAAACATGTCCAGCCAC 60
macaque --TCGGTAGGTGGCTGTCACCGGGTCTGATTAATGACCCCACTGAAACATGTCCAGCCAC 58
rat --------GGTGGCTGTCACCGGGTCTGATTAATGACCCCACTGAAACATGTCCAGCCAC 52
mouse --------GGTGGCTGTCACCGGGTCTGATTAATGACCCCACTGAAACATGTCCAGCCAC 52
squirrel -----------GGCTGTCACTGGGCCTGATTAATGACCCCACTGAAACATGTCCAGCCAC 49
rabbit ---CGGGAGGTGGCTGTCAGAGGGTCTGATTAATGACCGCACTGAAGCATGTCCGGCCAC 57
bushbaby -----------GGCTGTCAGCGGGTCTCAGAAATGACTCCACCGAAACATGTCCAGCCCC 49
armadillo --------------TGTCACCGGGTCTGATTAATGGCC-CAGTGAAACATGTCCAGCCAC 45
frog TGTTGGTTGGTGGCTGTCAGTGCGTCTGATTAATGACCCCAGTGAAACATGTCCAGCCAC 60
horse TGTTGGTAGGTGGCTGTCAGTGGGTCTGATTAATGACCCCAGTGAAACATGTCCAGCCAC 60
bat TGTGGGTAGGTGGCTGTCAGCGTGTCTGATTAATGACCCCAGTGAAACATGTCCAGCCAC 60
cat -GTCGGTAGGTGGCTGTCAGTGCGTCTGATTAATGACCCCGGTGAAACATGTCCAGCCAC 59
dog TGGCGGGAGGTGGGTGTCAGTGCCTCTGATTAATGACCCCGGTGAAACATGTCCAGCCAC 60
cow TGTCGGGAGGTGGCTGTCAGTGTGTCTGATTAATGACCCCAGTGAAACATGTCCAGCCAC 60
opossum TGTTGGTAGGTGGCTGTCACCGTGTCTGATTAATGACCCCAGTGAAACATGTCCAGCCAC 60
chicken ----GGTAGGTGGCTGTCACAGCGGCTGATTAATGACCCCAGTGAAACATGTCCAGCCAC 56
elephant TGTCGGGAGGTGGCTGTCACTGGGTCTGATTAATGACCCCGGTGAAACATGTCCAGCCAC 60
fugu TGGGAAGAAGTGGCTGTCACAGTGTCTGATTAATGACCCTTCGATGGCACGTCCAGCCAC 60
tetraodon TGGGAAGAAGTGGCTGTCACAGTGTCTGATTAATGACCCTTCGATGGCACGTCCAGCCAC 60
stickleback TGGGAAGAAGTGGCTGTCACGGTGTCTGATTAATGACCCGTCGGTGGCGTGTCCAGCCAC 60
zfish TGAGGAGAGGTGGCTGTCACTGTGTCTGATTAATGACCCGTCGGAGGCATGTCCAGCCAT 60
 ***** * ** * **** * * **** ***

## Slide 54
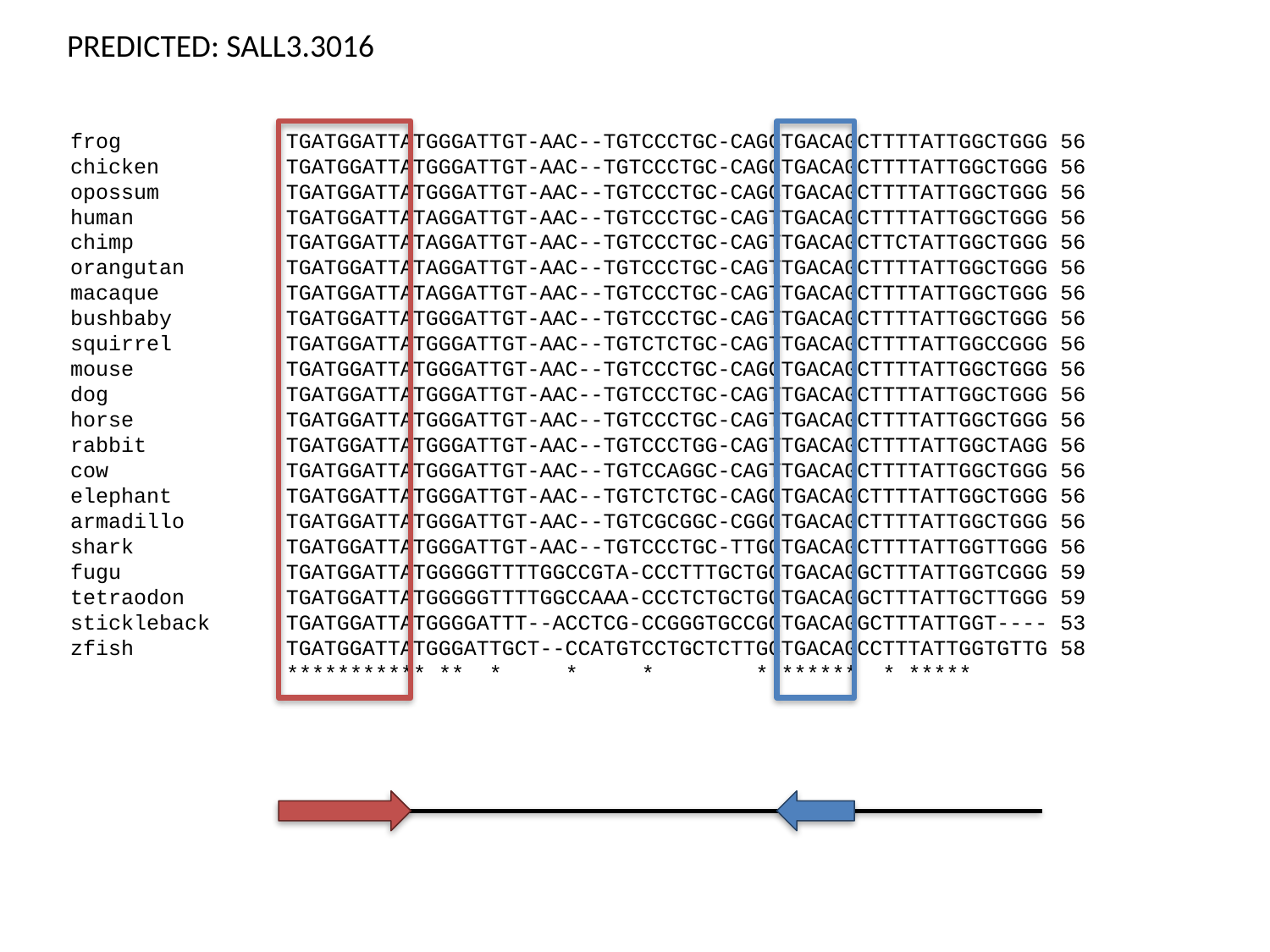

PREDICTED: SALL3.3016
frog TGATGGATTATGGGATTGT-AAC--TGTCCCTGC-CAGCTGACAGCTTTTATTGGCTGGG 56
chicken TGATGGATTATGGGATTGT-AAC--TGTCCCTGC-CAGCTGACAGCTTTTATTGGCTGGG 56
opossum TGATGGATTATGGGATTGT-AAC--TGTCCCTGC-CAGCTGACAGCTTTTATTGGCTGGG 56
human TGATGGATTATAGGATTGT-AAC--TGTCCCTGC-CAGTTGACAGCTTTTATTGGCTGGG 56
chimp TGATGGATTATAGGATTGT-AAC--TGTCCCTGC-CAGTTGACAGCTTCTATTGGCTGGG 56
orangutan TGATGGATTATAGGATTGT-AAC--TGTCCCTGC-CAGTTGACAGCTTTTATTGGCTGGG 56
macaque TGATGGATTATAGGATTGT-AAC--TGTCCCTGC-CAGTTGACAGCTTTTATTGGCTGGG 56
bushbaby TGATGGATTATGGGATTGT-AAC--TGTCCCTGC-CAGTTGACAGCTTTTATTGGCTGGG 56
squirrel TGATGGATTATGGGATTGT-AAC--TGTCTCTGC-CAGTTGACAGCTTTTATTGGCCGGG 56
mouse TGATGGATTATGGGATTGT-AAC--TGTCCCTGC-CAGGTGACAGCTTTTATTGGCTGGG 56
dog TGATGGATTATGGGATTGT-AAC--TGTCCCTGC-CAGTTGACAGCTTTTATTGGCTGGG 56
horse TGATGGATTATGGGATTGT-AAC--TGTCCCTGC-CAGTTGACAGCTTTTATTGGCTGGG 56
rabbit TGATGGATTATGGGATTGT-AAC--TGTCCCTGG-CAGTTGACAGCTTTTATTGGCTAGG 56
cow TGATGGATTATGGGATTGT-AAC--TGTCCAGGC-CAGTTGACAGCTTTTATTGGCTGGG 56
elephant TGATGGATTATGGGATTGT-AAC--TGTCTCTGC-CAGCTGACAGCTTTTATTGGCTGGG 56
armadillo TGATGGATTATGGGATTGT-AAC--TGTCGCGGC-CGGCTGACAGCTTTTATTGGCTGGG 56
shark TGATGGATTATGGGATTGT-AAC--TGTCCCTGC-TTGCTGACAGCTTTTATTGGTTGGG 56
fugu TGATGGATTATGGGGGTTTTGGCCGTA-CCCTTTGCTGCTGACAGGCTTTATTGGTCGGG 59
tetraodon TGATGGATTATGGGGGTTTTGGCCAAA-CCCTCTGCTGCTGACAGGCTTTATTGCTTGGG 59
stickleback TGATGGATTATGGGGATTT--ACCTCG-CCGGGTGCCGCTGACAGGCTTTATTGGT---- 53
zfish TGATGGATTATGGGATTGCT--CCATGTCCTGCTCTTGCTGACAGCCTTTATTGGTGTTG 58
 *********** ** * * * * ****** * *****

## Slide 55
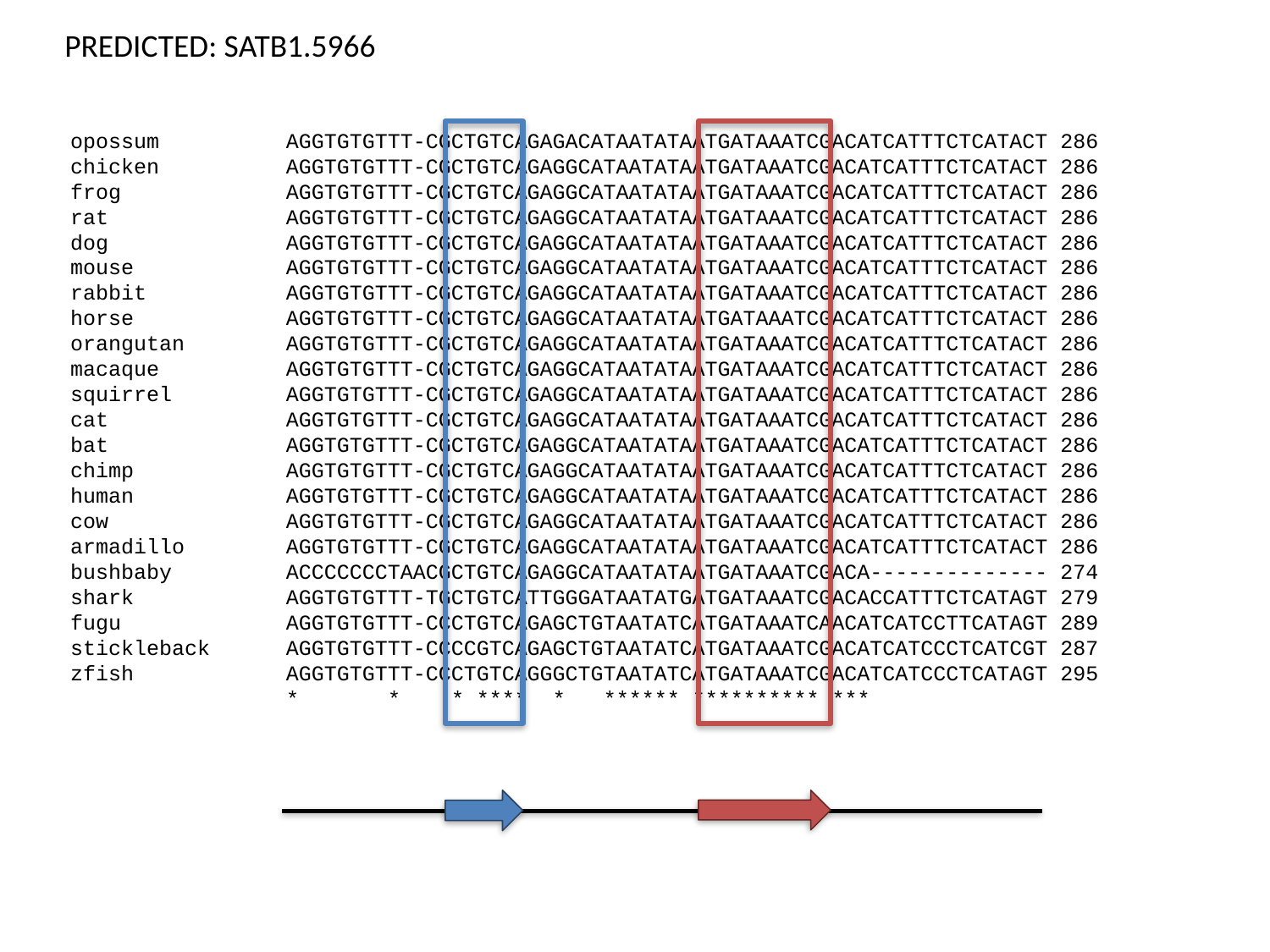

PREDICTED: SATB1.5966
opossum AGGTGTGTTT-CGCTGTCAGAGACATAATATAATGATAAATCGACATCATTTCTCATACT 286
chicken AGGTGTGTTT-CGCTGTCAGAGGCATAATATAATGATAAATCGACATCATTTCTCATACT 286
frog AGGTGTGTTT-CGCTGTCAGAGGCATAATATAATGATAAATCGACATCATTTCTCATACT 286
rat AGGTGTGTTT-CGCTGTCAGAGGCATAATATAATGATAAATCGACATCATTTCTCATACT 286
dog AGGTGTGTTT-CGCTGTCAGAGGCATAATATAATGATAAATCGACATCATTTCTCATACT 286
mouse AGGTGTGTTT-CGCTGTCAGAGGCATAATATAATGATAAATCGACATCATTTCTCATACT 286
rabbit AGGTGTGTTT-CGCTGTCAGAGGCATAATATAATGATAAATCGACATCATTTCTCATACT 286
horse AGGTGTGTTT-CGCTGTCAGAGGCATAATATAATGATAAATCGACATCATTTCTCATACT 286
orangutan AGGTGTGTTT-CGCTGTCAGAGGCATAATATAATGATAAATCGACATCATTTCTCATACT 286
macaque AGGTGTGTTT-CGCTGTCAGAGGCATAATATAATGATAAATCGACATCATTTCTCATACT 286
squirrel AGGTGTGTTT-CGCTGTCAGAGGCATAATATAATGATAAATCGACATCATTTCTCATACT 286
cat AGGTGTGTTT-CGCTGTCAGAGGCATAATATAATGATAAATCGACATCATTTCTCATACT 286
bat AGGTGTGTTT-CGCTGTCAGAGGCATAATATAATGATAAATCGACATCATTTCTCATACT 286
chimp AGGTGTGTTT-CGCTGTCAGAGGCATAATATAATGATAAATCGACATCATTTCTCATACT 286
human AGGTGTGTTT-CGCTGTCAGAGGCATAATATAATGATAAATCGACATCATTTCTCATACT 286
cow AGGTGTGTTT-CGCTGTCAGAGGCATAATATAATGATAAATCGACATCATTTCTCATACT 286
armadillo AGGTGTGTTT-CGCTGTCAGAGGCATAATATAATGATAAATCGACATCATTTCTCATACT 286
bushbaby ACCCCCCCTAACGCTGTCAGAGGCATAATATAATGATAAATCGACA-------------- 274
shark AGGTGTGTTT-TGCTGTCATTGGGATAATATGATGATAAATCGACACCATTTCTCATAGT 279
fugu AGGTGTGTTT-CCCTGTCAGAGCTGTAATATCATGATAAATCAACATCATCCTTCATAGT 289
stickleback AGGTGTGTTT-CCCCGTCAGAGCTGTAATATCATGATAAATCGACATCATCCCTCATCGT 287
zfish AGGTGTGTTT-CCCTGTCAGGGCTGTAATATCATGATAAATCGACATCATCCCTCATAGT 295
 * * * **** * ****** ********** ***

## Slide 56
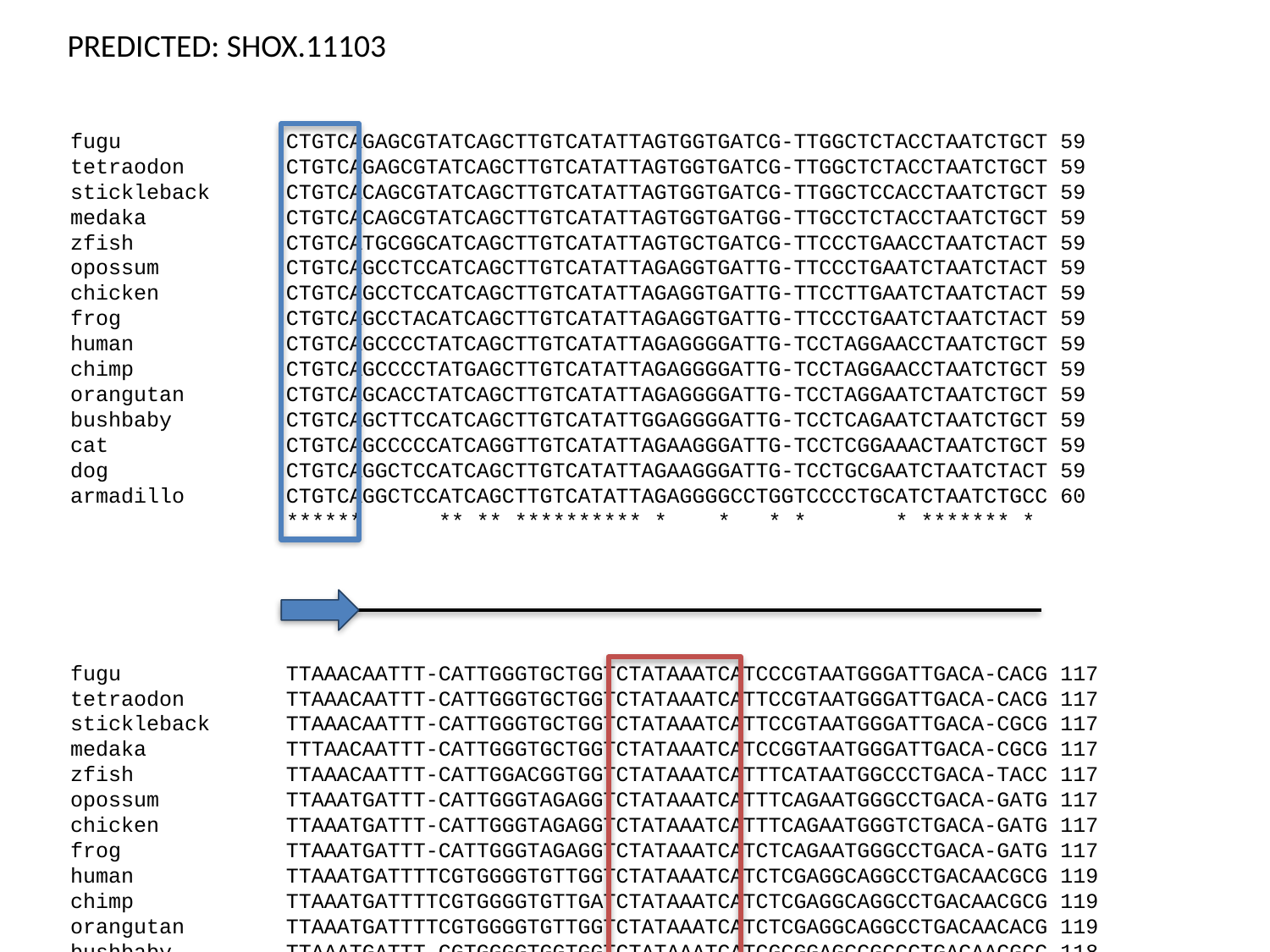

PREDICTED: SHOX.11103
fugu CTGTCAGAGCGTATCAGCTTGTCATATTAGTGGTGATCG-TTGGCTCTACCTAATCTGCT 59
tetraodon CTGTCAGAGCGTATCAGCTTGTCATATTAGTGGTGATCG-TTGGCTCTACCTAATCTGCT 59
stickleback CTGTCACAGCGTATCAGCTTGTCATATTAGTGGTGATCG-TTGGCTCCACCTAATCTGCT 59
medaka CTGTCACAGCGTATCAGCTTGTCATATTAGTGGTGATGG-TTGCCTCTACCTAATCTGCT 59
zfish CTGTCATGCGGCATCAGCTTGTCATATTAGTGCTGATCG-TTCCCTGAACCTAATCTACT 59
opossum CTGTCAGCCTCCATCAGCTTGTCATATTAGAGGTGATTG-TTCCCTGAATCTAATCTACT 59
chicken CTGTCAGCCTCCATCAGCTTGTCATATTAGAGGTGATTG-TTCCTTGAATCTAATCTACT 59
frog CTGTCAGCCTACATCAGCTTGTCATATTAGAGGTGATTG-TTCCCTGAATCTAATCTACT 59
human CTGTCAGCCCCTATCAGCTTGTCATATTAGAGGGGATTG-TCCTAGGAACCTAATCTGCT 59
chimp CTGTCAGCCCCTATGAGCTTGTCATATTAGAGGGGATTG-TCCTAGGAACCTAATCTGCT 59
orangutan CTGTCAGCACCTATCAGCTTGTCATATTAGAGGGGATTG-TCCTAGGAATCTAATCTGCT 59
bushbaby CTGTCAGCTTCCATCAGCTTGTCATATTGGAGGGGATTG-TCCTCAGAATCTAATCTGCT 59
cat CTGTCAGCCCCCATCAGGTTGTCATATTAGAAGGGATTG-TCCTCGGAAACTAATCTGCT 59
dog CTGTCAGGCTCCATCAGCTTGTCATATTAGAAGGGATTG-TCCTGCGAATCTAATCTACT 59
armadillo CTGTCAGGCTCCATCAGCTTGTCATATTAGAGGGGCCTGGTCCCCTGCATCTAATCTGCC 60
 ****** ** ** ********** * * * * * ******* *
fugu TTAAACAATTT-CATTGGGTGCTGGTCTATAAATCATCCCGTAATGGGATTGACA-CACG 117
tetraodon TTAAACAATTT-CATTGGGTGCTGGTCTATAAATCATTCCGTAATGGGATTGACA-CACG 117
stickleback TTAAACAATTT-CATTGGGTGCTGGTCTATAAATCATTCCGTAATGGGATTGACA-CGCG 117
medaka TTTAACAATTT-CATTGGGTGCTGGTCTATAAATCATCCGGTAATGGGATTGACA-CGCG 117
zfish TTAAACAATTT-CATTGGACGGTGGTCTATAAATCATTTCATAATGGCCCTGACA-TACC 117
opossum TTAAATGATTT-CATTGGGTAGAGGTCTATAAATCATTTCAGAATGGGCCTGACA-GATG 117
chicken TTAAATGATTT-CATTGGGTAGAGGTCTATAAATCATTTCAGAATGGGTCTGACA-GATG 117
frog TTAAATGATTT-CATTGGGTAGAGGTCTATAAATCATCTCAGAATGGGCCTGACA-GATG 117
human TTAAATGATTTTCGTGGGGTGTTGGTCTATAAATCATCTCGAGGCAGGCCTGACAACGCG 119
chimp TTAAATGATTTTCGTGGGGTGTTGATCTATAAATCATCTCGAGGCAGGCCTGACAACGCG 119
orangutan TTAAATGATTTTCGTGGGGTGTTGGTCTATAAATCATCTCGAGGCAGGCCTGACAACACG 119
bushbaby TTAAATGATTT-CGTGGGGTGGTGGTCTATAAATCATCGCGGAGCCGCCCTGACAACGCC 118
cat TTAAATGATTT-CGTGGGGTGCTGGTCTATAAATCATGTCTGAGGCGCCCTGACA-TGGC 117
dog TTAAATGATTT-CGTGGGGTGGTGGTCTATAAATCATTTCAGAATCGCCCTGACA-CGTC 117
armadillo TTAAATGGTTT-CGTGGGGCGGTGGTCTATAAATCATCTCGGAATGGGCCTGACA-CGTG 118
 ** ** *** * * ** * ************ * *****

## Slide 57
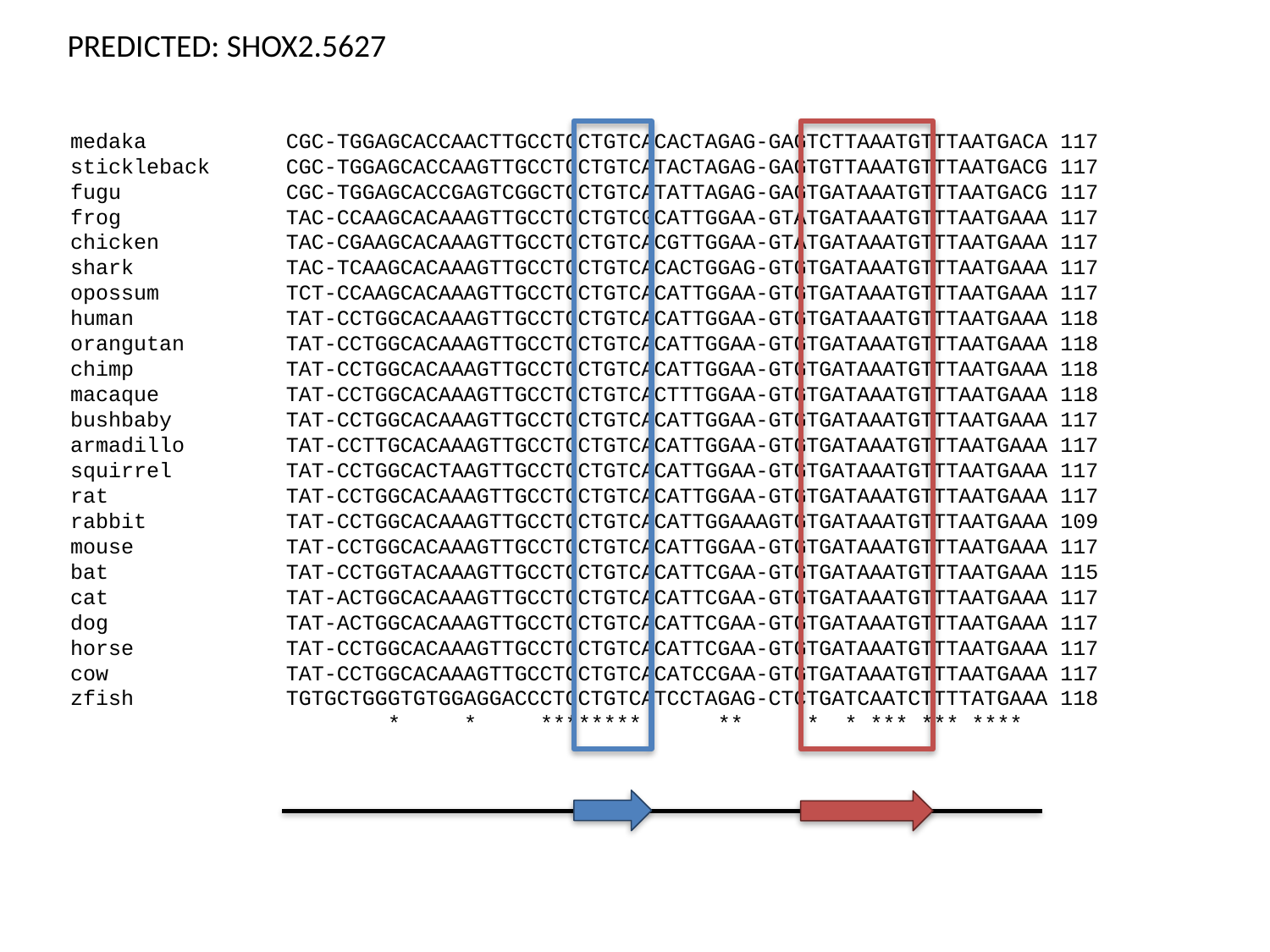

PREDICTED: SHOX2.5627
medaka CGC-TGGAGCACCAACTTGCCTGCTGTCACACTAGAG-GAGTCTTAAATGTTTAATGACA 117
stickleback CGC-TGGAGCACCAAGTTGCCTGCTGTCATACTAGAG-GAGTGTTAAATGTTTAATGACG 117
fugu CGC-TGGAGCACCGAGTCGGCTGCTGTCATATTAGAG-GAGTGATAAATGTTTAATGACG 117
frog TAC-CCAAGCACAAAGTTGCCTGCTGTCGCATTGGAA-GTATGATAAATGTTTAATGAAA 117
chicken TAC-CGAAGCACAAAGTTGCCTGCTGTCACGTTGGAA-GTATGATAAATGTTTAATGAAA 117
shark TAC-TCAAGCACAAAGTTGCCTGCTGTCACACTGGAG-GTGTGATAAATGTTTAATGAAA 117
opossum TCT-CCAAGCACAAAGTTGCCTGCTGTCACATTGGAA-GTGTGATAAATGTTTAATGAAA 117
human TAT-CCTGGCACAAAGTTGCCTGCTGTCACATTGGAA-GTGTGATAAATGTTTAATGAAA 118
orangutan TAT-CCTGGCACAAAGTTGCCTGCTGTCACATTGGAA-GTGTGATAAATGTTTAATGAAA 118
chimp TAT-CCTGGCACAAAGTTGCCTGCTGTCACATTGGAA-GTGTGATAAATGTTTAATGAAA 118
macaque TAT-CCTGGCACAAAGTTGCCTGCTGTCACTTTGGAA-GTGTGATAAATGTTTAATGAAA 118
bushbaby TAT-CCTGGCACAAAGTTGCCTGCTGTCACATTGGAA-GTGTGATAAATGTTTAATGAAA 117
armadillo TAT-CCTTGCACAAAGTTGCCTGCTGTCACATTGGAA-GTGTGATAAATGTTTAATGAAA 117
squirrel TAT-CCTGGCACTAAGTTGCCTGCTGTCACATTGGAA-GTGTGATAAATGTTTAATGAAA 117
rat TAT-CCTGGCACAAAGTTGCCTGCTGTCACATTGGAA-GTGTGATAAATGTTTAATGAAA 117
rabbit TAT-CCTGGCACAAAGTTGCCTGCTGTCACATTGGAAAGTGTGATAAATGTTTAATGAAA 109
mouse TAT-CCTGGCACAAAGTTGCCTGCTGTCACATTGGAA-GTGTGATAAATGTTTAATGAAA 117
bat TAT-CCTGGTACAAAGTTGCCTGCTGTCACATTCGAA-GTGTGATAAATGTTTAATGAAA 115
cat TAT-ACTGGCACAAAGTTGCCTGCTGTCACATTCGAA-GTGTGATAAATGTTTAATGAAA 117
dog TAT-ACTGGCACAAAGTTGCCTGCTGTCACATTCGAA-GTGTGATAAATGTTTAATGAAA 117
horse TAT-CCTGGCACAAAGTTGCCTGCTGTCACATTCGAA-GTGTGATAAATGTTTAATGAAA 117
cow TAT-CCTGGCACAAAGTTGCCTGCTGTCACATCCGAA-GTGTGATAAATGTTTAATGAAA 117
zfish TGTGCTGGGTGTGGAGGACCCTGCTGTCATCCTAGAG-CTCTGATCAATCTTTTATGAAA 118
 * * ******** ** * * *** *** ****

## Slide 58
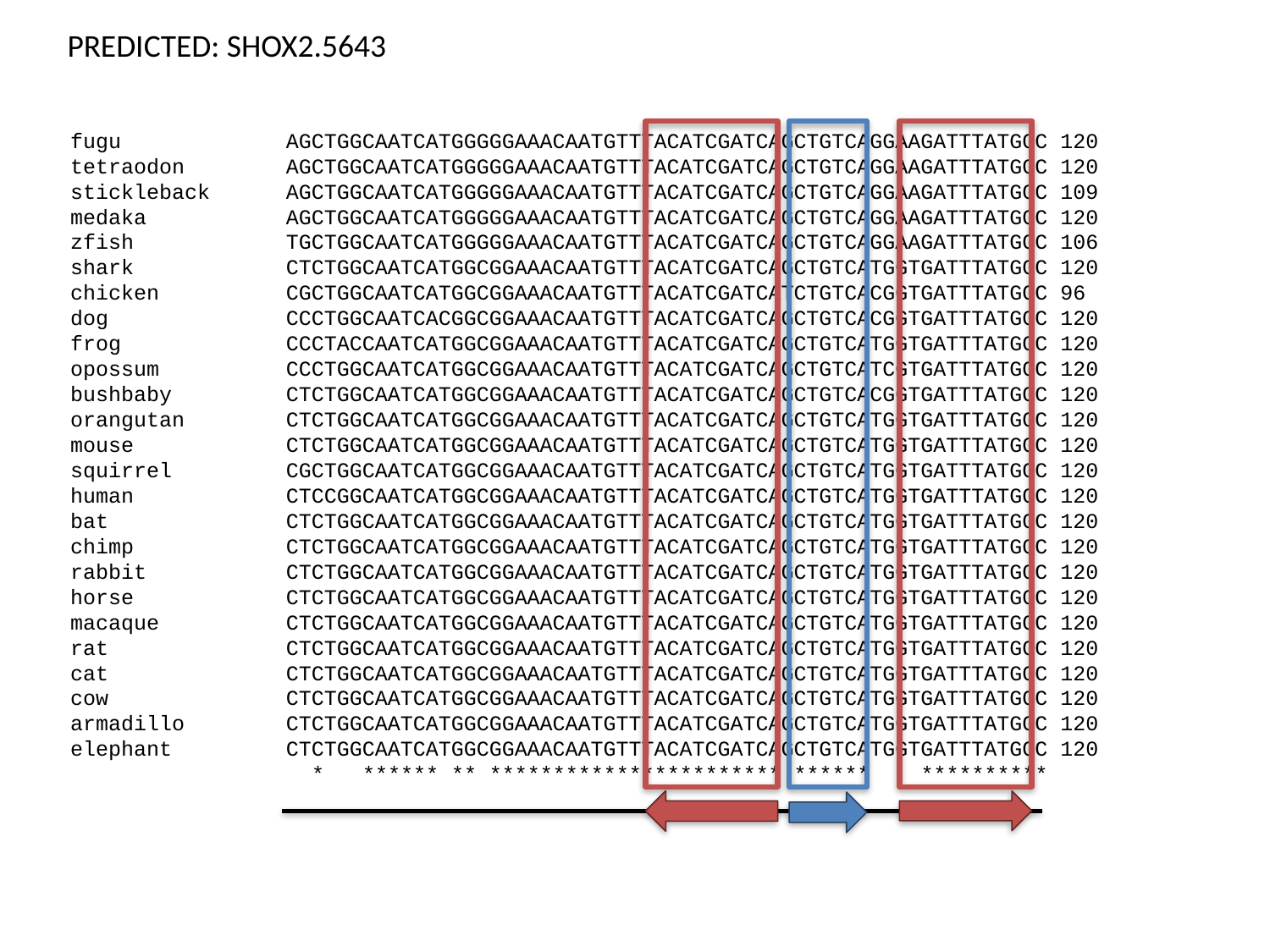

PREDICTED: SHOX2.5643
fugu AGCTGGCAATCATGGGGGAAACAATGTTTACATCGATCAGCTGTCAGGAAGATTTATGGC 120
tetraodon AGCTGGCAATCATGGGGGAAACAATGTTTACATCGATCAGCTGTCAGGAAGATTTATGGC 120
stickleback AGCTGGCAATCATGGGGGAAACAATGTTTACATCGATCAGCTGTCAGGAAGATTTATGGC 109
medaka AGCTGGCAATCATGGGGGAAACAATGTTTACATCGATCAGCTGTCAGGAAGATTTATGGC 120
zfish TGCTGGCAATCATGGGGGAAACAATGTTTACATCGATCAGCTGTCAGGAAGATTTATGGC 106
shark CTCTGGCAATCATGGCGGAAACAATGTTTACATCGATCAGCTGTCATGGTGATTTATGGC 120
chicken CGCTGGCAATCATGGCGGAAACAATGTTTACATCGATCATCTGTCACGGTGATTTATGGC 96
dog CCCTGGCAATCACGGCGGAAACAATGTTTACATCGATCAGCTGTCACGGTGATTTATGGC 120
frog CCCTACCAATCATGGCGGAAACAATGTTTACATCGATCAGCTGTCATGGTGATTTATGGC 120
opossum CCCTGGCAATCATGGCGGAAACAATGTTTACATCGATCAGCTGTCATCGTGATTTATGGC 120
bushbaby CTCTGGCAATCATGGCGGAAACAATGTTTACATCGATCAGCTGTCACGGTGATTTATGGC 120
orangutan CTCTGGCAATCATGGCGGAAACAATGTTTACATCGATCAGCTGTCATGGTGATTTATGGC 120
mouse CTCTGGCAATCATGGCGGAAACAATGTTTACATCGATCAGCTGTCATGGTGATTTATGGC 120
squirrel CGCTGGCAATCATGGCGGAAACAATGTTTACATCGATCAGCTGTCATGGTGATTTATGGC 120
human CTCCGGCAATCATGGCGGAAACAATGTTTACATCGATCAGCTGTCATGGTGATTTATGGC 120
bat CTCTGGCAATCATGGCGGAAACAATGTTTACATCGATCAGCTGTCATGGTGATTTATGGC 120
chimp CTCTGGCAATCATGGCGGAAACAATGTTTACATCGATCAGCTGTCATGGTGATTTATGGC 120
rabbit CTCTGGCAATCATGGCGGAAACAATGTTTACATCGATCAGCTGTCATGGTGATTTATGGC 120
horse CTCTGGCAATCATGGCGGAAACAATGTTTACATCGATCAGCTGTCATGGTGATTTATGGC 120
macaque CTCTGGCAATCATGGCGGAAACAATGTTTACATCGATCAGCTGTCATGGTGATTTATGGC 120
rat CTCTGGCAATCATGGCGGAAACAATGTTTACATCGATCAGCTGTCATGGTGATTTATGGC 120
cat CTCTGGCAATCATGGCGGAAACAATGTTTACATCGATCAGCTGTCATGGTGATTTATGGC 120
cow CTCTGGCAATCATGGCGGAAACAATGTTTACATCGATCAGCTGTCATGGTGATTTATGGC 120
armadillo CTCTGGCAATCATGGCGGAAACAATGTTTACATCGATCAGCTGTCATGGTGATTTATGGC 120
elephant CTCTGGCAATCATGGCGGAAACAATGTTTACATCGATCAGCTGTCATGGTGATTTATGGC 120
 * ****** ** *********************** ****** **********

## Slide 59
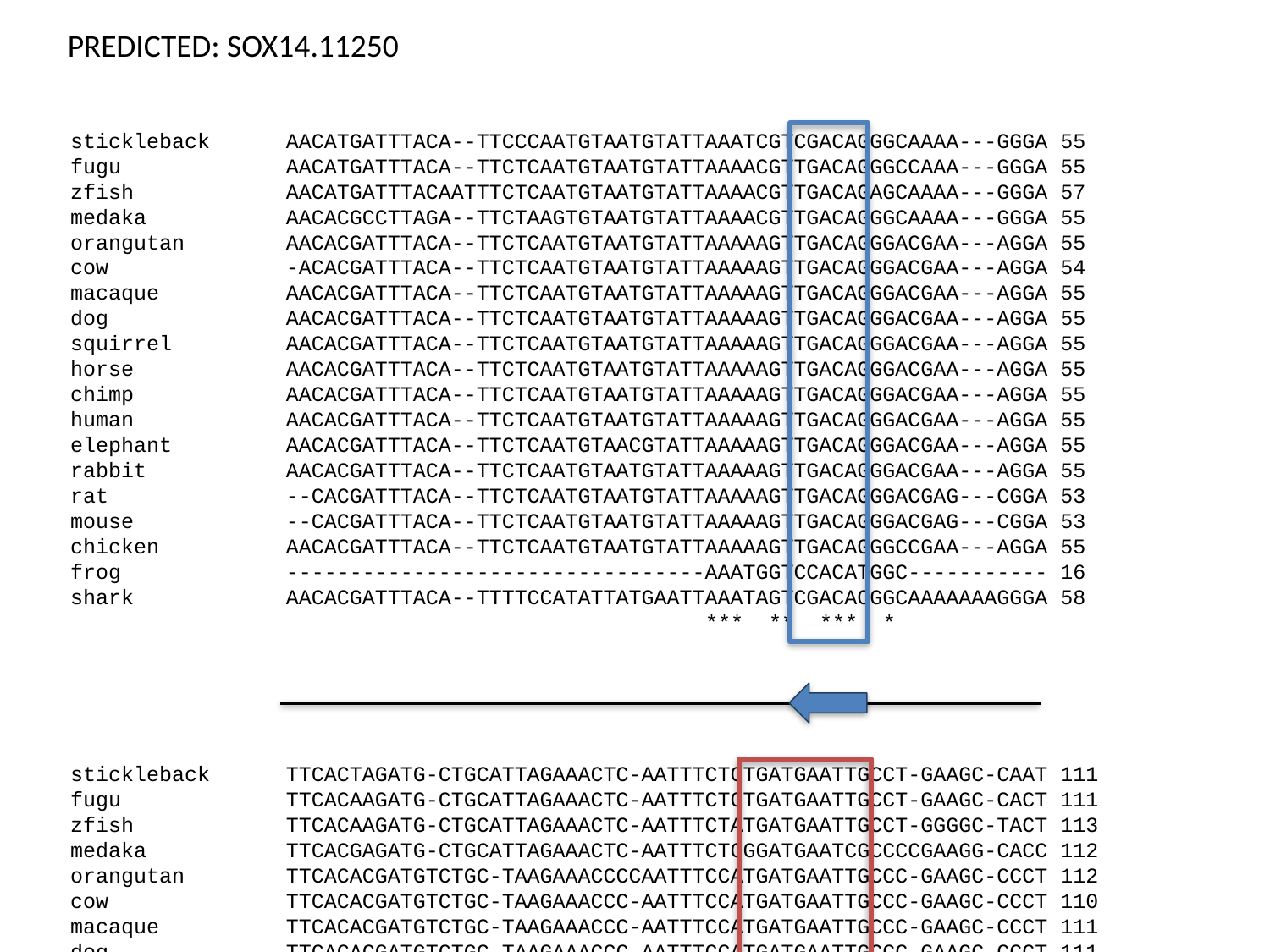

PREDICTED: SOX14.11250
stickleback AACATGATTTACA--TTCCCAATGTAATGTATTAAATCGTCGACAGGGCAAAA---GGGA 55
fugu AACATGATTTACA--TTCTCAATGTAATGTATTAAAACGTTGACAGGGCCAAA---GGGA 55
zfish AACATGATTTACAATTTCTCAATGTAATGTATTAAAACGTTGACAGAGCAAAA---GGGA 57
medaka AACACGCCTTAGA--TTCTAAGTGTAATGTATTAAAACGTTGACAGGGCAAAA---GGGA 55
orangutan AACACGATTTACA--TTCTCAATGTAATGTATTAAAAAGTTGACAGGGACGAA---AGGA 55
cow -ACACGATTTACA--TTCTCAATGTAATGTATTAAAAAGTTGACAGGGACGAA---AGGA 54
macaque AACACGATTTACA--TTCTCAATGTAATGTATTAAAAAGTTGACAGGGACGAA---AGGA 55
dog AACACGATTTACA--TTCTCAATGTAATGTATTAAAAAGTTGACAGGGACGAA---AGGA 55
squirrel AACACGATTTACA--TTCTCAATGTAATGTATTAAAAAGTTGACAGGGACGAA---AGGA 55
horse AACACGATTTACA--TTCTCAATGTAATGTATTAAAAAGTTGACAGGGACGAA---AGGA 55
chimp AACACGATTTACA--TTCTCAATGTAATGTATTAAAAAGTTGACAGGGACGAA---AGGA 55
human AACACGATTTACA--TTCTCAATGTAATGTATTAAAAAGTTGACAGGGACGAA---AGGA 55
elephant AACACGATTTACA--TTCTCAATGTAACGTATTAAAAAGTTGACAGGGACGAA---AGGA 55
rabbit AACACGATTTACA--TTCTCAATGTAATGTATTAAAAAGTTGACAGGGACGAA---AGGA 55
rat --CACGATTTACA--TTCTCAATGTAATGTATTAAAAAGTTGACAGGGACGAG---CGGA 53
mouse --CACGATTTACA--TTCTCAATGTAATGTATTAAAAAGTTGACAGGGACGAG---CGGA 53
chicken AACACGATTTACA--TTCTCAATGTAATGTATTAAAAAGTTGACAGGGCCGAA---AGGA 55
frog ---------------------------------AAATGGTCCACATGGC----------- 16
shark AACACGATTTACA--TTTTCCATATTATGAATTAAATAGTCGACACGGCAAAAAAAGGGA 58
 *** ** *** *
stickleback TTCACTAGATG-CTGCATTAGAAACTC-AATTTCTGTGATGAATTGCCT-GAAGC-CAAT 111
fugu TTCACAAGATG-CTGCATTAGAAACTC-AATTTCTGTGATGAATTGCCT-GAAGC-CACT 111
zfish TTCACAAGATG-CTGCATTAGAAACTC-AATTTCTATGATGAATTGCCT-GGGGC-TACT 113
medaka TTCACGAGATG-CTGCATTAGAAACTC-AATTTCTGGGATGAATCGCCCCGAAGG-CACC 112
orangutan TTCACACGATGTCTGC-TAAGAAACCCCAATTTCCATGATGAATTGCCC-GAAGC-CCCT 112
cow TTCACACGATGTCTGC-TAAGAAACCC-AATTTCCATGATGAATTGCCC-GAAGC-CCCT 110
macaque TTCACACGATGTCTGC-TAAGAAACCC-AATTTCCATGATGAATTGCCC-GAAGC-CCCT 111
dog TTCACACGATGTCTGC-TAAGAAACCC-AATTTCCATGATGAATTGCCC-GAAGC-CCCT 111
squirrel TTCACACGATGTCTGC-TAAGAAACCC-AATTTCCATGATGAATTGCCC-GAAGC-CCCT 111
horse TTCACACGATGTCTGC-TAAGAAACCC-AATTTCCATGATGAATTGCCC-GAAGC-CCCT 111
chimp TTCACACGATGTCTGC-TAAGAAACCC-AATTTCCATGATGAATTGCCC-GAAGC-CCCT 111
human TTCACACGATGTCTGC-TAAGAAACCC-AATTTCCATGATGAATTGCCC-GAAGC-CCCT 111
elephant TTCACACGATGTCTGC-TAAGAAACCC-AATTTCCATGATGAATTGCCC-GAAGC-CCCT 111
rabbit TTCACACGATGTCTGC-TACGAAACCC-AATTTCCATGATGAATTGCCC-GAAGC-CCCT 111
rat TTCACACGATGTCTGC-TAAGAAACCC-AATTTCCATGATGAATTGCCC-GGAGC-CCCT 109
mouse TTCACACGATGTCTGC-TAAGAAACCC-AATTTCCATGATGAATTGCCC-GGAGC-CCCT 109
chicken TTCACATGATGTCGGC-TAAGAAACCC-AATTTCCATGATGAATTGCCC-GGAGC-CCCT 111
frog CTCA------G--CGC---AGAAACCC--ATTTCCATGATGAATTGCCC-AAAGCATCTT 62
shark TTCACTCCATG-TTGCCTGAGAAACCT-AATTTCTATGATGAATCGCCT-GCAGT-CTCT 114
 *** * ** ***** ***** ******* *** *

## Slide 60
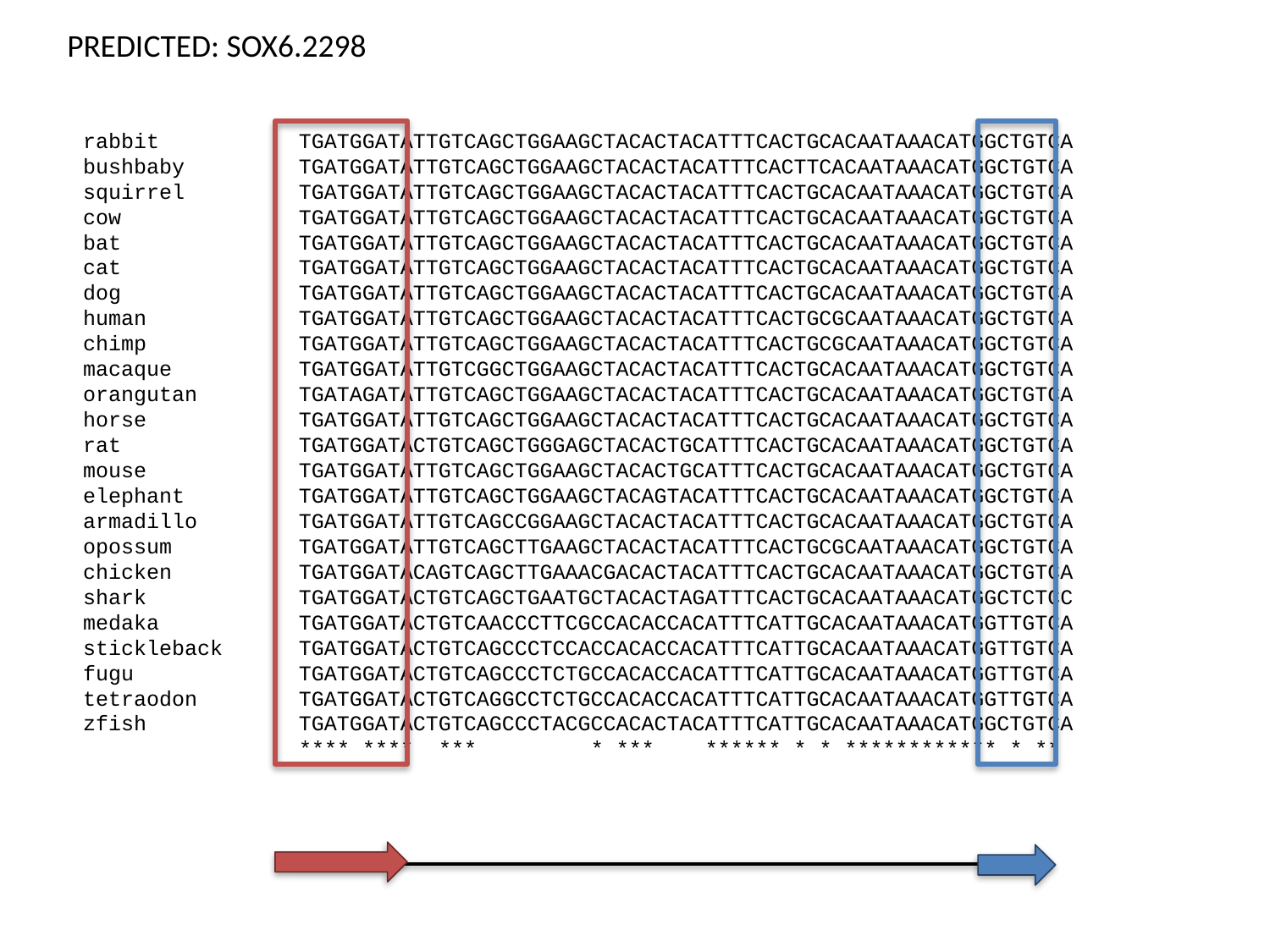

PREDICTED: SOX6.2298
rabbit TGATGGATATTGTCAGCTGGAAGCTACACTACATTTCACTGCACAATAAACATGGCTGTCA
bushbaby TGATGGATATTGTCAGCTGGAAGCTACACTACATTTCACTTCACAATAAACATGGCTGTCA
squirrel TGATGGATATTGTCAGCTGGAAGCTACACTACATTTCACTGCACAATAAACATGGCTGTCA
cow TGATGGATATTGTCAGCTGGAAGCTACACTACATTTCACTGCACAATAAACATGGCTGTCA
bat TGATGGATATTGTCAGCTGGAAGCTACACTACATTTCACTGCACAATAAACATGGCTGTCA
cat TGATGGATATTGTCAGCTGGAAGCTACACTACATTTCACTGCACAATAAACATGGCTGTCA
dog TGATGGATATTGTCAGCTGGAAGCTACACTACATTTCACTGCACAATAAACATGGCTGTCA
human TGATGGATATTGTCAGCTGGAAGCTACACTACATTTCACTGCGCAATAAACATGGCTGTCA
chimp TGATGGATATTGTCAGCTGGAAGCTACACTACATTTCACTGCGCAATAAACATGGCTGTCA
macaque TGATGGATATTGTCGGCTGGAAGCTACACTACATTTCACTGCACAATAAACATGGCTGTCA
orangutan TGATAGATATTGTCAGCTGGAAGCTACACTACATTTCACTGCACAATAAACATGGCTGTCA
horse TGATGGATATTGTCAGCTGGAAGCTACACTACATTTCACTGCACAATAAACATGGCTGTCA
rat TGATGGATACTGTCAGCTGGGAGCTACACTGCATTTCACTGCACAATAAACATGGCTGTCA
mouse TGATGGATATTGTCAGCTGGAAGCTACACTGCATTTCACTGCACAATAAACATGGCTGTCA
elephant TGATGGATATTGTCAGCTGGAAGCTACAGTACATTTCACTGCACAATAAACATGGCTGTCA
armadillo TGATGGATATTGTCAGCCGGAAGCTACACTACATTTCACTGCACAATAAACATGGCTGTCA
opossum TGATGGATATTGTCAGCTTGAAGCTACACTACATTTCACTGCGCAATAAACATGGCTGTCA
chicken TGATGGATACAGTCAGCTTGAAACGACACTACATTTCACTGCACAATAAACATGGCTGTCA
shark TGATGGATACTGTCAGCTGAATGCTACACTAGATTTCACTGCACAATAAACATGGCTCTCC
medaka TGATGGATACTGTCAACCCTTCGCCACACCACATTTCATTGCACAATAAACATGGTTGTCA
stickleback TGATGGATACTGTCAGCCCTCCACCACACCACATTTCATTGCACAATAAACATGGTTGTCA
fugu TGATGGATACTGTCAGCCCTCTGCCACACCACATTTCATTGCACAATAAACATGGTTGTCA
tetraodon TGATGGATACTGTCAGGCCTCTGCCACACCACATTTCATTGCACAATAAACATGGTTGTCA
zfish TGATGGATACTGTCAGCCCTACGCCACACTACATTTCATTGCACAATAAACATGGCTGTCA
 **** **** *** * *** ****** * * ************ * **

## Slide 61
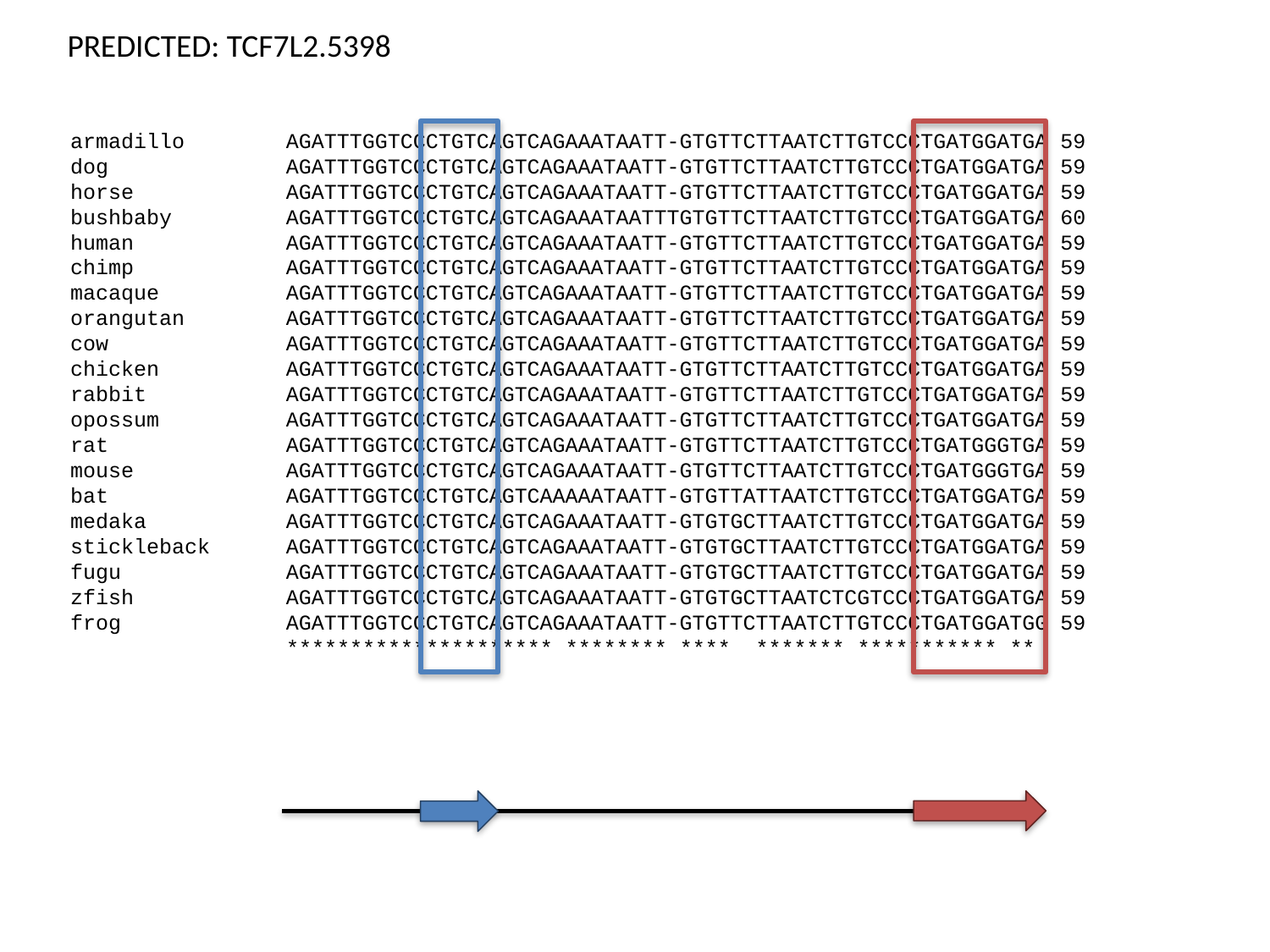

PREDICTED: TCF7L2.5398
armadillo AGATTTGGTCCCTGTCAGTCAGAAATAATT-GTGTTCTTAATCTTGTCCCTGATGGATGA 59
dog AGATTTGGTCCCTGTCAGTCAGAAATAATT-GTGTTCTTAATCTTGTCCCTGATGGATGA 59
horse AGATTTGGTCCCTGTCAGTCAGAAATAATT-GTGTTCTTAATCTTGTCCCTGATGGATGA 59
bushbaby AGATTTGGTCCCTGTCAGTCAGAAATAATTTGTGTTCTTAATCTTGTCCCTGATGGATGA 60
human AGATTTGGTCCCTGTCAGTCAGAAATAATT-GTGTTCTTAATCTTGTCCCTGATGGATGA 59
chimp AGATTTGGTCCCTGTCAGTCAGAAATAATT-GTGTTCTTAATCTTGTCCCTGATGGATGA 59
macaque AGATTTGGTCCCTGTCAGTCAGAAATAATT-GTGTTCTTAATCTTGTCCCTGATGGATGA 59
orangutan AGATTTGGTCCCTGTCAGTCAGAAATAATT-GTGTTCTTAATCTTGTCCCTGATGGATGA 59
cow AGATTTGGTCCCTGTCAGTCAGAAATAATT-GTGTTCTTAATCTTGTCCCTGATGGATGA 59
chicken AGATTTGGTCCCTGTCAGTCAGAAATAATT-GTGTTCTTAATCTTGTCCCTGATGGATGA 59
rabbit AGATTTGGTCCCTGTCAGTCAGAAATAATT-GTGTTCTTAATCTTGTCCCTGATGGATGA 59
opossum AGATTTGGTCCCTGTCAGTCAGAAATAATT-GTGTTCTTAATCTTGTCCCTGATGGATGA 59
rat AGATTTGGTCCCTGTCAGTCAGAAATAATT-GTGTTCTTAATCTTGTCCCTGATGGGTGA 59
mouse AGATTTGGTCCCTGTCAGTCAGAAATAATT-GTGTTCTTAATCTTGTCCCTGATGGGTGA 59
bat AGATTTGGTCCCTGTCAGTCAAAAATAATT-GTGTTATTAATCTTGTCCCTGATGGATGA 59
medaka AGATTTGGTCCCTGTCAGTCAGAAATAATT-GTGTGCTTAATCTTGTCCCTGATGGATGA 59
stickleback AGATTTGGTCCCTGTCAGTCAGAAATAATT-GTGTGCTTAATCTTGTCCCTGATGGATGA 59
fugu AGATTTGGTCCCTGTCAGTCAGAAATAATT-GTGTGCTTAATCTTGTCCCTGATGGATGA 59
zfish AGATTTGGTCCCTGTCAGTCAGAAATAATT-GTGTGCTTAATCTCGTCCCTGATGGATGA 59
frog AGATTTGGTCCCTGTCAGTCAGAAATAATT-GTGTTCTTAATCTTGTCCCTGATGGATGG 59
 ********************* ******** **** ******* *********** **

## Slide 62
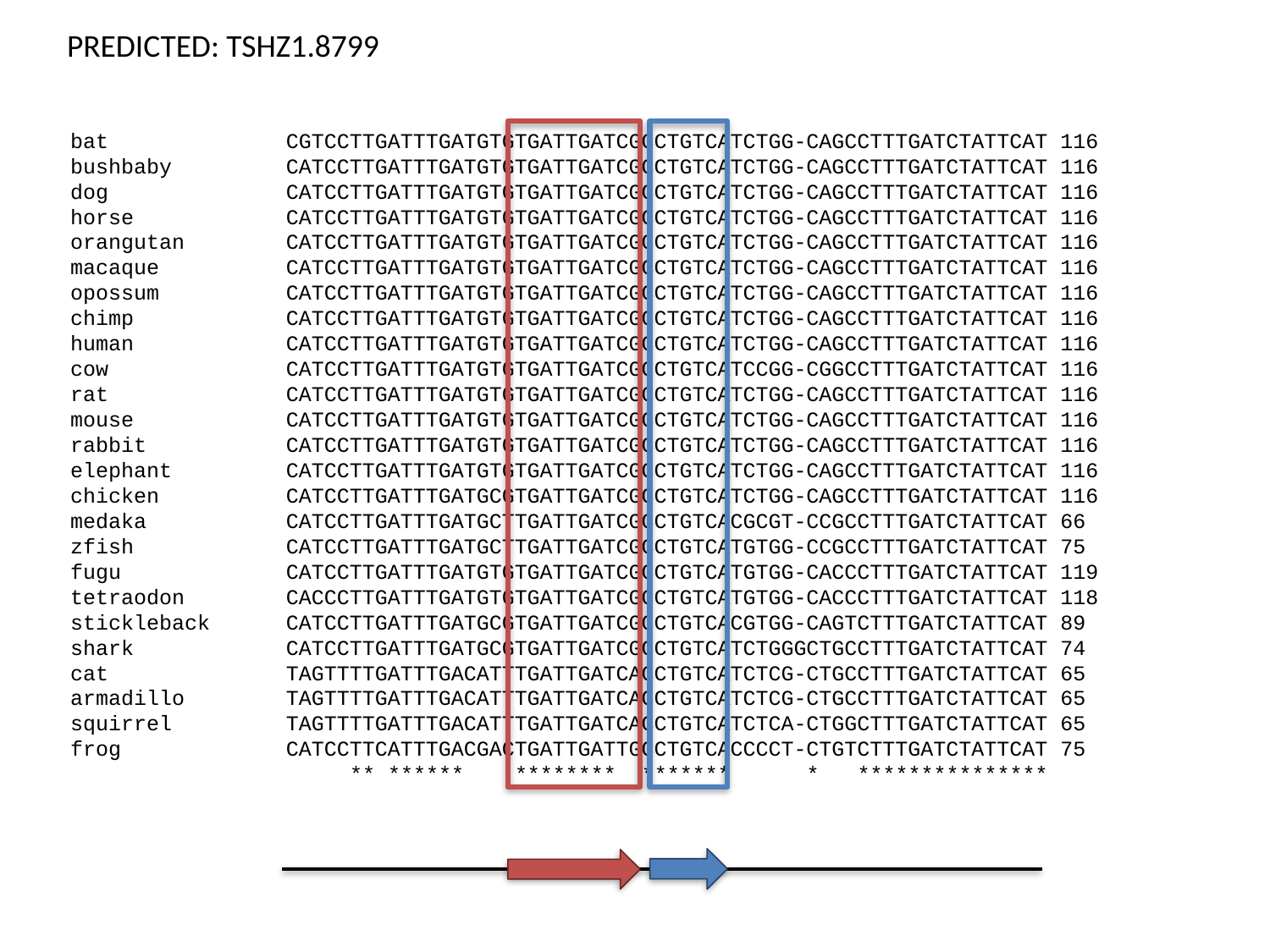

PREDICTED: TSHZ1.8799
bat CGTCCTTGATTTGATGTGTGATTGATCGCCTGTCATCTGG-CAGCCTTTGATCTATTCAT 116
bushbaby CATCCTTGATTTGATGTGTGATTGATCGCCTGTCATCTGG-CAGCCTTTGATCTATTCAT 116
dog CATCCTTGATTTGATGTGTGATTGATCGCCTGTCATCTGG-CAGCCTTTGATCTATTCAT 116
horse CATCCTTGATTTGATGTGTGATTGATCGCCTGTCATCTGG-CAGCCTTTGATCTATTCAT 116
orangutan CATCCTTGATTTGATGTGTGATTGATCGCCTGTCATCTGG-CAGCCTTTGATCTATTCAT 116
macaque CATCCTTGATTTGATGTGTGATTGATCGCCTGTCATCTGG-CAGCCTTTGATCTATTCAT 116
opossum CATCCTTGATTTGATGTGTGATTGATCGCCTGTCATCTGG-CAGCCTTTGATCTATTCAT 116
chimp CATCCTTGATTTGATGTGTGATTGATCGCCTGTCATCTGG-CAGCCTTTGATCTATTCAT 116
human CATCCTTGATTTGATGTGTGATTGATCGCCTGTCATCTGG-CAGCCTTTGATCTATTCAT 116
cow CATCCTTGATTTGATGTGTGATTGATCGCCTGTCATCCGG-CGGCCTTTGATCTATTCAT 116
rat CATCCTTGATTTGATGTGTGATTGATCGCCTGTCATCTGG-CAGCCTTTGATCTATTCAT 116
mouse CATCCTTGATTTGATGTGTGATTGATCGCCTGTCATCTGG-CAGCCTTTGATCTATTCAT 116
rabbit CATCCTTGATTTGATGTGTGATTGATCGCCTGTCATCTGG-CAGCCTTTGATCTATTCAT 116
elephant CATCCTTGATTTGATGTGTGATTGATCGCCTGTCATCTGG-CAGCCTTTGATCTATTCAT 116
chicken CATCCTTGATTTGATGCGTGATTGATCGCCTGTCATCTGG-CAGCCTTTGATCTATTCAT 116
medaka CATCCTTGATTTGATGCTTGATTGATCGCCTGTCACGCGT-CCGCCTTTGATCTATTCAT 66
zfish CATCCTTGATTTGATGCTTGATTGATCGCCTGTCATGTGG-CCGCCTTTGATCTATTCAT 75
fugu CATCCTTGATTTGATGTGTGATTGATCGCCTGTCATGTGG-CACCCTTTGATCTATTCAT 119
tetraodon CACCCTTGATTTGATGTGTGATTGATCGCCTGTCATGTGG-CACCCTTTGATCTATTCAT 118
stickleback CATCCTTGATTTGATGCGTGATTGATCGCCTGTCACGTGG-CAGTCTTTGATCTATTCAT 89
shark CATCCTTGATTTGATGCGTGATTGATCGCCTGTCATCTGGGCTGCCTTTGATCTATTCAT 74
cat TAGTTTTGATTTGACATTTGATTGATCACCTGTCATCTCG-CTGCCTTTGATCTATTCAT 65
armadillo TAGTTTTGATTTGACATTTGATTGATCACCTGTCATCTCG-CTGCCTTTGATCTATTCAT 65
squirrel TAGTTTTGATTTGACATTTGATTGATCACCTGTCATCTCA-CTGGCTTTGATCTATTCAT 65
frog CATCCTTCATTTGACGACTGATTGATTGCCTGTCACCCCT-CTGTCTTTGATCTATTCAT 75
 ** ****** ******** ******* * ***************

## Slide 63
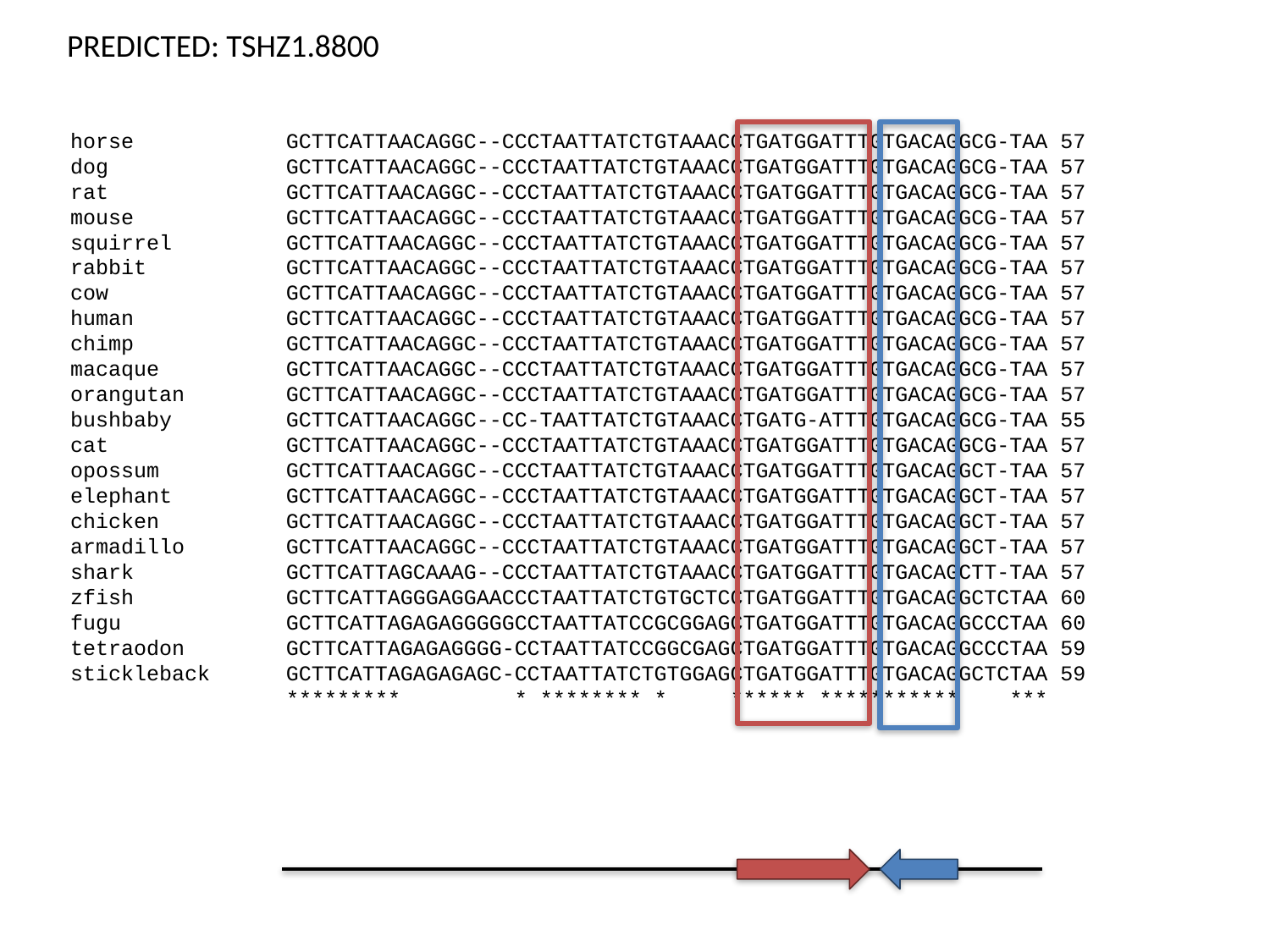

PREDICTED: TSHZ1.8800
horse GCTTCATTAACAGGC--CCCTAATTATCTGTAAACCTGATGGATTTGTGACAGGCG-TAA 57
dog GCTTCATTAACAGGC--CCCTAATTATCTGTAAACCTGATGGATTTGTGACAGGCG-TAA 57
rat GCTTCATTAACAGGC--CCCTAATTATCTGTAAACCTGATGGATTTGTGACAGGCG-TAA 57
mouse GCTTCATTAACAGGC--CCCTAATTATCTGTAAACCTGATGGATTTGTGACAGGCG-TAA 57
squirrel GCTTCATTAACAGGC--CCCTAATTATCTGTAAACCTGATGGATTTGTGACAGGCG-TAA 57
rabbit GCTTCATTAACAGGC--CCCTAATTATCTGTAAACCTGATGGATTTGTGACAGGCG-TAA 57
cow GCTTCATTAACAGGC--CCCTAATTATCTGTAAACCTGATGGATTTGTGACAGGCG-TAA 57
human GCTTCATTAACAGGC--CCCTAATTATCTGTAAACCTGATGGATTTGTGACAGGCG-TAA 57
chimp GCTTCATTAACAGGC--CCCTAATTATCTGTAAACCTGATGGATTTGTGACAGGCG-TAA 57
macaque GCTTCATTAACAGGC--CCCTAATTATCTGTAAACCTGATGGATTTGTGACAGGCG-TAA 57
orangutan GCTTCATTAACAGGC--CCCTAATTATCTGTAAACCTGATGGATTTGTGACAGGCG-TAA 57
bushbaby GCTTCATTAACAGGC--CC-TAATTATCTGTAAACCTGATG-ATTTGTGACAGGCG-TAA 55
cat GCTTCATTAACAGGC--CCCTAATTATCTGTAAACCTGATGGATTTGTGACAGGCG-TAA 57
opossum GCTTCATTAACAGGC--CCCTAATTATCTGTAAACCTGATGGATTTGTGACAGGCT-TAA 57
elephant GCTTCATTAACAGGC--CCCTAATTATCTGTAAACCTGATGGATTTGTGACAGGCT-TAA 57
chicken GCTTCATTAACAGGC--CCCTAATTATCTGTAAACCTGATGGATTTGTGACAGGCT-TAA 57
armadillo GCTTCATTAACAGGC--CCCTAATTATCTGTAAACCTGATGGATTTGTGACAGGCT-TAA 57
shark GCTTCATTAGCAAAG--CCCTAATTATCTGTAAACCTGATGGATTTGTGACAGCTT-TAA 57
zfish GCTTCATTAGGGAGGAACCCTAATTATCTGTGCTCCTGATGGATTTGTGACAGGCTCTAA 60
fugu GCTTCATTAGAGAGGGGGCCTAATTATCCGCGGAGCTGATGGATTTGTGACAGGCCCTAA 60
tetraodon GCTTCATTAGAGAGGGG-CCTAATTATCCGGCGAGCTGATGGATTTGTGACAGGCCCTAA 59
stickleback GCTTCATTAGAGAGAGC-CCTAATTATCTGTGGAGCTGATGGATTTGTGACAGGCTCTAA 59
 ********* * ******** * ****** *********** ***

## Slide 64
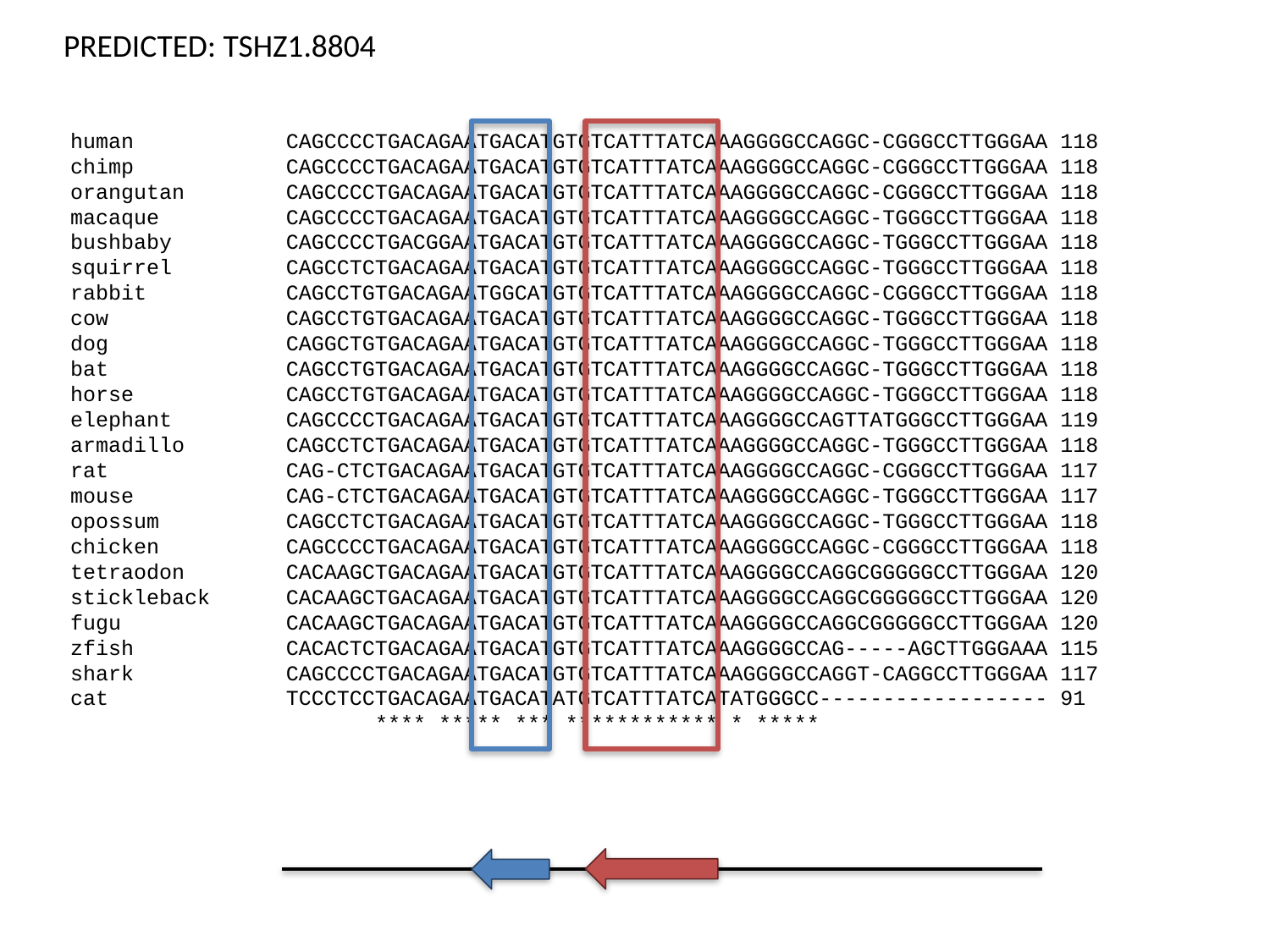

PREDICTED: TSHZ1.8804
human CAGCCCCTGACAGAATGACATGTGTCATTTATCAAAGGGGCCAGGC-CGGGCCTTGGGAA 118
chimp CAGCCCCTGACAGAATGACATGTGTCATTTATCAAAGGGGCCAGGC-CGGGCCTTGGGAA 118
orangutan CAGCCCCTGACAGAATGACATGTGTCATTTATCAAAGGGGCCAGGC-CGGGCCTTGGGAA 118
macaque CAGCCCCTGACAGAATGACATGTGTCATTTATCAAAGGGGCCAGGC-TGGGCCTTGGGAA 118
bushbaby CAGCCCCTGACGGAATGACATGTGTCATTTATCAAAGGGGCCAGGC-TGGGCCTTGGGAA 118
squirrel CAGCCTCTGACAGAATGACATGTGTCATTTATCAAAGGGGCCAGGC-TGGGCCTTGGGAA 118
rabbit CAGCCTGTGACAGAATGGCATGTGTCATTTATCAAAGGGGCCAGGC-CGGGCCTTGGGAA 118
cow CAGCCTGTGACAGAATGACATGTGTCATTTATCAAAGGGGCCAGGC-TGGGCCTTGGGAA 118
dog CAGGCTGTGACAGAATGACATGTGTCATTTATCAAAGGGGCCAGGC-TGGGCCTTGGGAA 118
bat CAGCCTGTGACAGAATGACATGTGTCATTTATCAAAGGGGCCAGGC-TGGGCCTTGGGAA 118
horse CAGCCTGTGACAGAATGACATGTGTCATTTATCAAAGGGGCCAGGC-TGGGCCTTGGGAA 118
elephant CAGCCCCTGACAGAATGACATGTGTCATTTATCAAAGGGGCCAGTTATGGGCCTTGGGAA 119
armadillo CAGCCTCTGACAGAATGACATGTGTCATTTATCAAAGGGGCCAGGC-TGGGCCTTGGGAA 118
rat CAG-CTCTGACAGAATGACATGTGTCATTTATCAAAGGGGCCAGGC-CGGGCCTTGGGAA 117
mouse CAG-CTCTGACAGAATGACATGTGTCATTTATCAAAGGGGCCAGGC-TGGGCCTTGGGAA 117
opossum CAGCCTCTGACAGAATGACATGTGTCATTTATCAAAGGGGCCAGGC-TGGGCCTTGGGAA 118
chicken CAGCCCCTGACAGAATGACATGTGTCATTTATCAAAGGGGCCAGGC-CGGGCCTTGGGAA 118
tetraodon CACAAGCTGACAGAATGACATGTGTCATTTATCAAAGGGGCCAGGCGGGGGCCTTGGGAA 120
stickleback CACAAGCTGACAGAATGACATGTGTCATTTATCAAAGGGGCCAGGCGGGGGCCTTGGGAA 120
fugu CACAAGCTGACAGAATGACATGTGTCATTTATCAAAGGGGCCAGGCGGGGGCCTTGGGAA 120
zfish CACACTCTGACAGAATGACATGTGTCATTTATCAAAGGGGCCAG-----AGCTTGGGAAA 115
shark CAGCCCCTGACAGAATGACATGTGTCATTTATCAAAGGGGCCAGGT-CAGGCCTTGGGAA 117
cat TCCCTCCTGACAGAATGACATATGTCATTTATCATATGGGCC------------------ 91
 **** ***** *** ************ * *****

## Slide 65
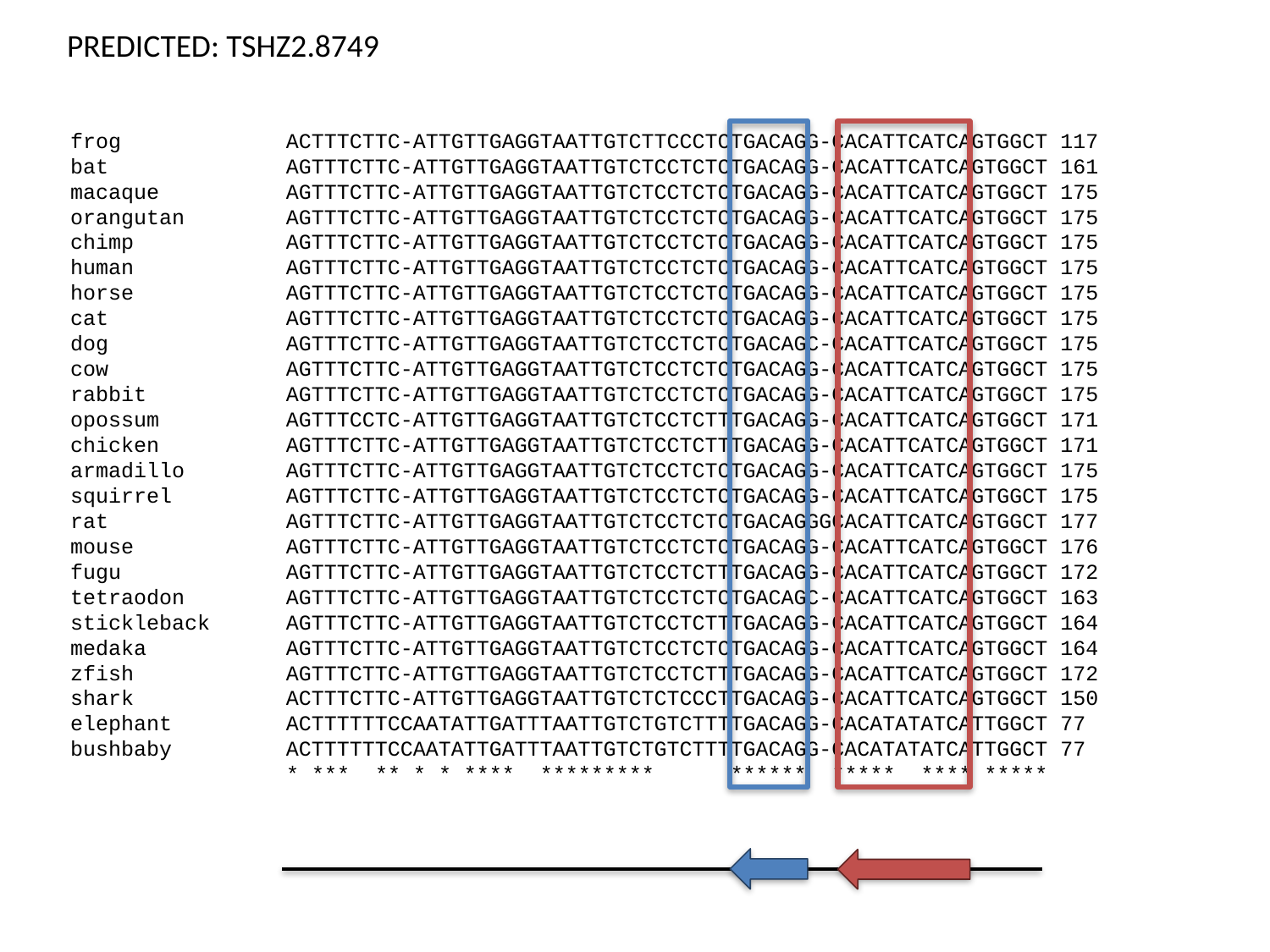

PREDICTED: TSHZ2.8749
frog ACTTTCTTC-ATTGTTGAGGTAATTGTCTTCCCTCTGACAGG-CACATTCATCAGTGGCT 117
bat AGTTTCTTC-ATTGTTGAGGTAATTGTCTCCTCTCTGACAGG-CACATTCATCAGTGGCT 161
macaque AGTTTCTTC-ATTGTTGAGGTAATTGTCTCCTCTCTGACAGG-CACATTCATCAGTGGCT 175
orangutan AGTTTCTTC-ATTGTTGAGGTAATTGTCTCCTCTCTGACAGG-CACATTCATCAGTGGCT 175
chimp AGTTTCTTC-ATTGTTGAGGTAATTGTCTCCTCTCTGACAGG-CACATTCATCAGTGGCT 175
human AGTTTCTTC-ATTGTTGAGGTAATTGTCTCCTCTCTGACAGG-CACATTCATCAGTGGCT 175
horse AGTTTCTTC-ATTGTTGAGGTAATTGTCTCCTCTCTGACAGG-CACATTCATCAGTGGCT 175
cat AGTTTCTTC-ATTGTTGAGGTAATTGTCTCCTCTCTGACAGG-CACATTCATCAGTGGCT 175
dog AGTTTCTTC-ATTGTTGAGGTAATTGTCTCCTCTCTGACAGC-CACATTCATCAGTGGCT 175
cow AGTTTCTTC-ATTGTTGAGGTAATTGTCTCCTCTCTGACAGG-CACATTCATCAGTGGCT 175
rabbit AGTTTCTTC-ATTGTTGAGGTAATTGTCTCCTCTCTGACAGG-CACATTCATCAGTGGCT 175
opossum AGTTTCCTC-ATTGTTGAGGTAATTGTCTCCTCTTTGACAGG-CACATTCATCAGTGGCT 171
chicken AGTTTCTTC-ATTGTTGAGGTAATTGTCTCCTCTTTGACAGG-CACATTCATCAGTGGCT 171
armadillo AGTTTCTTC-ATTGTTGAGGTAATTGTCTCCTCTCTGACAGG-CACATTCATCAGTGGCT 175
squirrel AGTTTCTTC-ATTGTTGAGGTAATTGTCTCCTCTCTGACAGG-CACATTCATCAGTGGCT 175
rat AGTTTCTTC-ATTGTTGAGGTAATTGTCTCCTCTCTGACAGGGCACATTCATCAGTGGCT 177
mouse AGTTTCTTC-ATTGTTGAGGTAATTGTCTCCTCTCTGACAGG-CACATTCATCAGTGGCT 176
fugu AGTTTCTTC-ATTGTTGAGGTAATTGTCTCCTCTTTGACAGG-CACATTCATCAGTGGCT 172
tetraodon AGTTTCTTC-ATTGTTGAGGTAATTGTCTCCTCTCTGACAGC-CACATTCATCAGTGGCT 163
stickleback AGTTTCTTC-ATTGTTGAGGTAATTGTCTCCTCTTTGACAGG-CACATTCATCAGTGGCT 164
medaka AGTTTCTTC-ATTGTTGAGGTAATTGTCTCCTCTCTGACAGG-CACATTCATCAGTGGCT 164
zfish AGTTTCTTC-ATTGTTGAGGTAATTGTCTCCTCTTTGACAGG-CACATTCATCAGTGGCT 172
shark ACTTTCTTC-ATTGTTGAGGTAATTGTCTCTCCCTTGACAGG-CACATTCATCAGTGGCT 150
elephant ACTTTTTTCCAATATTGATTTAATTGTCTGTCTTTTGACAGG-CACATATATCATTGGCT 77
bushbaby ACTTTTTTCCAATATTGATTTAATTGTCTGTCTTTTGACAGG-CACATATATCATTGGCT 77
 * *** ** * * **** ********* ****** ***** **** *****

## Slide 66
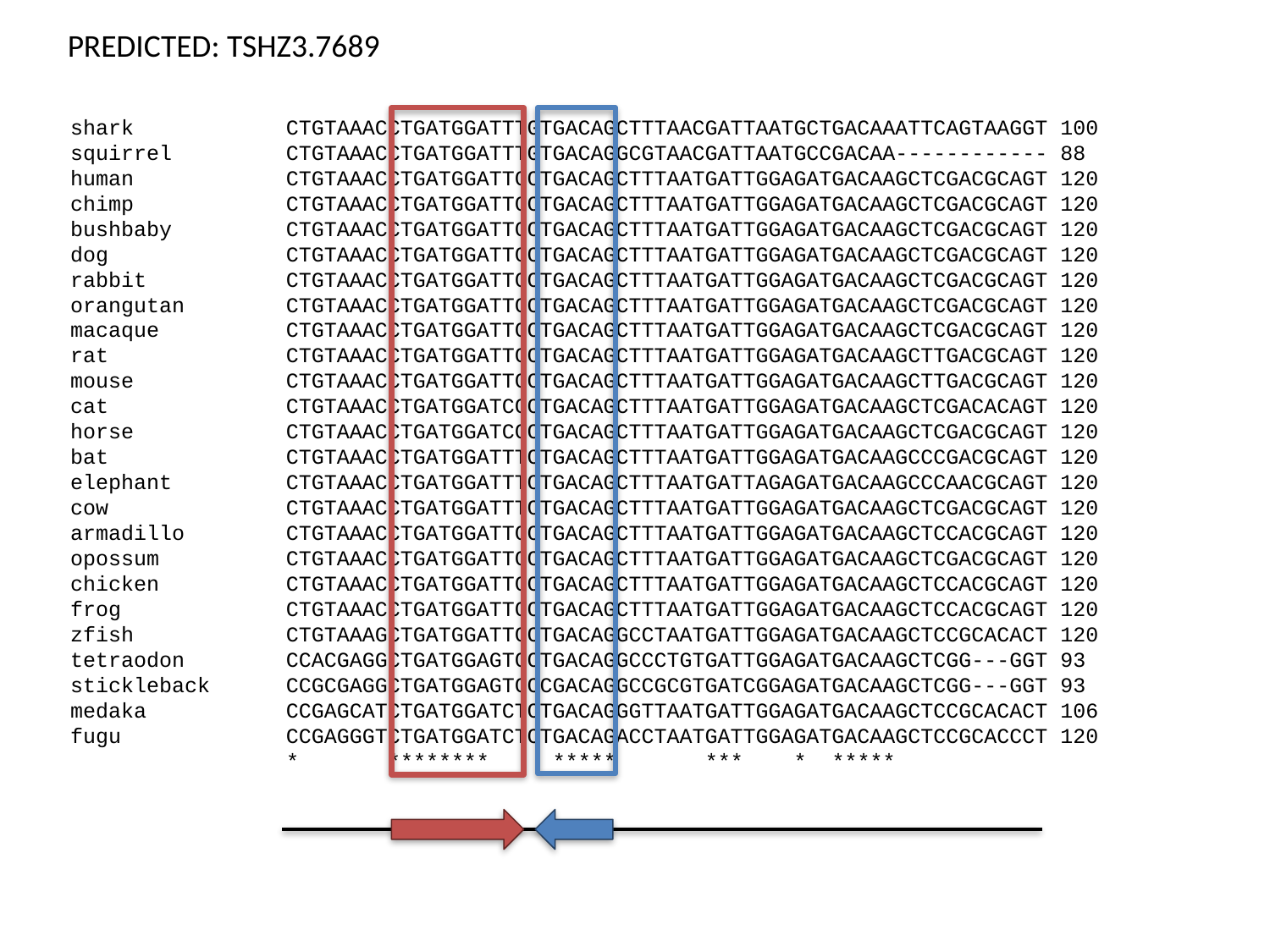

PREDICTED: TSHZ3.7689
shark CTGTAAACCTGATGGATTTGTGACAGCTTTAACGATTAATGCTGACAAATTCAGTAAGGT 100
squirrel CTGTAAACCTGATGGATTTGTGACAGGCGTAACGATTAATGCCGACAA------------ 88
human CTGTAAACCTGATGGATTCCTGACAGCTTTAATGATTGGAGATGACAAGCTCGACGCAGT 120
chimp CTGTAAACCTGATGGATTCCTGACAGCTTTAATGATTGGAGATGACAAGCTCGACGCAGT 120
bushbaby CTGTAAACCTGATGGATTCCTGACAGCTTTAATGATTGGAGATGACAAGCTCGACGCAGT 120
dog CTGTAAACCTGATGGATTCCTGACAGCTTTAATGATTGGAGATGACAAGCTCGACGCAGT 120
rabbit CTGTAAACCTGATGGATTCCTGACAGCTTTAATGATTGGAGATGACAAGCTCGACGCAGT 120
orangutan CTGTAAACCTGATGGATTCCTGACAGCTTTAATGATTGGAGATGACAAGCTCGACGCAGT 120
macaque CTGTAAACCTGATGGATTCCTGACAGCTTTAATGATTGGAGATGACAAGCTCGACGCAGT 120
rat CTGTAAACCTGATGGATTCCTGACAGCTTTAATGATTGGAGATGACAAGCTTGACGCAGT 120
mouse CTGTAAACCTGATGGATTCCTGACAGCTTTAATGATTGGAGATGACAAGCTTGACGCAGT 120
cat CTGTAAACCTGATGGATCCCTGACAGCTTTAATGATTGGAGATGACAAGCTCGACACAGT 120
horse CTGTAAACCTGATGGATCCCTGACAGCTTTAATGATTGGAGATGACAAGCTCGACGCAGT 120
bat CTGTAAACCTGATGGATTTCTGACAGCTTTAATGATTGGAGATGACAAGCCCGACGCAGT 120
elephant CTGTAAACCTGATGGATTTCTGACAGCTTTAATGATTAGAGATGACAAGCCCAACGCAGT 120
cow CTGTAAACCTGATGGATTTCTGACAGCTTTAATGATTGGAGATGACAAGCTCGACGCAGT 120
armadillo CTGTAAACCTGATGGATTCCTGACAGCTTTAATGATTGGAGATGACAAGCTCCACGCAGT 120
opossum CTGTAAACCTGATGGATTCCTGACAGCTTTAATGATTGGAGATGACAAGCTCGACGCAGT 120
chicken CTGTAAACCTGATGGATTCCTGACAGCTTTAATGATTGGAGATGACAAGCTCCACGCAGT 120
frog CTGTAAACCTGATGGATTCCTGACAGCTTTAATGATTGGAGATGACAAGCTCCACGCAGT 120
zfish CTGTAAAGCTGATGGATTCCTGACAGGCCTAATGATTGGAGATGACAAGCTCCGCACACT 120
tetraodon CCACGAGGCTGATGGAGTCCTGACAGGCCCTGTGATTGGAGATGACAAGCTCGG---GGT 93
stickleback CCGCGAGGCTGATGGAGTCCCGACAGGCCGCGTGATCGGAGATGACAAGCTCGG---GGT 93
medaka CCGAGCATCTGATGGATCTCTGACAGGGTTAATGATTGGAGATGACAAGCTCCGCACACT 106
fugu CCGAGGGTCTGATGGATCTCTGACAGACCTAATGATTGGAGATGACAAGCTCCGCACCCT 120
 * ******** ***** *** * *****

## Slide 67
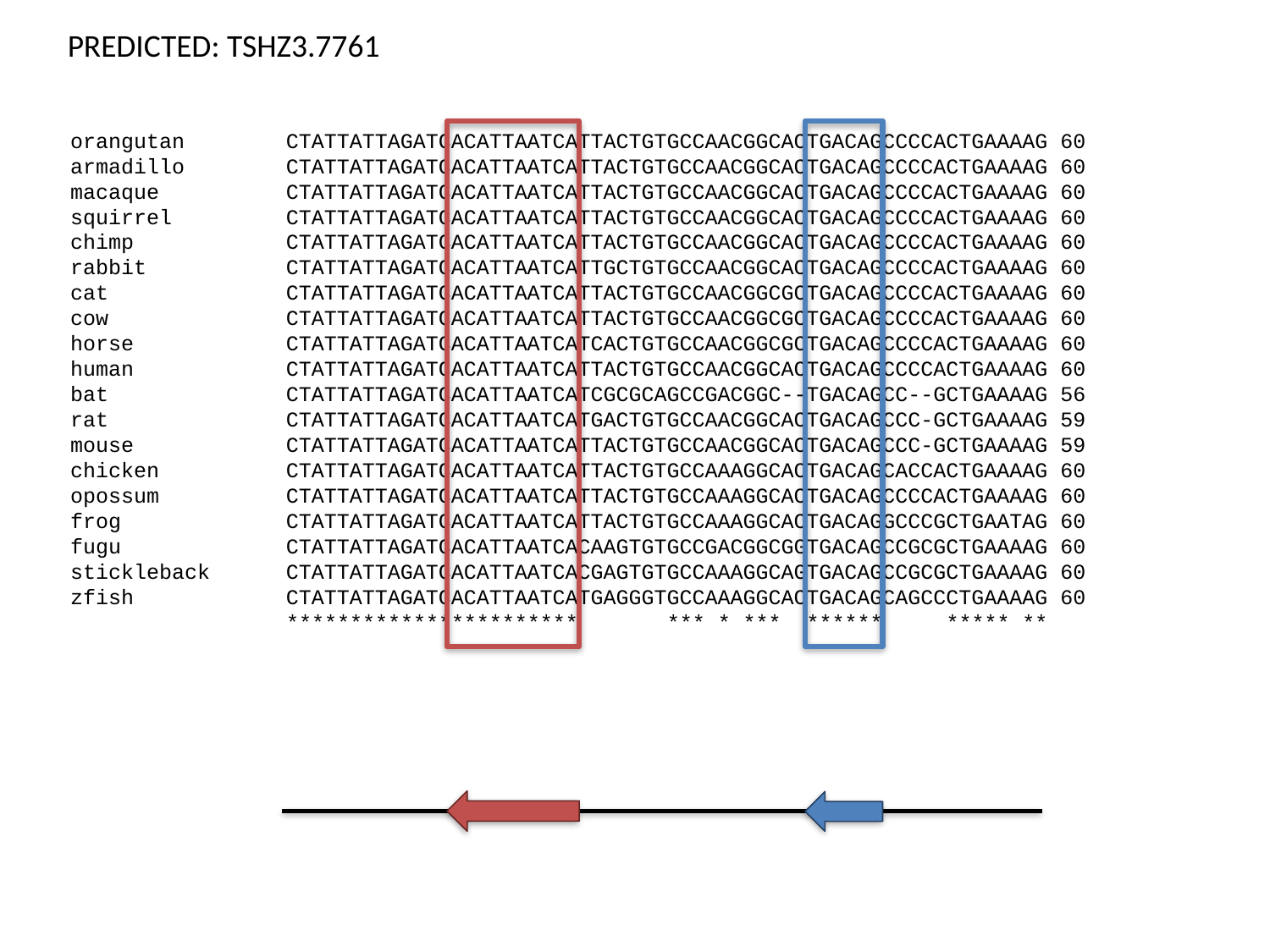

PREDICTED: TSHZ3.7761
orangutan CTATTATTAGATCACATTAATCATTACTGTGCCAACGGCACTGACAGCCCCACTGAAAAG 60
armadillo CTATTATTAGATCACATTAATCATTACTGTGCCAACGGCACTGACAGCCCCACTGAAAAG 60
macaque CTATTATTAGATCACATTAATCATTACTGTGCCAACGGCACTGACAGCCCCACTGAAAAG 60
squirrel CTATTATTAGATCACATTAATCATTACTGTGCCAACGGCACTGACAGCCCCACTGAAAAG 60
chimp CTATTATTAGATCACATTAATCATTACTGTGCCAACGGCACTGACAGCCCCACTGAAAAG 60
rabbit CTATTATTAGATCACATTAATCATTGCTGTGCCAACGGCACTGACAGCCCCACTGAAAAG 60
cat CTATTATTAGATCACATTAATCATTACTGTGCCAACGGCGCTGACAGCCCCACTGAAAAG 60
cow CTATTATTAGATCACATTAATCATTACTGTGCCAACGGCGCTGACAGCCCCACTGAAAAG 60
horse CTATTATTAGATCACATTAATCATCACTGTGCCAACGGCGCTGACAGCCCCACTGAAAAG 60
human CTATTATTAGATCACATTAATCATTACTGTGCCAACGGCACTGACAGCCCCACTGAAAAG 60
bat CTATTATTAGATCACATTAATCATCGCGCAGCCGACGGC--TGACAGCC--GCTGAAAAG 56
rat CTATTATTAGATCACATTAATCATGACTGTGCCAACGGCACTGACAGCCC-GCTGAAAAG 59
mouse CTATTATTAGATCACATTAATCATTACTGTGCCAACGGCACTGACAGCCC-GCTGAAAAG 59
chicken CTATTATTAGATCACATTAATCATTACTGTGCCAAAGGCACTGACAGCACCACTGAAAAG 60
opossum CTATTATTAGATCACATTAATCATTACTGTGCCAAAGGCACTGACAGCCCCACTGAAAAG 60
frog CTATTATTAGATCACATTAATCATTACTGTGCCAAAGGCACTGACAGGCCCGCTGAATAG 60
fugu CTATTATTAGATCACATTAATCACAAGTGTGCCGACGGCGGTGACAGCCGCGCTGAAAAG 60
stickleback CTATTATTAGATCACATTAATCACGAGTGTGCCAAAGGCAGTGACAGCCGCGCTGAAAAG 60
zfish CTATTATTAGATCACATTAATCATGAGGGTGCCAAAGGCACTGACAGCAGCCCTGAAAAG 60
 *********************** *** * *** ****** ***** **

## Slide 68
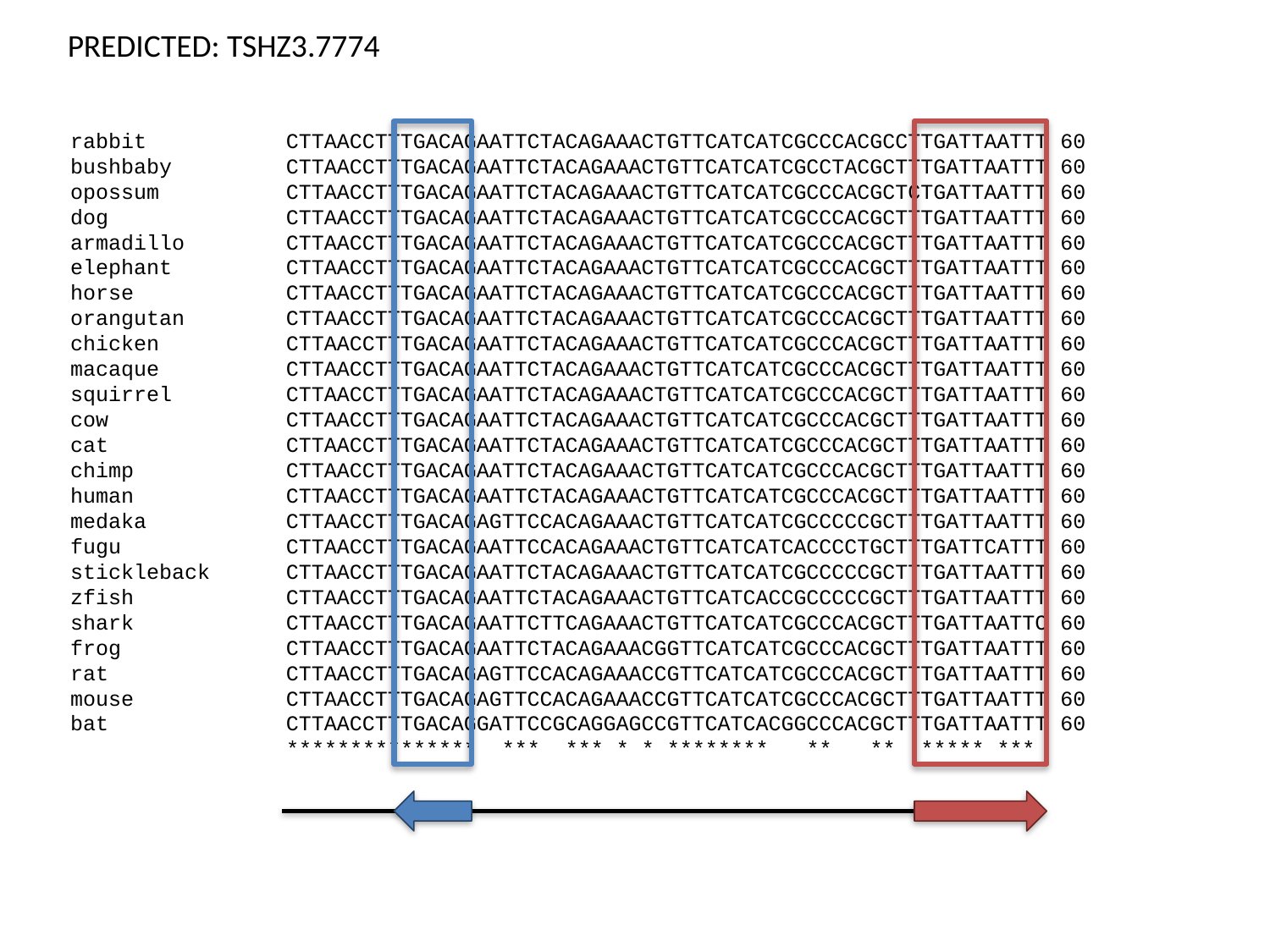

PREDICTED: TSHZ3.7774
rabbit CTTAACCTTTGACAGAATTCTACAGAAACTGTTCATCATCGCCCACGCCTTGATTAATTT 60
bushbaby CTTAACCTTTGACAGAATTCTACAGAAACTGTTCATCATCGCCTACGCTTTGATTAATTT 60
opossum CTTAACCTTTGACAGAATTCTACAGAAACTGTTCATCATCGCCCACGCTCTGATTAATTT 60
dog CTTAACCTTTGACAGAATTCTACAGAAACTGTTCATCATCGCCCACGCTTTGATTAATTT 60
armadillo CTTAACCTTTGACAGAATTCTACAGAAACTGTTCATCATCGCCCACGCTTTGATTAATTT 60
elephant CTTAACCTTTGACAGAATTCTACAGAAACTGTTCATCATCGCCCACGCTTTGATTAATTT 60
horse CTTAACCTTTGACAGAATTCTACAGAAACTGTTCATCATCGCCCACGCTTTGATTAATTT 60
orangutan CTTAACCTTTGACAGAATTCTACAGAAACTGTTCATCATCGCCCACGCTTTGATTAATTT 60
chicken CTTAACCTTTGACAGAATTCTACAGAAACTGTTCATCATCGCCCACGCTTTGATTAATTT 60
macaque CTTAACCTTTGACAGAATTCTACAGAAACTGTTCATCATCGCCCACGCTTTGATTAATTT 60
squirrel CTTAACCTTTGACAGAATTCTACAGAAACTGTTCATCATCGCCCACGCTTTGATTAATTT 60
cow CTTAACCTTTGACAGAATTCTACAGAAACTGTTCATCATCGCCCACGCTTTGATTAATTT 60
cat CTTAACCTTTGACAGAATTCTACAGAAACTGTTCATCATCGCCCACGCTTTGATTAATTT 60
chimp CTTAACCTTTGACAGAATTCTACAGAAACTGTTCATCATCGCCCACGCTTTGATTAATTT 60
human CTTAACCTTTGACAGAATTCTACAGAAACTGTTCATCATCGCCCACGCTTTGATTAATTT 60
medaka CTTAACCTTTGACAGAGTTCCACAGAAACTGTTCATCATCGCCCCCGCTTTGATTAATTT 60
fugu CTTAACCTTTGACAGAATTCCACAGAAACTGTTCATCATCACCCCTGCTTTGATTCATTT 60
stickleback CTTAACCTTTGACAGAATTCTACAGAAACTGTTCATCATCGCCCCCGCTTTGATTAATTT 60
zfish CTTAACCTTTGACAGAATTCTACAGAAACTGTTCATCACCGCCCCCGCTTTGATTAATTT 60
shark CTTAACCTTTGACAGAATTCTTCAGAAACTGTTCATCATCGCCCACGCTTTGATTAATTC 60
frog CTTAACCTTTGACAGAATTCTACAGAAACGGTTCATCATCGCCCACGCTTTGATTAATTT 60
rat CTTAACCTTTGACAGAGTTCCACAGAAACCGTTCATCATCGCCCACGCTTTGATTAATTT 60
mouse CTTAACCTTTGACAGAGTTCCACAGAAACCGTTCATCATCGCCCACGCTTTGATTAATTT 60
bat CTTAACCTTTGACAGGATTCCGCAGGAGCCGTTCATCACGGCCCACGCTTTGATTAATTT 60
 *************** *** *** * * ******** ** ** ***** ***

## Slide 69
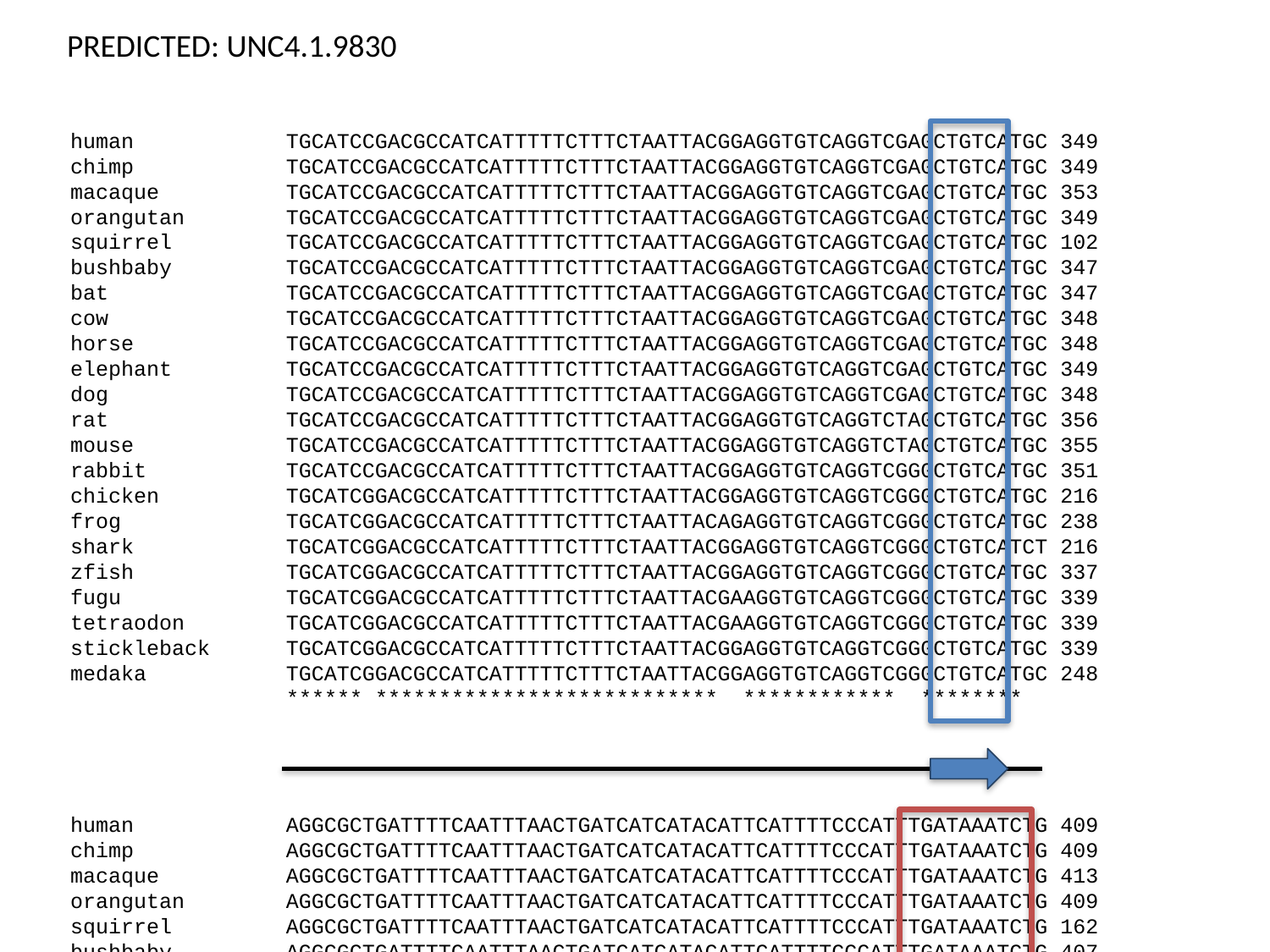

PREDICTED: UNC4.1.9830
human TGCATCCGACGCCATCATTTTTCTTTCTAATTACGGAGGTGTCAGGTCGAGCTGTCATGC 349
chimp TGCATCCGACGCCATCATTTTTCTTTCTAATTACGGAGGTGTCAGGTCGAGCTGTCATGC 349
macaque TGCATCCGACGCCATCATTTTTCTTTCTAATTACGGAGGTGTCAGGTCGAGCTGTCATGC 353
orangutan TGCATCCGACGCCATCATTTTTCTTTCTAATTACGGAGGTGTCAGGTCGAGCTGTCATGC 349
squirrel TGCATCCGACGCCATCATTTTTCTTTCTAATTACGGAGGTGTCAGGTCGAGCTGTCATGC 102
bushbaby TGCATCCGACGCCATCATTTTTCTTTCTAATTACGGAGGTGTCAGGTCGAGCTGTCATGC 347
bat TGCATCCGACGCCATCATTTTTCTTTCTAATTACGGAGGTGTCAGGTCGAGCTGTCATGC 347
cow TGCATCCGACGCCATCATTTTTCTTTCTAATTACGGAGGTGTCAGGTCGAGCTGTCATGC 348
horse TGCATCCGACGCCATCATTTTTCTTTCTAATTACGGAGGTGTCAGGTCGAGCTGTCATGC 348
elephant TGCATCCGACGCCATCATTTTTCTTTCTAATTACGGAGGTGTCAGGTCGAGCTGTCATGC 349
dog TGCATCCGACGCCATCATTTTTCTTTCTAATTACGGAGGTGTCAGGTCGAGCTGTCATGC 348
rat TGCATCCGACGCCATCATTTTTCTTTCTAATTACGGAGGTGTCAGGTCTAGCTGTCATGC 356
mouse TGCATCCGACGCCATCATTTTTCTTTCTAATTACGGAGGTGTCAGGTCTAGCTGTCATGC 355
rabbit TGCATCCGACGCCATCATTTTTCTTTCTAATTACGGAGGTGTCAGGTCGGGCTGTCATGC 351
chicken TGCATCGGACGCCATCATTTTTCTTTCTAATTACGGAGGTGTCAGGTCGGGCTGTCATGC 216
frog TGCATCGGACGCCATCATTTTTCTTTCTAATTACAGAGGTGTCAGGTCGGGCTGTCATGC 238
shark TGCATCGGACGCCATCATTTTTCTTTCTAATTACGGAGGTGTCAGGTCGGGCTGTCATCT 216
zfish TGCATCGGACGCCATCATTTTTCTTTCTAATTACGGAGGTGTCAGGTCGGGCTGTCATGC 337
fugu TGCATCGGACGCCATCATTTTTCTTTCTAATTACGAAGGTGTCAGGTCGGGCTGTCATGC 339
tetraodon TGCATCGGACGCCATCATTTTTCTTTCTAATTACGAAGGTGTCAGGTCGGGCTGTCATGC 339
stickleback TGCATCGGACGCCATCATTTTTCTTTCTAATTACGGAGGTGTCAGGTCGGGCTGTCATGC 339
medaka TGCATCGGACGCCATCATTTTTCTTTCTAATTACGGAGGTGTCAGGTCGGGCTGTCATGC 248
 ****** *************************** ************ ********
human AGGCGCTGATTTTCAATTTAACTGATCATCATACATTCATTTTCCCATTTGATAAATCTG 409
chimp AGGCGCTGATTTTCAATTTAACTGATCATCATACATTCATTTTCCCATTTGATAAATCTG 409
macaque AGGCGCTGATTTTCAATTTAACTGATCATCATACATTCATTTTCCCATTTGATAAATCTG 413
orangutan AGGCGCTGATTTTCAATTTAACTGATCATCATACATTCATTTTCCCATTTGATAAATCTG 409
squirrel AGGCGCTGATTTTCAATTTAACTGATCATCATACATTCATTTTCCCATTTGATAAATCTG 162
bushbaby AGGCGCTGATTTTCAATTTAACTGATCATCATACATTCATTTTCCCATTTGATAAATCTG 407
bat AGGCGCTGATTTTCAATTTAACTGATCATCATACATTCATTTTCCCATTTGATAAATCTG 407
cow AGGCGCTGATTTTCAATTTAACTGATCATCATACATTCATTTTCCCATTTGATAAATCTG 408
horse AGGCGCTGATTTTCAATTTAACTGATCATCATACATTCATTTTCCCATTTGATAAATCTG 408
elephant AGGCGCTGATTTTCAATTTAACTGATCATCATACATTCATTTTCCCATTTGATAAATCTG 409
dog AGGCGCTGATTTTCAATTTAACTGATCATCATACATTCATTTTCCCATTTGATAAATCTG 408
rat AGGCGCTGATTTTCAATTTAACTGATCATCATACATTCATTTTCCCATTTGATAAATCTG 416
mouse AGGCGCTGATTTTCAATTTAACTGATCATCATACATTCATTTTCCCATTTGATAAATCTG 415
rabbit AGGCGCTGATTTTCAATTTAACTGATCATCATACATTCATTTTCCCATTTGATAAATCTG 411
chicken AGGCGCTGATTTTCAATTTAACTGATCATCATACATTCATTATACCATTTGATAAATCTG 276
frog AGGCGCTGATTTTCAATTTAACTGATCATCATACATTCATTTTCCCATTTGATAAATCTG 298
shark CGGCGCTGATTTTCAATTTAACTGATCATCATACATTCATTTCCCCATTTGATAAATCTG 276
zfish AGGCGCTGATTTTCAATTTAACTGATCATCATGCATTCATTTTCCCATTTGATAAATCTG 397
fugu AGACGCTGATTTTCAATTTAACTGATCATCACGCATTCATTTTCCCATTTGATAAATCTG 399
tetraodon AGACGCTGATTTTCAATTTAACTGATCATCACGCATTCATTTTCCCATTTGATAAATCTG 399
stickleback AGACGCTGATTTTCAATTTAACTGATCATCATGCATTCATTTTCCCATTTGATAAATCTG 399
medaka AGACGCTGATTTTCAATTTAACTGATCATCGTGCATTCATTTCCCCATTTGATAAATCTG 308
 * *************************** ******** ****************

## Slide 70
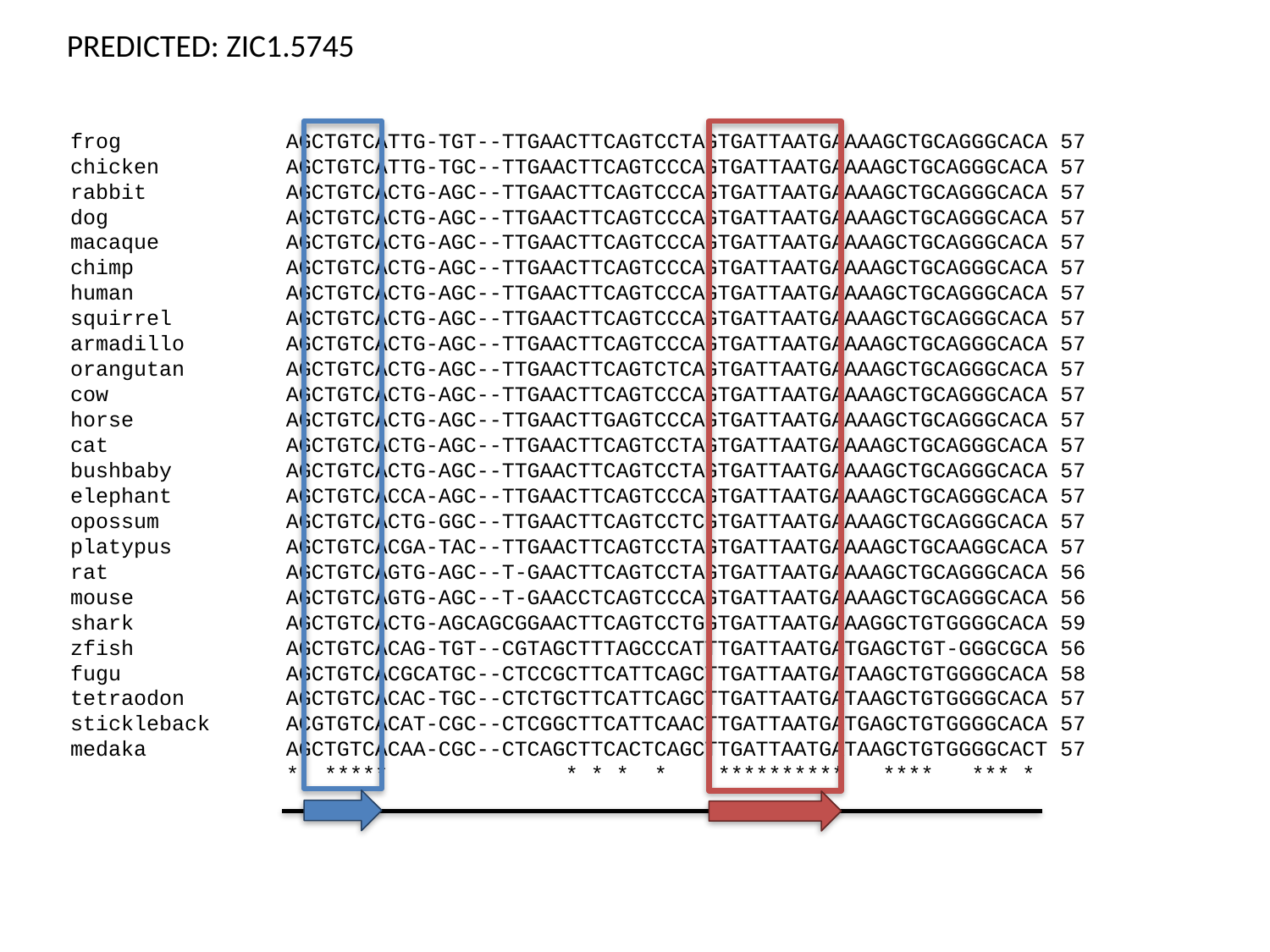

PREDICTED: ZIC1.5745
frog AGCTGTCATTG-TGT--TTGAACTTCAGTCCTAGTGATTAATGAAAAGCTGCAGGGCACA 57
chicken AGCTGTCATTG-TGC--TTGAACTTCAGTCCCAGTGATTAATGAAAAGCTGCAGGGCACA 57
rabbit AGCTGTCACTG-AGC--TTGAACTTCAGTCCCAGTGATTAATGAAAAGCTGCAGGGCACA 57
dog AGCTGTCACTG-AGC--TTGAACTTCAGTCCCAGTGATTAATGAAAAGCTGCAGGGCACA 57
macaque AGCTGTCACTG-AGC--TTGAACTTCAGTCCCAGTGATTAATGAAAAGCTGCAGGGCACA 57
chimp AGCTGTCACTG-AGC--TTGAACTTCAGTCCCAGTGATTAATGAAAAGCTGCAGGGCACA 57
human AGCTGTCACTG-AGC--TTGAACTTCAGTCCCAGTGATTAATGAAAAGCTGCAGGGCACA 57
squirrel AGCTGTCACTG-AGC--TTGAACTTCAGTCCCAGTGATTAATGAAAAGCTGCAGGGCACA 57
armadillo AGCTGTCACTG-AGC--TTGAACTTCAGTCCCAGTGATTAATGAAAAGCTGCAGGGCACA 57
orangutan AGCTGTCACTG-AGC--TTGAACTTCAGTCTCAGTGATTAATGAAAAGCTGCAGGGCACA 57
cow AGCTGTCACTG-AGC--TTGAACTTCAGTCCCAGTGATTAATGAAAAGCTGCAGGGCACA 57
horse AGCTGTCACTG-AGC--TTGAACTTGAGTCCCAGTGATTAATGAAAAGCTGCAGGGCACA 57
cat AGCTGTCACTG-AGC--TTGAACTTCAGTCCTAGTGATTAATGAAAAGCTGCAGGGCACA 57
bushbaby AGCTGTCACTG-AGC--TTGAACTTCAGTCCTAGTGATTAATGAAAAGCTGCAGGGCACA 57
elephant AGCTGTCACCA-AGC--TTGAACTTCAGTCCCAGTGATTAATGAAAAGCTGCAGGGCACA 57
opossum AGCTGTCACTG-GGC--TTGAACTTCAGTCCTCGTGATTAATGAAAAGCTGCAGGGCACA 57
platypus AGCTGTCACGA-TAC--TTGAACTTCAGTCCTAGTGATTAATGAAAAGCTGCAAGGCACA 57
rat AGCTGTCAGTG-AGC--T-GAACTTCAGTCCTAGTGATTAATGAAAAGCTGCAGGGCACA 56
mouse AGCTGTCAGTG-AGC--T-GAACCTCAGTCCCAGTGATTAATGAAAAGCTGCAGGGCACA 56
shark AGCTGTCACTG-AGCAGCGGAACTTCAGTCCTGGTGATTAATGAAAGGCTGTGGGGCACA 59
zfish AGCTGTCACAG-TGT--CGTAGCTTTAGCCCATTTGATTAATGATGAGCTGT-GGGCGCA 56
fugu AGCTGTCACGCATGC--CTCCGCTTCATTCAGCTTGATTAATGATAAGCTGTGGGGCACA 58
tetraodon AGCTGTCACAC-TGC--CTCTGCTTCATTCAGCTTGATTAATGATAAGCTGTGGGGCACA 57
stickleback ACGTGTCACAT-CGC--CTCGGCTTCATTCAACTTGATTAATGATGAGCTGTGGGGCACA 57
medaka AGCTGTCACAA-CGC--CTCAGCTTCACTCAGCTTGATTAATGATAAGCTGTGGGGCACT 57
 * ***** * * * * ********** **** *** *

## Slide 71
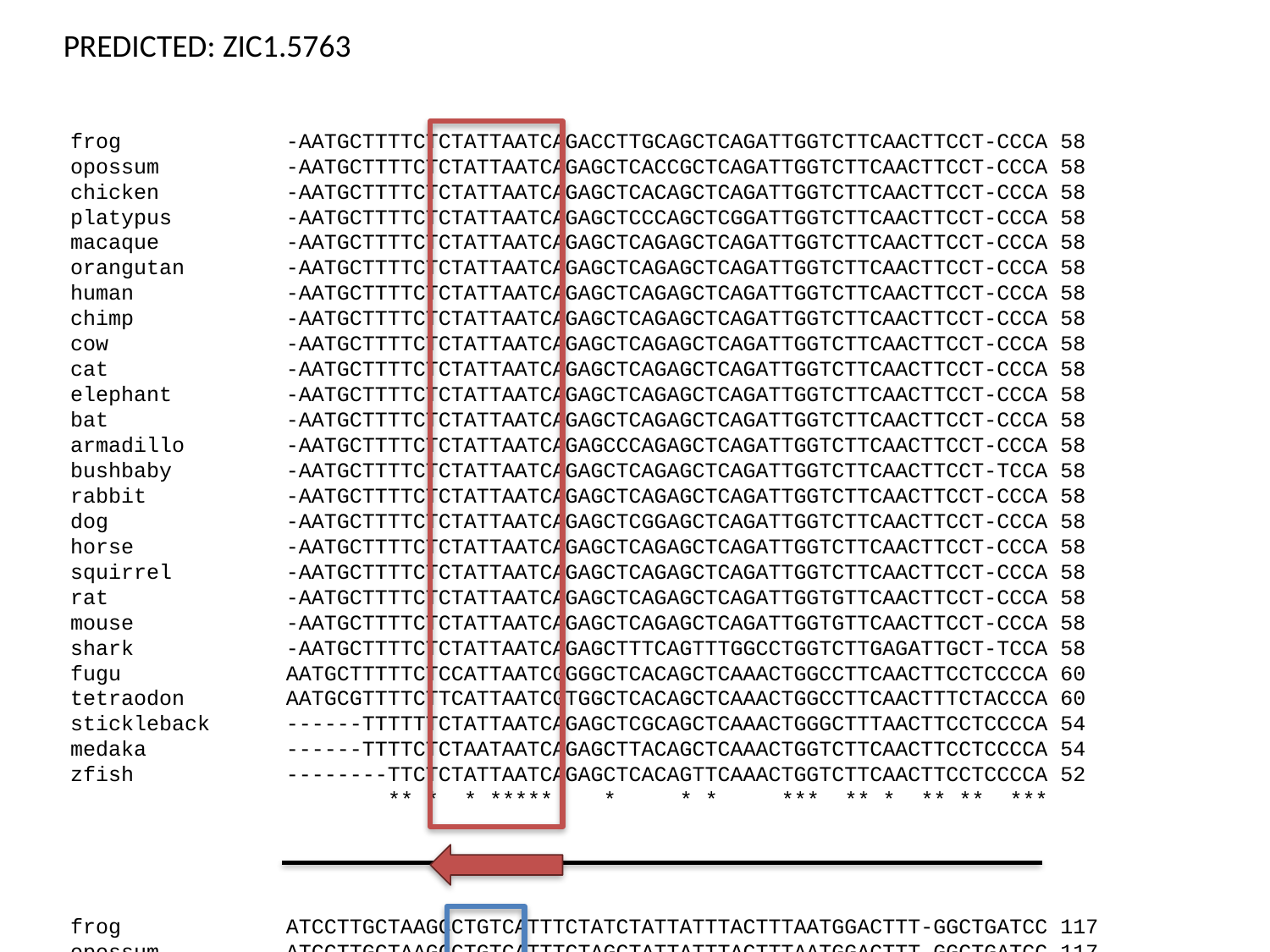

PREDICTED: ZIC1.5763
frog -AATGCTTTTCTCTATTAATCAGACCTTGCAGCTCAGATTGGTCTTCAACTTCCT-CCCA 58
opossum -AATGCTTTTCTCTATTAATCAGAGCTCACCGCTCAGATTGGTCTTCAACTTCCT-CCCA 58
chicken -AATGCTTTTCTCTATTAATCAGAGCTCACAGCTCAGATTGGTCTTCAACTTCCT-CCCA 58
platypus -AATGCTTTTCTCTATTAATCAGAGCTCCCAGCTCGGATTGGTCTTCAACTTCCT-CCCA 58
macaque -AATGCTTTTCTCTATTAATCAGAGCTCAGAGCTCAGATTGGTCTTCAACTTCCT-CCCA 58
orangutan -AATGCTTTTCTCTATTAATCAGAGCTCAGAGCTCAGATTGGTCTTCAACTTCCT-CCCA 58
human -AATGCTTTTCTCTATTAATCAGAGCTCAGAGCTCAGATTGGTCTTCAACTTCCT-CCCA 58
chimp -AATGCTTTTCTCTATTAATCAGAGCTCAGAGCTCAGATTGGTCTTCAACTTCCT-CCCA 58
cow -AATGCTTTTCTCTATTAATCAGAGCTCAGAGCTCAGATTGGTCTTCAACTTCCT-CCCA 58
cat -AATGCTTTTCTCTATTAATCAGAGCTCAGAGCTCAGATTGGTCTTCAACTTCCT-CCCA 58
elephant -AATGCTTTTCTCTATTAATCAGAGCTCAGAGCTCAGATTGGTCTTCAACTTCCT-CCCA 58
bat -AATGCTTTTCTCTATTAATCAGAGCTCAGAGCTCAGATTGGTCTTCAACTTCCT-CCCA 58
armadillo -AATGCTTTTCTCTATTAATCAGAGCCCAGAGCTCAGATTGGTCTTCAACTTCCT-CCCA 58
bushbaby -AATGCTTTTCTCTATTAATCAGAGCTCAGAGCTCAGATTGGTCTTCAACTTCCT-TCCA 58
rabbit -AATGCTTTTCTCTATTAATCAGAGCTCAGAGCTCAGATTGGTCTTCAACTTCCT-CCCA 58
dog -AATGCTTTTCTCTATTAATCAGAGCTCGGAGCTCAGATTGGTCTTCAACTTCCT-CCCA 58
horse -AATGCTTTTCTCTATTAATCAGAGCTCAGAGCTCAGATTGGTCTTCAACTTCCT-CCCA 58
squirrel -AATGCTTTTCTCTATTAATCAGAGCTCAGAGCTCAGATTGGTCTTCAACTTCCT-CCCA 58
rat -AATGCTTTTCTCTATTAATCAGAGCTCAGAGCTCAGATTGGTGTTCAACTTCCT-CCCA 58
mouse -AATGCTTTTCTCTATTAATCAGAGCTCAGAGCTCAGATTGGTGTTCAACTTCCT-CCCA 58
shark -AATGCTTTTCTCTATTAATCAGAGCTTTCAGTTTGGCCTGGTCTTGAGATTGCT-TCCA 58
fugu AATGCTTTTTCTCCATTAATCGGGGCTCACAGCTCAAACTGGCCTTCAACTTCCTCCCCA 60
tetraodon AATGCGTTTTCTTCATTAATCGTGGCTCACAGCTCAAACTGGCCTTCAACTTTCTACCCA 60
stickleback ------TTTTTTCTATTAATCAGAGCTCGCAGCTCAAACTGGGCTTTAACTTCCTCCCCA 54
medaka ------TTTTCTCTAATAATCAGAGCTTACAGCTCAAACTGGTCTTCAACTTCCTCCCCA 54
zfish --------TTCTCTATTAATCAGAGCTCACAGTTCAAACTGGTCTTCAACTTCCTCCCCA 52
 ** * * ***** * * * *** ** * ** ** ***
frog ATCCTTGCTAAGCCTGTCATTTCTATCTATTATTTACTTTAATGGACTTT-GGCTGATCC 117
opossum ATCCTTGCTAAGCCTGTCATTTCTAGCTATTATTTACTTTAATGGACTTT-GGCTGATCC 117
chicken ATCCTTGCTAAGCCTGTCATTTCTAGCTATTATTTACTTTAATGGACTTT-GGCTGATCC 117
platypus ATCCTTGCTAAGCCTGTCATTTCTAGCTATTATTTACTTTAATGGACTTT-GGCTGATCC 117
macaque ATCCTTGCTAAGCCTGTCATTTCTAGCTATTATTTACTTTAATGGACTTT-GGCTGATCC 117
orangutan ATCCTTGCTAAGCCTGTCATTTCTAGCTATTATTTACTTTAATGGACTTT-GGCTGATCC 117
human ATCCTTGCTAAGCCTGTCATTTCTAGCTATTATTTACTTTAATGGACTTT-GGCTGATCC 117
chimp ATCCTTGCTAAGCCTGTCATTTCTAGCTATTATTTACTTTAATGGACTTT-GGCTGATCC 117
cow ATCCTTGCTAAGCCTGTCATTTCTAGCTATTATTTACTTTAATGGACTTT-GGCTGATCC 117
cat ATCCTTGCTAAGCCTGTCATTTCTAGCTATTATTTACTTTAATGGACTTT-GGCTGATCC 117
elephant ATCCTTGCTAAGCCTGTCATTTCTAGCTATTATTTACTTTAATGGACTTT-GGCTGATCC 117
bat ATCTTTGCTAAGCCTGTCATTTCTAGCTATTATTTACTTTAATGGACTTT-GGCTGATCT 117
armadillo ATCCTTGCTAAGCCTGTCATTTCTAGCTATTATTTACTTTAATGGACTTT-GGCTGATCC 117
bushbaby ATCCTTGCTAAGCCTGTCATTTCTAGCTATTATTTACTTTAATGGACTTT-GGCTGATCC 117
rabbit ATCCTTGCTAAGCCTGTCATTTCTAGCTATTATTTACTTTAATGGACTTT-GGCTGATCC 117
dog ATCCTTGCTAAGCCTGTCATTTCTAGCTATTATTTACTTTAATGGATTTT-GGCTGATCC 117
horse ATCCTTGCTAAGCCTGTCATTTCTAGCTATTATTTACTTTAATGGACTTT-GGCTGATCC 117
squirrel ATCCTTGCTAAGCCTGTCATTTCTAGCTATTATTTACTTTAATGGACTTT-GGCTGATCC 117
rat ATCCTTGCTAAGCCTGTCATTTCTGGCTATTATTTACTTTAATGGACTTT-GGCTGATCC 117
mouse ATCCTTGCTAAGCCTGTCATTTCTGGCTATTATTTACTTTAATGGACTTT-GGCTGATCC 117
shark ATTCTGGATAAGCTCGTCGTTTCTAGCTATTATTTAATTTAATGGACTTTTGGCTGATCC 118
fugu ATCCTTGCTAAGCCTGTCATTTCTGGCTATTATTTACTTTAATGGGCTTT-GGCTGATCC 119
tetraodon ATCCTCACTAAGCCTGTCATTTCTGGCTATTATTTACTTTAATGGGCTTT-GGCTGATCC 119
stickleback ATCCTGGGTAAGCCTGGGATTTCTGGGTATTATTTACTTTAATGGGCTTT-GGGTGATCC 113
medaka ATCCGTGCTAAGCCTGTCATTTCTGGCTATTATTTACTTTAATGGGCTTT-GGCTGATCC 113
zfish ATCCGCACTAAGCCTGTCATTTCTGGCTATTATTTACTTTAATGGGCTTT-GGCTGATCC 111
 ** ***** * ***** ********* ******** *** ** *****

## Slide 72
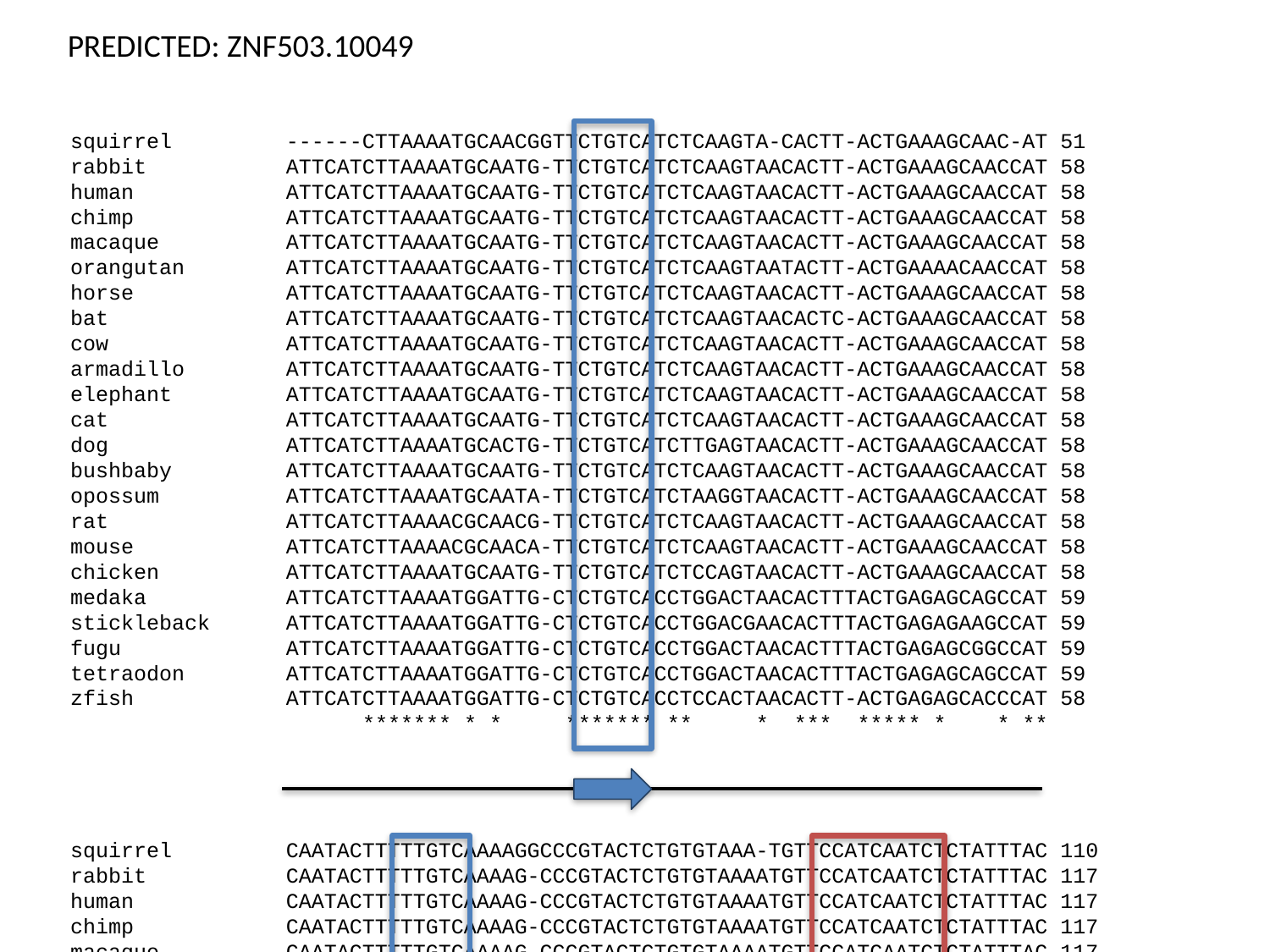

PREDICTED: ZNF503.10049
squirrel ------CTTAAAATGCAACGGTTCTGTCATCTCAAGTA-CACTT-ACTGAAAGCAAC-AT 51
rabbit ATTCATCTTAAAATGCAATG-TTCTGTCATCTCAAGTAACACTT-ACTGAAAGCAACCAT 58
human ATTCATCTTAAAATGCAATG-TTCTGTCATCTCAAGTAACACTT-ACTGAAAGCAACCAT 58
chimp ATTCATCTTAAAATGCAATG-TTCTGTCATCTCAAGTAACACTT-ACTGAAAGCAACCAT 58
macaque ATTCATCTTAAAATGCAATG-TTCTGTCATCTCAAGTAACACTT-ACTGAAAGCAACCAT 58
orangutan ATTCATCTTAAAATGCAATG-TTCTGTCATCTCAAGTAATACTT-ACTGAAAACAACCAT 58
horse ATTCATCTTAAAATGCAATG-TTCTGTCATCTCAAGTAACACTT-ACTGAAAGCAACCAT 58
bat ATTCATCTTAAAATGCAATG-TTCTGTCATCTCAAGTAACACTC-ACTGAAAGCAACCAT 58
cow ATTCATCTTAAAATGCAATG-TTCTGTCATCTCAAGTAACACTT-ACTGAAAGCAACCAT 58
armadillo ATTCATCTTAAAATGCAATG-TTCTGTCATCTCAAGTAACACTT-ACTGAAAGCAACCAT 58
elephant ATTCATCTTAAAATGCAATG-TTCTGTCATCTCAAGTAACACTT-ACTGAAAGCAACCAT 58
cat ATTCATCTTAAAATGCAATG-TTCTGTCATCTCAAGTAACACTT-ACTGAAAGCAACCAT 58
dog ATTCATCTTAAAATGCACTG-TTCTGTCATCTTGAGTAACACTT-ACTGAAAGCAACCAT 58
bushbaby ATTCATCTTAAAATGCAATG-TTCTGTCATCTCAAGTAACACTT-ACTGAAAGCAACCAT 58
opossum ATTCATCTTAAAATGCAATA-TTCTGTCATCTAAGGTAACACTT-ACTGAAAGCAACCAT 58
rat ATTCATCTTAAAACGCAACG-TTCTGTCATCTCAAGTAACACTT-ACTGAAAGCAACCAT 58
mouse ATTCATCTTAAAACGCAACA-TTCTGTCATCTCAAGTAACACTT-ACTGAAAGCAACCAT 58
chicken ATTCATCTTAAAATGCAATG-TTCTGTCATCTCCAGTAACACTT-ACTGAAAGCAACCAT 58
medaka ATTCATCTTAAAATGGATTG-CTCTGTCACCTGGACTAACACTTTACTGAGAGCAGCCAT 59
stickleback ATTCATCTTAAAATGGATTG-CTCTGTCACCTGGACGAACACTTTACTGAGAGAAGCCAT 59
fugu ATTCATCTTAAAATGGATTG-CTCTGTCACCTGGACTAACACTTTACTGAGAGCGGCCAT 59
tetraodon ATTCATCTTAAAATGGATTG-CTCTGTCACCTGGACTAACACTTTACTGAGAGCAGCCAT 59
zfish ATTCATCTTAAAATGGATTG-CTCTGTCACCTCCACTAACACTT-ACTGAGAGCACCCAT 58
 ******* * * ******* ** * *** ***** * * **
squirrel CAATACTTTTTGTCAAAAGGCCCGTACTCTGTGTAAA-TGTTCCATCAATCTCTATTTAC 110
rabbit CAATACTTTTTGTCAAAAG-CCCGTACTCTGTGTAAAATGTTCCATCAATCTCTATTTAC 117
human CAATACTTTTTGTCAAAAG-CCCGTACTCTGTGTAAAATGTTCCATCAATCTCTATTTAC 117
chimp CAATACTTTTTGTCAAAAG-CCCGTACTCTGTGTAAAATGTTCCATCAATCTCTATTTAC 117
macaque CAATACTTTTTGTCAAAAG-CCCGTACTCTGTGTAAAATGTTCCATCAATCTCTATTTAC 117
orangutan CAATACTTTTTGTCAAAAG-CCCGTACTCTGTGTAAAATGTTCCATCAATCTCTATTTAC 117
horse CAATACTTTTTGTCAAAAG-CCCGTACTCTGTGTAAAATGTTCCATCAATCTCTATTTAC 117
bat CAATACTTTTTGTCAAAAG-CCTGTACTCTGTGTAAAATGTTCCATCAATCTCTATTTAC 117
cow CAATACTTTTTGTCAAAAG-ACTGTACTCTGTGTAAAATGTTCCATCAATCTCTATTTAC 117
armadillo CAATACTTTTTGTCAAAAG-CCTGTACTCTGTGTAAAATGTTCCATCAATCTCTATTTAC 117
elephant CAATACTTTTTGTCAAAAG-CCTGTACTCTGTGTAAAATGTTCCATCAATCTCTATTTAC 117
cat CAATACTTTTTGTCAAAAG-CCCGTACTCTGTGTAAAATGTTCCATCAATCTCTATTTAC 117
dog CAATACTTTTTGTCAAAAG-CCCGTACTCTGTGTAAAATGTTCCATCAATCTCTATTTAC 117
bushbaby CAATACTTTTTGTCAAAAG-CCCGTACTCTGTGTAAAATGTTCCATCAATCTCTATTTAC 117
opossum CAATACTTTTTGTCAAAAA-CCCGTACTCTGTGTAAAATGTTCCATCAATCTCTATTTAC 117
rat CAATACTTTTTGTCAAAAG-CCTGTACTCTGTGTAAAATGTTCCATCAATCTCTATTTAC 117
mouse CAATACTTTTTGTCAAAAG-CCTGTACTCTGTGTAAAATGTTCCATCAATCTCTATTTAC 117
chicken CAATACTTTTTGTCAAAAA-CCTGCACTCTGTGTAAAATGTTCCATCAATCTCTATTTAC 117
medaka CAATACTTTTTGTCAAAAA--CTGCAGGCTGTGCCAGACATGCCATCAATCACCATTTAC 117
stickleback CAATACTTTTTGTCAAAAA--CTGCAGGCAGTGCCAGACATGCCATCAATCACCATTTAC 117
fugu CAATACTTTTTGTCAAAAA--CTGCAGGCTTCGCCAGACGTGTCATCAATCACCATTTAC 117
tetraodon CAATACTTTTTGTCTAAAA--CTGCAGGCTTCACCAGACATGTCATCAATCACCATTTAC 117
zfish CAATACTTTTTGTCAAAAG--CTGCACACTAGGTAAGATGTGCCATCAATCACTATTTAC 116
 ************** *** * * * * * * ******** * ******

## Slide 73
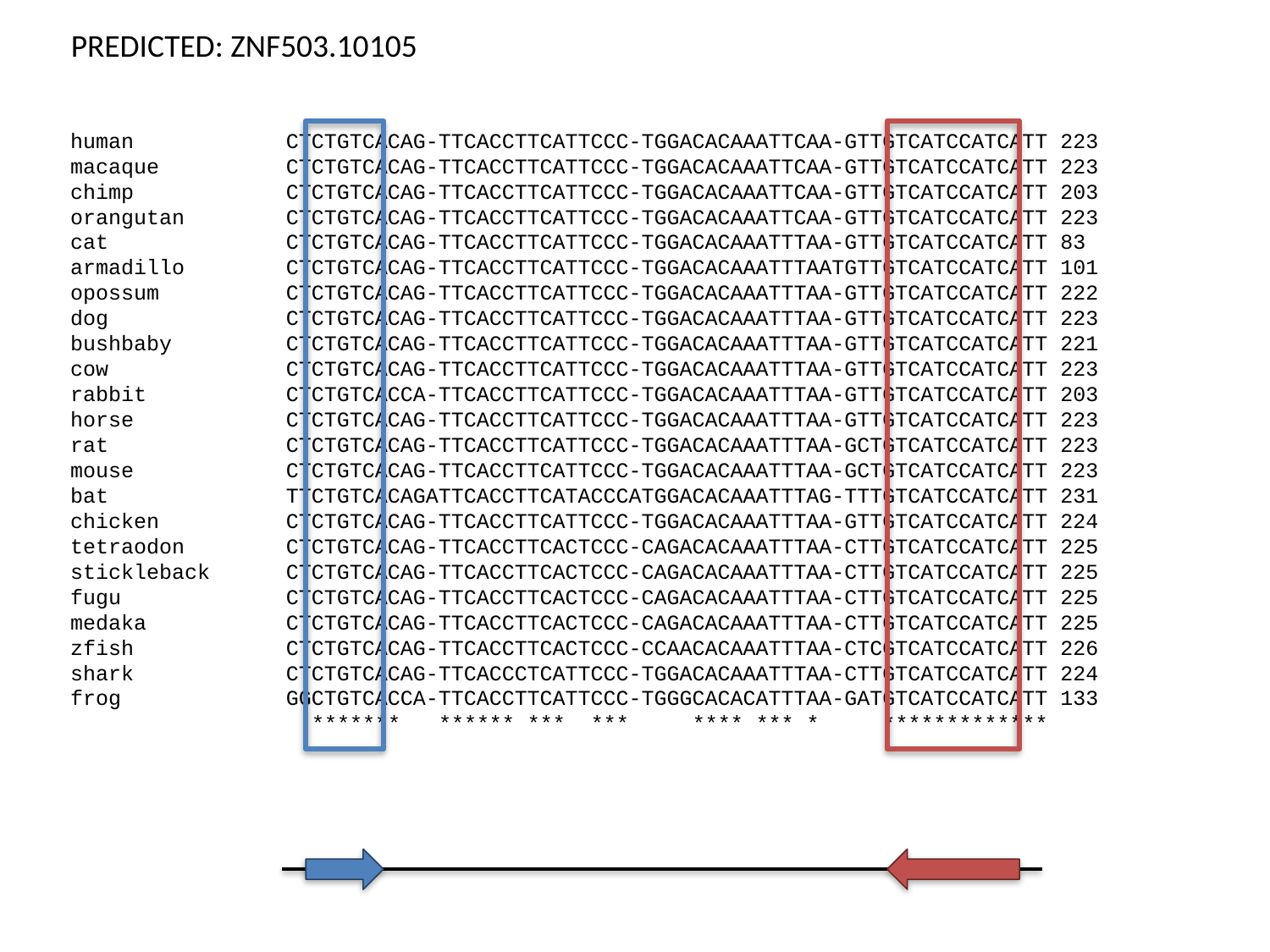

PREDICTED: ZNF503.10105
human CTCTGTCACAG-TTCACCTTCATTCCC-TGGACACAAATTCAA-GTTGTCATCCATCATT 223
macaque CTCTGTCACAG-TTCACCTTCATTCCC-TGGACACAAATTCAA-GTTGTCATCCATCATT 223
chimp CTCTGTCACAG-TTCACCTTCATTCCC-TGGACACAAATTCAA-GTTGTCATCCATCATT 203
orangutan CTCTGTCACAG-TTCACCTTCATTCCC-TGGACACAAATTCAA-GTTGTCATCCATCATT 223
cat CTCTGTCACAG-TTCACCTTCATTCCC-TGGACACAAATTTAA-GTTGTCATCCATCATT 83
armadillo CTCTGTCACAG-TTCACCTTCATTCCC-TGGACACAAATTTAATGTTGTCATCCATCATT 101
opossum CTCTGTCACAG-TTCACCTTCATTCCC-TGGACACAAATTTAA-GTTGTCATCCATCATT 222
dog CTCTGTCACAG-TTCACCTTCATTCCC-TGGACACAAATTTAA-GTTGTCATCCATCATT 223
bushbaby CTCTGTCACAG-TTCACCTTCATTCCC-TGGACACAAATTTAA-GTTGTCATCCATCATT 221
cow CTCTGTCACAG-TTCACCTTCATTCCC-TGGACACAAATTTAA-GTTGTCATCCATCATT 223
rabbit CTCTGTCACCA-TTCACCTTCATTCCC-TGGACACAAATTTAA-GTTGTCATCCATCATT 203
horse CTCTGTCACAG-TTCACCTTCATTCCC-TGGACACAAATTTAA-GTTGTCATCCATCATT 223
rat CTCTGTCACAG-TTCACCTTCATTCCC-TGGACACAAATTTAA-GCTGTCATCCATCATT 223
mouse CTCTGTCACAG-TTCACCTTCATTCCC-TGGACACAAATTTAA-GCTGTCATCCATCATT 223
bat TTCTGTCACAGATTCACCTTCATACCCATGGACACAAATTTAG-TTTGTCATCCATCATT 231
chicken CTCTGTCACAG-TTCACCTTCATTCCC-TGGACACAAATTTAA-GTTGTCATCCATCATT 224
tetraodon CTCTGTCACAG-TTCACCTTCACTCCC-CAGACACAAATTTAA-CTTGTCATCCATCATT 225
stickleback CTCTGTCACAG-TTCACCTTCACTCCC-CAGACACAAATTTAA-CTTGTCATCCATCATT 225
fugu CTCTGTCACAG-TTCACCTTCACTCCC-CAGACACAAATTTAA-CTTGTCATCCATCATT 225
medaka CTCTGTCACAG-TTCACCTTCACTCCC-CAGACACAAATTTAA-CTTGTCATCCATCATT 225
zfish CTCTGTCACAG-TTCACCTTCACTCCC-CCAACACAAATTTAA-CTCGTCATCCATCATT 226
shark CTCTGTCACAG-TTCACCCTCATTCCC-TGGACACAAATTTAA-CTTGTCATCCATCATT 224
frog GGCTGTCACCA-TTCACCTTCATTCCC-TGGGCACACATTTAA-GATGTCATCCATCATT 133
 ******* ****** *** *** **** *** * *************

## Slide 74
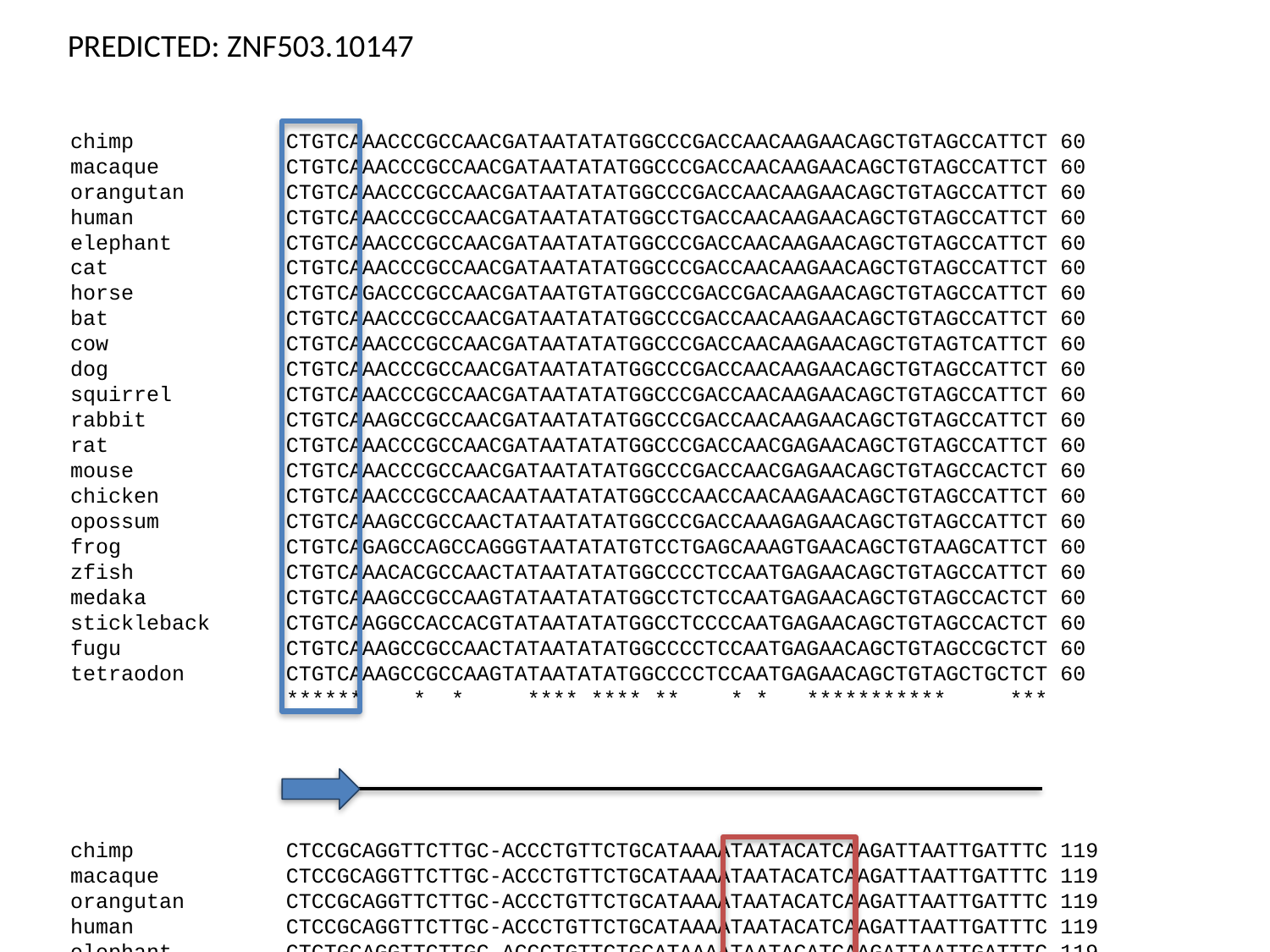

PREDICTED: ZNF503.10147
chimp CTGTCAAACCCGCCAACGATAATATATGGCCCGACCAACAAGAACAGCTGTAGCCATTCT 60
macaque CTGTCAAACCCGCCAACGATAATATATGGCCCGACCAACAAGAACAGCTGTAGCCATTCT 60
orangutan CTGTCAAACCCGCCAACGATAATATATGGCCCGACCAACAAGAACAGCTGTAGCCATTCT 60
human CTGTCAAACCCGCCAACGATAATATATGGCCTGACCAACAAGAACAGCTGTAGCCATTCT 60
elephant CTGTCAAACCCGCCAACGATAATATATGGCCCGACCAACAAGAACAGCTGTAGCCATTCT 60
cat CTGTCAAACCCGCCAACGATAATATATGGCCCGACCAACAAGAACAGCTGTAGCCATTCT 60
horse CTGTCAGACCCGCCAACGATAATGTATGGCCCGACCGACAAGAACAGCTGTAGCCATTCT 60
bat CTGTCAAACCCGCCAACGATAATATATGGCCCGACCAACAAGAACAGCTGTAGCCATTCT 60
cow CTGTCAAACCCGCCAACGATAATATATGGCCCGACCAACAAGAACAGCTGTAGTCATTCT 60
dog CTGTCAAACCCGCCAACGATAATATATGGCCCGACCAACAAGAACAGCTGTAGCCATTCT 60
squirrel CTGTCAAACCCGCCAACGATAATATATGGCCCGACCAACAAGAACAGCTGTAGCCATTCT 60
rabbit CTGTCAAAGCCGCCAACGATAATATATGGCCCGACCAACAAGAACAGCTGTAGCCATTCT 60
rat CTGTCAAACCCGCCAACGATAATATATGGCCCGACCAACGAGAACAGCTGTAGCCATTCT 60
mouse CTGTCAAACCCGCCAACGATAATATATGGCCCGACCAACGAGAACAGCTGTAGCCACTCT 60
chicken CTGTCAAACCCGCCAACAATAATATATGGCCCAACCAACAAGAACAGCTGTAGCCATTCT 60
opossum CTGTCAAAGCCGCCAACTATAATATATGGCCCGACCAAAGAGAACAGCTGTAGCCATTCT 60
frog CTGTCAGAGCCAGCCAGGGTAATATATGTCCTGAGCAAAGTGAACAGCTGTAAGCATTCT 60
zfish CTGTCAAACACGCCAACTATAATATATGGCCCCTCCAATGAGAACAGCTGTAGCCATTCT 60
medaka CTGTCAAAGCCGCCAAGTATAATATATGGCCTCTCCAATGAGAACAGCTGTAGCCACTCT 60
stickleback CTGTCAAGGCCACCACGTATAATATATGGCCTCCCCAATGAGAACAGCTGTAGCCACTCT 60
fugu CTGTCAAAGCCGCCAACTATAATATATGGCCCCTCCAATGAGAACAGCTGTAGCCGCTCT 60
tetraodon CTGTCAAAGCCGCCAAGTATAATATATGGCCCCTCCAATGAGAACAGCTGTAGCTGCTCT 60
 ****** * * **** **** ** * * *********** ***
chimp CTCCGCAGGTTCTTGC-ACCCTGTTCTGCATAAAATAATACATCAAGATTAATTGATTTC 119
macaque CTCCGCAGGTTCTTGC-ACCCTGTTCTGCATAAAATAATACATCAAGATTAATTGATTTC 119
orangutan CTCCGCAGGTTCTTGC-ACCCTGTTCTGCATAAAATAATACATCAAGATTAATTGATTTC 119
human CTCCGCAGGTTCTTGC-ACCCTGTTCTGCATAAAATAATACATCAAGATTAATTGATTTC 119
elephant CTCTGCAGGTTCTTGC-ACCCTGTTCTGCATAAAATAATACATCAAGATTAATTGATTTC 119
cat CTCTGCAGGTTCCTGC-ACCCTGTTCTGCATAAAATAATACATCAAGATTAATTGATTTC 119
horse CTCTGCAGGTTCTTGC-ACCCTGTTCTGCATAAAATAATACATCAAGATTAATTGATTTC 119
bat CTCTGCAGGTTCTTGC-ACCCTGTTCTGCATAAAATAATACATCAAGATTAATTGATTTC 119
cow CTCTGCAGGTTCTCGC-ACCCTGTTCTGCATAAAATAATACATCAAGATTAATTGATTTC 119
dog CTCTGCAGGTTCTTGC-ACCCTGTTCTGCATAAAATAATACATCAAGATTAATTGATTTC 119
squirrel CTCGGCAGGTTCTTGC-ACCCTGTTCTGCATAAAATAATACATCAAGATTAATTGATTTC 119
rabbit CTCCGCAGGTTCTTGC-ACCCTGTTCTGCATAAAATAATACATCAAGATTAATTGATTTC 119
rat CTCGGCAGGTTCCTGC-ACCCTGTTCTGCATAAAATAATACATCAAGATTAATTGATTTC 119
mouse CTCGGCAGGTTCCTGC-ACCCTGTTCTGCATAAAATAATACATCAAGATTAATTGATTTC 119
chicken CTCTGCTGGTTCCTGC-ACCCTGTTCTGCATAAAATAATACATCAAGATTAATTGATTTC 119
opossum CTCTCCTGGTTCCTGT-ACCCTGCTCTGCATAAAATAATATATCAAGATTAATTGATTTC 119
frog CTGTGCTGCTCCCTGT-ACCCTGTCCTGCATAAAATAATACATCAAGATTAATTGATTTG 119
zfish CTCCGCTGCCTCTTGT-ACCCCGTCCTGCATGAAATAATATATCAAGATTAATTGATTTC 119
medaka CTCTGC-TGCT---GCTACCCTGCCCTGCACGAAATAATACATCAAGATTAATTGATTTC 116
stickleback CTCTCTGTGCT---GCTGC--TACCCTGCATGAAATAATACATCAAGATTAATTGATTTC 115
fugu CTCCGC-TGCCACGGCTACCCTGCCCCGCACCCAATAATACATCAAGATTAATTGATTTC 119
tetraodon CTCTGC-TGCCACGGCTAGCCTGCCCCACACCCAATAATACATCAAGATTAATTGATTTC 119
 ** * * ** ******* ******************

## Slide 75
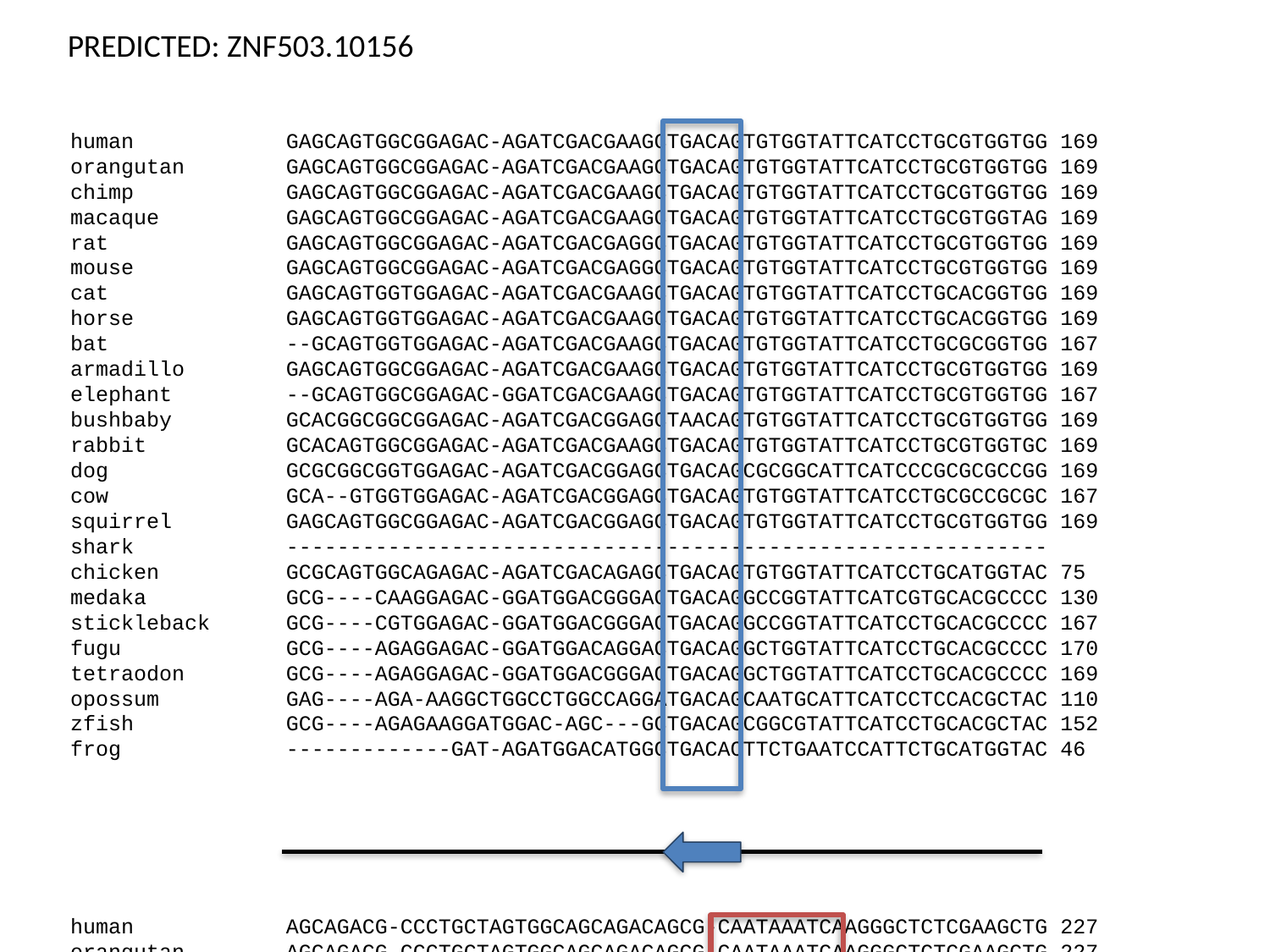

PREDICTED: ZNF503.10156
human GAGCAGTGGCGGAGAC-AGATCGACGAAGCTGACAGTGTGGTATTCATCCTGCGTGGTGG 169
orangutan GAGCAGTGGCGGAGAC-AGATCGACGAAGCTGACAGTGTGGTATTCATCCTGCGTGGTGG 169
chimp GAGCAGTGGCGGAGAC-AGATCGACGAAGCTGACAGTGTGGTATTCATCCTGCGTGGTGG 169
macaque GAGCAGTGGCGGAGAC-AGATCGACGAAGCTGACAGTGTGGTATTCATCCTGCGTGGTAG 169
rat GAGCAGTGGCGGAGAC-AGATCGACGAGGCTGACAGTGTGGTATTCATCCTGCGTGGTGG 169
mouse GAGCAGTGGCGGAGAC-AGATCGACGAGGCTGACAGTGTGGTATTCATCCTGCGTGGTGG 169
cat GAGCAGTGGTGGAGAC-AGATCGACGAAGCTGACAGTGTGGTATTCATCCTGCACGGTGG 169
horse GAGCAGTGGTGGAGAC-AGATCGACGAAGCTGACAGTGTGGTATTCATCCTGCACGGTGG 169
bat --GCAGTGGTGGAGAC-AGATCGACGAAGCTGACAGTGTGGTATTCATCCTGCGCGGTGG 167
armadillo GAGCAGTGGCGGAGAC-AGATCGACGAAGCTGACAGTGTGGTATTCATCCTGCGTGGTGG 169
elephant --GCAGTGGCGGAGAC-GGATCGACGAAGCTGACAGTGTGGTATTCATCCTGCGTGGTGG 167
bushbaby GCACGGCGGCGGAGAC-AGATCGACGGAGCTAACAGTGTGGTATTCATCCTGCGTGGTGG 169
rabbit GCACAGTGGCGGAGAC-AGATCGACGAAGCTGACAGTGTGGTATTCATCCTGCGTGGTGC 169
dog GCGCGGCGGTGGAGAC-AGATCGACGGAGCTGACAGCGCGGCATTCATCCCGCGCGCCGG 169
cow GCA--GTGGTGGAGAC-AGATCGACGGAGCTGACAGTGTGGTATTCATCCTGCGCCGCGC 167
squirrel GAGCAGTGGCGGAGAC-AGATCGACGGAGCTGACAGTGTGGTATTCATCCTGCGTGGTGG 169
shark ------------------------------------------------------------
chicken GCGCAGTGGCAGAGAC-AGATCGACAGAGCTGACAGTGTGGTATTCATCCTGCATGGTAC 75
medaka GCG----CAAGGAGAC-GGATGGACGGGAGTGACAGGCCGGTATTCATCGTGCACGCCCC 130
stickleback GCG----CGTGGAGAC-GGATGGACGGGAGTGACAGGCCGGTATTCATCCTGCACGCCCC 167
fugu GCG----AGAGGAGAC-GGATGGACAGGACTGACAGGCTGGTATTCATCCTGCACGCCCC 170
tetraodon GCG----AGAGGAGAC-GGATGGACGGGAGTGACAGGCTGGTATTCATCCTGCACGCCCC 169
opossum GAG----AGA-AAGGCTGGCCTGGCCAGGATGACAGCAATGCATTCATCCTCCACGCTAC 110
zfish GCG----AGAGAAGGATGGAC-AGC---GCTGACAGCGGCGTATTCATCCTGCACGCTAC 152
frog -------------GAT-AGATGGACATGGCTGACACTTCTGAATCCATTCTGCATGGTAC 46
human AGCAGACG-CCCTGCTAGTGGCAGCAGACAGCG-CAATAAATCAAGGGCTCTCGAAGCTG 227
orangutan AGCAGACG-CCCTGCTAGTGGCAGCAGACAGCG-CAATAAATCAAGGGCTCTCGAAGCTG 227
chimp AGCAGACG-CCCCGCTAGTGGCAGCAGACAGCG-CAATAAATCAAGGGCTCTCGAAGCTG 227
macaque AGCAGACG-CCCCGCTAGTGGCAGCAGACAGCG-CAATAAATCAAGGGCTCTCGAAGCTG 227
rat AGCAGGCG-TCCCGCTAGTGGCGGGAGACAGCG-CAATAAATCAAGGGCTCTCGAAGCTG 227
mouse AGCAGGCG-TCCCGCTAGTGGCGGTAGACAGCG-CAATAAATCAAGGGCTCTCGAAGCTG 227
cat AGCAGACG-CCCCGCTAGTGGCAGTAGACAGCG-CAATAAATCAAGGGCTCTCGAAGCTG 227
horse AGCAGACG-TCCCGCTAGTGGCAGTAGACAGCG-CAATAAATCAAGGGCTCTCGAAGCTG 227
bat AGCAGGCG-TCCCGCTAGTGGCAGTAGACAGCG-CAATAAATCAAGGGCTCTCGAAGCTG 225
armadillo AGCAGACG-TCCCGCTAGTGGCAGTAGACAGCG-CAATAAATCAAGGGCTCTCGAAGCTG 227
elephant AGCAGACGTCCCCGCTAGTGGCAGTAGACAGTG-CAATAAATCAAGGGCTCTCGAAGCTG 226
bushbaby AGCAGACG-CCCCGCTAGTGGCAGTAGACAGCG-CAATAAATCAAGGGCTCTCGAAGCTG 227
rabbit GGCAGACG-GCCCGCTAGTGGCAGGAGACAGCG-CAATAAATCAAGGGCTCTCGAAGCTG 227
dog AGCAGACG-CCCCGCTAGTGGCAGGAGACAGCA-CAATAAATCAAGGGCTCTCGAAGCTG 227
cow AGCAGACG-TCCCGCTAGTGGCAGGAGACAGCG-CAATAAATCAAGGGCTCTCGAAGCTG 225
squirrel AGCAGGCG-TCCCGCTAGTGGCAGCAGACAGCG-CAATAAATCAAGGGCTCTCGAAGCTG 227
shark --CAGACG-TCCTGCTTGTGGTGGTCGACAGAG-CAATAAATCAAGGGCTGGCGATGCTG 56
chicken AGCAGACG-TCCTGCTAGTGGCAGTCGACAGTG-CAATAAATCAAGGGCTCTCGATGCTG 133
medaka AGTGGCCG-GACTGCTACTGGCGCTCGACAGAG-CAATAAATCACCAGCTCTCCACTCTG 188
stickleback AGCGGCCG-GACTGCTACTGGAGCTCGACAGAG-CAATAAATCACCAGCTCTCCAGTCTG 225
fugu AGCGGCCG-GACTGCTACTGGCGCTCGACAGAG-CAATAAATCACCAGCTCTCCAGTCTG 228
tetraodon AGCGGCCG-GACCGCTACTGGCGCTCGGCAGAG-CAATAAATCACCGGCTCTCCAGTCTG 227
opossum AGCAGACA-AAGCGCTGGCACTGCCTGACAGGGACCATAAATCATGAGCAGGAGATGCTG 169
zfish AGCAGACG-TCCCGCTAGTGCTGGTCGACAGAG-CAATAAATCAAGGGCCCTCCATGCTG 210
frog ACCAGTTG-CGACGTTACTTGCTGCAGACAGCG-CAATAAATCAAGGTCTGCCGGTGCTG 104
 * * * * *** * ******** * ***

## Slide 76
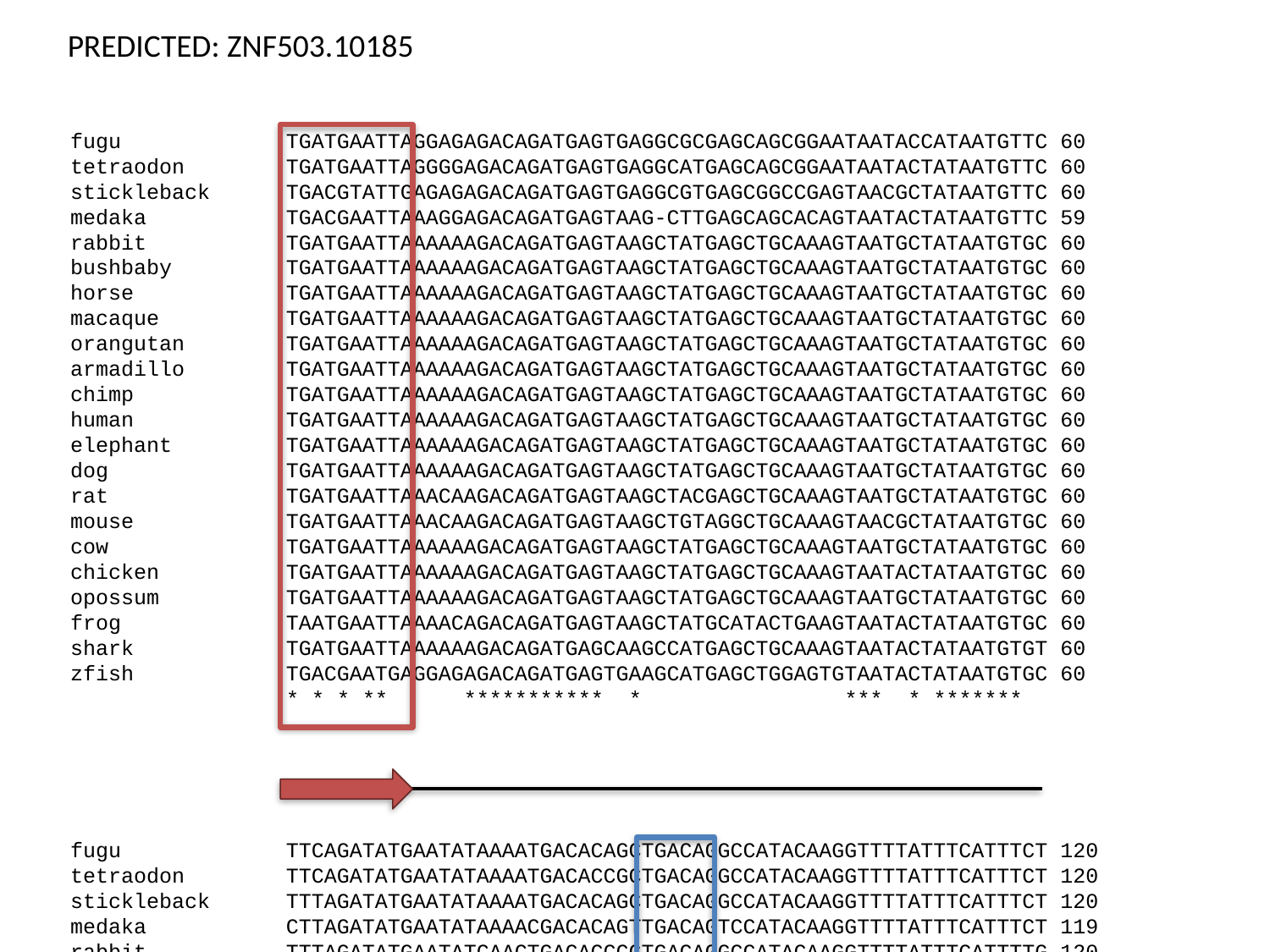

PREDICTED: ZNF503.10185
fugu TGATGAATTAGGAGAGACAGATGAGTGAGGCGCGAGCAGCGGAATAATACCATAATGTTC 60
tetraodon TGATGAATTAGGGGAGACAGATGAGTGAGGCATGAGCAGCGGAATAATACTATAATGTTC 60
stickleback TGACGTATTGAGAGAGACAGATGAGTGAGGCGTGAGCGGCCGAGTAACGCTATAATGTTC 60
medaka TGACGAATTAAAGGAGACAGATGAGTAAG-CTTGAGCAGCACAGTAATACTATAATGTTC 59
rabbit TGATGAATTAAAAAAGACAGATGAGTAAGCTATGAGCTGCAAAGTAATGCTATAATGTGC 60
bushbaby TGATGAATTAAAAAAGACAGATGAGTAAGCTATGAGCTGCAAAGTAATGCTATAATGTGC 60
horse TGATGAATTAAAAAAGACAGATGAGTAAGCTATGAGCTGCAAAGTAATGCTATAATGTGC 60
macaque TGATGAATTAAAAAAGACAGATGAGTAAGCTATGAGCTGCAAAGTAATGCTATAATGTGC 60
orangutan TGATGAATTAAAAAAGACAGATGAGTAAGCTATGAGCTGCAAAGTAATGCTATAATGTGC 60
armadillo TGATGAATTAAAAAAGACAGATGAGTAAGCTATGAGCTGCAAAGTAATGCTATAATGTGC 60
chimp TGATGAATTAAAAAAGACAGATGAGTAAGCTATGAGCTGCAAAGTAATGCTATAATGTGC 60
human TGATGAATTAAAAAAGACAGATGAGTAAGCTATGAGCTGCAAAGTAATGCTATAATGTGC 60
elephant TGATGAATTAAAAAAGACAGATGAGTAAGCTATGAGCTGCAAAGTAATGCTATAATGTGC 60
dog TGATGAATTAAAAAAGACAGATGAGTAAGCTATGAGCTGCAAAGTAATGCTATAATGTGC 60
rat TGATGAATTAAACAAGACAGATGAGTAAGCTACGAGCTGCAAAGTAATGCTATAATGTGC 60
mouse TGATGAATTAAACAAGACAGATGAGTAAGCTGTAGGCTGCAAAGTAACGCTATAATGTGC 60
cow TGATGAATTAAAAAAGACAGATGAGTAAGCTATGAGCTGCAAAGTAATGCTATAATGTGC 60
chicken TGATGAATTAAAAAAGACAGATGAGTAAGCTATGAGCTGCAAAGTAATACTATAATGTGC 60
opossum TGATGAATTAAAAAAGACAGATGAGTAAGCTATGAGCTGCAAAGTAATGCTATAATGTGC 60
frog TAATGAATTAAAACAGACAGATGAGTAAGCTATGCATACTGAAGTAATACTATAATGTGC 60
shark TGATGAATTAAAAAAGACAGATGAGCAAGCCATGAGCTGCAAAGTAATACTATAATGTGT 60
zfish TGACGAATGAGGAGAGACAGATGAGTGAAGCATGAGCTGGAGTGTAATACTATAATGTGC 60
 * * * ** *********** * *** * *******
fugu TTCAGATATGAATATAAAATGACACAGCTGACAGGCCATACAAGGTTTTATTTCATTTCT 120
tetraodon TTCAGATATGAATATAAAATGACACCGCTGACAGGCCATACAAGGTTTTATTTCATTTCT 120
stickleback TTTAGATATGAATATAAAATGACACAGCTGACAGGCCATACAAGGTTTTATTTCATTTCT 120
medaka CTTAGATATGAATATAAAACGACACAGTTGACAGTCCATACAAGGTTTTATTTCATTTCT 119
rabbit TTTAGATATGAATATCAACTGACACCCCTGACAGGCCATACAAGGTTTTATTTCATTTTG 120
bushbaby TTTAGATATGAATATCAACTGACACCCCTGACAGGCCATACAAGGTTTTATTTCATTTTG 120
horse TTTAGATATGAATATCAACTGACACCCCTGACAGGCCATACAAGGTTTTATTTCATTTTG 120
macaque TTTAGATATGAATATCAACTGACACCCCTGACAGGCCATACAAGGTTTTATTTCATTTTG 120
orangutan TTTAGATATGAATATCAACTGACACCCCTGACAGGCCATACAAGGTTTTATTTCATTTTG 120
armadillo TTTAGATATGAATATCAACTGACACCCCTGACAGGCCATACAAGGTTTTATTTCATTTTG 120
chimp TTTAGATATGAATATCAACTGACACCCCTGACAGGCCATACAAGGTTTTATTTCATTTTG 120
human TTTAGATATGAATATCAACTGACACCCCTGACAGGCCATACAAGGTTTTATTTCATTTTG 120
elephant TTTAGATATGAATATCAACTGACACCCCTGACAGGCCATACAAGGTTTTATTTCATTTTG 120
dog TTTAGATATGAATATCAACTGACACCCCTGACAGGCCATACAAGGTTTTATTTCATTTTG 120
rat TTTAGATATGAATATCAACTGACACCCCTGACAGGCCATACAAGGCTTTATTTCATTTTG 120
mouse TTTAGATATGAATATCAACTGACACCCCTGACAGGCCATACAAGGTTTTATTTCATTTTG 120
cow TTTAGATATGAATATCAACTGACACCCCTGACAGGCCATACAAGGTTTTATTTCATTTTG 120
chicken TTTAGATAGGAATATCAACTGACACCCCTGACAGGCCATACAAGGTTTTATTTCATTTTG 120
opossum TTTAGATATGAATATCAACTGACACCCTTGACAAGCCATACAAGGTTTTATTTCATTTTC 120
frog TTTAGATATGAATATCAACTGACACCAGTGACAGGCCGTACAAGGTTTTATTTCATTTTG 120
shark TTTAGATATGAATATCAACTGACACCTTTGACAGGCCATACAAGGTTTTATTTTATTTTG 120
zfish TTTAGATATGAATATAAAACGACATTGCTGACAGTCCACACAAGGTTAGATTTCATTTCT 120
 * ***** ****** ** **** ***** ** ****** * **** ****

## Slide 77
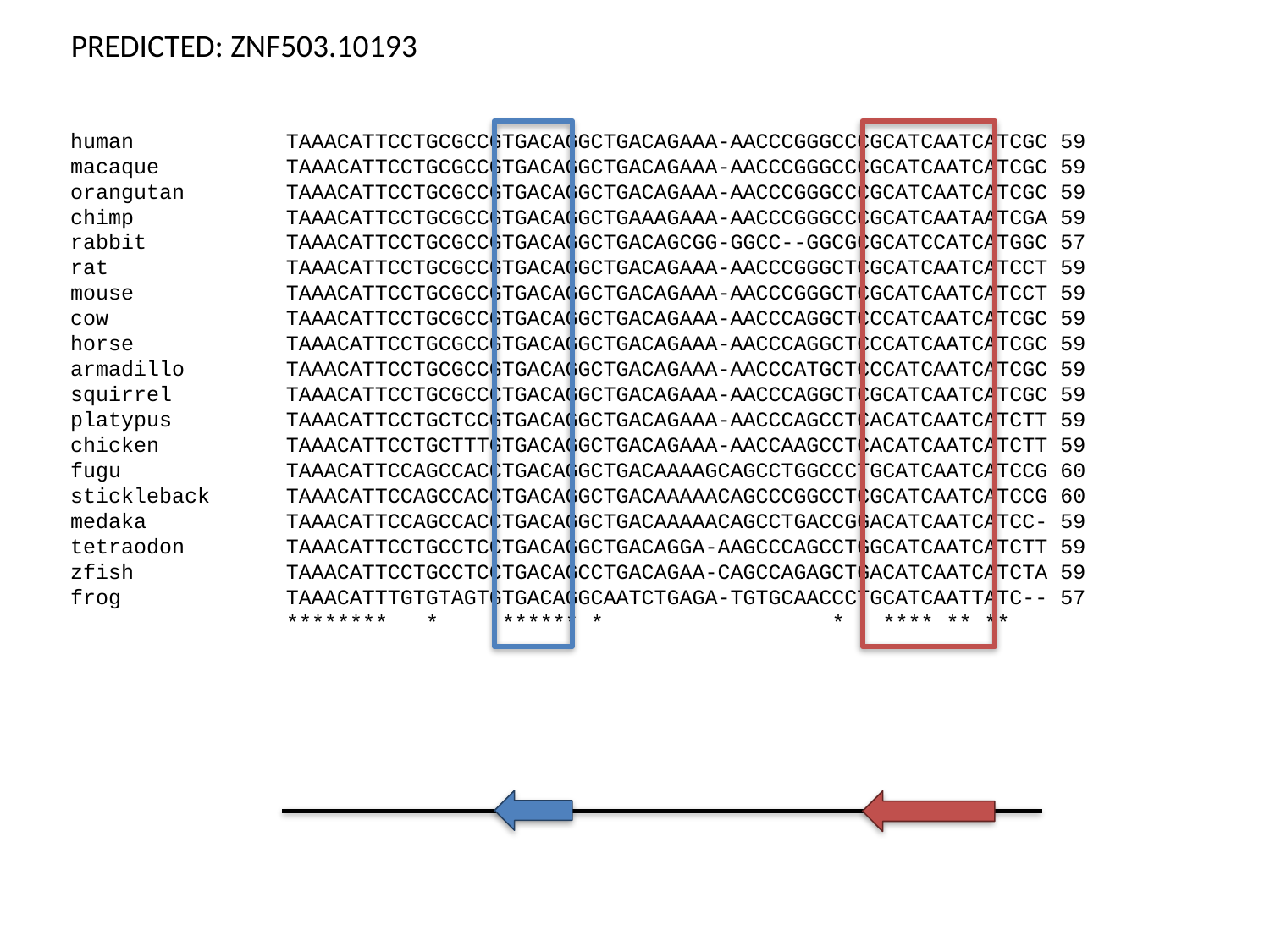

PREDICTED: ZNF503.10193
human TAAACATTCCTGCGCCGTGACAGGCTGACAGAAA-AACCCGGGCCCGCATCAATCATCGC 59
macaque TAAACATTCCTGCGCCGTGACAGGCTGACAGAAA-AACCCGGGCCCGCATCAATCATCGC 59
orangutan TAAACATTCCTGCGCCGTGACAGGCTGACAGAAA-AACCCGGGCCCGCATCAATCATCGC 59
chimp TAAACATTCCTGCGCCGTGACAGGCTGAAAGAAA-AACCCGGGCCCGCATCAATAATCGA 59
rabbit TAAACATTCCTGCGCCGTGACAGGCTGACAGCGG-GGCC--GGCGCGCATCCATCATGGC 57
rat TAAACATTCCTGCGCCGTGACAGGCTGACAGAAA-AACCCGGGCTCGCATCAATCATCCT 59
mouse TAAACATTCCTGCGCCGTGACAGGCTGACAGAAA-AACCCGGGCTCGCATCAATCATCCT 59
cow TAAACATTCCTGCGCCGTGACAGGCTGACAGAAA-AACCCAGGCTCCCATCAATCATCGC 59
horse TAAACATTCCTGCGCCGTGACAGGCTGACAGAAA-AACCCAGGCTCCCATCAATCATCGC 59
armadillo TAAACATTCCTGCGCCGTGACAGGCTGACAGAAA-AACCCATGCTCCCATCAATCATCGC 59
squirrel TAAACATTCCTGCGCCCTGACAGGCTGACAGAAA-AACCCAGGCTCGCATCAATCATCGC 59
platypus TAAACATTCCTGCTCCGTGACAGGCTGACAGAAA-AACCCAGCCTCACATCAATCATCTT 59
chicken TAAACATTCCTGCTTTGTGACAGGCTGACAGAAA-AACCAAGCCTCACATCAATCATCTT 59
fugu TAAACATTCCAGCCACCTGACAGGCTGACAAAAGCAGCCTGGCCCTGCATCAATCATCCG 60
stickleback TAAACATTCCAGCCACCTGACAGGCTGACAAAAACAGCCCGGCCTCGCATCAATCATCCG 60
medaka TAAACATTCCAGCCACCTGACAGGCTGACAAAAACAGCCTGACCGGACATCAATCATCC- 59
tetraodon TAAACATTCCTGCCTCCTGACAGGCTGACAGGA-AAGCCCAGCCTGGCATCAATCATCTT 59
zfish TAAACATTCCTGCCTCCTGACAGCCTGACAGAA-CAGCCAGAGCTGACATCAATCATCTA 59
frog TAAACATTTGTGTAGTGTGACAGGCAATCTGAGA-TGTGCAACCCTGCATCAATTATC-- 57
 ******** * ****** * * **** ** **

## Slide 78
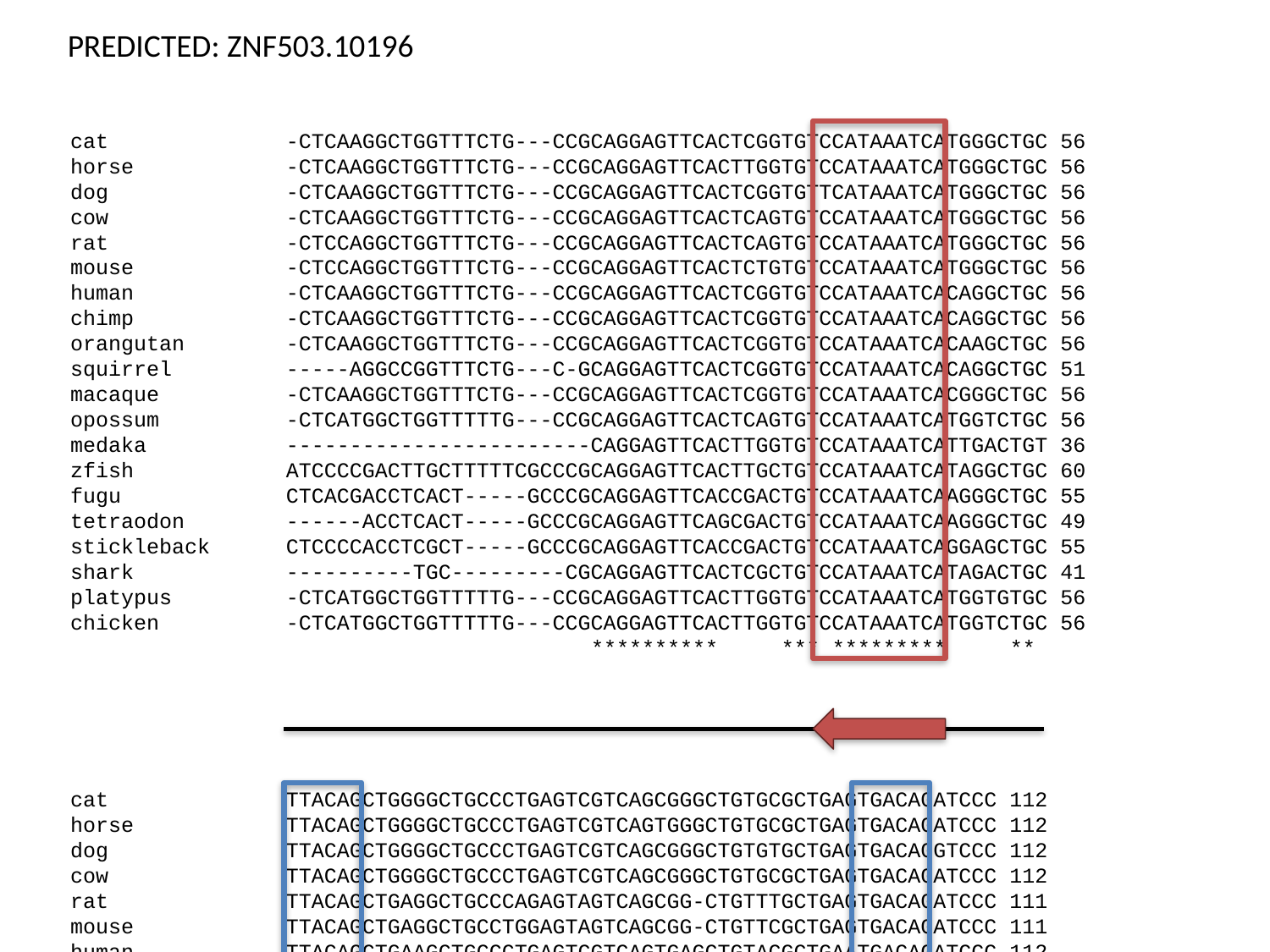

PREDICTED: ZNF503.10196
cat -CTCAAGGCTGGTTTCTG---CCGCAGGAGTTCACTCGGTGTCCATAAATCATGGGCTGC 56
horse -CTCAAGGCTGGTTTCTG---CCGCAGGAGTTCACTTGGTGTCCATAAATCATGGGCTGC 56
dog -CTCAAGGCTGGTTTCTG---CCGCAGGAGTTCACTCGGTGTTCATAAATCATGGGCTGC 56
cow -CTCAAGGCTGGTTTCTG---CCGCAGGAGTTCACTCAGTGTCCATAAATCATGGGCTGC 56
rat -CTCCAGGCTGGTTTCTG---CCGCAGGAGTTCACTCAGTGTCCATAAATCATGGGCTGC 56
mouse -CTCCAGGCTGGTTTCTG---CCGCAGGAGTTCACTCTGTGTCCATAAATCATGGGCTGC 56
human -CTCAAGGCTGGTTTCTG---CCGCAGGAGTTCACTCGGTGTCCATAAATCACAGGCTGC 56
chimp -CTCAAGGCTGGTTTCTG---CCGCAGGAGTTCACTCGGTGTCCATAAATCACAGGCTGC 56
orangutan -CTCAAGGCTGGTTTCTG---CCGCAGGAGTTCACTCGGTGTCCATAAATCACAAGCTGC 56
squirrel -----AGGCCGGTTTCTG---C-GCAGGAGTTCACTCGGTGTCCATAAATCACAGGCTGC 51
macaque -CTCAAGGCTGGTTTCTG---CCGCAGGAGTTCACTCGGTGTCCATAAATCACGGGCTGC 56
opossum -CTCATGGCTGGTTTTTG---CCGCAGGAGTTCACTCAGTGTCCATAAATCATGGTCTGC 56
medaka ------------------------CAGGAGTTCACTTGGTGTCCATAAATCATTGACTGT 36
zfish ATCCCCGACTTGCTTTTTCGCCCGCAGGAGTTCACTTGCTGTCCATAAATCATAGGCTGC 60
fugu CTCACGACCTCACT-----GCCCGCAGGAGTTCACCGACTGTCCATAAATCAAGGGCTGC 55
tetraodon ------ACCTCACT-----GCCCGCAGGAGTTCAGCGACTGTCCATAAATCAAGGGCTGC 49
stickleback CTCCCCACCTCGCT-----GCCCGCAGGAGTTCACCGACTGTCCATAAATCAGGAGCTGC 55
shark ----------TGC---------CGCAGGAGTTCACTCGCTGTCCATAAATCATAGACTGC 41
platypus -CTCATGGCTGGTTTTTG---CCGCAGGAGTTCACTTGGTGTCCATAAATCATGGTGTGC 56
chicken -CTCATGGCTGGTTTTTG---CCGCAGGAGTTCACTTGGTGTCCATAAATCATGGTCTGC 56
 ********** *** ********* **
cat TTACAGCTGGGGCTGCCCTGAGTCGTCAGCGGGCTGTGCGCTGAGTGACAGATCCC 112
horse TTACAGCTGGGGCTGCCCTGAGTCGTCAGTGGGCTGTGCGCTGAGTGACAGATCCC 112
dog TTACAGCTGGGGCTGCCCTGAGTCGTCAGCGGGCTGTGTGCTGAGTGACAGGTCCC 112
cow TTACAGCTGGGGCTGCCCTGAGTCGTCAGCGGGCTGTGCGCTGAGTGACAGATCCC 112
rat TTACAGCTGAGGCTGCCCAGAGTAGTCAGCGG-CTGTTTGCTGAGTGACAGATCCC 111
mouse TTACAGCTGAGGCTGCCTGGAGTAGTCAGCGG-CTGTTCGCTGAGTGACAGATCCC 111
human TTACAGCTGAAGCTGCCCTGAGTCGTCAGTGAGCTGTACGCTGAATGACAGATCCC 112
chimp TTACAGCTGAAGCTGCCCTGAGTCGTCAGTGAGCTGTACGCTGAATGACAGATCCC 112
orangutan TTACAGCTGAAGCTGCCCTGAGTCGTCAGTGAGCTGTACGCTGAGTGACAGATCCC 112
squirrel TTACAGCCCAAGCTGCCCTCAGTCGTCAGCGGGCTGGACGTTGAGTGACAGATCCC 107
macaque TTACAGCTGAAGCTGCCCTGAGTCGTCAGCGAGGTGTACGCTGAGTGACAGATCCC 112
opossum TTACAGCTGAAACTGCCCTGAGTCATCAGCATGCTGGAAGCTGAGTGACAGATTCC 112
medaka TTACAGCTGAAACCACCCTTAGTCATCAACATGCAATACACTGAGTGACAGATTCC 92
zfish TTACAGCTCAAACAGCCCTGAGTCATCAACATGCAATAAGCTGAGTGACAGATTCC 116
fugu TTACAGCTGAAACTACCCTGAGTCATCAGCATGCAATAAGCAGACTGACAGATTCC 111
tetraodon TTACAGCTGAAACTACCCTGAGTCATCAACATGCAGTAAGCAGACTGACAGATTCC 105
stickleback TTACAGCCAGAACTACCCTGAGTCATCATCGTGCAATAAGCTGAGTGGCAGAT--- 108
shark TTACAGCTGAAACTGCACTGAGTCATCAACATGCAATAAGCTGAGTGACAGATCCC 97
platypus TTACAGCTGAAACTGCACTGAGTCATCAACATGCAATAAGCTGAGTGACAGATTCC 112
chicken TTACAGCTGAAACTGCACTGAGTCATCAACATGCAATAAGCTGAGTGACAGATTCC 112
 ******* * * *** *** ** ** *** *

## Slide 79
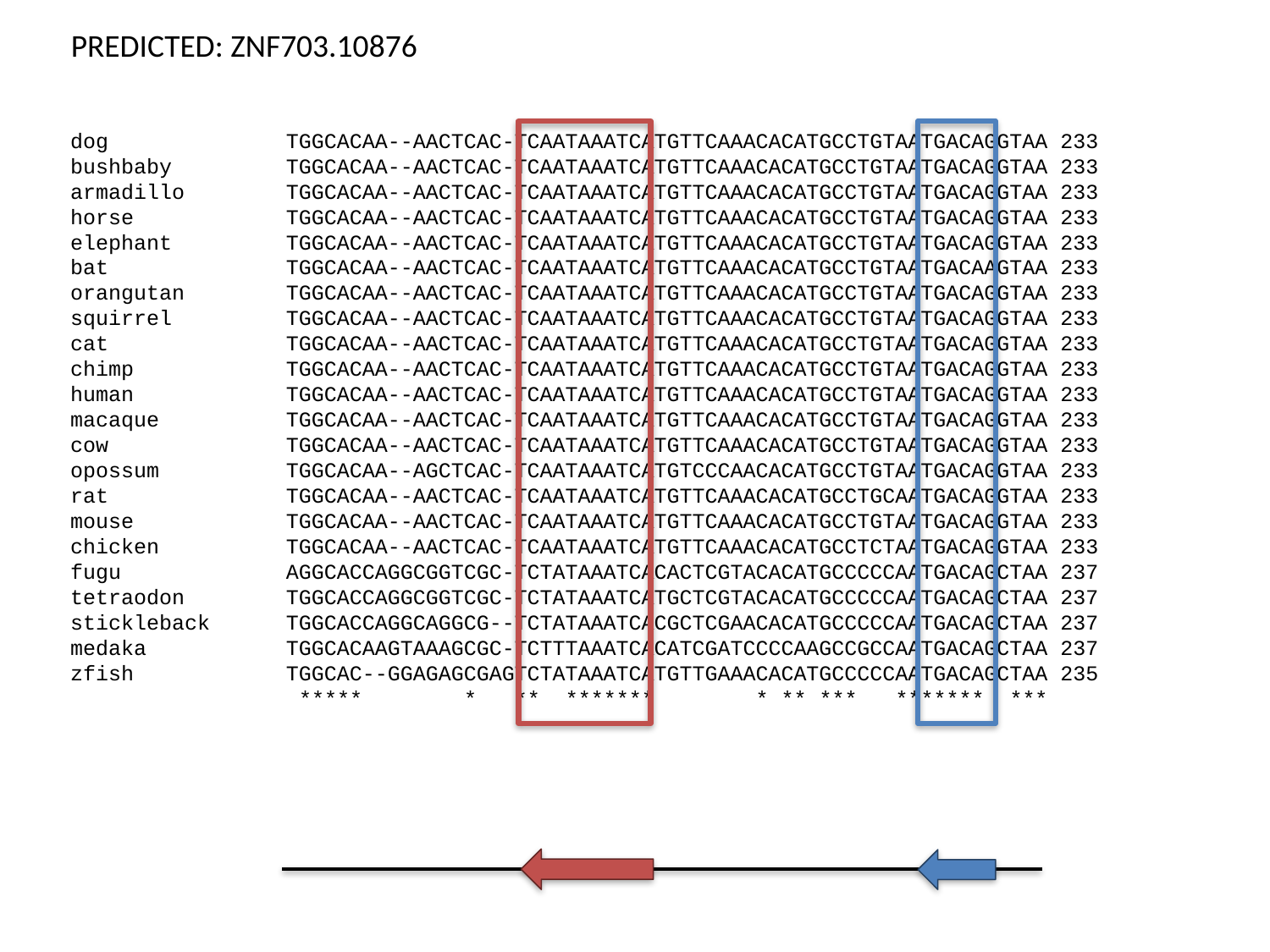

PREDICTED: ZNF703.10876
dog TGGCACAA--AACTCAC-TCAATAAATCATGTTCAAACACATGCCTGTAATGACAGGTAA 233
bushbaby TGGCACAA--AACTCAC-TCAATAAATCATGTTCAAACACATGCCTGTAATGACAGGTAA 233
armadillo TGGCACAA--AACTCAC-TCAATAAATCATGTTCAAACACATGCCTGTAATGACAGGTAA 233
horse TGGCACAA--AACTCAC-TCAATAAATCATGTTCAAACACATGCCTGTAATGACAGGTAA 233
elephant TGGCACAA--AACTCAC-TCAATAAATCATGTTCAAACACATGCCTGTAATGACAGGTAA 233
bat TGGCACAA--AACTCAC-TCAATAAATCATGTTCAAACACATGCCTGTAATGACAAGTAA 233
orangutan TGGCACAA--AACTCAC-TCAATAAATCATGTTCAAACACATGCCTGTAATGACAGGTAA 233
squirrel TGGCACAA--AACTCAC-TCAATAAATCATGTTCAAACACATGCCTGTAATGACAGGTAA 233
cat TGGCACAA--AACTCAC-TCAATAAATCATGTTCAAACACATGCCTGTAATGACAGGTAA 233
chimp TGGCACAA--AACTCAC-TCAATAAATCATGTTCAAACACATGCCTGTAATGACAGGTAA 233
human TGGCACAA--AACTCAC-TCAATAAATCATGTTCAAACACATGCCTGTAATGACAGGTAA 233
macaque TGGCACAA--AACTCAC-TCAATAAATCATGTTCAAACACATGCCTGTAATGACAGGTAA 233
cow TGGCACAA--AACTCAC-TCAATAAATCATGTTCAAACACATGCCTGTAATGACAGGTAA 233
opossum TGGCACAA--AGCTCAC-TCAATAAATCATGTCCCAACACATGCCTGTAATGACAGGTAA 233
rat TGGCACAA--AACTCAC-TCAATAAATCATGTTCAAACACATGCCTGCAATGACAGGTAA 233
mouse TGGCACAA--AACTCAC-TCAATAAATCATGTTCAAACACATGCCTGTAATGACAGGTAA 233
chicken TGGCACAA--AACTCAC-TCAATAAATCATGTTCAAACACATGCCTCTAATGACAGGTAA 233
fugu AGGCACCAGGCGGTCGC-TCTATAAATCACACTCGTACACATGCCCCCAATGACAGCTAA 237
tetraodon TGGCACCAGGCGGTCGC-TCTATAAATCATGCTCGTACACATGCCCCCAATGACAGCTAA 237
stickleback TGGCACCAGGCAGGCG--TCTATAAATCACGCTCGAACACATGCCCCCAATGACAGCTAA 237
medaka TGGCACAAGTAAAGCGC-TCTTTAAATCACATCGATCCCCAAGCCGCCAATGACAGCTAA 237
zfish TGGCAC--GGAGAGCGAGTCTATAAATCATGTTGAAACACATGCCCCCAATGACAGCTAA 235
 ***** * ** ******* * ** *** ******* ***

## Slide 80
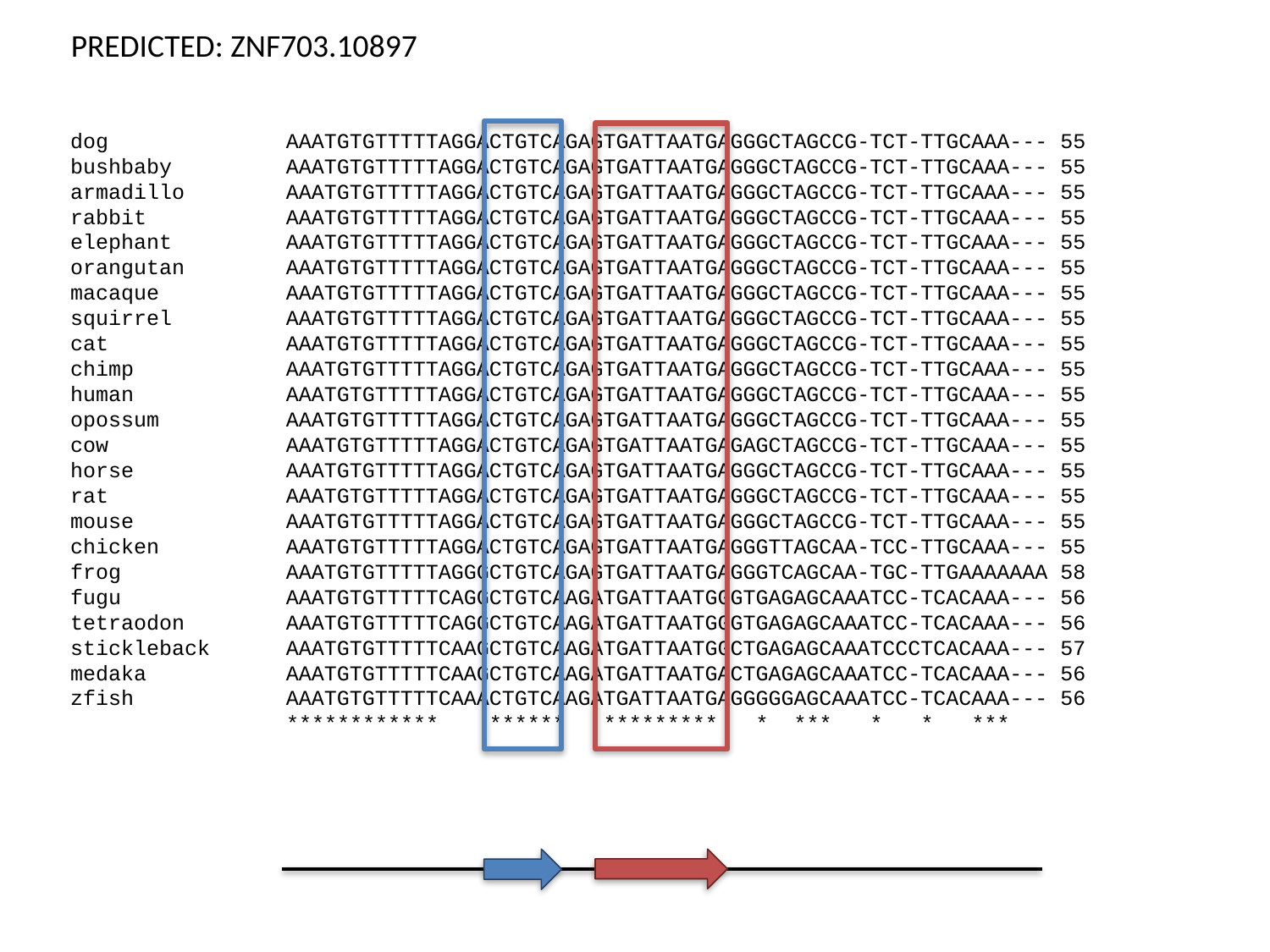

PREDICTED: ZNF703.10897
dog AAATGTGTTTTTAGGACTGTCAGAGTGATTAATGAGGGCTAGCCG-TCT-TTGCAAA--- 55
bushbaby AAATGTGTTTTTAGGACTGTCAGAGTGATTAATGAGGGCTAGCCG-TCT-TTGCAAA--- 55
armadillo AAATGTGTTTTTAGGACTGTCAGAGTGATTAATGAGGGCTAGCCG-TCT-TTGCAAA--- 55
rabbit AAATGTGTTTTTAGGACTGTCAGAGTGATTAATGAGGGCTAGCCG-TCT-TTGCAAA--- 55
elephant AAATGTGTTTTTAGGACTGTCAGAGTGATTAATGAGGGCTAGCCG-TCT-TTGCAAA--- 55
orangutan AAATGTGTTTTTAGGACTGTCAGAGTGATTAATGAGGGCTAGCCG-TCT-TTGCAAA--- 55
macaque AAATGTGTTTTTAGGACTGTCAGAGTGATTAATGAGGGCTAGCCG-TCT-TTGCAAA--- 55
squirrel AAATGTGTTTTTAGGACTGTCAGAGTGATTAATGAGGGCTAGCCG-TCT-TTGCAAA--- 55
cat AAATGTGTTTTTAGGACTGTCAGAGTGATTAATGAGGGCTAGCCG-TCT-TTGCAAA--- 55
chimp AAATGTGTTTTTAGGACTGTCAGAGTGATTAATGAGGGCTAGCCG-TCT-TTGCAAA--- 55
human AAATGTGTTTTTAGGACTGTCAGAGTGATTAATGAGGGCTAGCCG-TCT-TTGCAAA--- 55
opossum AAATGTGTTTTTAGGACTGTCAGAGTGATTAATGAGGGCTAGCCG-TCT-TTGCAAA--- 55
cow AAATGTGTTTTTAGGACTGTCAGAGTGATTAATGAGAGCTAGCCG-TCT-TTGCAAA--- 55
horse AAATGTGTTTTTAGGACTGTCAGAGTGATTAATGAGGGCTAGCCG-TCT-TTGCAAA--- 55
rat AAATGTGTTTTTAGGACTGTCAGAGTGATTAATGAGGGCTAGCCG-TCT-TTGCAAA--- 55
mouse AAATGTGTTTTTAGGACTGTCAGAGTGATTAATGAGGGCTAGCCG-TCT-TTGCAAA--- 55
chicken AAATGTGTTTTTAGGACTGTCAGAGTGATTAATGAGGGTTAGCAA-TCC-TTGCAAA--- 55
frog AAATGTGTTTTTAGGGCTGTCAGAGTGATTAATGAGGGTCAGCAA-TGC-TTGAAAAAAA 58
fugu AAATGTGTTTTTCAGGCTGTCAAGATGATTAATGGGTGAGAGCAAATCC-TCACAAA--- 56
tetraodon AAATGTGTTTTTCAGGCTGTCAAGATGATTAATGGGTGAGAGCAAATCC-TCACAAA--- 56
stickleback AAATGTGTTTTTCAAGCTGTCAAGATGATTAATGGCTGAGAGCAAATCCCTCACAAA--- 57
medaka AAATGTGTTTTTCAAGCTGTCAAGATGATTAATGACTGAGAGCAAATCC-TCACAAA--- 56
zfish AAATGTGTTTTTCAAACTGTCAAGATGATTAATGAGGGGGAGCAAATCC-TCACAAA--- 56
 ************ ****** ********* * *** * * ***

## Slide 81
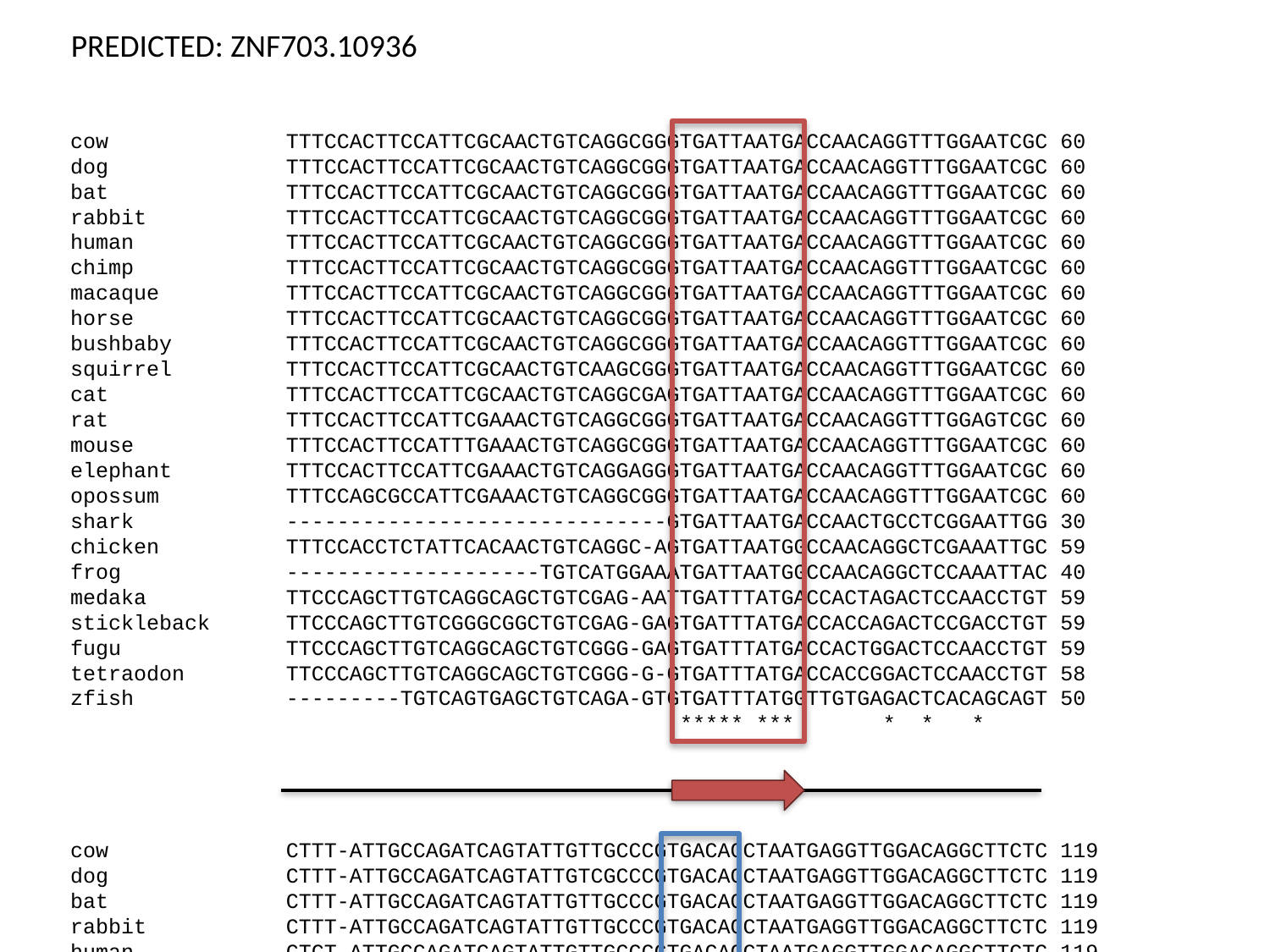

PREDICTED: ZNF703.10936
cow TTTCCACTTCCATTCGCAACTGTCAGGCGGGTGATTAATGACCAACAGGTTTGGAATCGC 60
dog TTTCCACTTCCATTCGCAACTGTCAGGCGGGTGATTAATGACCAACAGGTTTGGAATCGC 60
bat TTTCCACTTCCATTCGCAACTGTCAGGCGGGTGATTAATGACCAACAGGTTTGGAATCGC 60
rabbit TTTCCACTTCCATTCGCAACTGTCAGGCGGGTGATTAATGACCAACAGGTTTGGAATCGC 60
human TTTCCACTTCCATTCGCAACTGTCAGGCGGGTGATTAATGACCAACAGGTTTGGAATCGC 60
chimp TTTCCACTTCCATTCGCAACTGTCAGGCGGGTGATTAATGACCAACAGGTTTGGAATCGC 60
macaque TTTCCACTTCCATTCGCAACTGTCAGGCGGGTGATTAATGACCAACAGGTTTGGAATCGC 60
horse TTTCCACTTCCATTCGCAACTGTCAGGCGGGTGATTAATGACCAACAGGTTTGGAATCGC 60
bushbaby TTTCCACTTCCATTCGCAACTGTCAGGCGGGTGATTAATGACCAACAGGTTTGGAATCGC 60
squirrel TTTCCACTTCCATTCGCAACTGTCAAGCGGGTGATTAATGACCAACAGGTTTGGAATCGC 60
cat TTTCCACTTCCATTCGCAACTGTCAGGCGAGTGATTAATGACCAACAGGTTTGGAATCGC 60
rat TTTCCACTTCCATTCGAAACTGTCAGGCGGGTGATTAATGACCAACAGGTTTGGAGTCGC 60
mouse TTTCCACTTCCATTTGAAACTGTCAGGCGGGTGATTAATGACCAACAGGTTTGGAATCGC 60
elephant TTTCCACTTCCATTCGAAACTGTCAGGAGGGTGATTAATGACCAACAGGTTTGGAATCGC 60
opossum TTTCCAGCGCCATTCGAAACTGTCAGGCGGGTGATTAATGACCAACAGGTTTGGAATCGC 60
shark ------------------------------GTGATTAATGACCAACTGCCTCGGAATTGG 30
chicken TTTCCACCTCTATTCACAACTGTCAGGC-AGTGATTAATGGCCAACAGGCTCGAAATTGC 59
frog --------------------TGTCATGGAAATGATTAATGGCCAACAGGCTCCAAATTAC 40
medaka TTCCCAGCTTGTCAGGCAGCTGTCGAG-AATTGATTTATGACCACTAGACTCCAACCTGT 59
stickleback TTCCCAGCTTGTCGGGCGGCTGTCGAG-GAGTGATTTATGACCACCAGACTCCGACCTGT 59
fugu TTCCCAGCTTGTCAGGCAGCTGTCGGG-GAGTGATTTATGACCACTGGACTCCAACCTGT 59
tetraodon TTCCCAGCTTGTCAGGCAGCTGTCGGG-G-GTGATTTATGACCACCGGACTCCAACCTGT 58
zfish ---------TGTCAGTGAGCTGTCAGA-GTGTGATTTATGGTTGTGAGACTCACAGCAGT 50
 ***** *** * * *
cow CTTT-ATTGCCAGATCAGTATTGTTGCCCGTGACAGCTAATGAGGTTGGACAGGCTTCTC 119
dog CTTT-ATTGCCAGATCAGTATTGTCGCCCGTGACAGCTAATGAGGTTGGACAGGCTTCTC 119
bat CTTT-ATTGCCAGATCAGTATTGTTGCCCGTGACAGCTAATGAGGTTGGACAGGCTTCTC 119
rabbit CTTT-ATTGCCAGATCAGTATTGTTGCCCGTGACAGCTAATGAGGTTGGACAGGCTTCTC 119
human CTCT-ATTGCCAGATCAGTATTGTTGCCCCTGACAGCTAATGAGGTTGGACAGGCTTCTC 119
chimp CTCT-ATTGCCAGATCAGTATTGTTGCCCCTGACAGCTAATGAGGTTGGACAGGCTTCTC 119
macaque CTCT-ATTGCCAGATCAGTATTGTTGCCCGTGACAGCTAATGAGGTTGGA---------- 109
horse CTTT-ATTGCCAGATCAGTATTGTTGCCCATGACAGCTAATGAGGTTGGACAGGCTTCTC 119
bushbaby CTTT-ATTGCCAGATCAGTATTGTTGCCTGTGACAGCTAATGAGGTTGGACAGGCTTCTC 119
squirrel CTTT-ATTGCCAGATCAGTATTGTTGCCCGTGACAGCTAATGAGGTTGGACAGGCTTCTC 119
cat CTTT-ATTGCCAGATCAGTATTGTTGCCCGTGACAGCTAATGAGGTTGGACAGGCTTCTC 119
rat CTTTTATTGCCAGATCAGTATTGTTGCCCGTGACAGCTAATGAGGTTGGACAGGCTTCTC 120
mouse CTTTTATTGCCAGATCAGTATTGTTGCCCGTGACAGCTAATGAGGTTGGACAGGCTTCTC 120
elephant CTTT-ATTGCCAGATCAGTATTGTTGCCCGTGACAGCTAATGAGGTTGGACAGGCTTCTC 119
opossum CTTT-ATTGCCAGATCAGTATTGTTGCCCGTGACAGCTAATGAGGTTGGACAGGCTTCTC 119
shark CTTT-ATACTCAACTCCGCATTGTTTGCGCTGACAGTTAATGAGGTTAGACATACTT--- 86
chicken CTTT-ATTGCCAGCTTGGTATTGTTACTTGTGACAGCTAATGAGGTTGGACAGACTTTTA 118
frog CTTC-ATTGTCAGCTCTGTATTGTTAGTTCTGACAACTAATGAGGTCCGACAG------- 92
medaka CTTC-ACTGCCAGTTTGGTATTGTTGCAACTGACAGCTAATGAGGCCAGACATGCTTTCA 118
stickleback CTTC-ACTGCCAGTTCGCCATTGTTACAGCTGACAGCTAATGAGGCCAGACATGCTTTCA 118
fugu CTTC-ACTGCCAGCTCAGTATTGTTACAACTGACAGCTAATGAGGCCAGACATGCTTTCA 118
tetraodon CTTC-GCTGCCAGCTCAGTATTGTTACAACTGACAGCTAATGAGGCCAGACATGCTTTCA 117
zfish CCTT-GCTGCCAGTTCAGTATTGTTACAACTGACATCTAATGAGGCCAGACGTGCTTTCA 109
 * ** * ***** ***** ******** **
